# Erythronate utilization activates VdtR regulating its metabolism to promote *Brucella* proliferation, inducing abortion in mice

**DOI:** 10.1128/spectrum.02074-23

**Published:** 2023-09-06

**Authors:** Yi Yin, Tian Fang, Zhengmin Lian, Dong Zuo, Hai Hu, Guangdong Zhang, Chan Ding, Mingxing Tian, Shengqing Yu

**Affiliations:** 1 Shanghai Veterinary Research Institute, Chinese Academy of Agricultural Sciences (CAAS), Shanghai, China; 2 Veterinary Bio-Pharmaceutical, Jiangsu Agri-Animal Husbandry Vocational College, Jiangsu Key Laboratory for High-Tech Research and Development of Veterinary Biopharmaceuticals, Taizhou, Jiangsu, China; Yangzhou University, Yangzhou, Jiangsu, China

**Keywords:** *Brucella*, VdtR regulation, erythronate metabolic pathway, abortion

## Abstract

*Brucella* is a facultative intracellular pathogen that preferentially colonizes reproductive organs and utilizes erythritol as a preferred carbon source for its survival and proliferation. In this study, we identified a virulence-related DeoR-family transcriptional regulator (VdtR) and an erythronate metabolic pathway responsible for four-carbon acid sugar metabolism of D-erythronate and L-threonate in *Brucella*. We found that VdtR plays an important role in *Brucella* intracellular survival and trafficking to the endoplasmic reticulum in RAW 264.7 macrophages and in virulence in a mouse model. More importantly, we found that VdtR negatively regulates the erythronate metabolic pathway to promote extracellular proliferation of *Brucella*, depending on utilization of D-erythronate, an oxidative product of erythritol in the host. In a pregnant mouse model, the erythronate metabolic pathway was shown to cooperate with erythritol metabolism and play a crucial role in *Brucella* proliferation in the placenta, inducing placentitis and finally resulting in abortion or stillbirth. Our results demonstrate that, in addition to erythritol, erythronate is a preferred carbon source for *Brucella* utilization to promote its extracellular proliferation. This discovery updates the information on the preferential colonization of reproductive organs by *Brucella* and provides a novel insight into the *Brucella*-associated induction of abortion in pregnant animals.

**IMPORTANCE:**

*Brucella* is an intracellular parasitic bacterium causing zoonosis, which is distributed worldwide and mainly characterized by reproductive disorders. Erythritol is found in allantoic fluid, chorion, and placenta of aborted animals, preferentially utilized by *Brucella* to cause infertility and abortion. However, the erythritol metabolism-defected mutant was unable to function as a vaccine strain due to its residual virulence. Here, we found that erythronate, an oxidative product of erythritol in the host, was also preferentially utilized by *Brucella* relying on the function of a deoxyribonucleoside regulator-family transcriptional regulator VdtR. Erythronate utilization activates VdtR regulation of the erythronate metabolic pathway to promote *Brucella* extracellular proliferation, inducing placentitis/abortion in mice. Double mutations on *Brucella* erythritol and D-erythronate metabolisms significantly reduced bacterial virulence. This study revealed a novel mechanism of *Brucella* infection-induced abortion, thus providing a new clue for the study of safer *Brucella* attenuated vaccines.

## INTRODUCTION

*Brucella* spp. are the etiological agents of brucellosis, a zoonosis with worldwide distribution. A relevant symptomatology of this disease is related to the particular tropism of the pathogen for the reproductive tract of ungulates and swine, which results in orchitis, epididymitis, abortion, and infertility (1, 2). *Brucella*, a facultative intracellular pathogen, has no classic virulence factors such as exotoxins, cytolysins, and exoenzymes, and its virulence depends on the ability to survive and replicate within host cells (3). The metabolic network and substrates used by bacteria within their host are a fundamental aspect of pathogenic or symbiotic lifestyles. Hence, *Brucella* spp. are able to fine-tune their metabolism in response to the extra- and intracellular environments encountered during an infectious cycle (4, 5). However, this aspect of *Brucella* biology is not fully understood, and the nutrients available in the intracellular niche are not well known.

Erythritol, a four-carbon polyol, is a preferred carbon source for *Brucella*. It is well accepted that *Brucella* tropism and extensive multiplication are associated with high concentrations of this four-carbon polyol in the genital organs of host animals (6). Erythritol can promote *Brucella* growth at low concentrations (7). Smith et al. described that cow allantoic fluid, chorion, and placenta can produce high concentrations of erythritol, suggesting this to be the reason for the presence of *Brucella* in bovine abortion (8). Erythritol is usually utilized by *Brucella abortus* via a five-step erythritol metabolic pathway (9) in which EryA presents eight times higher activity than glucokinase, providing a possible explanation for the preferred utilization of erythritol over glucose *in vitro* (10). However, the preferential colonization of reproductive organs by *Brucella* is not fully understood. Some controversial observations focus on the role of erythritol in *Brucella* colonization. The *B. abortus* vaccine strain S19 is sensitive to erythritol and can cause abortion in cattle (11). However, *Brucella ovis* and *Brucella canis* establish genital infections and cause abortion in sheep and dogs, which are unable to catabolize erythritol (12, 13). These observations have led to speculation that erythritol is not the only factor involved in *Brucella* colonization. Other nutrients found in abundance in genital organs are also available for *Brucella* to utilize, including glycerol, lactate, and glutamate (14).

Previously, a deoxyribonucleoside regulator (DeoR)-family transcriptional regulator encoded by *BAB_RS27040* (old locus *BAB2_0143*) was identified and linked to *B. abortus* virulence (15 – 19), which was designated as virulence-associated DeoR-family transcriptional regulator (VdtR) in this study. VdtR has a typical N-terminal DNA-binding domain and a C-terminal sugar-binding domain. DeoR-type regulators usually act as repressors in sugar metabolism (20). DeoR from *Bacillus subtilis* negatively regulates the expression of enzymes involved in the catabolism of deoxyribonucleosides and deoxyribose (21). In *Escherichia coli*, YciT, a DeoR-type global regulator, inhibits the expression of a number of genes involved in various metabolic pathways, including transport of maltose, fatty acid beta-oxidation, and degradation of peptides (22). The DeoR-type regulator SugR in *Corynebacterium glutamicum* acts as a repressor for genes in the phosphoenolpyruvate-sugar phosphotransferase system (20). However, the role of VdtR in *Brucella* metabolism or virulence has not been defined.

In this study, we found that VdtR negatively regulates the expression of a novel gene cluster, a homolog of the aldolase-DUF1537 gene cluster, involved in the metabolism of the four-carbon acid sugars D-erythronate and L-threonate (named as erythronate metabolic pathway thereafter). Furthermore, the erythronate metabolic pathway was identified to coordinate with the erythritol metabolic pathway and played a crucial role in *Brucella* proliferation in placenta, inducing placentitis and abortion in pregnant mice. This study provides novel insights into *Brucella* tropism to genital organs and virulence.

## RESULTS

### A novel DeoR-family transcriptional regulator, VdtR, plays an important role in *Brucella* intracellular survival, trafficking, and *Brucella* pathogenicity to mice

To investigate the role of VdtR in *Brucella* survival, trafficking and virulence, we first constructed a *vdtR* deletion mutant (2308Δ*vdtR*) and its revertant mutant (2308Δ*vdtR*-Rev) using *B. abortus* strain 2308. Growth curve analysis showed that the VdtR deletion did not affect *Brucella* growth in tryptic soy broth (TSB) (Fig. S1A), nor the lipopolysaccharide pattern in detection of SDS-PAGE followed by silver staining (Fig. S1B). We further evaluated the resistance of 2308Δ*vdtR* to hydrogen peroxide, polymyxin B, normal guinea pig serum, and sodium nitroprusside (SNP) and found that deletion of the *vdtR* gene did not affect bacterial resistance to these bactericidal factors (Fig. S1C and D). These results indicated that *vdtR* was not associated with the regulation of *Brucella* phenotype and resistance.

**FIG 1:**
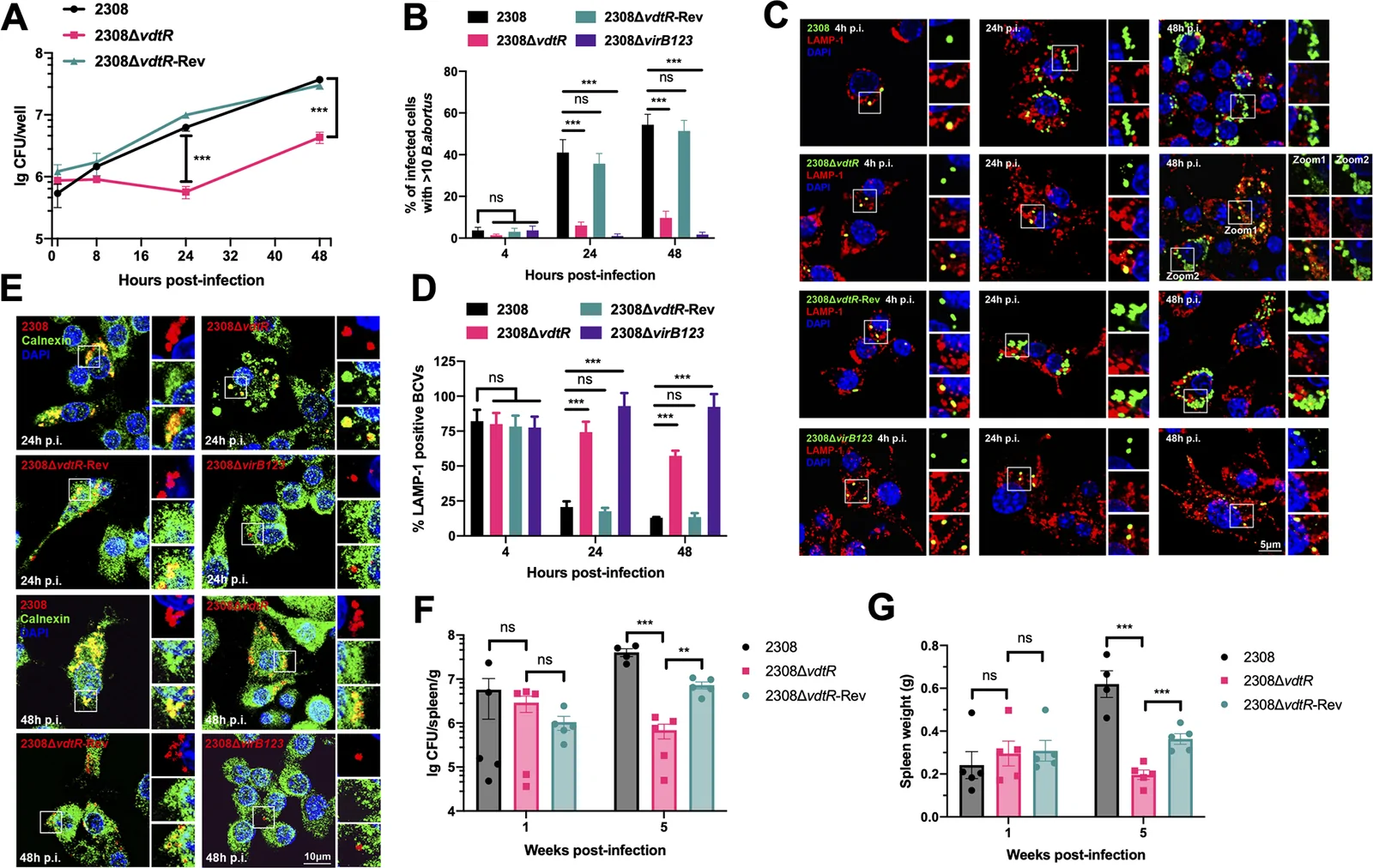
VdtR plays an important role in *Brucella* intracellular survival, trafficking, and *Brucella* pathogenicity in mice. (**A**) Intracellular survival of *B. abortus* 2308, mutant 2308Δ*vdtR*, and reverted mutant 2308Δ*vdtR*-Rev in RAW264.7 macrophages. Cells were infected and intracellular CFUs were enumerated at 1, 8, 24, and 48 h post-infection (p.i.). Data shown are means ± SDs from a representative experiment performed in triplicate (*N* = 3, two-way analysis of variance [ANOVA]). (**B**) The proportion of cells with more than 10 *B. abortus* per 100 infected cells (*N* = 3, mean ± SD, two-way ANOVA). (**C**) Representative image of co-localization of *Brucella* with lysosomal-associated membrane protein 1 (LAMP-1) in RAW264.7 macrophages by indirect immunofluorescence. Infected cells were stained with anti-LAMP1 monoclonal antibody (red) to visualize lysosomes. (**D**) The proportion of LAMP-1-positive *Brucella*-containing vacuoles (BCVs) per 100 BCVs (*N* = 3, mean ± SD, two-way ANOVA). (**E**) Representative image of co-localization of *Brucella* with the endoplasmic reticulum (ER) marker calnexin stained with anticalnexin antibody (green) to visualize ER. (**F**) Comparative analysis of bacterial loads in spleen infected with the 2308, 2308Δ*vdtR*, and 2308Δ*vdtR*-Rev strains at 1 and 5 weeks p.i. (*N* = 5, mean ± SD, multiple unpaired *t*-test). (**G**) Comparative analysis of splenomegaly after 2308, 2308Δ*vdtR*, and 2308Δ*vdtR*-Rev infections at 1 and 5 weeks p.i. (*N* = 5, mean ± SD, multiple unpaired *t*-test). **P < 0.01; ***P < 0.001. ns, not significant.

To determine the role of *vdtR* in the survival of *Brucella* within host cells, we evaluated the intracellular survival of strains 2308, 2308Δ*vdtR*, and 2308Δ*vdtR*-Rev at 1, 8, 24, and 48 h post-infection (p.i.) within RAW264.7 macrophages by counting the colony-forming units (CFUs) (Fig. 1A). The CFUs of strain 2308Δ*vdtR* recovered from cells showed no significant difference at 1 and 8 h p.i., but a distinct decrease at 24 and 48 h p.i. in comparison with strain 2308. At these time points, 2308Δ*vdtR*-Rev had recovered its intracellular survival to the level of strain 2308. To visually assess the intracellular survival of strain 2308Δ*vdtR*, we further evaluated the percentage of cells infected with ≥10 *Brucella* cells at 4, 24, and 48 h p.i. (23) (Fig. 1B), and the results showed that there was no significant difference among 2308, 2308Δ*vdtR*, 2308Δ*vdtR*-Rev, and a T4SS deletion mutant control, strain 2308Δ*virB123*, at 4 h p.i., but the percentage of cells infected with 2308Δ*vdtR* showed a significant decrease at 24 and 48 h p.i. compared with strain 2308. At these time points, the 2308Δ*vdtR*-Rev strain had restored its ability to survive relative to 2308, while the control strain 2308Δ*virB123* was defective in ability to survive intracellularly (Fig. 1B). These data suggested that *vdtR* affects the intracellular survival of *B. abortus*. To further analyze the role of VdtR in *Brucella melitensis* and *Brucella suis*, we constructed two *B. melitensis* deletion mutants, 16MΔ*vdtR* and M5Δ*vdtR*, as well as a *B. suis* deletion mutant 1330Δ*vdtR*. The intracellular survival of strains 16MΔ*vdtR*, M5Δ*vdtR*, and 1330Δ*vdtR* also showed an obvious decrease at 24 and 48 h p.i., while their sibling revertant strains had restored ability to survive within macrophages (Fig. S2A through C). These results confirmed that VdtR is required for the intracellular survival of *Brucella* spp.

Defective intracellular survival of *Brucella* is usually due to a failure to escape lysosomal degradation (24). Hence, we further evaluated the co-localization of 2308, 2308Δ*vdtR*, and 2308Δ*vdtR*-Rev with lysosomes at 4, 24, and 48 h p.i. within macrophages; 2308Δ*virB123* was used as a control that was co-localized with lysosome and failed to traffick to and sustain interactions with the endoplasmic reticulum (ER) (23, 25). As shown in Fig. 1C, strain 2308 co-localized with lysosomal-associated membrane protein 1 (LAMP-1) at 4 h p.i. and then excluded the LAMP-1 from *Brucella*-containing vacuoles (BCVs) at 24 hr and 48 h p.i., which is consistent with previous reports (24, 26). Similar to strain 2308, 2308Δ*vdtR* showed co-localization with LAMP-1 at 4 h p.i.; however, most of the mutants failed to exclude LAMP-1 from BCVs at 24 and 48 h p.i. (Fig. 1C, magnified panel 1). Interestingly, we found that some replicative 2308Δ*vdtR* strains excluded LAMP-1 at 48 h p.i. (Fig. 1C, magnified panel 2). The results revealed that, although 2308Δ*vdtR* had a reduced ability for intracellular survival, it showed an obvious trend to proliferate at 48 h p.i. (Fig. 1A). Furthermore, the co-localization of 2308Δ*vdtR*-Rev with LAMP-1 was restored to the level of strain 2308, and strain 2308Δ*virB123* completely co-localized with LAMP-1 at all indicated times. In a further study, we quantitatively monitored the percentage of LAMP-1-positive BCVs (Fig. 1D) and found that ~80% of BCVs in strains 2308, 2308Δ*vdtR*, 2308Δ*vdtR*-Rev, and 2308Δ*virB123* co-localized with the LAMP-1 at 4 h p.i., while only ~20% of those from strains 2308 and 2308Δ*vdtR*-Rev showed co-localization at 24 and 48 h p.i. However, strain 2308Δ*vdtR* exhibited ~75% and ~60% co-localization with LAMP-1 at 24 and 48 h p.i., respectively, showing a significant increase in comparison with 2308 and 2308Δ*vdtR*-Rev. Meanwhile, the replication-defective strain 2308Δ*virB123* showed ~90% co-localization with LAMP-1 at 24 and 48 h p.i., as expected. These results suggested that deletion of *vdtR* affects the escape of *B. abortus* from lysosomal degradation within macrophages. According to previous literature, it is well known that, after escaping lysosomal degradation, *Brucella* traffics to the ER and acquires ER markers at the replicative stage (23). To evaluate 2308Δ*vdtR* trafficking to the ER, the co-localization of *Brucella* with the ER marker calnexin at 24 and 48 h p.i. was investigated (Fig. 1E). Strain 2308Δ*vdtR* successfully co-localized with calnexin at 24 and 48 h p.i. Compared with strains 2308 and 2308Δ*vdtR*-Rev, 2308Δ*vdtR* displayed a slightly lower efficiency for co-localization but with no significant difference. However, 2308Δ*virB123* showed almost no co-localization with calnexin. All these data verified that *vdtR* is necessary for the intracellular trafficking of *Brucella*.

To explore the role of VdtR in *Brucella* virulence, we assessed the virulence of strains 2308 and 2308Δ*vdtR* in a mouse model. As shown in Fig. 1F, the bacterial load in the spleens of 2308Δ*vdtR*-infected mice at 1 week p.i. was 2.91 × 10^6^ CFU/spleen/g with no significant difference compared to that in strain 2308, but at 5 weeks p.i., the bacterial loads in 2308Δ*vdtR*-infected mice were 6.88 × 10^5^ CFU/spleen/g, which were significantly less in comparison with the 2308-infected group with bacterial loads of 4.06 × 10^7^ CFU/spleen/g. Spleen weight was also determined to reflect the degree of splenomegaly. As shown in Fig. 1G, there was no obvious difference in spleen weight of mice infected with 2308 and 2308Δ*vdtR* at 1 week p.i. Spleen weight in the 2308-infected mice, but not 2308Δ*vdtR*, was increased significantly at 5 weeks p.i. with the infection progresses, suggesting the virulence of 2308Δ*vdtR* is significantly reduced. Bacterial loads and spleen weight in the 2308Δ*vdtR*-Rev-infected mice were significantly restored to some extent in comparison with those of strain 2308Δ*vdtR* (Fig. 1F and G). In summary, these results confirmed that VdtR plays an important role in *B. abortus* virulence.

### VdtR negatively regulates gene cluster *BAB_RS27025-RS27055*, a homolog of the aldolase-DUF1537 pathway

**FIG 2:**
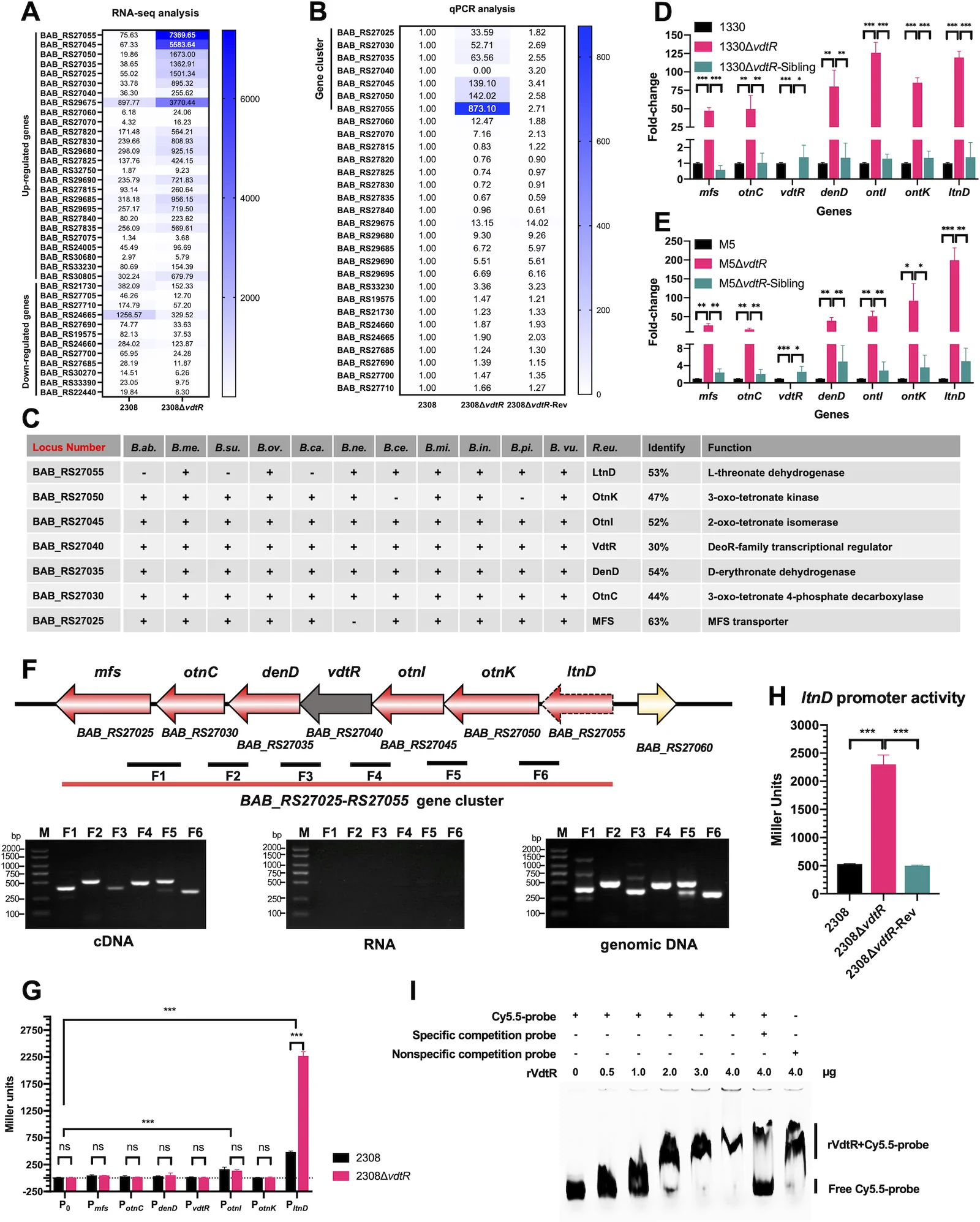
VdtR negatively regulates the *BAB_RS27025-RS27055* gene cluster. (**A**) RNA-seq analysis of 2308Δ*vdtR* strain versus the 2308 strain was performed. The expression levels of 26 up-regulated genes and 12 down-regulated genes were changed more than twofold in the 2308Δ*vdtR* strain. (**B**) qPCR verified the results of RNA-seq, showing that a series of adjacent genes of *BAB_RS27025-RS27055* were significantly up-regulated more than 30-fold in the 2308Δ*vdtR* strain. (**C**) The alignment of amino acid sequences of the *BAB_RS27025-RS27055* genes in *Brucella* spp. was performed with homologous proteins of the aldolase-DUF1537 pathway in the *Ralstonia eutropha* H16 strain. (**D and E**) The transcriptional levels of the genes in the clusters in *B. suis* 1330, 1330Δ*vdtR*, and 1330Δ*vdtR*-sibling strains (**D**) or *B. melitensis* M5, M5Δ*vdtR*, and M5Δ*vdtR*-Sibling strains (**E**) (*N* = 3, mean ± SD, unpaired *t*-test). (**F**) Agarose gel electrophoresis of the PCR fragments F1-F6 from cDNA, RNA (negative control, no reverse transcription), and DNA (positive control) was carried out. (**G and H**) The promoter activity of genes in the cluster was analyzed in strains 2308 and 2308Δ*vdtR* (**G**), and the *ltnD* promoter activity was further compared in strains 2308, 2308Δ*vdtR*, and 2308Δ*vdtR*-Rev (**H**) (*N* = 3, mean ± SD, two-way ANOVA). (**I**) An electrophoretic mobility shift assay was performed to evaluate the binding of VdtR to a Cy5.5-labeled DNA fragment containing the 369-bp promoter region of the *ltnD* gene. *P < 0.05; **P < 0.01; ***P < 0.001. ns, not significant.

To gain insights into VdtR regulation, we probed differentially expressed genes in strain 2308Δ*vdtR* versus strain 2308 using comparative transcriptomics. Based on RNA-seq analysis, a total of 3213 transcripts were detected and compared. According to the statistical analysis, 26 genes were significantly up-regulated and 12 genes were down-regulated with a greater than twofold decrease in expression (Fig. 2A; Dataset S1). The transcriptome data were further verified by quantitative PCR (qPCR). Interestingly, it was found that the expression of gene cluster *BAB_RS27025-RS27055* was significantly up-regulated more than 30-fold, except for the deletion gene *BAB_RS27040*, in strain 2308Δ*vdtR*, and the expression of those genes was successfully restored in strain 2308Δ*vdtR*-Rev (Fig. 2B), suggesting that these adjacent genes may collectively be involved in a biological process.

To further investigate the function of the gene cluster *BAB_RS27025-RS27055*, the aligment of amino acid sequences was performed using the Protein-Protein Basic Local Alignment Search Tool (BLASTP) on the National Center for Biotechnology Information database. As shown in Fig. 2C, this gene cluster shares 30%–63% homology with the aldolase-DUF1537 gene cluster in *Ralstonia eutropha* H16, which is involved in the metabolism of the four-carbon acid sugars L-threonate and D-erythronate (27). To further confirm the occurrence of VdtR-based negative regulation in other *Brucella* spp., we evaluated the gene cluster expression in *B. melitensis* and *B. suis vdtR* mutants, as well as their sibling revertant strains. As shown in Fig. 2D, in *B. suis*, except for the deleted gene *vdtR*, the expression of the gene cluster was significantly increased in the 1330Δ*vdtR* strain versus the 1330 strain, and the 1330Δ*vdtR*-sibling strain restored expression of the gene cluster to the level of the parental strain. In *B. melitensis*, a similar result was observed in the M5Δ*vdtR* strain and the M5Δ*vdtR*-sibling strain (Fig. 2E). These results were consistent with that of the *B. abortus* strain 2308, suggesting that the VdtR has a similar regulatory function in different species of *Brucella*.

Since genes in the cluster were adjacent to each other, co-regulated in 2308Δ*vdtR*, and assumed to co-participate in metabolism of four-carbon acid sugars, we further determined whether they were transcribed under an operon. The DNA sequence of the gene cluster region was analyzed. Reverse transcription PCR (RT-PCR) was performed on total RNA with primer pairs that spanned the adjacent genes, resulting in overlapping amplification products (Fig. 2F). The primer pairs covering two adjacent genes in the gene cluster produced RT-PCR products of the same size, with PCR amplification from DNA template as the positive control. No PCR amplification products were obtained from the negative-control RNA template (Fig. 2F), indicating that these genes were co-transcribed into a polycistronic mRNA and controlled by an operon.

To investigate the sites potentially regulated by VdtR in the operon, the predicted promoter fragments of *ltnD*, *otnK*, *otnI*, *vdtR*, *denD*, *otnC*, and *mfs* (corresponding to genes of *BAB_RS27055*, *BAB_RS27050*, *BAB_RS27045*, *BAB_RS27040*, *BAB_RS27035*, *BAB_RS27030*, and *BAB_RS27025*, respectively, according to the homolog genes in the aldolase-DUF1537; Fig. 2C) were inserted into the promoterless plasmid pMCR-LacZ to evaluate the promoter activity. Miller units were determined by hydrolysis of o-nitrophenyl-β-d-galactoside via β-galactosidase in strains 2308 and 2308Δ*vdtR* harboring the recombinant plasmids with different promoters. As shown in Fig. 2G, the promoters of *ltnD* and *otnI* had obvious β-galactosidase activity, but those of *otnK*, *vdtR*, *denD*, *otnC*, and *mfs* did not have this activity. Only the *ltnD* promoter showed significantly increased activity in the 2308Δ*vdtR* strain compared with strain 2308. To confirm whether the *ltnD* promoter activity is dependent on VdtR regulation, the recombinant plasmid pMCR-P*_ltnD_*-LacZ was transformed into strain 2308Δ*vdtR*-Rev. The β-galactosidase activity showed that the activity of the *ltnD* promoter in the 2308Δ*vdtR*-Rev strain was restored to that in strain 2308 (Fig. 2H). These data indicated that the gene cluster was co-transcribed under the control of the *ltnD* promoter, whereby the *ltnD* promoter was negatively regulated by the VdtR protein.

Although *ltnD* promoter activity depends on VdtR, whether VdtR directly binds the *ltnD* promoter region to negatively regulate the expression of the gene cluster remains unknown. To investigate the function, an electrophoretic mobility shift assay (EMSA) was performed to evaluate the binding between VdtR and the *ltnD* promoter. As shown in Fig. 2I, the recombinant VdtR protein (rVdtR) clearly bound the Cy5.5-labeled *ltnD* promoter probe, the binding activity of rVdtR and the Cy5.5-labeled probe was significantly increased with the increase in protein concentration (Fig. 2I). When the non-labeled specific competition probe was added to the system, the binding of the rVdtR protein to the Cy5.5-labeled probe was significantly inhibited. Yet, when the non-labeled, non-specific competition probe was added, the binding of the rVdtR protein to the Cy5.5-labeled probe was not effectively disturbed (Fig. 2I). In addition, we truncated the *ltnD* promoter to the different lengths of Cy5.5-labeled probes (Fig. S3A); the binding of rVdtR to the shortened probes showed that rVdtR could bind to multiple truncated fragments of the *ltnD* promoter region (Fig. S3B), suggesting that there are multiple VdtR binding sites in the *ltnD* promoter region. The results confirmed that rVdtR binds directly to the promoter region of the *ltnD* gene to negatively regulate the expression of the *BAB_RS27025-RS27055* gene cluster.

### VdtR regulation of the erythronate metabolic pathway is activated by L-threonate or D-erythronate

**FIG 3:**
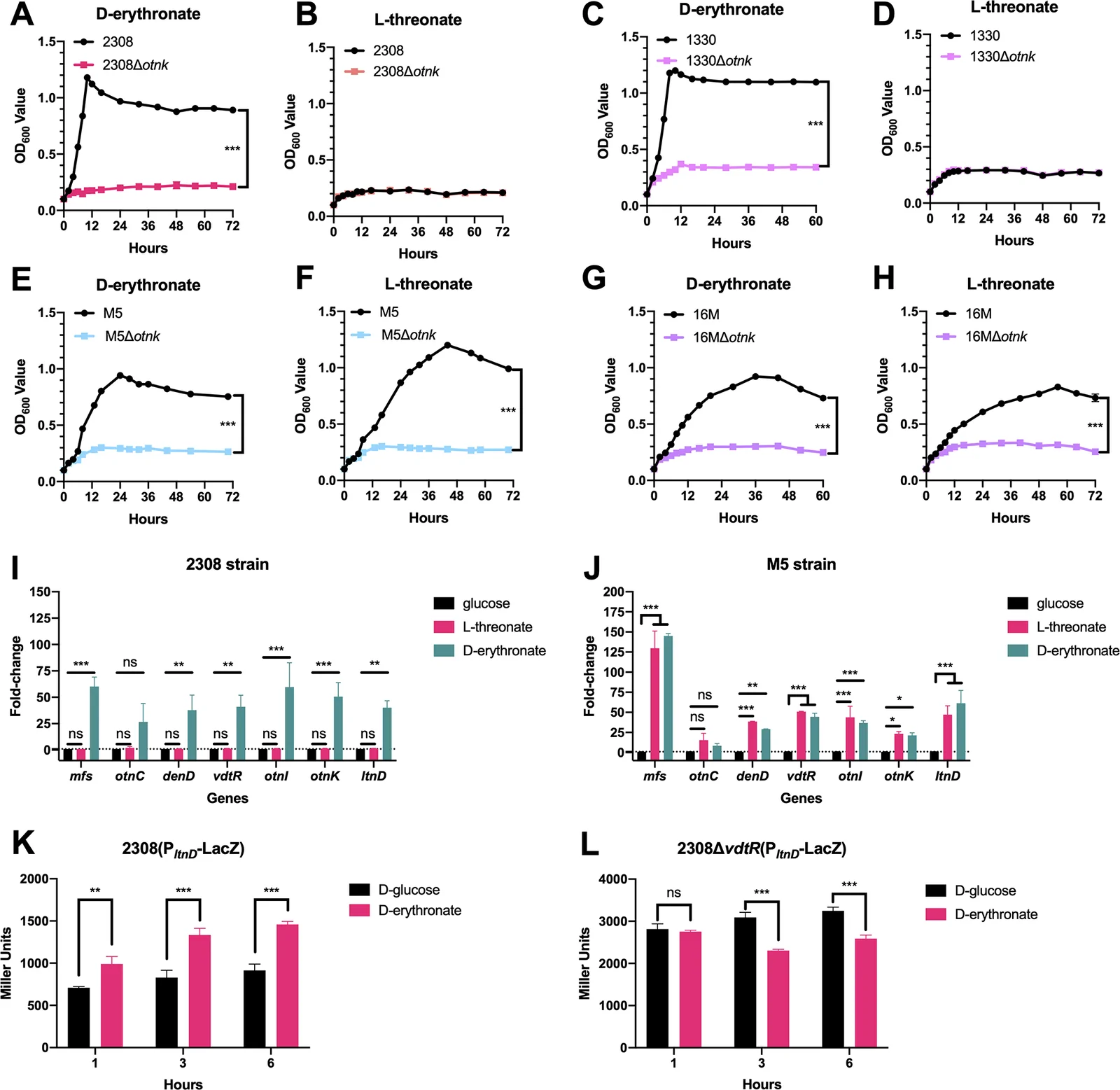
D-erythronate activated expression of the erythronate metabolic pathway to facilitate *Brucella* growth, which is VdtR-dependent. (**A-H**) Growth curves of *B. abortus* 2308 and 2308Δ*otnK* were determined in minimal medium (MM) supplemented with 10-mM D-erythronate (**A**) or 10-mM L-threonate (**B**) (*N* = 3, mean ± SD, multiple unpaired *t*-test). Growth of *B. suis* 1330 and 1330Δ*otnK* was analyzed in MM with 10-mM D-erythronate (**C**) or 10-mM L-threonate (**D**) (*N* = 3, mean ± SD, multiple unpaired *t*-test). Growth of *B. melitensis* M5 and M5Δ*otnK* was analyzed in MM with 10-mM D-erythronate (**E**) or 10-mM L-threonate (**F**) (*N* = 3, mean ± SD, multiple unpaired *t*-test). Growth of *B. melitensis* 16M and 16MΔ*otnK* was determined in MM with 10-mM D-erythronate (**G**) or 10-mM L-threonate (**H**) (*N* = 3, mean ± SD, multiple unpaired *t*-test). (**I and J**) The transcription level of the erythronate metabolic gene cluster was analyzed by qPCR in strains 2308 (**I**) and M5 (**J**) induced in MM with 10-mM D-glucose, D-erythronate, or 10-mM L-threonate for 6 h (*N* = 3, mean ± SD, two-way ANOVA). (**K and L**). The *ltnD* promoter activity in strain 2308 (**K**) or 2308Δ*vdtR* (**L**) was compared after incubation with D-glucose and D-erythronate for 1, 3, and 6 hr(*N* = 3, mean ± SD, two-way ANOVA). *P < 0.05; **P < 0.01; ***P < 0.001. ns, not significant.

A BLAST analysis showed that *Brucella BAB_RS27025-RS27055* is a homolog of aldolase-DUF1537 gene cluster, which is involved in the metabolism of L-threonate and D-erythronate. To confirm this, the growth of *Brucella* was evaluated *in vitro* in minimal medium (MM) supplemented with 10-mM L-threonate or D-erythronate as the additional carbon source. In addition, OtnK in *R. eutropha* H16 is proved essential for L-threonate and D-erythronate catabolism (27); hence, to verify the gene cluster functions in the metabolism of the four-carbon acid sugars, the *Brucella* deletion mutants lacking the key kinase OtnK were constructed to evaluate their growth in MM with L-threonate or D-erythronate. The results showed that *B. abortus* 2308 and *B. suis* 1330 can proliferate with D-erythronate, but not L-threonate, as the additional carbon source (Fig. 3A through D), whereas the *B. melitensis* 16M and M5 strains can grow well in MM supplemented with either additional L-threonate or D-erythronate (Fig. 3E through H), consistent with our expected result, because of the LtnD mutation in *B. abortus* and *B. suis* (Fig. 2C). Subsequently, we found that all *otnK* mutants derived from *B. abortus*, *B. melitensis*, and *B. suis* cannot proliferate in MM with either additional L-threonate or D-erythronate carbon source (Fig. 3A through H), confirming that *Brucella BAB_RS27025-RS27055* is involved in the metabolism of L-threonate or D-erythronate, thus designated as the *Brucella* erythronate metabolic pathway.

As mentioned above, VdtR belongs to the DeoR family of transcription regulators, which have an helix-turn-helix (HTH) motif for DNA-binding at the N-terminal and a sugar-binding domain at the C-terminal. The DNA-binding domain is engaged in the binding of VdtR to DNA, and the sugar-binding domain is involved in the interaction between VdtR and substrates. In previous literature, the sugar-binding domain of DeoR has been reported to be a catalytically inactive version of the ISOCOT fold, which, however, maintains substrate-binding ability (28). The C-terminal of DeoR senses diverse sugar derivatives, such as deoxyribose nucleoside, galactosamine, tagatose phosphate, and L-ascorbate (28, 29). Based on this evidence, we proposed the hypothesis that VdtR binds and interacts with L-threonate, D-erythronate, or their derivatives, resulting in dissociation of VdtR from DNA, allowing transcription of the *BAB_RS27025-RS27055* gene cluster. To verify this, we first evaluated the transcription levels of the gene cluster in *B. abortus* 2308 and *B. melitensis* M5 stimulated for 6 h with MM containing 10-mM D-glucose, L-threonate, or D-erythronate (Fig. 3I and J). Interestingly, compared to D-glucose, D-erythronate, but not L-threonate, significantly induced up-regulation of the gene cluster in *B. abortus* 2308 (Fig. 3I). However, in *B. melitensis* M5, both L-threonate and D-erythronate induced the expression of the gene cluster (Fig. 3J). As mentioned above, *B. abortus* 2308 had a mutation on the *ltnD* gene of the cluster, which might be the reason for its utilization of D-erythronate, but not L-threonate, as a carbon source. These findings suggested that the metabolism of L-threonate or D-erythronate is a key to inducing the expression of the gene cluster in *Brucella*.

Since the erythronate metabolic pathway is negatively regulated by VdtR, to verify whether L-threonate- or D-erythronate-induced up-regulation of the gene cluster depends on VdtR, *ltnD* promoter activity in strains 2308 and 2308Δ*vdtR* was evaluated by induction with D-glucose or D-erythronate. After being induced for 1, 3, and 6 h, promoter activity in strain 2308(P*_ltnD_*-lacZ) was greatly increased under D-erythronate induction compared with that following D-glucose induction at all indicated times (Fig. 3K). However, promoter activity in strain 2308Δ*vdtR*(P*_ltnD_*-lacZ) remained unchanged following a 1-h incubation and considerably decreased following 3 and 6 h of D-glucose incubation in comparison with that seen under D-erythronate induction (Fig. 3L). These data suggested that the erythronate metabolic pathway induced by D-erythronate is VdtR dependent.

To confirm whether VdtR interacts directly with D-erythronate to affect its binding to DNA, we evaluated the electrophoretic mobility of the rVdtR protein with or without L-threonate/D-erythronate under non-reducing SDS-PAGE gel conditions. The results showed that the rVdtR protein exhibited a slight or no shift in electrophoretic mobility following the addition of L-threonate or D-erythronate to the electrophoretic system (Fig. S4A). Subsequently, EMSA was performed to assess the effect of L-threonate or D-erythronate on rVdtR binding to the *ltnD* promoter probe, and the results indicated that neither had an effect (Fig. S4B). These data suggested that VdtR regulation of the erythronate metabolic pathway is activated by L-threonate or D-erythronate, but neither of the acid sugars has a direct effect on VdtR binding to the *ltnD* promoter.

### VdtR-mediated negative regulation of the erythronate metabolic pathway is necessary for *Brucella* intracellular survival and pathogenicity in mice

**FIG 4:**
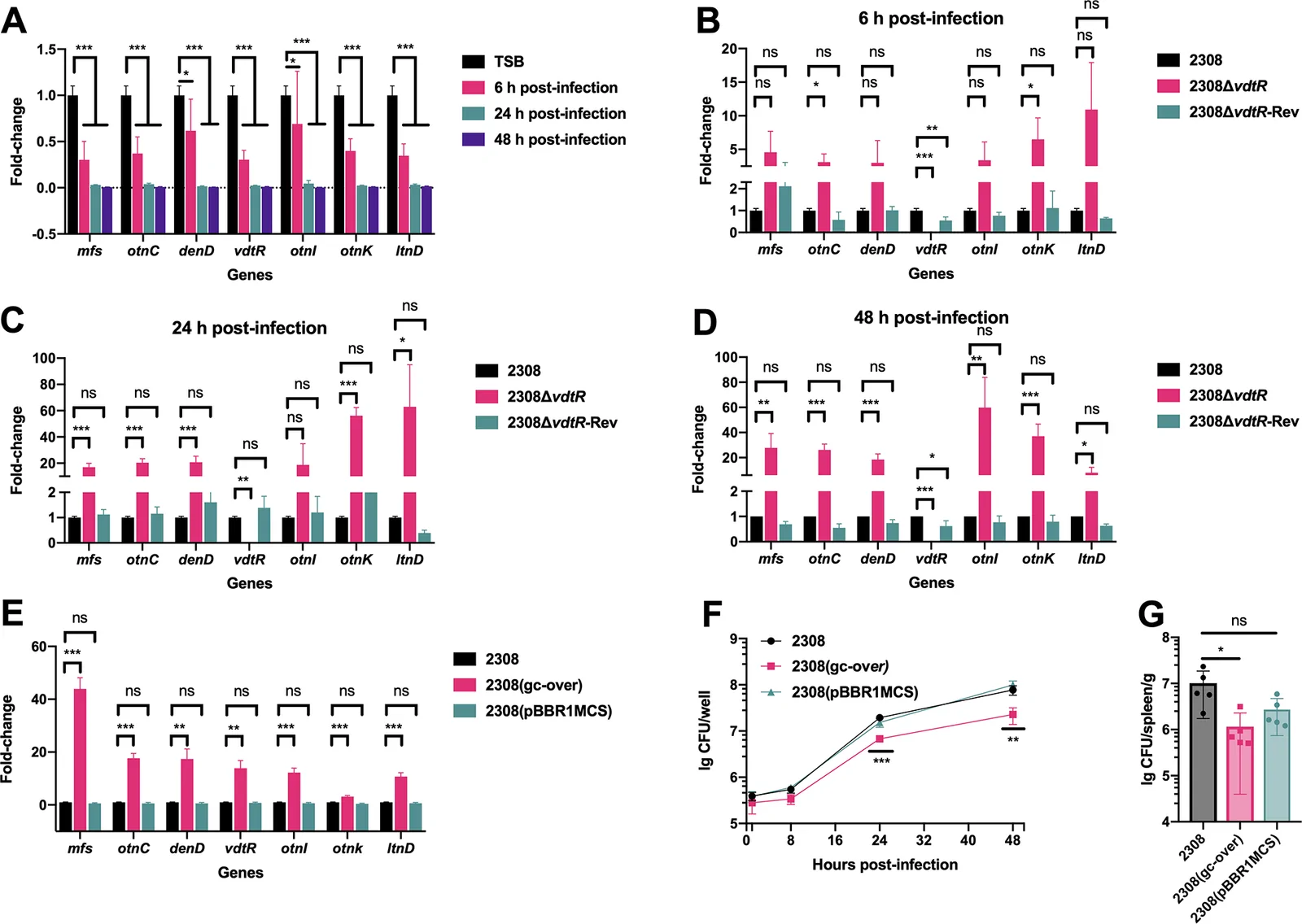
The VdtR-mediated negative regulation of the erythronate metabolic pathway is necessary for *Brucella* intracellular replication and pathogenicity in mice. (**A**) Transcription levels of the genes in the erythronate metabolic pathway in intracellular *Brucella* were assessed by qPCR at 6, 24, and 48 h p.i. (*N* = 3, mean ± SD, two-way ANOVA). (**B-D**) Transcription levels of the genes in the erythronate metabolic pathway in intracellular 2308, 2308Δ*vdtR*, and 2308Δ*vdtR*-Rev at 6 h (**B**), 24 h (**C**), and 48 h (**D**) p.i. were assessed by qPCR (*N* = 3, mean ± SD, one-way ANOVA). (**E**) Over-expression of the genes in the erythronate metabolic pathway was verified in strain 2308(gc-over) by qPCR (*N* = 3, mean ± SD, multiple unpaired *t*-test). (**F**) Intracellular survival of the 2308, 2308(gc-over), and 2308 (pBBR1MCS) strains was assessed in RAW264.7 macrophages (*N* = 3, mean ± SD, multiple unpaired *t*-test). (**G**) Comparative analysis of bacterial loads in spleen infected with *B. abortus* 2308, 2308(gc-over), and 2308(pBBR1MCS) at 4 weeks p.i. (*N* = 5, mean ± SD, one-way ANOVA). *P < 0.05; **P < 0.01; ***P < 0.001. ns, not significant.

Considering the negative regulation of the *BAB_RS27025-RS27055* gene cluster by VdtR, we hypothesized that the up-regulation of the gene cluster is detrimental to *Brucella* virulence. To investigate this hypothesis, we first evaluated the expression of the gene cluster in intracellular *Brucella* at 6, 24, and 48 h p.i. The qPCR result showed that seven genes in the erythronate metabolic pathway were significantly down-regulated at all indicated times compared to *Brucella* cultured in TSB, especially during the replication stages at 24 and 48 h p.i. (Fig. 4A), suggesting that the expression of the gene cluster was repressed during *Brucella* intracellular replication. To further confirm that the suppressed expression was VdtR dependent, we determined the expression of the gene cluster intracellularly for strains 2308, 2308Δ*vdtR*, and 2308Δ*vdtR*-Rev at 6, 24, and 48 h p.i. At 6 h p.i., expression of *otnC* and *otnK* was up-regulated in the 2308Δ*vdtR* strain (Fig. 4B). At 24 and 48 h p.i., expression of the gene cluster except for deleted *vdtR* was significantly up-regulated in the 2308Δ*vdtR* strain, compared to the 2308 strain. The revertant strain 2308Δ*vdtR*-Rev, however, showed restored cluster gene expression to the level of the parental strain 2308 at these time points (Fig. 4B through D). These results confirmed that the expression of the *BAB_RS27025-RS27055* gene cluster depends on VdtR regulation in intracellular *Brucella*.

Since VdtR negatively regulates expression of the gene cluster and the 2308Δ*vdtR* strain is defective in intracellular survival, we further investigated whether the up-regulation of the *BAB_RS27025-RS27055* gene cluster contributes to the attenuated virulence of the 2308Δ*vdtR* strain. To this end, we constructed a gene cluster over-expression strain 2308(gc-over), and qPCR verified that all seven cluster genes were over-expressed in the 2308(gc-over) strain (Fig. 4E). Subsequently, we performed a macrophage infection experiment to assess the intracellular survival of the 2308, 2308(gc-over), and 2308(pBBR1MCS) strains (empty plasmid control strain). As shown in Fig. 4F, 2308(gc-over) presented obviously reduced intracellular survival in comparison with the 2308 and 2308(pBBR1MCS) strains at 24 and 48 h p.i.; this result was consistent with the intracellular survival of the 2308Δ*vdtR* strain. In a further study, mice were infected to evaluate the virulence of these *Brucella* strains at 4 weeks p.i. Strain 2308(gc-over) showed a significantly decreased number of CFUs/spleen/g, compared to that of the 2308 strain. The 2308 (pBBR1MCS) recovered partial bacterial CFU levels of no significant difference from those of the 2308 strain (Fig. 4G). These data indicated that over-expression of the gene cluster is detrimental to the intracellular survival and virulence of *B. abortus*.

### Erythronate is a preferred carbon source for *Brucella* as erythritol but adopts a different carbon metabolic pathway

**FIG 5:**
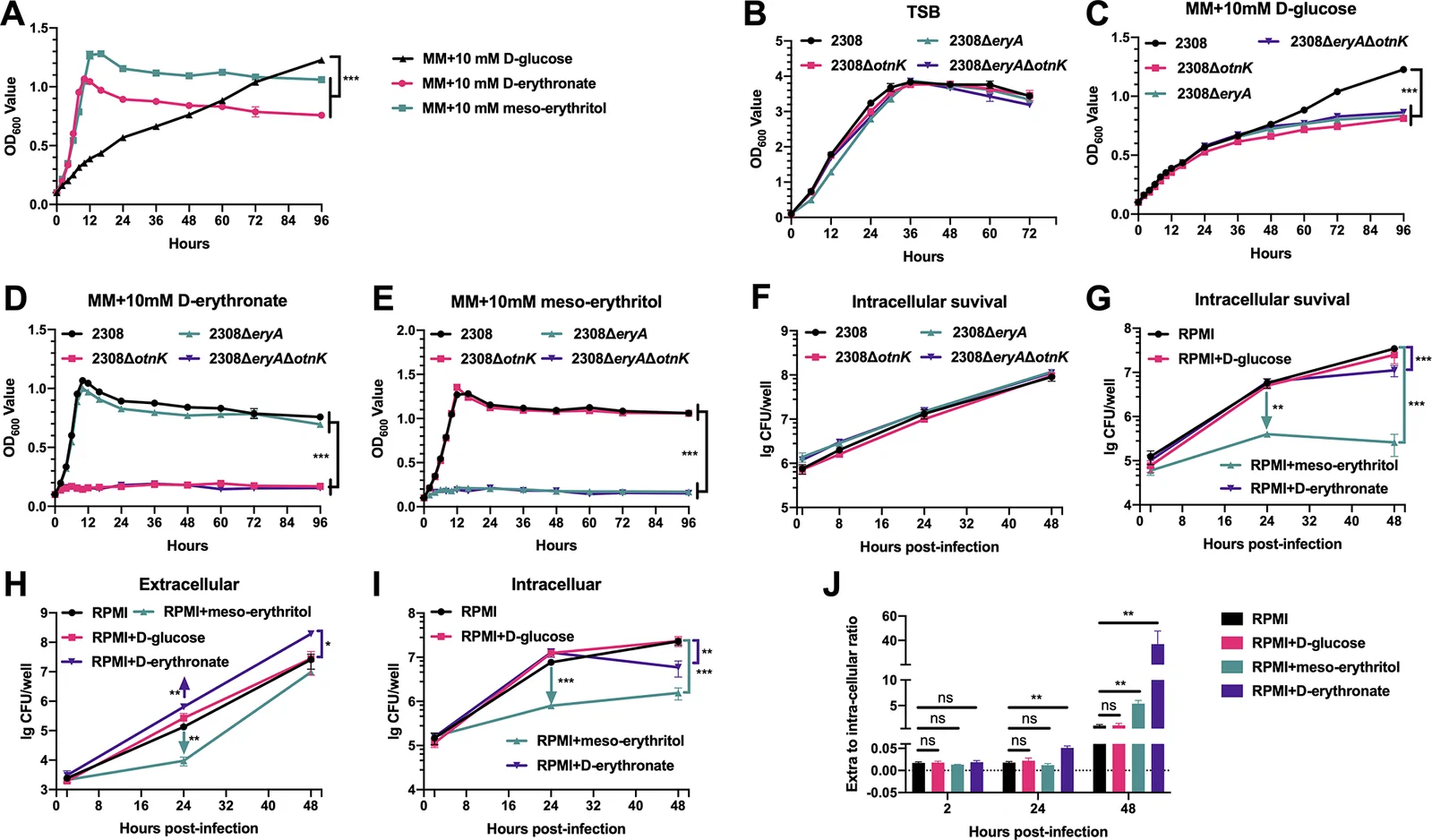
Erythritol and D-erythronate are preferentially utilized for *Brucella* replication through respective metabolic pathways *in vitro*. (**A**) Growth curves of *B. abortus* strain 2308 in MM with 10-mM D-glucose, 10-mM D-erythronate, or 10-mM meso-erythritol were determined (*N* = 3, mean ± SD, multiple unpaired *t*-test). (**B**) Growth of *B. abortus* strains 2308, 2308Δ*otnK*, 2308Δ*eryA*, and 2308Δ*eryA*Δ*otnK* was analyzed in TSB (*N* = 3, mean ± SD, multiple unpaired *t*-test). (**C-E**) Growth of *B. abortus* strains 2308, 2308Δ*otnK*, 2308Δ*eryA*, and 2308Δ*eryA*Δ*otnK* in MM with 10-mM D-glucose (**C**), 10-mM D-erythronate (**D**), and 10-mM meso-erythritol (**E**) was compared (*N* = 3, mean ± SD, multiple unpaired *t*-test). (**F**) Intracellular survival of *B. abortus* strains 2308, 2308Δ*otnK*, 2308Δ*eryA*, and 2308Δ*eryA*Δ*otnK* was evaluated in RAW264.7 macrophages (*N* = 3, mean ± SD, two-way ANOVA). (**G**) RAW 264.7 macrophages were infected by *B. abortus* 2308 at a multiplicity of infection of 100:1 for 1 h; extracellular *Brucella* was killed by Roswell Park Memorial Institute (RPMI) with 100-µg/mL gentamicin. The infected cells were maintained in RPMI with 20-µg/mL gentamicin and 1% glucose or 1% meso-erythritol or 1% D-erythronate (*N* = 3, mean ± SD, two-way ANOVA). (**H-J**) RAW264.7 macrophages were infected as described above. After gentamycin killing, the infected cells were maintained in RPMI without gentamicin to allow extracellular *Brucella* growth in RPMI with 1% glucose or 1% meso-erythritol or 1% D-erythronate (**H**). Meanwhile, intracellular *Brucella* (**I**) was also detected. The ratio of extracellular-to-intracellular bacteria in the presence of RPMI or RPMI plus 1% D-glucose or 1% meso-erythritol or 1% D-erythronate was determined (**J**) (*N* = 3, mean ± SD, multiple unpaired *t*-test). *P < 0.05; **P < 0.01; ***P < 0.001. ns, not significant.

Erythritol, as a four-carbon polyol, is a preferred carbon source for *Brucella* spp. Erythronate, as a four-carbon acid sugar, is a chemical derivative formed from erythritol by oxidation (30). Therefore, we queried whether erythronate is also a preferred carbon source utilized by *Brucella*. To confirm this prediction, we first evaluated the preference of the 2308 strain for utilization of glucose, erythronate, and erythritol (Fig. 5A). The growth curves of strain 2308 cultured in MM supplied with additional 10 mM D-glucose, D-erythronate, or meso-erythritol as a carbon source were plotted, and the results demonstrated that *Brucella* used D-erythronate and meso-erythritol more efficiently than D-glucose, reaching the stationary phase in both sugars at 8- to 12-h incubation. When grown in glucose, *Brucella* took a long time to reach similar levels of growth (Fig. 5A), suggesting that *Brucella* prefers to utilize D-erythronate, which is similar to erythritol.

Next, we investigated whether *Brucella* could convert erythritol into erythronate and then exploit the erythronate metabolic pathway. To this end, we constructed a 2308Δ*otnK* strain with a disrupted erythronate metabolic pathway, a 2308Δ*eryA* strain in which erythritol kinase EryA was deleted to disturb the erythritol metabolic pathway, and a double gene deletion strain, 2308Δ*eryA*Δ*otnK*, in which both metabolic pathways were disrupted. First, the growth curves of 2308, 2308Δ*otnK*, 2308Δ*eryA*, and 2308Δ*eryA*Δ*otnK* strains were determined in TSB, indicating that the *eryA* and *otnK* gene deletion had no effect on *Brucella* growth in rich medium (Fig. 5B). Then, the growth curves were assessed in MM supplied with 10-mM D-glucose, D-erythronate, or erythritol as an additional carbon source. In MM containing glucose, the growth of strain 2308 was faster than that of 2308Δ*otnK*, 2308Δ*eryA*, and 2308Δ*eryA*Δ*otnK* after 48 h of incubation, but no growth difference among the 2308Δ*otnK*, 2308Δ*eryA*, and 2308Δ*eryA*Δ*otnK* strains was observed, suggesting that *otnK* or *eryA* deletion affects *Brucella*’s utilization of glucose, especially in the later stage of growth (Fig. 5C). When D-erythronate was added into the MM as an additional carbon source, the growth of the 2308Δ*eryA* strain was similar to that of parental strain 2308, but the 2308Δ*otnK* and 2308Δ*eryA*Δ*otnK* strains did not grow in MM containing D-erythronate (Fig. 5D), suggesting that erythronate was metabolized by the erythronate metabolic pathway but not by the erythritol pathway in *Brucella*. When cultured in MM with meso-erythritol, the growth of the 2308Δ*otnK* and 2308 strains was similar, but the 2308Δ*eryA* and 2308Δ*eryA*Δ*otnK* strains did not grow (Fig. 5E), indicating that *Brucella* metabolized erythritol through the erythritol metabolic pathway but not the erythronate metabolic pathway. These results suggested that erythronate and erythritol pathways are independent.

### *Brucella* prefers erythronate and erythritol to promote the proliferation of extracellular bacteria

Since erythronate and erythritol are preferred sugars for *Brucella*, whether the presence of the erythronate and erythritol can enhance the ability of *Brucella* intracellular survival remains unknown. In this study, we first evaluated the intracellular survival of the 2308, 2308Δ*otnK*, 2308Δ*eryA*, and 2308Δ*eryA*Δ*otnK* strains within RAW264.7 macrophages, indicating that deletion of *otnK*, *eryA*, or both genes did not affect the intracellular survival of *Brucella* (Fig. 5F). Then, cell infection was performed to evaluate *Brucella* intracellular survival after D-glucose, D-erythronate, or meso-erythritol (1% wt/vol) was added to the cell culture medium Roswell Park Memorial Institute (RPMI) 1640. The results showed that glucose addition did not affect *Brucella* intracellular survival (Fig. 5G). However, intracellular survival was significantly decreased in RPMI + erythritol at 24 and 48 h p.i. compared to RPMI and RPMI + glucose (Fig. 5G), which was consistent with a previous study (31). The intracellular survival was also decreased in RPMI + erythronate at 48 h p.i. in comparison with RPMI or RPMI + glucose, while survival was not changed in RPMI, RPMI + D-erythronate, or glucose at 24 h p.i. (Fig. 5G), suggesting that the inhibitory activity of D-erythronate to intracellular survival was less than that of erythritol.

It was found that erythritol can inhibit *Brucella* intracellular survival while promoting extracellular replication (31); hence, extracellular replication was further assessed in 2308-infected macrophages when RPMI was supplied with additional D-glucose, D-erythronate, or meso-erythritol. At 2, 24, and 48 h p.i., the number of CFUs recovered from the cellular supernatant was determined. The results showed that the number of CFUs increased at 24 and 48 h p.i. in the presence of D-erythronate compared with that found in the presence of RPMI or RPMI + glucose (Fig. 5H). While the number of CFUs observed in the presence of RPMI + erythritol was less than that found in RPMI, RPMI + glucose, and RPMI + D-erythronate at 24 h p.i., the extracellular *Brucella* proliferated rapidly from 24 to 48 h p.i. Moreover, to enumerate the intracellular bacteria simultaneously, the cells were treated with gentamycin to kill extracellular bacteria at 2, 24, and 48 h p.i. The result was consistent with that of intracellular survival seen in the gentamycin protection assay as described above. In RPMI + erythritol or D-erythronate, intracellular survival was significantly inhibited (Fig. 5I). To compare the proliferation efficiency of extracellular and intracellular bacteria, the extracellular-to-intracellular ratio was calculated as CFUs of extracellular bacteria/CFUs of intracellular bacteria. As shown in Fig. 5J, the proliferation efficiency of extracellular *Brucella* was higher than that of intracellular *Brucella* in RPMI + erythritol at 48 h p.i. (Fig. 5J), with the results being consistent with those reported previously (31). When erythronate was added to the RPMI medium, the ratio of extracellular-to-intracellular bacteria at 24 and 48 h p.i. was notably increased by about 2.8- and 30-fold, respectively, compared with that seen in RPMI only (Fig. 5J), indicating that D-erythronate promotes extracellular *Brucella* proliferation but not intracellular survival. These data suggested that erythronate, as well as erythritol, is preferentially utilized by extracellular *Brucella* but is not favorable to intracellular *Brucella* survival.

### Preferential utilization of erythronate and erythritol by *Brucella* in pregnant mice leads to a placental inflammatory response and abortion

**Fig 6:**
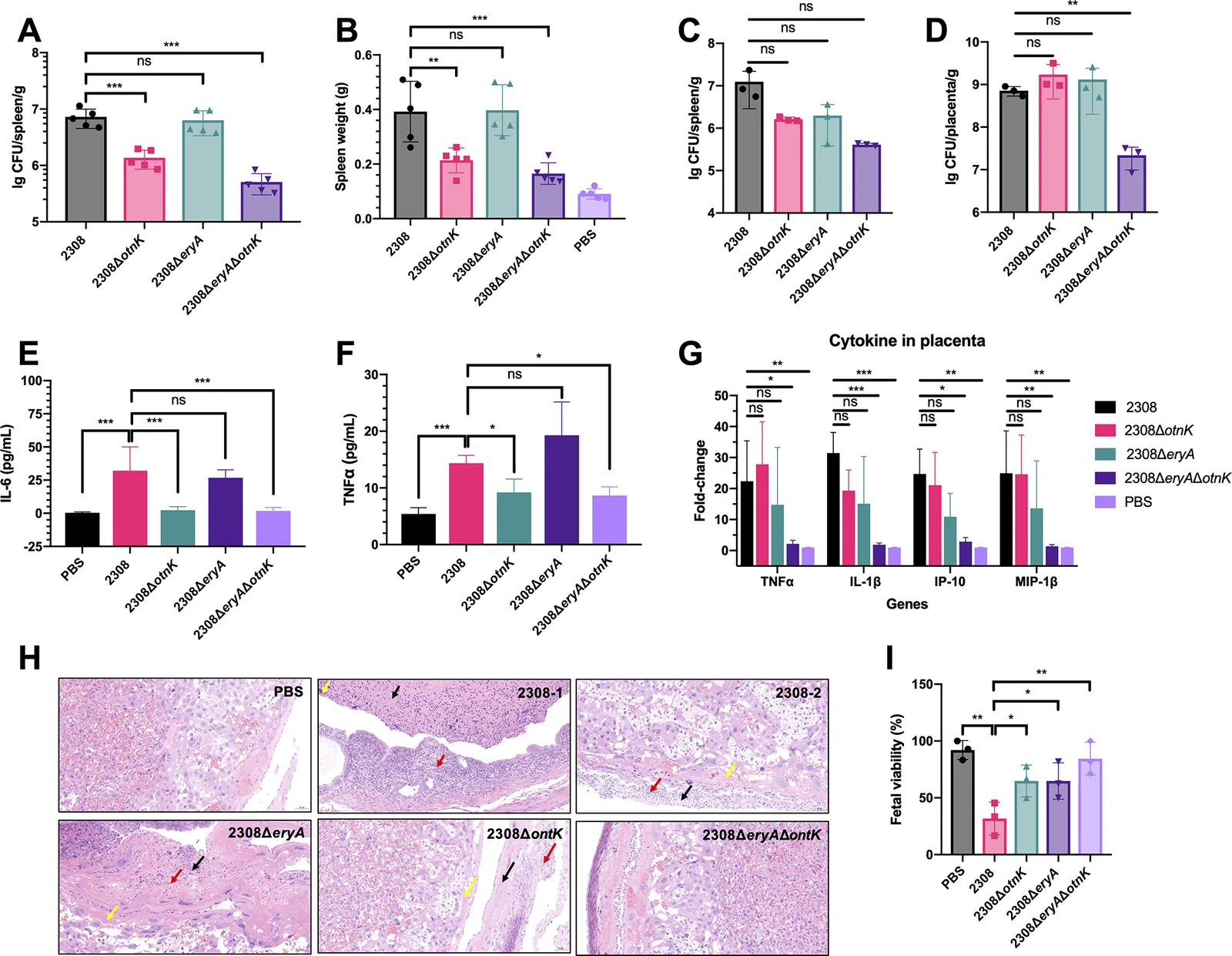
The preferential utilization of erythronate and erythritol by *Brucella* in pregnant mice leads to a placental inflammatory response and abortion. (**A and B**) Comparative analysis of bacterial loads in spleen (**A**) and splenomegaly (**B**) of mice infected by *B. abortus* strains 2308, 2308Δ*otnK*, 2308Δ*eryA*, and 2308Δ*eryA*Δ*otnK* at 13 days p.i. (*N* = 5, mean ± SD, one-way ANOVA). (**C and D**) Comparative analysis of bacterial loads in spleen (**C**) and placenta (**D**) of pregnant mice infected by *B. abortus* strains 2308, 2308Δ*otnK*, 2308Δ*eryA*, and 2308Δ*eryA*Δ*otnK* at 13 days p.i. (*N* = 3, mean ± SD, unpaired *t*-test). (**E-G**) Release of IL-6 (**E**) and TNF-α (**F**) in the serum of non-pregnant mice (*N* = 3, mean ± SD, unpaired *t*-test), and the transcriptional levels of TNF-α, IL-1β, IP-10, and MIP-1β in the placenta of pregnant mice (**G**) infected with *B. abortus* strains 2308, 2308Δ*otnK*, 2308Δ*eryA*, and 2308Δ*eryA*Δ*otnK* at 13 days p.i. (*N* = 3, mean ± SD, two-way ANOVA). (**H**) Representative images of the histopathology observed in the placenta of *B. abortus-*infected pregnant mice at 13 days p.i. Yellow arrows denote calcification. Black arrows denote necrosis. Red arrows denote neutrophil infiltration. (**I**) Viability of pups was evaluated based on fetal size and skin color, and the percentage of fetal viability was calculated (*N* = 3, mean ± SD, unpaired *t*-test). *P < 0.05; **P < 0.01; ***P < 0.001. ns, not significant.

*B. abortus* induces moderate chronic inflammation during its persistence in experimentally infected mice and the natural bovine reservoir (8, 32, 33). The pathogen, on the other hand, produces significant acute inflammation in the placenta of pregnant cows, leading to abortion. It is presumed that abortion is caused by the accumulation of pathogens due to the presence of large amounts of erythritol in the placenta (31). In this study, we found that erythronate is a novel preferred sugar for *Brucella* extracellular proliferation. Therefore, we assumed that erythronate utilization may also contribute to the *Brucella-*associated induction of the inflammatory response and placentitis in pregnant animals. First, we assessed the virulence of the 2308, 2308Δ*otnK*, 2308Δ*eryA*, and 2308Δ*eryA*Δ*otnK* strains in non-pregnant mice at 13 days p.i. As shown in Fig. 6A, compared to the 2308 strain, bacterial loads in spleen infected with the 2308Δ*otnK* and 2308Δ*eryA*Δ*otnK* strains were greatly decreased, but bacterial loads in the 2308Δ*eryA*-infected group were not obviously changed. The results of spleen weight showed that splenomegaly was less in mice infected by the 2308Δ*otnK* and 2308Δ*eryA*Δ*otnK* strains than that seen following infection with strain 2308, while there was no significant change in spleen weight between the 2308- and 2308Δ*eryA*-infected groups (Fig. 6B). In addition, the inflammatory response was evaluated by determining the levels of the pro-inflammatory cytokines interleukin (IL)-6 and tumor necrosis factor alpha (TNF-α) in sera of infected mice. The results revealed that the release of IL-6 and TNF-α in sera following 2308Δ*otnK* and 2308Δ*eryA*Δ*otnK* infection was significantly reduced relative to that found following infection with strain 2308. The 2308Δ*eryA* infection induced similar levels of IL-6 and TNF-α to 2308 infection (Fig. 6E and F). These data suggested that the OtnK-mediated erythronate metabolic pathway plays an important role in *B. abortus* virulence, inducing splenomegaly and inflammatory response. However, the EryA-mediated erythritol metabolic pathway is not essential for *B. abortus* virulence in the mouse model, which is consistent with previously reported results (12).

In a further study, we investigated the roles of the erythronate and erythritol metabolic pathways in pregnant mice. Bacterial loads and the inflammatory response were evaluated in pregnant mice at 13 days p.i. (corresponding to the 18th day of gestation). The CFUs recovered from the spleen infected with 2308Δ*otnK*, 2308Δ*eryA*, and 2308Δ*eryA*Δ*otnK* were reduced in number compared to those seen following 2308 infection, but there was no remarkable difference (*P* > 0.0500) (Fig. 6C). Meanwhile, the CFUs recovered from placenta infected with 2308Δ*otnK* and 2308Δ*eryA* showed no change in comparison with the 2308 infection. However, strain 2308Δ*eryA*Δ*otnK* showed a significant decrease in placental colonization compared to that of strain 2308 (Fig. 6D). As described above, erythronate is a preferred carbon source utilized by *Brucella* and can promote the proliferation of extracellular *Brucella*, for which the metabolic pathway is activated by erythronate or threonate. To investigate the utilization of erythronate in placenta by *Brucella*, qPCR was performed to evaluate the expression of the gene cluster in the erythronate metabolic pathway. The results showed that the expression of six genes (the *ltnD* gene was not included due to its mutation in *B. abortus*) was significantly increased in placenta infected with *Brucella* (Fig. S5), suggesting that the erythronate metabolic pathway was significantly activated in *Brucella* in the placenta of pregnant mice.

To assess the inflammatory response of *Brucella*-infected placenta, the transcriptional levels of TNF-α, IL-1β, IP-10, and MIP-1β were analyzed by qPCR in the placenta, indicating that the transcription of cytokines was obviously increased following infection with strain 2308 compared to that found in the phosphate-buffered saline (PBS) group. Following 2308Δ*otnK* and 2308Δ*eryA* infection, the cytokine levels showed a slight decrease relative to those found following infection with strain 2308, with no significant difference. Interestingly, the transcription of cytokines in the 2308Δ*eryA*Δ*otnK*-infected placenta was significantly reduced in comparison with the 2308-infected placenta, and the levels of cytokines were similar to those found in the PBS group (Fig. 6G). Furthermore, the levels of IL-6 and TNF-α released into sera of the pregnant mice were determined. The results showed that the production of IL-6 and TNF-α in sera was obviously deceased following 2308Δ*eryA*Δ*otnK* infection compared to 2308 infection, and 2308Δ*otnK* and 2308Δ*eryA* infections also resulted in a decrease in IL-6 and TNF-α release relative to 2308 infection, but there was no significant difference (Fig. S6), which was consistent with the results found in infected placenta.

Moreover, we evaluated the histopathological changes induced in the placenta by *Brucella* infection. As shown in Fig. 6H, 2308 infection caused significant pathological changes such as neutrophil infiltration, necrosis, and calcification. Infection of placenta by 2308Δ*otnK* and 2308Δ*eryA* caused slightly less severe, but similar, pathological changes to those induced by strain 2308, while 2308Δ*eryA*Δ*otnK* infection hardly resulted in histopathological changes in the placenta (Fig. 6H).

In addition, the fetal survival ratio was statistically analyzed in 2308-, 2308Δ*otnK*-, 2308Δ*eryA*-, and 2308Δ*eryA*Δ*otnK*-infected pregnant mice, with the results indicating that 31.8% of the pups were viable in pregnant mice infected by strain 2308. Individual fetuses displayed visible calcification and were re-absorbed by the uterus (Fig. 6H; Fig.S7). Following 2308Δ*otnK* and 2308Δ*eryA* infection, fetal viability was significantly increased relative to that following infection by strain 2308, with 64.8% and 64.7% survival, respectively. The 2308Δ*eryA*Δ*otnK*-infected pregnant mice showed 84.4% fetal viability, which was considerably higher than that seen with strain 2308. Close to 92.1% survival was found in the PBS group (Fig. 6I). These data suggested that the erythronate and erythritol metabolic pathways work synergistically and play a crucial role in inducing placental inflammation and abortion in pregnant mice.

## DISCUSSION

In the natural host, *Brucella* spp. cause an acute severe inﬂammatory response (34), which can lead to abortion and infertility. The reproductive disease caused by *Brucella* seems crucial for the pathogen’s transmission and life cycle (35). Erythritol, a four-carbon sugar preferentially utilized by *Brucella*, has been considered as the main factor for *Brucella* colonization in genital organs. The capacity of *Brucella* to catabolize erythritol is suspected to be associated with bacterial virulence for two reasons. The first is that the abundant presence of erythritol in the placentas of goats, cows, and pigs explains *Brucella* localization and the subsequent rapid proliferation of the bacteria, eventually leading to abortion (31). The second is that *B. abortus* B19, an avirulent vaccine strain for the control of bovine brucellosis, shows sensitivity to erythritol (36 – 38). However, further research shows that erythritol is not an essential carbon source for the pathogen in the macrophage host cell (12), and the defect in erythritol metabolism of the *B. abortus* B19 is not related to its attenuated virulence in mice (39, 40), suggesting that *Brucella* utilizes not only erythritol but also other carbon sources, such as glycerol, mannitol, and inositol, that exist in genital organs of *Brucella* hosts (6, 41).

In this work, we found that VdtR negatively regulates the erythronate metabolic pathway, which is responsible for the metabolism of four-carbon acid sugars L-threonate and D-erythronate. This gene cluster is relatively conserved in *Brucella* spp. and exists in 11 *Brucella* spp. in currently available genome sequences in the GenBank database (Fig. 2C). It suggested that this novel pathway may play a conserved role in the pathogen *Brucella*. However, different species of *Brucella* appear to differ in their utilization of these acid sugars; for example, *B. abortus* and *B. suis* can utilize D-erythronate, but not L-threonate, while *B. melitensis* can utilize both acid sugars. By alignment, we found that the first step of oxidization of L-threonate into 2-oxo-tetronate is disrupted in *B. abortus*, *B. suis*, and *B. canis* because of the mutation of the LtnD, but the oxidation of D-erythronate into 2-oxo-tetronate by the DenD is highly conserved in 11 *Brucella* spp. (Fig. 2C), suggesting that the utilization of D-erythronate is more important for *Brucella* spp. than that of L-threonate. Therefore, both acid sugars are preferred carbon sources for *Brucella* spp., which utilize acid sugars and erythritol with similar levels of efficiency. However, when we analyzed the expression of the intracellular *Brucella* erythronate metabolic pathway, we found that its expression was significantly decreased, suggesting that *Brucella* can reduce the utilization of acid sugars within host cells for intracellular survival, which explains the reason for the attenuated virulence of the *vdtR* mutant with up-regulated expression of the erythronate metabolic pathway. *In vivo* and *in vitro* experiments showed that artificial over-expression of the acid sugar metabolic pathway reduced *Brucella* intracellular survival and spleen colonization in the mouse model. Abnormal activation of the erythronate metabolic pathway may result in energy depletion, as disruption of the late steps of erythritol metabolism depletes ATP and leads to growth inhibition (12), which may affect *Brucella* intracellular trafficking by injecting effectors via the Type IV secretion system in which ATP is invested. These data suggested that acid sugar utilization is strictly regulated in *Brucella*.

VdtR, a DeoR-type regulator, has a C-terminal sugar-binding site, which usually acts as a repressor after binding sugar molecules such as arabinose and xylose to dissociate N-terminal binding DNA (28, 42). As expected, D-erythronate can activate the erythronate metabolic pathway in *B. abortus* and *B. melitensis*; however, L-threonate can activate this pathway in *B. melitensis*, but not in *B. abortus*, in which *ltnD* is mutated. Using an EMSA, we found that, although a small shift in the rVdtR protein was observed after L-threonate was added, the ability of rVdtR binding to DNA did not change at all with the addition of D-erythronate or L-threonate. Thus, D-erythronate or L-threonate did not affect the binding of VdtR to the operon of the cluster but could activate the erythronate metabolic pathway, depending on whether *Brucella* could metabolize those acid sugars. Based on these data, it is highly likely that intermediates or downstream molecules of acid sugar metabolism can be recognized and bound by VdtR to activate this pathway, although this needs to be further clarified by structural biological analysis of the VdtR protein with different sugar molecules. However, in this study, we did not focus further on this investigation.

It is worth noting that D-erythronate is an oxidation product of D-erythritol or D-erythrose metabolism *in vivo*. In humans, erythritol is fleetingly oxidized to erythrose before being further oxidized to erythronate (30). The increased intake of erythritol enhances the amount of erythronate in the human body (43), suggesting that D-erythronate might be an alternative carbon source for *Brucella* in hosts deprived of the oxidation of D-erythritol or D-erythrose. In fact, *Brucella* cannot directly convert erythritol into erythronate for metabolism; erythronate in *Brucella* needs to be assimilated from the host via the MFS (BAB_RS27025) transporter (Fig. S8). Growth analysis found that *Brucella* preferred erythronate to glucose and preferred to utilize erythritol (10). The presence of erythritol in the placenta is proposed to be linked to *Brucella* localization to these sites (31, 44), but the relationship between virulence and erythritol utilization is not fully understood. *B. abortus* vaccine S19 inhibited growth by erythritol (11, 37) and can still cause abortion in cattle (13, 40). An interesting result reported by Petersen et al. showed that the replication of *B. melitensis* is inhibited within macrophages in the presence of erythritol, and erythritol encourages the bacteria to replicate extracellularly rather than intracellularly (31). In this study, we found that D-erythronate has a similar effect to erythritol in extracellular *Brucella* replication and is inhibitory to intracellular bacterial growth. Recently, Barbier et al. have used erythritol-sensitive and tolerant reporting strains to evaluate erythritol availability and *Brucella* proliferation in mouse placenta, showing that erythritol is available but not required for *B. abortus* multiplication in murine trophoblastic cells (12). We found that in pregnant mice, *Brucella* failed to utilize either erythritol or erythronate, which did not affect bacterial multiplication in the placenta, whereas when *Brucella* was unable to utilize both erythritol and erythronate, bacterial multiplication in the placenta was significantly inhibited. The results provided evidence that erythritol is not the only factor in *Brucella* localization, as disruption of erythritol metabolism did not affect *B. abortus* multiplication in mouse placenta (12, 41). Other nutrients, including erythronate, also play an important role in *Brucella* localization, proliferation and induction of placentitis in mouse models.

**Fig 7:**
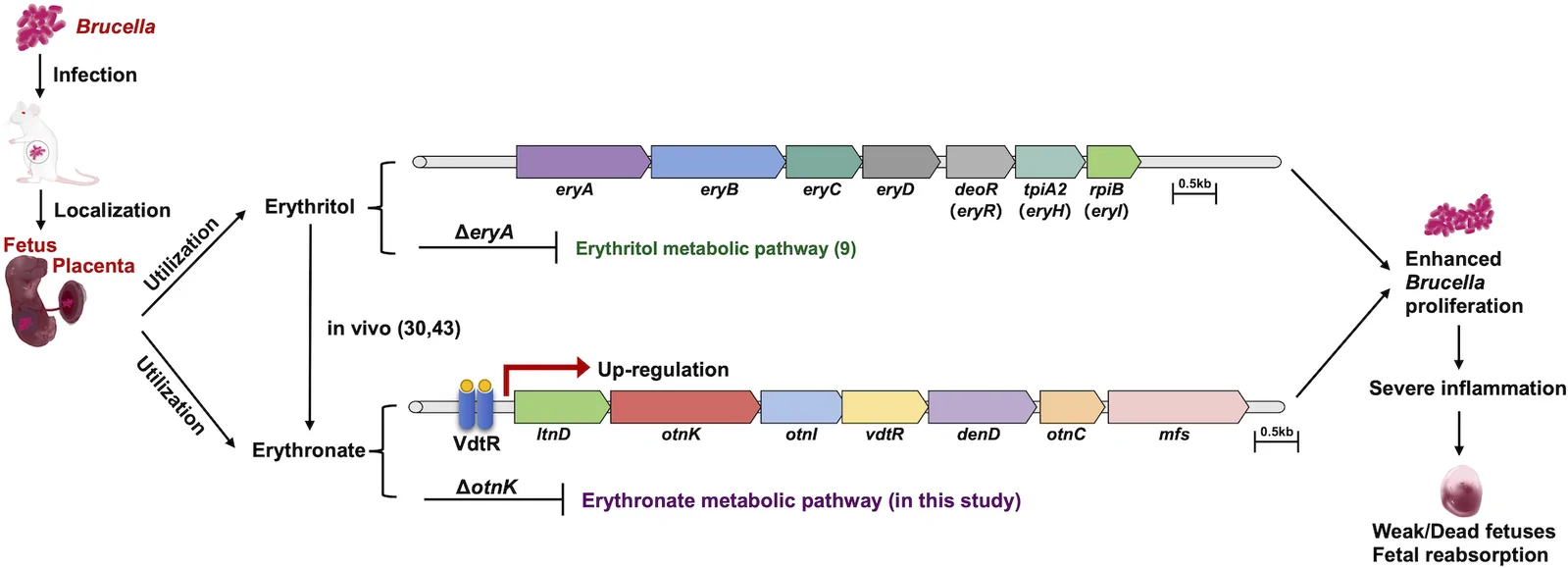
Working model of *Brucella* localization and multiplication in mouse placenta associated with preferential utilization of erythritol and erythronate. *Brucella* infects pregnant mice at a late stage and utilizes enriched erythritol and erythronate in the placenta as preferred carbon sources for colonization and rapid proliferation. Erythritol is utilized by *Brucella* through the erythritol metabolic pathway, and the compound can be oxidized to erythronate in the host. Erythronate is preferentially utilized by the erythronate metabolic pathway based on VdtR regulation. The preferred utilization of erythritol and erythronate promotes the rapid proliferation of extracellular *Brucella* in the placenta, which induces an inflammatory response and abortion.

Finally, based on the research data, we drew a working model of *Brucella* localization and multiplication in mouse placenta associated with erythritol and erythronate metabolism (Fig. 7). When the *Brucella* infects pregnant mice, enriched erythritol in the placenta is utilized by *Brucella* through the erythritol metabolic pathway. Moreover, erythritol can be oxidized into erythronate in the host, and we found that *Brucella* can preferentially utilize erythronate for replication via the erythronate metabolic pathway based on VdtR regulation. The preferred utilization of erythritol and erythronate promotes the rapid proliferation of extracellular *Brucella* in the placenta, which induces an inflammatory response and abortion.

## MATERIALS AND METHODS

### Bacterial strains, cells, and growth conditions

*B. abortus* strain 2308, *B. suis* strain 1330, *B. melitensis* strain 16M, and avirulent strain M5 were obtained from the Chinese Veterinary Culture Collection Center (Beijing, China). *E. coli* strains were routinely cultured aerobically at 37°C in lysogeny broth (LB) and on LB agar plates. The *Brucella* strain and its derivatives were routinely grown on TSB (Difco, Franklin Lakes, NJ, USA) or TSA (Difco) at 37°C under an atmosphere of 5% CO_2_. When needed, 50 µg/mL of kanamycin or 20 µg/mL of chloramphenicol was added to the broth. The RAW264.7 macrophage cell line (ATCC TIB-71, Manassas, VA, USA) was maintained in Dulbecco’s modified Eagle’s medium (DMEM, Gibco, Grand Island, NY, USA) containing 10% (vol/vol) heat-inactivated fetal bovine serum (Gibco) at 37°C in a 5% CO_2_ atmosphere.

### Construction of the deletion mutant and revertant mutant

A ~1,000-bp upstream fragment and a ~1,000-bp downstream fragment of the *vdtR* gene were amplified by PCR using the primer pairs *vdtR*-UF/UR and *vdtR*-DF/DR, respectively, and then two fragments were overlapped by PCR using the primers *vdtR*-UF and *vdtR*-DR (to avoid affecting the expression of flanking genes, the *vdtR* mutant was constructed with 43% deletion of its coding sequence containing putative DNA binding sites). The overlap PCR product was cloned into the pKB plasmid (45, 46). The recombinant suicide plasmid pKB-Δ*vdtR* was transformed into DH5α cells for amplification and then introduced into *Brucella* strains. The *vdtR* mutant was constructed by kanamycin screening and then via a SacB-assisted negative screening using 5% sucrose (45). The *otnK*, *eryA*, and *mfs* deletion mutants were constructed using a similar experimental method. The sibling strains (1330Δ*vdtR*-sibling, 16MΔ*vdtR*-sibling, and M5Δ*vdtR*-sibling strains) carrying an intact *vdtR* gene were selected by sucrose resistance and kanamycin sensitivity. The 2308Δ*vdtR* revertant strain was constructed similarly dependent on the pKB plasmid. The *vdtR* gene and its flanking fragments were amplified by PCR and inserted into the pKB plasmid, and the revertant plasmid pKB-*vdtR* was introduced into the *vdtR* mutant; then the *vdtR* gene was *in situ* restored to the *vdtR* mutant by a similar screening strategy described above. The *BAB_RS27025-RS27055* gene cluster over-expression strain 2308(gc-over) was acquired based on the broad-host-range cloning vector pBBR1MCS. A ~7-kb fragment of this gene cluster was amplified by PCR with the primers gc-over-F and gc-over-R. The recovered fragment was ligated with XbaI- and KpnI-digested pBBR1MCS, and the recombinant plasmid and the empty vector pBBR1MCS were introduced into *B. abortus* strain 2308 by electroporation and then screened by chloramphenicol resistance. The bacterial strains and plasmids used in the study are listed in Table S1, and all primers used are listed in Table S2.

### Growth curves

The growth curve of the *Brucella* strains and derivatives were determined in TSB or MM (1-g/L yeast extract, 13.2-g/L (NH_4_)_2_SO_4_, 0.1-g/L Na_2_S_2_O_3_·5H_2_O, 10-mg/L MgSO_4_, 0.1-mg/L MnSO_4_, 5-g/L NaCl, 3-g/L KH_2_PO_4_, pH = 6.8~7) supplemented with 10-mM L-threonate, 10-mM D-erythronate, 10-mM meso-erythritol, or 10-mM glucose. The original optical density at 600 nm (OD_600_) of bacterial suspensions was adjusted to 0.1 in TSB or MM supplemented with respective carbon source, then the suspensions were cultured at 37°C at 200 rpm to generate the growth curves. The OD_600_ absorbance of aliquots was measured at the indicated times until the growth of bacteria reached a stationary phase.

### Stress resistance assay

To evaluate the sensitivity of *Brucella* to H_2_O_2_, polymyxin B, and normal guinea pig serum, *Brucella* strains were cultured to the midlog phase (the value of OD_600_ = 1.0) in TSB, and then the bacterial suspension was diluted with phosphate-buffered saline to a concentration of 5 × 10^5^ CFUs/mL. Then, 50 µL of the bacterial suspension was mixed with 50 µL of the appropriate reagent. H_2_O_2_ was added to the system at a final concentration of 2 mM. Polymyxin B was added at a concentration of 1 mg/mL. In all tested groups, a negative-control group was introduced by adding 50 µL of PBS to the same bacterial suspension. The bacterial survival percentages were calculated as CFUs obtained from bacteria treated with different factors/CFUs obtained from bacteria in PBS × 100%. The experiments were performed in triplicate. The results are expressed as the mean percentage of triplicate samples ± standard deviation from one independent experiment. To test the sensitivity to sodium nitroprusside, *Brucella* strains were cultured and adjusted to a concentration of 5 × 10^9^ CFUs/mL; the suspension was serially diluted by 10-fold; and 2 µL of the solution was blotted on the TSA containing 2-mM SNP and cultured for 3–5 days at 37°C. TSA without SNP was used as the negative control.

### LPS extraction and silver staining

*B. abortus* 2308 and its derivatives were cultured to the exponential phase in TSB. The bacterial LPS was extracted using an LPS Extraction Kit (iNtRON, Seoul, Korea). All samples were loaded and separated by 12.5% SDS polyacrylamide gel. A silver staining assay was performed as previously described (45). Images were acquired with a digital camera (Nikon).

### SDS-PAGE

The protein samples were mixed with 5 × SDS loading buffer (0.25-M Tris buffer, pH 6.8, 10% SDS, 5% β-mercaptoethanol, 50% glycerol, and 0.5% bromphenol blue) and boiled for 10 min. Samples were subjected to SDS-PAGE using 12.5% resolving gels at 80 V for 2 h. The gel was stained with Coomassie Brilliant Blue Staining Solution and depigmented by de-staining solution (30% methanol, 10% acetic acid). The gels were scanned using an Odyssey Infrared Imaging System (LI-COR Biosciences, Lincoln, NE, USA) to obtain images. To detect whether VdtR interacts directly with L-threonate or D-erythronate, we evaluated the electrophoretic mobility of rVdtR protein with or without L-threonate or D-erythronate under non-reducing SDS-PAGE gel conditions (no β-mercaptoethanol was added in the 5 × SDS loading buffer). The molar concentration ratio of acidic sugar to protein was 100:1, and 100 mM Mg^2+^ was added as the negative control.

### Cell infection

Cell infection was performed in RAW 264.7 macrophages cultured in DMEM with 10% fetal bovine serum (FBS). Twenty-four-well plates were seeded with 5 × 10^5^ cells/well, and macrophages were synchronously infected at a multiplicity of infection (MOI) of 100:1 (bacteria to cells) by centrifuging the plates at 400 × *g* for 5 min. After incubation for 1 h at 37°C under a 5% CO_2_ atmosphere, extracellular bacteria were killed by gentamycin treatment (100 µg/mL) for 1 h. Then, fresh DMEM supplemented with 20-µg/mL gentamycin was added and cultures were incubated. At 1, 8, 24 and 48 h, cells were washed three times with PBS and lysed with 0.2% (vol/vol) Triton X-100 in PBS, and 100-µL lysate was serially diluted 10-fold and plated on TSA to determine the bacterial CFUs. All experiments were performed in triplicate.

To determine the role of erythritol, erythronate, or glucose in *Brucella* intracellular or extracellular survival, RAW 264.7 macrophages were infected with *B. abortus* strain 2308 at a multiplicity of infection of 100:1 as described above. The extracellular bacteria were killed using RPMI 1640 (Gibco) containing 100-µg/mL gentamycin for 1 h. To determine the intracellular survival of *Brucella*, the medium was replaced with RPMI containing 20-µg/mL gentamycin and 1% glucose or erythritol or D-erythronate (wt/vol) to maintain cell cultures. Enumeration of bacteria within the cells was determined at 2, 24, and 48 h p.i. as described above. To test the survival of extracellular *Brucella*, RAW 264.7 macrophages were infected with *B. abortus* strain 2308 as described above. After killing extracellular bacteria, cells were washed and the medium was replaced with RPMI containing 1% glucose, erythritol, or D-erythronate (wt/vol). Supernatant samples were collected at 2, 24, and 48 h to determine the number of extracellular bacteria. Meanwhile, cells were lysed and plated in TSA to determine the number of intracellular bacteria. The ratio of extracellular-to-intracellular bacteria in each well was determined at the indicated times. The experiments were performed in triplicate.

### Indirect immunofluorescence

Macrophages were infected with *Brucella* strains at MOI of 100:1 in 24-well plates preloaded with 14-mm glass coverslips (Nest, Wuxi, China). At 4, 24, and 48 h p.i., the infected cells were washed twice with PBS and fixed overnight in 4% (wt/vol) paraformaldehyde, and then the coverslips were incubated with Rabbit anti-*Brucella* polyclonal antibody (1:1,000 dilution, prepared in our lab) or mouse anti-*Brucella* polyclonal antibody (1:1,000 dilution, prepared in our lab) to track intracellular bacteria. Rat anti-LAMP1 monoclonal antibody (clone 1D4B, 1:1,000 dilution; Abcam, Cambridge, UK) and rabbit anticalnexin polyclonal antibody (1:200 dilution; Enzo, Farmingdale, NY, USA) were used as primary antibodies for tracking lysosomes and endoplasmic reticulum, respectively. All antibodies were diluted in PBS buffer (pH 7.4) with 0.5% bovine serum albumin and incubated in a humidity chamber for 1 h. After that, the coverslips were washed with PBS (pH 7.4) and Alexa Fluor 488-conjugated goat anti-rabbit IgG and Alexa Fluor 555-conjugated goat anti-rat or anti-mouse IgG (Thermo Fisher Scientific, Waltham, MA, USA) were used as secondary antibodies at dilutions of 1:1,000. The coverslips were incubated with 0.2-mg/mL 4',6-diamidino-2-phenylindole (DAPI) at room temperature to stain DNA. Finally, the samples were mounted on slides using a fluorescent mounting medium (Yeasen, Shanghai, China). The samples were observed under a Zeiss LSM880 laser scanning confocal microscope for image acquisition (Zeiss, Jena, Germany). The intracellular co-localization data and images of 1,024 × 1,024 pixels were acquired and assembled by image analysis using Adobe Photoshop CS4 (Adobe Systems Incorporated, San Jose, CA, USA). To determine the percentage of bacteria positive for the LAMP-1, 100 intracellular bacteria were counted randomly. Data are representative of at least three independent experiments.

### RNA isolation, RNA-seq analysis, and qPCR

Total RNA was extracted from *Brucella* strains using the RiboPure-Bacteria kit (Ambion, Foster City, CA, USA). For RNA-seq analysis, *B. abortus* strain 2308 and its derivatives were cultured to the midlog phase (the value of OD_600_ = 1.0) in TSB, and their RNA was extracted by the RiboPure-Bacteria kit (Ambion). The RNA-seq library was prepared following the TruSeq RNA sample preparation Kit from Illumina Inc. (San Diego, CA, USA). Ribosomal RNA depletion is performed by Ribo-Zero Magnetic kit (Epicentre Biotechnologies, Madison, WI, USA), and double-stranded cDNA was synthesized using a SuperScript double-stranded cDNA synthesis kit (Invitrogen, Carlsbad, CA, USA) with random hexamer primers (Illumina Inc.). RNA-seq sequencing library was sequenced with the Illumina HiSeq × TEN; the processing of original images to sequences, base-calling, and quality value calculations were performed using the Illumina GA Pipeline (version 1.6). The data generated from the Illumina platform were used for bioinformatics analysis. All of the analyses were performed using the free online Majorbio Cloud Platform (www.majorbio.com) from Shanghai Majorbio Bio-Pharm Technology Co. Ltd. (Shanghai, China). For each dataset and for each alignment and quantification protocol, we analyzed differential expression using the edgeR, DESeq2, and DESeq packages.

For qPCR, total RNA was isolated from *Brucella* strains or *Brucella*- infected RAW264.7 cells or tissues using the TRIzol reagent (Invitrogen), and DNA contamination was removed using a Turbo DNA-free kit (Ambion). RNA was reverse transcribed using the PrimeScript RT reagent kit (Takara Bio, Inc., Shiga, Japan) at 37°C for 15 min, then at 85°C for 5 s for cDNA templates. A 2 × AceQ Universal U^+^ Probe Master Mix V2 (Vazyme, Nanjing, China) was used for TaqMan qPCR, and 2× ChamQ Universal SYBR qPCR Master Mix (Vazyme) was used for SYBR Green qPCR. Reactions were carried out on a Mastercycler ep Realplex system (Eppendorf AG, Hamburg, Germany). TaqMan qPCR for host cytokine or chemokine expression analysis was performed at 37°C for 2 min, and then at 95°C for 5 min, followed by 40 cycles at 95°C for 10 s and at 60°C for 30 s. The SYBR Green qPCR for *Brucella* genes expression analysis was performed at 95°C for 30 s, followed by 40 cycles at 95°C for 10 s, and at 60°C for 30 s. For each gene, qPCR was performed in triplicate, and relative transcription levels were determined by the 2^−ΔΔCt^ method using 16S for *Brucella* or beta-actin for mice as an internal control for data normalization. All primers and TaqMan probes used for qPCR are listed in Table S2.

### Co-transcriptional analysis

To detect whether the gene *BAB_RS27025-55* in the cluster was co-transcribed, the primers for the bridging fragments F1, F2, F3, F4, and F5 between the *BAB_RS27025-55* gene were designed (Fig. 2F). *B. abortus* 2308 genomic DNA and RNA were extracted using a TIANamp Bacteria DNA kit (TIANGEN, Beijing, China) and RiboPure-Bacteria kit (Ambion), respectively. RNA was reverse transcribed to cDNA by RT-PCR, and nucleic acid gel electrophoresis was performed to detect whether the cDNA of each bridging fragment was expressed, as well as to detect whether the RNA had DNA contamination. The genomic DNA of each articulated fragment was used as a positive control.

### Alignment of protein sequences

Amino acid sequences of *BAB_RS27025-55* in 11 species in *Brucella* spp. were downloaded from the GenBank database for comparison. For the amino acid sequence alignment, a Protein Blast (https://blast.ncbi.nlm.nih.gov/Blast.cgi) and Clustal X2 (47) (http://www.clustal.org) were used, and the sequence alignment results were displayed via JalView version 2 (48) (http://www.jalview.org/).

### Protein purification

The *vdtR* gene was amplified by PCR from the genome of *B. abortus* 2308 using the primers rVdtR-F and rVdtR-R, then inserted into pCold-TF vectors (Takara), which allow expression of N-terminal His-tagged proteins in *E. coli* BL21 (TIANGEN). A culture of BL21(pColdTF-VdtR) was grown in LB to OD_600_ = 0.6~0.8, and expression was then induced with 1-mM β-D-thiogalactopyranoside (TIANGEN) for an additional 24-h culture at 16°C. The recombinant protein rVdtR was purified according to standard protocols of BeaverBeads His-Tag (Beaver, Suzhou, China).

### Promoter activity analysis

The promoter region (~300 bp) of genes in the cluster (*BAB_RS27025-RS27055*) was amplified by PCR. The PCR products were purified by gel extraction and ligated into the KpnI- and BamHI-digested pMCR-LacZ plasmid using the EasyGeno Assembly Cloning kit (TIANGEN) to construct the LacZ reporter plasmids. The successfully constructed recombinant plasmids were electroporated into 2308, 2308Δ*vdtR*, and 2308Δ*vdtR*-Rev, while the empty pMCR-LacZ plasmid was also transformed into *Brucella* strains as a negative control. LacZ activation was determined using o-nitrophenyl-β-d-galactoside as the substrate described in our previous study (46).

### Electrophoretic mobility shift assay

A 369-bp predicted promoter region of *ltnD* gene (*BAB_RS27055*) was amplified by PCR. A 205-bp fragment of negative control was obtained from a complementary sequence of *egfp* gene. Two fragments were ligated with linearized plasmid pUC19, respectively, and then the recombinant plasmids pUC19-P*_ltnD_* and pUC19-Neg were constructed. The Cy5.5-labeled probe was acquired by PCR using Cy5.5-pUC19-F/R as primer and pUC19-P*_ltnD_* as a template; non-labeled specific competition probes and non-specific competition probes were obtained using pUC19-F/R as primer and pUC19-P*_ltnD_* and pUC19-Neg as templates, respectively.

To identify the binding site in the promoter region by rVdtR, the *ltnD* promoter was truncated to different length fragments (Fig. S3A). Firstly, the 369-bp fragment was truncated to a 180-bp fragment as P1, a 109-bp fragment as P2, and a 180-bp fragment as P3. The Cy5.5-labeled P1, P2, and P3 fragments were constructed by PCR from the pUC19-P*_ltnD_* plasmid using the primers Cy5.5-P1-F/R, Cy5.5-P2-F/R, and Cy5.5-P3-F/R, respectively; the Cy5.5-labeled P2 + P3 fragment was constructed using P2-F/P3-R; then, the P3 fragment was further shortened to different length fragments (P4–P8), and Cy5.5-labeled P4–P8 probes were obtained by PCR using the forward primer Cy5.5-P4/5/6/7/8-F and the reverse primer Cy5.5-pUC19-R.

EMSA was performed using a LightShift EMSA Optimization and Control Kit (Thermo Fisher Scientific), and each reaction system was composed of 1× binding buffer, 5-mM MgCl_2_ (100mM), 0.05%NP40, 2.5% glycerol, 50-ng/µL Poly (dI-dC), 0.05-pmol/µL Cy5.5-labeled probes, and rVdtR protein at different indicated concentrations; ddH_2_O was added to make the final volume 20 µL. In the competitive reaction system, 5-pmol/µL non-labeled specific competition probes or non-specific competition probes were added to assess the specificity of rVdtR binding to the *ltnD* promoter probe. Samples were incubated at room temperature for 20 min and electrophoresed on a 5% non-denaturing polyacrylamide gel in 0.5× TBE, which had pre-electrophoresed at 100 V for 30 min at 4°C. Gels were scanned using an Odyssey Infrared Imaging System (LI-COR). EMSAs were repeated at least three times with similar results. EMSA was also carried out to assess the effect of L-threonate or D-erythronate on rVdtR binding to the *ltnD* promoter probe. rVdtR (1.5 µg) was added and incubated with glucose, D-erythronate, or L-threonate in the reaction system, and EMSA was performed as described above.

### Mice infections

Six- to eight-week-old female BALB/c mice were obtained from Shanghai JieSiJie Laboratory Animal Co., Ltd. (Shanghai, China). Five mice for each group were inoculated intraperitoneally with 1 × 10^5^ CFU of *Brucella* strain or its derivatives. At the indicated times, samples of spleen were collected aseptically, weighed, and homogenized with 3-mL PBS. Tissue suspension (100 µL) was serially diluted 10-fold and plated on TSA to determine bacterial CFUs.

For pregnant mice infection, groups of three to six pregnant mice, obtained from the Laboratory Animal Centre in Yangzhou University (Yangzhou, China), were infected intraperitoneally with 1 × 10^5^ CFU of *B. abortus* 2308, 2308Δ*otnK*, 2308Δ*eryA*, or 2308Δ*eryA-otnK* at 5 days of gestation. At 13 days post-infection (corresponding to day 18 of gestation), the viability of fetuses was evaluated based on the presence of fetal size and skin color. Fetuses were classified as viable if they exhibited visible blood vessels and bright pink skin and were of normal size for their gestational period. Fetuses were judged as non-viable if they lacked visible blood vessels, had pale or opaque skin, were undersized for their gestational period when compared to their littermates, or showed signs of fetal reabsorption. The equation [(number viable fetuses per litter/total number of fetuses per litter) × 100] was used to calculate the percentage of viability. In addition, sera of infected mice were collected and the levels of the pro-inflammatory cytokines IL-6 and TNF-α was detected by the LEGEND MAX Mouse IL-6 and TNF-α ELISA kit; the spleen and placenta were collected aseptically at necropsy for bacteriology and gene expression analysis, and placenta samples were also collected for blinded histopathological analysis. The method of bacteriology analysis is described above. The method of blinded histopathological analysis is described below.

### Histopathology

Samples of placenta were collected and fixed in 10% formalin for more than 24 h. Formalin-fixed sections of placenta were stained with hematoxylin and eosin as described (49). Neutrophil infiltration, trophoblast death, or coagulative necrosis and vascular lesions were some of the main indicators observed for placental sections.

### Statistical analysis

Statistical analysis was performed using GraphPad Prism version 9.0 software (Graph Pad Software, San Diego, CA, USA). Statistical significance was determined using an unpaired *t*-test or multiple unpaired *t*-test. For group analysis, one- or two-way analysis of variance followed by Dunnett’s multiple comparisons test was used. For all comparisons, a probability (*P*) value of <0.05 was considered statistically significant.

## Data Availability

Supplementary materials for this article were online submitted to support this research. This study includes no data deposited in external repositories. Original data and materials will be available from the corresponding author upon request.

## SUPPLEMENTAL MATERIAL

Supplemental figures spectrum.02074-23-s0001.pdf Figures S1 to S8.

Supplemental dataset spectrum.02074-23-s0002.pdf Dataset S1.

Supplemental tables spectrum.02074-23-s0003.pdf Tables S1 and S2.

ASM does not own the copyrights to Supplemental Material that may be linked to, or accessed through, an article. The authors have granted ASM a non-exclusive, world-wide license to publish the Supplemental Material files. Please contact the corresponding author directly for reuse.
