# Supplementary material for: Erythronate utilization activates VdtR regulating its metabolism to promote Brucella proliferation, inducing abortion in mice
Source: Microbiol Spectr. 2023 Sep 6;11(5):e02074-23. doi: 10.1128/spectrum.02074-23 (PMC10580937; doi:10.1128/spectrum.02074-23)
Supplement: Supplemental figures — Figures S1 to S8. [file spectrum.02074-23-s0001.pdf]

Supplementary Information Figures

Figure S1

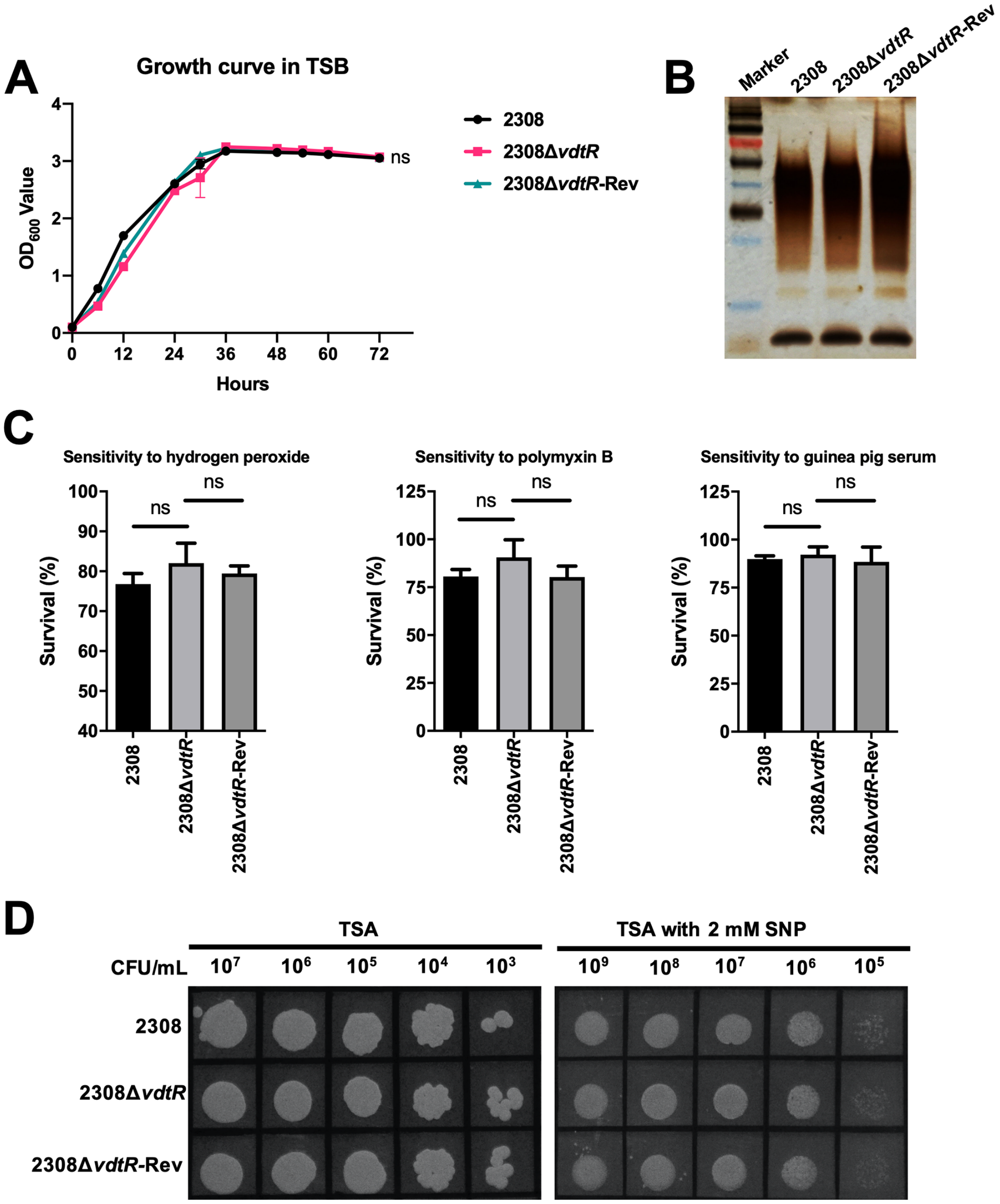

Figure S1. Deletion of *B. abortus vdtR* does not affect the bacterial phenotype and ability to resist killing by bactericidal factors. (**A**) Determination of bacterial growth curves in TSB. (N=3, mean ± SD, ns, not significant by unpaired t-test). (**B**) Identification of LPS by silver staining. (**C**) The sensitivity of *B. abortus* strain 2308, the mutant strain 2308Δ*vdtR* and the revertant strain 2308Δ*vdtR*-Rev to hydrogen peroxide, polymyxin B, and guinea pig serum. (N=3, mean ± SD, ns, not significant by unpaired t-test). (**D**) The sensitivity of 2308, 2308Δ*vdtR* and 2308Δ*vdtR*-Rev strains to SNP.

Figure S2

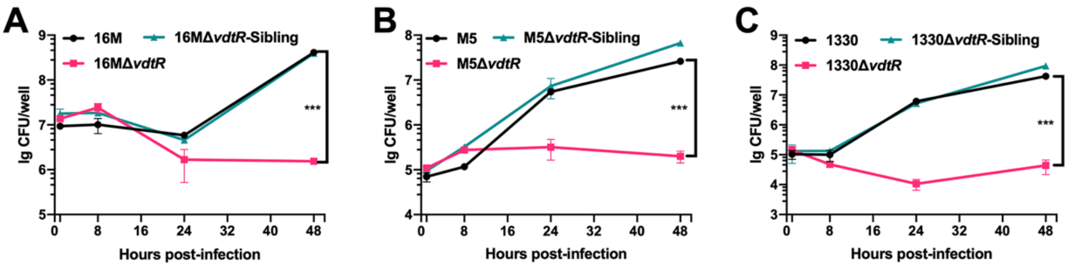

Figure S2. Intracellular survival of different *Brucella* strains within RAW264.7 macrophages. (**A**) Intracellular survival of *B. melitensis* 16M, its deletion mutant 16MΔ*vdtR*, and its sibling revertant mutant 16MΔ*vdtR*-Sibling in RAW264.7 macrophages. (**B**) Intracellular survival of *B. melitensis* M5, its deletion mutant M5Δ*vdtR*, and its sibling revertant mutant M5Δ*vdtR*-Sibling in RAW264.7 macrophages. (**C**) Intracellular survival of *B. suis* 1330, its deletion mutant 1330Δ*vdtR*, and its sibling revertant mutant 1330Δ*vdtR*-Sibling in RAW264.7 macrophages. Cells were infected with *Brucella* strains and intracellular CFUs were enumerated at indicated time points. Data shown are means ± SD from a representative experiment performed in triplicates (*N*=3, mean ± SD, ***, $p < 0.001$ by two-way ANOVA).

Figure S3

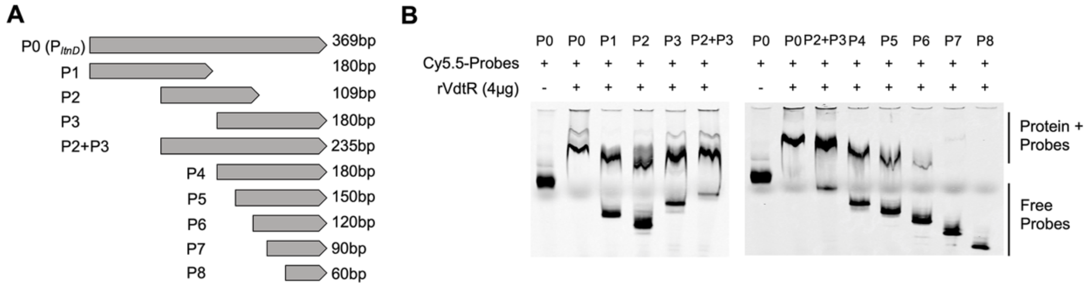

Figure S3. Analysis of the binding site in the *ltnD* promoter to the rVdtR. (**A**) The different truncated fragments of the *ltnD* promoter as nucleotide probes, was labeled by PCR amplification using Cy5.5-labeled primers. (**B**) EMSA experiments showing multiple binding of the rVdtR to regions in the *ltnD* promoter. In the first lane, no rVdtR was added to the binding reaction, and in the subsequent lanes, rVdtR and different truncated Cy5.5-labeled probes were added.

Figure S4

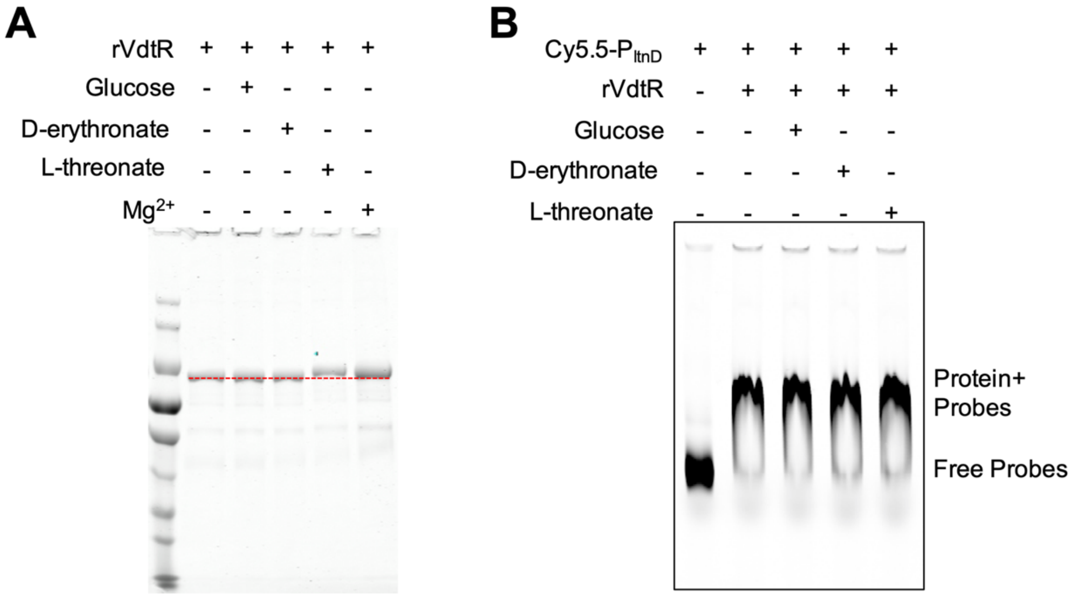

Figure S4. D-erythronate and L-threonate did not affect the rVdtR binding to the ltnD promoter. (**A**) The protein electrophoretic mobility shift of rVdtR protein with or without L-threonate or D-trythronate was evaluated under non-reducing SDS-PAGE gel conditions. L-threonate slightly affects electrophoretic mobility shift of the rVdtR protein at molar concentration ratio of 1:100 (Protein: Sugar). (**B**) EMSA was performed to assess the effect of L-threonate or D-erythronate on rVdtR binding to the *ltnD* promoter probe. In the first lane, no rVdtR was added to the binding reaction, and in the subsequent lanes, rVdtR and different sugars were added at molar concentration ratio of 1:10 (Protein: Sugar).

Figure S5

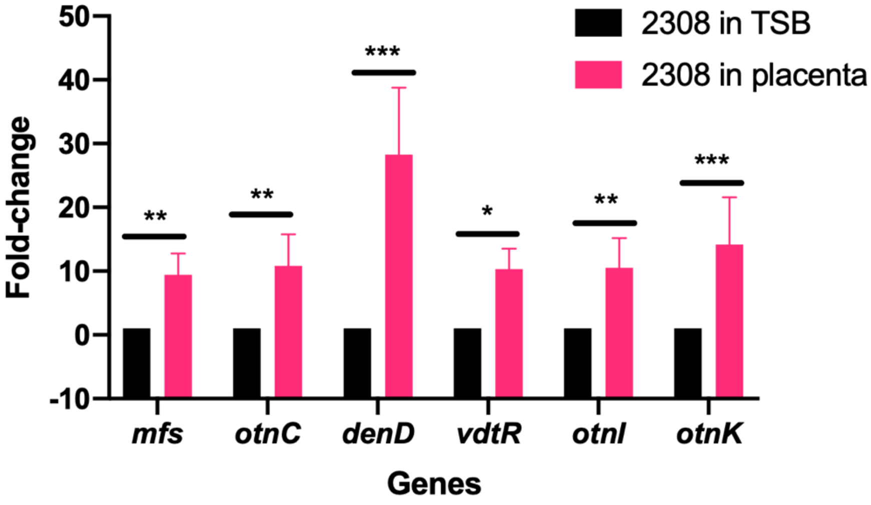

Figure S5. qPCR analysis. Expression of six component genes (not include the *ltnD* gene due to its mutation in *B. abortus*) of the erythronate metabolic pathway in *Brucella* were significantly up-regulated post infection of placenta. (N=3 to 5, mean ± SD, *, $p < 0.05$; **, $p < 0.01$; ***, $p < 0.001$ by two-way ANOVA).

Figure S6

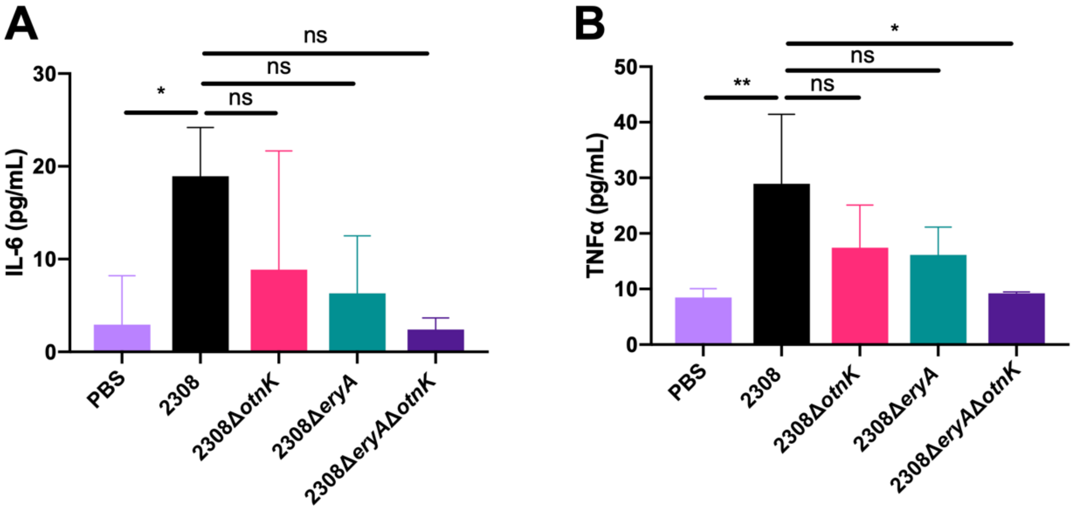

Figure S6. Determination of the cytokines in the serum of pregnant mice infected by *B. abortus* 2308 and its derivatives at 13 days post infection. (A) IL-6; (B) TNFα. (N=3 to 5, mean ± SD, *, $p < 0.05$; **, $p < 0.01$; ns, not significant by two-way ANOVA).

Figure S7

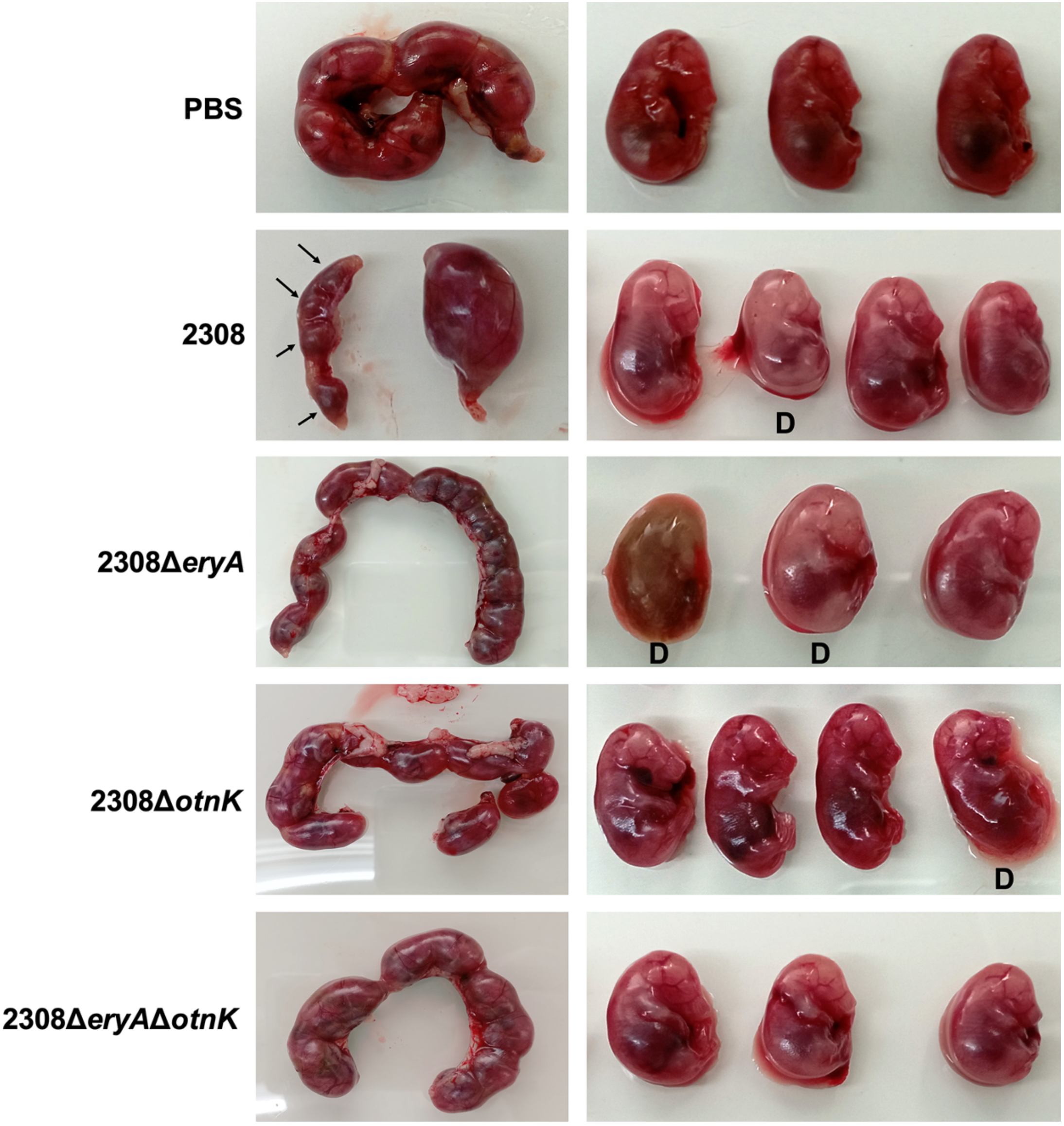

Figure S7. Macroscopic features of gravid uterus and fetuses in pregnant mice infected by *B. abortus* 2308 and its derivatives at 13 days post infection. *B*. *abortus* 2308 infection caused fetal absorption (panel 2, left) and dead/weak fetuses (panel 2, right); 2308Δ*eryA* and 2308Δ*otnK* infection induced no fetal absorption (panels 3 and 4, left), but dead/weak fetuses (panels 3 and 4, right); 2308Δ*eryA*Δ*otnK* (panel 5) infection induced no fetal absorption, nor the dead/weak fetuses, as the PBS control (panel 1). Arrow, Fetal absorption. D, Dead or weak fetuses.

Figure S8

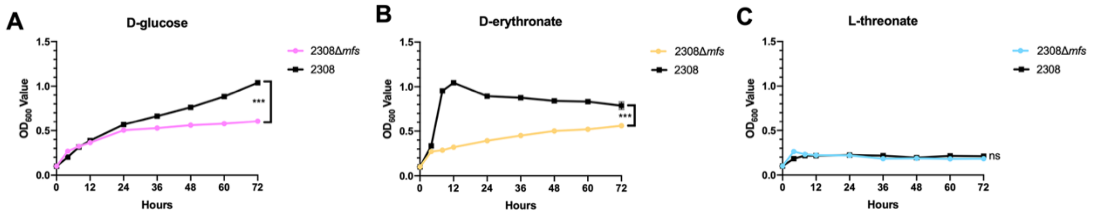

Figure S8. *mfs* is involved in the metabolism of D-erythronate. Growth curve of 2308 and 2308Δ*mfs* with 10 mM D-glucose (A), 10 mM D-erythronate (B), and 10 mM L-threonate (C). (N=3, mean ± SD, ***, $p < 0.00$; ns, not significant by unpaired *t*-test).
