# Supplementary material for: Erythronate utilization activates VdtR regulating its metabolism to promote Brucella proliferation, inducing abortion in mice
Source: Microbiol Spectr. 2023 Sep 6;11(5):e02074-23. doi: 10.1128/spectrum.02074-23 (PMC10580937; doi:10.1128/spectrum.02074-23)
Supplement: Supplemental dataset — Dataset S1. [file spectrum.02074-23-s0002.pdf]

Genes up-regulated in 2308Δ*vdtR* comparing to 2308

Gene_id	Gene name	Gene description	FC(2308ΔvdtR/2308)	Log2FC(vdtR/S2308)	Pvalue	Padjust	Significant	Regulate	Type	2308_1	2308_2	2308_3	2308ΔvdtR_1	2308ΔvdtR_2	2308ΔvdtR_3	2308	2308ΔvdtR	COG_id	COG_Functional Categories	GO_id	GO_term
BAB_RS27055	-	oxidoreductase	98.532	6.622524871	5.84136E-16	9.38415E-13	yes	up	mRNA	74.23	75.63	82.96	6903.39	7369.65	7450.57	77.6067	7241.2033	COG2084	S:Function unknown	GO:0051287;GO:0004616;GO:0003858;GO:0051287;GO:0004616	MF:NAD binding;MF:phosphogluconate dehydrogenas;MF:3-hydroxybutyrate dehydrogenase activity;MF:NAD binding;MF:phosphogluconate dehydrogenas;
BAB_RS27045	-	hydroxypyruvate isomerase	89.567	6.484888206	1.47265E-16	4.73162E-13	yes	up	mRNA	67.33	62.42	67.91	5436.11	5583.64	5728.62	65.8867	5582.79	COG3622	G:Carbohydrate transport and metabolism	GO:0004519;GO:0016853;GO:0004519;GO:0008903;GO:0016853;GO:0004519;GO:0046872;GO:0008903;GO:0016853;GO:0016853	MF:endonuclease activity;MF:isomerase activity;MF:endonuclease activity;MF:hydroxypyruvate isomerase activity;MF:isomerase activity;MF:endonuclease activity;MF:metal ion binding;MF:hydroxypyruvate isomerase activity;MF:isomerase activity;MF:isomerase activity;
BAB_RS27050	-	four-carbon acid sugar kinase family protein	86.947	6.442070888	1.20369E-15	9.66863E-13	yes	up	mRNA	18.11	19.86	23.05	1644.76	1673	1721.15	20.34	1679.6367	COG3395	S:Function unknown	------	------
BAB_RS27035	-	hypothetical protein	37.787	5.239824764	8.39366E-16	8.98961E-13	yes	up	mRNA	38.65	38.66	38.25	1362.91	1354.05	1411.05	38.52	1376.0033	COG0451	G:Carbohydrate transport and metabolism	GO:0006694;GO:0006694;GO:0003854;GO:0003854;GO:0047577	BP:steroid biosynthetic process;BP:steroid biosynthetic process;MF:3-beta-hydroxy-delta5-steroid dehydrogenase activity;MF:3-beta-hydroxy-delta5-steroid dehydrogenase activity;MF:4-hydroxybutyrate dehydrogenase activity;
BAB_RS27025	-	MFS transporter	29.387	4.877108992	1.20638E-14	7.75222E-12	yes	up	mRNA	59.57	53.16	55.02	1501.34	1474.28	1663.57	55.9167	1546.3967	**ENOG410XNQ6**	G:Carbohydrate transport and metabolism	GO:0055085;GO:0055085;GO:0016021;GO:0016021	BP:transmembrane transport;BP:transmembrane transport;CC:integral component of membrane;CC:integral component of membrane;
BAB_RS27030	-	aldolase	28.785	4.847238206	2.81088E-14	1.50523E-11	yes	up	mRNA	28.38	33.78	34.61	923.95	850.49	895.32	32.2567	889.92	COG0235	G:Carbohydrate transport and metabolism	------	------
BAB_RS27040	-	DeoR/GlpR transcriptional regulator	7.364	2.880559084	2.29163E-12	1.05186E-09	yes	up	mRNA	39.84	32.07	36.3	255.62	235.53	258.96	36.07	250.03667	COG1349	G:Carbohydrate transport and metabolism	GO:0006351;GO:0005622;GO:0003700;GO:0003677	BP:transcription, DNA-templated;CC:intracellular;MF:transcription factor activity, sequence-specific DNA binding;MF:DNA binding;
BAB_RS29675	-	peptide ABC transporter substrate-binding protein	4.394	2.135652646	2.39629E-11	8.55476E-09	yes	up	mRNA	973.13	897.77	892.78	3770.44	3702.79	3981.97	921.227	3818.4	COG4166	S:Function unknown	GO:0055085;GO:0055085;GO:0043190;GO:0043190	BP:transmembrane transport;BP:transmembrane transport;CC:ATP-binding cassett;CC:ATP-binding cassett;
BAB_RS27060	-	flagellar basal body rod protein FlgB	4.25	2.087403364	7.0261E-08	8.06245E-06	yes	up	mRNA	6.18	5.49	7.33	23.73	24.06	29.54	6.33333	25.776667	COG1815	N:Cell motility	GO:0071973;GO:0030694	BP:bacterial-type flagellum-dependent cell motility;CC:bacterial-type flagellum basal body, rod;
BAB_RS27070	-	flagellar hook-basal body complex protein FliE	3.621	1.856215994	0.006189939	0.028740283	yes	up	mRNA	4.32	9.04	0.59	16.23	11.77	19.67	4.65	15.89	COG1677	N:Cell motility	GO:0071973;GO:0071973;GO:0071973;GO:0009425;GO:0009425;GO:0009425;GO:0003774;GO:0005198;GO:0003774;GO:0005198;GO:0003774;GO:0005198	BP:bacterial-type flagellum-dependent cell motility;BP:bacterial-type flagellum-dependent cell motility;BP:bacterial-type flagellum-dependent cell motility;CC:bacterial-type flagellum basal body;CC:bacterial-type flagellum basal body;CC:bacterial-type flagellum basal body;MF:motor activity;MF:structural molecule activity;MF:motor activity;MF:structural molecule activity;MF:motor activity;MF:structural molecule activity;
BAB_RS27820	-	urocanate hydratase	3.549	1.827298913	4.47223E-08	5.74771E-06	yes	up	mRNA	171.33	171.48	174.63	456.65	564.21	711.63	172.48	577.49667	COG2987	E:Amino acid transport and metabolism	GO:0019556;GO:0019557;GO:0019556;GO:0019557;GO:0005737;GO:0005737;GO:0016153;GO:0016153;GO:0016757	BP:histidine catabolic process to glutamate and formamide;BP:histidine catabolic process to glutamate and formate;BP:histidine catabolic process to glutamate and formamide;BP:histidine catabolic process to glutamate and formate;CC:cytoplasm;CC:cytoplasm;MF:urocanate hydratase activity;MF:urocanate hydratase activity;MF:transferase activity, transferring glycosyl groups;
BAB_RS33070	-	hypothetical protein	3.443	1.783707413	0.024149965	0.07354866	no	up	mRNA	4.32	2.91	3.71	9.13	14.31	17.72	3.64667	13.72	------	------	------	------
BAB_RS27830	-	histidine ammonia-lyase	3.397	1.76432427	3.44856E-09	7.38682E-07	yes	up	mRNA	227.14	247.13	239.66	646.1	836.25	808.93	237.977	763.76	COG2986	E:Amino acid transport and metabolism	GO:0019556;GO:0019557;GO:0019556;GO:0019557;GO:0005737;GO:0005737;GO:0004397;GO:0004397;GO:0016491	BP:histidine catabolic process to glutamate and formamide;BP:histidine catabolic process to glutamate and formate;BP:histidine catabolic process to glutamate and formamide;BP:histidine catabolic process to glutamate and formate;CC:cytoplasm;CC:cytoplasm;MF:histidine ammonia-lyase activity;MF:histidine ammonia-lyase activity;MF:oxidoreductase activity;
BAB_RS29680	-	peptide ABC transporter substrate-binding protein	3.349	1.743862776	1.65962E-10	4.84759E-08	yes	up	mRNA	324.7	292.18	298.09	925.15	921.88	1040.44	304.99	962.49	COG4166	S:Function unknown	GO:0055085;GO:0043190	BP:transmembrane transport;CC:ATP-binding cassett;
BAB_RS27825	-	N-formylglutamate deformylase	3.321	1.731792576	2.65312E-10	7.10372E-08	yes	up	mRNA	124.85	138.05	137.76	390.77	448.09	424.15	133.553	421.00333	COG3741	E:Amino acid transport and metabolism	GO:0016787;GO:0050415;GO:0016787;GO:0050129	MF:hydrolase activity;MF:formimidoylglutamase activity;MF:hydrolase activity;MF:N-formylglutamate deformylase activity;
BAB_RS32750	-	hypothetical protein	3.181	1.669699111	0.009185902	0.037502291	yes	up	mRNA	1.35	1.87	4.26	9.23	7.29	10.91	2.49333	9.1433333	ENOG4111WBA	S:Function unknown	------	------
BAB_RS29690	-	peptide ABC transporter permease	3.177	1.667862798	4.61693E-11	1.48342E-08	yes	up	mRNA	247.59	226.66	235.79	663.02	721.83	744.07	236.68	709.64	COG1173	E:Amino acid transport and metabolism	GO:0055085;GO:0055085;GO:0016021;GO:0005886;GO:0016021;GO:0005886	BP:transmembrane transport;BP:transmembrane transport;CC:integral component of membrane;CC:plasma membrane;CC:integral component of membrane;CC:plasma membrane;
BAB_RS27815	-	hypothetical protein	3.164	1.661874238	2.00727E-07	1.74307E-05	yes	up	mRNA	93.14	95.77	92.28	230.37	260.64	354.37	93.73	281.79333	COG3758	S:Function unknown	------	------
BAB_RS29685	-	ABC transporter	3.092	1.628382119	4.35326E-10	1.07593E-07	yes	up	mRNA	366.23	318.18	313.57	926.55	956.15	1031.42	332.66	971.37333	COG0601	E:Amino acid transport and metabolism	GO:0055085;GO:0055085;GO:0016021;GO:0005886;GO:0016021;GO:0005886	BP:transmembrane transport;BP:transmembrane transport;CC:integral component of membrane;CC:plasma membrane;CC:integral component of membrane;CC:plasma membrane;
BAB_RS29695	-	ABC transporter ATP-binding protein	2.922	1.547038466	2.36325E-11	9.49141E-09	yes	up	mRNA	257.17	246.62	266.18	674.16	726.16	719.5	256.657	706.60667	COG4172	E:Amino acid transport and metabolism	GO:0015833;GO:0015833;GO:0005524;GO:0016887;GO:0005524;GO:0016887	BP:peptide transport;BP:peptide transport;MF:ATP binding;MF:ATPase activity;MF:ATP binding;MF:ATPase activity;
BAB_RS27840	-	formimidoylglutamate deiminase	2.823	1.497217299	4.57335E-09	9.18385E-07	yes	up	mRNA	88.16	74.74	80.2	188.23	233.8	223.62	81.0333	215.21667	COG0402	Q:Secondary metabolites biosynthesis, transport and catabolism	GO:0016810;GO:0046872;GO:0050416;GO:0016810	MF:hydrolase activity, acting on carbon-nitrogen;MF:metal ion binding;MF:formimidoylglutamate deiminase activity;MF:hydrolase activity, acting on carbon-nitrogen;
BAB_RS27835	-	imidazolonepropionase	2.356	1.236181828	2.77201E-08	4.24118E-06	yes	up	mRNA	257.51	232.36	256.09	472.79	614.1	569.61	248.653	552.16667	COG2986;COG1228	E:Amino acid transport and metabolism;Q:Secondary metabolites biosynthesis, transport and catabolism	GO:0019556;GO:0019557;GO:0019556;GO:0019557;GO:0019556;GO:0019557;GO:0005737;GO:0005737;GO:0016829;GO:0050480;GO:0005506;GO:0046872;GO:0008270;GO:0004397;GO:0050480;GO:0046872;GO:0016841;GO:0050480;GO:0005506;GO:0008270	BP:histidine catabolic process to glutamate and formamide;BP:histidine catabolic process to glutamate and formate;BP:histidine catabolic process to glutamate and formamide;BP:histidine catabolic process to glutamate and formate;BP:histidine catabolic process to glutamate and formamide;BP:histidine catabolic process to glutamate and formate;CC:cytoplasm;CC:cytoplasm;MF:lyase activity;MF:imidazolonepropionase activity;MF:iron ion binding;MF:metal ion binding;MF:zinc ion binding;MF:histidine ammonia-lyase activity;MF:imidazolonepropionase activity;MF:metal ion binding;MF:ammonia-lyase activity;MF:imidazolonepropionase activity;MF:iron ion binding;MF:zinc ion binding;
BAB_RS27075	-	flagellar basal-body rod protein FlgG	2.301	1.202490289	0.002912565	0.017297728	yes	up	mRNA	1.28	2.05	1.34	3.68	4.08	2.47	1.55667	3.41	COG4786	N:Cell motility	GO:0071973;GO:0009426	BP:bacterial-type flagellum-dependent cell motility;CC:bacterial-type flagellum basal body, distal rod;
BAB_RS26930	-	flagellar motor switch protein FliG	2.213	1.145763694	0.026556411	0.078137134	no	up	mRNA	0.95	0.67	1.38	1.71	2.56	2.23	1	2.1666667	COG1536	N:Cell motility	GO:0071973;GO:0006935;GO:0009288;GO:0003774	BP:bacterial-type flagellum-dependent cell motility;BP:chemotaxis;CC:bacterial-type flagellum;MF:motor activity;
BAB_RS24005	-	hypothetical protein	2.152	1.105658537	8.44038E-05	0.001674009	yes	up	mRNA	45.49	53.85	38.84	78	96.69	116.18	46.06	96.956667	ENOG411282K	S:Function unknown	GO:0016021	CC:integral component of membrane;
BAB_RS30680	-	nitrous-oxide reductase	2.105	1.074033969	0.001211015	0.009536744	yes	up	mRNA	2.97	3.85	2.36	5.39	5.79	7.2	3.06	6.1266667	COG1277	S:Function unknown	GO:0055085;GO:0016021	BP:transmembrane transport;CC:integral component of membrane;
BAB_RS33230	-	nickel ABC transporter	2.086	1.060706701	2.80055E-08	4.09007E-06	yes	up	mRNA	87.63	68.49	80.69	153.17	157.64	154.39	78.9367	155.06667	COG0747	K:Transcription	------	------
BAB_RS30805	-	hypothetical protein	2.036	1.025874416	0.002123322	0.01381019	yes	up	mRNA	261.16	445.15	302.24	433.5	917.72	679.79	336.183	677.00333	COG4309	S:Function unknown	------	------
BAB_RS27100	-	flagellar basal body L-ring protein FlgH	1.984	0.988139208	0.272096896	0.427713957	no	up	mRNA	0.21	0	0.68	0.89	0.32	0.68	0.29667	0.63	COG2063	N:Cell motility	GO:0071973;GO:0071973;GO:0009279;GO:0009427;GO:0009427;GO:0009279;GO:0003774;GO:0003774	BP:bacterial-type flagellum-dependent cell motility;BP:bacterial-type flagellum-dependent cell motility;CC:cell outer membrane;CC:bacterial-type flagellum basal body, distal rod, L ring;CC:bacterial-type flagellum basal body, distal rod, L ring;CC:cell outer membrane;MF:motor activity;MF:motor activity;
BAB_RS18550	-	IS5/IS1182 family transposase	1.978	0.984102546	0.121085005	0.237367982	no	up	mRNA	61.91	27.19	19.35	13.55	109.54	67.05	36.15	63.38	COG3293	L:Replication, recombination and repair	------	------
BAB_RS29080	-	hypothetical protein	1.972	0.979836327	0.00015833	0.00253092	no	up	mRNA	572.82	508.03	826.57	1259.77	1279.94	1186.73	635.807	1242.1467	------	------	------	------
BAB_RS30845	-	hemerythrin domain-containing protein	1.97	0.978449163	0.003190562	0.018504109	no	up	mRNA	123.59	193.33	145.58	181.93	408.87	280.31	154.167	290.37	ENOG410XXHV	S:Function unknown	------	------
BAB_RS30670	-	FAD:protein FMN transferase	1.958	0.969628747	0.00142115	0.010284131	no	up	mRNA	4.22	3.46	2.5	5.75	5.42	7.34	3.39333	6.17	COG1477	C:Energy production and conversion	GO:0017013;GO:0017013;GO:0005886;GO:0005886;GO:0046872;GO:0016740;GO:0016740;GO:0046872	BP:protein flavinylation;BP:protein flavinylation;CC:plasma membrane;CC:plasma membrane;MF:metal ion binding;MF:transferase activity;MF:transferase activity;MF:metal ion binding;
BAB_RS27915	-	SIS domain-containing protein	1.956	0.968216962	0.000709957	0.006554858	no	up	mRNA	130.49	126.9	149.11	353.69	221.49	174.85	135.5	250.01	COG2222	M:Cell wall/membrane/envelope biogenesis	GO:1901135;GO:1901135;GO:1901135;GO:0097367;GO:0008483;GO:0016853;GO:0004342;GO:0097367;GO:0097367;GO:0016853	BP:carbohydrate derivative metabolic process;BP:carbohydrate derivative metabolic process;BP:carbohydrate derivative metabolic process;MF:carbohydrate derivative binding;MF:transaminase activity;MF:isomerase activity;MF:glucosamine-6-phosphate deaminase activity;MF:carbohydrate derivative binding;MF:carbohydrate derivative binding;MF:isomerase activity;
BAB_RS27845	-	histidine utilization repressor	1.95	0.963427317	2.42099E-06	0.000121541	no	up	mRNA	27.51	25.74	26.65	47.29	56.18	43.21	26.6333	48.893333	COG2188	Q:Secondary metabolites biosynthesis, transport and catabolism	GO:0006547;GO:0045892;GO:0006351;GO:0006547;GO:0045892;GO:0006351;GO:0003677;GO:0003700;GO:0003700;GO:0003677	BP:histidine metabolic process;BP:negative regulation of transcription, DNA-templated;BP:transcription, DNA-templated;BP:histidine metabolic process;BP:negative regulation of transcription, DNA-templated;BP:transcription, DNA-templated;MF:DNA binding;MF:transcription factor activity, sequence-specific DNA binding;MF:transcription factor activity, sequence-specific DNA binding;MF:DNA binding;
BAB_RS30685	-	ABC transporter ATP-binding protein	1.946	0.96034119	0.003304401	0.018857978	no	up	mRNA	6.14	8.65	4.78	9.68	10.87	15.75	6.52333	12.1	COG1131	S:Function unknown	------	------
BAB_RS22610	-	ketol-acid reductoisomerase	1.941	0.956656272	2.3457E-07	1.93249E-05	no	up	mRNA	180.47	155.81	158.81	330.48	275.71	299.94	165.03	302.04333	COG0059	E:Amino acid transport and metabolism	GO:0009099;GO:0009097;GO:0050661;GO:0016853;GO:0000287;GO:0004455;GO:0046872	BP:valine biosynthetic process;BP:isoleucine biosynthetic process;MF:NADP binding;MF:isomerase activity;MF:magnesium ion binding;MF:ketol-acid reductoisomerase activity;MF:metal ion binding;
BAB_RS27920	-	N-acetylglucosamine-6-phosphate deacetylase	1.924	0.944420335	0.000712906	0.00654448	no	up	mRNA	82.42	84.65	95.18	222.05	141.99	112.16	87.4167	158.73333	COG1820	G:Carbohydrate transport and metabolism	------	------
BAB_RS27080	-	flagella basal body P-ring formation protein FlgA	1.904	0.928913012	0.14925896	0.278011037	no	up	mRNA	1.71	0.3	2.35	3.31	1.51	3.55	1.45333	2.79	COG1261	N:Cell motility	GO:0044781;GO:0042597	BP:bacterial-type flagellum organization;CC:periplasmic space;
BAB_RS33300	-	hypothetical protein	1.898	0.92454489	0.165940319	0.30003728	no	up	mRNA	26.64	35.01	47.56	75.55	62.18	115.12	36.4033	84.283333	------	------	GO:0008152;GO:0008483;GO:0008483	BP:metabolic process;MF:transaminase activity;MF:transaminase activity;
BAB_RS28790	-	DUF1127 domain-containing protein	1.892	0.920085104	1.55646E-05	0.000537731	no	up	mRNA	8055.2	7330.84	7847.05	16953.9	13677.6	12840.49	7744.36	14490.663	ENOG410Y8SB;COG5457	S:Function unknown;	------	------
BAB_RS25545	-	heavy metal translocating P-type ATPase	1.883	0.913338732	4.66537E-06	0.000214141	no	up	mRNA	12.87	12.55	14.12	26.71	23.66	19.68	13.18	23.35	COG2217	S:Function unknown	GO:0030001;GO:0030001;GO:0030001;GO:0016021;GO:0005886;GO:0016021;GO:0005886;GO:0016021;GO:0005886;GO:0019829;GO:0005507;GO:0000166;GO:0019829;GO:0005507;GO:0047381;GO:0000166;GO:0019829;GO:0000166;GO:0005507	BP:metal ion transport;BP:metal ion transport;BP:metal ion transport;CC:integral component of membrane;CC:plasma membrane;CC:integral component of membrane;CC:plasma membrane;CC:integral component of membrane;CC:plasma membrane;MF:cation-transporting ATPase activity;MF:copper ion binding;MF:nucleotide binding;MF:cation-transporting ATPase activity;MF:copper ion binding;MF:dodecanoyl-[acyl-carrier-protein] hydrolase activity;MF:nucleotide binding;MF:cation-transporting ATPase activity;MF:nucleotide binding;MF:copper ion binding;
BAB_RS30825	-	cytochrome c	1.879	0.91003448	0.003632305	0.020018176	no	up	mRNA	174.93	285.71	197.12	271.41	545.8	371.67	219.253	396.29333	COG2010	P:Inorganic ion transport and metabolism	GO:0016021;GO:0016021;GO:0016021;GO:0046872;GO:0016966;GO:0020037;GO:0009055;GO:0009055;GO:0020037;GO:0046872;GO:0046872;GO:0020037;GO:0009055	CC:integral component of membrane;CC:integral component of membrane;CC:integral component of membrane;MF:metal ion binding;MF:nitric oxide reductase activity;MF:heme binding;MF:electron carrier activity;MF:electron carrier activity;MF:heme binding;MF:metal ion binding;MF:metal ion binding;MF:heme binding;MF:electron carrier activity;
BAB_RS20755	-	acetylmuramidase	1.851	0.88839283	0.032430857	0.090138706	no	up	mRNA	1.19	0.85	2.17	2.89	2.13	2.69	1.40333	2.57	COG3926	S:Function unknown	GO:0016021	CC:integral component of membrane;
BAB_RS30690	-	nitrous oxide reductase family maturation protein NosD	1.849	0.886836955	0.024616926	0.074617155	no	up	mRNA	7.14	12.41	7.26	8.41	16.02	23.16	8.93667	15.863333	COG3420	P:Inorganic ion transport and metabolism	------	------
BAB_RS27435	-	hypothetical protein	1.835	0.875449624	5.45153E-06	0.000239942	no	up	mRNA	222.1	185.73	167.85	363.16	341.2	296.79	191.893	333.71667	COG5591	T:Signal transduction mechanisms	------	------
BAB_RS27130	-	CoA transferase	1.833	0.874031448	1.79176E-07	1.64484E-05	no	up	mRNA	240.29	229.17	273.72	383.18	437.63	462.54	247.727	427.78333	COG1804	K:Transcription	GO:0008152;GO:0008152;GO:0008111;GO:0033608;GO:0033608	BP:metabolic process;BP:metabolic process;MF:alpha-methylacyl-CoA racemase activity;MF:formyl-CoA transferase activity;MF:formyl-CoA transferase activity;
BAB_RS27440	-	DUF2271 domain-containing protein	1.828	0.870466096	4.55136E-05	0.001151459	no	up	mRNA	30.73	35.33	33.78	64.89	60.41	49.19	33.28	58.163333	ENOG4111NWN	S:Function unknown	------	------
BAB_RS33235	-	nickel ABC transporter	1.798	0.846169919	1.74502E-06	9.66679E-05	no	up	mRNA	32.96	36.53	32.8	56.96	59.14	58.4	34.0967	58.166667	COG0747	E:Amino acid transport and metabolism	GO:0015675;GO:0055085;GO:0043190;GO:0020037;GO:0016151	BP:nickel cation transport;BP:transmembrane transport;CC:ATP-binding cassett;MF:heme binding;MF:nickel cation binding;
BAB_RS33530	-	D-lactate dehydrogenase	1.793	0.842307788	0.052440642	0.128914905	no	up	mRNA	3.09	3.79	2.66	5.88	5.78	4.89	3.18	5.5166667	COG0277	S:Function unknown	GO:0019516;GO:0022904;GO:0055085;GO:0031234;GO:0005887;GO:0050660;GO:0016901;GO:0048038	BP:lactate oxidation;BP:respiratory electron transport chain;BP:transmembrane transport;CC:extrinsic component of cytoplasmic side of plasma membrane;CC:integral component of plasma membrane;MF:flavin adenine dinucleotide binding;MF:oxidoreductase activity, acting on the CH-OH group of donors, quinone or similar compound as acceptor;MF:quinone binding;
BAB_RS20410	-	IS5/IS1182 family transposase	1.785	0.83627074	0.110113488	0.221398397	no	up	mRNA	8.09	2.59	2.55	6.07	4.31	10.67	4.41	7.0166667	COG3293	L:Replication, recombination and repair	GO:0006313;GO:0016021;GO:0003677;GO:0004803	BP:transposition, DNA-mediated;CC:integral component of membrane;MF:DNA binding;MF:transposase activity;
BAB_RS27980	-	ornithine cyclodeaminase	1.775	0.827464419	3.67266E-08	4.91677E-06	no	up	mRNA	197.95	194.6	212.48	351.33	314.61	345.12	201.677	337.02	COG2423	E:Amino acid transport and metabolism	GO:0006525;GO:0055129;GO:0008473;GO:0008473	BP:arginine metabolic process;BP:L-proline biosynthetic process;MF:ornithine cyclodeaminase activity;MF:ornithine cyclodeaminase activity;
BAB_RS19275	-	EamA family transporter RarD	1.758	0.81372075	6.69816E-05	0.001434745	no	up	mRNA	105.76	89.75	105.44	206.62	148.4	143.55	100.317	166.19	COG2962	S:Function unknown	GO:0005887	CC:integral component of plasma membrane;
BAB_RS28625	-	DUF1127 domain-containing protein	1.755	0.811344721	1.0542E-05	0.000389326	no	up	mRNA	195.93	152.14	147.17	302.27	250.6	277.58	165.08	276.81667	------	------	------	------
BAB_RS27430	-	DNA-binding response regulator	1.755	0.811243128	0.003249979	0.018680113	no	up	mRNA	39.85	39.04	41.93	41.54	87.64	71.32	40.2733	66.833333	ENOG4111FPD	T:Signal transduction mechanisms	GO:0000160;GO:0006355;GO:0006351;GO:0000160;GO:0006351;GO:0006355;GO:0005622;GO:0005622;GO:0003677;GO:0003677	BP:phosphorelay signal transduction system;BP:regulation of transcription, DNA-templated;BP:transcription, DNA-templated;BP:phosphorelay signal transduction system;BP:transcription, DNA-templated;BP:regulation of transcription, DNA-templated;CC:intracellular;CC:intracellular;MF:DNA binding;MF:DNA binding;
BAB_RS26455	-	cytochrome b/b6 domain-containing protein	1.742	0.801018833	0.007379947	0.032260912	no	up	mRNA	2.15	3.77	4.81	5.72	6.76	5.87	3.57667	6.1166667	COG4117	C:Energy production and conversion	GO:0022904;GO:0022904;GO:0016021;GO:0016021;GO:0009055;GO:0009055	BP:respiratory electron transport chain;BP:respiratory electron transport chain;CC:integral component of membrane;CC:integral component of membrane;MF:electron carrier activity;MF:electron carrier activity;
BAB_RS28265	-	DUF3892 domain-containing protein	1.733	0.793073018	0.000839174	0.007267563	no	up	mRNA	523.26	445.37	505.4	1080.27	732.15	598.35	491.343	803.59	ENOG410XYZU;ENOG4112762	S:Function unknown;	------	------
BAB_RS28080	-	magnesium transporter	1.73	0.791088877	0.001698134	0.011708379	no	up	mRNA	13.59	12.12	12.67	28.17	18.18	16.05	12.7933	20.8	COG2239	P:Inorganic ion transport and metabolism	GO:0016021;GO:0005886;GO:0016021;GO:0005886;GO:0046872;GO:0015095;GO:0015095;GO:0046872	CC:integral component of membrane;CC:plasma membrane;CC:integral component of membrane;CC:plasma membrane;MF:metal ion binding;MF:magnesium ion transmembrane transporter activity;MF:magnesium ion transmembrane transporter activity;MF:metal ion binding;
BAB_RS20800	-	beta-(1-->2)glucan export ATP-binding/permease NdvA	1.718	0.780797405	2.58366E-07	2.02471E-05	no	up	mRNA	53.21	47.24	51.44	88.17	78.01	79.18	50.63	81.786667	COG1132	S:Function unknown	GO:0016021;GO:0005886;GO:0015441;GO:0043621;GO:0005524	CC:integral component of membrane;CC:plasma membrane;MF:beta-glucan-transporting ATPase activity;MF:protein self-association;MF:ATP binding;
BAB_RS22605	-	hypothetical protein	1.712	0.775914013	0.001978079	0.013023708	no	up	mRNA	58.48	34.27	48.55	91.96	65.6	74.03	47.1	77.196667	------	------	GO:0016021	CC:integral component of membrane;
BAB_RS30810	-	protein norD	1.697	0.762665172	0.013803113	0.048842955	no	up	mRNA	19.86	32.58	19.03	25.15	47.1	43.32	23.8233	38.523333	COG4548	P:Inorganic ion transport and metabolism	GO:0009405	BP:pathogenesis;
BAB_RS27975	-	arginase	1.691	0.757751558	1.77671E-07	1.67899E-05	no	up	mRNA	337.51	332.61	337.1	552.72	496.19	561.72	335.74	536.87667	COG0010	K:Transcription	GO:0006525;GO:0000050;GO:0006525;GO:0000050;GO:0006525;GO:0004053;GO:0046872;GO:0004053;GO:0046872;GO:0004053;GO:0046872;GO:0004053;GO:0004177	BP:arginine metabolic process;BP:urea cycle;BP:arginine metabolic process;BP:urea cycle;BP:arginine metabolic process;MF:arginase activity;MF:metal ion binding;MF:arginase activity;MF:metal ion binding;MF:arginase activity;MF:metal ion binding;MF:arginase activity;MF:aminopeptidase activity;
BAB_RS28250	-	SAM-dependent DNA methyltransferase	1.69	0.756718802	0.000354268	0.004154241	no	up	mRNA	180.76	161.67	182.65	355.89	264.89	213.71	175.027	278.16333	COG0286	V:Defense mechanisms	GO:0006306;GO:0006306;GO:0006306;GO:0003677;GO:0008170;GO:0003677;GO:0050104;GO:0008170;GO:0004519;GO:0009007;GO:0008170;GO:0003677;GO:0050104	BP:DNA methylation;BP:DNA methylation;BP:DNA methylation;MF:DNA binding;MF:N-methyltransferase activity;MF:DNA binding;MF:L-gulonate 3-dehydrogenase activity;MF:N-methyltransferase activity;MF:endonuclease activity;MF:site-specific DNA-methyltransferase (adenine-specific) activity;MF:N-methyltransferase activity;MF:DNA binding;MF:L-gulonate 3-dehydrogenase activity;
BAB_RS27925	-	sugar isomerase domain-containing protein	1.687	0.754552319	0.002583491	0.015871429	no	up	mRNA	51.02	48.27	48.91	108.6	68.11	59.77	49.4	78.826667	COG4821	S:Function unknown	GO:1901135;GO:0097367	BP:carbohydrate derivative metabolic process;MF:carbohydrate derivative binding;
BAB_RS20300	-	DUF1127 domain-containing protein	1.684	0.752279124	1.09722E-06	7.34449E-05	no	up	mRNA	13141	12230.1	10416.6	21456.41	19695.35	18159.76	11929.2	19770.507	COG5457	S:Function unknown	------	------
BAB_RS26940	-	flagellar motor switch protein FliN	1.683	0.750669448	0.501408407	0.656757118	no	up	mRNA	4.81	1.35	0	0	4.78	3.03	2.05333	2.6033333	COG1886	N:Cell motility	GO:0006935;GO:0071973;GO:0071973;GO:0006935;GO:0006935;GO:0071973;GO:0009425;GO:0005886;GO:0009425;GO:0005886;GO:0009425;GO:0003774;GO:0003774;GO:0003774	BP:chemotaxis;BP:bacterial-type flagellum-dependent cell motility;BP:bacterial-type flagellum-dependent cell motility;BP:chemotaxis;BP:chemotaxis;BP:bacterial-type flagellum-dependent cell motility;CC:bacterial-type flagellum basal body;CC:plasma membrane;CC:bacterial-type flagellum basal body;CC:plasma membrane;CC:bacterial-type flagellum basal body;MF:motor activity;MF:motor activity;MF:motor activity;
BAB_RS30695	-	TAT-dependent nitrous-oxide reductase	1.676	0.745163254	0.032558831	0.090104672	no	up	mRNA	43.1	86.57	41.81	60.27	101.39	113.9	57.16	91.853333	COG4263	C:Energy production and conversion	GO:0019333;GO:0016020;GO:0042597;GO:0016020;GO:0042597;GO:0005509;GO:0005507;GO:0004129;GO:0050304;GO:0004129;GO:0050304;GO:0005509;GO:0005507	BP:denitrification pathway;CC:membrane;CC:periplasmic space;CC:membrane;CC:periplasmic space;MF:calcium ion binding;MF:copper ion binding;MF:cytochrome-c oxidase activity;MF:nitrous-oxide reductase activity;MF:cytochrome-c oxidase activity;MF:nitrous-oxide reductase activity;MF:calcium ion binding;MF:copper ion binding;
BAB_RS27485	-	TonB-dependent copper receptor	1.65	0.722062558	2.24766E-05	0.000687785	no	up	mRNA	24.97	29.18	24.59	39.25	39.72	43.93	26.2467	40.966667	COG1629	P:Inorganic ion transport and metabolism	GO:0016021;GO:0009279;GO:0009279;GO:0016021	CC:integral component of membrane;CC:cell outer membrane;CC:cell outer membrane;CC:integral component of membrane;
BAB_RS28345	-	camphor resistance protein CrcB	1.642	0.715231649	0.025769934	0.076665554	no	up	mRNA	4.4	6.98	5.92	10.77	8.58	8.76	5.76667	9.37	COG0239	S:Function unknown	GO:0016021;GO:0005887;GO:0005886;GO:0005887;GO:0015103;GO:0015103	CC:integral component of membrane;CC:integral component of plasma membrane;CC:plasma membrane;CC:integral component of plasma membrane;MF:inorganic anion transmembrane transporter activity;MF:inorganic anion transmembrane transporter activity;
BAB_RS29085	-	DUF2938 domain-containing protein	1.634	0.70884814	8.89344E-05	0.001753045	no	up	mRNA	168.46	139.53	169.36	295.77	227.06	213.29	159.117	245.37333	ENOG4111NNN	S:Function unknown	GO:0016021	CC:integral component of membrane;
BAB_RS29075	-	DUF1127 domain-containing protein	1.628	0.703473609	0.001053822	0.008593735	no	up	mRNA	2193.43	2088.4	3402.14	4361.11	3850.03	3747.95	2561.32	3986.3633	COG5457	S:Function unknown	------	------
BAB_RS23030	-	hypothetical protein	1.627	0.701813719	7.88443E-05	0.001613547	no	up	mRNA	93.63	73.81	83.63	143.74	134.3	105.59	83.69	127.87667	ENOG4112D4Y	S:Function unknown	GO:0042597	CC:periplasmic space;
BAB_RS28440	-	nickel import ATP-binding protein NikD	1.618	0.69411867	0.013041459	0.047293687	no	up	mRNA	3.39	5.89	5.44	8.87	6.33	8.11	4.90667	7.77	COG0444	E:Amino acid transport and metabolism	GO:0005886;GO:0016151;GO:0015413;GO:0005524	CC:plasma membrane;MF:nickel cation binding;MF:nickel-transporting ATPase activity;MF:ATP binding;
BAB_RS28245	-	restriction endonuclease subunit S	1.61	0.687036438	0.00019751	0.00287149	no	up	mRNA	126.87	103.64	115.18	213.95	159.76	149.75	115.23	174.48667	COG0732	V:Defense mechanisms	GO:0006304;GO:0006304;GO:0003677;GO:0009035;GO:0003677	BP:DNA modification;BP:DNA modification;MF:DNA binding;MF:Type I site-specific deoxyribonuclease activity;MF:DNA binding;
BAB_RS33205	-	hypothetical protein	1.608	0.685568036	2.03146E-05	0.00063991	no	up	mRNA	37.56	35.75	39.45	55.44	60.93	56.42	37.5867	57.596667	COG0582	S:Function unknown	------	------
BAB_RS29070	-	EamA/RhaT family transporter	1.607	0.684561413	0.001360685	0.010096722	no	up	mRNA	12.24	8.02	10.08	17.68	14.77	12.88	10.1133	15.11	ENOG410XTFY	S:Function unknown	GO:0016021	CC:integral component of membrane;
BAB_RS33540	-	hypothetical protein	1.607	0.684655347	0.363809397	0.529881955	no	up	mRNA	0	7.75	6.56	13.95	6.82	6.84	4.77	9.2033333	------	------	------	------
BAB_RS17930	-	hypothetical protein	1.604	0.681541381	0.051120388	0.12644327	no	up	mRNA	106.08	65.63	81.24	119.63	110.11	189.79	84.3167	139.84333	------	------	------	------
BAB_RS33125	-	ABC transporter substrate-binding protein	1.595	0.673172068	0.004217509	0.022214519	no	up	mRNA	32.46	22.13	26.81	52.79	37.48	30.84	27.1333	40.37	COG1879;ENOG410ZAHE	S:Function unknown;G:Carbohydrate transport and metabolism	GO:0008643	BP:carbohydrate transport;
BAB_RS26595	-	hypothetical protein	1.589	0.667702954	1.90932E-06	9.89457E-05	no	up	mRNA	102.21	96.62	107.95	144.15	160.96	159.16	102.26	154.75667	------	------	------	------
BAB_RS28925	-	iron ABC transporter substrate-binding protein	1.581	0.660674173	3.54682E-05	0.00095764	no	up	mRNA	156.1	172.55	153.35	268.22	236.55	216.7	160.667	240.49	COG1840	S:Function unknown	GO:0046872	MF:metal ion binding;
BAB_RS18890	-	tail protein	1.572	0.65303506	0.110361391	0.221619468	no	up	mRNA	1.35	1.68	1.9	2.03	2.75	2.82	1.64333	2.5333333	COG5281	S:Function unknown	------	------
BAB_RS28085	-	translational GTPase TypA	1.57	0.650463133	7.45061E-06	0.000295541	no	up	mRNA	469.19	407.03	432.87	716.17	592.83	625.05	436.363	644.68333	COG1217	T:Signal transduction mechanisms	GO:0003746;GO:0003924;GO:0005525;GO:0003924;GO:0005525;GO:0005525;GO:0003924	MF:translation elongation factor activity;MF:GTPase activity;MF:GTP binding;MF:GTPase activity;MF:GTP binding;MF:GTP binding;MF:GTPase activity;
BAB_RS23105	-	carbamoyl-phosphate synthase large subunit	1.566	0.646813296	6.64127E-06	0.000270106	no	up	mRNA	108.75	97.06	103.42	169.11	148.69	137.34	103.077	151.71333	COG0458	F:Nucleotide transport and metabolism	GO:0044205;GO:0006526;GO:0044205;GO:0006526;GO:0004088;GO:0046872;GO:0005524;GO:0016491;GO:0004088;GO:0046872;GO:0005524	BP:'de novo' UMP biosynthetic process;BP:arginine biosynthetic process;BP:'de novo' UMP biosynthetic process;BP:arginine biosynthetic process;MF:carbamoyl-phosphate synthas;MF:metal ion binding;MF:ATP binding;MF:oxidoreductase activity;MF:carbamoyl-phosphate synthas;MF:metal ion binding;MF:ATP binding;
BAB_RS18650	-	xylulokinase	1.555	0.637314364	0.001068444	0.008690912	no	up	mRNA	10.7	12.82	12.81	21.77	16.1	15.78	12.11	17.883333	COG1070	G:Carbohydrate transport and metabolism	GO:0042732;GO:0005997;GO:0004856;GO:0005524;GO:0016491	BP:D-xylose metabolic process;BP:xylulose metabolic process;MF:xylulokinase activity;MF:ATP binding;MF:oxidoreductase activity;
BAB_RS32000	-	hypothetical protein	1.545	0.627664954	0.060693885	0.142864067	no	up	mRNA	3.43	7.61	8.3	8.46	11.12	10.63	6.44667	10.07	------	------	------	------
BAB_RS22890	-	lytic transglycosylase	1.537	0.620085102	0.00031492	0.003861981	no	up	mRNA	158.52	117.98	143.26	236.38	171.98	198.96	139.92	202.44	COG0741	S:Function unknown	------	------
BAB_RS27405	-	CoA transferase	1.532	0.61585528	3.0681E-06	0.000151658	no	up	mRNA	32.34	30.18	31.37	42.76	47.65	45.02	31.2967	45.143333	COG1804	G:Carbohydrate transport and metabolism	GO:0008152;GO:0008152;GO:0033608;GO:0033608	BP:metabolic process;BP:metabolic process;MF:formyl-CoA transferase activity;MF:formyl-CoA transferase activity;
BAB_RS27650	-	hypothetical protein	1.521	0.604939672	0.040162859	0.106033909	no	up	mRNA	13	16.24	20.67	24.55	25.59	28.38	16.6367	26.173333	ENOG410Y07C	S:Function unknown	------	------
BAB_RS29645	-	DUF995 domain-containing protein	1.521	0.604801278	0.106629918	0.216425727	no	up	mRNA	4.49	1.82	3.21	5.2	4.72	3.29	3.17333	4.4033333	ENOG410YKTA;ENOG410XSE	S:Function unknown;	------	------
BAB_RS17360	-	urease subunit alpha 1	1.517	0.600875419	1.90232E-06	0.000100199	no	up	mRNA	35.72	37.14	41.3	52.94	54.71	55.72	38.0533	54.456667	COG0804	E:Amino acid transport and metabolism	GO:0009405;GO:0043419;GO:0005737;GO:0009039;GO:0016151	BP:pathogenesis;BP:urea catabolic process;CC:cytoplasm;MF:urease activity;MF:nickel cation binding;
BAB_RS30675	-	hypothetical protein	1.514	0.598452256	0.032016912	0.089374751	no	up	mRNA	8.99	7.55	7.48	9.79	9.29	15.59	8.00667	11.556667	COG4314	C:Energy production and conversion	GO:0016853	MF:isomerase activity;
BAB_RS20935	-	thioredoxin reductase	1.486	0.571101906	0.215406	0.361787496	no	up	mRNA	8.65	10.37	14.66	10.8	25.11	17.14	11.2267	17.683333	COG0492	O:Posttranslational modification, protein turnover, chaperones	GO:0016021	CC:integral component of membrane;
BAB_RS27400	-	3-hydroxyacyl-CoA dehydrogenase	1.485	0.570163183	4.95355E-05	0.001214943	no	up	mRNA	29.12	27.79	28.04	42.57	41.53	34.5	28.3167	39.533333	COG1250	G:Carbohydrate transport and metabolism	GO:0006635;GO:0006631;GO:0003857;GO:0003857;GO:0008691	BP:fatty acid beta-oxidation;BP:fatty acid metabolic process;MF:3-hydroxyacyl-CoA dehydrogenase activity;MF:3-hydroxyacyl-CoA dehydrogenase activity;MF:3-hydroxybutyryl-CoA dehydrogenase activity;
BAB_RS24745	-	N-acetyltransferase	1.479	0.564561815	0.000369335	0.004284014	no	up	mRNA	18.18	20.95	22.8	30.36	29.47	27.51	20.6433	29.113333	COG1670	S:Function unknown	GO:0016740	MF:transferase activity;
BAB_RS32180	-	hypothetical protein	1.47	0.555351099	0.274945752	0.430927171	no	up	mRNA	86.71	75.9	51.39	95.18	134.95	77.81	71.3333	102.64667	COG4631	F:Nucleotide transport and metabolism	------	------
BAB_RS30450	-	hydrophobe/amphiphile efflux-1 family RND transporter	1.462	0.548381503	3.22178E-07	2.46466E-05	no	up	mRNA	64.52	63.15	69.71	91.11	91.9	88.47	65.7933	90.493333	COG0841	S:Function unknown	GO:0016021;GO:0022857	CC:integral component of membrane;MF:transmembrane transporter activity;
BAB_RS30440	-	MexE family multidrug efflux RND transporter periplasmic adaptor subunit	1.455	0.540976236	3.39335E-07	2.47792E-05	no	up	mRNA	229.15	219.94	241.28	312.04	310.6	324.06	230.123	315.56667	ENOG410XNVN	S:Function unknown	GO:0055085;GO:0055085;GO:0016020;GO:0016020;GO:0005215;GO:0005215	BP:transmembrane transport;BP:transmembrane transport;CC:membrane;CC:membrane;MF:transporter activity;MF:transporter activity;
BAB_RS27445	-	oxidoreductase	1.451	0.537078686	0.043203784	0.111676395	no	up	mRNA	22.36	17.34	21.57	33.1	29.77	24.6	20.4233	29.156667	COG0369	P:Inorganic ion transport and metabolism	GO:0016021	CC:integral component of membrane;
BAB_RS29450	-	bifunctional lysylphosphatidylglycerol flippase/synthetase MprF	1.45	0.535953323	1.45053E-06	8.63065E-05	no	up	mRNA	50.66	44.95	50.57	68.54	65.3	65.33	48.7267	66.39	COG0392	U:Intracellular trafficking, secretion, and vesicular transport	GO:0016021	CC:integral component of membrane;
BAB_RS27515	-	ABC transporter permease	1.45	0.536098212	0.059895904	0.141921489	no	up	mRNA	1.72	2.75	2.14	3.48	3.07	2.68	2.20333	3.0766667	COG1172	G:Carbohydrate transport and metabolism	GO:0008643;GO:0016021;GO:0005886;GO:0016021;GO:0005886;GO:0022857;GO:0022857	BP:carbohydrate transport;CC:integral component of membrane;CC:plasma membrane;CC:integral component of membrane;CC:plasma membrane;MF:transmembrane transporter activity;MF:transmembrane transporter activity;
BAB_RS24135	-	membrane protein	1.449	0.534754632	1.73672E-06	9.78964E-05	no	up	mRNA	93.87	82.3	91.18	124.11	119.66	120.8	89.1167	121.52333	COG4645	S:Function unknown	GO:0016021;GO:0016021;GO:0016740	CC:integral component of membrane;CC:integral component of membrane;MF:transferase activity;
BAB_RS26650	-	type IV secretion system protein virB8	1.448	0.534490582	0.002546625	0.015826509	no	up	mRNA	17.43	15.61	19.94	24.05	21.16	27.97	17.66	24.393333	COG3736	U:Intracellular trafficking, secretion, and vesicular transport	GO:0030255;GO:0009405;GO:0030255;GO:0016021;GO:0005886;GO:0016021;GO:0042802	BP:protein secretion by the type IV secretion system;BP:pathogenesis;BP:protein secretion by the type IV secretion system;CC:integral component of membrane;CC:plasma membrane;CC:integral component of membrane;MF:identical protein binding;
BAB_RS26005	-	hypothetical protein	1.447	0.533392499	0.501140675	0.656674139	no	up	mRNA	0	6.35	8.67	9.54	2.25	15.17	5.00667	8.9866667	COG3135	Q:Secondary metabolites biosynthesis, transport and catabolism	GO:0016021;GO:0042925	CC:integral component of membrane;MF:benzoate transporter activity;
BAB_RS28435	-	nickel ABC transporter permease subunit NikC	1.444	0.530513498	0.012651642	0.046350885	no	up	mRNA	7.5	5.69	6.63	7.6	10.65	8.4	6.60667	8.8833333	COG1173	E:Amino acid transport and metabolism	GO:0008152;GO:0008152;GO:0016021;GO:0005886;GO:0016021;GO:0005886;GO:0016021;GO:0005886;GO:0015099;GO:0015099;GO:0015413;GO:0031219;GO:0015099	BP:metabolic process;BP:metabolic process;CC:integral component of membrane;CC:plasma membrane;CC:integral component of membrane;CC:plasma membrane;CC:integral component of membrane;CC:plasma membrane;MF:nickel cation transmembrane transporter activity;MF:nickel cation transmembrane transporter activity;MF:levanase activity;MF:nickel-transporting ATPase activity;MF:levanase activity;MF:nickel cation transmembrane transporter activity;
BAB_RS19260	-	hypothetical protein	1.443	0.529461552	0.00122979	0.00956735	no	up	mRNA	27293.4	28188.9	25956.2	36548.51	44495.62	38644.66	27146.2	39896.263	------	------	GO:0016787	MF:hydrolase activity;
BAB_RS28185	-	transporter	1.437	0.522641052	0.051095173	0.126478268	no	up	mRNA	9.1	6.11	8.32	11.67	8.74	11.93	7.84333	10.78	COG0697	S:Function unknown	GO:0016021	CC:integral component of membrane;
BAB_RS26715	-	SPFH/Band 7/PHB domain protein	1.434	0.519680209	1.3993E-06	8.64607E-05	no	up	mRNA	171.34	169.83	190.89	241.97	237.9	240.64	177.353	240.17	COG0330;COG0330	O:Posttranslational modification, protein turnover, chaperones;U:Intracellular trafficking, secretion, and vesicular transport	GO:0016020	CC:membrane;
BAB_RS22615	-	TetR/AcrR family transcriptional regulator	1.43	0.516007613	1.70789E-05	0.000565717	no	up	mRNA	76.5	70.63	75.22	104.47	94.48	102.25	74.1167	100.4	COG1309	E:Amino acid transport and metabolism	GO:0006355;GO:0006351;GO:0003677	BP:regulation of transcription, DNA-templated;BP:transcription, DNA-templated;MF:DNA binding;
BAB_RS27950	-	FAD-binding oxidoreductase	1.425	0.511233146	2.16205E-06	0.000110265	no	up	mRNA	221.13	192.82	209.86	272.67	289.27	275.07	207.937	279.00333	COG0665	S:Function unknown	GO:0008115;GO:0016491;GO:0016491	MF:sarcosine oxidase activity;MF:oxidoreductase activity;MF:oxidoreductase activity;
BAB_RS29550	-	30S ribosomal protein S12 methylthiotransferase RimO	1.424	0.509695068	0.000997533	0.008281841	no	up	mRNA	35.95	26.14	35.04	49.53	39.18	40.86	32.3767	43.19	COG0621	C:Energy production and conversion	GO:0018339;GO:0006400;GO:0018339;GO:0006400;GO:0018339;GO:0006400;GO:0005737;GO:0005840;GO:0103039;GO:0005840;GO:0005737;GO:0046872;GO:0103039;GO:0051539;GO:0046872;GO:0016491;GO:0051539;GO:0046872;GO:0103039	BP:peptidyl-L-beta-methylthioaspartic acid biosynthetic process from peptidyl-aspartic acid;BP:tRNA modification;BP:peptidyl-L-beta-methylthioaspartic acid biosynthetic process from peptidyl-aspartic acid;BP:tRNA modification;BP:peptidyl-L-beta-methylthioaspartic acid biosynthetic process from peptidyl-aspartic acid;BP:tRNA modification;CC:cytoplasm;CC:ribosome;CC:cytoplasm;CC:ribosome;CC:old GO;CC:ribosome;MF:4 iron, 4 sulfur cluster binding;MF:metal ion binding;MF:old GO;MF:4 iron, 4 sulfur cluster binding;MF:metal ion binding;MF:oxidoreductase activity;MF:4 iron, 4 sulfur cluster binding;MF:metal ion binding;MF:old GO;
BAB_RS28430	-	nickel ABC transporter permease subunit NikB	1.424	0.509979062	0.004806339	0.024242963	no	up	mRNA	8.77	7.8	11.27	12.23	13.65	11.68	9.28	12.52	COG0601	E:Amino acid transport and metabolism	GO:0008152;GO:0016021;GO:0005886;GO:0016021;GO:0005886;GO:0015099;GO:0015413;GO:0031219;GO:0015099	BP:metabolic process;CC:integral component of membrane;CC:plasma membrane;CC:integral component of membrane;CC:plasma membrane;MF:nickel cation transmembrane transporter activity;MF:nickel-transporting ATPase activity;MF:levanase activity;MF:nickel cation transmembrane transporter activity;
BAB_RS16505	-	helicase	1.416	0.501323842	8.1323E-05	0.001643338	no	up	mRNA	310.98	270.29	292.65	437.49	364.88	361.46	291.307	387.94333	COG0513	L:Replication, recombination and repair	GO:0005524;GO:0003676;GO:0004386;GO:0005524;GO:0004386;GO:0003676	MF:ATP binding;MF:nucleic acid binding;MF:helicase activity;MF:ATP binding;MF:helicase activity;MF:nucleic acid binding;
BAB_RS32905	-	hypothetical protein	1.416	0.501418521	0.000649166	0.006244823	no	up	mRNA	49.11	41.97	64.48	68.76	73.62	63.71	51.8533	68.696667	------	------	------	------
BAB_RS31560	-	flagellum biosynthesis repressor protein FlbT	1.413	0.498477803	0.370707519	0.536523991	no	up	mRNA	1.9	3.68	3.17	7.06	1.68	3.94	2.91667	4.2266667	COG5443	N:Cell motility	GO:0044781;GO:0006402;GO:1902209;GO:0044781;GO:0006402;GO:1902209;GO:0048027;GO:0048027	BP:bacterial-type flagellum organization;BP:mRNA catabolic process;BP:negative regulation of bacterial-type flagellum assembly;BP:bacterial-type flagellum organization;BP:mRNA catabolic process;BP:negative regulation of bacterial-type flagellum assembly;MF:mRNA 5'-UTR binding;MF:mRNA 5'-UTR binding;
BAB_RS24600	-	bifunctional phosphoribosylaminoimidazolecarboxamide formyltransferase/inosine monophosphate cyclohydrolase	1.412	0.497316702	4.48273E-05	0.001143097	no	up	mRNA	110.75	97.44	111.1	156	128.08	139.92	106.43	141.33333	COG0138	F:Nucleotide transport and metabolism	GO:0006189;GO:0003937;GO:0004643	BP:'de novo' IMP biosynthetic process;MF:IMP cyclohydrolase activity;MF:phosphoribosylaminoimidazolecarboxamide formyltransferase activity;
BAB_RS16330	-	ABC transporter permease	1.409	0.495132009	2.97633E-05	0.000838856	no	up	mRNA	41.28	41.09	43.5	58.01	58.77	50.16	41.9567	55.646667	COG3127	Q:Secondary metabolites biosynthesis, transport and catabolism	GO:0016021;GO:0016740	CC:integral component of membrane;MF:transferase activity;
BAB_RS30870	-	EamA/RhaT family transporter	1.409	0.494551936	0.01551822	0.053383343	no	up	mRNA	10.23	8.48	12.45	16.84	11.76	13.08	10.3867	13.893333	COG0697	K:Transcription	GO:0016021	CC:integral component of membrane;
BAB_RS29970	-	HlyD family secretion protein	1.407	0.492475128	0.000233243	0.003188971	no	up	mRNA	24.32	21.92	30.22	34.69	32.2	34.54	25.4867	33.81	COG1566	S:Function unknown	GO:0055085;GO:0055085;GO:0016021;GO:0016021;GO:0016021;GO:0005215;GO:0005215	BP:transmembrane transport;BP:transmembrane transport;CC:integral component of membrane;CC:integral component of membrane;CC:integral component of membrane;MF:transporter activity;MF:transporter activity;
BAB_RS29560	-	(2Fe-2S)-binding protein	1.407	0.492468059	0.001932301	0.012800993	no	up	mRNA	159.88	152.08	121.27	170.46	223.61	185.24	144.41	193.10333	ENOG4111Z7T	S:Function unknown	------	------
BAB_RS17355	-	urease subunit beta	1.407	0.492787648	0.005526753	0.026864534	no	up	mRNA	22.72	24.84	26.42	33	33.73	34.39	24.66	33.706667	COG0832	E:Amino acid transport and metabolism	GO:0009405;GO:0043419;GO:0043419;GO:0005737;GO:0005737;GO:0009039;GO:0009039	BP:pathogenesis;BP:urea catabolic process;BP:urea catabolic process;CC:cytoplasm;CC:cytoplasm;MF:urease activity;MF:urease activity;
BAB_RS33160	-	hypothetical protein	1.404	0.489750708	9.22206E-05	0.001795786	no	up	mRNA	67.84	59.93	84.13	89.61	97.89	91.31	70.6333	92.936667	COG5383	S:Function unknown	------	------
BAB_RS16245	-	DNA polymerase Y family protein	1.403	0.488580058	0.005865154	0.027835659	no	up	mRNA	3.91	3.64	4.97	5.59	5.61	5.43	4.17333	5.5433333	COG0389	L:Replication, recombination and repair	GO:0006281	BP:DNA repair;
BAB_RS23130	-	pyridoxal phosphate-dependent aminotransferase	1.399	0.484626988	5.88479E-05	0.001322227	no	up	mRNA	188.59	158.88	162.81	237.48	210.05	224.93	170.093	224.15333	COG0436	S:Function unknown	GO:0009058;GO:0009058;GO:0030170;GO:0008483;GO:0008483;GO:0030170	BP:biosynthetic process;BP:biosynthetic process;MF:pyridoxal phosphate binding;MF:transaminase activity;MF:transaminase activity;MF:pyridoxal phosphate binding;
BAB_RS17000	-	sarcosine oxidase subunit delta family protein	1.395	0.480667055	0.362947786	0.529346907	no	up	mRNA	4.91	5.97	2.63	8.45	4.24	5.37	4.50333	6.02	COG4311	E:Amino acid transport and metabolism	GO:0046653;GO:0046653;GO:0008115;GO:0008115	BP:tetrahydrofolate metabolic process;BP:tetrahydrofolate metabolic process;MF:sarcosine oxidase activity;MF:sarcosine oxidase activity;
BAB_RS27865	-	alanine racemase	1.392	0.476690318	0.000201613	0.002917934	no	up	mRNA	18.51	20.41	20.52	25.91	25.1	27.38	19.8133	26.13	COG0787	M:Cell wall/membrane/envelope biogenesis	GO:0030632;GO:0030632;GO:0008784;GO:0030170;GO:0008784;GO:0030170	BP:D-alanine biosynthetic process;BP:D-alanine biosynthetic process;MF:alanine racemase activity;MF:pyridoxal phosphate binding;MF:alanine racemase activity;MF:pyridoxal phosphate binding;
BAB_RS19255	-	amino acid transporter LysE	1.392	0.477665592	0.000540472	0.005565819	no	up	mRNA	415.99	349.21	503.46	526.06	611.04	529.74	422.887	555.61333	COG1279	S:Function unknown	GO:0006865;GO:0016021;GO:0005886	BP:amino acid transport;CC:integral component of membrane;CC:plasma membrane;
BAB_RS17555	-	CAP domain-containing protein	1.391	0.476527736	6.71002E-05	0.001427769	no	up	mRNA	204.49	182.98	204.92	276.66	271.08	234.81	197.463	260.85	COG2340	S:Function unknown	------	------
BAB_RS22810	-	cell division protein FtsZ	1.387	0.471707963	5.7186E-06	0.000244985	no	up	mRNA	1205.76	1159	1249.73	1524	1563.38	1634.39	1204.83	1573.9233	COG0206	D:Cell cycle control, cell division, chromosome partitioning	GO:0090529;GO:0043093;GO:0051258;GO:0051258;GO:0043093;GO:0090529;GO:0032153;GO:0005737;GO:0005737;GO:0032153;GO:0005525;GO:0003924;GO:0005525;GO:0003924;GO:0005525	BP:cell septum assembly;BP:FtsZ-dependent cytokinesis;BP:protein polymerization;BP:protein polymerization;BP:FtsZ-dependent cytokinesis;BP:cell septum assembly;CC:cell division site;CC:cytoplasm;CC:cytoplasm;CC:cell division site;MF:GTP binding;MF:GTPase activity;MF:GTP binding;MF:GTPase activity;MF:GTP binding;
BAB_RS29215	-	acetyl-CoA C-acyltransferase	1.387	0.471958461	6.49782E-05	0.001420237	no	up	mRNA	47.47	47.15	50.71	66.29	66.2	57.71	48.4433	63.4	COG0183	I:Lipid transport and metabolism	GO:0008152;GO:0008152;GO:0003988;GO:0016747;GO:0008966;GO:0016747	BP:metabolic process;BP:metabolic process;MF:acetyl-CoA C-acyltransferase activity;MF:transferase activity, transferring acyl groups other than amino-acyl groups;MF:phosphoglucosamine mutase activity;MF:transferase activity, transferring acyl groups other than amino-acyl groups;
BAB_RS19970	-	hypothetical protein	1.382	0.466470767	3.55274E-06	0.000170373	no	up	mRNA	118.49	112.89	120.19	156.77	153.51	147.55	117.19	152.61	ENOG410ZUWP	S:Function unknown	------	------
BAB_RS26430	-	isochorismatase	1.382	0.467219871	0.001961611	0.012968425	no	up	mRNA	36.2	39.11	33.38	41.08	48.83	52.42	36.23	47.443333	COG3433	Q:Secondary metabolites biosynthesis, transport and catabolism	GO:0008152;GO:0008152;GO:0008908;GO:0008908	BP:metabolic process;BP:metabolic process;MF:isochorismatase activity;MF:isochorismatase activity;
BAB_RS33420	-	hypothetical protein	1.38	0.464637614	0.00183985	0.012392955	no	up	mRNA	10.53	9.07	12.4	15.48	13.57	12.48	10.6667	13.843333	COG0516	F:Nucleotide transport and metabolism	GO:0006177;GO:0003938;GO:0046872;GO:0000166	BP:GMP biosynthetic process;MF:IMP dehydrogenase activity;MF:metal ion binding;MF:nucleotide binding;
BAB_RS27940	-	DUF1338 domain-containing protein	1.379	0.463328225	1.52995E-05	0.000534317	no	up	mRNA	945.66	915.59	965.06	1138.14	1238.88	1296.92	942.103	1224.6467	COG5383	S:Function unknown	------	------
BAB_RS31995	-	hypothetical protein	1.376	0.460567626	0.000568449	0.005725474	no	up	mRNA	35.36	33.1	48.12	50.94	50.71	48.84	38.86	50.163333	------	------	------	------
BAB_RS30115	-	hypothetical protein	1.376	0.460866271	0.004827826	0.024275125	no	up	mRNA	72.43	68.34	78.25	72.23	113.22	98.53	73.0067	94.66	ENOG410XP8J	G:Carbohydrate transport and metabolism	GO:0055085;GO:0016021;GO:0016021;GO:0005215	BP:transmembrane transport;CC:integral component of membrane;CC:integral component of membrane;MF:transporter activity;
BAB_RS24840	-	ATP-dependent chaperone ClpB	1.375	0.459791725	1.10828E-05	0.0004001	no	up	mRNA	804.04	744.1	823.46	1050.72	1043.86	972.58	790.533	1022.3867	COG0542	S:Function unknown	GO:0019538;GO:0042026;GO:0009408;GO:0019538;GO:0042026;GO:0009408;GO:0005737;GO:0005737;GO:0005524;GO:0008233;GO:0005524	BP:protein metabolic process;BP:protein refolding;BP:response to heat;BP:protein metabolic process;BP:protein refolding;BP:response to heat;CC:cytoplasm;CC:cytoplasm;MF:ATP binding;MF:peptidase activity;MF:ATP binding;
BAB_RS24965	-	EamA/RhaT family transporter	1.373	0.4578492	0.000127615	0.002169462	no	up	mRNA	44.17	41.5	43.66	61.29	50.49	55.5	43.11	55.76	COG0697	S:Function unknown	GO:0016021	CC:integral component of membrane;
BAB_RS30770	-	nitrite reductase%2C copper-containing	1.373	0.45767308	0.016960122	0.0570606	no	up	mRNA	236.72	245.92	234.26	262.2	417.06	250.94	238.967	310.06667	COG2132	S:Function unknown	GO:0006807;GO:0006807;GO:0006807;GO:0005507;GO:0003919;GO:0050421;GO:0005507;GO:0050421;GO:0003919;GO:0005507;GO:0050421	BP:nitrogen compound metabolic process;BP:nitrogen compound metabolic process;BP:nitrogen compound metabolic process;MF:copper ion binding;MF:FMN adenylyltransferase activity;MF:nitrite reductas;MF:copper ion binding;MF:nitrite reductas;MF:FMN adenylyltransferase activity;MF:copper ion binding;MF:nitrite reductas;
BAB_RS27935	-	aldehyde dehydrogenase family protein	1.372	0.456694415	1.16218E-05	0.000414897	no	up	mRNA	701.88	652.63	703.9	839.77	904.84	913.25	686.137	885.95333	COG1012	F:Nucleotide transport and metabolism	GO:0016620	MF:oxidoreductase activity, acting on the aldehyde or oxo group of donors, NAD or NADP as acceptor;
BAB_RS20335	-	NADH:ubiquinone oxidoreductase subunit NDUFA12	1.372	0.456125034	2.69719E-05	0.000795052	no	up	mRNA	383.83	401.97	405.7	524.28	532.18	518.42	397.167	524.96	COG3761	C:Energy production and conversion	GO:0016020;GO:0016020;GO:0009055;GO:0008137;GO:0008137;GO:0009055	CC:membrane;CC:membrane;MF:electron carrier activity;MF:NADH dehydrogenas;MF:NADH dehydrogenas;MF:electron carrier activity;
BAB_RS26460	-	pentapeptide MXKDX repeat protein	1.371	0.455482206	0.014621615	0.051008955	no	up	mRNA	53.32	45.74	43.69	60.25	64.73	66.92	47.5833	63.966667	ENOG410XVJD	S:Function unknown	------	------
BAB_RS30025	-	DUF1956 domain-containing protein	1.368	0.452092689	0.00442691	0.022978454	no	up	mRNA	81.75	84.07	93.59	136.8	105.67	94.35	86.47	112.27333	COG1309	V:Defense mechanisms	GO:0006355;GO:0006351;GO:0006355;GO:0006351;GO:0003677;GO:0003677	BP:regulation of transcription, DNA-templated;BP:transcription, DNA-templated;BP:regulation of transcription, DNA-templated;BP:transcription, DNA-templated;MF:DNA binding;MF:DNA binding;
BAB_RS31165	-	peptide chain release factor 3	1.366	0.449947424	0.000148434	0.002408686	no	up	mRNA	40.23	38.81	40.01	56.32	49.8	47.03	39.6833	51.05	COG4108	J:Translation, ribosomal structure and biogenesis	GO:0006449;GO:0005737;GO:0016149;GO:0003924;GO:0003746;GO:0005525	BP:regulation of translational termination;CC:cytoplasm;MF:translation release factor activity, codon specific;MF:GTPase activity;MF:translation elongation factor activity;MF:GTP binding;
BAB_RS21935	-	elongation factor G	1.364	0.447415192	0.000344759	0.004117886	no	up	mRNA	781.55	680.5	774.61	1086.57	856.98	923.49	745.553	955.68	COG0480	J:Translation, ribosomal structure and biogenesis	GO:0005737;GO:0005737;GO:0005525;GO:0003924;GO:0003746;GO:0003746;GO:0003924;GO:0005525	CC:cytoplasm;CC:cytoplasm;MF:GTP binding;MF:GTPase activity;MF:translation elongation factor activity;MF:translation elongation factor activity;MF:GTPase activity;MF:GTP binding;
BAB_RS27810	-	D-amino-acid transaminase	1.363	0.446322282	5.32208E-05	0.001239119	no	up	mRNA	144.98	133.92	139.62	161.83	185.49	190.55	139.507	179.29	COG0115	G:Carbohydrate transport and metabolism	GO:0008152;GO:0047810;GO:0008483;GO:0003824	BP:metabolic process;MF:D-alanin;MF:transaminase activity;MF:catalytic activity;
BAB_RS30815	-	CbbQ/NirQ/NorQ/GpvN family protein	1.362	0.445210397	0.068666934	0.157141637	no	up	mRNA	60.47	88.72	47.85	68.47	101.65	85.35	65.68	85.156667	COG0714	P:Inorganic ion transport and metabolism	GO:0016887;GO:0005524;GO:0005524;GO:0016887;GO:0016966;GO:0005524;GO:0016887;GO:0016491	MF:ATPase activity;MF:ATP binding;MF:ATP binding;MF:ATPase activity;MF:nitric oxide reductase activity;MF:ATP binding;MF:ATPase activity;MF:oxidoreductase activity;
BAB_RS24525	-	ATP synthase subunit alpha	1.361	0.44491388	0.000122747	0.002131819	no	up	mRNA	582.63	531.75	584.45	814.77	674.72	687.88	566.277	725.79	COG0056	C:Energy production and conversion	GO:0015986;GO:0015986;GO:0005886;GO:0045261;GO:0045261;GO:0005886;GO:0005524;GO:0046933;GO:0046933;GO:0016798;GO:0005524	BP:ATP synthesis coupled proton transport;BP:ATP synthesis coupled proton transport;CC:plasma membrane;CC:proton-transporting ATP synthase complex, catalytic core;CC:proton-transporting ATP synthase complex, catalytic core;CC:plasma membrane;MF:ATP binding;MF:proton-transporting ATP synthase activity, rotational mechanism;MF:proton-transporting ATP synthase activity, rotational mechanism;MF:hydrolase activity, acting on glycosyl bonds;MF:ATP binding;
BAB_RS26440	-	isochorismate synthase	1.36	0.443527403	0.005381031	0.026315452	no	up	mRNA	46.48	60.63	46.93	62.7	73.48	62.56	51.3467	66.246667	COG1169	H:Coenzyme transport and metabolism	GO:0009058;GO:0008909;GO:0047373	BP:biosynthetic process;MF:isochorismate synthase activity;MF:acetoxybutynylbithiophene deacetylase activity;
BAB_RS26770	-	rhodanese domain-containing protein	1.358	0.441518394	3.88526E-05	0.001031682	no	up	mRNA	82.22	76.97	88.12	112.61	101.23	103.29	82.4367	105.71	COG1054	S:Function unknown	------	------
BAB_RS21040	-	NAD(P)H dehydrogenase (quinone)	1.355	0.438049057	1.96952E-05	0.000632807	no	up	mRNA	422.85	395.93	423.78	483.89	559.07	550.63	414.187	531.19667	COG0655	S:Function unknown	GO:0051287;GO:0050661;GO:0050660;GO:0003955;GO:0010181;GO:0050661;GO:0050660;GO:0051287;GO:0016740;GO:0010181;GO:0003955	MF:NAD binding;MF:NADP binding;MF:flavin adenine dinucleotide binding;MF:NA;MF:FMN binding;MF:NADP binding;MF:flavin adenine dinucleotide binding;MF:NAD binding;MF:transferase activity;MF:FMN binding;MF:NA;
BAB_RS32365	-	2-isopropylmalate synthase	1.355	0.438354873	0.062641972	0.146483738	no	up	mRNA	511	905.66	437.12	711.5	1007.19	818.46	617.927	845.71667	------	------	GO:0009098;GO:0003852;GO:0003852	BP:leucine biosynthetic process;MF:2-isopropylmalate synthase activity;MF:2-isopropylmalate synthase activity;
BAB_RS23085	-	carbamoyl phosphate synthase small subunit	1.354	0.436863602	4.69673E-05	0.001169814	no	up	mRNA	145.23	130.26	134.66	185.31	173.55	163.58	136.717	174.14667	COG0505	F:Nucleotide transport and metabolism	GO:0006207;GO:0006541;GO:0006526;GO:0044205;GO:0006207;GO:0044205;GO:0006526;GO:0006541;GO:0006207;GO:0044205;GO:0006526;GO:0006541;GO:0004088;GO:0005524;GO:0005524;GO:0004088;GO:0016491;GO:0005524;GO:0004088	BP:'de novo' pyrimidine nucleobase biosynthetic process;BP:glutamine metabolic process;BP:arginine biosynthetic process;BP:'de novo' UMP biosynthetic process;BP:'de novo' pyrimidine nucleobase biosynthetic process;BP:'de novo' UMP biosynthetic process;BP:arginine biosynthetic process;BP:glutamine metabolic process;BP:'de novo' pyrimidine nucleobase biosynthetic process;BP:'de novo' UMP biosynthetic process;BP:arginine biosynthetic process;BP:glutamine metabolic process;MF:carbamoyl-phosphate synthas;MF:ATP binding;MF:ATP binding;MF:carbamoyl-phosphate synthas;MF:oxidoreductase activity;MF:ATP binding;MF:carbamoyl-phosphate synthas;
BAB_RS28015	-	hypothetical protein	1.354	0.437629904	0.009306609	0.037802951	no	up	mRNA	512.85	507.38	554.52	699.54	735.7	752.7	524.917	729.31333	COG2835	S:Function unknown	------	------

BAB_RS19565	-	phosphatase PAP2 family protein	1.353	0.435708173	0.000221796	0.003125573	no	up	mRNA	46.22	50.14	47.85	65.36	59.45	59.78	48.07	61.53	COG0671	I:Lipid transport and metabolism	GO:0016021	CC:integral component of membrane;
BAB_RS18560	-	IS5/IS1182 family transposase	1.353	0.436005539	0.55610399	0.700416354	no	up	mRNA	27.86	9.37	1.97	17.11	15.37	8.14	13.0667	13.54	COG3293	L:Replication, recombination and repair	------	------
BAB_RS19010	-	MFS transporter	1.348	0.43049503	3.25249E-05	0.000900882	no	up	mRNA	31.59	29.52	32.16	39.47	40.72	38.12	31.09	39.436667	ENOG410Y0GE	G:Carbohydrate transport and metabolism	GO:0055085;GO:0055085;GO:0016021;GO:0016021	BP:transmembrane transport;BP:transmembrane transport;CC:integral component of membrane;CC:integral component of membrane;
BAB_RS22080	-	hypothetical protein	1.347	0.429634976	5.29931E-05	0.001242823	no	up	mRNA	426.86	443.24	465.37	600.44	577.63	523.34	445.157	567.13667	COG4395	S:Function unknown	GO:0016021	CC:integral component of membrane;
BAB_RS17915	-	radical SAM protein	1.345	0.427187497	9.8154E-05	0.00185511	no	up	mRNA	25.16	22.97	27.52	32.68	31.01	32.19	25.2167	31.96	COG1533	L:Replication, recombination and repair	GO:0003824;GO:0051536	MF:catalytic activity;MF:iron-sulfur cluster binding;
BAB_RS32660	-	hypothetical protein	1.345	0.42723171	0.003000613	0.017689855	no	up	mRNA	81.07	68.64	107.28	115.86	107.9	100.29	85.6633	108.01667	COG0050	J:Translation, ribosomal structure and biogenesis	GO:0005737;GO:0016021;GO:0005525;GO:0003924;GO:0003746	CC:cytoplasm;CC:integral component of membrane;MF:GTP binding;MF:GTPase activity;MF:translation elongation factor activity;
BAB_RS25705	-	flavin-dependent oxidoreductase	1.344	0.426093565	0.000688252	0.006465951	no	up	mRNA	19.44	14.76	19.52	22.45	22.88	22.3	17.9067	22.543333	COG2141	S:Function unknown	GO:0016705;GO:0004497;GO:0047646	MF:oxidoreductase activity, acting on paired donors, with incorporation or reduction of molecular oxygen;MF:monooxygenase activity;MF:alkanal monooxygenas;
BAB_RS31085	-	penicillin-insensitive murein endopeptidase	1.343	0.425429754	5.49516E-05	0.001252194	no	up	mRNA	142.23	140.26	167.53	178.53	194.21	197.62	150.007	190.12	COG3770	M:Cell wall/membrane/envelope biogenesis	GO:0030288;GO:0004252	CC:outer membrane-bounded periplasmic space;MF:serine-type endopeptidase activity;
BAB_RS31780	-	sugar ABC transporter permease	1.343	0.425191616	0.118132792	0.233002247	no	up	mRNA	5.02	3.1	2.7	4.86	4.18	4.2	3.60667	4.4133333	COG0395	S:Function unknown	GO:0008643;GO:0055085;GO:0055085;GO:0016021;GO:0005886;GO:0016021	BP:carbohydrate transport;BP:transmembrane transport;BP:transmembrane transport;CC:integral component of membrane;CC:plasma membrane;CC:integral component of membrane;
BAB_RS33385	-	multidrug ABC transporter ATP-binding protein	1.341	0.423310688	0.001865067	0.012432492	no	up	mRNA	44.18	42.55	51.62	67.49	53.74	54.41	46.1167	58.546667	COG1131	V:Defense mechanisms	GO:0005524;GO:0016887	MF:ATP binding;MF:ATPase activity;
BAB_RS30555	-	trehalose utilization protein ThuA	1.341	0.42348946	0.003391402	0.019116799	no	up	mRNA	24.23	17.75	23.27	28.56	27.57	26.08	21.75	27.403333	COG4813	G:Carbohydrate transport and metabolism	------	------
BAB_RS24030	-	adenylosuccinate synthetase	1.34	0.422579049	4.15997E-05	0.001086665	no	up	mRNA	148.8	144.69	150.47	201.96	182.52	176.18	147.987	186.88667	COG0104	F:Nucleotide transport and metabolism	GO:0044208;GO:0044208;GO:0044208;GO:0005737;GO:0005737;GO:0005737;GO:0004019;GO:0005525;GO:0046872;GO:0004019;GO:0000287;GO:0046872;GO:0005525;GO:0004019;GO:0005525;GO:0000287	BP:'de novo' AMP biosynthetic process;BP:'de novo' AMP biosynthetic process;BP:'de novo' AMP biosynthetic process;CC:cytoplasm;CC:cytoplasm;CC:cytoplasm;MF:adenylosuccinate synthase activity;MF:GTP binding;MF:metal ion binding;MF:adenylosuccinate synthase activity;MF:magnesium ion binding;MF:metal ion binding;MF:GTP binding;MF:adenylosuccinate synthase activity;MF:GTP binding;MF:magnesium ion binding;
BAB_RS23860	-	HNH endonuclease	1.34	0.422622427	0.000348135	0.004127515	no	up	mRNA	91.43	93.59	112.98	112.7	130.17	133.85	99.3333	125.57333	COG1403	V:Defense mechanisms	GO:0004519;GO:0016787;GO:0004519	MF:endonuclease activity;MF:hydrolase activity;MF:endonuclease activity;
BAB_RS18655	-	LacI family DNA-binding transcriptional regulator	1.34	0.422649833	0.006939622	0.031184625	no	up	mRNA	33.6	28.94	33.87	47.65	41.37	32.5	32.1367	40.506667	ENOG410XTAD	K:Transcription	GO:0006355;GO:0006351;GO:0003677	BP:regulation of transcription, DNA-templated;BP:transcription, DNA-templated;MF:DNA binding;
BAB_RS28785	-	NAD(P)/FAD-dependent oxidoreductase	1.339	0.421599216	0.000498777	0.005271609	no	up	mRNA	62.91	60.59	77.12	93.37	81.08	78.27	66.8733	84.24	COG0446	C:Energy production and conversion	GO:0050660;GO:0016491	MF:flavin adenine dinucleotide binding;MF:oxidoreductase activity;
BAB_RS22085	-	hypothetical protein	1.336	0.418381783	0.007295487	0.032110137	no	up	mRNA	31.47	26.7	41.87	43.65	45.04	37.33	33.3467	42.006667	COG4395	S:Function unknown	------	------
BAB_RS22000	-	elongation factor Tu	1.334	0.415885104	0.000263255	0.003383353	no	up	mRNA	713.25	680.92	696.08	970.45	787.86	872.39	696.75	876.9	COG0050	J:Translation, ribosomal structure and biogenesis	GO:0005737;GO:0005737;GO:0005525;GO:0003924;GO:0003746;GO:0003746;GO:0003743;GO:0003924;GO:0005525	CC:cytoplasm;CC:cytoplasm;MF:GTP binding;MF:GTPase activity;MF:translation elongation factor activity;MF:translation elongation factor activity;MF:translation initiation factor activity;MF:GTPase activity;MF:GTP binding;
BAB_RS27460	-	sensor domain-containing diguanylate cyclase	1.334	0.415261668	0.258080575	0.412338581	no	up	mRNA	0.73	1.03	1.5	1.23	1.7	1.31	1.08667	1.4133333	ENOG4111Q6T;ENOG410XNMH	T:Signal transduction mechanisms;	------	------
BAB_RS33365	-	hypothetical protein	1.334	0.415942746	0.47702198	0.638879376	no	up	mRNA	0	217.43	171.02	169.65	225.04	259.09	129.483	217.92667	------	------	------	------
BAB_RS24835	-	L%2CD-transpeptidase	1.331	0.412356804	9.21841E-06	0.000348456	no	up	mRNA	123.53	114.13	125.17	156.11	144.71	153.48	120.943	151.43333	COG3409	S:Function unknown	GO:0016740	MF:transferase activity;
BAB_RS17925	-	hypothetical protein	1.331	0.412107872	0.185026459	0.323092398	no	up	mRNA	76.27	51.07	69.88	103.33	93.86	74.46	65.74	90.55	------	------	------	------
BAB_RS19990	-	30S ribosomal protein S4	1.33	0.41127565	0.000433791	0.004724649	no	up	mRNA	824.05	660.21	730.83	1023.7	863.67	895.24	738.363	927.53667	COG0522	J:Translation, ribosomal structure and biogenesis	GO:0006412;GO:0006412;GO:0006412;GO:0015935;GO:0015935;GO:0015935;GO:0019843;GO:0003735;GO:0003735;GO:0019843;GO:0003735;GO:0019843	BP:translation;BP:translation;BP:translation;CC:small ribosomal subunit;CC:small ribosomal subunit;CC:small ribosomal subunit;MF:rRNA binding;MF:structural constituent of ribosome;MF:structural constituent of ribosome;MF:rRNA binding;MF:structural constituent of ribosome;MF:rRNA binding;
BAB_RS29805	-	cytochrome d ubiquinol oxidase subunit II	1.328	0.409264003	5.37243E-05	0.001232973	no	up	mRNA	1118.12	1039.35	1083.7	1311.63	1291.55	1459.59	1080.39	1354.2567	COG1294	C:Energy production and conversion	GO:0055114;GO:0055114;GO:0055114;GO:0016021;GO:0016020;GO:0016021;GO:0016021;GO:0004565;GO:0016491;GO:0004565;GO:0016491	BP:oxidation-reduction process;BP:oxidation-reduction process;BP:oxidation-reduction process;CC:integral component of membrane;CC:membrane;CC:integral component of membrane;CC:integral component of membrane;MF:beta-galactosidase activity;MF:oxidoreductase activity;MF:beta-galactosidase activity;MF:oxidoreductase activity;
BAB_RS18380	-	restriction endonuclease subunit M	1.328	0.409419641	0.00033906	0.004095485	no	up	mRNA	186.58	170.49	191.62	218.01	207.46	260.86	182.897	228.77667	COG0863	L:Replication, recombination and repair	GO:0006306;GO:0006306;GO:0006260;GO:0006260;GO:0003677;GO:0008170;GO:0008170;GO:0009007;GO:0003677;GO:0003677;GO:0008170;GO:0009007	BP:DNA methylation;BP:DNA methylation;BP:DNA replication;MF:DNA binding;MF:N-methyltransferase activity;MF:N-methyltransferase activity;MF:site-specific DNA-methyltransferas;MF:DNA binding;MF:DNA binding;MF:N-methyltransferase activity;MF:site-specific DNA-methyltransferas;
BAB_RS27165	-	cytochrome c family protein	1.327	0.407672196	0.017660411	0.058740063	no	up	mRNA	427.81	461.65	439.99	423.13	704.06	541.69	443.15	556.29333	COG3474;COG3474	M:Cell wall/membrane/envelope biogenesis;U:Intracellular trafficking, secretion, and vesicular transport	GO:0009055;GO:0020037;GO:0046872;GO:0046872;GO:0020037;GO:0009055	MF:electron carrier activity;MF:heme binding;MF:metal ion binding;MF:metal ion binding;MF:heme binding;MF:electron carrier activity;
BAB_RS16275	-	hypothetical protein	1.324	0.405018374	0.00010454	0.001941543	no	up	mRNA	34.94	32.9	38.56	44.98	43.3	44.71	35.4667	44.33	------	------	GO:0016021	CC:integral component of membrane;
BAB_RS26265	-	polyribonucleotide nucleotidyltransferase	1.324	0.404818516	0.000112649	0.002022024	no	up	mRNA	1019.7	930.46	1001.09	1306.85	1137.98	1232.13	983.75	1225.6533	COG1185	J:Translation, ribosomal structure and biogenesis	GO:0006402;GO:0006396;GO:0006402;GO:0006396;GO:0005737;GO:0005737;GO:0005840;GO:0000287;GO:0004654;GO:0003723;GO:0046872;GO:0000287;GO:0004654;GO:0003723;GO:0016757;GO:0046872	BP:mRNA catabolic process;BP:RNA processing;BP:mRNA catabolic process;BP:RNA processing;CC:cytoplasm;CC:cytoplasm;CC:ribosome;MF:magnesium ion binding;MF:polyribonucleotide nucleotidyltransferase activity;MF:RNA binding;MF:metal ion binding;MF:magnesium ion binding;MF:polyribonucleotide nucleotidyltransferase activity;MF:RNA binding;MF:transferase activity, transferring glycosyl groups;MF:metal ion binding;
BAB_RS23170	-	transcription elongation factor GreA	1.323	0.4036919	0.000105724	0.001930057	no	up	mRNA	373.93	307.68	325.12	433.92	405.49	424.05	335.577	421.15333	COG0782	P:Inorganic ion transport and metabolism	GO:0032784;GO:0006351;GO:0032784;GO:0006351;GO:0003677;GO:0070063;GO:0003746;GO:0070063;GO:0003746;GO:0003677	BP:regulation of DNA-templated transcription, elongation;BP:transcription, DNA-templated;BP:regulation of DNA-templated transcription, elongation;BP:transcription, DNA-templated;MF:DNA binding;MF:RNA polymerase binding;MF:translation elongation factor activity;MF:RNA polymerase binding;MF:translation elongation factor activity;MF:DNA binding;
BAB_RS25075	-	tyrosine recombinase XerC	1.323	0.40391095	0.00040233	0.004504133	no	up	mRNA	42.56	45.86	44.02	58.3	53.64	54.01	44.1467	55.316667	COG0582;COG4974	J:Translation, ribosomal structure and biogenesis;L:Replication, recombination and repair	GO:0007049;GO:0051301;GO:0007059;GO:0006313;GO:0006310;GO:0006313;GO:0015074;GO:0051301;GO:0007059;GO:0007049;GO:0006313;GO:0007049;GO:0007059;GO:0051301;GO:0005737;GO:0005737;GO:0005737;GO:0003677;GO:0009037;GO:0009037;GO:0003677;GO:0009037;GO:0003677	BP:cell cycle;BP:cell division;BP:chromosome segregation;BP:transposition, DNA-mediated;BP:DNA recombination;BP:transposition, DNA-mediated;BP:DNA integration;BP:cell division;BP:chromosome segregation;BP:cell cycle;BP:transposition, DNA-mediated;BP:cell cycle;BP:chromosome segregation;BP:cell division;CC:cytoplasm;CC:cytoplasm;CC:cytoplasm;MF:DNA binding;MF:tyrosine-based site-specific recombinase activity;MF:tyrosine-based site-specific recombinase activity;MF:DNA binding;MF:tyrosine-based site-specific recombinase activity;MF:DNA binding;
BAB_RS28795	-	glycine cleavage system aminomethyltransferase T	1.322	0.403175072	0.000315063	0.003849042	no	up	mRNA	453.96	434.15	528.07	651.12	567.69	542.54	472.06	587.11667	COG0404	E:Amino acid transport and metabolism	GO:0006546;GO:0004047;GO:0047586;GO:0008483	BP:glycine catabolic process;MF:aminomethyltransferase activity;MF:5'-acylphosphoadenosine hydrolase activity;MF:transaminase activity;
BAB_RS20525	-	copper chaperone	1.322	0.402375841	0.027803364	0.080407027	no	up	mRNA	7018.3	6973.66	6286.73	10747.57	8990.2	7306.17	6759.56	9014.6467	ENOG410XUQ1;COG2608	P:Inorganic ion transport and metabolism;S:Function unknown	GO:0030001;GO:0046872	BP:metal ion transport;MF:metal ion binding;
BAB_RS25030	-	50S ribosomal protein L19	1.32	0.400683241	0.000151858	0.002451865	no	up	mRNA	796.21	669.84	764.71	1013.57	880.08	894.21	743.587	929.28667	COG0335	J:Translation, ribosomal structure and biogenesis	GO:0006412;GO:0005840;GO:0003735	BP:translation;CC:ribosome;MF:structural constituent of ribosome;
BAB_RS29810	-	cytochrome bd oxidase subunit I	1.319	0.399919002	0.000118859	0.002109915	no	up	mRNA	1402.51	1317.15	1314.16	1628.42	1617.58	1774.14	1344.61	1673.38	COG1271	C:Energy production and conversion	GO:0019646;GO:0019646;GO:0070069;GO:0016021;GO:0016021;GO:0070069;GO:0009055;GO:0009055	BP:aerobic electron transport chain;BP:aerobic electron transport chain;CC:cytochrome complex;CC:integral component of membrane;CC:integral component of membrane;CC:cytochrome complex;MF:electron carrier activity;MF:electron carrier activity;
BAB_RS19625	-	aspartate--tRNA(Asp/Asn) ligase	1.319	0.399105827	0.000343807	0.004121831	no	up	mRNA	69.77	56.72	61.76	81.71	72.71	78.82	62.75	77.746667	COG0173	J:Translation, ribosomal structure and biogenesis	GO:0006422;GO:0006418;GO:0006422;GO:0006418;GO:0006422;GO:0005737;GO:0005737;GO:0005737;GO:0016787;GO:0004815;GO:0003676;GO:0050560;GO:0005524;GO:0016787;GO:0050560;GO:0003676;GO:0004815;GO:0005524;GO:0004815;GO:0050560;GO:0005524;GO:0003676	BP:aspartyl-tRNA aminoacylation;BP:tRNA aminoacylation for protein translation;BP:aspartyl-tRNA aminoacylation;BP:tRNA aminoacylation for protein translation;BP:aspartyl-tRNA aminoacylation;CC:cytoplasm;CC:cytoplasm;CC:cytoplasm;MF:hydrolase activity;MF:aspartate-tRNA ligase activity;MF:nucleic acid binding;MF:aspartate-tRN;MF:ATP binding;MF:hydrolase activity;MF:aspartate-tRN;MF:nucleic acid binding;MF:aspartate-tRNA ligase activity;MF:ATP binding;MF:aspartate-tRNA ligase activity;MF:aspartate-tRN;MF:ATP binding;MF:nucleic acid binding;
BAB_RS31280	-	enoyl-CoA hydratase	1.319	0.399398654	0.041026269	0.107518273	no	up	mRNA	25.72	20.05	26.7	35.79	32.31	21.61	24.1567	29.903333	COG1024	C:Energy production and conversion	GO:0008152;GO:0008152;GO:0003824;GO:0003859	BP:metabolic process;BP:metabolic process;MF:catalytic activity;MF:3-hydroxybutyryl-CoA dehydratase activity;
BAB_RS27110	-	flagellar biosynthetic protein FliP	1.319	0.399861455	0.444062933	0.61025415	no	up	mRNA	1.03	0.73	1.45	1.62	0.63	1.99	1.07	1.4133333	COG1338	N:Cell motility	GO:0044781;GO:0009306;GO:0016021;GO:0009425;GO:0005886	BP:bacterial-type flagellum organization;BP:protein secretion;CC:integral component of membrane;CC:bacterial-type flagellum basal body;CC:plasma membrane;
BAB_RS27390	-	acyl-CoA dehydrogenase	1.317	0.397270764	0.000391431	0.004444052	no	up	mRNA	32.09	30.8	35.51	44.03	40.21	38.03	32.8	40.756667	COG1960	G:Carbohydrate transport and metabolism	GO:0003995;GO:0050660	MF:acyl-CoA dehydrogenase activity;MF:flavin adenine dinucleotide binding;
BAB_RS18760	-	S9 family peptidase	1.316	0.395643699	0.000117446	0.002096417	no	up	mRNA	98.18	82.07	86.15	111.22	110.09	108.14	88.8	109.81667	COG1770	E:Amino acid transport and metabolism	GO:0004252;GO:0070008	MF:serine-type endopeptidase activity;MF:serine-type exopeptidase activity;
BAB_RS25375	-	argininosuccinate lyase	1.316	0.396201617	0.001453351	0.010400037	no	up	mRNA	35.16	28.53	29.94	41.4	35.14	39.14	31.21	38.56	COG0165	E:Amino acid transport and metabolism	GO:0042450;GO:0042450;GO:0042450;GO:0005737;GO:0005737;GO:0005737;GO:0004056;GO:0004056;GO:0004056;GO:0016746	BP:arginine biosynthetic process via ornithine;BP:arginine biosynthetic process via ornithine;BP:arginine biosynthetic process via ornithine;CC:cytoplasm;CC:cytoplasm;CC:cytoplasm;MF:argininosuccinate lyase activity;MF:argininosuccinate lyase activity;MF:argininosuccinate lyase activity;MF:transferase activity, transferring acyl groups;
BAB_RS25895	-	phosphoenolpyruvate carboxykinase	1.315	0.394947152	0.019678132	0.063864482	no	up	mRNA	4.78	3.52	4.41	5.37	4.89	5.38	4.23667	5.2133333	COG1866	C:Energy production and conversion	GO:0006094;GO:0006094;GO:0005737;GO:0005737;GO:0016301;GO:0004612;GO:0046872;GO:0005524;GO:0016787;GO:0016301;GO:0004612;GO:0046872;GO:0005524	BP:gluconeogenesis;BP:gluconeogenesis;CC:cytoplasm;CC:cytoplasm;MF:kinase activity;MF:phosphoenolpyruvate carboxykinas;MF:metal ion binding;MF:ATP binding;MF:hydrolase activity;MF:kinase activity;MF:phosphoenolpyruvate carboxykinas;MF:metal ion binding;MF:ATP binding;
BAB_RS30525	-	class 1b ribonucleoside-diphosphate reductase subunit alpha	1.314	0.393691611	0.000141314	0.002316541	no	up	mRNA	345.94	286.06	320.04	373.22	384.24	418.64	317.347	392.03333	COG0209	F:Nucleotide transport and metabolism	GO:0006260;GO:0004748;GO:0005524	BP:DNA replication;MF:ribonucleoside-diphosphate reductase activity, thioredoxin disulfide as acceptor;MF:ATP binding;
BAB_RS17345	-	urease subunit gamma	1.313	0.39303393	0.02539411	0.07604033	no	up	mRNA	81.09	61.36	59.12	93.68	80.14	82.48	67.19	85.433333	COG0831	E:Amino acid transport and metabolism	GO:0009405;GO:0043419;GO:0043419;GO:0005737;GO:0005737;GO:0009039;GO:0016151;GO:0016151;GO:0009039	BP:pathogenesis;BP:urea catabolic process;BP:urea catabolic process;CC:cytoplasm;CC:cytoplasm;MF:urease activity;MF:nickel cation binding;MF:nickel cation binding;MF:urease activity;
BAB_RS32215	-	hypothetical protein	1.312	0.391451408	0.193552171	0.333807367	no	up	mRNA	15.62	15.8	28.45	32.41	22.48	25.21	19.9567	26.7	------	------	------	------
BAB_RS26645	-	type IV secretion system protein virB9	1.311	0.390631462	0.015990326	0.054598212	no	up	mRNA	16.38	20.06	22.32	26.1	21.15	26.6	19.5867	24.616667	COG3504	U:Intracellular trafficking, secretion, and vesicular transport	GO:0009405	BP:pathogenesis;
BAB_RS20190	-	DUF533 domain-containing protein	1.307	0.385758742	0.000252967	0.003303997	no	up	mRNA	616.45	554.78	575.11	748.13	767.17	644.07	582.113	719.79	COG2979	S:Function unknown	------	------
BAB_RS26435	entE	2%2C3-dihydroxybenzoate-AMP ligase	1.307	0.385763218	0.004599956	0.023647452	no	up	mRNA	20.81	21.09	17.92	22.19	26.87	24.48	19.94	24.513333	COG1021	Q:Secondary metabolites biosynthesis, transport and catabolism	GO:0019290;GO:0019290;GO:0019290;GO:0008668;GO:0047969;GO:0016874;GO:0008668;GO:0016874;GO:0008668;GO:0016874	BP:siderophore biosynthetic process;BP:siderophore biosynthetic process;BP:siderophore biosynthetic process;MF:;MF:glyoxylate oxidase activity;MF:ligase activity;MF:;MF:ligase activity;MF:;MF:ligase activity;
BAB_RS19535	-	hypothetical protein	1.306	0.3850574	0.308063309	0.469770959	no	up	mRNA	1.88	2.22	1.96	1.88	2.55	3.22	2.02	2.55	ENOG410YJV0	S:Function unknown	------	------
BAB_RS18440	-	MBL fold metallo-hydrolase	1.305	0.383531494	0.001371873	0.010132939	no	up	mRNA	34.99	33.56	40.42	49.46	41.12	43.87	36.3233	44.816667	COG0491	S:Function unknown	GO:0016787	MF:hydrolase activity;
BAB_RS23855	-	disulfide bond formation protein B	1.304	0.383434281	2.75005E-05	0.000803264	no	up	mRNA	120.43	114.29	130.1	156.75	147.24	144.07	121.607	149.35333	COG1495	O:Posttranslational modification, protein turnover, chaperones	GO:0016021;GO:0005886;GO:0016021;GO:0005886;GO:0015035;GO:0015035	CC:integral component of membrane;CC:plasma membrane;CC:integral component of membrane;CC:plasma membrane;MF:protein disulfide oxidoreductase activity;MF:protein disulfide oxidoreductase activity;
BAB_RS21625	-	ATP-dependent Clp protease ATP-binding subunit ClpA	1.304	0.382552767	5.07281E-05	0.001234768	no	up	mRNA	596.9	558.09	601.4	691.9	753.38	708.5	585.463	717.92667	COG0542	O:Posttranslational modification, protein turnover, chaperones	GO:0043335;GO:0043335;GO:0019538;GO:0005524;GO:0016887;GO:0008233;GO:0008233;GO:0016887;GO:0005524	BP:protein unfolding;BP:protein unfolding;BP:protein metabolic process;MF:ATP binding;MF:ATPase activity;MF:peptidase activity;MF:peptidase activity;MF:ATPase activity;MF:ATP binding;
BAB_RS18645	-	xylose isomerase	1.302	0.380385759	0.011562168	0.043963606	no	up	mRNA	31.29	25.92	25.78	40.3	31.68	29.34	27.6633	33.773333	COG2115	G:Carbohydrate transport and metabolism	GO:0042732;GO:0006098;GO:0042732;GO:0006098;GO:0042732;GO:0006098;GO:0005737;GO:0005737;GO:0005737;GO:0000287;GO:0009045;GO:0016787;GO:0000287;GO:0009045;GO:0000287;GO:0046872;GO:0009045	BP:D-xylose metabolic process;BP:pentose-phosphate shunt;BP:D-xylose metabolic process;BP:pentose-phosphate shunt;BP:D-xylose metabolic process;BP:pentose-phosphate shunt;CC:cytoplasm;CC:cytoplasm;CC:cytoplasm;MF:magnesium ion binding;MF:xylose isomerase activity;MF:hydrolase activity;MF:magnesium ion binding;MF:xylose isomerase activity;MF:magnesium ion binding;MF:metal ion binding;MF:xylose isomerase activity;
BAB_RS27535	-	mononuclear molybdenum enzyme YedY	1.301	0.379206774	2.23596E-05	0.000690781	no	up	mRNA	446.85	390.15	421.74	505.12	520.18	518.88	419.58	514.72667	COG2041	P:Inorganic ion transport and metabolism	GO:0042128;GO:0030091;GO:0042597;GO:0043546;GO:0016672;GO:0016667;GO:0046872	BP:nitrate assimilation;BP:protein repair;CC:periplasmic space;MF:molybdopterin cofactor binding;MF:oxidoreductase activity, acting on a sulfur group of donors, quinone or similar compound as acceptor;MF:oxidoreductase activity, acting on a sulfur group of donors;MF:metal ion binding;
BAB_RS31630	-	membrane protein	1.3	0.378110836	3.84037E-05	0.001028258	no	up	mRNA	172.98	169.19	184.77	201.56	227.86	215.55	175.647	214.99	COG3268	S:Function unknown	GO:0016491	MF:oxidoreductase activity;
BAB_RS22075	-	hypothetical protein	1.3	0.37885517	0.185235747	0.32328216	no	up	mRNA	168.52	247.2	325.47	368.37	347.41	336.62	247.063	350.8	COG4395	S:Function unknown	GO:0016021	CC:integral component of membrane;
BAB_RS26390	-	nucleoside hydrolase	1.299	0.377879286	5.7677E-05	0.001305044	no	up	mRNA	124.2	122.93	127.13	150.58	148.33	160.69	124.753	153.2	COG1957	F:Nucleotide transport and metabolism	GO:0008152;GO:0016799;GO:0008477	BP:metabolic process;MF:hydrolase activity, hydrolyzing N-glycosyl compounds;MF:purine nucleosidase activity;
BAB_RS29445	-	virulence factor family protein	1.298	0.376429656	0.000186075	0.002780743	no	up	mRNA	90.69	75.32	80.58	100.28	101.08	99.69	82.1967	100.35	COG3946	U:Intracellular trafficking, secretion, and vesicular transport	GO:1904979;GO:0031339;GO:0009405;GO:0031334;GO:0005737;GO:0042597	BP:negative regulation of endosome organization;BP:negative regulation of vesicle fusion;BP:pathogenesis;BP:positive regulation of protein complex assembly;CC:cytoplasm;CC:periplasmic space;
BAB_RS26320	-	peptidase P60	1.298	0.376632159	0.000214993	0.003070101	no	up	mRNA	121.27	124.24	131.88	143.39	166.31	153.94	125.797	154.54667	COG0791	S:Function unknown	------	------
BAB_RS20680	-	hypothetical protein	1.298	0.376300328	0.607195305	0.740667621	no	up	mRNA	4.11	14.12	1	10.3	6.98	6.29	6.41	7.8566667	ENOG410Z889	S:Function unknown	------	------
BAB_RS23995	-	deoxyuridine 5'-triphosphate nucleotidohydrolase	1.297	0.37492591	0.000948488	0.008040878	no	up	mRNA	131.25	124.46	124.18	158	170.19	140.57	126.63	156.25333	COG0756	F:Nucleotide transport and metabolism	GO:0006226;GO:0046080;GO:0006226;GO:0046080;GO:0006226;GO:0046080;GO:0004170;GO:0046872;GO:0004170;GO:0000287;GO:0046872;GO:0016491;GO:0004170;GO:0000287	BP:dUMP biosynthetic process;BP:dUTP metabolic process;BP:dUMP biosynthetic process;BP:dUTP metabolic process;BP:dUMP biosynthetic process;BP:dUTP metabolic process;MF:dUTP diphosphatase activity;MF:metal ion binding;MF:dUTP diphosphatase activity;MF:magnesium ion binding;MF:metal ion binding;MF:oxidoreductase activity;MF:dUTP diphosphatase activity;MF:magnesium ion binding;
BAB_RS27910	-	GntR family transcriptional regulator	1.296	0.37450081	0.11484272	0.228193977	no	up	mRNA	97.62	62.78	81.51	133.85	93.25	66.48	80.6367	97.86	COG2188	M:Cell wall/membrane/envelope biogenesis	GO:0006351;GO:0006351;GO:0003700;GO:0003677;GO:0003677;GO:0003700	BP:transcription, DNA-templated;BP:transcription, DNA-templated;MF:transcription factor activity, sequence-specific DNA binding;MF:DNA binding;MF:DNA binding;MF:transcription factor activity, sequence-specific DNA binding;
BAB_RS20050	-	DUF1476 domain-containing protein	1.295	0.372998166	0.000101927	0.001904023	no	up	mRNA	839.01	711.36	730.21	950.08	987.69	933.25	760.193	957.00667	COG5467	S:Function unknown	------	------
BAB_RS20085	-	NADPH:quinone oxidoreductase	1.294	0.372137616	0.000134333	0.002247975	no	up	mRNA	69.83	58.99	69.13	81.83	81.39	77.9	65.9833	80.373333	COG0604	C:Energy production and conversion	GO:0004022;GO:0016491;GO:0016491	MF:alcohol dehydrogenas;MF:oxidoreductase activity;MF:oxidoreductase activity;
BAB_RS26600	-	heat-shock protein IbpA	1.294	0.371527205	0.004339864	0.022673145	no	up	mRNA	256.5	213.24	260.27	351.53	270.49	265.39	243.337	295.80333	COG0071	E:Amino acid transport and metabolism	------	------
BAB_RS21585	-	elongation factor Ts	1.293	0.371224627	0.000272949	0.003480098	no	up	mRNA	327.1	281.63	313.56	406.44	373.65	343.78	307.43	374.62333	COG0264	J:Translation, ribosomal structure and biogenesis	GO:0005737;GO:0005737;GO:0003746;GO:0003746	CC:cytoplasm;CC:cytoplasm;MF:translation elongation factor activity;MF:translation elongation factor activity;
BAB_RS20720	-	IS5/IS1182 family transposase	1.293	0.370321147	0.168280754	0.302905356	no	up	mRNA	50.39	19.4	43.91	47.05	49.87	41.38	37.9	46.1	COG3293	L:Replication, recombination and repair	------	------
BAB_RS20825	-	type I glutamate--ammonia ligase	1.292	0.369875611	0.001688635	0.011667921	no	up	mRNA	340.43	324.99	324.63	365.95	375.59	464.45	330.017	401.99667	COG0174	E:Amino acid transport and metabolism	GO:0009399;GO:0006542;GO:0009399;GO:0006542;GO:0006542;GO:0009399;GO:0005737;GO:0005737;GO:0005737;GO:0004356;GO:0005524;GO:0004356;GO:0016757;GO:0005524;GO:0005524;GO:0004356	BP:nitrogen fixation;BP:glutamine biosynthetic process;BP:nitrogen fixation;BP:glutamine biosynthetic process;BP:glutamine biosynthetic process;BP:nitrogen fixation;CC:cytoplasm;CC:cytoplasm;CC:cytoplasm;MF:glutamate-ammonia ligase activity;MF:ATP binding;MF:glutamate-ammonia ligase activity;MF:transferase activity, transferring glycosyl groups;MF:ATP binding;MF:ATP binding;MF:glutamate-ammonia ligase activity;
BAB_RS30510	-	septum site-determining protein MinD	1.291	0.368448537	2.79007E-05	0.000807613	no	up	mRNA	1056.81	997.65	1109.51	1217.19	1326.03	1319.2	1054.66	1287.4733	COG2894	D:Cell cycle control, cell division, chromosome partitioning	GO:0016887;GO:0005524;GO:0005524;GO:0016887	MF:ATPase activity;MF:ATP binding;MF:ATP binding;MF:ATPase activity;
BAB_RS28805	-	glycine dehydrogenase	1.29	0.367376331	2.32161E-05	0.000703711	no	up	mRNA	305.93	283.62	323.09	376.94	358.86	370.43	304.213	368.74333	COG1003	E:Amino acid transport and metabolism	GO:0019464;GO:0019464;GO:0006546;GO:0004375;GO:0008168;GO:0004375	BP:glycine decarboxylation via glycine cleavage system;BP:glycine decarboxylation via glycine cleavage system;BP:glycine catabolic process;MF:glycine dehydrogenas;MF:methyltransferase activity;MF:glycine dehydrogenas;
BAB_RS21520	-	glutamate--tRNA ligase	1.29	0.367565739	0.001873774	0.012464673	no	up	mRNA	51.78	46.67	48.24	67.56	54.52	56.04	48.8967	59.373333	COG0008	S:Function unknown	GO:0006424;GO:0006424;GO:0006424;GO:0005737;GO:0005737;GO:0005737;GO:0016787;GO:0000049;GO:0004818;GO:0008270;GO:0005524;GO:0000049;GO:0004818;GO:0008270;GO:0005524;GO:0005524;GO:0004818;GO:0016787;GO:0000049;GO:0008270	BP:glutamyl-tRNA aminoacylation;BP:glutamyl-tRNA aminoacylation;BP:glutamyl-tRNA aminoacylation;CC:cytoplasm;CC:cytoplasm;CC:cytoplasm;MF:hydrolase activity;MF:tRNA binding;MF:glutamate-tRNA ligase activity;MF:zinc ion binding;MF:ATP binding;MF:tRNA binding;MF:glutamate-tRNA ligase activity;MF:zinc ion binding;MF:ATP binding;MF:ATP binding;MF:glutamate-tRNA ligase activity;MF:hydrolase activity;MF:tRNA binding;MF:zinc ion binding;
BAB_RS24790	-	hypothetical protein	1.289	0.366739482	0.000238941	0.003239319	no	up	mRNA	64.25	63.12	66.92	74.94	79.83	82.61	64.7633	79.126667	ENOG410YSMN	S:Function unknown	------	------
BAB_RS30375	-	IMP dehydrogenase	1.289	0.366320953	0.000353375	0.004158952	no	up	mRNA	166.38	148.62	159.07	211.15	178.85	185.07	158.023	191.69	COG0516	F:Nucleotide transport and metabolism	GO:0006177;GO:0006177;GO:0046872;GO:0003938;GO:0000166;GO:0003938;GO:0046872;GO:0000166	BP:GMP biosynthetic process;BP:GMP biosynthetic process;MF:metal ion binding;MF:IMP dehydrogenase activity;MF:nucleotide binding;MF:IMP dehydrogenase activity;MF:metal ion binding;MF:nucleotide binding;
BAB_RS25550	-	hypothetical protein	1.288	0.365072268	0.022003613	0.068906051	no	up	mRNA	11.29	12.67	12.48	16.14	15.81	12.5	12.1467	14.816667	ENOG4111GVA	S:Function unknown	------	------
BAB_RS18455	-	5-hydroxyisourate hydrolase	1.288	0.365044573	0.029249635	0.083314784	no	up	mRNA	49.24	39.63	45.18	55.78	49.17	62.33	44.6833	55.76	COG2351	F:Nucleotide transport and metabolism	GO:0006144;GO:0033971	BP:purine nucleobase metabolic process;MF:hydroxyisourate hydrolase activity;
BAB_RS21995	-	-	1.286	0.363132882	0.538397457	0.688368893	no	up	mRNA	194.37	50.89	163.8	215.93	109.05	248.48	136.353	191.15333	------	------	------	------
BAB_RS16360	-	aconitate hydratase AcnA	1.285	0.362182951	6.64886E-05	0.001433744	no	up	mRNA	569.35	539.54	580.67	659.71	667.52	716.12	563.187	681.11667	COG1048	C:Energy production and conversion	GO:0008152;GO:0003994;GO:0004180;GO:0051539	BP:metabolic process;MF:aconitate hydratase activity;MF:carboxypeptidase activity;MF:4 iron, 4 sulfur cluster binding;
BAB_RS29200	-	IclR family transcriptional regulator	1.283	0.359048843	0.046967093	0.118823048	no	up	mRNA	229.92	174.29	192.62	309.31	224.87	185.29	198.943	239.82333	COG1414	K:Transcription	GO:0046278;GO:0045893;GO:0006351;GO:0046278;GO:0006351;GO:0045893;GO:0003677;GO:0003677	BP:3,4-dihydroxybenzoate metabolic process;BP:positive regulation of transcription, DNA-templated;BP:transcription, DNA-templated;BP:3,4-dihydroxybenzoate metabolic process;BP:transcription, DNA-templated;BP:positive regulation of transcription, DNA-templated;MF:DNA binding;MF:DNA binding;
BAB_RS16955	-	copper-translocating P-type ATPase	1.282	0.358322164	4.35067E-05	0.001118297	no	up	mRNA	152.34	147.5	169.97	196.7	185.44	183.56	156.603	188.56667	COG2217	P:Inorganic ion transport and metabolism	GO:0030001;GO:0030001;GO:0016021;GO:0005886;GO:0016021;GO:0005886;GO:0019829;GO:0047381;GO:0046872;GO:0000166;GO:0019829;GO:0000166;GO:0046872	BP:metal ion transport;BP:metal ion transport;CC:integral component of membrane;CC:plasma membrane;CC:integral component of membrane;CC:plasma membrane;MF:cation-transporting ATPase activity;MF:dodecanoyl-[acyl-carrier-protein] hydrolase activity;MF:metal ion binding;MF:nucleotide binding;MF:cation-transporting ATPase activity;MF:nucleotide binding;MF:metal ion binding;
BAB_RS23025	-	hypothetical protein	1.282	0.358802148	0.411433009	0.575004027	no	up	mRNA	3.47	3.67	4.47	5.82	2.62	6.62	3.87	5.02	------	------	------	------
BAB_RS26450	-	molybdopterin oxidoreductase	1.281	0.356939303	0.056917631	0.13719156	no	up	mRNA	5.9	6.54	6.93	6.72	8.23	8.72	6.45667	7.89	COG2041	S:Function unknown	GO:0042128;GO:0042128;GO:0009703	BP:nitrate assimilation;BP:nitrate assimilation;MF:nitrate reductas;
BAB_RS19630	-	hypothetical protein	1.28	0.355631305	0.000311106	0.003859393	no	up	mRNA	163.25	139.31	142.6	181.34	172.51	182.34	148.387	178.73	ENOG4111TM6;ENOG4111YTU	S:Function unknown;	------	------
BAB_RS24090	-	protein TolB	1.278	0.353668069	1.96451E-05	0.00064408	no	up	mRNA	348.43	319.74	345.96	399.87	405.57	415.99	338.043	407.14333	COG0823	U:Intracellular trafficking, secretion, and vesicular transport	GO:0017038;GO:0051301;GO:0051301;GO:0017038;GO:0017038;GO:0007049;GO:0051301;GO:0042597;GO:0042597;GO:0042597	BP:protein import;BP:cell division;BP:cell division;BP:protein import;BP:protein import;BP:cell cycle;BP:cell division;CC:periplasmic space;CC:periplasmic space;CC:periplasmic space;
BAB_RS21055	-	ATP-dependent helicase	1.276	0.351184936	0.000335471	0.004067429	no	up	mRNA	2142.68	1933.05	2056.55	2613.8	2396.46	2358.93	2044.09	2456.3967	COG0513	E:Amino acid transport and metabolism	GO:0005524;GO:0004386;GO:0003676;GO:0003676;GO:0005524;GO:0004386	MF:ATP binding;MF:helicase activity;MF:nucleic acid binding;MF:nucleic acid binding;MF:ATP binding;MF:helicase activity;
BAB_RS27450	-	hypothetical protein	1.275	0.350132519	0.037884035	0.101434503	no	up	mRNA	3.86	4.68	5.94	6.19	5.99	5.4	4.82667	5.86	COG0369	P:Inorganic ion transport and metabolism	GO:0016021;GO:0010181;GO:0016491	CC:integral component of membrane;MF:FMN binding;MF:oxidoreductase activity;
BAB_RS31255	-	acetoin dehydrogenase dihydrolipoyllysine-residue acetyltransferase subunit	1.274	0.348897012	0.001071151	0.008690928	no	up	mRNA	78.38	74.58	81.24	103.91	93.15	83.9	78.0667	93.653333	COG0596	S:Function unknown	GO:0008152;GO:0008152;GO:0016787;GO:0004742;GO:0016746;GO:0016746	BP:metabolic process;BP:metabolic process;MF:hydrolase activity;MF:dihydrolipoyllysine-residue acetyltransferase activity;MF:transferase activity, transferring acyl groups;MF:transferase activity, transferring acyl groups;
BAB_RS19435	-	hypothetical protein	1.273	0.348592606	0.000468866	0.005021552	no	up	mRNA	107.23	108.9	104.95	129.12	121.66	136.09	107.027	128.95667	------	------	------	------
BAB_RS33025	-	hypothetical protein	1.273	0.34865209	0.007782895	0.033565695	no	up	mRNA	69.78	59.86	88.49	95.38	88.49	76.05	72.71	86.64	------	------	GO:0016021	CC:integral component of membrane;
BAB_RS19315	-	cytosol aminopeptidase	1.272	0.346724493	0.00012883	0.002178591	no	up	mRNA	228.93	198.4	211.78	243.19	262.58	259.48	213.037	255.08333	COG0260	E:Amino acid transport and metabolism	GO:0005737;GO:0005737;GO:0004177;GO:0030145;GO:0008235;GO:0004177;GO:0008235;GO:0030145	CC:cytoplasm;CC:cytoplasm;MF:aminopeptidase activity;MF:manganese ion binding;MF:metalloexopeptidase activity;MF:aminopeptidase activity;MF:metalloexopeptidase activity;MF:manganese ion binding;
BAB_RS30325	-	hypothetical protein	1.272	0.347155866	0.001181895	0.009376365	no	up	mRNA	953.43	958.97	922.91	1023.18	1282.35	1120.29	945.103	1141.94	COG3470	P:Inorganic ion transport and metabolism	------	------
BAB_RS26425	-	2%2C3-dihydro-2%2C3-dihydroxybenzoate dehydrogenase	1.272	0.346691637	0.003621997	0.02003008	no	up	mRNA	25.96	27.08	29.63	33.49	31.76	34.79	27.5567	33.346667	COG1028	Q:Secondary metabolites biosynthesis, transport and catabolism	GO:0019290;GO:0019290;GO:0008667;GO:0008667	BP:siderophore biosynthetic process;BP:siderophore biosynthetic process;MF:2,3-dihydro-2,3-dihydroxybenzoate dehydrogenase activity;MF:2,3-dihydro-2,3-dihydroxybenzoate dehydrogenase activity;
BAB_RS31130	-	universal stress protein	1.272	0.347434976	0.081197146	0.178567031	no	up	mRNA	26.72	29.92	37.24	29.76	45.45	40.43	31.2933	38.546667	COG0589	G:Carbohydrate transport and metabolism	GO:0006950	BP:response to stress;
BAB_RS17880	-	cell wall hydrolase	1.271	0.346165963	0.000500708	0.005257436	no	up	mRNA	73.08	70.83	75.42	86.61	80.66	95.66	73.11	87.643333	COG3773	M:Cell wall/membrane/envelope biogenesis	GO:0016787	MF:hydrolase activity;
BAB_RS31950	-	cytochrome aa3 quinol oxidase subunit I	1.271	0.346159987	0.000769315	0.006828199	no	up	mRNA	288.37	231.23	243.21	312.58	304.73	295.92	254.27	304.41	COG0843	C:Energy production and conversion	GO:0009060;GO:0016021;GO:0070469;GO:0004129;GO:0020037;GO:0016682	BP:aerobic respiration;CC:integral component of membrane;CC:respiratory chain;MF:cytochrome-c oxidase activity;MF:heme binding;MF:oxidoreductase activity, acting on diphenols and related substances as donors, oxygen as acceptor;
BAB_RS16130	-	cytochrome o ubiquinol oxidase subunit III	1.271	0.345448472	0.003994593	0.021391048	no	up	mRNA	46.57	43.91	42.63	57.7	50.04	52.58	44.37	53.44	COG1845	C:Energy production and conversion	GO:0019646;GO:0016021;GO:0005886;GO:0004129;GO:0004565;GO:0008827	BP:aerobic electron transport chain;CC:integral component of membrane;CC:plasma membrane;MF:cytochrome-c oxidase activity;MF:beta-galactosidase activity;MF:cytochrome o ubiquinol oxidase activity;
BAB_RS28215	-	IS5/IS1182 family transposase	1.271	0.345792004	0.627119551	0.752402956	no	up	mRNA	6.58	10.56	0	7.9	5.64	6.17	5.71333	6.57	COG3293	L:Replication, recombination and repair	GO:0006313;GO:0016021;GO:0003677;GO:0004803	BP:transposition, DNA-mediated;CC:integral component of membrane;MF:DNA binding;MF:transposase activity;
BAB_RS21465	-	CTP synthetase	1.27	0.344753022	0.000253703	0.003286882	no	up	mRNA	267.26	244.04	239.77	308.73	294.62	295.92	250.357	299.75667	COG0504	F:Nucleotide transport and metabolism	GO:0044210;GO:0006541;GO:0044210;GO:0006541;GO:0003883;GO:0046872;GO:0005524;GO:0016491;GO:0016740;GO:0003883;GO:0046872;GO:0005524	BP:'de novo' CTP biosynthetic process;BP:glutamine metabolic process;BP:'de novo' CTP biosynthetic process;BP:glutamine metabolic process;MF:CTP synthase activity;MF:metal ion binding;MF:ATP binding;MF:oxidoreductase activity;MF:transferase activity;MF:CTP synthase activity;MF:metal ion binding;MF:ATP binding;
BAB_RS16510	-	DNA topoisomerase (ATP-hydrolyzing) subunit B	1.27	0.34426481	0.000253278	0.003294665	no	up	mRNA	285.1	255.34	279.62	343.82	331.87	302.74	273.353	326.14333	COG0187	L:Replication, recombination and repair	GO:0006265;GO:0006261;GO:0006265;GO:0006261;GO:0005694;GO:0005737;GO:0005737;GO:0005694;GO:0005524;GO:0003677;GO:0003918;GO:0046872;GO:0003677;GO:0046872;GO:0003918;GO:0005524	BP:DNA topological change;BP:DNA-dependent DNA replication;BP:DNA topological change;BP:DNA-dependent DNA replication;CC:chromosome;CC:cytoplasm;CC:cytoplasm;CC:chromosome;MF:ATP binding;MF:DNA binding;MF:DNA topoisomerase type I;MF:metal ion binding;MF:DNA binding;MF:metal ion binding;MF:DNA topoisomerase type I;MF:ATP binding;
BAB_RS17125	-	hypothetical protein	1.27	0.345351269	0.077838909	0.173077104	no	up	mRNA	5.63	4.46	5.61	6.62	5.41	6.84	5.23333	6.29	ENOG4111KAY	S:Function unknown	------	------
BAB_RS24045	-	phosphoserine transaminase	1.269	0.343449432	0.000550778	0.005653833	no	up	mRNA	272.3	254.73	263.22	281.55	323.32	341.5	263.417	315.45667	COG1932	E:Amino acid transport and metabolism	GO:0006564;GO:0008643;GO:0006564;GO:0005737;GO:0005737;GO:0004648;GO:0004648	BP:L-serine biosynthetic process;BP:carbohydrate transport;BP:L-serine biosynthetic process;CC:cytoplasm;CC:cytoplasm;MF:O-phospho-L-serin;MF:O-phospho-L-serin;
BAB_RS28445	-	nickel import ATP-binding protein NikE	1.269	0.344223653	0.064112268	0.149270086	no	up	mRNA	9.39	13.36	11.24	13.58	15.29	12.33	11.33	13.733333	COG1124;COG1124	E:Amino acid transport and metabolism;P:Inorganic ion transport and metabolism	GO:0008152;GO:0005886;GO:0005886;GO:0016151;GO:0016798;GO:0015413;GO:0005524;GO:0005524;GO:0016151;GO:0015413	BP:metabolic process;CC:plasma membrane;CC:plasma membrane;MF:nickel cation binding;MF:hydrolase activity, acting on glycosyl bonds;MF:nickel-transporting ATPase activity;MF:ATP binding;MF:ATP binding;MF:nickel cation binding;MF:nickel-transporting ATPase activity;
BAB_RS24555	-	leucine--tRNA ligase	1.268	0.342693912	0.000661735	0.006327843	no	up	mRNA	77.5	71.65	81.68	102.45	85.7	86.99	76.9433	91.713333	COG0495	J:Translation, ribosomal structure and biogenesis	GO:0006429;GO:0006429;GO:0006429;GO:0005737;GO:0005737;GO:0005737;GO:0016853;GO:0004823;GO:0002161;GO:0005524;GO:0005524;GO:0002161;GO:0004823;GO:0002161;GO:0005524;GO:0004823	BP:leucyl-tRNA aminoacylation;BP:leucyl-tRNA aminoacylation;BP:leucyl-tRNA aminoacylation;CC:cytoplasm;CC:cytoplasm;CC:cytoplasm;MF:isomerase activity;MF:leucine-tRNA ligase activity;MF:aminoacyl-tRNA editing activity;MF:ATP binding;MF:ATP binding;MF:aminoacyl-tRNA editing activity;MF:leucine-tRNA ligase activity;MF:aminoacyl-tRNA editing activity;MF:ATP binding;MF:leucine-tRNA ligase activity;
BAB_RS31670	-	FAA hydrolase family protein	1.268	0.343078316	0.236458026	0.387820132	no	up	mRNA	1.8	2.1	2.96	2.18	3.26	2.96	2.28667	2.8	COG0179	Q:Secondary metabolites biosynthesis, transport and catabolism	GO:0008152;GO:0008152;GO:0016787;GO:0016853;GO:0016853;GO:0016829	BP:metabolic process;BP:metabolic process;MF:hydrolase activity;MF:isomerase activity;MF:isomerase activity;MF:lyase activity;
BAB_RS21390	-	tRNA dihydrouridine synthase DusB	1.267	0.341006368	0.001137019	0.009179	no	up	mRNA	102.65	91.99	119.5	128.76	130.38	115.53	104.713	124.89	COG0042	T:Signal transduction mechanisms	GO:0017150;GO:0050660;GO:0050660;GO:0017150	MF:tRNA dihydrouridine synthase activity;MF:flavin adenine dinucleotide binding;MF:flavin adenine dinucleotide binding;MF:tRNA dihydrouridine synthase activity;
BAB_RS19215	-	HD family hydrolase	1.267	0.341545799	0.087639301	0.187598318	no	up	mRNA	10.59	14.07	12	16.35	15.87	12.21	12.22	14.81	COG1896	S:Function unknown	GO:0016787	MF:hydrolase activity;
BAB_RS23125	-	glutathione S-transferase family protein	1.266	0.339732381	0.000735594	0.006714386	no	up	mRNA	121.13	98.92	125.67	144.62	130.16	136.8	115.24	137.19333	COG0625	S:Function unknown	GO:0016740;GO:0004364	MF:transferase activity;MF:glutathione transferase activity;
BAB_RS21400	-	CinA family protein	1.266	0.34037897	0.011643633	0.044064772	no	up	mRNA	34.4	29.37	28.31	37.88	35.08	37.19	30.6933	36.716667	COG1546	S:Function unknown	------	------
BAB_RS33170	-	hypothetical protein	1.266	0.340649628	0.019615785	0.06372651	no	up	mRNA	21.48	15.29	25.25	23.71	26.59	23.11	20.6733	24.47	------	------	------	------
BAB_RS16320	-	arylesterase	1.264	0.337744668	9.26365E-05	0.001782283	no	up	mRNA	273.56	243.12	284.09	303.51	324.5	328.32	266.923	318.77667	COG2755	E:Amino acid transport and metabolism	GO:0016787	MF:hydrolase activity;
BAB_RS28880	-	peroxiredoxin	1.262	0.336201881	0.000162109	0.002540767	no	up	mRNA	2460.33	2219.13	2267.14	2601.85	2841.49	2880.33	2315.53	2774.5567	COG0450	O:Posttranslational modification, protein turnover, chaperones	GO:0045454;GO:0045454;GO:0005623;GO:0005623;GO:0004601;GO:0016209;GO:0016491;GO:0016209;GO:0016491	BP:cell redox homeostasis;BP:cell redox homeostasis;CC:cell;CC:cell;MF:peroxidase activity;MF:antioxidant activity;MF:oxidoreductase activity;MF:antioxidant activity;MF:oxidoreductase activity;
BAB_RS33440	-	hypothetical protein	1.262	0.336282252	0.007209657	0.031863311	no	up	mRNA	12.48	11.12	16.13	16.91	15.28	14.95	13.2433	15.713333	------	------	------	------
BAB_RS29945	-	DUF488 domain-containing protein	1.261	0.334248856	0.00012128	0.002117785	no	up	mRNA	121.64	112.53	123.31	133.91	150.05	140.51	119.16	141.49	COG3189	S:Function unknown	GO:0008168;GO:0004851	MF:methyltransferase activity;MF:uroporphyrin-III C-methyltransferase activity;
BAB_RS27545	-	WD40 repeat domain-containing protein	1.26	0.333244371	0.000417809	0.004629031	no	up	mRNA	83.05	79.7	90.55	107.16	100.58	92.86	84.4333	100.2	COG2319	S:Function unknown	------	------
BAB_RS29095	-	nucleoside deaminase	1.259	0.332442712	0.00240176	0.015250704	no	up	mRNA	28.02	25.4	30.66	34.93	33.45	31.57	28.0267	33.316667	COG0590	S:Function unknown	GO:0003824	MF:catalytic activity;
BAB_RS29800	-	cytochrome bd-I oxidase subunit CydX	1.259	0.332140106	0.020701083	0.066313639	no	up	mRNA	86.65	105.04	127.43	127.29	117.27	140.3	106.373	128.28667	ENOG410ZYFM	S:Function unknown	GO:0006119;GO:0009405;GO:0016021;GO:0016021;GO:0005886;GO:0016491	BP:oxidative phosphorylation;BP:pathogenesis;CC:integral component of membrane;CC:integral component of membrane;CC:plasma membrane;MF:oxidoreductase activity;
BAB_RS20265	-	LysM peptidoglycan-binding domain-containing protein	1.258	0.331667765	0.000148335	0.002419297	no	up	mRNA	425.53	420.19	455.46	522.41	481.91	539.65	433.727	514.65667	COG0739	O:Posttranslational modification, protein turnover, chaperones	------	------
BAB_RS27750	-	hypothetical protein	1.258	0.33151584	0.000720335	0.006593839	no	up	mRNA	234.51	230.32	249.8	250.39	292.03	303.75	238.21	282.05667	COG0028	E:Amino acid transport and metabolism	GO:0003824;GO:0003984;GO:0000287;GO:0030976;GO:0003824;GO:0000287;GO:0030976	MF:catalytic activity;MF:acetolactate synthase activity;MF:magnesium ion binding;MF:thiamine pyrophosphate binding;MF:catalytic activity;MF:magnesium ion binding;MF:thiamine pyrophosphate binding;
BAB_RS29315	-	DUF3572 domain-containing protein	1.258	0.330700987	0.054615108	0.132737022	no	up	mRNA	25.07	17.99	17.19	23.99	25.24	21.26	20.0833	23.496667	ENOG4112C9H	S:Function unknown	------	------
BAB_RS31875	-	oxygen-independent coproporphyrinogen III oxidase	1.258	0.330630954	0.174677858	0.310248732	no	up	mRNA	9.84	15.93	11.66	10.7	19.32	14.75	12.4767	14.923333	COG0635	E:Amino acid transport and metabolism	GO:0006782;GO:0006782;GO:0006782;GO:0005737;GO:0005737;GO:0005737;GO:0051539;GO:0004109;GO:0016853;GO:0046872;GO:0051539;GO:0004109;GO:0046872;GO:0004109;GO:0046872;GO:0051539	BP:protoporphyrinogen IX biosynthetic process;BP:protoporphyrinogen IX biosynthetic process;BP:protoporphyrinogen IX biosynthetic process;CC:cytoplasm;CC:cytoplasm;CC:cytoplasm;MF:4 iron, 4 sulfur cluster binding;MF:coproporphyrinogen oxidase activity;MF:isomerase activity;MF:metal ion binding;MF:4 iron, 4 sulfur cluster binding;MF:coproporphyrinogen oxidase activity;MF:metal ion binding;MF:coproporphyrinogen oxidase activity;MF:metal ion binding;MF:4 iron, 4 sulfur cluster binding;
BAB_RS19675	glyA	serine hydroxymethyltransferase	1.257	0.329431584	0.000831995	0.007224864	no	up	mRNA	101.56	86.84	90.35	116.08	104.52	109.03	92.9167	109.87667	COG0112	E:Amino acid transport and metabolism	GO:0035999;GO:0019264;GO:0019264;GO:0035999;GO:0005737;GO:0005737;GO:0004372;GO:0030170;GO:0008168;GO:0004372;GO:0008168;GO:0030170	BP:tetrahydrofolate interconversion;BP:glycine biosynthetic process from serine;BP:glycine biosynthetic process from serine;BP:tetrahydrofolate interconversion;CC:cytoplasm;CC:cytoplasm;MF:glycine hydroxymethyltransferase activity;MF:pyridoxal phosphate binding;MF:methyltransferase activity;MF:glycine hydroxymethyltransferase activity;MF:methyltransferase activity;MF:pyridoxal phosphate binding;
BAB_RS23625	-	gfo/Idh/MocA family oxidoreductase	1.257	0.329541113	0.00392048	0.021135073	no	up	mRNA	80.18	69.55	74.4	100.35	84.81	80.04	74.71	88.4	COG0673	S:Function unknown	GO:0016491	MF:oxidoreductase activity;
BAB_RS20830	-	nitrogen regulatory protein P-II 1	1.256	0.328921356	0.041916365	0.108874114	no	up	mRNA	486.89	429.93	413.93	440.6	463.08	685.2	443.583	529.62667	COG0347	E:Amino acid transport and metabolism	GO:0006808;GO:0006355;GO:0006351;GO:0006355;GO:0006808;GO:0006351;GO:0030234;GO:0030234	BP:regulation of nitrogen utilization;BP:regulation of transcription, DNA-templated;BP:transcription, DNA-templated;BP:regulation of transcription, DNA-templated;BP:regulation of nitrogen utilization;BP:transcription, DNA-templated;MF:enzyme regulator activity;MF:enzyme regulator activity;
BAB_RS16680	-	DUF1150 domain-containing protein	1.255	0.327172093	0.003037715	0.017875785	no	up	mRNA	1831.61	1803.78	1980.11	2399.76	2261.03	2257.42	1871.83	2306.07	COG5568	S:Function unknown	------	------
BAB_RS30505	-	cell division topological specificity factor	1.254	0.32649559	7.31192E-05	0.001515691	no	up	mRNA	1080.25	1024.79	1073.92	1209.6	1333.19	1245.07	1059.65	1262.62	COG0851	D:Cell cycle control, cell division, chromosome partitioning	GO:0032955;GO:0051301;GO:0007049;GO:0007049;GO:0051301;GO:0032955	BP:regulation of barrier septum assembly;BP:cell division;BP:cell cycle;BP:cell cycle;BP:cell division;BP:regulation of barrier septum assembly;
BAB_RS32630	-	hypothetical protein	1.254	0.326216598	0.033653089	0.092574806	no	up	mRNA	621.65	473.36	753.81	673.73	775.3	782.2	616.273	743.74333	------	------	------	------
BAB_RS25900	-	DNA-binding response regulator	1.253	0.325309358	2.92917E-05	0.000840306	no	up	mRNA	421.59	395.76	429.08	493.86	484.79	496.7	415.477	491.78333	COG0745	C:Energy production and conversion	GO:0000160;GO:0006355;GO:0006351;GO:0000160;GO:0006355;GO:0006351;GO:0005622;GO:0005622;GO:0003677;GO:0003677	BP:phosphorelay signal transduction system;BP:regulation of transcription, DNA-templated;BP:transcription, DNA-templated;BP:phosphorelay signal transduction system;BP:regulation of transcription, DNA-templated;BP:transcription, DNA-templated;CC:intracellular;CC:intracellular;MF:DNA binding;MF:DNA binding;
BAB_RS17495	-	polyphosphate kinase 2	1.253	0.325710716	6.35962E-05	0.001409203	no	up	mRNA	148.82	143.29	154.41	166.95	181.43	178.6	148.84	175.66	COG2326	S:Function unknown	GO:0008976	MF:polyphosphate kinase activity;
BAB_RS27005	-	glycosyltransferase family 39 protein	1.252	0.323806829	0.000509022	0.005327325	no	up	mRNA	65.52	59.24	64.74	75.98	69.14	78.32	63.1667	74.48	COG1807	M:Cell wall/membrane/envelope biogenesis	GO:0006493;GO:0016021;GO:0000030	BP:protein O-linked glycosylation;CC:integral component of membrane;MF:mannosyltransferase activity;
BAB_RS29725	-	DNA translocase FtsK	1.251	0.322532154	0.000246877	0.003291354	no	up	mRNA	280.33	275.7	296.07	325.64	317.66	359.75	284.033	334.35	COG1674;COG1674	E:Amino acid transport and metabolism;Q:Secondary metabolites biosynthesis, transport and catabolism	GO:0051301;GO:0005524;GO:0003677	BP:cell division;MF:ATP binding;MF:DNA binding;
BAB_RS27140	-	CoA ester lyase	1.251	0.322646537	0.000736452	0.006703175	no	up	mRNA	202.59	184.58	209.46	211.27	241.8	252.32	198.877	235.13	COG2301	G:Carbohydrate transport and metabolism	GO:0008815;GO:0003824;GO:0016829;GO:0046872;GO:0016829;GO:0046872	MF:citrat;MF:catalytic activity;MF:lyase activity;MF:metal ion binding;MF:lyase activity;MF:metal ion binding;
BAB_RS17810	-	transcriptional repressor	1.251	0.322577771	0.079723465	0.176170215	no	up	mRNA	23.53	17.57	21.84	18.35	29.14	26.29	20.98	24.593333	COG0735	K:Transcription	GO:0006351;GO:0006351;GO:0005737;GO:0005737;GO:0003677;GO:0003700;GO:0046872;GO:0003700;GO:0046872;GO:0003677;GO:0003677;GO:0003700	BP:transcription, DNA-templated;BP:transcription, DNA-templated;CC:cytoplasm;CC:cytoplasm;MF:DNA binding;MF:transcription factor activity, sequence-specific DNA binding;MF:metal ion binding;MF:transcription factor activity, sequence-specific DNA binding;MF:metal ion binding;MF:DNA binding;MF:DNA binding;MF:transcription factor activity, sequence-specific DNA binding;
BAB_RS16800	-	BA14K family protein	1.25	0.322430338	0.00016312	0.002544205	no	up	mRNA	108.18	98.94	107.75	121.05	128	122.76	104.957	123.93667	ENOG410Z10D;ENOG410YUXB	S:Function unknown;	GO:0009405;GO:0009405;GO:0016021	BP:pathogenesis;BP:pathogenesis;CC:integral component of membrane;
BAB_RS29935	-	MarR family transcriptional regulator	1.25	0.321628908	0.119039861	0.234359726	no	up	mRNA	16.51	16.31	15.18	14.48	23.13	19.18	16	18.93	ENOG41122MC	E:Amino acid transport and metabolism	GO:0006351;GO:0006351;GO:0003700;GO:0003677;GO:0003677;GO:0003700	BP:transcription, DNA-templated;BP:transcription, DNA-templated;MF:transcription factor activity, sequence-specific DNA binding;MF:DNA binding;MF:DNA binding;MF:transcription factor activity, sequence-specific DNA binding;
BAB_RS24105	-	protein TolQ	1.249	0.320505756	6.57178E-05	0.001426697	no	up	mRNA	431.66	384.91	415.6	496.28	474.02	485.42	410.723	485.24	COG0811	S:Function unknown	GO:0015031;GO:0016021	BP:protein transport;CC:integral component of membrane;
BAB_RS23225	-	MBL fold metallo-hydrolase	1.249	0.320543497	0.000251217	0.003294526	no	up	mRNA	199.32	184.91	188.2	229.45	230.92	217.05	190.81	225.80667	COG0491	S:Function unknown	GO:0016787;GO:0008800	MF:hydrolase activity;MF:beta-lactamase activity;
BAB_RS23270	-	hypothetical protein	1.249	0.320219997	0.000380524	0.004350976	no	up	mRNA	600.94	538.51	491.96	629.5	696.16	649.13	543.803	658.26333	COG2960	S:Function unknown	------	------
BAB_RS21930	-	elongation factor Tu	1.249	0.320500025	0.00052889	0.005481689	no	up	mRNA	1497.44	1234.22	1522.88	1730.08	1633.1	1636.45	1418.18	1666.5433	COG0050	J:Translation, ribosomal structure and biogenesis	GO:0005737;GO:0005737;GO:0005525;GO:0003924;GO:0003746;GO:0003746;GO:0003743;GO:0003924;GO:0005525	CC:cytoplasm;CC:cytoplasm;MF:GTP binding;MF:GTPase activity;MF:translation elongation factor activity;MF:translation elongation factor activity;MF:translation initiation factor activity;MF:GTPase activity;MF:GTP binding;
BAB_RS21620	-	HIT family protein	1.249	0.321308604	0.001198284	0.009459671	no	up	mRNA	55.6	53.93	69.92	68.04	74.57	67.79	59.8167	70.133333	COG0537	S:Function unknown	GO:0003824;GO:0004081	MF:catalytic activity;MF:bi;
BAB_RS17185	-	hypothetical protein	1.249	0.321113589	0.010135524	0.039811049	no	up	mRNA	40.39	33.89	37.44	40.43	43.72	48.07	37.24	44.073333	ENOG410YZCT	S:Function unknown	GO:0003677	MF:DNA binding;
BAB_RS20865	-	3-deoxy-7-phosphoheptulonate synthase class II	1.248	0.31977728	9.31004E-05	0.001780544	no	up	mRNA	230.5	232.22	245	283.42	268.29	281.36	235.907	277.69	COG3200	E:Amino acid transport and metabolism	GO:0009073;GO:0009073;GO:0009073;GO:0003849;GO:0004812;GO:0016746;GO:0003849;GO:0004812;GO:0003849	BP:aromatic amino acid family biosynthetic process;BP:aromatic amino acid family biosynthetic process;BP:aromatic amino acid family biosynthetic process;MF:3-deoxy-7-phosphoheptulonate synthase activity;MF:aminoacyl-tRNA ligase activity;MF:transferase activity, transferring acyl groups;MF:3-deoxy-7-phosphoheptulonate synthase activity;MF:aminoacyl-tRNA ligase activity;MF:3-deoxy-7-phosphoheptulonate synthase activity;
BAB_RS23190	-	extensin	1.248	0.319405338	0.000248769	0.003275798	no	up	mRNA	74.18	75.18	84.9	92.05	89.96	93.7	78.0867	91.903333	COG3921	S:Function unknown	------	------
BAB_RS21940	-	30S ribosomal protein S7	1.248	0.319386881	0.002208109	0.014217747	no	up	mRNA	1032.32	892.79	921.21	1266.64	1050.82	1062.7	948.773	1126.72	COG0049	J:Translation, ribosomal structure and biogenesis	GO:0006412;GO:0006412;GO:0015935;GO:0015935;GO:0003735;GO:0000049;GO:0019843;GO:0003735;GO:0019843;GO:0000049	BP:translation;BP:translation;CC:small ribosomal subunit;CC:small ribosomal subunit;MF:structural constituent of ribosome;MF:tRNA binding;MF:rRNA binding;MF:structural constituent of ribosome;MF:rRNA binding;MF:tRNA binding;
BAB_RS27135	-	hypothetical protein	1.247	0.318350978	0.000788961	0.006964102	no	up	mRNA	365.06	326.6	345.3	364.25	424.06	433.26	345.653	407.19	COG3777	S:Function unknown	------	------
BAB_RS28930	-	iron ABC transporter permease	1.247	0.318805705	0.009860119	0.039208619	no	up	mRNA	15.87	19.59	17.05	20.42	21.32	20.27	17.5033	20.67	COG1178	S:Function unknown	GO:0055085;GO:0016021;GO:0005886	BP:transmembrane transport;CC:integral component of membrane;CC:plasma membrane;
BAB_RS25620	-	GTP-binding protein	1.246	0.317389272	0.000138329	0.002302855	no	up	mRNA	123.71	116.74	123.31	143.76	146.83	136.62	121.253	142.40333	COG0523	O:Posttranslational modification, protein turnover, chaperones	------	------
BAB_RS20055	-	2%2C4-dihydroxyhept-2-ene-1%2C7-dioic acid aldolase	1.246	0.317155622	0.000297589	0.003734972	no	up	mRNA	66.9	63.67	69.95	82.59	77.66	75.14	66.84	78.463333	COG3836	G:Carbohydrate transport and metabolism	GO:0003824;GO:0046872	MF:catalytic activity;MF:metal ion binding;
BAB_RS31010	-	succinyl-diaminopimelate desuccinylase	1.245	0.31639656	0.000558709	0.005698829	no	up	mRNA	171.74	159.55	158.57	178.79	196.35	200.37	163.287	191.83667	COG0624	E:Amino acid transport and metabolism	GO:0019877;GO:0009089;GO:0019877;GO:0009089;GO:0050897;GO:0009014;GO:0008270;GO:0046872;GO:0008237;GO:0050897;GO:0016805;GO:0009014;GO:0008270;GO:0046872;GO:0008237	BP:diaminopimelate biosynthetic process;BP:lysine biosynthetic process via diaminopimelate;BP:diaminopimelate biosynthetic process;BP:lysine biosynthetic process via diaminopimelate;MF:cobalt ion binding;MF:succinyl-diaminopimelate desuccinylase activity;MF:zinc ion binding;MF:metal ion binding;MF:metallopeptidase activity;MF:cobalt ion binding;MF:dipeptidase activity;MF:succinyl-diaminopimelate desuccinylase activity;MF:zinc ion binding;MF:metal ion binding;MF:metallopeptidase activity;
BAB_RS28005	-	thioredoxin	1.243	0.313366199	0.000242609	0.003275221	no	up	mRNA	485.69	426.63	463.18	537.19	565.54	509.48	458.5	537.40333	COG3118	O:Posttranslational modification, protein turnover, chaperones	GO:0045454;GO:0006662;GO:0045454;GO:0006662;GO:0005623;GO:0005623;GO:0015035;GO:0015035	BP:cell redox homeostasis;BP:glycerol ether metabolic process;BP:cell redox homeostasis;BP:glycerol ether metabolic process;CC:cell;CC:cell;MF:protein disulfide oxidoreductase activity;MF:protein disulfide oxidoreductase activity;
BAB_RS27860	-	D-amino acid dehydrogenase small subunit	1.243	0.31415967	0.000831905	0.007243662	no	up	mRNA	84.92	74.8	90.97	88.74	103.32	100.87	83.5633	97.643333	COG0665	K:Transcription	GO:0019478;GO:0055130;GO:0019478;GO:0008718;GO:0008718	BP:D-amino acid catabolic process;BP:D-alanine catabolic process;BP:D-amino acid catabolic process;MF:D-amino-acid dehydrogenase activity;MF:D-amino-acid dehydrogenase activity;
BAB_RS31775	-	glycerol-3-phosphate ABC transporter substrate-binding protein	1.243	0.314281621	0.064447385	0.149616654	no	up	mRNA	14.1	8.3	11.55	12.73	12.68	13.83	11.3167	13.08	COG1653	S:Function unknown	------	------
BAB_RS18215	-	30S ribosomal protein S6	1.242	0.312589803	0.004010708	0.021370487	no	up	mRNA	748.12	606.2	654.55	869.11	803.09	702.41	669.623	791.53667	COG0360	J:Translation, ribosomal structure and biogenesis	GO:0006412;GO:0006412;GO:0005840;GO:0005840;GO:0019843;GO:0003735;GO:0003735;GO:0019843	BP:translation;BP:translation;CC:ribosome;CC:ribosome;MF:rRNA binding;MF:structural constituent of ribosome;MF:structural constituent of ribosome;MF:rRNA binding;
BAB_RS28800	-	glycine cleavage system protein H	1.241	0.311845033	0.001842005	0.012381511	no	up	mRNA	689.38	677.65	649.14	881.55	791.1	744.88	672.057	805.84333	COG0509	E:Amino acid transport and metabolism	GO:0019464;GO:0005960	BP:glycine decarboxylation via glycine cleavage system;CC:glycine cleavage complex;
BAB_RS20010	-	monothiol glutaredoxin%2C Grx4 family	1.24	0.310076385	0.000968263	0.008144057	no	up	mRNA	872.03	740.33	784.17	1011.03	945.88	925.99	798.843	960.96667	COG0278	O:Posttranslational modification, protein turnover, chaperones	GO:0045454;GO:0045454;GO:0005623;GO:0005623;GO:0051537;GO:0046872;GO:0015035;GO:0009055;GO:0051537;GO:0009055;GO:0046872;GO:0015035	BP:cell redox homeostasis;BP:cell redox homeostasis;CC:cell;CC:cell;MF:2 iron, 2 sulfur cluster binding;MF:metal ion binding;MF:protein disulfide oxidoreductase activity;MF:electron carrier activity;MF:2 iron, 2 sulfur cluster binding;MF:electron carrier activity;MF:metal ion binding;MF:protein disulfide oxidoreductase activity;
BAB_RS30495	-	-	1.24	0.310528625	0.009442201	0.037969701	no	up	mRNA	49.62	45.59	66.98	61.42	65.32	62.28	54.0633	63.006667	ENOG410Y1PH	S:Function unknown	------	------
BAB_RS27985	-	hypothetical protein	1.24	0.31012129	0.011169048	0.042874733	no	up	mRNA	274.35	281.76	259.09	267.6	357.92	329.09	271.733	318.20333	COG2346	S:Function unknown	GO:0019825;GO:0020037;GO:0020037;GO:0019825	MF:oxygen binding;MF:heme binding;MF:heme binding;MF:oxygen binding;

BAB_RS18445	-	biotin transporter BioY	1.239	0.309273529	0.001270245	0.009694291	no	up	mRNA	610.22	590.86	632.43	630.18	756.71	766.88	611.17	717.92333	COG1268	S:Function unknown	GO:0016021;GO:0005886;GO:0016021;GO:0005886;GO:0015225;GO:0015225	CC:integral component of membrane;CC:plasma membrane;CC:integral component of membrane;CC:plasma membrane;MF:biotin transporter activity;MF:biotin transporter activity;
BAB_RS20475	-	Fe-S cluster assembly ATPase SufC	1.239	0.30965639	0.001798217	0.012189179	no	up	mRNA	207.36	184.98	197.58	253.12	227.41	210.62	196.64	230.38333	COG0396	O:Posttranslational modification, protein turnover, chaperones	GO:0016887;GO:0005524;GO:0005524;GO:0016887	MF:ATPase activity;MF:ATP binding;MF:ATP binding;MF:ATPase activity;
BAB_RS28035	-	aspartate-semialdehyde dehydrogenase	1.239	0.309507954	0.009823726	0.039209479	no	up	mRNA	138.96	104.9	117.54	157.33	132.29	131.16	120.467	140.26	COG0136	E:Amino acid transport and metabolism	GO:0071266;GO:0019877;GO:0009097;GO:0009089;GO:0009088;GO:0071266;GO:0019877;GO:0009089;GO:0009088;GO:0009097;GO:0071266;GO:0019877;GO:0009097;GO:0009089;GO:0009088;GO:0005737;GO:0005737;GO:0005737;GO:0004073;GO:0051287;GO:0050661;GO:0046983;GO:0050661;GO:0051287;GO:0016740;GO:0046983;GO:0004073;GO:0004073;GO:0051287;GO:0050661;GO:0046983;GO:0016740	BP:'de novo' L-methionine biosynthetic process;BP:diaminopimelate biosynthetic process;BP:isoleucine biosynthetic process;BP:lysine biosynthetic process via diaminopimelate;BP:threonine biosynthetic process;BP:'de novo' L-methionine biosynthetic process;BP:diaminopimelate biosynthetic process;BP:lysine biosynthetic process via diaminopimelate;BP:threonine biosynthetic process;BP:isoleucine biosynthetic process;BP:'de novo' L-methionine biosynthetic process;BP:diaminopimelate biosynthetic process;BP:isoleucine biosynthetic process;BP:lysine biosynthetic process via diaminopimelate;BP:threonine biosynthetic process;CC:cytoplasm;CC:cytoplasm;CC:cytoplasm;MF:aspartate-semialdehyde dehydrogenase activity;MF:NAD binding;MF:NADP binding;MF:protein dimerization activity;MF:NADP binding;MF:NAD binding;MF:transferase activity;MF:protein dimerization activity;MF:aspartate-semialdehyde dehydrogenase activity;MF:aspartate-semialdehyde dehydrogenase activity;MF:NAD binding;MF:NADP [illegible]
BAB_RS33445	-	hypothetical protein	1.239	0.308705728	0.029552699	0.08380655	no	up	mRNA	35.69	31.61	51.66	44.16	48.14	46.01	39.6533	46.103333	------	------	GO:0004812	MF:aminoacyl-tRNA ligase activity;
BAB_RS28090	-	DUF1949 domain-containing protein	1.239	0.308982564	0.030609909	0.086120523	no	up	mRNA	20.29	16.34	19.21	24.27	19.83	21.18	18.6133	21.76	COG1739	S:Function unknown	------	------
BAB_RS31700	-	aspartate aminotransferase family protein	1.239	0.308923348	0.043585177	0.112481265	no	up	mRNA	6.42	6.27	8.73	8.05	8.54	8.61	7.14	8.4	COG0160	E:Amino acid transport and metabolism	GO:0030170;GO:0008483	MF:pyridoxal phosphate binding;MF:transaminase activity;
BAB_RS23960	-	tonB-system energizer ExbB	1.238	0.308078051	0.005362483	0.026264723	no	up	mRNA	65.42	70.18	63.86	77.57	84.5	71.47	66.4867	77.846667	COG0811	U:Intracellular trafficking, secretion, and vesicular transport	GO:0015031;GO:0015031;GO:0016021;GO:0016021;GO:0022857;GO:0022857	BP:protein transport;BP:protein transport;CC:integral component of membrane;CC:integral component of membrane;MF:transmembrane transporter activity;MF:transmembrane transporter activity;
BAB_RS24945	-	glycerol-3-phosphate dehydrogenase (NAD(P)(+))	1.237	0.306489544	0.019913395	0.064432768	no	up	mRNA	18.07	20.1	21.11	22.18	21.37	26.26	19.76	23.27	COG0240	C:Energy production and conversion	GO:0005975;GO:0006650;GO:0046168;GO:0046167;GO:0008654;GO:0005975;GO:0046167;GO:0046168;GO:0006650;GO:0008654;GO:0009331;GO:0009331;GO:0004367;GO:0047952;GO:0051287;GO:0036439;GO:0047952;GO:0004367;GO:0036439;GO:0051287	BP:carbohydrate metabolic process;BP:glycerophospholipid metabolic process;BP:glycerol-3-phosphate catabolic process;BP:glycerol-3-phosphate biosynthetic process;BP:phospholipid biosynthetic process;BP:carbohydrate metabolic process;BP:glycerol-3-phosphate biosynthetic process;BP:glycerol-3-phosphate catabolic process;BP:glycerophospholipid metabolic process;BP:phospholipid biosynthetic process;CC:glycerol-3-phosphate dehydrogenase complex;CC:glycerol-3-phosphate dehydrogenase complex;MF:glycerol-3-phosphate dehydrogenase [NAD+] activity;MF:glycerol-3-phosphate dehydrogenase [NA;MF:NAD binding;MF:glycerol-3-phosphate dehydrogenase [NADP+] activity;MF:glycerol-3-phosphate dehydrogenase [NA;MF:glycerol-3-phosphate dehydrogenase [NAD+] activity;MF:glycerol-3-phosphate dehydrogenase [NADP+] activity;MF:NAD binding;
BAB_RS16615	-	GNAT family N-acetyltransferase	1.236	0.305535667	7.32568E-05	0.001508808	no	up	mRNA	245.15	232.04	249.05	288.78	277.74	279.08	242.08	281.86667	COG3176	S:Function unknown	GO:0016740;GO:0016746	MF:transferase activity;MF:transferase activity, transferring acyl groups;
BAB_RS30530	nrdI	class Ib ribonucleoside-diphosphate reductase assembly flavoprotein NrdI	1.236	0.306130309	0.001619087	0.011308971	no	up	mRNA	408.69	407.36	434.1	466.41	482.76	541.37	416.717	496.84667	COG1780	F:Nucleotide transport and metabolism	------	------
BAB_RS26630	-	type VI secretion protein	1.236	0.305494711	0.035295123	0.095779754	no	up	mRNA	21.91	18.94	21.39	24.47	24.65	24.03	20.7467	24.383333	ENOG410Z6Y0;COG2885	M:Cell wall/membrane/envelope biogenesis;	GO:0009405;GO:0009405;GO:0016020;GO:0009279;GO:0016020;GO:0009279;GO:0016020	BP:pathogenesis;BP:pathogenesis;CC:membrane;CC:cell outer membrane;CC:membrane;CC:cell outer membrane;CC:membrane;
BAB_RS31035	-	DNA recombination protein RmuC	1.235	0.304474955	0.000187592	0.00279043	no	up	mRNA	136.78	119.42	141.04	146.58	158.13	156.64	132.413	153.78333	COG1322	S:Function unknown	GO:0016021	CC:integral component of membrane;
BAB_RS30000	-	cupin domain-containing protein	1.235	0.305092383	0.005119407	0.025305622	no	up	mRNA	368.37	388.53	401.17	392.22	484.86	491.2	386.023	456.09333	COG1917	S:Function unknown	------	------
BAB_RS22110	-	pyridoxal phosphate-dependent aminotransferase	1.234	0.303096507	0.000119686	0.002112913	no	up	mRNA	267.62	251.74	258.89	306.33	298.03	301.54	259.417	301.96667	COG0436	E:Amino acid transport and metabolism	GO:0009058;GO:0008483;GO:0030170	BP:biosynthetic process;MF:transaminase activity;MF:pyridoxal phosphate binding;
BAB_RS17860	-	polysaccharide deacetylase	1.234	0.302823403	0.000313094	0.003854301	no	up	mRNA	74.06	64.44	71.87	83.81	78.25	81.87	70.1233	81.31	ENOG410XW47	S:Function unknown	GO:0005975;GO:0016810	BP:carbohydrate metabolic process;MF:hydrolase activity, acting on carbon-nitrogen;
BAB_RS26065	-	molecular chaperone DnaK	1.234	0.303919444	0.002163532	0.014043293	no	up	mRNA	1421.58	1251.19	1376.16	1709.81	1560.22	1432.96	1349.64	1567.6633	COG0443	J:Translation, ribosomal structure and biogenesis	GO:0016021	CC:integral component of membrane;
BAB_RS24065	-	ATP-dependent metallopeptidase FtsH/Yme1/Tma family protein	1.233	0.302234209	0.000922423	0.007882301	no	up	mRNA	2097.04	1846.9	1950.88	2264.43	2340.22	2240.21	1964.94	2281.62	COG0465	O:Posttranslational modification, protein turnover, chaperones	GO:0030163;GO:0051301;GO:0030163;GO:0051301;GO:0030163;GO:0016021;GO:0005886;GO:0016021;GO:0005886;GO:0016021;GO:0005886;GO:0004222;GO:0005524;GO:0016887;GO:0008270;GO:0005524;GO:0016887;GO:0004222;GO:0008270;GO:0005524;GO:0016887;GO:0004222;GO:0008270	BP:protein catabolic process;BP:cell division;BP:protein catabolic process;BP:cell division;BP:protein catabolic process;CC:integral component of membrane;CC:plasma membrane;CC:integral component of membrane;CC:plasma membrane;CC:integral component of membrane;CC:plasma membrane;MF:metalloendopeptidase activity;MF:ATP binding;MF:ATPase activity;MF:zinc ion binding;MF:ATP binding;MF:ATPase activity;MF:metalloendopeptidase activity;MF:zinc ion binding;MF:ATP binding;MF:ATPase activity;MF:metalloendopeptidase activity;MF:zinc ion binding;
BAB_RS23535	-	dipeptide ABC transporter permease DppC	1.233	0.30247226	0.010602268	0.041241027	no	up	mRNA	32.85	34.48	37.52	39.63	37.15	46.12	34.95	40.966667	COG1173	P:Inorganic ion transport and metabolism	GO:0055085;GO:0055085;GO:0016021;GO:0005886;GO:0016021;GO:0005886	BP:transmembrane transport;BP:transmembrane transport;CC:integral component of membrane;CC:plasma membrane;CC:integral component of membrane;CC:plasma membrane;
BAB_RS23240	-	MarR family transcriptional regulator	1.233	0.30172053	0.011806386	0.044419109	no	up	mRNA	36.52	39.39	40.45	44.31	49.17	43.16	38.7867	45.546667	ENOG4111G0I	K:Transcription	GO:0006351;GO:0006351;GO:0003700;GO:0003677;GO:0003677;GO:0003700	BP:transcription, DNA-templated;BP:transcription, DNA-templated;MF:transcription factor activity, sequence-specific DNA binding;MF:DNA binding;MF:DNA binding;MF:transcription factor activity, sequence-specific DNA binding;
BAB_RS30435	-	DUF1269 domain-containing protein	1.232	0.301252725	0.000112629	0.002033017	no	up	mRNA	775.14	740.31	740.87	895.08	859.26	888.92	752.107	881.08667	COG4803	S:Function unknown	GO:0016021	CC:integral component of membrane;
BAB_RS26125	-	phosphonate ABC transporter substrate-binding protein	1.232	0.300811406	0.000293799	0.003701868	no	up	mRNA	88.77	83.34	91.09	98.55	106.08	101.29	87.7333	101.97333	COG0226	P:Inorganic ion transport and metabolism	------	------
BAB_RS16080	-	YbaB/EbfC family nucleoid-associated protein	1.232	0.301503191	0.000455076	0.004939728	no	up	mRNA	886.78	841.87	820.13	965.02	1077.36	1018.85	849.593	1020.41	COG0718	S:Function unknown	GO:0005737;GO:0009295;GO:0005737;GO:0043590;GO:0009295;GO:0043590;GO:0005737;GO:0009295;GO:0043590;GO:0003677;GO:0003677;GO:0003677;GO:0003677	CC:cytoplasm;CC:nucleoid;CC:cytoplasm;CC:bacterial nucleoid;CC:nucleoid;CC:bacterial nucleoid;CC:cytoplasm;CC:nucleoid;CC:bacterial nucleoid;MF:DNA binding;MF:DNA binding;MF:DNA binding;MF:DNA binding;
BAB_RS24570	-	usg family protein	1.232	0.301313046	0.017953667	0.059469209	no	up	mRNA	355.58	318.14	329.97	378.52	356.21	478.12	334.563	404.28333	COG5425	E:Amino acid transport and metabolism	------	------
BAB_RS32870	-	hypothetical protein	1.232	0.300524368	0.538832017	0.688650466	no	up	mRNA	42.87	19.06	14.29	29.22	30.17	24.48	25.4067	27.956667	------	------	GO:0016853	MF:isomerase activity;
BAB_RS23320	-	peptidyl-tRNA hydrolase	1.231	0.299374735	0.036978181	0.099423344	no	up	mRNA	28.17	30.05	25.47	36.43	28.81	32.49	27.8967	32.576667	COG0193	J:Translation, ribosomal structure and biogenesis	GO:0006412;GO:0005737;GO:0005737;GO:0004045;GO:0004045	BP:translation;CC:cytoplasm;CC:cytoplasm;MF:aminoacyl-tRNA hydrolase activity;MF:aminoacyl-tRNA hydrolase activity;
BAB_RS27525	-	RidA family protein	1.231	0.299318485	0.098834484	0.205139015	no	up	mRNA	21.02	17.39	18.83	25.08	23.05	18.59	19.08	22.24	COG0251	G:Carbohydrate transport and metabolism	------	------
BAB_RS18755	-	hypothetical protein	1.23	0.298444688	0.013093913	0.047376961	no	up	mRNA	72.6	54.14	59.77	74.51	72.75	69.05	62.17	72.103333	------	------	------	------
BAB_RS27740	-	D-aminopeptidase	1.229	0.298012136	0.001792739	0.012177739	no	up	mRNA	23.53	19.87	23.32	25.89	26.74	24.32	22.24	25.65	COG1680	E:Amino acid transport and metabolism	GO:0004177	MF:aminopeptidase activity;
BAB_RS24905	-	MFS transporter	1.229	0.297896669	0.002454933	0.015435811	no	up	mRNA	17.22	15.26	16.4	19.08	19.13	18.21	16.2933	18.806667	COG0477	G:Carbohydrate transport and metabolism	GO:0055085;GO:0008643;GO:0055085;GO:0016021;GO:0016021;GO:0005215;GO:0005215	BP:transmembrane transport;BP:carbohydrate transport;BP:transmembrane transport;CC:integral component of membrane;CC:integral component of membrane;MF:transporter activity;MF:transporter activity;
BAB_RS30520	-	class 1b ribonucleoside-diphosphate reductase subunit beta	1.228	0.296595446	0.000500598	0.005273514	no	up	mRNA	441.38	372.72	425.93	455.66	472.66	508.05	413.343	478.79	COG0208	F:Nucleotide transport and metabolism	GO:0009263;GO:0006260;GO:0009263;GO:0006260;GO:0005971;GO:0005971;GO:0004748;GO:0046872;GO:0046872;GO:0004748	BP:deoxyribonucleotide biosynthetic process;BP:DNA replication;BP:deoxyribonucleotide biosynthetic process;BP:DNA replication;CC:ribonucleoside-diphosphate reductase complex;CC:ribonucleoside-diphosphate reductase complex;MF:ribonucleoside-diphosphate reductase activity, thioredoxin disulfide as acceptor;MF:metal ion binding;MF:metal ion binding;MF:ribonucleoside-diphosphate reductase activity, thioredoxin disulfide as acceptor;
BAB_RS24975	-	DNA translocase FtsK	1.226	0.294454379	0.000105518	0.001937317	no	up	mRNA	108.12	103.38	118	124.81	124.29	130.75	109.833	126.61667	COG1674	D:Cell cycle control, cell division, chromosome partitioning	GO:0007049;GO:0007059;GO:0051301;GO:0007049;GO:0051301;GO:0007059;GO:0016021;GO:0005886;GO:0016021;GO:0005886;GO:0005524;GO:0003677;GO:0005524;GO:0003677	BP:cell cycle;BP:chromosome segregation;BP:cell division;BP:cell cycle;BP:cell division;BP:chromosome segregation;CC:integral component of membrane;CC:plasma membrane;CC:integral component of membrane;CC:plasma membrane;MF:ATP binding;MF:DNA binding;MF:ATP binding;MF:DNA binding;
BAB_RS18430	-	hypothetical protein	1.226	0.293749641	0.000987695	0.008221413	no	up	mRNA	133.2	127.86	141.55	140.92	168.15	155.89	134.203	154.98667	------	------	------	------
BAB_RS22915	-	lytic murein transglycosylase	1.226	0.293567378	0.003956051	0.021255506	no	up	mRNA	56.68	52.31	53.48	66.23	65.33	55.94	54.1567	62.5	COG2951	M:Cell wall/membrane/envelope biogenesis	GO:0008152;GO:0016798	BP:metabolic process;MF:hydrolase activity, acting on glycosyl bonds;
BAB_RS29040	-	iron ABC transporter substrate-binding protein	1.226	0.293654125	0.04726116	0.11947294	no	up	mRNA	55.9	73.47	54.46	72.97	75.36	65.73	61.2767	71.353333	COG4607	P:Inorganic ion transport and metabolism	GO:0006826	BP:iron ion transport;
BAB_RS28485	-	3-hydroxyacyl-CoA dehydrogenase	1.225	0.292884313	0.000424157	0.004667181	no	up	mRNA	312.69	285.89	307.73	324.05	358.5	362.54	302.103	348.36333	COG1250	K:Transcription	GO:0006635;GO:0004300;GO:0003857;GO:0016853	BP:fatty acid beta-oxidation;MF:enoyl-CoA hydratase activity;MF:3-hydroxyacyl-CoA dehydrogenase activity;MF:isomerase activity;
BAB_RS21335	-	Lon protease	1.224	0.291322293	0.000753622	0.00676365	no	up	mRNA	1149.94	1028.15	1113.99	1309.67	1266.75	1213.81	1097.36	1263.41	COG0466	O:Posttranslational modification, protein turnover, chaperones	GO:0030163;GO:0030163;GO:0033554;GO:0006515;GO:0033554;GO:0006515;GO:0005737;GO:0005737;GO:0005737;GO:0005524;GO:0004176;GO:0043565;GO:0004252;GO:0004176;GO:0043565;GO:0004252;GO:0005524;GO:0016491;GO:0005524;GO:0004176;GO:0043565;GO:0004252	BP:protein catabolic process;BP:protein catabolic process;BP:cellular response to stress;BP:misfolded or incompletely synthesized protein catabolic process;BP:cellular response to stress;BP:misfolded or incompletely synthesized protein catabolic process;CC:cytoplasm;CC:cytoplasm;CC:cytoplasm;MF:ATP binding;MF:ATP-dependent peptidase activity;MF:sequence-specific DNA binding;MF:serine-type endopeptidase activity;MF:ATP-dependent peptidase activity;MF:sequence-specific DNA binding;MF:serine-type endopeptidase activity;MF:ATP binding;MF:oxidoreductase activity;MF:ATP binding;MF:ATP-dependent peptidase activity;MF:sequence-specific DNA binding;MF:serine-type endopeptidase activity;
BAB_RS27875	-	porin family protein	1.224	0.292163387	0.000820546	0.007183689	no	up	mRNA	183.34	159.07	168.89	196.68	186.51	206.92	170.433	196.70333	COG3637;ENOG410Y6IE	M:Cell wall/membrane/envelope biogenesis;	------	------
BAB_RS25745	-	tRNA uridine-5-carboxymethylaminomethyl(34) synthesis enzyme MnmG	1.224	0.291030852	0.001237712	0.009605725	no	up	mRNA	80.48	75.02	81.21	99.04	85.37	88.21	78.9033	90.873333	COG0445	D:Cell cycle control, cell division, chromosome partitioning	GO:0002098;GO:0002098;GO:0005737;GO:0005737;GO:0050660;GO:0050660	BP:tRNA wobble uridine modification;BP:tRNA wobble uridine modification;CC:cytoplasm;CC:cytoplasm;MF:flavin adenine dinucleotide binding;MF:flavin adenine dinucleotide binding;
BAB_RS31055	-	elongation factor 4	1.224	0.291982407	0.001444798	0.010385094	no	up	mRNA	67.51	60.41	62.2	77.62	70.83	70.59	63.3733	73.013333	COG0481	M:Cell wall/membrane/envelope biogenesis	GO:0006412;GO:0045727;GO:0006412;GO:0045727;GO:0005886;GO:0005886;GO:0005886;GO:0003924;GO:0005525;GO:0043022;GO:0003924;GO:0003746;GO:0005525;GO:0005525;GO:0003924;GO:0043022;GO:0003746	BP:translation;BP:positive regulation of translation;BP:translation;BP:positive regulation of translation;CC:plasma membrane;CC:plasma membrane;CC:plasma membrane;MF:GTPase activity;MF:GTP binding;MF:ribosome binding;MF:GTPase activity;MF:translation elongation factor activity;MF:GTP binding;MF:GTP binding;MF:GTPase activity;MF:ribosome binding;MF:translation elongation factor activity;
BAB_RS22935	-	DUF2293 domain-containing protein	1.224	0.291019021	0.004252243	0.022324278	no	up	mRNA	287.11	272.12	313.9	315.22	368.34	345.74	291.043	343.1	COG5586	S:Function unknown	------	------
BAB_RS26445	-	condensation protein	1.224	0.291853296	0.02336491	0.071976468	no	up	mRNA	25.06	29.79	24.2	30.59	32.47	28.44	26.35	30.5	COG1020	Q:Secondary metabolites biosynthesis, transport and catabolism	------	------
BAB_RS16700	-	cysteine biosynthesis protein	1.223	0.29024837	0.00672279	0.030508934	no	up	mRNA	27.49	29.01	33.58	36.13	35.8	32.33	30.0267	34.753333	COG2981	E:Amino acid transport and metabolism	GO:0016021;GO:0016021;GO:0016874	CC:integral component of membrane;CC:integral component of membrane;MF:ligase activity;
BAB_RS25135	-	hypothetical protein	1.222	0.288727592	0.001838392	0.012409143	no	up	mRNA	213.13	226.89	218.02	240.14	256.83	269.93	219.347	255.63333	ENOG41120YI	M:Cell wall/membrane/envelope biogenesis	GO:0009279;GO:0009279;GO:0004866;GO:0004866	CC:cell outer membrane;CC:cell outer membrane;MF:endopeptidase inhibitor activity;MF:endopeptidase inhibitor activity;
BAB_RS24560	-	YggS family pyridoxal phosphate-dependent enzyme	1.222	0.289293071	0.013182271	0.04758948	no	up	mRNA	15.99	16.27	17.93	18.15	21.25	18.5	16.73	19.3	COG0325	S:Function unknown	GO:0016740;GO:0030170;GO:0030170	MF:transferase activity;MF:pyridoxal phosphate binding;MF:pyridoxal phosphate binding;
BAB_RS16200	-	alpha/beta hydrolase	1.222	0.288847339	0.016481879	0.055861051	no	up	mRNA	18.4	16.24	20.03	22.13	19.68	21.35	18.2233	21.053333	COG1073	S:Function unknown	GO:0016787;GO:0008236	MF:hydrolase activity;MF:serine-type peptidase activity;
BAB_RS24020	-	MFS transporter	1.221	0.288231932	0.000311877	0.003854082	no	up	mRNA	34.14	30.78	35.08	39.06	38.29	37.36	33.3333	38.236667	ENOG410XP7I	S:Function unknown	GO:0055085;GO:0055085;GO:0016021;GO:0016021	BP:transmembrane transport;BP:transmembrane transport;CC:integral component of membrane;CC:integral component of membrane;
BAB_RS22070	-	lactoylglutathione lyase	1.221	0.288517777	0.000797872	0.00702346	no	up	mRNA	160.19	148.11	147.56	175.89	177.03	176.8	151.953	176.57333	COG0346	S:Function unknown	GO:0046872;GO:0004462	MF:metal ion binding;MF:lactoylglutathione lyase activity;
BAB_RS27340	-	potassium transporter TrkA	1.22	0.286333691	0.000161596	0.002545129	no	up	mRNA	152.73	141.78	149.63	169.89	167.62	172.29	148.047	169.93333	COG0475	P:Inorganic ion transport and metabolism	GO:0006813;GO:0006813;GO:0016021;GO:0016021;GO:0015299;GO:0015299	BP:potassium ion transport;BP:potassium ion transport;CC:integral component of membrane;CC:integral component of membrane;MF:solut;MF:solut;
BAB_RS19120	-	lytic transglycosylase	1.22	0.28644395	0.000247954	0.003292047	no	up	mRNA	162.06	155.02	172.57	189.82	177.44	194.37	163.217	187.21	COG0741	S:Function unknown	GO:0042597;GO:0004553	CC:periplasmic space;MF:hydrolase activity, hydrolyzing O-glycosyl compounds;
BAB_RS29395	-	metal-dependent hydrolase	1.22	0.287131631	0.000367195	0.004274626	no	up	mRNA	482.1	417.13	438.77	503.97	531.65	508.51	446	514.71	COG2220	S:Function unknown	GO:0016787	MF:hydrolase activity;
BAB_RS30020	-	hemolysin secretion protein D	1.22	0.287291722	0.045297886	0.115693249	no	up	mRNA	67.1	66.48	66.04	95.1	67.33	67.9	66.54	76.776667	COG0845	V:Defense mechanisms	------	------
BAB_RS29025	-	iron ABC transporter ATP-binding protein	1.22	0.287231581	0.047520028	0.119750471	no	up	mRNA	7.64	9.58	10.95	10.26	11.28	11.23	9.39	10.923333	COG4604	P:Inorganic ion transport and metabolism	GO:0005524;GO:0016887	MF:ATP binding;MF:ATPase activity;
BAB_RS20080	-	lauroyl acyltransferase	1.218	0.284216251	0.003150287	0.018370006	no	up	mRNA	35.61	31.95	36.26	42.18	37.64	39.42	34.6067	39.746667	COG1560	M:Cell wall/membrane/envelope biogenesis	GO:0016021;GO:0016021;GO:0016021;GO:0016746;GO:0016740;GO:0016746;GO:0016746	CC:integral component of membrane;CC:integral component of membrane;CC:integral component of membrane;MF:transferase activity, transferring acyl groups;MF:transferase activity;MF:transferase activity, transferring acyl groups;MF:transferase activity, transferring acyl groups;
BAB_RS18605	-	mannose-1-phosphate guanylyltransferase/mannose-6-phosphate isomerase	1.217	0.282912789	0.001286439	0.009748418	no	up	mRNA	193.05	162.08	169.18	194.61	202.13	203.91	174.77	200.21667	COG0836	M:Cell wall/membrane/envelope biogenesis	GO:0000271;GO:0016779;GO:0016853;GO:0008928	BP:polysaccharide biosynthetic process;MF:nucleotidyltransferase activity;MF:isomerase activity;MF:mannose-1-phosphate guanylyltransferas;
BAB_RS19375	-	membrane protein	1.217	0.283867137	0.003892649	0.021055691	no	up	mRNA	7239.49	6871.03	7165.57	7812.42	8446.09	8164.83	7092.03	8141.1133	COG3637	L:Replication, recombination and repair	GO:0016021;GO:0016021;GO:0009279	CC:integral component of membrane;CC:integral component of membrane;CC:cell outer membrane;
BAB_RS20375	-	1-aminocyclopropane-1-carboxylate deaminase	1.217	0.283133938	0.005828421	0.027743283	no	up	mRNA	58.3	49.41	53.02	67.52	58.39	58.06	53.5767	61.323333	COG0436	E:Amino acid transport and metabolism	GO:0009058;GO:0009058;GO:0030170;GO:0008483;GO:0008483;GO:0030170;GO:0004069;GO:0003824	BP:biosynthetic process;BP:biosynthetic process;MF:pyridoxal phosphate binding;MF:transaminase activity;MF:transaminase activity;MF:pyridoxal phosphate binding;MF:L-aspartat;MF:catalytic activity;
BAB_RS19420	-	phosphoribosylformylglycinamidine cyclo-ligase	1.217	0.283629876	0.006185372	0.028760635	no	up	mRNA	44.72	37.43	43.05	52.22	46.76	44.37	41.7333	47.783333	COG0150	F:Nucleotide transport and metabolism	GO:0006189;GO:0006189;GO:0005737;GO:0005737;GO:0005524;GO:0004641;GO:0004641;GO:0016829;GO:0005524	BP:'de novo' IMP biosynthetic process;BP:'de novo' IMP biosynthetic process;CC:cytoplasm;CC:cytoplasm;MF:ATP binding;MF:phosphoribosylformylglycinamidine cyclo-ligase activity;MF:phosphoribosylformylglycinamidine cyclo-ligase activity;MF:lyase activity;MF:ATP binding;
BAB_RS31710	-	haloacid dehalogenase	1.217	0.283122845	0.476257798	0.638921213	no	up	mRNA	3.05	2.35	1.61	3.67	2.33	1.8	2.33667	2.6	COG1011	E:Amino acid transport and metabolism	GO:0008152;GO:0016787	BP:metabolic process;MF:hydrolase activity;
BAB_RS20545	-	valine--tRNA ligase	1.215	0.280442621	0.0006439	0.006250307	no	up	mRNA	173.81	150.99	170.92	196.59	177.78	191.44	165.24	188.60333	COG0525	J:Translation, ribosomal structure and biogenesis	GO:0006438;GO:0006438;GO:0005737;GO:0005737;GO:0004832;GO:0002161;GO:0005524;GO:0002161;GO:0005524;GO:0004832	BP:valyl-tRNA aminoacylation;BP:valyl-tRNA aminoacylation;CC:cytoplasm;CC:cytoplasm;MF:valine-tRNA ligase activity;MF:aminoacyl-tRNA editing activity;MF:ATP binding;MF:aminoacyl-tRNA editing activity;MF:ATP binding;MF:valine-tRNA ligase activity;
BAB_RS19800	-	50S ribosomal protein L13	1.215	0.280566949	0.012178988	0.045186015	no	up	mRNA	621.11	465.41	544.1	697.31	577.97	602.51	543.54	625.93	COG0102	J:Translation, ribosomal structure and biogenesis	GO:0006412;GO:0005840;GO:0003735	BP:translation;CC:ribosome;MF:structural constituent of ribosome;
BAB_RS24900	-	methyltransferase domain-containing protein	1.215	0.280370036	0.142071694	0.268357645	no	up	mRNA	12.88	10.58	9.43	15.4	11.51	10.38	10.9633	12.43	COG0500	S:Function unknown	GO:0008168	MF:methyltransferase activity;
BAB_RS24225	-	transketolase	1.214	0.279855872	0.000366214	0.004278707	no	up	mRNA	281.63	258.6	282.58	328.51	300.56	310.75	274.27	313.27333	COG0021	G:Carbohydrate transport and metabolism	GO:0008152;GO:0008152;GO:0046872;GO:0004802;GO:0004802;GO:0046872	BP:metabolic process;BP:metabolic process;MF:metal ion binding;MF:transketolase activity;MF:transketolase activity;MF:metal ion binding;
BAB_RS17150	-	winged helix-turn-helix domain-containing protein	1.214	0.280035066	0.040398356	0.106393376	no	up	mRNA	8.78	9.06	10.31	11.37	10.51	10.56	9.38333	10.813333	ENOG4111ZP1	S:Function unknown	GO:0032088;GO:0043565;GO:0043565	BP:negative regulation of NF-kappaB transcription factor activity;MF:sequence-specific DNA binding;MF:sequence-specific DNA binding;
BAB_RS25365	-	YaiI/YqxD family protein	1.214	0.279475454	0.056326814	0.136073724	no	up	mRNA	18.52	18.73	24.98	24.78	26.64	19.99	20.7433	23.803333	COG1671	S:Function unknown	------	------
BAB_RS23095	-	DUF2306 domain-containing protein	1.214	0.279885457	0.737479289	0.839957801	no	up	mRNA	0	1.05	0.92	1.33	0.38	0.95	0.65667	0.8866667	COG5395	S:Function unknown	GO:0016021	CC:integral component of membrane;
BAB_RS17990	-	FAD-binding oxidoreductase	1.213	0.278236419	0.000940536	0.008015759	no	up	mRNA	42.01	38.24	45.85	48.23	46.82	48.92	42.0333	47.99	COG0277	C:Energy production and conversion	GO:0050660;GO:0016614	MF:flavin adenine dinucleotide binding;MF:oxidoreductase activity, acting on CH-OH group of donors;
BAB_RS25610	-	hypothetical protein	1.213	0.278388273	0.000980099	0.008179372	no	up	mRNA	265.88	260.81	279.99	284.35	305.49	333.15	268.893	307.66333	COG3034	S:Function unknown	------	------
BAB_RS27290	-	isoleucine--tRNA ligase	1.213	0.278477311	0.001229447	0.009587893	no	up	mRNA	56.87	46.63	53.74	61.71	57.8	59.49	52.4133	59.666667	COG0060	J:Translation, ribosomal structure and biogenesis	GO:0006428;GO:0006428;GO:0005737;GO:0005737;GO:0000049;GO:0016853;GO:0004822;GO:0002161;GO:0005524;GO:0002161;GO:0005524;GO:0004822;GO:0000049	BP:isoleucyl-tRNA aminoacylation;BP:isoleucyl-tRNA aminoacylation;CC:cytoplasm;CC:cytoplasm;MF:tRNA binding;MF:isomerase activity;MF:isoleucine-tRNA ligase activity;MF:aminoacyl-tRNA editing activity;MF:ATP binding;MF:aminoacyl-tRNA editing activity;MF:ATP binding;MF:isoleucine-tRNA ligase activity;MF:tRNA binding;
BAB_RS20820	-	ATPase	1.213	0.278607909	0.001465164	0.010438072	no	up	mRNA	61.02	60.08	66.7	70.53	71.28	74.03	62.6	71.946667	COG5387	O:Posttranslational modification, protein turnover, chaperones	GO:0043461	BP:proton-transporting ATP synthase complex assembly;
BAB_RS22155	-	porin family protein	1.213	0.278516508	0.004815051	0.024248838	no	up	mRNA	306.94	262.38	265.91	349.17	306.57	302.15	278.41	319.29667	COG3637	L:Replication, recombination and repair	------	------
BAB_RS23420	-	MoxR family ATPase	1.212	0.277620557	0.00048628	0.005156498	no	up	mRNA	87.29	80.82	85.86	95.24	96.78	98.43	84.6567	96.816667	COG0714	S:Function unknown	GO:0005524;GO:0016887;GO:0016887;GO:0005524	MF:ATP binding;MF:ATPase activity;MF:ATPase activity;MF:ATP binding;
BAB_RS15985	-	ABC transporter substrate-binding protein	1.212	0.277793304	0.001273895	0.009676178	no	up	mRNA	266.14	236.47	236.05	282.44	285.28	275.99	246.22	281.23667	COG4166	E:Amino acid transport and metabolism	GO:0055085;GO:0043190	BP:transmembrane transport;CC:ATP-binding cassett;
BAB_RS26820	-	amidase	1.212	0.277671467	0.001842063	0.012356049	no	up	mRNA	88.11	78.56	84.14	87.65	100.4	98.35	83.6033	95.466667	COG0154	K:Transcription	GO:0016740;GO:0004040;GO:0004040	MF:transferase activity;MF:amidase activity;MF:amidase activity;
BAB_RS30550	-	gfo/Idh/MocA family oxidoreductase	1.212	0.277342145	0.0047816	0.024194145	no	up	mRNA	18.57	19.18	21.14	21.75	22.83	22.86	19.63	22.48	COG0673	G:Carbohydrate transport and metabolism	GO:0050112;GO:0016491;GO:0016491	MF:inositol 2-dehydrogenase activity;MF:oxidoreductase activity;MF:oxidoreductase activity;
BAB_RS16405	-	AsnC family transcriptional regulator	1.212	0.276851135	0.016525997	0.055951559	no	up	mRNA	33.67	31.9	29.77	36.97	35.46	36.84	31.78	36.423333	ENOG4111XEU	K:Transcription	------	------
BAB_RS29195	-	AraC family transcriptional regulator	1.212	0.277236268	0.200490903	0.343378076	no	up	mRNA	10.02	7.4	10.76	14.19	10.14	7.62	9.39333	10.65	COG2207	K:Transcription	GO:0006351;GO:0003700;GO:0043565	BP:transcription, DNA-templated;MF:transcription factor activity, sequence-specific DNA binding;MF:sequence-specific DNA binding;
BAB_RS16625	-	RNA methyltransferase	1.21	0.274978505	0.008126548	0.034629442	no	up	mRNA	54.76	44.93	48.05	51.43	58.54	58.16	49.2467	56.043333	COG0566	J:Translation, ribosomal structure and biogenesis	GO:0006396;GO:0006396;GO:0008173;GO:0003723;GO:0003723;GO:0008173	BP:RNA processing;BP:RNA processing;MF:RNA methyltransferase activity;MF:RNA binding;MF:RNA binding;MF:RNA methyltransferase activity;
BAB_RS26540	-	magnesium-translocating P-type ATPase	1.21	0.274422423	0.010820887	0.041888566	no	up	mRNA	8.52	6.88	8.01	8.38	9.13	8.97	7.80333	8.8266667	COG0474	P:Inorganic ion transport and metabolism	GO:0008152;GO:0016021;GO:0016021;GO:0015444;GO:0000166;GO:0032428;GO:0015444;GO:0000166	BP:metabolic process;CC:integral component of membrane;CC:integral component of membrane;MF:magnesium-importing ATPase activity;MF:nucleotide binding;MF:beta-N-acetylgalactosaminidase activity;MF:magnesium-importing ATPase activity;MF:nucleotide binding;
BAB_RS22560	-	fluoride ion transporter CrcB	1.209	0.273725806	0.000605626	0.005987313	no	up	mRNA	275.32	285.71	313.52	326.63	330.64	343.18	291.517	333.48333	COG3439	S:Function unknown	GO:0016021;GO:0005887;GO:0005886;GO:0015103	CC:integral component of membrane;CC:integral component of plasma membrane;CC:plasma membrane;MF:inorganic anion transmembrane transporter activity;
BAB_RS28105	-	fructose-bisphosphate aldolase class II	1.208	0.272208922	0.000348133	0.004142779	no	up	mRNA	161.57	151.19	164.55	175.09	180.33	188.56	159.103	181.32667	COG0191	G:Carbohydrate transport and metabolism	GO:0006096;GO:0006096;GO:0004332;GO:0008270;GO:0004180;GO:0008270;GO:0004332	BP:glycolytic process;BP:glycolytic process;MF:fructose-bisphosphate aldolase activity;MF:zinc ion binding;MF:carboxypeptidase activity;MF:zinc ion binding;MF:fructose-bisphosphate aldolase activity;
BAB_RS20210	-	sporulation domain-containing protein	1.208	0.27279004	0.000644287	0.006216498	no	up	mRNA	741.87	699.96	769.17	835.71	807.4	868.8	737	837.30333	ENOG41122N3;ENOG4111DAJ	S:Function unknown;	GO:0016021;GO:0042834	CC:integral component of membrane;MF:peptidoglycan binding;
BAB_RS18955	-	cytochrome c-type biogenesis protein CcmE	1.208	0.2727741	0.015729984	0.053881066	no	up	mRNA	151.9	138.25	159.81	195.86	167.57	155.12	149.987	172.85	COG2332	O:Posttranslational modification, protein turnover, chaperones	GO:0017004;GO:0017003;GO:0017003;GO:0017004;GO:0016021;GO:0005886;GO:0016021;GO:0005886;GO:0020037;GO:0046872;GO:0020037;GO:0046872	BP:cytochrome complex assembly;BP:protein-heme linkage;BP:protein-heme linkage;BP:cytochrome complex assembly;CC:integral component of membrane;CC:plasma membrane;CC:integral component of membrane;CC:plasma membrane;MF:heme binding;MF:metal ion binding;MF:heme binding;MF:metal ion binding;
BAB_RS24585	-	protease HtpX	1.207	0.271612474	0.000246791	0.003303913	no	up	mRNA	291.78	258.75	295.12	329.02	319.83	313.64	281.883	320.83	COG0501	O:Posttranslational modification, protein turnover, chaperones	GO:0016021;GO:0005886;GO:0016021;GO:0005886;GO:0004222;GO:0008270;GO:0046872;GO:0004222;GO:0008270	CC:integral component of membrane;CC:plasma membrane;CC:integral component of membrane;CC:plasma membrane;MF:metalloendopeptidase activity;MF:zinc ion binding;MF:metal ion binding;MF:metalloendopeptidase activity;MF:zinc ion binding;
BAB_RS25540	-	MerR family DNA-binding transcriptional regulator	1.207	0.270907906	0.016708412	0.056450187	no	up	mRNA	27.71	28.9	35.69	35.37	38.1	31.64	30.7667	35.036667	COG0789	S:Function unknown	GO:0006355;GO:0006355;GO:0003677;GO:0003677	BP:regulation of transcription, DNA-templated;BP:regulation of transcription, DNA-templated;MF:DNA binding;MF:DNA binding;
BAB_RS28690	-	RidA family protein	1.207	0.271671122	0.025413886	0.076028691	no	up	mRNA	94.72	97.04	108.96	119.48	112.21	122.02	100.24	117.90333	COG0251	S:Function unknown	------	------
BAB_RS30205	-	dihydrodipicolinate synthase family protein	1.207	0.271223665	0.0644688	0.149558306	no	up	mRNA	10.35	9.97	9.75	12.54	10.56	11.18	10.0233	11.426667	COG0329	K:Transcription	GO:0008152;GO:0008152;GO:0016829;GO:0016829;GO:0016301	BP:metabolic process;BP:metabolic process;MF:lyase activity;MF:lyase activity;MF:kinase activity;
BAB_RS26640	-	type IV secretion system protein VirB10	1.206	0.269653278	0.02042681	0.065697037	no	up	mRNA	24.25	20.13	23.96	24.66	23.82	29.14	22.78	25.873333	COG2948	U:Intracellular trafficking, secretion, and vesicular transport	GO:0009405;GO:0009405;GO:0016021;GO:0005886;GO:0016021;GO:0016021;GO:0005886	BP:pathogenesis;BP:pathogenesis;CC:integral component of membrane;CC:plasma membrane;CC:integral component of membrane;CC:integral component of membrane;CC:plasma membrane;
BAB_RS16205	-	nucleoside/nucleotide kinase family protein	1.206	0.269911692	0.252419329	0.405308997	no	up	mRNA	6.74	5.81	4.05	6.29	6.55	5.65	5.53333	6.1633333	COG1072	H:Coenzyme transport and metabolism	GO:0016301	MF:kinase activity;
BAB_RS17375	-	Urease accessory protein UreG 1	1.205	0.269429463	0.001401227	0.010255453	no	up	mRNA	115.51	101.26	107.78	118.33	125.49	125.87	108.183	123.23	COG0378;COG0378	K:Transcription;O:Posttranslational modification, protein turnover, chaperones	GO:0006807;GO:0009405;GO:0006807;GO:0005737;GO:0005737;GO:0016151;GO:0003924;GO:0005525;GO:0016151;GO:0003924;GO:0005525	BP:nitrogen compound metabolic process;BP:pathogenesis;BP:nitrogen compound metabolic process;CC:cytoplasm;CC:cytoplasm;MF:nickel cation binding;MF:GTPase activity;MF:GTP binding;MF:nickel cation binding;MF:GTPase activity;MF:GTP binding;
BAB_RS19025	-	AP endonuclease	1.205	0.268631545	0.002807272	0.016827918	no	up	mRNA	39.35	38.26	38.99	46.48	40.94	44.93	38.8667	44.116667	COG3021	S:Function unknown	GO:0016021;GO:0016021;GO:0004519;GO:0004527;GO:0004519	CC:integral component of membrane;CC:integral component of membrane;MF:endonuclease activity;MF:exonuclease activity;MF:endonuclease activity;
BAB_RS27965	-	alkene reductase	1.205	0.26844889	0.004743873	0.02411719	no	up	mRNA	35.81	34.96	37.03	36.56	42.93	42.87	35.9333	40.786667	COG1902	K:Transcription	GO:0010181;GO:0016491;GO:0016491;GO:0010181	MF:FMN binding;MF:oxidoreductase activity;MF:oxidoreductase activity;MF:FMN binding;
BAB_RS30005	-	ABC transporter permease	1.205	0.268853435	0.184254611	0.322270041	no	up	mRNA	9.03	8.85	7.59	12.48	8.2	8.23	8.49	9.6366667	COG0842	S:Function unknown	GO:0055085;GO:0055085;GO:0043190;GO:0043190;GO:0016740	BP:transmembrane transport;BP:transmembrane transport;CC:ATP-binding cassett;CC:ATP-binding cassett;MF:transferase activity;
BAB_RS18580	-	transposase	1.205	0.268754455	0.353280282	0.518305729	no	up	mRNA	6.1	4.34	5.92	4.32	5.47	8.94	5.45333	6.2433333	COG2801;ENOG410XP6T	L:Replication, recombination and repair;	GO:0015074	BP:DNA integration;
BAB_RS30140	-	PIG-L family deacetylase	1.205	0.269236041	0.61240345	0.745322836	no	up	mRNA	1.71	0.61	0.9	1.37	1.16	0.92	1.07333	1.15	COG2120	S:Function unknown	------	------
BAB_RS21325	-	excinuclease ABC subunit A	1.204	0.267670555	0.00027464	0.003487822	no	up	mRNA	104.31	93.19	104.39	112.91	117.29	111.27	100.63	113.82333	COG0178	L:Replication, recombination and repair	GO:0006289;GO:0009432;GO:0005737;GO:0009380;GO:0016887;GO:0009381;GO:0004518;GO:0008270;GO:0003677;GO:0046872;GO:0005524	BP:nucleotide-excision repair;BP:SOS response;CC:cytoplasm;CC:excinuclease repair complex;MF:ATPase activity;MF:excinuclease ABC activity;MF:nuclease activity;MF:zinc ion binding;MF:DNA binding;MF:metal ion binding;MF:ATP binding;
BAB_RS21005	-	6-aminohexanoate-dimer hydrolase	1.204	0.267775323	0.000466576	0.005013737	no	up	mRNA	92.26	89.55	95.33	99.44	106.38	108.42	92.38	104.74667	COG1680	V:Defense mechanisms	GO:0016021;GO:0016787	CC:integral component of membrane;MF:hydrolase activity;
BAB_RS23375	-	hypothetical protein	1.204	0.268144131	0.000710065	0.006537072	no	up	mRNA	50.88	50.72	54.4	58.11	59.88	59.15	52	59.046667	COG5042	F:Nucleotide transport and metabolism	GO:0055085	BP:transmembrane transport;
BAB_RS33040	-	hypothetical protein	1.204	0.268152254	0.002079377	0.013551805	no	up	mRNA	70.23	64.5	70.4	79.09	80.1	74.33	68.3767	77.84	------	------	------	------
BAB_RS26000	-	hypothetical protein	1.204	0.267726059	0.003686178	0.020211074	no	up	mRNA	1040.06	1107.09	1170.88	1204.6	1388.77	1215.93	1106.01	1269.7667	ENOG4111NUQ	S:Function unknown	GO:0016021	CC:integral component of membrane;
BAB_RS23080	-	GatB/YqeY domain-containing protein	1.204	0.267653395	0.003949294	0.02125474	no	up	mRNA	48.19	42.58	52.59	59.11	50.98	51.97	47.7867	54.02	COG1610	S:Function unknown	GO:0016740;GO:0016884;GO:0016884	MF:transferase activity;MF:carbon-nitrogen ligase activity, with glutamine as amido-N-donor;MF:carbon-nitrogen ligase activity, with glutamine as amido-N-donor;
BAB_RS24155	-	inositol monophosphatase	1.204	0.268003022	0.004743722	0.024154642	no	up	mRNA	68.59	62.46	67.3	81.93	74.61	68.44	66.1167	74.993333	COG0483	J:Translation, ribosomal structure and biogenesis	GO:0046855;GO:0046854;GO:0046855;GO:0046854;GO:0008934;GO:0052832;GO:0052833;GO:0046872;GO:0008934;GO:0052832;GO:0052833;GO:0046872	BP:inositol phosphate dephosphorylation;BP:phosphatidylinositol phosphorylation;BP:inositol phosphate dephosphorylation;BP:phosphatidylinositol phosphorylation;MF:inositol monophosphate 1-phosphatase activity;MF:inositol monophosphate 3-phosphatase activity;MF:inositol monophosphate 4-phosphatase activity;MF:metal ion binding;MF:inositol monophosphate 1-phosphatase activity;MF:inositol monophosphate 3-phosphatase activity;MF:inositol monophosphate 4-phosphatase activity;MF:metal ion binding;
BAB_RS29495	-	ABC transporter substrate-binding protein	1.203	0.266396632	0.000570374	0.005709068	no	up	mRNA	270.52	238.32	253.57	284.77	293.72	284.75	254.137	287.74667	COG4166	Q:Secondary metabolites biosynthesis, transport and catabolism	GO:0055085;GO:0055085;GO:0055085;GO:0043190;GO:0043190;GO:0042597;GO:0043190	BP:transmembrane transport;BP:transmembrane transport;BP:transmembrane transport;CC:ATP-binding cassett;CC:ATP-binding cassett;CC:periplasmic space;CC:ATP-binding cassett;
BAB_RS30425	-	amino acid permease	1.203	0.266300857	0.000740989	0.00672542	no	up	mRNA	68.84	60.57	70.38	74.87	77.6	73.48	66.5967	75.316667	COG0531	E:Amino acid transport and metabolism	GO:0016021;GO:0022857	CC:integral component of membrane;MF:transmembrane transporter activity;
BAB_RS26030	-	isoprenylcysteine carboxylmethyltransferase family protein	1.203	0.266160013	0.028584279	0.08185498	no	up	mRNA	84.46	63.9	64.2	84.86	80.61	74.52	70.8533	79.996667	COG2020	O:Posttranslational modification, protein turnover, chaperones	GO:0016021;GO:0016021;GO:0008168	CC:integral component of membrane;CC:integral component of membrane;MF:methyltransferase activity;
BAB_RS19670	-	amidase	1.202	0.265419295	0.000635628	0.006188709	no	up	mRNA	713.85	630.22	673.48	755.64	785.58	744.58	672.517	761.93333	COG2989	S:Function unknown	GO:0016740	MF:transferase activity;
BAB_RS21210	-	recombinase family protein	1.201	0.264248085	0.112076763	0.224083782	no	up	mRNA	28.23	18.54	23.54	30.49	26.72	21.48	23.4367	26.23	COG1961	L:Replication, recombination and repair	GO:0000150;GO:0003677;GO:0003677;GO:0000150	MF:recombinase activity;MF:DNA binding;MF:DNA binding;MF:recombinase activity;
BAB_RS17320	-	recombinase family protein	1.201	0.264248085	0.112076763	0.224083782	no	up	mRNA	28.23	18.54	23.54	30.49	26.72	21.48	23.4367	26.23	COG1961	L:Replication, recombination and repair	GO:0000150;GO:0003677;GO:0003677;GO:0000150	MF:recombinase activity;MF:DNA binding;MF:DNA binding;MF:recombinase activity;
BAB_RS25125	-	malate dehydrogenase	1.2	0.262818045	0.000511324	0.005334035	no	up	mRNA	1463.16	1306.56	1402.73	1591.29	1558.1	1576.3	1390.82	1575.23	COG0039	C:Energy production and conversion	GO:0005975;GO:0006099;GO:0005975;GO:0006099;GO:0006099;GO:0005975;GO:0016021;GO:0030060;GO:0030060;GO:0030060	BP:carbohydrate metabolic process;BP:tricarboxylic acid cycle;BP:carbohydrate metabolic process;BP:tricarboxylic acid cycle;BP:tricarboxylic acid cycle;BP:carbohydrate metabolic process;CC:integral component of membrane;MF:L-malate dehydrogenase activity;MF:L-malate dehydrogenase activity;MF:L-malate dehydrogenase activity;
BAB_RS28075	-	peptidase M3	1.2	0.262850867	0.001316727	0.009884683	no	up	mRNA	159.99	145.32	155.49	168.91	186.62	164.49	153.6	173.34	COG0339	E:Amino acid transport and metabolism	GO:0046872;GO:0004222;GO:0046872;GO:0004222;GO:0004180;GO:0004180;GO:0046872;GO:0004222	MF:metal ion binding;MF:metalloendopeptidase activity;MF:metal ion binding;MF:metalloendopeptidase activity;MF:carboxypeptidase activity;MF:carboxypeptidase activity;MF:metal ion binding;MF:metalloendopeptidase activity;
BAB_RS20480	-	Fe-S cluster assembly protein SufD	1.2	0.262816307	0.002211924	0.01418545	no	up	mRNA	143.03	132.33	138.47	168.71	148.33	151.08	137.943	156.04	COG0719	O:Posttranslational modification, protein turnover, chaperones	GO:0016226	BP:iron-sulfur cluster assembly;
BAB_RS24500	-	GntR family transcriptional regulator	1.2	0.262960809	0.007565891	0.032850281	no	up	mRNA	48.23	46.51	52.47	59.17	55.79	52.49	49.07	55.816667	COG1802	E:Amino acid transport and metabolism	GO:0006351;GO:0006351;GO:0003700;GO:0003677;GO:0003677;GO:0003700	BP:transcription, DNA-templated;BP:transcription, DNA-templated;MF:transcription factor activity, sequence-specific DNA binding;MF:DNA binding;MF:DNA binding;MF:transcription factor activity, sequence-specific DNA binding;
BAB_RS22845	-	putative lipid II flippase FtsW	1.199	0.261876025	0.00122612	0.009585218	no	up	mRNA	126.02	122.06	137.29	139.7	141.09	155.29	128.457	145.36	COG0772	D:Cell cycle control, cell division, chromosome partitioning	GO:0008360;GO:0009252;GO:0051301;GO:0051301;GO:0009252;GO:0008360;GO:0016021;GO:0005886;GO:0016021;GO:0005886	BP:regulation of cell shape;BP:peptidoglycan biosynthetic process;BP:cell division;BP:cell division;BP:peptidoglycan biosynthetic process;BP:regulation of cell shape;CC:integral component of membrane;CC:plasma membrane;CC:integral component of membrane;CC:plasma membrane;
BAB_RS24730	obgE	GTPase ObgE	1.199	0.26192825	0.008376489	0.035459368	no	up	mRNA	26.99	25.62	29.54	33.33	30.64	28.98	27.3833	30.983333	COG0536	S:Function unknown	GO:0042254;GO:0042254;GO:0005737;GO:0005737;GO:0005737;GO:0000287;GO:0003924;GO:0005525;GO:0003924;GO:0000287;GO:0005525;GO:0003924;GO:0000287	BP:ribosome biogenesis;BP:ribosome biogenesis;CC:cytoplasm;CC:cytoplasm;CC:cytoplasm;MF:magnesium ion binding;MF:GTPase activity;MF:GTP binding;MF:GTPase activity;MF:magnesium ion binding;MF:GTP binding;MF:GTPase activity;MF:magnesium ion binding;
BAB_RS23895	-	hypothetical protein	1.199	0.261966297	0.01025301	0.040174294	no	up	mRNA	92.15	83.48	82.39	107.8	92.57	91.41	86.0067	97.26	ENOG4112A78	S:Function unknown	GO:0016021	CC:integral component of membrane;
BAB_RS22945	-	trans-aconitate 2-methyltransferase	1.199	0.261951633	0.013357824	0.048007483	no	up	mRNA	29.18	26.91	25.39	31.41	30.95	29.58	27.16	30.646667	COG4106	S:Function unknown	GO:0005737;GO:0005737;GO:0030798;GO:0030798	CC:cytoplasm;CC:cytoplasm;MF:trans-aconitate 2-methyltransferase activity;MF:trans-aconitate 2-methyltransferase activity;
BAB_RS17985	-	threonylcarbamoyl-AMP synthase	1.199	0.261596802	0.015019965	0.051947413	no	up	mRNA	29.71	35.91	35.81	38.19	38.16	39.15	33.81	38.5	COG0009	J:Translation, ribosomal structure and biogenesis	GO:0008033;GO:0008033;GO:0005737;GO:0005737;GO:0003725;GO:0005524;GO:0061710;GO:0005524;GO:0003725;GO:0061710	BP:tRNA processing;BP:tRNA processing;CC:cytoplasm;CC:cytoplasm;MF:double-stranded RNA binding;MF:ATP binding;MF:L-threonylcarbamoyladenylate synthase;MF:ATP binding;MF:double-stranded RNA binding;MF:L-threonylcarbamoyladenylate synthase;
BAB_RS26725	-	hypothetical protein	1.198	0.261074842	0.000668918	0.006358677	no	up	mRNA	522.07	476.27	478.13	552.64	566.35	585.66	492.157	568.21667	ENOG41128TQ	M:Cell wall/membrane/envelope biogenesis	GO:0009279	CC:cell outer membrane;
BAB_RS16265	-	argininosuccinate synthase	1.197	0.259901235	0.000951856	0.00804819	no	up	mRNA	132.24	122.52	124.7	144.51	137.86	145.79	126.487	142.72	COG0137	E:Amino acid transport and metabolism	GO:0006526;GO:0006526;GO:0005737;GO:0005737;GO:0004055;GO:0005524;GO:0016491;GO:0004055;GO:0005524	BP:arginine biosynthetic process;BP:arginine biosynthetic process;CC:cytoplasm;CC:cytoplasm;MF:argininosuccinate synthase activity;MF:ATP binding;MF:oxidoreductase activity;MF:argininosuccinate synthase activity;MF:ATP binding;
BAB_RS28330	-	hypothetical protein	1.197	0.259396757	0.001731591	0.01181232	no	up	mRNA	131.55	129.83	139.1	145.17	142.2	163.82	133.493	150.39667	COG0790	S:Function unknown	------	------
BAB_RS28545	-	pyrroline-5-carboxylate reductase	1.197	0.258993512	0.002698728	0.016329594	no	up	mRNA	57.96	56.52	56.07	65.97	64.12	62.6	56.85	64.23	COG0345	E:Amino acid transport and metabolism	GO:0055129;GO:0055129;GO:0055129;GO:0055129;GO:0005737;GO:0005737;GO:0005737;GO:0005737;GO:0004735;GO:0016757;GO:0004735;GO:0004735;GO:0016757;GO:0004735	BP:L-proline biosynthetic process;BP:L-proline biosynthetic process;BP:L-proline biosynthetic process;BP:L-proline biosynthetic process;CC:cytoplasm;CC:cytoplasm;CC:cytoplasm;CC:cytoplasm;MF:pyrroline-5-carboxylate reductase activity;MF:transferase activity, transferring glycosyl groups;MF:pyrroline-5-carboxylate reductase activity;MF:pyrroline-5-carboxylate reductase activity;MF:transferase activity, transferring glycosyl groups;MF:pyrroline-5-carboxylate reductase activity;
BAB_RS20355	-	acetyl-CoA carboxylase biotin carboxyl carrier protein	1.197	0.25979756	0.003187825	0.018521666	no	up	mRNA	182.16	188.07	188.72	224	209.04	205.78	186.317	212.94	COG0511	I:Lipid transport and metabolism	GO:0006633;GO:0006633;GO:0009317;GO:0009317;GO:0003989;GO:0003989	BP:fatty acid biosynthetic process;BP:fatty acid biosynthetic process;CC:acetyl-CoA carboxylase complex;CC:acetyl-CoA carboxylase complex;MF:acetyl-CoA carboxylase activity;MF:acetyl-CoA carboxylase activity;
BAB_RS16280	-	23S rRNA (adenine(2503)-C(2))-methyltransferase RlmN	1.197	0.259528324	0.009780532	0.039085633	no	up	mRNA	126.66	113.96	121.08	154.31	126.55	127	120.567	135.95333	COG0820	J:Translation, ribosomal structure and biogenesis	GO:0070475;GO:0030488;GO:0070475;GO:0030488;GO:0070475;GO:0005737;GO:0005737;GO:0005737;GO:0051539;GO:0046872;GO:0070040;GO:0019843;GO:0002935;GO:0000049;GO:0000049;GO:0051539;GO:0070040;GO:0019843;GO:0002935;GO:0046872;GO:0000049;GO:0051539;GO:0070040;GO:0019843;GO:0002935;GO:0046872	BP:rRNA base methylation;BP:tRNA methylation;BP:rRNA base methylation;BP:tRNA methylation;BP:rRNA base methylation;CC:cytoplasm;CC:cytoplasm;CC:cytoplasm;MF:4 iron, 4 sulfur cluster binding;MF:metal ion binding;MF:rRN;MF:rRNA binding;MF:tRN;MF:tRNA binding;MF:tRNA binding;MF:4 iron, 4 sulfur cluster binding;MF:rRN;MF:rRNA binding;MF:tRN;MF:metal ion binding;MF:tRNA binding;MF:4 iron, 4 sulfur cluster binding;MF:rRN;MF:rRNA binding;MF:tRN;MF:metal ion binding;
BAB_RS29440	-	-	1.197	0.25978927	0.082541518	0.180657969	no	up	mRNA	8.23	7.47	9.56	8.69	9.14	10.84	8.42	9.5566667	------	------	------	------
BAB_RS28280	-	hypothetical protein	1.196	0.257683847	0.003818817	0.020726114	no	up	mRNA	204.19	201.85	209.57	208.41	247.78	241.05	205.203	232.41333	ENOG4111G65	S:Function unknown	------	------
BAB_RS23305	-	ribose-phosphate pyrophosphokinase	1.196	0.25774785	0.017114242	0.057279227	no	up	mRNA	58.86	48.06	53.9	67.59	55.93	57.35	53.6067	60.29	COG0462	F:Nucleotide transport and metabolism	GO:0006015;GO:0009116;GO:0009165;GO:0009156;GO:0009156;GO:0009165;GO:0009116;GO:0006015;GO:0009156;GO:0009165;GO:0009116;GO:0006015;GO:0005737;GO:0005737;GO:0005737;GO:0005524;GO:0016301;GO:0000287;GO:0004749;GO:0016787;GO:0016301;GO:0016757;GO:0000287;GO:0004749;GO:0005524;GO:0016301;GO:0000287;GO:0004749;GO:0005524	BP:5-phosphoribose 1-diphosphate biosynthetic process;BP:nucleoside metabolic process;BP:nucleotide biosynthetic process;BP:ribonucleoside monophosphate biosynthetic process;BP:ribonucleoside monophosphate biosynthetic process;BP:nucleotide biosynthetic process;BP:nucleoside metabolic process;BP:5-phosphoribose 1-diphosphate biosynthetic process;BP:ribonucleoside monophosphate biosynthetic process;BP:nucleotide biosynthetic process;BP:nucleoside metabolic process;BP:5-phosphoribose 1-diphosphate biosynthetic process;CC:cytoplasm;CC:cytoplasm;CC:cytoplasm;MF:ATP binding;MF:kinase activity;MF:magnesium ion binding;MF:ribose phosphate diphosphokinase activity;MF:hydrolase activity;MF:kinase activity;MF:transferase activity, transferring glycosyl groups;MF:magnesium ion binding;MF:ribose phosphate diphosphokinase activity;MF:ATP binding;MF:kinase activity;MF:magnesium ion binding;MF:ribose phosphate [illegible]
BAB_RS29355	-	type I DNA topoisomerase	1.195	0.257523288	0.000229341	0.003189926	no	up	mRNA	98.16	92.87	101.26	111.48	108.11	109.1	97.43	109.56333	COG0550	L:Replication, recombination and repair	GO:0006265;GO:0006265;GO:0005694;GO:0005694;GO:0003917;GO:0003677;GO:0046872;GO:0005524;GO:0003677;GO:0003917;GO:0016787;GO:0046872	BP:DNA topological change;BP:DNA topological change;CC:chromosome;CC:chromosome;MF:DNA topoisomerase type I activity;MF:DNA binding;MF:metal ion binding;MF:ATP binding;MF:DNA binding;MF:DNA topoisomerase type I activity;MF:hydrolase activity;MF:metal ion binding;
BAB_RS19225	-	dihydroorotase	1.195	0.257286788	0.002276825	0.014543615	no	up	mRNA	51.85	44.78	51.47	58.51	55	52.81	49.3667	55.44	COG0044	S:Function unknown	GO:0004151;GO:0046872	MF:dihydroorotase activity;MF:metal ion binding;
BAB_RS24260	-	PaaI family thioesterase	1.195	0.256950413	0.003624353	0.020008671	no	up	mRNA	35.39	31.48	37.85	40.57	39.2	38.09	34.9067	39.286667	COG2050	K:Transcription	------	------
BAB_RS25740	-	ribosomal RNA small subunit methyltransferase G	1.195	0.256937252	0.021592177	0.068215991	no	up	mRNA	42.95	37.81	40.25	48.36	41.73	46.94	40.3367	45.676667	COG0357	M:Cell wall/membrane/envelope biogenesis	GO:0005737;GO:0008649;GO:0070043	CC:cytoplasm;MF:rRNA methyltransferase activity;MF:rRN;
BAB_RS17235	-	hypothetical protein	1.195	0.256965764	0.5775995	0.718477427	no	up	mRNA	1.62	0.96	2.04	0.97	2.19	2.08	1.54	1.7466667	------	------	------	------
BAB_RS20295	-	FADH(2)-oxidizing methylenetetrahydrofolate--tRNA-(uracil(54)-C(5))-methyltransferase TrmFO	1.194	0.255371767	0.009359048	0.037920077	no	up	mRNA	36.42	30.78	36.07	41.83	34.93	39.1	34.4233	38.62	COG1206	J:Translation, ribosomal structure and biogenesis	GO:0008033;GO:0008033;GO:0005737;GO:0005737;GO:0005737;GO:0050660;GO:0047151;GO:0030698;GO:0050660;GO:0047151;GO:0047151;GO:0050660;GO:0030698	BP:tRNA processing;BP:tRNA processing;CC:cytoplasm;CC:cytoplasm;CC:cytoplasm;MF:flavin adenine dinucleotide binding;MF:methylenetetrahydrofolate-tRNA;MF:5,10-methylenetetrahydrofolate-dependent tRN;MF:flavin adenine dinucleotide binding;MF:methylenetetrahydrofolate-tRNA;MF:methylenetetrahydrofolate-tRNA;MF:flavin adenine dinucleotide binding;MF:5,10-methylenetetrahydrofolate-dependent tRN;
BAB_RS29050	-	DUF3775 domain-containing protein	1.194	0.255897737	0.011027733	0.042484541	no	up	mRNA	657	634.59	664.87	640.67	786.97	821.52	652.153	749.72	ENOG41124H2	S:Function unknown	GO:0016021	CC:integral component of membrane;
BAB_RS30850	-	transporter	1.194	0.255860309	0.025558842	0.076249358	no	up	mRNA	15.38	15.59	16.3	15.19	19.78	18.18	15.7567	17.716667	COG2244	M:Cell wall/membrane/envelope biogenesis	GO:0016021;GO:0016021;GO:0016020	CC:integral component of membrane;CC:integral component of membrane;CC:membrane;
BAB_RS28910	-	FAD-binding oxidoreductase	1.194	0.25575618	0.031459875	0.08812605	no	up	mRNA	121.34	118.8	170.84	149.92	161.39	151.21	136.993	154.17333	ENOG410XNXD	S:Function unknown	GO:0016491	MF:oxidoreductase activity;
BAB_RS28135	-	glycerol-3-phosphate dehydrogenase	1.193	0.255173868	0.001038359	0.008510832	no	up	mRNA	54.35	50.73	53.52	59.49	58.96	59.86	52.8667	59.436667	COG0578	G:Carbohydrate transport and metabolism	GO:0006072;GO:0009331;GO:0052591	BP:glycerol-3-phosphate metabolic process;CC:glycerol-3-phosphate dehydrogenase complex;MF:sn-glycerol-3-phosphat;
BAB_RS21460	-	2-dehydro-3-deoxyphosphooctonate aldolase	1.193	0.254980432	0.004075583	0.021573062	no	up	mRNA	116.98	106.43	105.14	129.89	118.78	121.17	109.517	123.28	COG2877	M:Cell wall/membrane/envelope biogenesis	GO:0019294;GO:0009103;GO:0019294;GO:0005737;GO:0005737;GO:0008676;GO:0016746;GO:0008676	BP:keto-3-deoxy-D-manno-octulosonic acid biosynthetic process;BP:lipopolysaccharide biosynthetic process;BP:keto-3-deoxy-D-manno-octulosonic acid biosynthetic process;CC:cytoplasm;CC:cytoplasm;MF:3-deoxy-8-phosphooctulonate synthase activity;MF:transferase activity, transferring acyl groups;MF:3-deoxy-8-phosphooctulonate synthase activity;
BAB_RS16050	-	cytidylate kinase	1.193	0.254804793	0.074248102	0.167645222	no	up	mRNA	35.58	44.97	53.66	54.81	51.17	47.24	44.7367	51.073333	COG0283	F:Nucleotide transport and metabolism	GO:0006220;GO:0006220;GO:0006220;GO:0006220;GO:0005737;GO:0005737;GO:0005737;GO:0005737;GO:0005524;GO:0004127;GO:0016491;GO:0005524;GO:0004127;GO:0005524;GO:0004127;GO:0004127;GO:0005524;GO:0016491	BP:pyrimidine nucleotide metabolic process;BP:pyrimidine nucleotide metabolic process;BP:pyrimidine nucleotide metabolic process;BP:pyrimidine nucleotide metabolic process;CC:cytoplasm;CC:cytoplasm;CC:cytoplasm;CC:cytoplasm;MF:ATP binding;MF:cytidylate kinase activity;MF:oxidoreductase activity;MF:ATP binding;MF:cytidylate kinase activity;MF:ATP binding;MF:cytidylate kinase activity;MF:cytidylate kinase activity;MF:ATP binding;MF:oxidoreductase activity;
BAB_RS16250	-	hypothetical protein	1.193	0.254962476	0.134625804	0.257932443	no	up	mRNA	9.6	12.09	12.61	15.35	11.8	12.06	11.4333	13.07	COG4544	S:Function unknown	------	------
BAB_RS19795	-	30S ribosomal protein S9	1.192	0.253368812	0.004749492	0.024107612	no	up	mRNA	887.3	731.29	781.11	982.44	855.79	886.01	799.9	908.08	COG0103	J:Translation, ribosomal structure and biogenesis	GO:0006412;GO:0005840;GO:0003735	BP:translation;CC:ribosome;MF:structural constituent of ribosome;
BAB_RS32430	-	hypothetical protein	1.192	0.253247643	0.01857683	0.061343632	no	up	mRNA	38.22	34.15	44.7	48.83	41.1	41.43	39.0233	43.786667	------	------	------	------
BAB_RS26615	gltD	glutamate synthase subunit beta	1.191	0.252754897	0.000292715	0.003702732	no	up	mRNA	82.93	75.27	85.15	88.57	93.01	90.89	81.1167	90.823333	COG0493	E:Amino acid transport and metabolism	GO:0006537;GO:0006537;GO:0004031;GO:0102798;GO:0102797;GO:0051536;GO:0004355;GO:0016639;GO:0004355;GO:0051536	BP:glutamate biosynthetic process;BP:glutamate biosynthetic process;MF:aldehyde oxidase activity;MF:old GO;MF:old GO;MF:iron-sulfur cluster binding;MF:glutamate synthas;MF:oxidoreductase activity, acting on the CH-NH2 group of donors, NAD or NADP as acceptor;MF:glutamate synthas;MF:iron-sulfur cluster binding;

BAB_RS18335	-	sensor domain-containing phosphodiesterase	1.191	0.252590492	0.000629522	0.006166629	no	up	mRNA	137.05	128.83	134.36	144.18	153.03	151.46	133.413	149.55667	COG2202;ENOG410XNMH	K:Transcription;	[illegible]	[illegible]
BAB_RS25605	-	acetyl-CoA carboxylase carboxyl transferase subunit alpha	1.191	0.252243107	0.000813717	0.007143364	no	up	mRNA	217.75	206.07	212.62	248.14	233.2	234.28	212.147	238.54	COG0825	I:Lipid transport and metabolism	[illegible]	[illegible]
BAB_RS26755	-	DUF882 domain-containing protein	1.191	0.252268564	0.001985229	0.013044049	no	up	mRNA	194.31	203.29	228.7	234.2	223.02	244.54	208.767	233.92	COG3108	E:Amino acid transport and metabolism	[illegible]	[illegible]
BAB_RS16790	-	methylated-DNA--[protein]-cysteine S-methyltransferase	1.19	0.251285783	0.001428299	0.010289514	no	up	mRNA	284.81	282	303.98	312.4	351.7	315.9	290.263	326.66667	COG0350	L:Replication, recombination and repair	[illegible]	[illegible]
BAB_RS29500	-	invasion associated locus B family protein	1.19	0.251086334	0.003307073	0.018839761	no	up	mRNA	267.13	228.39	243.36	292.02	275.61	260.12	246.293	275.91667	COG5342	Q:Secondary metabolites biosynthesis, transport and catabolism	------	------
BAB_RS20445	-	tyrosine--tRNA ligase	1.19	0.251007584	0.003676401	0.020191928	no	up	mRNA	151.53	137.25	138.02	170.23	153.18	155.29	142.267	159.56667	COG0162	J:Translation, ribosomal structure and biogenesis	[illegible]	[illegible]
BAB_RS28765	-	alcohol dehydrogenase	1.19	0.251314404	0.127224135	0.24595135	no	up	mRNA	5.36	6.92	5.94	6.18	7.42	6.98	6.07333	6.86	COG1454	C:Energy production and conversion	[illegible]	[illegible]
BAB_RS26160	-	hypothetical protein	1.189	0.249471694	0.001176532	0.009356928	no	up	mRNA	197.84	182.2	218.62	211.12	235.31	223.12	199.553	223.18333	ENOG410XVFM	S:Function unknown	------	------
BAB_RS30430	-	glutamate decarboxylase	1.189	0.249655597	0.001291131	0.009760952	no	up	mRNA	102.38	88.92	104.65	111.24	106.19	113.63	98.65	110.35333	COG0076	E:Amino acid transport and metabolism	[illegible]	[illegible]
BAB_RS21370	-	sigma-54-dependent Fis family transcriptional regulator	1.189	0.249248805	0.002528059	0.01577214	no	up	mRNA	179.08	176.94	185.34	218.71	189.47	198.73	180.453	202.30333	COG2204	T:Signal transduction mechanisms	[illegible]	[illegible]
BAB_RS28370	-	mannonate dehydratase	1.188	0.248001008	0.011855795	0.044448856	no	up	mRNA	29.97	30.92	34.88	36.35	32.68	38.54	31.9233	35.856667	COG1312	G:Carbohydrate transport and metabolism	[illegible]	[illegible]
BAB_RS31635	-	D-xylose ABC transporter substrate-binding protein	1.188	0.248300071	0.036037636	0.097547536	no	up	mRNA	48.38	37.41	39.85	52.06	45.39	42.45	41.88	46.633333	COG4213	S:Function unknown	[illegible]	[illegible]
BAB_RS21485	-	peptidyl-prolyl cis-trans isomerase	1.187	0.246889943	0.000305394	0.003818022	no	up	mRNA	167.87	155.71	172.77	187.38	181.97	184.92	165.45	184.75667	COG0760	G:Carbohydrate transport and metabolism	[illegible]	[illegible]
BAB_RS20610	-	peptidoglycan-binding protein	1.187	0.247702295	0.000394809	0.004435386	no	up	mRNA	160.37	155.28	168.8	179.62	176.12	185.8	161.483	180.51333	COG2989	C:Energy production and conversion	------	------
BAB_RS23620	-	DegT/DnrJ/EryC1/StrS aminotransferase family protein	1.187	0.247345787	0.001640304	0.011432311	no	up	mRNA	85.61	74.62	87.32	95.43	92.97	87.86	82.5167	92.086667	COG0399	S:Function unknown	[illegible]	[illegible]
BAB_RS20075	-	ribulose-phosphate 3-epimerase	1.187	0.247044807	0.008550667	0.035772517	no	up	mRNA	57.65	53.79	64.74	65.19	68.11	65.11	58.7267	66.136667	COG0036	G:Carbohydrate transport and metabolism	[illegible]	[illegible]
BAB_RS29465	-	glutamine--fructose-6-phosphate aminotransferase	1.186	0.246537145	0.000540129	0.005580174	no	up	mRNA	124.21	113.74	120.22	131.59	134.29	133.98	119.39	133.28667	COG0449	M:Cell wall/membrane/envelope biogenesis	[illegible]	[illegible]
BAB_RS24990	-	2-octaprenyl-6-methoxyphenyl hydroxylase	1.186	0.246667973	0.000667237	0.006361517	no	up	mRNA	112.54	107.09	112.17	123.53	123.7	123.89	110.6	123.70667	COG0654	I:Lipid transport and metabolism	[illegible]	[illegible]
BAB_RS25965	-	acetyl-CoA carboxylase carboxyl transferase subunit beta	1.186	0.246077623	0.001974897	0.013029452	no	up	mRNA	614.27	530.29	544.67	638.68	627.1	626.42	563.077	630.73333	COG0777	I:Lipid transport and metabolism	[illegible]	[illegible]
BAB_RS23615	-	hypothetical protein	1.185	0.244583841	0.001222345	0.009579011	no	up	mRNA	126.67	114.11	125.69	139.5	129.01	140.27	122.157	136.26	ENOG4111N16;ENOG4111QXW	S:Function unknown;	------	------
BAB_RS17565	-	dihydroorotate dehydrogenase (quinone)	1.185	0.244581139	0.00596111	0.028166245	no	up	mRNA	33.52	29.14	31.51	34.85	35.73	34.33	31.39	34.97	COG0167	F:Nucleotide transport and metabolism	[illegible]	[illegible]
BAB_RS22745	-	heme-degrading domain-containing protein	1.185	0.245417995	0.007198532	0.031857965	no	up	mRNA	77.37	69.73	77.26	81.6	80.88	90.06	74.7867	84.18	COG4702	S:Function unknown	------	------
BAB_RS18750	-	superoxide dismutase	1.185	0.245155584	0.008526591	0.035718303	no	up	mRNA	680.36	551.21	584.12	631.08	716.3	681.29	605.23	676.22333	COG0605	S:Function unknown	[illegible]	[illegible]
BAB_RS22835	-	UDP-N-acetylmuramate--L-alanine ligase	1.184	0.244086002	0.000706334	0.006540208	no	up	mRNA	89.04	85.91	93.3	99.18	100.04	100.26	89.4167	99.826667	COG0773	M:Cell wall/membrane/envelope biogenesis	[illegible]	[illegible]
BAB_RS19550	-	hypothetical protein	1.184	0.243333929	0.002721032	0.0164336	no	up	mRNA	5291.27	5187.65	4408.96	5463.46	6076.42	5980.57	4962.63	5840.15	ENOG410ZCCB	S:Function unknown	------	------
BAB_RS31040	-	SelT/SelW/SelH family protein	1.184	0.244000358	0.033947868	0.093066979	no	up	mRNA	20.63	20.11	25.16	22.02	27.95	23.5	21.9667	24.49	COG3526	O:Posttranslational modification, protein turnover, chaperones	------	------
BAB_RS20180	-	sel1 repeat family protein	1.183	0.242849534	0.001250825	0.009637655	no	up	mRNA	287.97	250.72	277.63	308.25	310.67	289.83	272.107	302.91667	COG0790	L:Replication, recombination and repair	------	------
BAB_RS28945	-	sulfurtransferase FdhD	1.183	0.242084529	0.053816391	0.131592134	no	up	mRNA	67.85	60.8	89.8	79.89	90.25	72.44	72.8167	80.86	COG1526	C:Energy production and conversion	[illegible]	[illegible]
BAB_RS29205	-	3-oxoadipate CoA-transferase subunit A	1.183	0.242394988	0.074270357	0.167577709	no	up	mRNA	36.67	26.91	36.3	42.02	33.65	35.66	33.2933	37.11	COG1788	I:Lipid transport and metabolism	[illegible]	[illegible]
BAB_RS32165	-	hypothetical protein	1.183	0.242772749	0.266772361	0.422444355	no	up	mRNA	14.18	14.68	21.87	22.44	20.26	15.37	16.91	19.356667	------	------	------	------
BAB_RS27385	-	MFS transporter	1.182	0.241253988	0.0155827	0.053490615	no	up	mRNA	19.47	16.05	18.86	19.17	20.46	20.7	18.1267	20.11	ENOG410ZVI6	G:Carbohydrate transport and metabolism	[illegible]	[illegible]
BAB_RS22950	-	energy-dependent translational throttle protein EttA	1.182	0.241045061	0.024606612	0.074656321	no	up	mRNA	151.18	119.35	120.77	155.16	136.11	143.75	130.433	145.00667	COG0488	S:Function unknown	[illegible]	[illegible]
BAB_RS17250	-	toll/interleukin-1 receptor domain-containing protein	1.182	0.241075339	0.024970671	0.075404856	no	up	mRNA	40.32	40.74	51.89	53.41	48.96	46.16	44.3167	49.51	ENOG4111Q5J	S:Function unknown	[illegible]	[illegible]
BAB_RS17005	-	hypothetical protein	1.182	0.241112335	0.451393919	0.615589415	no	up	mRNA	3.55	5.26	3.03	4.26	5.24	3.72	3.94667	4.4066667	COG0446	S:Function unknown	------	------
BAB_RS17605	-	polysaccharide deacetylase family protein	1.181	0.240275056	0.013270115	0.047799191	no	up	mRNA	125.33	109.4	121.73	146.1	132.62	118.53	118.82	132.41667	COG0726	G:Carbohydrate transport and metabolism	[illegible]	[illegible]
BAB_RS18210	-	30S ribosomal protein S18	1.181	0.240243361	0.0333376	0.091943099	no	up	mRNA	1648.4	1527.04	1452.73	1887.92	1808.76	1805.13	1542.72	1833.9367	COG0238	J:Translation, ribosomal structure and biogenesis	[illegible]	[illegible]
BAB_RS26660	-	type IV secretion system protein VirB6	1.181	0.240357452	0.067107762	0.15456433	no	up	mRNA	11.52	10.85	13.66	12.38	12.68	15.27	12.01	13.443333	COG3704	U:Intracellular trafficking, secretion, and vesicular transport	[illegible]	[illegible]
BAB_RS27020	-	transcriptional regulator	1.18	0.23935136	0.001715154	0.011725087	no	up	mRNA	60.96	56.01	61.86	65.45	64.65	68.79	59.61	66.296667	ENOG410Y363	K:Transcription	[illegible]	[illegible]
BAB_RS25350	-	twin transmembrane helix small protein	1.18	0.239182229	0.623427084	0.751903611	no	up	mRNA	65.41	60.53	24.52	38.63	79.4	43.58	50.1533	53.87	ENOG410XVZX;ENOG410ZRJ9	S:Function unknown;	[illegible]	[illegible]
BAB_RS29125	-	membrane dipeptidase	1.179	0.23752584	0.007109881	0.031683837	no	up	mRNA	40.14	35.63	37.87	43.84	42.66	39.49	37.88	41.996667	COG2355	E:Amino acid transport and metabolism	[illegible]	[illegible]
BAB_RS33110	-	hypothetical protein	1.179	0.237237617	0.040200781	0.106046889	no	up	mRNA	153.03	128.21	183.78	149.44	191.81	172.15	155.007	171.13333	------	------	------	------
BAB_RS21800	-	DNA-directed RNA polymerase subunit alpha	1.178	0.236262889	0.001853188	0.01240478	no	up	mRNA	480.48	416.46	462.16	524.28	481.21	502.67	453.033	502.72	COG0202	K:Transcription	[illegible]	[illegible]
BAB_RS22215	-	cobalamin biosynthesis protein	1.178	0.236673111	0.002200864	0.014199549	no	up	mRNA	58.13	52.27	63.4	64.75	63.65	64.3	57.9333	64.233333	COG1270	H:Coenzyme transport and metabolism	[illegible]	[illegible]
BAB_RS21280	-	peptidyl-prolyl cis-trans isomerase	1.178	0.236530582	0.002254392	0.014429005	no	up	mRNA	360.09	317.33	326.39	355.93	379.89	385.88	334.603	373.9	COG0652	O:Posttranslational modification, protein turnover, chaperones	[illegible]	[illegible]
BAB_RS23115	-	NUDIX domain-containing protein	1.178	0.235933618	0.009013292	0.037127828	no	up	mRNA	111.99	112.61	118.68	126.98	137.76	119.8	114.427	128.18	COG0494	S:Function unknown	[illegible]	[illegible]
BAB_RS27510	-	sugar ABC transporter ATP-binding protein	1.178	0.236265323	0.349678406	0.51513834	no	up	mRNA	1.74	1.69	2.97	2.79	2.47	1.94	2.13333	2.4	COG1129	G:Carbohydrate transport and metabolism	[illegible]	[illegible]
BAB_RS17035	-	sugar ABC transporter substrate-binding protein	1.177	0.234536009	0.04179266	0.108816706	no	up	mRNA	867.76	748.87	841.36	1072.36	864.62	785.29	819.33	907.42333	COG1653	G:Carbohydrate transport and metabolism	[illegible]	[illegible]
BAB_RS24750	-	50S ribosomal protein L27	1.176	0.233597564	0.000682726	0.006451761	no	up	mRNA	5207.63	4616.25	3921.32	5315.56	5212.01	5360.38	4581.73	5295.9833	COG0211	J:Translation, ribosomal structure and biogenesis	[illegible]	[illegible]
BAB_RS27550	-	GTP-binding protein	1.176	0.233656106	0.002391487	0.015215538	no	up	mRNA	179.36	156.15	166.59	187.09	190.49	178.77	167.367	185.45	COG0523	S:Function unknown	------	------
BAB_RS26040	-	50S ribosomal protein L20	1.176	0.233882924	0.007331982	0.032182594	no	up	mRNA	746.82	663.69	695.32	856.06	719.95	791.68	701.943	789.23	COG0292	J:Translation, ribosomal structure and biogenesis	[illegible]	[illegible]
BAB_RS33295	-	hypothetical protein	1.176	0.233441591	0.025275431	0.076110552	no	up	mRNA	18.96	19.49	25.73	23.77	23.44	23.92	21.3933	23.71	------	------	[illegible]	[illegible]
BAB_RS23525	-	dipeptide ABC transporter ATP-binding protein	1.176	0.234443678	0.034822343	0.094817109	no	up	mRNA	20.87	20.59	24.83	24.49	24.15	25.41	22.0967	24.683333	COG4608	E:Amino acid transport and metabolism	[illegible]	[illegible]
BAB_RS27300	-	cytochrome b	1.175	0.233075939	0.002928619	0.017329013	no	up	mRNA	292.42	259.53	285.84	289.44	316.14	328.03	279.263	311.20333	COG3038	C:Energy production and conversion	[illegible]	[illegible]
BAB_RS24330	-	N-formylglutamate amidohydrolase	1.175	0.233261443	0.004895387	0.024538034	no	up	mRNA	588.96	583.84	699.94	647.13	703.23	726.43	624.247	692.26333	COG3931	E:Amino acid transport and metabolism	[illegible]	[illegible]
BAB_RS27930	-	formyltetrahydrofolate deformylase	1.175	0.232777559	0.107664966	0.217701407	no	up	mRNA	19.11	21.91	21.61	27.24	23.13	19.41	20.8767	23.26	COG0788	F:Nucleotide transport and metabolism	[illegible]	[illegible]
BAB_RS26610	-	glutamate synthase large subunit	1.174	0.231147104	0.000512028	0.0053241	no	up	mRNA	126.03	119.42	134.27	137.79	140.76	140.17	126.573	139.57333	COG0070	E:Amino acid transport and metabolism	[illegible]	[illegible]
BAB_RS18735	-	L%2CD-transpeptidase	1.174	0.231567148	0.001706784	0.01174282	no	up	mRNA	282.88	245.83	273.24	305.56	298.17	282.88	267.317	295.53667	COG1376	S:Function unknown	[illegible]	[illegible]
BAB_RS27630	-	homoserine dehydrogenase	1.174	0.231123044	0.008569367	0.03580413	no	up	mRNA	29.15	26.37	31.21	34.29	29.73	31.74	28.91	31.92	COG4091	E:Amino acid transport and metabolism	[illegible]	[illegible]
BAB_RS31660	-	4-hydroxy-2-oxoheptanedioate aldolase	1.174	0.23161032	0.221458026	0.369633578	no	up	mRNA	5.17	6.17	5.7	5.45	6.63	6.98	5.68	6.3533333	COG3836	S:Function unknown	[illegible]	[illegible]
BAB_RS32230	-	hypothetical protein	1.173	0.230297219	0.055964047	0.135299085	no	up	mRNA	15.52	14.77	20.51	20.72	17.9	17.58	16.9333	18.733333	------	------	------	------
BAB_RS31195	-	peptide-methionine (S)-S-oxide reductase	1.172	0.228401501	0.001261802	0.009675824	no	up	mRNA	146.24	138.37	155.87	166.69	165.52	153.59	146.827	161.93333	COG0225	O:Posttranslational modification, protein turnover, chaperones	[illegible]	[illegible]
BAB_RS22780	-	aminopeptidase P family protein	1.172	0.228782529	0.004983433	0.024862998	no	up	mRNA	74.83	70.35	70.26	74.46	81.43	81.91	71.8133	79.266667	COG0006	J:Translation, ribosomal structure and biogenesis	[illegible]	[illegible]
BAB_RS28240	-	type I restriction endonuclease subunit R	1.172	0.228976543	0.01920223	0.062763747	no	up	mRNA	54.65	51.23	54.75	67.29	56.25	53.49	53.5433	59.01	COG0610	V:Defense mechanisms	[illegible]	[illegible]
BAB_RS32490	-	hypothetical protein	1.172	0.228593352	0.024090799	0.073577698	no	up	mRNA	16.99	14.17	20	19.66	17.72	18.82	17.0533	18.733333	------	------	[illegible]	[illegible]
BAB_RS21755	-	isocitrate dehydrogenase (NADP(+))	1.171	0.228298438	0.006092912	0.028454253	no	up	mRNA	288.35	236.33	259.95	303.54	278.89	283.09	261.543	288.50667	COG0538	C:Energy production and conversion	[illegible]	[illegible]
BAB_RS21590	-	30S ribosomal protein S2	1.171	0.227346281	0.006667324	0.030429138	no	up	mRNA	738.16	623.61	678.69	799.48	750.9	701.97	680.153	750.78333	COG0052	J:Translation, ribosomal structure and biogenesis	[illegible]	[illegible]
BAB_RS25315	-	3-hydroxybutyryl-CoA dehydrogenase	1.171	0.227474595	0.006758332	0.030540818	no	up	mRNA	276.63	233.37	243.46	259.81	288.05	284.38	251.153	277.41333	COG1250	I:Lipid transport and metabolism	[illegible]	[illegible]
BAB_RS26775	-	molybdate ABC transporter substrate-binding protein	1.171	0.227728518	0.010924826	0.042189262	no	up	mRNA	79.71	73.98	81.75	90.5	91.1	78.9	78.48	86.833333	COG0725	P:Inorganic ion transport and metabolism	[illegible]	[illegible]
BAB_RS26870	-	phosphogluconate dehydrogenase (NADP(+)-dependent%2C decarboxylating)	1.17	0.226164717	0.001271681	0.009682251	no	up	mRNA	190.19	169.19	182.13	199.02	199.02	197.76	180.503	198.6	COG0362	G:Carbohydrate transport and metabolism	[illegible]	[illegible]
BAB_RS29045	-	aldehyde dehydrogenase family protein	1.17	0.226404318	0.002601192	0.015949677	no	up	mRNA	376.86	340.36	360.56	376.29	392.67	419.25	359.26	396.07	COG1012	P:Inorganic ion transport and metabolism	[illegible]	[illegible]
BAB_RS27555	-	creatininase	1.17	0.22642046	0.009264228	0.037678436	no	up	mRNA	110.87	101.03	105.41	105.42	121.52	123.43	105.77	116.79	COG1402	S:Function unknown	[illegible]	[illegible]
BAB_RS27285	-	bifunctional riboflavin kinase/FAD synthetase	1.17	0.225918566	0.015248671	0.052625111	no	up	mRNA	40.16	36.22	39.28	45.52	39.17	42.88	38.5533	42.523333	COG0196	H:Coenzyme transport and metabolism	[illegible]	[illegible]
BAB_RS26995	-	glycosyltransferase	1.17	0.226839628	0.019060486	0.062427465	no	up	mRNA	100.38	81.99	98.57	108.33	92.55	108.96	93.6467	103.28	COG0463	S:Function unknown	[illegible]	[illegible]
BAB_RS31540	-	flagellar biosynthesis protein FlhA	1.17	0.226073607	0.600018847	0.735543898	no	up	mRNA	0.48	1.03	0.45	1.01	0.67	0.54	0.65333	0.74	COG1298	N:Cell motility	[illegible]	[illegible]
BAB_RS30975	-	membrane protein insertase YidC	1.169	0.224660648	0.009476209	0.038011312	no	up	mRNA	157.32	127.9	141.74	166.59	149.45	152.94	142.32	156.32667	COG0706	U:Intracellular trafficking, secretion, and vesicular transport	[illegible]	[illegible]
BAB_RS32195	-	hypothetical protein	1.169	0.225591636	0.049947324	0.124307322	no	up	mRNA	14.81	13.93	18.31	17.51	18.08	16.35	15.6833	17.313333	COG0616	S:Function unknown	------	------
BAB_RS31240	-	ABC transporter permease	1.169	0.225649587	0.201201077	0.344227402	no	up	mRNA	17.85	15.19	21.23	23.1	21.16	15.86	18.09	20.04	COG1173	S:Function unknown	[illegible]	[illegible]
BAB_RS29405	gatA	Asp-tRNA(Asn)/Glu-tRNA(Gln) amidotransferase GatCAB subunit A	1.168	0.224242043	0.000631947	0.006171568	no	up	mRNA	190.72	179.21	196.96	211.14	208.65	204	188.963	207.93	COG0154	J:Translation, ribosomal structure and biogenesis	[illegible]	[illegible]
BAB_RS31065	-	undecaprenyl-diphosphatase	1.168	0.22463639	0.003291209	0.018816111	no	up	mRNA	43.72	41.23	46.58	49.59	45.84	49.28	43.8433	48.236667	COG1968	V:Defense mechanisms	[illegible]	[illegible]
BAB_RS20025	-	hypothetical protein	1.168	0.224454282	0.005245303	0.025769355	no	up	mRNA	145.47	136.26	144.22	159.33	161.87	153.32	141.983	158.17333	ENOG4111VSG	S:Function unknown	------	------
BAB_RS20130	-	glycerol-3-phosphatase	1.168	0.22364151	0.007522304	0.032749544	no	up	mRNA	58.15	58.98	63.61	62.55	68.58	68.55	60.2467	66.56	COG0637	P:Inorganic ion transport and metabolism	[illegible]	[illegible]
BAB_RS33075	-	hypothetical protein	1.168	0.224108829	0.150852712	0.279521202	no	up	mRNA	13.42	11.51	17.97	15.52	15.53	16.62	14.3	15.89	COG3108	E:Amino acid transport and metabolism	------	------
BAB_RS22260	-	membrane protein	1.167	0.222455576	0.078662121	0.17430441	no	up	mRNA	24.6	20.29	23.89	28.91	23.81	22.81	22.9267	25.176667	COG0730	S:Function unknown	[illegible]	[illegible]
BAB_RS31675	-	3%2C4-dihydroxyphenylacetate 2%2C3-dioxygenase	1.167	0.223136915	0.281096945	0.437367789	no	up	mRNA	4.18	3.41	4.28	5.37	3.91	3.78	3.95667	4.3533333	COG0346	Q:Secondary metabolites biosynthesis, transport and catabolism	[illegible]	[illegible]
BAB_RS17260	-	DNA gyrase inhibitor YacG	1.167	0.222252047	0.369788235	0.535434701	no	up	mRNA	88	106.06	62.65	79.02	110.42	108.3	85.57	99.246667	COG3024;ENOG410XUEH	S:Function unknown;	[illegible]	[illegible]
BAB_RS28010	-	ATP-dependent protease	1.166	0.222030816	0.006998226	0.031404052	no	up	mRNA	265.98	235.4	244.87	272.7	294	255.1	248.75	273.93333	COG2802	O:Posttranslational modification, protein turnover, chaperones	[illegible]	[illegible]
BAB_RS27670	-	ABC transporter permease	1.166	0.221426133	0.013069021	0.04734021	no	up	mRNA	28.69	23.48	27.3	29.58	29.09	28.28	26.49	28.983333	COG2011	P:Inorganic ion transport and metabolism	[illegible]	[illegible]
BAB_RS19175	-	AraC family transcriptional regulator	1.166	0.221975123	0.022915682	0.070932647	no	up	mRNA	89.25	102.89	114.63	112.37	116.79	110.04	102.257	113.06667	ENOG410XRKS	K:Transcription	[illegible]	[illegible]
BAB_RS16170	-	DUF1775 domain-containing protein	1.166	0.221883697	0.147184806	0.274784882	no	up	mRNA	19.74	22.96	20.28	18.39	26.78	24.3	20.9933	23.156667	COG4549	S:Function unknown	------	------
BAB_RS26960	-	flagellar motor stator protein MotA	1.166	0.222143127	0.734811093	0.837215617	no	up	mRNA	0.49	1.51	0.21	0.99	0.58	0.95	0.73667	0.84	COG1291	N:Cell motility	[illegible]	[illegible]
BAB_RS25005	-	succinate dehydrogenase flavoprotein subunit	1.165	0.219718563	0.001567967	0.011072256	no	up	mRNA	409.22	372.69	417.94	453.88	421.6	439.25	399.95	438.24333	COG1053	C:Energy production and conversion	[illegible]	[illegible]
BAB_RS24545	-	transaldolase	1.165	0.220285053	0.002451478	0.015444314	no	up	mRNA	470.11	401.07	441.67	472.46	483	484.33	437.617	479.93	COG0176	G:Carbohydrate transport and metabolism	[illegible]	[illegible]
BAB_RS20605	-	fumarate hydratase	1.165	0.220669558	0.009834973	0.039205667	no	up	mRNA	47.7	39.51	43.25	49.14	47.37	46.26	43.4867	47.59	COG1951	C:Energy production and conversion	[illegible]	[illegible]
BAB_RS32640	-	hypothetical protein	1.165	0.219986501	0.026098671	0.077428467	no	up	mRNA	13.76	12.32	16.05	15.27	16.52	14.27	14.0433	15.353333	------	------	------	------
BAB_RS21945	-	30S ribosomal protein S12	1.165	0.220213055	0.036464944	0.098372684	no	up	mRNA	1681.06	1387.93	1484.84	1925.85	1573.48	1527.86	1517.94	1675.73	COG0048	J:Translation, ribosomal structure and biogenesis	[illegible]	[illegible]
BAB_RS19400	-	EamA family transporter	1.165	0.220493859	0.170026884	0.305705863	no	up	mRNA	14.37	13.83	15.08	17.71	17.5	12.31	14.4267	15.84	COG0697;ENOG4111JSY	S:Function unknown;	[illegible]	[illegible]
BAB_RS22700	-	Bcr/CflA family drug resistance efflux transporter	1.164	0.219637045	0.003757941	0.020499597	no	up	mRNA	133.77	120.12	151.96	151.79	148.62	143.66	135.283	148.02333	ENOG410XNNX	S:Function unknown	[illegible]	[illegible]
BAB_RS28815	putA	trifunctional transcriptional regulator/proline dehydrogenase/L-glutamate gamma-semialdehyde dehydrogenase	1.164	0.21920682	0.005563987	0.02700467	no	up	mRNA	44.19	45.66	48.41	50.96	47.19	53.39	46.0867	50.513333	COG0506	E:Amino acid transport and metabolism	[illegible]	[illegible]
BAB_RS21395	ispDF	bifunctional 2-C-methyl-D-erythritol 4-phosphate cytidylyltransferase/2-C-methyl-D-erythritol 2%2C4-cyclodiphosphate synthase	1.164	0.218829685	0.009852028	0.039224988	no	up	mRNA	35.48	34.81	34.56	39.62	37.91	37.54	34.95	38.356667	COG0245	I:Lipid transport and metabolism	[illegible]	[illegible]
BAB_RS32210	-	hypothetical protein	1.164	0.219196266	0.054670463	0.132771124	no	up	mRNA	13.1	10.56	15.73	14.4	14.08	14.58	13.13	14.353333	COG0034	F:Nucleotide transport and metabolism	------	------
BAB_RS18255	-	metalloprotease TldD	1.163	0.217296338	0.001174836	0.009366623	no	up	mRNA	178.21	163.81	180.88	189.31	198.16	184.87	174.3	190.78	COG0312	S:Function unknown	[illegible]	[illegible]
BAB_RS16155	-	L%2CD-transpeptidase	1.163	0.21781087	0.002504232	0.015684399	no	up	mRNA	396.82	374.38	411.35	435.01	406.07	454.7	394.183	431.92667	COG1376	S:Function unknown	[illegible]	[illegible]
BAB_RS23040	-	-	1.163	0.217712798	0.011564468	0.043920375	no	up	mRNA	115.78	94.06	125.43	122.16	123.01	121.21	111.757	122.12667	------	------	------	------
BAB_RS30995	-	LOG family protein	1.162	0.216398941	0.00397159	0.021303371	no	up	mRNA	109.52	96.16	107.41	112.47	110.17	120.01	104.363	114.21667	COG1611	E:Amino acid transport and metabolism	[illegible]	[illegible]
BAB_RS26710	-	NfeD family protein	1.162	0.216757461	0.069065089	0.157715799	no	up	mRNA	21.8	24.03	31.3	28.13	26.66	30.15	25.71	28.313333	COG1585;COG1585	O:Posttranslational modification, protein turnover, chaperones;U:Intracellular trafficking, secretion, and vesicular transport	[illegible]	[illegible]
BAB_RS27095	-	hypothetical protein	1.162	0.216188054	0.629102103	0.753655876	no	up	mRNA	1.29	1.83	1.82	1.74	1.31	2.69	1.64667	1.9133333	COG3334	S:Function unknown	------	------
BAB_RS23600	-	DUF1134 domain-containing protein	1.161	0.215868865	0.000869346	0.007488496	no	up	mRNA	948.39	873.04	945.89	1008.88	1005.05	1025.32	922.44	1013.0833	COG5400	S:Function unknown	------	------
BAB_RS21525	-	citrate (Si)-synthase	1.161	0.215779135	0.004010684	0.021405859	no	up	mRNA	272.36	228.54	250.31	266.76	273.27	281.54	250.403	273.85667	COG0372	C:Energy production and conversion	[illegible]	[illegible]
BAB_RS29410	-	hypothetical protein	1.161	0.21508447	0.006672273	0.030365458	no	up	mRNA	38.62	37.16	45.8	44.41	45.14	43.15	40.5267	44.233333	------	------	------	------
BAB_RS18470	-	glycoside hydrolase	1.161	0.215545693	0.021731862	0.068388318	no	up	mRNA	20.24	19.58	20.56	22.88	21.5	21.79	20.1267	22.056667	COG0472	M:Cell wall/membrane/envelope biogenesis	[illegible]	[illegible]
BAB_RS32415	-	hypothetical protein	1.161	0.215121367	0.041757448	0.108813203	no	up	mRNA	17.13	14.7	19.54	19.19	19.31	17.58	17.1233	18.693333	------	------	------	------
BAB_RS20395	-	penicillin-binding protein 1A	1.16	0.214243321	0.001001773	0.00829561	no	up	mRNA	337.91	315.04	347.8	362.55	368.01	361.51	333.583	364.02333	COG5009	M:Cell wall/membrane/envelope biogenesis	[illegible]	[illegible]
BAB_RS23205	-	excinuclease ABC subunit UvrB	1.16	0.214684187	0.001496386	0.01061344	no	up	mRNA	136.41	123.88	140.45	143.79	152.09	141.17	133.58	145.68333	COG0556	L:Replication, recombination and repair	[illegible]	[illegible]
BAB_RS25190	-	peptidylprolyl isomerase	1.16	0.213890434	0.005507193	0.026810018	no	up	mRNA	239.48	225.03	219.52	257.79	249.41	241.51	228.01	249.57	COG0760	E:Amino acid transport and metabolism	[illegible]	[illegible]
BAB_RS25785	-	septum formation inhibitor Maf	1.16	0.214338606	0.013221739	0.047678391	no	up	mRNA	135.64	111.98	122.88	130.72	143.37	132.5	123.5	135.53	COG0424	S:Function unknown	[illegible]	[illegible]
BAB_RS23325	-	redox-regulated ATPase YchF	1.16	0.213647576	0.027459753	0.080134592	no	up	mRNA	60.28	53.31	61.46	71.39	57.9	61.98	58.35	63.756667	COG0012	J:Translation, ribosomal structure and biogenesis	[illegible]	[illegible]
BAB_RS33115	-	hypothetical protein	1.16	0.213799168	0.049475526	0.123515824	no	up	mRNA	132.5	115.56	137.5	148.44	131.7	151.64	128.52	143.92667	COG0612	L:Replication, recombination and repair	[illegible]	[illegible]
BAB_RS16190	-	alpha-D-glucose phosphate-specific phosphoglucomutase	1.159	0.212614158	0.001651302	0.011459252	no	up	mRNA	153.98	139.82	153	155.9	165.45	165.53	148.933	162.29333	COG0033	G:Carbohydrate transport and metabolism	[illegible]	[illegible]
BAB_RS31105	-	4-hydroxy-tetrahydrodipicolinate reductase	1.159	0.212826766	0.013595818	0.048483199	no	up	mRNA	75.46	80.16	88.88	93.08	87.1	88.45	81.5	89.543333	COG0289	E:Amino acid transport and metabolism	[illegible]	[illegible]
BAB_RS31170	-	bifunctional phosphopantothenoylcysteine decarboxylase/phosphopantothenate--cysteine ligase CoaBC	1.159	0.213396509	0.01407401	0.049637536	no	up	mRNA	38.25	38.46	45.89	46.01	46.46	41.32	40.8667	44.596667	COG0452	H:Coenzyme transport and metabolism	[illegible]	[illegible]
BAB_RS16960	-	Cu(I)-responsive transcriptional regulator	1.158	0.211442553	0.017650402	0.058767608	no	up	mRNA	36.05	32.09	38.92	42.55	38	35.89	35.6867	38.813333	COG0789	K:Transcription	[illegible]	[illegible]
BAB_RS29895	-	adenosylmethionine--8-amino-7-oxononanoate transaminase	1.158	0.211123847	0.02057866	0.06605318	no	up	mRNA	29.58	31.66	34.6	33.29	37.02	34.58	31.9467	34.963333	COG0161	H:Coenzyme transport and metabolism	[illegible]	[illegible]
BAB_RS27350	-	3'(2')%2C5'-bisphosphate nucleotidase CysQ	1.158	0.211338122	0.041267906	0.107887536	no	up	mRNA	35.88	32.55	40.79	42.73	39.64	37.25	36.4067	39.873333	COG0483	P:Inorganic ion transport and metabolism	[illegible]	[illegible]
BAB_RS25195	-	hypothetical protein	1.158	0.211887359	0.051369376	0.126766364	no	up	mRNA	10.02	8.61	12.6	11.08	11.54	11.34	10.41	11.32	------	------	[illegible]	[illegible]
BAB_RS30075	-	AraC family transcriptional regulator	1.158	0.211996371	0.280329607	0.436385188	no	up	mRNA	7.18	5.55	6.66	8.72	5.6	6.83	6.46333	7.05	ENOG410XPGX	K:Transcription	[illegible]	[illegible]
BAB_RS25025	-	3-isopropylmalate dehydratase large subunit	1.157	0.209979585	0.003045089	0.017886418	no	up	mRNA	131.56	118.94	126.74	135.44	142.46	132.97	125.747	136.95667	COG0065	E:Amino acid transport and metabolism	[illegible]	[illegible]
BAB_RS23230	-	hypothetical protein	1.157	0.209937746	0.02752422	0.080249836	no	up	mRNA	669.08	665.52	655.21	636.15	778.55	809.71	663.27	741.47	------	------	------	------
BAB_RS28755	-	EamA/RhaT family transporter	1.157	0.210344513	0.034412953	0.094021103	no	up	mRNA	19.51	19.64	20.89	22.02	21.26	22.48	20.0133	21.92	COG0697	S:Function unknown	[illegible]	[illegible]
BAB_RS32655	-	hypothetical protein	1.157	0.210940911	0.057742439	0.138659535	no	up	mRNA	39.14	35.55	51.69	49.26	45.3	42.61	42.1267	45.723333	------	------	------	------
BAB_RS28410	-	ABC transporter permease	1.157	0.210047603	0.129430307	0.249166913	no	up	mRNA	15.17	14.96	12.77	16.66	15.32	14.77	14.3	15.583333	COG1177	P:Inorganic ion transport and metabolism	[illegible]	[illegible]
BAB_RS26245	-	translation initiation factor IF-2	1.156	0.208931341	0.002635198	0.016127411	no	up	mRNA	783.91	744.21	828.44	862.85	848.03	850.2	785.52	853.69333	COG0532	J:Translation, ribosomal structure and biogenesis	[illegible]	[illegible]
BAB_RS25185	-	bifunctional glutamate N-acetyltransferase/amino-acid N-acetyltransferase	1.156	0.209101496	0.003202207	0.018504842	no	up	mRNA	108.84	106.52	110.44	114.68	118.03	122.57	108.6	118.42667	COG1364	E:Amino acid transport and metabolism	[illegible]	[illegible]
BAB_RS19945	-	proline--tRNA ligase	1.155	0.207978872	0.004514733	0.023358835	no	up	mRNA	237.1	218.41	226.73	260.85	238.35	243.41	227.413	247.53667	COG0442	J:Translation, ribosomal structure and biogenesis	[illegible]	[illegible]
BAB_RS16495	-	dipeptidase	1.155	0.207960116	0.006205052	0.028768878	no	up	mRNA	175.61	155	165.25	168.67	190.6	179.77	165.287	179.68	COG0624	E:Amino acid transport and metabolism	[illegible]	[illegible]
BAB_RS21575	-	ribosome-recycling factor	1.155	0.20755506	0.006500009	0.029792482	no	up	mRNA	219.65	193.53	202.02	232.66	219.18	221.7	205.067	224.51333	COG0233	J:Translation, ribosomal structure and biogenesis	[illegible]	[illegible]
BAB_RS16045	-	3-phosphoshikimate 1-carboxyvinyltransferase	1.155	0.207683504	0.009172183	0.037493922	no	up	mRNA	79.64	70.27	80.21	89.4	79.66	81.07	76.7067	83.376667	COG0128	E:Amino acid transport and metabolism	[illegible]	[illegible]
BAB_RS28450	-	acyl-CoA dehydrogenase	1.155	0.20850632	0.012192343	0.045183389	no	up	mRNA	76.86	63.5	69.21	73.15	78.39	76.14	69.8567	75.893333	COG1960;COG1960	E:Amino acid transport and metabolism;P:Inorganic ion transport and metabolism	[illegible]	[illegible]
BAB_RS31480	-	GTP-binding protein	1.155	0.207877203	0.044070699	0.113460863	no	up	mRNA	16.04	13.37	15.71	17.13	14.83	17.02	15.04	16.326667	COG0523	S:Function unknown	------	------
BAB_RS27120	-	DUF2195 domain-containing protein	1.155	0.207821134	0.046434818	0.117847606	no	up	mRNA	31.85	26.46	31.63	33.5	34.65	29.51	29.98	32.553333	COG4893	S:Function unknown	------	------
BAB_RS31790	-	ABC transporter ATP-binding protein	1.155	0.207585394	0.269347666	0.424430629	no	up	mRNA	5.93	3.83	5.66	6.14	4.88	5.67	5.14	5.5633333	COG3839	S:Function unknown	[illegible]	[illegible]
BAB_RS24160	-	flagellar motor protein MotA	1.154	0.206900665	0.004635898	0.023718378	no	up	mRNA	142.44	124.52	147.47	143.69	152.01	155.1	138.143	150.26667	ENOG410ZQA4	S:Function unknown	[illegible]	[illegible]
BAB_RS19410	-	DUF992 domain-containing protein	1.153	0.205608322	0.002187105	0.01416768	no	up	mRNA	1344.79	1307.89	1378.5	1413.64	1541.61	1472.46	1343.73	1475.9033	ENOG4111WVD	S:Function unknown	------	------
BAB_RS28480	-	acetyl-CoA acetyltransferase	1.153	0.205885435	0.00707877	0.031632944	no	up	mRNA	259.43	246.51	270.12	254.63	291.61	297.88	258.687	281.37333	COG0183	K:Transcription	[illegible]	[illegible]

BAB_RS20515	-	Fe-S cluster assembly scaffold SufA	1.153	0.205887319	0.009021665	0.037114738	no	up	mRNA	409.73	343.3	361.56	384.09	410.49	422.94	371.53	405.84	COG0316	G:Carbohydrate transport and metabolism	GO:0097428;GO:0097428;GO:0051536;GO:0005198;GO:0051536;GO:0005198	BP:protein maturation by iron-sulfur cluster transfer;BP:protein maturation by iron-sulfur cluster transfer;MF:iron-sulfur cluster binding;MF:structural molecule activity;MF:iron-sulfur cluster binding;MF:structural molecule activity;
BAB_RS29065	-	branched-chain amino acid aminotransferase	1.153	0.205199601	0.009222605	0.037604353	no	up	mRNA	203.76	187.16	196.02	194.91	219.48	225.24	195.647	213.21	COG0115	C:Energy production and conversion	GO:0008152;GO:0008152;GO:0052656;GO:0052654;GO:0052655;GO:0008483	BP:metabolic process;BP:metabolic process;MF:L-isoleucine transaminase activity;MF:L-leucine transaminase activity;MF:L-valine transaminase activity;MF:transaminase activity;
BAB_RS29950	-	hypothetical protein	1.153	0.205752615	0.382366225	0.547235047	no	up	mRNA	122.18	90.92	118.19	145.11	115.8	133.66	110.43	131.52333	------	------	------	------
BAB_RS16390	-	dihydroxy-acid dehydratase	1.152	0.204638837	0.001426042	0.010296346	no	up	mRNA	226.29	215.2	232.17	245.16	241.7	243.47	224.553	243.44333	COG0129	E:Amino acid transport and metabolism	GO:0009097;GO:0009099;GO:0009097;GO:0009099;GO:0009097;GO:0009099;GO:0051539;GO:0004160;GO:0046872;GO:0051539;GO:0004160;GO:0046872;GO:0016757;GO:0004160;GO:0046872;GO:0051539	BP:isoleucine biosynthetic process;BP:valine biosynthetic process;BP:isoleucine biosynthetic process;BP:valine biosynthetic process;BP:isoleucine biosynthetic process;BP:valine biosynthetic process;MF:4 iron, 4 sulfur cluster binding;MF:dihydroxy-acid dehydratase activity;MF:metal ion binding;MF:4 iron, 4 sulfur cluster binding;MF:dihydroxy-acid dehydratase activity;MF:metal ion binding;MF:transferase activity, transferring glycosyl groups;MF:dihydroxy-acid dehydratase activity;MF:metal ion binding;MF:4 iron, 4 sulfur cluster binding;
BAB_RS22675	-	protease modulator HflC	1.152	0.203732909	0.002721744	0.016407062	no	up	mRNA	482.07	463.25	487.88	506.98	548.94	504.22	477.733	520.04667	COG0330	O:Posttranslational modification, protein turnover, chaperones	GO:0052547;GO:0052547;GO:0052547;GO:0052547;GO:0016021;GO:0016021;GO:0016021;GO:0016021;GO:0016787;GO:0008233;GO:0008233	BP:regulation of peptidase activity;BP:regulation of peptidase activity;BP:regulation of peptidase activity;BP:regulation of peptidase activity;CC:integral component of membrane;CC:integral component of membrane;CC:integral component of membrane;CC:integral component of membrane;MF:hydrolase activity;MF:peptidase activity;MF:peptidase activity;
BAB_RS30535	-	glutaredoxin-like protein NrdH	1.152	0.204212848	0.004622049	0.023685237	no	up	mRNA	1197.02	1155.92	1261.08	1215.22	1356.75	1369.22	1204.67	1313.73	COG0695	F:Nucleotide transport and metabolism	GO:0045454;GO:0045454;GO:0005623;GO:0005623;GO:0015035;GO:0009055;GO:0009055;GO:0015035	BP:cell redox homeostasis;BP:cell redox homeostasis;CC:cell;CC:cell;MF:protein disulfide oxidoreductase activity;MF:electron carrier activity;MF:electron carrier activity;MF:protein disulfide oxidoreductase activity;
BAB_RS25200	-	protein translocase subunit SecA	1.152	0.203770934	0.007363745	0.032233941	no	up	mRNA	149.84	137.66	142.89	165.81	151.55	149.09	143.463	155.48333	COG0653	U:Intracellular trafficking, secretion, and vesicular transport	GO:0017038;GO:0006605;GO:0065002;GO:0065002;GO:0017038;GO:0006605;GO:0005737;GO:0005886;GO:0005737;GO:0005886;GO:0046872;GO:0005524;GO:0005524;GO:0046872	BP:protein import;BP:protein targeting;BP:intracellular protein transmembrane transport;BP:intracellular protein transmembrane transport;BP:protein import;BP:protein targeting;CC:cytoplasm;CC:plasma membrane;CC:cytoplasm;CC:plasma membrane;MF:metal ion binding;MF:ATP binding;MF:ATP binding;MF:metal ion binding;
BAB_RS23950	-	photosystem reaction center subunit H	1.152	0.203979614	0.013955297	0.049272933	no	up	mRNA	255.29	247.95	276.64	250.28	291.74	304.19	259.96	282.07	ENOG41125DS	S:Function unknown	------	------
BAB_RS24100	-	protein TolR	1.152	0.203917306	0.02089841	0.066547662	no	up	mRNA	396.74	299.21	352.95	377.78	384.44	385.2	349.633	382.47333	COG0848	S:Function unknown	GO:0015031;GO:0015031;GO:0016021;GO:0005886;GO:0016021;GO:0005886;GO:0022857;GO:0022857	BP:protein transport;BP:protein transport;CC:integral component of membrane;CC:plasma membrane;CC:integral component of membrane;CC:plasma membrane;MF:transmembrane transporter activity;MF:transmembrane transporter activity;
BAB_RS29020	-	transcriptional regulator	1.152	0.203636394	0.03679139	0.099003966	no	up	mRNA	141.87	136.92	185.29	169.72	172.74	161.11	154.693	167.85667	COG5631	K:Transcription	GO:0006351;GO:0003700;GO:0003677;GO:0003700	BP:transcription, DNA-templated;MF:transcription factor activity, sequence-specific DNA binding;MF:DNA binding;MF:transcription factor activity, sequence-specific DNA binding;
BAB_RS27395	-	IclR family transcriptional regulator	1.152	0.20366629	0.046607931	0.11810038	no	up	mRNA	140.53	115.62	133.69	151.33	149.94	121.34	129.947	140.87	COG1414	G:Carbohydrate transport and metabolism	GO:0006355;GO:0006351;GO:0006355;GO:0006351;GO:0003677;GO:0003677	BP:regulation of transcription, DNA-templated;BP:transcription, DNA-templated;BP:regulation of transcription, DNA-templated;BP:transcription, DNA-templated;MF:DNA binding;MF:DNA binding;
BAB_RS16590	-	tryptophan--tRNA ligase	1.151	0.202689029	0.00914495	0.037430222	no	up	mRNA	104	91.97	98.14	111.38	106	101.61	98.0367	106.33	COG0180	J:Translation, ribosomal structure and biogenesis	GO:0006436;GO:0006436;GO:0005737;GO:0005737;GO:0004830;GO:0005524;GO:0005524;GO:0004830	BP:tryptophanyl-tRNA aminoacylation;BP:tryptophanyl-tRNA aminoacylation;CC:cytoplasm;CC:cytoplasm;MF:tryptophan-tRNA ligase activity;MF:ATP binding;MF:ATP binding;MF:tryptophan-tRNA ligase activity;
BAB_RS29235	-	amino acid ABC transporter	1.151	0.203007645	0.048667335	0.121972034	no	up	mRNA	40.81	36.73	44.31	40.46	43.86	48.55	40.6167	44.29	COG0834;ENOG410YVP9	S:Function unknown;E:Amino acid transport and metabolism	GO:0016020;GO:0016020;GO:0004970;GO:0004970	CC:membrane;CC:membrane;MF:ionotropic glutamate receptor activity;MF:ionotropic glutamate receptor activity;
BAB_RS20015	-	BolA family transcriptional regulator	1.15	0.20185385	0.005425553	0.02645266	no	up	mRNA	488.38	480.97	501.81	534.25	556.75	535.42	490.387	542.14	COG0271	T:Signal transduction mechanisms	GO:0005524	MF:ATP binding;
BAB_RS23505	-	DUF2333 domain-containing protein	1.149	0.200246554	0.005837498	0.027745388	no	up	mRNA	194.78	166.8	185.75	199.56	204.61	187.78	182.443	197.31667	COG5345	S:Function unknown	GO:0016021	CC:integral component of membrane;
BAB_RS20785	-	methionine--tRNA ligase	1.149	0.2009659	0.00849064	0.035707366	no	up	mRNA	110.68	96.35	105.25	119.15	109.1	109.53	104.093	112.59333	COG0143	J:Translation, ribosomal structure and biogenesis	GO:0006431;GO:0006431;GO:0006431;GO:0005737;GO:0005737;GO:0005737;GO:0004825;GO:0005524;GO:0004825;GO:0005524;GO:0016757;GO:0005524;GO:0004825;GO:0016757	BP:methionyl-tRNA aminoacylation;BP:methionyl-tRNA aminoacylation;BP:methionyl-tRNA aminoacylation;CC:cytoplasm;CC:cytoplasm;CC:cytoplasm;MF:methionine-tRNA ligase activity;MF:ATP binding;MF:methionine-tRNA ligase activity;MF:ATP binding;MF:transferase activity, transferring glycosyl groups;MF:ATP binding;MF:methionine-tRNA ligase activity;MF:transferase activity, transferring glycosyl groups;
BAB_RS29305	-	DNA polymerase IV	1.149	0.199891334	0.016977941	0.057060801	no	up	mRNA	41.93	40.23	39.76	41.89	44.06	46.08	40.64	44.01	COG0389	S:Function unknown	GO:0006281;GO:0006261;GO:0006281;GO:0006260;GO:0006261;GO:0005737;GO:0005737;GO:0003684;GO:0003887;GO:0000287;GO:0000287;GO:0003887;GO:0046872;GO:0003684	BP:DNA repair;BP:DNA-dependent DNA replication;BP:DNA repair;BP:DNA replication;BP:DNA-dependent DNA replication;CC:cytoplasm;CC:cytoplasm;MF:damaged DNA binding;MF:DNA-directed DNA polymerase activity;MF:magnesium ion binding;MF:magnesium ion binding;MF:DNA-directed DNA polymerase activity;MF:metal ion binding;MF:damaged DNA binding;
BAB_RS23315	-	50S ribosomal protein L25	1.149	0.200654619	0.025333643	0.076143121	no	up	mRNA	541.64	447.95	500.96	595.46	504.95	513.22	496.85	537.87667	COG1825	J:Translation, ribosomal structure and biogenesis	GO:0006412;GO:0006412;GO:0005840;GO:0005840;GO:0008097;GO:0003735;GO:0003735;GO:0008097	BP:translation;BP:translation;CC:ribosome;CC:ribosome;MF:5S rRNA binding;MF:structural constituent of ribosome;MF:structural constituent of ribosome;MF:5S rRNA binding;
BAB_RS28665	-	HlyD family secretion protein	1.149	0.200877656	0.057725364	0.13872221	no	up	mRNA	23.44	27.67	27.94	28.36	29.21	28.76	26.35	28.776667	COG1566	S:Function unknown	GO:0055085;GO:0055085;GO:0016021;GO:0016021;GO:0005215;GO:0005215	BP:transmembrane transport;BP:transmembrane transport;CC:integral component of membrane;CC:integral component of membrane;MF:transporter activity;MF:transporter activity;
BAB_RS16175	-	hypothetical protein	1.149	0.200173924	0.21922292	0.366855855	no	up	mRNA	90.98	105.02	98.7	84.59	133.66	109.01	98.2333	109.08667	ENOG410ZA2F	S:Function unknown	GO:0016021	CC:integral component of membrane;
BAB_RS21790	-	PDZ domain-containing protein	1.148	0.199747373	0.007198169	0.031900301	no	up	mRNA	56.23	50.13	55.41	58.4	60.54	55.79	53.9233	58.243333	COG0265	L:Replication, recombination and repair	GO:0042597;GO:0004252	CC:periplasmic space;MF:serine-type endopeptidase activity;
BAB_RS23850	-	DedA family protein	1.148	0.198498369	0.010393642	0.040675726	no	up	mRNA	227.7	205.4	221.66	247.01	244.85	217.96	218.253	236.60667	COG1238	S:Function unknown	GO:0016021	CC:integral component of membrane;
BAB_RS31500	-	transcriptional repressor	1.147	0.197316323	0.039451744	0.104759052	no	up	mRNA	160.3	188.58	182.46	196.55	190.81	197.5	177.113	194.95333	COG0735	P:Inorganic ion transport and metabolism	GO:0003700;GO:0003677	MF:transcription factor activity, sequence-specific DNA binding;MF:DNA binding;
BAB_RS16785	-	stress responsive protein	1.147	0.198336707	0.08042608	0.177356894	no	up	mRNA	38.12	27.85	37.53	35.13	38.54	37.92	34.5	37.196667	ENOG411234B	S:Function unknown	------	------
BAB_RS17030	-	IclR family transcriptional regulator	1.147	0.198332368	0.088659591	0.18890137	no	up	mRNA	92.95	81.41	76.67	103.02	80.59	88.08	83.6767	90.563333	COG1414	K:Transcription	GO:0006355;GO:0006351;GO:0006355;GO:0006351;GO:0003677;GO:0003677	BP:regulation of transcription, DNA-templated;BP:transcription, DNA-templated;BP:regulation of transcription, DNA-templated;BP:transcription, DNA-templated;MF:DNA binding;MF:DNA binding;
BAB_RS18400	-	DSBA oxidoreductase	1.146	0.197218392	0.004422235	0.022991331	no	up	mRNA	808.78	757.81	830.98	813.2	921.36	871.83	799.19	868.79667	COG1651	S:Function unknown	GO:0045454;GO:0005623	BP:cell redox homeostasis;CC:cell;
BAB_RS27320	-	insulinase family protein	1.146	0.196267347	0.008621969	0.035930461	no	up	mRNA	213.47	198.56	203.56	235.74	211.87	217.05	205.197	221.55333	COG0612	L:Replication, recombination and repair	GO:0046872;GO:0004222	MF:metal ion binding;MF:metalloendopeptidase activity;
BAB_RS28130	-	aminotransferase	1.146	0.197070764	0.010598012	0.041324531	no	up	mRNA	60.18	57.65	67.36	65.86	67.37	67.43	61.73	66.886667	ENOG410Z4EP	G:Carbohydrate transport and metabolism	GO:0008483;GO:0016491	MF:transaminase activity;MF:oxidoreductase activity;
BAB_RS30105	-	peptide-methionine (R)-S-oxide reductase	1.145	0.194944131	0.004257437	0.022315083	no	up	mRNA	148.97	137.19	152.93	155.47	166.73	151.04	146.363	157.74667	COG0229	O:Posttranslational modification, protein turnover, chaperones	GO:0030091;GO:0006979;GO:0006979;GO:0030091;GO:0033743;GO:0033743;GO:0008168	BP:protein repair;BP:response to oxidative stress;BP:response to oxidative stress;BP:protein repair;MF:peptide-methionine (R)-S-oxide reductase activity;MF:peptide-methionin;MF:methyltransferase activity;
BAB_RS23495	-	MFS transporter	1.145	0.195966954	0.00932088	0.037813114	no	up	mRNA	525.63	465.95	498.73	563.89	524.76	517.4	496.77	535.35	ENOG410XPZP	G:Carbohydrate transport and metabolism	GO:0055085;GO:0055085;GO:0016021;GO:0016021	BP:transmembrane transport;BP:transmembrane transport;CC:integral component of membrane;CC:integral component of membrane;
BAB_RS23795	-	hypothetical protein	1.145	0.195008748	0.323562693	0.486024746	no	up	mRNA	405.82	519.89	355.58	336.76	579.17	487.36	427.097	467.76333	COG5591	S:Function unknown	------	------
BAB_RS29335	-	MFS transporter	1.144	0.194522511	0.003202518	0.018473413	no	up	mRNA	191.58	188.78	194.54	200.5	212.52	207.8	191.633	206.94	ENOG410XR1I	G:Carbohydrate transport and metabolism	GO:0055085;GO:0055085;GO:0016021;GO:0016021	BP:transmembrane transport;BP:transmembrane transport;CC:integral component of membrane;CC:integral component of membrane;
BAB_RS19065	-	YitT family protein	1.144	0.193875553	0.009425817	0.038046672	no	up	mRNA	69.19	65.25	74.4	79.18	74.44	71.38	69.6133	75	COG1284	S:Function unknown	GO:0016021	CC:integral component of membrane;
BAB_RS19180	-	DUF2062 domain-containing protein	1.144	0.194692831	0.029862209	0.084386349	no	up	mRNA	277.22	268.55	258.28	315.84	294.93	263.51	268.017	291.42667	COG3216	S:Function unknown	GO:0016021	CC:integral component of membrane;
BAB_RS33130	-	hypothetical protein	1.144	0.194032327	0.125206985	0.24292877	no	up	mRNA	84.64	71.23	81.52	96.92	72.84	89.67	79.13	86.476667	------	------	------	------
BAB_RS20890	-	NADP-dependent malic enzyme	1.143	0.193050777	0.004015642	0.021361354	no	up	mRNA	135.69	117.36	130.88	135.29	136.7	140.62	127.977	137.53667	COG0280	J:Translation, ribosomal structure and biogenesis	GO:0006108;GO:0006108;GO:0004473;GO:0051287;GO:0004471;GO:0008948;GO:0016746;GO:0046872;GO:0004471;GO:0046872;GO:0051287;GO:0016746	BP:malate metabolic process;BP:malate metabolic process;MF:malate dehydrogenas;MF:NAD binding;MF:malate dehydrogenas;MF:oxaloacetate decarboxylase activity;MF:transferase activity, transferring acyl groups;MF:metal ion binding;MF:malate dehydrogenas;MF:metal ion binding;MF:NAD binding;MF:transferase activity, transferring acyl groups;
BAB_RS16715	-	nucleotide exchange factor GrpE	1.143	0.193139549	0.007642615	0.033005001	no	up	mRNA	271.62	241.31	260.59	290.88	279.82	266.45	257.84	279.05	COG0576	O:Posttranslational modification, protein turnover, chaperones	GO:0006457;GO:0006457;GO:0006457;GO:0005737;GO:0005737;GO:0005737;GO:0051087;GO:0000774;GO:0042803;GO:0000774;GO:0051087;GO:0042803;GO:0000774;GO:0051087;GO:0042803	BP:protein folding;BP:protein folding;BP:protein folding;CC:cytoplasm;CC:cytoplasm;CC:cytoplasm;MF:chaperone binding;MF:adenyl-nucleotide exchange factor activity;MF:protein homodimerization activity;MF:adenyl-nucleotide exchange factor activity;MF:chaperone binding;MF:protein homodimerization activity;MF:adenyl-nucleotide exchange factor activity;MF:chaperone binding;MF:protein homodimerization activity;
BAB_RS17870	-	ribosome biogenesis GTPase Der	1.143	0.192284029	0.01365664	0.04853848	no	up	mRNA	105.65	109.11	109.44	122.71	116.99	109.86	108.067	116.52	COG1160	S:Function unknown	GO:0042254;GO:0005525	BP:ribosome biogenesis;MF:GTP binding;
BAB_RS24355	-	thiamine-binding periplasmic protein	1.143	0.192461651	0.016860004	0.056842805	no	up	mRNA	38.26	36.68	41.23	39.04	41.59	44.54	38.7233	41.723333	COG4143	H:Coenzyme transport and metabolism	GO:0015888;GO:0030288;GO:0030975	BP:thiamine transport;CC:outer membrane-bounded periplasmic space;MF:thiamine binding;
BAB_RS23965	-	TonB system transport protein ExbD	1.143	0.193149573	0.128131394	0.246962309	no	up	mRNA	32.7	28.51	32.42	31	35.72	35.23	31.21	33.983333	COG0848	U:Intracellular trafficking, secretion, and vesicular transport	GO:0015031;GO:0015031;GO:0016021;GO:0005886;GO:0016021;GO:0005886;GO:0022857;GO:0022857	BP:protein transport;BP:protein transport;CC:integral component of membrane;CC:plasma membrane;CC:integral component of membrane;CC:plasma membrane;MF:transmembrane transporter activity;MF:transmembrane transporter activity;
BAB_RS26690	-	lytic murein transglycosylase	1.142	0.191580482	0.006390613	0.029416961	no	up	mRNA	142.76	136.56	148.91	149.67	146.56	164.59	142.743	153.60667	COG2951	U:Intracellular trafficking, secretion, and vesicular transport	GO:0008152;GO:0016798	BP:metabolic process;MF:hydrolase activity, acting on glycosyl bonds;
BAB_RS25535	-	DUF3108 domain-containing protein	1.142	0.191278935	0.012107038	0.045075219	no	up	mRNA	84.14	77.71	79.96	82.73	88.27	89.64	80.6033	86.88	ENOG4111PCD;ENOG4111QKT	S:Function unknown;	------	------
BAB_RS30065	-	phosphoglycerate dehydrogenase	1.142	0.19201525	0.018282201	0.060495069	no	up	mRNA	57.67	52.32	57.05	64.85	55.96	58.76	55.68	59.856667	COG0111	J:Translation, ribosomal structure and biogenesis	GO:0006564;GO:0006564;GO:0051287;GO:0004617;GO:0051287;GO:0004617	BP:L-serine biosynthetic process;BP:L-serine biosynthetic process;MF:NAD binding;MF:phosphoglycerate dehydrogenase activity;MF:NAD binding;MF:phosphoglycerate dehydrogenase activity;
BAB_RS31185	-	uracil phosphoribosyltransferase	1.142	0.191750308	0.019757651	0.064057852	no	up	mRNA	133.96	118.31	121.17	126.03	135.83	142.12	124.48	134.66	COG0035	F:Nucleotide transport and metabolism	GO:0044206;GO:0006223;GO:0044206;GO:0006223;GO:0006223;GO:0044206;GO:0005525;GO:0000287;GO:0004845;GO:0000287;GO:0004845;GO:0005525;GO:0016787;GO:0000287;GO:0004845;GO:0005525	BP:UMP salvage;BP:uracil salvage;BP:UMP salvage;BP:uracil salvage;BP:uracil salvage;BP:UMP salvage;MF:GTP binding;MF:magnesium ion binding;MF:uracil phosphoribosyltransferase activity;MF:magnesium ion binding;MF:uracil phosphoribosyltransferase activity;MF:GTP binding;MF:hydrolase activity;MF:magnesium ion binding;MF:uracil phosphoribosyltransferase activity;MF:GTP binding;
BAB_RS18280	-	cytochrome c oxidase assembly protein	1.142	0.191924964	0.149753643	0.278609412	no	up	mRNA	24.63	20.46	23.72	26.77	26.26	20.92	22.9367	24.65	COG3175	O:Posttranslational modification, protein turnover, chaperones	GO:0008535;GO:0016021;GO:0005886;GO:0016021;GO:0005507;GO:0005507	BP:respiratory chain complex IV assembly;CC:integral component of membrane;CC:plasma membrane;CC:integral component of membrane;MF:copper ion binding;MF:copper ion binding;
BAB_RS30820	-	nitric-oxide reductase large subunit	1.142	0.191354064	0.416297348	0.58053966	no	up	mRNA	149.68	200.64	118.03	120.81	215.39	172.62	156.117	169.60667	COG3256	P:Inorganic ion transport and metabolism	GO:0009060;GO:0009060;GO:0009060;GO:0016021;GO:0016021;GO:0016021;GO:0004129;GO:0016966;GO:0020037;GO:0004129;GO:0020037;GO:0004129;GO:0020037	BP:aerobic respiration;BP:aerobic respiration;BP:aerobic respiration;CC:integral component of membrane;CC:integral component of membrane;CC:integral component of membrane;MF:cytochrome-c oxidase activity;MF:nitric oxide reductase activity;MF:heme binding;MF:cytochrome-c oxidase activity;MF:heme binding;MF:cytochrome-c oxidase activity;MF:heme binding;
BAB_RS23195	-	acyl-CoA thioesterase	1.141	0.189743654	0.004861134	0.024404409	no	up	mRNA	233.56	230.29	237.79	243.6	264	247.12	233.88	251.57333	COG1607	I:Lipid transport and metabolism	GO:0016787;GO:0047617	MF:hydrolase activity;MF:acyl-CoA hydrolase activity;
BAB_RS24625	-	LemA family protein	1.141	0.189899836	0.004921927	0.024632636	no	up	mRNA	687.57	635.79	708.19	685.55	766.17	745.76	677.183	732.49333	COG1704	S:Function unknown	------	------
BAB_RS28905	-	superoxide dismutase [Cu-Zn]	1.141	0.189901721	0.008010729	0.034317961	no	up	mRNA	6515.2	5847.27	5898.48	6355.01	6754.26	6712.75	6086.98	6607.34	COG2032	P:Inorganic ion transport and metabolism	GO:0042597;GO:0042597;GO:0004784;GO:0046872;GO:0046872;GO:0004784;GO:0046872;GO:0004784	CC:periplasmic space;CC:periplasmic space;MF:superoxide dismutase activity;MF:metal ion binding;MF:metal ion binding;MF:superoxide dismutase activity;MF:metal ion binding;MF:superoxide dismutase activity;
BAB_RS24690	-	S41 family peptidase	1.141	0.190445505	0.01185252	0.04448849	no	up	mRNA	1078.63	911.64	969.32	1065.29	1057.39	1060.34	986.53	1061.0067	COG0793	M:Cell wall/membrane/envelope biogenesis	GO:0008236	MF:serine-type peptidase activity;
BAB_RS21320	-	single-stranded DNA-binding protein	1.141	0.189883128	0.048185673	0.121142853	no	up	mRNA	1713.89	1366.95	1655.41	1539.68	1691.28	1842.22	1578.75	1691.06	COG0629	L:Replication, recombination and repair	GO:0006310;GO:0006281;GO:0006260;GO:0006310;GO:0006260;GO:0006281;GO:0003697;GO:0003697	BP:DNA recombination;BP:DNA repair;BP:DNA replication;BP:DNA recombination;BP:DNA replication;BP:DNA repair;MF:single-stranded DNA binding;MF:single-stranded DNA binding;
BAB_RS25045	-	hypothetical protein	1.141	0.189934997	0.436850885	0.602145815	no	up	mRNA	9.12	14.57	10.8	12.59	13.14	12.78	11.4967	12.836667	ENOG4111HP8	S:Function unknown	------	------
BAB_RS18960	-	heme lyase CcmF/NrfE family subunit	1.14	0.189636702	0.004784627	0.024171394	no	up	mRNA	115.56	106.52	115.77	114.53	126.61	121.45	112.617	120.86333	COG1138	O:Posttranslational modification, protein turnover, chaperones	GO:0017004;GO:0017004;GO:0016021;GO:0016021;GO:0020037;GO:0015232;GO:0015232;GO:0020037	BP:cytochrome complex assembly;BP:cytochrome complex assembly;CC:integral component of membrane;CC:integral component of membrane;MF:heme binding;MF:heme transporter activity;MF:heme transporter activity;MF:heme binding;
BAB_RS21155	-	D-alanyl-D-alanine carboxypeptidase	1.14	0.189443607	0.005798327	0.027640988	no	up	mRNA	146.77	149.7	156.12	166.71	157.83	162.55	150.863	162.36333	COG1686	J:Translation, ribosomal structure and biogenesis	GO:0042834;GO:0009002	MF:peptidoglycan binding;MF:serine-type D-Ala-D-Ala carboxypeptidase activity;
BAB_RS22855	-	phospho-N-acetylmuramoyl-pentapeptide-transferase	1.14	0.188442427	0.006258284	0.028973869	no	up	mRNA	128.45	121.94	125.96	134.51	132.12	138.25	125.45	134.96	COG0472	M:Cell wall/membrane/envelope biogenesis	GO:0071555;GO:0008360;GO:0007049;GO:0009252;GO:0051301;GO:0007049;GO:0051301;GO:0071555;GO:0009252;GO:0008360;GO:0005886;GO:0016021;GO:0016021;GO:0005886;GO:0008963;GO:0051992;GO:0016491;GO:0008963;GO:0051992	BP:cell wall organization;BP:regulation of cell shape;BP:cell cycle;BP:peptidoglycan biosynthetic process;BP:cell division;BP:cell cycle;BP:cell division;BP:cell wall organization;BP:peptidoglycan biosynthetic process;BP:regulation of cell shape;CC:plasma membrane;CC:integral component of membrane;CC:integral component of membrane;CC:plasma membrane;MF:phospho-N-acetylmuramoyl-pentapeptide-transferase activity;MF:UDP-N-acetylmuramoyl-L-alanyl-D-glutamyl-meso-2,6-diaminopimelyl-D-alanyl-D-alanin;MF:oxidoreductase activity;MF:phospho-N-acetylmuramoyl-pentapeptide-transferase activity;MF:UDP-N-acetylmuramoyl-L-alanyl-D-glutamyl-meso-2,6-diaminopimelyl-D-alanyl-D-alanin;
BAB_RS17510	-	aspartate aminotransferase family protein	1.14	0.188607741	0.007098266	0.031676011	no	up	mRNA	92.6	83.36	88.76	95.39	95.77	93.01	88.24	94.723333	COG4992	E:Amino acid transport and metabolism	GO:0006526;GO:0006526;GO:0005737;GO:0005737;GO:0003992;GO:0030170;GO:0030170;GO:0003992	BP:arginine biosynthetic process;BP:arginine biosynthetic process;CC:cytoplasm;CC:cytoplasm;MF:N2-acetyl-L-ornithin;MF:pyridoxal phosphate binding;MF:pyridoxal phosphate binding;MF:N2-acetyl-L-ornithin;
BAB_RS25370	-	TlpA family protein disulfide reductase	1.14	0.189061334	0.008892347	0.036723794	no	up	mRNA	309.07	281.24	295.95	338.8	311.76	303.21	295.42	317.92333	COG0526	S:Function unknown	GO:0045454;GO:0045454;GO:0005623;GO:0016021;GO:0016021;GO:0005623;GO:0016491;GO:0016491	BP:cell redox homeostasis;BP:cell redox homeostasis;CC:cell;CC:integral component of membrane;CC:integral component of membrane;CC:cell;MF:oxidoreductase activity;MF:oxidoreductase activity;
BAB_RS23165	-	glycosyltransferase family 1 protein	1.14	0.188657317	0.028546609	0.081893085	no	up	mRNA	41.73	41.27	44.63	49.17	41.73	46.37	42.5433	45.756667	COG0438	P:Inorganic ion transport and metabolism	GO:0016740;GO:0016757	MF:transferase activity;MF:transferase activity, transferring glycosyl groups;
BAB_RS31160	-	hypothetical protein	1.14	0.188658775	0.086150131	0.18527468	no	up	mRNA	566.55	606.16	588.35	665.75	683.08	661.77	587.02	670.2	COG3247	S:Function unknown	GO:0016021	CC:integral component of membrane;
BAB_RS20070	-	adenylosuccinate lyase	1.139	0.187804439	0.011014218	0.042483411	no	up	mRNA	59.81	52.01	58.19	58.04	62.58	61.51	56.67	60.71	COG0015	F:Nucleotide transport and metabolism	GO:0006189;GO:0044208;GO:0016491;GO:0004018;GO:0070626	BP:'de novo' IMP biosynthetic process;BP:'de novo' AMP biosynthetic process;MF:oxidoreductase activity;MF:N6;MF:;
BAB_RS27540	-	hypothetical protein	1.139	0.18816136	0.013666816	0.04852097	no	up	mRNA	65.16	61.17	66.57	73.53	64.04	69.3	64.3	68.956667	COG0400	S:Function unknown	------	------
BAB_RS18745	-	haloacid dehalogenase type II	1.139	0.187896513	0.014671045	0.051125887	no	up	mRNA	60.71	58.45	62.24	63.59	63.15	68.61	60.4667	65.116667	COG1011	S:Function unknown	GO:0008152;GO:0008152;GO:0008152;GO:0008152;GO:0018784;GO:0019120;GO:0019120;GO:0018784;GO:0019120;GO:0018784;GO:0050019	BP:metabolic process;BP:metabolic process;BP:metabolic process;BP:metabolic process;MF:;MF:hydrolase activity, acting on acid halide bonds, in C-halide compounds;MF:hydrolase activity, acting on acid halide bonds, in C-halide compounds;MF:;MF:hydrolase activity, acting on acid halide bonds, in C-halide compounds;MF:;MF:L-arabinitol 4-dehydrogenase activity;
BAB_RS27330	-	peptidase M16	1.138	0.186454275	0.01576991	0.053960298	no	up	mRNA	78.77	75.7	83.93	86.87	80.13	89.5	79.4667	85.5	COG0612	O:Posttranslational modification, protein turnover, chaperones	GO:0046872;GO:0008233	MF:metal ion binding;MF:peptidase activity;
BAB_RS32360	-	hypothetical protein	1.138	0.186499411	0.214385699	0.360828314	no	up	mRNA	2634.75	3072.32	2251.85	2748.33	3581.46	2920.23	2652.97	3083.34	------	------	GO:0003852	MF:2-isopropylmalate synthase activity;
BAB_RS24015	-	membrane protein	1.137	0.185701413	0.005108233	0.025289298	no	up	mRNA	101.32	92.02	102.87	105.09	102.57	109.45	98.7367	105.70333	COG1633	S:Function unknown	GO:0016021;GO:0016021;GO:0046872;GO:0016491;GO:0046872;GO:0016491	CC:integral component of membrane;CC:integral component of membrane;MF:metal ion binding;MF:oxidoreductase activity;MF:metal ion binding;MF:oxidoreductase activity;
BAB_RS24305	-	5-(carboxyamino)imidazole ribonucleotide synthase	1.137	0.184694134	0.006733395	0.030513963	no	up	mRNA	71.98	69.61	75.86	77.23	79.41	76.41	72.4833	77.683333	COG0026	F:Nucleotide transport and metabolism	GO:0006189;GO:0006189;GO:0006189;GO:0016787;GO:0046872;GO:0004638;GO:0034028;GO:0005524;GO:0046872;GO:0034028;GO:0005524;GO:0004638;GO:0016787;GO:0034028;GO:0004638;GO:0046872;GO:0005524	BP:'de novo' IMP biosynthetic process;BP:'de novo' IMP biosynthetic process;BP:'de novo' IMP biosynthetic process;MF:hydrolase activity;MF:metal ion binding;MF:phosphoribosylaminoimidazole carboxylase activity;MF:5;MF:ATP binding;MF:metal ion binding;MF:5;MF:ATP binding;MF:phosphoribosylaminoimidazole carboxylase activity;MF:hydrolase activity;MF:5;MF:phosphoribosylaminoimidazole carboxylase activity;MF:metal ion binding;MF:ATP binding;
BAB_RS20035	-	phosphoribosylformylglycinamidine synthase subunit PurQ	1.137	0.185124003	0.012358644	0.045589348	no	up	mRNA	206.67	185.78	197.93	223.72	206.86	204.78	196.793	211.78667	COG0047	F:Nucleotide transport and metabolism	GO:0006189;GO:0006541;GO:0006541;GO:0006189;GO:0005737;GO:0005737;GO:0005524;GO:0004359;GO:0004642;GO:0004359;GO:0004642;GO:0005524;GO:0016491	BP:'de novo' IMP biosynthetic process;BP:glutamine metabolic process;BP:glutamine metabolic process;BP:'de novo' IMP biosynthetic process;CC:cytoplasm;CC:cytoplasm;MF:ATP binding;MF:glutaminase activity;MF:phosphoribosylformylglycinamidine synthase activity;MF:glutaminase activity;MF:phosphoribosylformylglycinamidine synthase activity;MF:ATP binding;MF:oxidoreductase activity;
BAB_RS32945	-	phosphoethanolamine transferase	1.137	0.185127411	0.024149469	0.073686841	no	up	mRNA	80.62	74.28	81.94	74.85	88.03	90.43	78.9467	84.436667	COG2194	S:Function unknown	GO:0008152;GO:0008152;GO:0008152;GO:0008152;GO:0016021;GO:0016021;GO:0016021;GO:0008484;GO:0016740;GO:0008484;GO:0016740;GO:0008484;GO:0008484	BP:metabolic process;BP:metabolic process;BP:metabolic process;BP:metabolic process;CC:integral component of membrane;CC:integral component of membrane;CC:integral component of membrane;MF:sulfuric ester hydrolase activity;MF:transferase activity;MF:sulfuric ester hydrolase activity;MF:transferase activity;MF:sulfuric ester hydrolase activity;MF:sulfuric ester hydrolase activity;
BAB_RS24725	-	glutamate 5-kinase	1.137	0.185129696	0.028626577	0.081903109	no	up	mRNA	49.28	49.06	55.91	50.73	58.62	56.24	51.4167	55.196667	COG0263	E:Amino acid transport and metabolism	GO:0055129;GO:0055129;GO:0005737;GO:0005737;GO:0005524;GO:0004349;GO:0003723;GO:0004349;GO:0003723;GO:0005524;GO:0016491	BP:L-proline biosynthetic process;BP:L-proline biosynthetic process;CC:cytoplasm;CC:cytoplasm;MF:ATP binding;MF:glutamate 5-kinase activity;MF:RNA binding;MF:glutamate 5-kinase activity;MF:RNA binding;MF:ATP binding;MF:oxidoreductase activity;
BAB_RS23385	-	coproporphyrinogen III oxidase	1.136	0.183731933	0.014760699	0.051216118	no	up	mRNA	121.25	107.07	109.04	116.55	123.74	120.54	112.453	120.27667	COG0408	H:Coenzyme transport and metabolism	GO:0006782;GO:0006782;GO:0005737;GO:0005737;GO:0004109;GO:0046872;GO:0042803;GO:0004109;GO:0046872;GO:0042803	BP:protoporphyrinogen IX biosynthetic process;BP:protoporphyrinogen IX biosynthetic process;CC:cytoplasm;CC:cytoplasm;MF:coproporphyrinogen oxidase activity;MF:metal ion binding;MF:protein homodimerization activity;MF:coproporphyrinogen oxidase activity;MF:metal ion binding;MF:protein homodimerization activity;
BAB_RS20455	-	multidrug transporter	1.136	0.184046648	0.037040412	0.099507393	no	up	mRNA	60.69	57.89	56.39	60.47	61.8	66.03	58.3233	62.766667	COG2076	O:Posttranslational modification, protein turnover, chaperones	GO:0016021;GO:0005886	CC:integral component of membrane;CC:plasma membrane;
BAB_RS32570	-	hypothetical protein	1.136	0.18398713	0.101789708	0.208578017	no	up	mRNA	13.19	11.88	17.1	14.48	15.81	14.8	14.0567	15.03	COG1058	H:Coenzyme transport and metabolism	------	------
BAB_RS16690	-	DUF2244 domain-containing protein	1.136	0.183968674	0.175391939	0.310829729	no	up	mRNA	55.54	47.23	52.37	44.37	62.95	59.53	51.7133	55.616667	COG5488	S:Function unknown	GO:0016021	CC:integral component of membrane;
BAB_RS27645	-	hypothetical protein	1.136	0.184461147	0.215618747	0.361955608	no	up	mRNA	8.44	7.83	8.78	9.5	9.46	7.86	8.35	8.94	ENOG410Y07C	S:Function unknown	GO:0016021	CC:integral component of membrane;
BAB_RS26980	-	hypothetical protein	1.136	0.184381557	0.30844488	0.470129695	no	up	mRNA	5.88	5.13	5.61	5.74	6.39	5.62	5.54	5.9166667	ENOG4111WB0	S:Function unknown	GO:0016021	CC:integral component of membrane;
BAB_RS25145	-	tRNA (N(6)-L-threonylcarbamoyladenosine(37)-C(2))-methylthiotransferase MtaB	1.135	0.182321284	0.010227934	0.040124971	no	up	mRNA	70.69	67.21	73.02	77.59	76.13	71.88	70.3067	75.2	COG0621	J:Translation, ribosomal structure and biogenesis	GO:0006400;GO:0006400;GO:0016740;GO:0051539;GO:0051539;GO:0016740	BP:tRNA modification;BP:tRNA modification;MF:transferase activity;MF:4 iron, 4 sulfur cluster binding;MF:4 iron, 4 sulfur cluster binding;MF:transferase activity;
BAB_RS22680	-	FtsH protease activity modulator HflK	1.135	0.182816185	0.01704988	0.057242701	no	up	mRNA	469.48	413.26	422.75	488.62	466.85	442.34	435.163	465.93667	COG0330	O:Posttranslational modification, protein turnover, chaperones	GO:0016021	CC:integral component of membrane;
BAB_RS17160	-	flagellar protein FlgJ	1.135	0.182262563	0.042846058	0.110840888	no	up	mRNA	16.73	13.86	18.3	17.69	17.4	16.96	16.2967	17.35	COG1705;COG1705;ENOG410XPFT	N:Cell motility;U:Intracellular trafficking, secretion, and vesicular transport;S:Function unknown	GO:0004040	MF:amidase activity;
BAB_RS32555	-	hypothetical protein	1.135	0.182813031	0.077982021	0.173155655	no	up	mRNA	29.2	23.68	35.72	30.42	32.53	31.14	29.5333	31.363333	------	------	------	------
BAB_RS33505	-	hypothetical protein	1.135	0.183142432	0.083743065	0.181924589	no	up	mRNA	13.52	12.62	16.39	15.19	15.6	14.75	14.1767	15.18	------	------	------	------
BAB_RS30790	-	metal-sulfur cluster assembly factor	1.135	0.183256843	0.378868629	0.544411854	no	up	mRNA	45	66.02	48.74	42.38	64.36	68.83	53.2533	58.523333	COG2151	Q:Secondary metabolites biosynthesis, transport and catabolism	------	------
BAB_RS23065	-	RNA polymerase sigma factor RpoD	1.134	0.180901703	0.007829257	0.033720379	no	up	mRNA	647.33	580.56	634.63	679.02	659.45	647.56	620.84	662.01	COG0568	K:Transcription	GO:0001123;GO:0001123;GO:0005737;GO:0005737;GO:0016987;GO:0003677;GO:0003700;GO:0003700;GO:0003677;GO:0016987	BP:transcription initiation from bacterial-type RNA polymerase promoter;BP:transcription initiation from bacterial-type RNA polymerase promoter;CC:cytoplasm;CC:cytoplasm;MF:sigma factor activity;MF:DNA binding;MF:transcription factor activity, sequence-specific DNA binding;MF:transcription factor activity, sequence-specific DNA binding;MF:DNA binding;MF:sigma factor activity;
BAB_RS25630	-	amidohydrolase	1.134	0.181493555	0.008680445	0.036080558	no	up	mRNA	215.48	203.12	216.49	209.45	235.7	234.02	211.697	226.39	COG1473	E:Amino acid transport and metabolism	GO:0008152;GO:0016787	BP:metabolic process;MF:hydrolase activity;
BAB_RS27580	-	gamma-glutamyl-gamma-aminobutyrate hydrolase	1.134	0.181172569	0.043660214	0.112584484	no	up	mRNA	18.71	16.78	20.37	20.54	19.1	20.03	18.62	19.89	COG2071	S:Function unknown	GO:0006541;GO:0006541;GO:0016787;GO:0016740;GO:0004049;GO:0016787;GO:0016740	BP:glutamine metabolic process;BP:glutamine metabolic process;MF:hydrolase activity;MF:transferase activity;MF:anthranilate synthase activity;MF:hydrolase activity;MF:transferase activity;
BAB_RS24220	-	DUF4164 domain-containing protein	1.134	0.18156937	0.091592647	0.193865069	no	up	mRNA	424.43	394.71	454.97	420.97	480.11	517.01	424.703	472.69667	ENOG411295F	S:Function unknown	------	------
BAB_RS23445	-	glutathione S-transferase family protein	1.134	0.181879455	0.235344284	0.386981159	no	up	mRNA	6.44	6.64	6.32	6.7	7.52	6.52	6.46667	6.9133333	COG0435	O:Posttranslational modification, protein turnover, chaperones	GO:0004364	MF:glutathione transferase activity;
BAB_RS29640	-	mannan endo-1%2C4-beta-mannosidase	1.134	0.181672357	0.277143893	0.43289418	no	up	mRNA	6.3	4.79	5.94	5.64	6.43	6	5.67667	6.0233333	COG4124	G:Carbohydrate transport and metabolism	GO:0006080;GO:0006080;GO:0016985;GO:0016985	BP:substituted mannan metabolic process;BP:substituted mannan metabolic process;MF:mannan endo-1,4-beta-mannosidase activity;MF:mannan endo-1,4-beta-mannosidase activity;
BAB_RS19845	-	-	1.134	0.181321079	0.339901877	0.504669468	no	up	mRNA	11.85	9.95	14.65	12.36	12.19	15.2	12.15	13.25	ENOG410Y1PH	S:Function unknown	------	------
BAB_RS18045	-	J domain-containing protein	1.133	0.180687558	0.010716699	0.041635735	no	up	mRNA	372.29	340.97	373.84	413.31	386.27	363.21	362.367	387.59667	COG2214	O:Posttranslational modification, protein turnover, chaperones	GO:0006457;GO:0006457;GO:0051082;GO:0051082	BP:protein folding;BP:protein folding;MF:unfolded protein binding;MF:unfolded protein binding;
BAB_RS31245	-	ABC transporter permease	1.133	0.18050996	0.166439888	0.300602226	no	up	mRNA	48.5	40.56	50.86	57.54	51.5	40.02	46.64	49.686667	COG0601	S:Function unknown	GO:0055085;GO:0055085;GO:0016021;GO:0005886;GO:0016021;GO:0005886	BP:transmembrane transport;BP:transmembrane transport;CC:integral component of membrane;CC:plasma membrane;CC:integral component of membrane;CC:plasma membrane;
BAB_RS24630	-	30S ribosomal protein S16	1.132	0.178352664	0.012732503	0.046540992	no	up	mRNA	221.61	188.56	205.69	222.75	219.66	216.87	205.287	219.76	COG0228	J:Translation, ribosomal structure and biogenesis	GO:0006412;GO:0005840;GO:0003735	BP:translation;CC:ribosome;MF:structural constituent of ribosome;
BAB_RS23575	-	tRNA(5-methylaminomethyl-2-thiouridine)-methyltransferase	1.132	0.178306646	0.021755658	0.068396212	no	up	mRNA	163.61	141.66	146.06	168.22	158.09	154.65	150.443	160.32	COG0482	J:Translation, ribosomal structure and biogenesis	------	------
BAB_RS21130	-	nucleoside triphosphate pyrophosphohydrolase	1.132	0.179246083	0.023188525	0.071570346	no	up	mRNA	37.44	31.28	35.56	35.63	37.24	38.03	34.76	36.966667	COG1694	S:Function unknown	GO:0047429;GO:0016787	MF:nucleoside-triphosphate diphosphatase activity;MF:hydrolase activity;
BAB_RS24010	-	class II glutamine amidotransferase	1.132	0.178909647	0.052427966	0.12898243	no	up	mRNA	33.88	37.86	43.83	39.34	41.83	42.42	38.5233	41.196667	COG0121	S:Function unknown	GO:0006541;GO:0016740	BP:glutamine metabolic process;MF:transferase activity;
BAB_RS32920	-	hypothetical protein	1.132	0.179493727	0.059663928	0.141790088	no	up	mRNA	20.36	18.3	25.95	23	23.41	22.16	21.5367	22.856667	------	------	------	------
BAB_RS20860	-	glutathione-disulfide reductase	1.131	0.177248191	0.026631779	0.078287196	no	up	mRNA	105.46	87.76	91.99	98.98	103.11	101.24	95.07	101.11	COG1249	C:Energy production and conversion	GO:0006749;GO:0045454;GO:0006749;GO:0045454;GO:0045454;GO:0006749;GO:0045454;GO:0006749;GO:0005623;GO:0005623;GO:0005623;GO:0005623;GO:0050661;GO:0050660;GO:0004362;GO:0050003;GO:0009055;GO:0050661;GO:0050660;GO:0004362;GO:0016874;GO:0009055;GO:0050003;GO:0009055;GO:0050660;GO:0004362;GO:0050661;GO:0009055;GO:0050660;GO:0004362;GO:0050661	BP:glutathione metabolic process;BP:cell redox homeostasis;BP:glutathione metabolic process;BP:cell redox homeostasis;BP:cell redox homeostasis;BP:glutathione metabolic process;BP:cell redox homeostasis;BP:glutathione metabolic process;CC:cell;CC:cell;CC:cell;CC:cell;MF:NADP binding;MF:flavin adenine dinucleotide binding;MF:glutathione-disulfide reductase activity;MF:deoxycytidylate C-methyltransferase activity;MF:electron carrier activity;MF:NADP binding;MF:flavin adenine dinucleotide binding;MF:glutathione-disulfide reductase activity;MF:ligase activity;MF:electron carrier activity;MF:deoxycytidylate C-methyltransferase activity;MF:electron carrier activity;MF:flavin adenine dinucleotide binding;MF:glutathione-disulfide reductase activity;MF:NADP binding;MF:electron carrier activity;MF:flavin adenine dinucleotide binding;MF:glutathione-disulfide reductase
BAB_RS17595	-	large conductance mechanosensitive channel protein MscL	1.131	0.177814142	0.028447188	0.08182705	no	up	mRNA	270.33	230.18	254.47	243.99	282.51	282.48	251.66	269.66	COG1970	M:Cell wall/membrane/envelope biogenesis	GO:0034220;GO:0034220;GO:0016021;GO:0005887;GO:0005886;GO:0016021;GO:0005886;GO:0008381;GO:0008381	BP:ion transmembrane transport;BP:ion transmembrane transport;CC:integral component of membrane;CC:integral component of plasma membrane;CC:plasma membrane;CC:integral component of membrane;CC:plasma membrane;MF:mechanically-gated ion channel activity;MF:mechanically-gated ion channel activity;
BAB_RS26140	-	phosphate ABC transporter ATP-binding protein	1.131	0.177387909	0.029423018	0.083586344	no	up	mRNA	97.3	91.74	92.57	91.99	102.71	106.28	93.87	100.32667	COG1117	P:Inorganic ion transport and metabolism	GO:0008152;GO:0005886;GO:0005886;GO:0005524;GO:0015415;GO:0005315;GO:0005315;GO:0016798;GO:0015415;GO:0005524	BP:metabolic process;CC:plasma membrane;CC:plasma membrane;MF:ATP binding;MF:ATPase-coupled phosphate ion transmembrane transporter activity;MF:inorganic phosphate transmembrane transporter activity;MF:inorganic phosphate transmembrane transporter activity;MF:hydrolase activity, acting on glycosyl bonds;MF:ATPase-coupled phosphate ion transmembrane transporter activity;MF:ATP binding;
BAB_RS26420	-	4'-phosphopantetheinyl transferase	1.131	0.178027909	0.464763088	0.628222045	no	up	mRNA	3.94	3.01	4.29	2.82	4.67	4.45	3.74667	3.98	COG2977	Q:Secondary metabolites biosynthesis, transport and catabolism	GO:0009239;GO:0009239;GO:0009366;GO:0009366;GO:0008897;GO:0000287;GO:0008897;GO:0000287	BP:enterobactin biosynthetic process;BP:enterobactin biosynthetic process;CC:enterobactin synthetase complex;CC:enterobactin synthetase complex;MF:holo-[acyl-carrier-protein] synthase activity;MF:magnesium ion binding;MF:holo-[acyl-carrier-protein] synthase activity;MF:magnesium ion binding;
BAB_RS23175	-	DUF1176 domain-containing protein	1.13	0.175893565	0.034215941	0.093642093	no	up	mRNA	57	49.54	51.44	52.79	56.54	58.66	52.66	55.996667	ENOG410ZMG7;ENOG410ZY6K	S:Function unknown;	------	------
BAB_RS26135	-	phosphate ABC transporter%2C permease protein PstA	1.13	0.176045705	0.059605527	0.141756151	no	up	mRNA	67.13	63.54	65.34	61.04	77.29	70.33	65.3367	69.553333	COG0581	P:Inorganic ion transport and metabolism	GO:0035435;GO:0035435;GO:0005887;GO:0005887;GO:0005315;GO:0005315	BP:phosphate ion transmembrane transport;BP:phosphate ion transmembrane transport;CC:integral component of plasma membrane;CC:integral component of plasma membrane;MF:inorganic phosphate transmembrane transporter activity;MF:inorganic phosphate transmembrane transporter activity;
BAB_RS29455	-	cytochrome c biogenesis protein CcdA	1.129	0.175553295	0.014270082	0.050054339	no	up	mRNA	189.99	188.76	201.58	194.75	221.56	202.64	193.443	206.31667	COG0785	O:Posttranslational modification, protein turnover, chaperones	GO:0055114;GO:0017004;GO:0017004;GO:0055114;GO:0016021;GO:0016020;GO:0016021	BP:oxidation-reduction process;BP:cytochrome complex assembly;BP:cytochrome complex assembly;BP:oxidation-reduction process;CC:integral component of membrane;CC:membrane;CC:integral component of membrane;
BAB_RS16105	-	3-deoxy-manno-octulosonate cytidylyltransferase	1.129	0.1755606	0.017620246	0.058789046	no	up	mRNA	84.08	76.74	86.39	88.78	90.91	84	82.4033	87.896667	COG1212	M:Cell wall/membrane/envelope biogenesis	GO:0033468;GO:0009103;GO:0033468;GO:0009103;GO:0005737;GO:0005737;GO:0008690;GO:0008690	BP:CMP-keto-3-deoxy-D-manno-octulosonic acid biosynthetic process;BP:lipopolysaccharide biosynthetic process;BP:CMP-keto-3-deoxy-D-manno-octulosonic acid biosynthetic process;BP:lipopolysaccharide biosynthetic process;CC:cytoplasm;CC:cytoplasm;MF:3-deoxy-manno-octulosonate cytidylyltransferase activity;MF:3-deoxy-manno-octulosonate cytidylyltransferase activity;
BAB_RS25735	-	ParA family protein	1.129	0.17553986	0.019008389	0.06238402	no	up	mRNA	161.6	138.61	151.05	150.92	163.82	166.34	150.42	160.36	COG1192	D:Cell cycle control, cell division, chromosome partitioning	------	------
BAB_RS22740	-	FAD-binding protein	1.129	0.17463521	0.022593403	0.070409896	no	up	mRNA	60	59.33	68.07	62.3	70.77	66.23	62.4667	66.433333	COG0277	S:Function unknown	GO:0050660;GO:0016614;GO:0052854;GO:0052852;GO:0052853;GO:0004458;GO:0050660;GO:0016614	MF:flavin adenine dinucleotide binding;MF:oxidoreductase activity, acting on CH-OH group of donors;MF:medium-chain;MF:very-long-chain;MF:long-chain;MF:D-lactate dehydrogenas;MF:flavin adenine dinucleotide binding;MF:oxidoreductase activity, acting on CH-OH group of donors;
BAB_RS25875	-	phosphoribosyl-ATP pyrophosphatase	1.129	0.175491706	0.255897713	0.409665845	no	up	mRNA	45.53	42.83	44.71	51.44	47.21	48.55	44.3567	49.066667	COG0140	E:Amino acid transport and metabolism	GO:0000105;GO:0000105;GO:0005737;GO:0005737;GO:0005524;GO:0004636;GO:0005524;GO:0004636	BP:histidine biosynthetic process;BP:histidine biosynthetic process;CC:cytoplasm;CC:cytoplasm;MF:ATP binding;MF:phosphoribosyl-ATP diphosphatase activity;MF:ATP binding;MF:phosphoribosyl-ATP diphosphatase activity;
BAB_RS27415	-	2%2C4-dienoyl-CoA reductase	1.129	0.175332813	0.371130193	0.536893881	no	up	mRNA	3.37	3.77	3.23	3.87	4.24	2.87	3.45667	3.66	COG1902	C:Energy production and conversion	GO:0016491;GO:0010181	MF:oxidoreductase activity;MF:FMN binding;
BAB_RS27270	-	molecular chaperone GroEL	1.128	0.173960917	0.099603161	0.205803831	no	up	mRNA	2905.84	2434.68	2575.96	2410.1	3058.72	2947.36	2638.83	2805.3933	COG0459	O:Posttranslational modification, protein turnover, chaperones	GO:0042026;GO:0042026;GO:0042026;GO:0005737;GO:0005737;GO:0005737;GO:0005524;GO:0051082;GO:0051082;GO:0005524;GO:0005524;GO:0051082	BP:protein refolding;BP:protein refolding;BP:protein refolding;CC:cytoplasm;CC:cytoplasm;CC:cytoplasm;MF:ATP binding;MF:unfolded protein binding;MF:unfolded protein binding;MF:ATP binding;MF:ATP binding;MF:unfolded protein binding;
BAB_RS17130	-	hypothetical protein	1.128	0.174404197	0.222905425	0.371663275	no	up	mRNA	6.32	7.24	6.93	8.07	7.44	6.35	6.83	7.2866667	ENOG410XR3E	S:Function unknown	------	------
BAB_RS24190	-	biotin transporter BioY	1.127	0.171886771	0.007174224	0.031838096	no	up	mRNA	747.08	754.6	781.08	788.41	843.39	814.21	760.92	815.33667	COG1268	K:Transcription	GO:0016021;GO:0005886;GO:0016021;GO:0005886;GO:0015225;GO:0015225	CC:integral component of membrane;CC:plasma membrane;CC:integral component of membrane;CC:plasma membrane;MF:biotin transporter activity;MF:biotin transporter activity;
BAB_RS18370	-	HAD family phosphatase	1.127	0.172480816	0.050567701	0.12565663	no	up	mRNA	38.17	33.55	42.35	42.37	39.38	39.51	38.0233	40.42	COG0637	S:Function unknown	GO:0008152;GO:0016787	BP:metabolic process;MF:hydrolase activity;
BAB_RS17165	-	hypothetical protein	1.127	0.172145609	0.084232647	0.182371628	no	up	mRNA	21.02	19.43	20.86	20.79	22.38	22.01	20.4367	21.726667	ENOG4110S5I	S:Function unknown	------	------
BAB_RS32495	-	hypothetical protein	1.127	0.172767826	0.282078399	0.437834732	no	up	mRNA	201.19	187.88	226.46	256.22	241.38	193.6	205.177	230.4	------	------	------	------
BAB_RS19020	-	electron transfer flavoprotein-ubiquinone oxidoreductase	1.126	0.170803166	0.004697458	0.023957038	no	up	mRNA	162.7	149.32	162.57	163.49	169.55	169.73	158.197	167.59	COG0644	C:Energy production and conversion	GO:0004174	MF:electron-transferring-flavoprotein dehydrogenase activity;
BAB_RS22870	-	penicillin-binding protein 2	1.126	0.170676926	0.010025795	0.039719948	no	up	mRNA	106.42	97.25	104.79	110.44	104.17	112.23	102.82	108.94667	COG0768	M:Cell wall/membrane/envelope biogenesis	GO:0051301;GO:0051301;GO:0016021;GO:0016021;GO:0008658;GO:0016740;GO:0008658	BP:cell division;BP:cell division;CC:integral component of membrane;CC:integral component of membrane;MF:penicillin binding;MF:transferase activity;MF:penicillin binding;
BAB_RS27230	-	histidine--tRNA ligase	1.126	0.171586539	0.01214509	0.045164552	no	up	mRNA	73.07	68.31	76.96	77.43	73.79	80.39	72.78	77.203333	COG0124	J:Translation, ribosomal structure and biogenesis	GO:0006427;GO:0006427;GO:0006427;GO:0005737;GO:0005737;GO:0005737;GO:0005524;GO:0004821;GO:0016829;GO:0004821;GO:0005524;GO:0004821;GO:0005524	BP:histidyl-tRNA aminoacylation;BP:histidyl-tRNA aminoacylation;BP:histidyl-tRNA aminoacylation;CC:cytoplasm;CC:cytoplasm;CC:cytoplasm;MF:ATP binding;MF:histidine-tRNA ligase activity;MF:lyase activity;MF:histidine-tRNA ligase activity;MF:ATP binding;MF:histidine-tRNA ligase activity;MF:ATP binding;
BAB_RS26780	-	molybdate ABC transporter permease subunit	1.126	0.171363491	0.042178748	0.109467138	no	up	mRNA	55.26	49.78	54.63	56.23	58.84	55.01	53.2233	56.693333	COG4149	P:Inorganic ion transport and metabolism	GO:0016021;GO:0005886;GO:0016021;GO:0005886;GO:0015098;GO:0015098	CC:integral component of membrane;CC:plasma membrane;CC:integral component of membrane;CC:plasma membrane;MF:molybdate ion transmembrane transporter activity;MF:molybdate ion transmembrane transporter activity;
BAB_RS21230	-	CoA transferase	1.126	0.171319305	0.05974475	0.141772438	no	up	mRNA	45.1	46.72	53.6	47.47	56.4	50.76	48.4733	51.543333	COG1804	C:Energy production and conversion	GO:0008152;GO:0033608	BP:metabolic process;MF:formyl-CoA transferase activity;
BAB_RS29255	-	hypothetical protein	1.126	0.170944544	0.076350118	0.171188367	no	up	mRNA	111.39	91.3	89.12	107.11	102.68	99.45	97.27	103.08	ENOG410Y8MV	S:Function unknown	GO:0016021	CC:integral component of membrane;
BAB_RS32755	-	hypothetical protein	1.126	0.1714107	0.083153544	0.181749888	no	up	mRNA	39.4	34.38	49.02	44.89	44.7	40	40.9333	43.196667	------	------	------	------
BAB_RS24595	-	heparinase II/III family protein	1.125	0.169412952	0.016660267	0.056346778	no	up	mRNA	77.56	71.19	82.43	79.06	78.17	87.37	77.06	81.533333	COG5360	S:Function unknown	GO:0016829	MF:lyase activity;
BAB_RS26330	-	leucyl aminopeptidase	1.125	0.169411314	0.01931211	0.06305875	no	up	mRNA	34.87	34.04	36	36.56	36.94	37.74	34.97	37.08	COG0260	K:Transcription	GO:0005737;GO:0005737;GO:0004177;GO:0030145;GO:0008235;GO:0004177;GO:0008235;GO:0030145	CC:cytoplasm;CC:cytoplasm;MF:aminopeptidase activity;MF:manganese ion binding;MF:metalloexopeptidase activity;MF:aminopeptidase activity;MF:metalloexopeptidase activity;MF:manganese ion binding;
BAB_RS31795	-	metallophosphoesterase	1.125	0.170380924	0.036368603	0.098277815	no	up	mRNA	29.89	27.8	32.23	31.41	32.39	31.67	29.9733	31.823333	COG1409	S:Function unknown	GO:0016787	MF:hydrolase activity;
BAB_RS26685	-	type IV secretion system protein virB1	1.125	0.169575337	0.061822968	0.144884899	no	up	mRNA	187.87	160.87	179.58	163.7	205.17	192.43	176.107	187.1	ENOG41124SS;ENOG4111WYD	U:Intracellular trafficking, secretion, and vesicular transport;	GO:0009405	BP:pathogenesis;
BAB_RS21260	-	TetR/AcrR family transcriptional regulator	1.124	0.169027721	0.012773695	0.046532746	no	up	mRNA	82.97	77.25	84.75	84.95	90.34	84.26	81.6567	86.516667	COG1309	C:Energy production and conversion	GO:0006355;GO:0006351;GO:0006355;GO:0006351;GO:0003677;GO:0003677	BP:regulation of transcription, DNA-templated;BP:transcription, DNA-templated;BP:regulation of transcription, DNA-templated;BP:transcription, DNA-templated;MF:DNA binding;MF:DNA binding;
BAB_RS24845	-	DUF4167 domain-containing protein	1.124	0.168300667	0.040282603	0.106175556	no	up	mRNA	2016.24	1715.93	1859.1	2153.76	1943.35	1841.72	1863.76	1979.61	ENOG4111WB9	S:Function unknown	------	------
BAB_RS28950	-	6%2C7-dimethyl-8-ribityllumazine synthase 2	1.124	0.168272371	0.040862489	0.107264033	no	up	mRNA	1855.5	1692.31	1753.64	1735.72	2043.85	1837.74	1767.15	1872.4367	COG0054	C:Energy production and conversion	GO:0009405;GO:0009231;GO:0009231;GO:0005737;GO:0009349;GO:0009349;GO:0000906;GO:0000906	BP:pathogenesis;BP:riboflavin biosynthetic process;BP:riboflavin biosynthetic process;CC:cytoplasm;CC:riboflavin synthase complex;CC:riboflavin synthase complex;MF:6,7-dimethyl-8-ribityllumazine synthase activity;MF:6,7-dimethyl-8-ribityllumazine synthase activity;
BAB_RS30170	-	hypothetical protein	1.124	0.168724647	0.054891437	0.13320709	no	up	mRNA	262.3	238.99	209.71	256.14	267.19	243.48	237	255.60333	------	------	------	------
BAB_RS26830	-	chain-length determining protein	1.124	0.168232035	0.068953593	0.157685335	no	up	mRNA	85.35	78.99	87.64	86.35	78.7	101.48	83.9933	88.843333	COG3206	K:Transcription	GO:0009103;GO:0009103;GO:0016021;GO:0016021	BP:lipopolysaccharide biosynthetic process;BP:lipopolysaccharide biosynthetic process;CC:integral component of membrane;CC:integral component of membrane;
BAB_RS21245	-	LLM class flavin-dependent oxidoreductase	1.124	0.169048959	0.070410228	0.160445435	no	up	mRNA	19.27	18.54	22.94	22	22.71	19.58	20.25	21.43	COG2141	C:Energy production and conversion	GO:0047646;GO:0016705;GO:0047646;GO:0004497;GO:0016705	MF:alkanal monooxygenas;MF:oxidoreductase activity, acting on paired donors, with incorporation or reduction of molecular oxygen;MF:alkanal monooxygenas;MF:monooxygenase activity;MF:oxidoreductase activity, acting on paired donors, with incorporation or reduction of molecular oxygen;
BAB_RS30715	-	DUF2384 domain-containing protein	1.124	0.168594012	0.108141615	0.218390326	no	up	mRNA	61.15	70.86	64.08	68.56	71.83	70.54	65.3633	70.31	ENOG4111VRI	S:Function unknown	------	------
BAB_RS25730	-	chromosome partitioning protein ParB	1.123	0.16710182	0.005586353	0.027072328	no	up	mRNA	276.02	250.11	269.68	277.4	285.38	279.71	265.27	280.83	COG1475	K:Transcription	GO:0003677	MF:DNA binding;
BAB_RS25810	-	membrane protein FxsA	1.123	0.167319368	0.009059338	0.037174524	no	up	mRNA	298.43	280.21	296.63	319.7	305	306.62	291.757	310.44	COG3030	S:Function unknown	GO:0016021	CC:integral component of membrane;
BAB_RS21235	-	acyl-CoA dehydrogenase	1.123	0.167891063	0.02414947	0.073616934	no	up	mRNA	465.85	393.79	418.36	432.8	446.95	473.79	426	451.18	COG1960	C:Energy production and conversion	GO:0003995;GO:0050660;GO:0003995;GO:0050660;GO:0004361	MF:acyl-CoA dehydrogenase activity;MF:flavin adenine dinucleotide binding;MF:acyl-CoA dehydrogenase activity;MF:flavin adenine dinucleotide binding;MF:glutaryl-CoA dehydrogenase activity;
BAB_RS30330	-	GMP synthase (glutamine-hydrolyzing)	1.123	0.167565476	0.032431601	0.090062865	no	up	mRNA	139.27	122.22	132.16	150.99	131.19	133.97	131.217	138.71667	COG0518	F:Nucleotide transport and metabolism	GO:0006541;GO:0006177;GO:0006177;GO:0006541;GO:0006541;GO:0006177;GO:0003922;GO:0016462;GO:0016740;GO:0005524;GO:0016491;GO:0016462;GO:0003922;GO:0005524;GO:0005524;GO:0003922;GO:0016462	BP:glutamine metabolic process;BP:GMP biosynthetic process;BP:GMP biosynthetic process;BP:glutamine metabolic process;BP:glutamine metabolic process;BP:GMP biosynthetic process;MF:GMP synthas;MF:pyrophosphatase activity;MF:transferase activity;MF:ATP binding;MF:oxidoreductase activity;MF:pyrophosphatase activity;MF:GMP synthas;MF:ATP binding;MF:ATP binding;MF:GMP synthas;MF:pyrophosphatase activity;
BAB_RS25080	-	GumN family protein	1.123	0.167316383	0.038283832	0.102249336	no	up	mRNA	65.46	59.67	61.17	61.38	69.99	65.75	62.1	65.706667	COG3735	S:Function unknown	------	------
BAB_RS25885	-	aminoacyl-tRNA hydrolase	1.123	0.166973614	0.078026166	0.173134027	no	up	mRNA	77.57	73.24	73.93	85.56	71.94	82.18	74.9133	79.893333	COG1186	E:Amino acid transport and metabolism	GO:0016787;GO:0003747	MF:hydrolase activity;MF:translation release factor activity;
BAB_RS27150	-	cytochrome b	1.123	0.167173765	0.103795939	0.212147806	no	up	mRNA	114.8	113.9	109.7	103.63	136.46	118.82	112.8	119.63667	COG3038	C:Energy production and conversion	GO:0022904;GO:0022904;GO:0016021;GO:0016021;GO:0009055;GO:0009055	BP:respiratory electron transport chain;BP:respiratory electron transport chain;CC:integral component of membrane;CC:integral component of membrane;MF:electron carrier activity;MF:electron carrier activity;
BAB_RS27455	-	FAD:protein FMN transferase	1.123	0.167970634	0.669850565	0.788362588	no	up	mRNA	0.56	1.19	1.72	1.53	1.07	1.35	1.15667	1.3166667	COG1477	H:Coenzyme transport and metabolism	GO:0017013;GO:0005886;GO:0016740;GO:0046872	BP:protein flavinylation;CC:plasma membrane;MF:transferase activity;MF:metal ion binding;
BAB_RS22015	-	RNA methyltransferase	1.122	0.166622456	0.010886905	0.042093411	no	up	mRNA	102.97	94.41	105.88	108.23	109.77	102.46	101.087	106.82	COG0566	J:Translation, ribosomal structure and biogenesis	GO:0006396;GO:0006396;GO:0008173;GO:0003723;GO:0003723;GO:0008173	BP:RNA processing;BP:RNA processing;MF:RNA methyltransferase activity;MF:RNA binding;MF:RNA binding;MF:RNA methyltransferase activity;
BAB_RS26720	-	ferrochelatase	1.121	0.164217803	0.01338688	0.048004516	no	up	mRNA	96.67	93.99	97.12	98.69	102.09	103.44	95.9267	101.40667	COG0276	H:Coenzyme transport and metabolism	GO:0006783;GO:0006783;GO:0005737;GO:0005737;GO:0046872;GO:0004325;GO:0016787;GO:0004325;GO:0046872	BP:heme biosynthetic process;BP:heme biosynthetic process;CC:cytoplasm;CC:cytoplasm;MF:metal ion binding;MF:ferrochelatase activity;MF:hydrolase activity;MF:ferrochelatase activity;MF:metal ion binding;
BAB_RS28155	-	ABC transporter permease	1.121	0.165426303	0.01343234	0.048113832	no	up	mRNA	184.26	175.37	181.06	181.74	198.62	192.24	180.23	190.86667	COG1172	S:Function unknown	GO:0016021;GO:0005886;GO:0022857	CC:integral component of membrane;CC:plasma membrane;MF:transmembrane transporter activity;
BAB_RS24520	-	ATP synthase subunit gamma	1.121	0.164443673	0.027680201	0.080195208	no	up	mRNA	356.02	319.22	373.25	401.29	346.66	360.38	349.497	369.44333	COG0224	C:Energy production and conversion	GO:0015986;GO:0042777;GO:0045261;GO:0005886;GO:0046933;GO:0016798;GO:0005524	BP:ATP synthesis coupled proton transport;BP:plasma membrane ATP synthesis coupled proton transport;CC:proton-transporting ATP synthase complex, catalytic core;CC:plasma membrane;MF:proton-transporting ATP synthase activity, rotational mechanism;MF:hydrolase activity, acting on glycosyl bonds;MF:ATP binding;
BAB_RS27495	-	IclR family transcriptional regulator	1.121	0.16536658	0.281859199	0.437705948	no	up	mRNA	62.09	41.63	47.6	63.11	51.08	44.47	50.44	52.886667	COG1414	S:Function unknown	GO:0006355;GO:0006351;GO:0006355;GO:0006351;GO:0003677;GO:0003677	BP:regulation of transcription, DNA-templated;BP:transcription, DNA-templated;BP:regulation of transcription, DNA-templated;BP:transcription, DNA-templated;MF:DNA binding;MF:DNA binding;
BAB_RS21405	-	short-chain dehydrogenase	1.12	0.163452677	0.011233654	0.042917634	no	up	mRNA	49.75	49.58	57.57	53.63	56.4	55.11	52.3	55.046667	COG4221;COG1028	S:Function unknown;	GO:0047936	MF:glucose 1-dehydrogenase [NA;
BAB_RS24270	-	NAD(P)H quinone oxidoreductase	1.12	0.16325678	0.012561772	0.046179603	no	up	mRNA	101.49	94.96	101.69	102.52	104.24	108.13	99.38	104.96333	COG0604	S:Function unknown	GO:0016491	MF:oxidoreductase activity;
BAB_RS16100	-	prephenate dehydratase	1.12	0.164024053	0.013618554	0.048456714	no	up	mRNA	85.44	75.61	88.34	88.01	89.24	85.64	83.13	87.63	COG0077	E:Amino acid transport and metabolism	GO:0009094;GO:0009094;GO:0005737;GO:0005737;GO:0004106;GO:0004664;GO:0004664;GO:0004106	BP:L-phenylalanine biosynthetic process;BP:L-phenylalanine biosynthetic process;CC:cytoplasm;CC:cytoplasm;MF:chorismate mutase activity;MF:prephenate dehydratase activity;MF:prephenate dehydratase activity;MF:chorismate mutase activity;
BAB_RS24710	-	ribosome silencing factor	1.12	0.163442903	0.02040666	0.065697993	no	up	mRNA	2277.54	2294.23	2306.04	2266.56	2598.34	2519.14	2292.6	2461.3467	COG0799	S:Function unknown	GO:0042256;GO:0090071;GO:0017148;GO:0090071;GO:0042256;GO:0017148;GO:0005737;GO:0005737	BP:mature ribosome assembly;BP:negative regulation of ribosome biogenesis;BP:negative regulation of translation;BP:negative regulation of ribosome biogenesis;BP:mature ribosome assembly;BP:negative regulation of translation;CC:cytoplasm;CC:cytoplasm;
BAB_RS21660	-	DUF1624 domain-containing protein	1.12	0.163512645	0.021102258	0.066997585	no	up	mRNA	63.73	58.47	64.07	63.47	69.22	63.76	62.09	65.483333	COG3503	S:Function unknown	GO:0016021	CC:integral component of membrane;

BAB_RS22850	-	UDP-N-acetylmuramoyl-L-alanine--D-glutamate ligase	1.12	0.163049277	0.025314421	0.076156586	no	up	mRNA	88.54	84.59	96.45	87.38	99.22	97.8	89.86	94.8	COG0771	M:Cell wall/membrane/envelope biogenesis	[illegible]	[illegible]
BAB_RS21455	-	enolase	1.119	0.162280188	0.008183671	0.034780602	no	up	mRNA	316.02	290.61	305.65	313.31	324.17	325.22	304.093	320.9	COG0148	G:Carbohydrate transport and metabolism	[illegible]	[illegible]
BAB_RS25925	-	adenosylhomocysteinase	1.119	0.162220204	0.030982533	0.087092633	no	up	mRNA	1024.26	901.8	936.46	1059.19	964.35	996.04	954.173	1006.5267	COG0499	H:Coenzyme transport and metabolism	[illegible]	[illegible]
BAB_RS16315	-	RNA 2'%2C3'-cyclic phosphodiesterase	1.119	0.162632788	0.044262023	0.113771103	no	up	mRNA	395.83	386.19	402.9	369.08	445.73	443.38	394.973	419.39667	COG1514	J:Translation, ribosomal structure and biogenesis	[illegible]	[illegible]
BAB_RS16485	-	porin family protein	1.119	0.162557157	0.051482535	0.126850755	no	up	mRNA	7278.09	6024.28	6391.58	6548.27	7344.24	6975.13	6564.65	6955.88	COG3637	M:Cell wall/membrane/envelope biogenesis	GO:0019867	CC:outer membrane;
BAB_RS24925	-	hypothetical protein	1.119	0.161586912	0.070885967	0.160731555	no	up	mRNA	331.84	380.36	423.89	377.43	424.75	396.49	378.697	399.55667	COG4223	S:Function unknown	------	------
BAB_RS28920	-	ABC transporter ATP-binding protein	1.119	0.161796581	0.07784212	0.172964544	no	up	mRNA	40.7	36.4	38.07	43.08	41.77	36.31	38.39	40.386667	COG3842	S:Function unknown	[illegible]	[illegible]
BAB_RS19590	-	amino acid ABC transporter permease	1.119	0.162417911	0.367928828	0.533945494	no	up	mRNA	6.51	4.48	5.26	6.3	4.91	5.77	5.41667	5.66	COG0765	E:Amino acid transport and metabolism	[illegible]	[illegible]
BAB_RS31905	rnpB	-	1.119	0.161670967	0.37255017	0.537979189	no	up	mRNA	31959.2	34479.4	53603	42623.16	46440.84	39775.12	40013.8	42946.373	ENOG410Y4Y0	S:Function unknown	------	------
BAB_RS22470	-	TonB-dependent receptor	1.118	0.160382494	0.014525474	0.050839158	no	up	mRNA	135.94	134.89	135.72	144.6	138.25	145.32	135.517	142.72333	COG4206	P:Inorganic ion transport and metabolism	[illegible]	[illegible]
BAB_RS29435	-	BA14K family protein	1.118	0.161086838	0.01604554	0.054728579	no	up	mRNA	627.47	603.1	684.59	639.62	663.12	717.04	638.387	673.26	ENOG41122WP	S:Function unknown	[illegible]	[illegible]
BAB_RS18155	-	ABC transporter ATP-binding protein/permease	1.118	0.16121542	0.035888131	0.097224759	no	up	mRNA	25.82	25.56	25.2	27.02	26.44	27.22	25.5267	26.893333	COG5265	V:Defense mechanisms	[illegible]	[illegible]
BAB_RS22750	-	serine/threonine protein kinase	1.118	0.160998345	0.0730039	0.164951851	no	up	mRNA	28.92	27.75	27.88	31.55	29.26	28.32	28.1833	29.71	ENOG410ZKTU	T:Signal transduction mechanisms	[illegible]	[illegible]
BAB_RS28255	-	hypothetical protein	1.118	0.161281603	0.186335788	0.324672932	no	up	mRNA	194.53	146.59	193.42	212.23	194.27	154.22	178.18	186.90667	COG0732	V:Defense mechanisms	[illegible]	[illegible]
BAB_RS31080	-	NAD(P)-dependent oxidoreductase	1.117	0.159420336	0.076081281	0.170824009	no	up	mRNA	43.8	45.07	50.93	46.41	47.99	53.66	46.6	49.353333	COG0451	C:Energy production and conversion	------	------
BAB_RS16135	-	cytochrome o ubiquinol oxidase subunit IV	1.117	0.159569805	0.217960103	0.365122947	no	up	mRNA	41.93	46.19	42.05	45.21	52.33	40.88	43.39	46.14	COG3125	C:Energy production and conversion	[illegible]	[illegible]
BAB_RS20205	-	arginine--tRNA ligase	1.116	0.15789077	0.006923144	0.031154149	no	up	mRNA	150.76	136.27	150.65	151.8	152.78	154.98	145.893	153.18667	COG0018	J:Translation, ribosomal structure and biogenesis	[illegible]	[illegible]
BAB_RS18250	-	guanylate kinase	1.116	0.157904298	0.012584002	0.046155706	no	up	mRNA	99.41	89.74	107.66	102.31	106.3	102.68	98.9367	103.76333	COG0194	F:Nucleotide transport and metabolism	[illegible]	[illegible]
BAB_RS31060	-	complex I NDUFA9 subunit family protein	1.116	0.157992235	0.012861016	0.046797787	no	up	mRNA	193.09	165.45	188.19	188.87	188.66	196.31	182.243	191.28	COG0702;COG0702	G:Carbohydrate transport and metabolism;M:Cell wall/membrane/envelope biogenesis	[illegible]	[illegible]
BAB_RS20965	-	hypothetical protein	1.116	0.158364348	0.02293738	0.070931475	no	up	mRNA	231.79	198.75	213.65	227.45	221.91	231.13	214.73	226.83	ENOG4111XB7;ENOG4111IVK	S:Function unknown;	------	------
BAB_RS20805	-	RluA family pseudouridine synthase	1.116	0.158185197	0.060581512	0.142808803	no	up	mRNA	48.86	42.27	45.24	50.58	44.66	48.01	45.4567	47.75	COG0564	J:Translation, ribosomal structure and biogenesis	[illegible]	[illegible]
BAB_RS16920	-	ABC transporter ATP-binding protein	1.116	0.158930048	0.354692379	0.520139943	no	up	mRNA	7.5	7.36	6.98	9.01	6.57	7.48	7.28	7.6866667	COG0715	P:Inorganic ion transport and metabolism	[illegible]	[illegible]
BAB_RS29600	-	IS66 family insertion sequence hypothetical protein	1.116	0.157870003	0.496217336	0.652351187	no	up	mRNA	11.28	10.59	19.06	13.35	17	13.84	13.6433	14.73	ENOG41122WU;COG2963;ENOG410XU4Z	L:Replication, recombination and repair;	[illegible]	[illegible]
BAB_RS17505	-	GcrA cell cycle regulator	1.115	0.15736945	0.018712826	0.061665959	no	up	mRNA	215.88	210.27	240.77	225.02	239.52	239.11	222.307	234.55	COG5352	T:Signal transduction mechanisms	------	------
BAB_RS31110	-	2%2C3-bisphosphoglycerate-dependent phosphoglycerate mutase	1.115	0.156790861	0.021380868	0.067748252	no	up	mRNA	240.91	209.66	219.81	231.25	238.28	236.89	223.46	235.47333	COG0588	G:Carbohydrate transport and metabolism	[illegible]	[illegible]
BAB_RS26255	-	tRNA pseudouridine(55) synthase TruB	1.115	0.156971092	0.059040407	0.141038535	no	up	mRNA	98.27	84.92	99.65	108.48	92.58	96.08	94.28	99.046667	COG0130	J:Translation, ribosomal structure and biogenesis	[illegible]	[illegible]
BAB_RS18945	-	hypothetical protein	1.115	0.157537432	0.064808043	0.149912342	no	up	mRNA	98.25	108.13	106.56	112.79	110.76	109.9	104.313	111.15	COG4520	M:Cell wall/membrane/envelope biogenesis	------	------
BAB_RS28340	-	hypothetical protein	1.115	0.156918393	0.091327399	0.193558663	no	up	mRNA	31.94	26.8	32.44	30.36	33.37	32	30.3933	31.91	COG1993	S:Function unknown	------	------
BAB_RS19540	-	TIR domain-containing protein	1.115	0.157495017	0.143490771	0.270085441	no	up	mRNA	12.87	11.12	15.6	13.45	13.61	14.53	13.1967	13.863333	ENOG410ZXCB;COG4916	S:Function unknown;	GO:0007165	BP:signal transduction;
BAB_RS31825	-	heme transporter BhuA	1.115	0.1566277	0.203024709	0.346793402	no	up	mRNA	22.79	23.4	20.13	25.02	24.9	19.65	22.1067	23.19	COG1629	G:Carbohydrate transport and metabolism	[illegible]	[illegible]
BAB_RS16500	-	DNA polymerase I	1.114	0.155377086	0.012259383	0.04532727	no	up	mRNA	40.4	37.82	43.11	42.25	43.02	41.73	40.4433	42.333333	COG0258	L:Replication, recombination and repair	[illegible]	[illegible]
BAB_RS22145	-	type I methionyl aminopeptidase	1.114	0.155357913	0.013715979	0.048588137	no	up	mRNA	242.04	213.87	233.86	236.36	247.48	241.48	229.923	241.77333	COG0024	J:Translation, ribosomal structure and biogenesis	[illegible]	[illegible]
BAB_RS31955	-	ubiquinol oxidase	1.114	0.156077226	0.077070673	0.171963938	no	up	mRNA	56.84	53.53	58.64	64.85	58.59	54.54	56.3367	59.326667	COG0843	C:Energy production and conversion	[illegible]	[illegible]
BAB_RS24040	-	phosphoglycerate dehydrogenase	1.114	0.156191527	0.084563608	0.182718812	no	up	mRNA	192.79	156.08	162.15	163.36	186.11	186.4	170.34	178.62333	COG0111	S:Function unknown	[illegible]	[illegible]
BAB_RS29370	-	glycerol-3-phosphate acyltransferase	1.114	0.155632665	0.114056308	0.226771606	no	up	mRNA	37.87	34.51	37.77	38.27	39.64	38.48	36.7167	38.796667	COG0344	S:Function unknown	[illegible]	[illegible]
BAB_RS23090	-	flagellar biosynthesis protein FlgM	1.113	0.154603756	0.011176147	0.042799716	no	up	mRNA	212.39	206.75	229.03	221.69	230.62	229.24	216.057	227.18333	COG2321	S:Function unknown	[illegible]	[illegible]
BAB_RS21345	clpP	ATP-dependent Clp protease proteolytic subunit	1.113	0.154394815	0.011947817	0.044637602	no	up	mRNA	429.91	381.96	406.13	423.94	437.13	419.87	406	426.98	COG0740	O:Posttranslational modification, protein turnover, chaperones	[illegible]	[illegible]
BAB_RS29490	-	DsbA family protein	1.113	0.154392804	0.03243539	0.089995604	no	up	mRNA	115.48	110.32	121.16	111.15	124.72	128.31	115.653	121.39333	COG2761	Q:Secondary metabolites biosynthesis, transport and catabolism	[illegible]	[illegible]
BAB_RS18220	-	[acyl-carrier-protein] S-malonyltransferase	1.113	0.15467647	0.047835759	0.120357316	no	up	mRNA	70.46	66.23	70.28	78.07	70.57	68.78	68.99	72.473333	COG0331	I:Lipid transport and metabolism	[illegible]	[illegible]
BAB_RS30985	-	acetylglutamate kinase	1.113	0.1539148	0.068649182	0.157325123	no	up	mRNA	72.32	60.19	64.89	72.21	65.3	69.39	65.8	68.966667	COG0548	E:Amino acid transport and metabolism	[illegible]	[illegible]
BAB_RS30100	-	acyl-CoA dehydrogenase	1.113	0.154003923	0.343876376	0.507988412	no	up	mRNA	10.6	7.07	12.02	10.01	11.56	9.28	9.89667	10.283333	COG1960	S:Function unknown	[illegible]	[illegible]
BAB_RS31370	-	ABC transporter permease	1.113	0.154930044	0.355633545	0.521282199	no	up	mRNA	6.77	7.94	7.3	8.09	8.03	7.19	7.33667	7.77	COG1177	S:Function unknown	[illegible]	[illegible]
BAB_RS31600	-	chemotaxis protein MotC	1.113	0.154760579	0.468048579	0.631070115	no	up	mRNA	2.06	1.67	2.05	2.08	2.05	1.9	1.92667	2.01	ENOG410XZW1	S:Function unknown	------	------
BAB_RS30455	-	hypothetical protein	1.113	0.153844758	0.59661538	0.733610874	no	up	mRNA	13.23	6.77	15.03	13.87	14.15	8.71	11.6767	12.243333	ENOG410Y1R3	S:Function unknown	------	------
BAB_RS27465	-	DUF2778 domain-containing protein	1.113	0.153984816	0.725006165	0.830757777	no	up	mRNA	0.62	1.1	1.27	1.25	1.47	0.46	0.99667	1.06	ENOG4111KE8	S:Function unknown	GO:0016021	CC:integral component of membrane;
BAB_RS22865	-	UDP-N-acetylmuramoyl-L-alanyl-D-glutamate--2%2C6-diaminopimelate ligase	1.112	0.152866	0.016904474	0.056932993	no	up	mRNA	115.08	108.34	128.83	122.62	121.21	124.93	117.417	122.92	COG0769	M:Cell wall/membrane/envelope biogenesis	[illegible]	[illegible]
BAB_RS27295	-	hypothetical protein	1.111	0.152118731	0.009363983	0.03789229	no	up	mRNA	587.85	551.11	574.58	604.71	594.29	602.03	571.18	600.34333	ENOG41123B6	S:Function unknown	------	------
BAB_RS25625	-	D-alanyl-D-alanine carboxypeptidase	1.111	0.151552012	0.010795949	0.041842442	no	up	mRNA	231.15	227.91	247.57	252.59	242.38	245.75	235.543	246.90667	COG1686	O:Posttranslational modification, protein turnover, chaperones	GO:0009002	MF:serine-type D-Ala-D-Ala carboxypeptidase activity;
BAB_RS28685	-	hypothetical protein	1.111	0.151551789	0.043483228	0.112308368	no	up	mRNA	72.02	67.92	85.1	74.28	80.84	79.99	75.0133	78.37	COG5476	S:Function unknown	[illegible]	[illegible]
BAB_RS26825	-	type III PLP-dependent enzyme	1.111	0.152068515	0.080651502	0.177732013	no	up	mRNA	27.63	28.14	31.05	33.1	27.44	30.4	28.94	30.313333	COG0019	K:Transcription	[illegible]	[illegible]
BAB_RS24170	-	ABC transporter	1.11	0.150775911	0.018451643	0.06099293	no	up	mRNA	78.28	73.32	82.38	83.04	84.85	76.22	77.9933	81.37	COG1132	N:Cell motility	[illegible]	[illegible]
BAB_RS18235	-	beta-ketoacyl-[acyl-carrier-protein] synthase II	1.11	0.149962036	0.03262258	0.090203399	no	up	mRNA	278.46	257	281.39	308.46	276.03	269.52	272.283	284.67	COG0304	I:Lipid transport and metabolism	[illegible]	[illegible]
BAB_RS18885	-	gene transfer agent family protein	1.11	0.15011743	0.829990439	0.896088468	no	up	mRNA	0.81	1.71	1.86	0.95	2.44	1.54	1.46	1.6433333	ENOG4111YM5;ENOG410ZZBM	S:Function unknown;	------	------
BAB_RS20260	-	hypothetical protein	1.11	0.150721507	0.863334593	0.916989767	no	up	mRNA	5.17	8.82	1.21	2.83	2.49	10.93	5.06667	5.4166667	------	------	GO:0016021	CC:integral component of membrane;
BAB_RS30890	-	3'-5' exonuclease	1.109	0.149850178	0.023871915	0.073257366	no	up	mRNA	238.81	209	230.07	239.63	245.05	223.18	225.96	235.95333	COG0847	C:Energy production and conversion	[illegible]	[illegible]
BAB_RS22860	-	UDP-N-acetylmuramoylalanyl-D-glutamyl-2%2C 6-diaminopimelate--D-alanyl-D-alanine ligase	1.109	0.148922425	0.029763939	0.08425686	no	up	mRNA	106.81	109.98	112.68	112.14	112.23	120.44	109.823	114.93667	COG0770	M:Cell wall/membrane/envelope biogenesis	[illegible]	[illegible]
BAB_RS25650	-	DUF4354 domain-containing protein	1.109	0.149315157	0.055472375	0.134515276	no	up	mRNA	145.48	133.66	134.6	132.8	145.58	154.52	137.913	144.3	ENOG410Y1EF	S:Function unknown	GO:0005524	MF:ATP binding;
BAB_RS19220	-	folate-binding protein	1.109	0.148789684	0.264651765	0.419706871	no	up	mRNA	31.11	24.56	28.68	34.05	29.1	24.64	28.1167	29.263333	COG0354	S:Function unknown	[illegible]	[illegible]
BAB_RS17665	-	carboxynorspermidine decarboxylase	1.109	0.149528507	0.277716157	0.433366689	no	up	mRNA	26.68	19.7	21.38	27.22	21.19	21.94	22.5867	23.45	COG0019	E:Amino acid transport and metabolism	[illegible]	[illegible]
BAB_RS17515	-	ornithine carbamoyltransferase	1.108	0.147944583	0.024568705	0.074611766	no	up	mRNA	117.56	107.01	121.82	120.56	124.59	117.02	115.463	120.72333	COG0078	E:Amino acid transport and metabolism	[illegible]	[illegible]
BAB_RS25120	-	succinyl-CoA ligase subunit beta	1.108	0.147797523	0.031363991	0.088087852	no	up	mRNA	1143.05	959.01	1069.68	1099.06	1085.77	1125.26	1057.25	1103.3633	COG0045	C:Energy production and conversion	[illegible]	[illegible]
BAB_RS25410	-	prephenate dehydrogenase/arogenate dehydrogenase family protein	1.108	0.14835522	0.035020441	0.095195157	no	up	mRNA	76.03	66.11	77.79	77.69	76.16	75.92	73.31	76.59	COG0287	E:Amino acid transport and metabolism	[illegible]	[illegible]
BAB_RS32435	-	hypothetical protein	1.108	0.148511508	0.05696713	0.137207938	no	up	mRNA	37.29	34.06	39.85	38.51	41.25	36.17	37.0667	38.643333	------	------	------	------
BAB_RS25615	-	response regulator SirA	1.108	0.147374004	0.457193251	0.620600725	no	up	mRNA	20.85	19.46	21.19	28.35	17.8	18.94	20.5	21.696667	COG0425	O:Posttranslational modification, protein turnover, chaperones	------	------
BAB_RS31180	-	ubiquinone/menaquinone biosynthesis C-methyltransferase UbiE	1.107	0.146712145	0.024731535	0.074823374	no	up	mRNA	332.37	298.56	325.51	348.13	336.65	313.6	318.813	332.79333	COG2226	H:Coenzyme transport and metabolism	[illegible]	[illegible]
BAB_RS22875	-	hypothetical protein	1.107	0.146811915	0.091019639	0.19303373	no	up	mRNA	209.2	178.17	180.4	206.61	184.79	210.45	189.257	200.61667	COG5462	S:Function unknown	------	------
BAB_RS27560	-	hypothetical protein	1.107	0.147091085	0.128691248	0.247892675	no	up	mRNA	385.98	360.59	330.42	353.24	435.94	383.78	358.997	390.98667	COG1846;ENOG410XUB6;ENOG4111JSB	S:Function unknown;K:Transcription;K:Transcription	[illegible]	[illegible]
BAB_RS19135	-	uracil-DNA glycosylase	1.107	0.147068874	0.144194438	0.270775412	no	up	mRNA	41.84	43.47	37.93	43.75	40.54	44.86	41.08	43.05	COG1573	O:Posttranslational modification, protein turnover, chaperones	------	------
BAB_RS24645	-	lytic murein transglycosylase	1.106	0.145420108	0.019015124	0.062342443	no	up	mRNA	295.83	265.98	279.33	292.1	288.43	298.78	280.38	293.10333	COG2951	U:Intracellular trafficking, secretion, and vesicular transport	------	------
BAB_RS24095	-	hypothetical protein	1.106	0.145778361	0.028647661	0.081890511	no	up	mRNA	170.51	151.39	169.17	179	163.21	169.88	163.69	170.69667	ENOG4111M5S	S:Function unknown	------	------
BAB_RS27880	lldD	alpha-hydroxy-acid oxidizing protein	1.106	0.145294748	0.064920184	0.149955823	no	up	mRNA	56.37	50.01	51.7	51.37	55.84	57.39	52.6933	54.866667	COG1304	C:Energy production and conversion	[illegible]	[illegible]
BAB_RS22760	-	hypothetical protein	1.106	0.144781231	0.127193337	0.24603985	no	up	mRNA	60.95	54.88	53.52	53.76	64.52	58.04	56.45	58.773333	COG3045	S:Function unknown	------	------
BAB_RS22880	-	ribosomal RNA small subunit methyltransferase H	1.105	0.143646776	0.029006636	0.082842953	no	up	mRNA	112.24	111.08	121.73	119.83	113.95	125.59	115.017	119.79	COG0275	M:Cell wall/membrane/envelope biogenesis	[illegible]	[illegible]
BAB_RS22345	-	mechanosensitive ion channel family protein	1.105	0.143716442	0.033119578	0.091420278	no	up	mRNA	59.44	57.28	58.1	60.13	63.07	58.6	58.2733	60.6	COG3264	M:Cell wall/membrane/envelope biogenesis	[illegible]	[illegible]
BAB_RS16580	-	NifU family protein	1.105	0.143398386	0.052128496	0.128343951	no	up	mRNA	219.34	179.7	214.5	221.63	217.08	198.42	204.513	212.37667	COG0694	O:Posttranslational modification, protein turnover, chaperones	[illegible]	[illegible]
BAB_RS27225	-	LysE family translocator	1.105	0.144296254	0.068662572	0.157243652	no	up	mRNA	64.83	58.32	62.1	63.23	65.45	65.17	61.75	64.616667	COG1280;ENOG41120U4	L:Replication, recombination and repair;S:Function unknown	[illegible]	[illegible]
BAB_RS20320	-	trigger factor	1.105	0.144608725	0.083677396	0.18190492	no	up	mRNA	758.92	622.72	658.71	750.89	706.92	666.74	680.117	708.18333	COG0544	O:Posttranslational modification, protein turnover, chaperones	[illegible]	[illegible]
BAB_RS29310	-	nucleoside diphosphate kinase regulator	1.105	0.144645527	0.085323107	0.183865287	no	up	mRNA	41.3	37.45	40.24	41.1	44.37	38.34	39.6633	41.27	COG0782	S:Function unknown	[illegible]	[illegible]
BAB_RS16445	-	DUF3131 domain-containing protein	1.104	0.143216221	0.014163648	0.049735303	no	up	mRNA	104.36	100.62	109.92	106.88	110.12	109.77	104.967	108.92333	COG3459	G:Carbohydrate transport and metabolism	[illegible]	[illegible]
BAB_RS18420	-	pyruvate%2C phosphate dikinase	1.104	0.142792557	0.029789096	0.084253842	no	up	mRNA	59.87	59.58	64.94	66.92	60.95	63.74	61.4633	63.87	COG0574	G:Carbohydrate transport and metabolism	[illegible]	[illegible]
BAB_RS19875	-	NADH-quinone oxidoreductase subunit NuoE	1.103	0.141006613	0.012756087	0.046574213	no	up	mRNA	445.1	415.57	450.86	444.41	456.65	468.8	437.177	456.62	COG1905;COG3743	C:Energy production and conversion;	[illegible]	[illegible]
BAB_RS20350	-	acetyl-CoA carboxylase biotin carboxylase subunit	1.103	0.141091861	0.014801052	0.051245453	no	up	mRNA	206.13	195.67	204.24	207.32	213.65	209.16	202.013	210.04333	COG0439	I:Lipid transport and metabolism	[illegible]	[illegible]
BAB_RS26375	-	replication initiation protein	1.103	0.142081081	0.030465491	0.085864582	no	up	mRNA	77.72	70.69	82.49	82.43	78.82	78.68	76.9667	79.976667	ENOG410ZBH0	L:Replication, recombination and repair	------	------
BAB_RS21030	-	universal stress protein	1.103	0.141973882	0.032501386	0.090100909	no	up	mRNA	454.59	441.34	490.58	444.65	504.43	497.18	462.17	482.08667	COG0589	F:Nucleotide transport and metabolism	GO:0006950	BP:response to stress;
BAB_RS16475	-	penicillin-binding protein	1.103	0.141664933	0.036102419	0.097640635	no	up	mRNA	91.16	81.92	89.66	91.28	85.16	96.18	87.58	90.873333	COG0744	M:Cell wall/membrane/envelope biogenesis	[illegible]	[illegible]
BAB_RS21635	-	D-alanyl-D-alanine carboxypeptidase	1.102	0.140645424	0.021384073	0.067691651	no	up	mRNA	387.79	386.36	415.91	396.67	410.49	429.44	396.687	412.2	COG1686	O:Posttranslational modification, protein turnover, chaperones	[illegible]	[illegible]
BAB_RS21200	-	hypothetical protein	1.102	0.139654425	0.344096416	0.508079864	no	up	mRNA	27.77	31.22	31.29	34.85	31.37	29.86	30.0933	32.026667	------	------	------	------
BAB_RS19415	-	phosphoribosylglycinamide formyltransferase	1.102	0.140477193	0.382099612	0.547341085	no	up	mRNA	15.03	14.17	18.38	18.43	14.36	17.49	15.86	16.76	COG0299	F:Nucleotide transport and metabolism	[illegible]	[illegible]
BAB_RS19295	-	molecular chaperone SurA	1.101	0.138324354	0.034477685	0.094117928	no	up	mRNA	357.05	323.47	343.35	372.84	336.17	354.52	341.29	354.51	COG0760	O:Posttranslational modification, protein turnover, chaperones	GO:0016853	MF:isomerase activity;
BAB_RS21710	-	acetyl/propionyl/methylcrotonyl-CoA carboxylase subunit alpha	1.101	0.139130492	0.132953587	0.255032761	no	up	mRNA	12.71	13.94	14.02	13.38	14.02	14.92	13.5567	14.106667	COG4770	K:Transcription	[illegible]	[illegible]
BAB_RS25720	-	ABC transporter ATP-binding protein	1.101	0.138260994	0.142917184	0.269321357	no	up	mRNA	49.24	44.05	47.46	52.69	49.78	43.61	46.9167	48.693333	COG1101	S:Function unknown	[illegible]	[illegible]
BAB_RS23380	-	hypothetical protein	1.101	0.139278577	0.248824985	0.402352631	no	up	mRNA	142.21	152.41	185.48	137.56	189.29	173.07	160.033	166.64	ENOG411295W	S:Function unknown	------	------
BAB_RS31155	-	hypothetical protein	1.1	0.136959422	0.020648348	0.066210721	no	up	mRNA	2571.49	2325.06	2136.36	2364.28	2546.25	2577.66	2344.3	2496.0633	COG3247	S:Function unknown	GO:0016021	CC:integral component of membrane;
BAB_RS24885	-	carbon-nitrogen hydrolase family protein	1.1	0.138045359	0.031471067	0.088080608	no	up	mRNA	323.3	271.92	307.27	313.13	318.04	305.99	300.83	312.38667	COG0388	S:Function unknown	[illegible]	[illegible]
BAB_RS27855	-	cupin domain-containing protein	1.1	0.136857272	0.03907721	0.10393632	no	up	mRNA	276.21	274.78	316.9	289.2	317.76	290.59	289.297	299.18333	ENOG4111ISD	K:Transcription	GO:0043565	MF:sequence-specific DNA binding;
BAB_RS26300	-	MetQ/NlpA family ABC transporter substrate-binding protein	1.1	0.137722236	0.041685631	0.108714231	no	up	mRNA	62.27	55.18	62.63	64.97	60.52	60.86	60.0267	62.116667	COG1464	P:Inorganic ion transport and metabolism	------	------
BAB_RS18095	-	acetoin utilization protein	1.1	0.137206426	0.076859805	0.171731957	no	up	mRNA	112.61	103.33	120.77	105.48	124.37	119.64	112.237	116.49667	COG0123;COG0123	B:Chromatin structure and dynamics;Q:Secondary metabolites biosynthesis, transport and catabolism	------	------
BAB_RS17890	-	ATP synthase subunit A	1.1	0.137449636	0.100918139	0.207453602	no	up	mRNA	207.95	171.65	181.68	205.16	180.02	197.7	187.093	194.29333	COG0356	C:Energy production and conversion	[illegible]	[illegible]
BAB_RS29960	-	DNA-binding response regulator	1.1	0.137826373	0.166215422	0.300365664	no	up	mRNA	44.59	51.48	55.23	48.63	54.86	54.87	50.4333	52.786667	COG0745	S:Function unknown	[illegible]	[illegible]
BAB_RS16310	-	protein-tyrosine-phosphatase	1.1	0.138048257	0.303755026	0.463861643	no	up	mRNA	19.19	16.48	25.77	20.28	23.92	19.3	20.48	21.166667	COG0394	T:Signal transduction mechanisms	------	------
BAB_RS21255	-	MexH family multidrug efflux RND transporter periplasmic adaptor subunit	1.099	0.135710948	0.017943851	0.059498033	no	up	mRNA	332.49	322.58	351.03	335.91	358.51	348.5	335.367	347.64	COG0845	C:Energy production and conversion	[illegible]	[illegible]
BAB_RS20795	-	phosphoribosyl 1%2C2-cyclic phosphodiesterase	1.099	0.135977696	0.102715092	0.210206108	no	up	mRNA	30.89	27.1	30.9	32.48	29.13	30.38	29.63	30.663333	COG1235	S:Function unknown	[illegible]	[illegible]
BAB_RS16005	-	enoyl-CoA hydratase	1.099	0.136736825	0.157820828	0.289263161	no	up	mRNA	42.08	39.43	48.86	44.07	49.05	42.49	43.4567	45.203333	COG1024	I:Lipid transport and metabolism	[illegible]	[illegible]
BAB_RS31570	flgL	flagellar hook-associated protein FlgL	1.099	0.136375093	0.509405036	0.662907404	no	up	mRNA	3.76	2.94	4.35	4.49	3.47	3.52	3.68333	3.8266667	COG1344	N:Cell motility	[illegible]	[illegible]
BAB_RS27900	-	efflux RND transporter periplasmic adaptor subunit	1.098	0.13461425	0.077360179	0.172251042	no	up	mRNA	50.78	53.22	58.96	56	55.99	57.39	54.32	56.46	COG0845	M:Cell wall/membrane/envelope biogenesis	[illegible]	[illegible]
BAB_RS20435	-	thioredoxin-dependent thiol peroxidase	1.098	0.13435512	0.101724447	0.208577311	no	up	mRNA	77.16	82.9	80.56	81.45	81.9	87.8	80.2067	83.716667	COG1225	O:Posttranslational modification, protein turnover, chaperones	[illegible]	[illegible]
BAB_RS17685	-	MATE family efflux transporter	1.098	0.134596149	0.211957792	0.357303456	no	up	mRNA	52.88	45.72	67.88	55.3	62.18	53.93	55.4933	57.136667	COG0534	V:Defense mechanisms	[illegible]	[illegible]
BAB_RS29650	-	UDP-glucose/GDP-mannose dehydrogenase family protein	1.098	0.135294857	0.440999404	0.606562964	no	up	mRNA	4.61	4.15	5.02	4.23	4.23	5.9	4.59333	4.7866667	COG1004	M:Cell wall/membrane/envelope biogenesis	[illegible]	[illegible]
BAB_RS24320	-	pyruvate kinase	1.097	0.133676416	0.019117464	0.062550317	no	up	mRNA	116.58	104.33	114.83	114.92	116.09	115.37	111.913	115.46	COG0469	G:Carbohydrate transport and metabolism	[illegible]	[illegible]
BAB_RS29555	-	bacterioferritin	1.097	0.134000258	0.034558577	0.094258665	no	up	mRNA	1468.02	1261.53	1417.07	1439.19	1390.35	1508.38	1382.21	1445.9733	COG2193	P:Inorganic ion transport and metabolism	[illegible]	[illegible]
BAB_RS26095	-	glutathione synthetase	1.097	0.133111776	0.063483349	0.148235465	no	up	mRNA	87.7	82.21	82.51	89.93	84.03	87.33	84.14	87.096667	COG0189;COG0189	H:Coenzyme transport and metabolism;J:Translation, ribosomal structure and biogenesis	[illegible]	[illegible]
BAB_RS29865	-	hypothetical protein	1.097	0.133422164	0.142236916	0.268511874	no	up	mRNA	36.9	35.55	36.78	33.1	39.2	40.68	36.41	37.66	COG3560	S:Function unknown	[illegible]	[illegible]
BAB_RS28985	-	MFS transporter	1.096	0.131820418	0.05977606	0.141742052	no	up	mRNA	35.29	33.42	37.64	33.9	38.45	37.24	35.45	36.53	ENOG410XNN3	G:Carbohydrate transport and metabolism	[illegible]	[illegible]
BAB_RS27850	-	aspartate aminotransferase family protein	1.096	0.13159313	0.090346266	0.192112873	no	up	mRNA	29.1	27.06	29.68	27.32	31.33	29.83	28.6133	29.493333	COG0161	Q:Secondary metabolites biosynthesis, transport and catabolism	[illegible]	[illegible]
BAB_RS18310	-	homoserine kinase	1.096	0.132416087	0.189565306	0.328518515	no	up	mRNA	43.01	38.9	46.32	50.24	42.36	40.03	42.7433	44.21	COG2334	S:Function unknown	[illegible]	[illegible]
BAB_RS24150	-	elongation factor P	1.096	0.132391853	0.19635323	0.337912655	no	up	mRNA	265.89	209.2	230.38	276.62	230.74	222.44	235.157	243.26667	COG0231	J:Translation, ribosomal structure and biogenesis	[illegible]	[illegible]
BAB_RS22505	-	urease subunit alpha 2	1.095	0.131460433	0.026155964	0.077384082	no	up	mRNA	163.79	163.02	166.7	167.4	172.84	169.39	164.503	169.87667	COG0804	S:Function unknown	[illegible]	[illegible]
BAB_RS21275	-	peptidylprolyl isomerase	1.095	0.130382731	0.027607675	0.080202043	no	up	mRNA	777.79	740.85	772.35	765.36	839.14	780.87	763.663	795.12333	COG0652	O:Posttranslational modification, protein turnover, chaperones	[illegible]	[illegible]
BAB_RS16685	-	heat-shock protein Hsp20	1.095	0.130645384	0.042462504	0.109937167	no	up	mRNA	3249.47	2758.27	2997.13	3185.75	3198.9	3009.17	3001.62	3131.2733	COG0071	O:Posttranslational modification, protein turnover, chaperones	------	------
BAB_RS16550	-	sensor histidine kinase	1.095	0.130681088	0.175068324	0.310427442	no	up	mRNA	17.71	18.45	17.08	18.19	18.35	18.48	17.7467	18.34	ENOG410XP2A	T:Signal transduction mechanisms	[illegible]	[illegible]
BAB_RS30705	-	hypothetical protein	1.095	0.131322037	0.32170991	0.484601004	no	up	mRNA	43.85	42.06	45.5	40.55	53.73	42.89	43.8033	45.723333	------	------	------	------
BAB_RS17285	-	histidinol dehydrogenase	1.094	0.129222862	0.025962885	0.077096811	no	up	mRNA	253.45	238.23	256.56	246.28	267.71	257.56	249.413	257.18333	COG0141	E:Amino acid transport and metabolism	[illegible]	[illegible]
BAB_RS19865	-	NADH-quinone oxidoreductase subunit C	1.094	0.129571958	0.053401132	0.13067619	no	up	mRNA	477.37	439.78	479.52	517.31	454.63	476.1	465.557	482.68	COG0852	C:Energy production and conversion	[illegible]	[illegible]
BAB_RS26010	-	benzoate transporter	1.094	0.129821475	0.118199579	0.232990948	no	up	mRNA	37.49	37.52	40.06	38.66	41.39	39.15	38.3567	39.733333	COG3135	Q:Secondary metabolites biosynthesis, transport and catabolism	[illegible]	[illegible]
BAB_RS24950	-	hypothetical protein	1.094	0.129252533	0.257434947	0.411511684	no	up	mRNA	162.53	140.88	145.89	157.7	157.35	169.54	149.767	161.53	COG2350	S:Function unknown	------	------
BAB_RS27335	-	patatin	1.093	0.127970729	0.059380968	0.141431469	no	up	mRNA	29.08	27.02	30.44	29.93	29.11	29.98	28.8467	29.673333	COG1752	O:Posttranslational modification, protein turnover, chaperones	[illegible]	[illegible]
BAB_RS22770	-	SCO family protein	1.092	0.126549002	0.050789854	0.12611113	no	up	mRNA	355.59	330.66	326.01	337.4	359.14	350.15	337.42	348.89667	COG1999	S:Function unknown	GO:0016021	CC:integral component of membrane;
BAB_RS19345	-	META domain-containing protein	1.092	0.127423436	0.051192659	0.126524625	no	up	mRNA	383.04	354.02	373.36	402.59	386.9	357.15	370.14	382.21333	COG3187	O:Posttranslational modification, protein turnover, chaperones	GO:0016021	CC:integral component of membrane;
BAB_RS19190	-	signal peptidase I	1.092	0.126436704	0.051081293	0.126541398	no	up	mRNA	281.88	252.63	265.54	283.92	263.46	278.52	266.683	275.3	COG0681	U:Intracellular trafficking, secretion, and vesicular transport	[illegible]	[illegible]
BAB_RS24880	-	DUF1178 domain-containing protein	1.092	0.127304119	0.18073921	0.317677835	no	up	mRNA	143.87	135.01	163.12	145.26	165.1	153.96	147.333	154.77333	COG5319	S:Function unknown	------	------
BAB_RS33395	-	uracil permease	1.092	0.127227124	0.206276331	0.350299076	no	up	mRNA	106.6	83.01	94.3	106	98	89.19	94.6367	97.73	COG2233	F:Nucleotide transport and metabolism	[illegible]	[illegible]
BAB_RS26565	-	DNA-binding response regulator	1.092	0.126495687	0.300460778	0.459704991	no	up	mRNA	17.74	15.94	21.69	18.77	19.1	19.51	18.4567	19.126667	COG4566	T:Signal transduction mechanisms	[illegible]	[illegible]
BAB_RS29855	-	monovalent cation/H+ antiporter subunit A	1.091	0.125807847	0.026230793	0.077391678	no	up	mRNA	99.8	94.31	100.66	98.06	101.22	103.33	98.2567	100.87	COG1009	P:Inorganic ion transport and metabolism	[illegible]	[illegible]
BAB_RS24395	-	ferredoxin family protein	1.091	0.125279955	0.027788431	0.080436242	no	up	mRNA	329.53	305.68	351.97	341.96	330.21	344.6	329.06	338.92333	COG1146	K:Transcription	[illegible]	[illegible]
BAB_RS23120	-	cold-shock protein	1.091	0.125382865	0.032323871	0.089919133	no	up	mRNA	1250.64	1173.94	1348.83	1287.45	1273.86	1322.14	1257.8	1294.4833	COG1278	S:Function unknown	[illegible]	[illegible]
BAB_RS20020	-	phosphoribosylformylglycinamidine synthase subunit PurL	1.091	0.12630207	0.054596385	0.132791965	no	up	mRNA	104.41	93.98	103	109.87	97.81	101.64	100.463	103.10667	COG0046	F:Nucleotide transport and metabolism	[illegible]	[illegible]
BAB_RS20470	-	Fe-S cluster assembly protein SufB	1.091	0.125441465	0.102556032	0.21001436	no	up	mRNA	183.9	179.36	191.18	210.75	183.78	175.27	184.813	189.93333	COG0719	O:Posttranslational modification, protein turnover, chaperones	GO:0016226	BP:iron-sulfur cluster assembly;
BAB_RS18105	-	DedA family protein	1.091	0.126274546	0.121529076	0.238093244	no	up	mRNA	53.26	43.6	52.03	48.42	53.64	50.77	49.63	50.943333	COG0586	S:Function unknown	GO:0016021	CC:integral component of membrane;
BAB_RS18375	-	chloride channel protein	1.09	0.124093208	0.023729005	0.072888425	no	up	mRNA	140.68	137.66	148.2	144.39	145.22	148.28	142.18	145.96333	COG0517	S:Function unknown	[illegible]	[illegible]
BAB_RS25840	-	HslU--HslV peptidase ATPase subunit	1.09	0.123845622	0.038603184	0.103016637	no	up	mRNA	203.26	182.87	192.88	198.5	201.95	194.12	193.003	198.19	COG1220	O:Posttranslational modification, protein turnover, chaperones	[illegible]	[illegible]
BAB_RS25110	-	2-oxoglutarate dehydrogenase subunit E1	1.09	0.124766052	0.046553877	0.118056518	no	up	mRNA	527.84	478.78	522.93	502.83	536.2	529.26	509.85	522.76333	COG0567	C:Energy production and conversion	[illegible]	[illegible]
BAB_RS23050	-	hypothetical protein	1.09	0.1237078	0.060689775	0.142959126	no	up	mRNA	2229.12	2222.14	2199.72	2158.51	2427.1	2433.29	2216.99	2339.6333	------	------	------	------
BAB_RS18530	-	GDP-mannose 4%2C6-dehydratase	1.09	0.124732691	0.119622805	0.235075274	no	up	mRNA	784.5	674.06	663.78	748.37	712.17	721.71	707.447	727.41667	COG1089	M:Cell wall/membrane/envelope biogenesis	[illegible]	[illegible]
BAB_RS31880	-	plasmid partitioning protein RepA	1.09	0.123866848	0.142049274	0.268473128	no	up	mRNA	141.65	121.81	131.54	148.17	135.06	121.86	131.667	135.03	COG1192	E:Amino acid transport and metabolism	[illegible]	[illegible]
BAB_RS26990	-	hypothetical protein	1.09	0.124285513	0.158270247	0.289921497	no	up	mRNA	182.59	148.9	148.97	167.1	156.23	171.55	160.153	164.96	------	------	[illegible]	[illegible]
BAB_RS27625	-	NAD synthetase	1.089	0.123042113	0.059065023	0.140992509	no	up	mRNA	149.32	136.86	143.69	137.3	154.33	149.15	143.29	146.92667	COG1282	C:Energy production and conversion	[illegible]	[illegible]
BAB_RS23150	-	thioredoxin-disulfide reductase	1.088	0.121865584	0.025360844	0.076082532	no	up	mRNA	497.62	477.26	518.29	504.99	519.6	507.33	497.723	510.64	COG0492	S:Function unknown	[illegible]	[illegible]

BAB_RS19640	-	arginyl-tRNA-protein transferase	1.088	0.121694846	0.064473421	0.149461111	no	up	mRNA	126.17	117.75	129.47	117.96	136.34	128.13	124.463	127.47667	COG2935	O:Posttranslational modification, protein turnover, chaperones	GO:0071596;GO:0071596;GO:0005737;GO:0005737;GO:0004057;GO:0008914;GO:0004057;GO:0008914	BP:ubiquitin-dependent protein catabolic process via the N-end rule pathway;BP:ubiquitin-dependent protein catabolic process via the N-end rule pathway;CC:cytoplasm;CC:cytoplasm;MF:arginyltransferase activity;MF:leucyltransferase activity;MF:arginyltransferase activity;MF:leucyltransferase activity;
BAB_RS17105	-	site-specific integrase	1.088	0.121349606	0.07653214	0.171357329	no	up	mRNA	100.95	90.88	100.43	101.17	104.88	93.32	97.42	99.79	COG0582	L:Replication, recombination and repair	GO:0006310;GO:0015074;GO:0003677	BP:DNA recombination;BP:DNA integration;MF:DNA binding;
BAB_RS28615	-	glutamine synthetase	1.088	0.122092729	0.105215519	0.21396042	no	up	mRNA	511.47	463.03	455.7	455.73	511.01	501.84	476.733	489.52667	COG0174	S:Function unknown	GO:0006542;GO:0004356	BP:glutamine biosynthetic process;MF:glutamate-ammonia ligase activity;
BAB_RS16965	-	ABC transporter permease	1.088	0.121925827	0.10983933	0.221123914	no	up	mRNA	52.12	46.09	54.83	52.9	53.17	51.17	51.0133	52.413333	COG1174	E:Amino acid transport and metabolism	GO:0055085;GO:0016021;GO:0005886	BP:transmembrane transport;CC:integral component of membrane;CC:plasma membrane;
BAB_RS17640	-	alpha/beta hydrolase	1.088	0.122261465	0.153082523	0.283000084	no	up	mRNA	57.39	45.41	55.91	56.95	54.73	50.5	52.9033	54.06	COG4782	S:Function unknown	------	------
BAB_RS19155	-	orotate phosphoribosyltransferase	1.087	0.12061072	0.068053745	0.156183344	no	up	mRNA	142.5	127.87	135.4	134.74	138.32	144.3	135.257	139.12	COG0461	F:Nucleotide transport and metabolism	GO:0044205;GO:0019856;GO:0044205;GO:0019856;GO:0000287;GO:0004588;GO:0000287;GO:0004588	BP:'de novo' UMP biosynthetic process;BP:pyrimidine nucleobase biosynthetic process;BP:'de novo' UMP biosynthetic process;BP:pyrimidine nucleobase biosynthetic process;MF:magnesium ion binding;MF:orotate phosphoribosyltransferase activity;MF:magnesium ion binding;MF:orotate phosphoribosyltransferase activity;
BAB_RS29090	-	NCS2 family permease	1.087	0.120412141	0.083307796	0.181716191	no	up	mRNA	80.69	74.47	80.27	86.11	78.59	76.19	78.4767	80.296667	COG2252	S:Function unknown	GO:0016021;GO:0022857	CC:integral component of membrane;MF:transmembrane transporter activity;
BAB_RS24550	-	hypothetical protein	1.087	0.120088013	0.096951055	0.201881879	no	up	mRNA	129.72	114.47	128.17	120.87	133.12	129.98	124.12	127.99	COG5468	S:Function unknown	GO:0043165;GO:0043165;GO:0016021;GO:0019867;GO:0016021;GO:0019867	BP:Gram-negative-bacterium-type cell outer membrane assembly;BP:Gram-negative-bacterium-type cell outer membrane assembly;CC:integral component of membrane;CC:outer membrane;CC:integral component of membrane;CC:outer membrane;
BAB_RS28840	-	5-deoxy-glucuronate isomerase	1.087	0.119833218	0.113759838	0.226322204	no	up	mRNA	63.9	55.32	61.39	57.13	62.46	65.23	60.2033	61.606667	COG3718	G:Carbohydrate transport and metabolism	GO:0019310;GO:0019310;GO:0008880;GO:0008880	BP:inositol catabolic process;BP:inositol catabolic process;MF:glucuronate isomerase activity;MF:glucuronate isomerase activity;
BAB_RS26675	-	type IV secretion system protein virB3	1.087	0.120568318	0.711602116	0.8203723	no	up	mRNA	9.95	8.23	13.22	12.13	13.51	8	10.4667	11.213333	COG3702	U:Intracellular trafficking, secretion, and vesicular transport	GO:0009405;GO:0016021;GO:0005886;GO:0016021	BP:pathogenesis;CC:integral component of membrane;CC:plasma membrane;CC:integral component of membrane;
BAB_RS21510	-	LexA repressor	1.086	0.119307308	0.033824161	0.09288635	no	up	mRNA	200.85	189.41	199.04	201	204.03	197.88	196.433	200.97	COG1974	K:Transcription	GO:0006281;GO:0006260;GO:0045892;GO:0009432;GO:0006351;GO:0006281;GO:0045892;GO:0006351;GO:0009432;GO:0006260;GO:0006377;GO:0004252;GO:0004252;GO:0003677;GO:0016874	BP:DNA repair;BP:DNA replication;BP:negative regulation of transcription, DNA-templated;BP:SOS response;BP:transcription, DNA-templated;BP:DNA repair;BP:negative regulation of transcription, DNA-templated;BP:transcription, DNA-templated;BP:SOS response;BP:DNA replication;MF:DNA binding;MF:serine-type endopeptidase activity;MF:serine-type endopeptidase activity;MF:DNA binding;MF:ligase activity;
BAB_RS20245	-	serine--tRNA ligase	1.086	0.118825818	0.054502283	0.132764089	no	up	mRNA	139.23	121.31	132.74	134.67	132.24	135.2	131.093	134.03667	COG0172	J:Translation, ribosomal structure and biogenesis	GO:0006434;GO:0016260;GO:0097056;GO:0016260;GO:0097056;GO:0006434;GO:0005737;GO:0005737;GO:0004828;GO:0005524;GO:0005524;GO:0004828	BP:seryl-tRNA aminoacylation;BP:selenocysteine biosynthetic process;BP:selenocysteinyl-tRNA;BP:selenocysteine biosynthetic process;BP:selenocysteinyl-tRN;BP:seryl-tRNA aminoacylation;CC:cytoplasm;CC:cytoplasm;MF:serine-tRNA ligase activity;MF:ATP binding;MF:ATP binding;MF:serine-tRNA ligase activity;
BAB_RS18600	-	phosphomannomutase	1.086	0.119337053	0.078693336	0.174253403	no	up	mRNA	113.83	98.32	110.63	110.15	104.4	115.54	107.593	110.03	COG1109	G:Carbohydrate transport and metabolism	GO:0005975;GO:0005975;GO:0000287;GO:0016868;GO:0016868;GO:0000287	BP:carbohydrate metabolic process;BP:carbohydrate metabolic process;MF:magnesium ion binding;MF:intramolecular transferase activity, phosphotransferases;MF:intramolecular transferase activity, phosphotransferases;MF:magnesium ion binding;
BAB_RS24505	-	MarR family transcriptional regulator	1.086	0.118375228	0.08722133	0.186952725	no	up	mRNA	182.65	162	168.9	166.3	185.32	174.53	171.183	175.38333	COG1846	E:Amino acid transport and metabolism	GO:0006351;GO:0006351;GO:0003700;GO:0003677;GO:0003677;GO:0003700	BP:transcription, DNA-templated;BP:transcription, DNA-templated;MF:transcription factor activity, sequence-specific DNA binding;MF:DNA binding;MF:DNA binding;MF:transcription factor activity, sequence-specific DNA binding;
BAB_RS20540	-	DUF2497 domain-containing protein	1.086	0.11948062	0.108935922	0.219580375	no	up	mRNA	214.21	200.09	204.02	191.14	225.4	217.92	206.107	211.48667	COG3827	S:Function unknown	------	------
BAB_RS20855	-	DUF2059 domain-containing protein	1.086	0.119190012	0.119575553	0.235126224	no	up	mRNA	582.75	486.67	483.36	550.69	510.43	540.59	517.593	533.90333	COG3184	S:Function unknown	------	------
BAB_RS29995	-	ABC transporter permease	1.086	0.119312135	0.393770006	0.557103931	no	up	mRNA	23.83	21.72	35.38	26.16	28.49	28.45	26.9767	27.7	COG0600	P:Inorganic ion transport and metabolism	GO:0055085;GO:0055085;GO:0016021;GO:0005886;GO:0016021;GO:0005886	BP:transmembrane transport;BP:transmembrane transport;CC:integral component of membrane;CC:plasma membrane;CC:integral component of membrane;CC:plasma membrane;
BAB_RS32715	-	hypothetical protein	1.086	0.119386675	0.65380618	0.774015938	no	up	mRNA	15.86	9.61	22.92	18.11	16.94	15.93	16.13	16.993333	------	------	------	------
BAB_RS21015	-	ribonucleoside-diphosphate reductase%2C adenosylcobalamin-dependent	1.085	0.118248709	0.063519021	0.148211048	no	up	mRNA	614.24	560.11	607.01	583.62	616.62	618.01	593.787	606.08333	COG0209	F:Nucleotide transport and metabolism	GO:0071897;GO:0006260;GO:0031419;GO:0050897;GO:0000166;GO:0004748	BP:DNA biosynthetic process;BP:DNA replication;MF:cobalamin binding;MF:cobalt ion binding;MF:nucleotide binding;MF:ribonucleoside-diphosphate reductase activity, thioredoxin disulfide as acceptor;
BAB_RS24450	-	pyruvate carboxylase	1.085	0.117673707	0.092577702	0.194795124	no	up	mRNA	47.78	45.61	48.81	44.04	49.88	51.21	47.4	48.376667	COG1038	C:Energy production and conversion	GO:0006090;GO:0006094;GO:0016829;GO:0016798;GO:0004736;GO:0009374;GO:0046872;GO:0005524	BP:pyruvate metabolic process;BP:gluconeogenesis;MF:lyase activity;MF:hydrolase activity, acting on glycosyl bonds;MF:pyruvate carboxylase activity;MF:biotin binding;MF:metal ion binding;MF:ATP binding;
BAB_RS18815	-	DUF2336 domain-containing protein	1.085	0.118146248	0.150631069	0.279271566	no	up	mRNA	42.75	42.4	45.5	45.4	39.83	48.44	43.55	44.556667	COG5330	S:Function unknown	------	------
BAB_RS27870	-	Lrp/AsnC family transcriptional regulator	1.085	0.118062005	0.243236372	0.394706294	no	up	mRNA	16.36	14.32	18.69	16.01	17.03	17.46	16.4567	16.833333	COG1522	M:Cell wall/membrane/envelope biogenesis	GO:0006351;GO:0005622;GO:0003700;GO:0043565	BP:transcription, DNA-templated;CC:intracellular;MF:transcription factor activity, sequence-specific DNA binding;MF:sequence-specific DNA binding;
BAB_RS20620	-	-	1.085	0.118015748	0.620292004	0.749811215	no	up	mRNA	14.13	9.5	13.6	13.65	13.86	11.03	12.41	12.846667	------	------	------	------
BAB_RS19470	-	hypothetical protein	1.084	0.116930878	0.055552516	0.134506583	no	up	mRNA	103.96	94.2	102.34	98.01	103.86	104.96	100.167	102.27667	------	------	------	------
BAB_RS31605	-	flagellar motor protein MotB	1.084	0.115933513	0.651227671	0.771816491	no	up	mRNA	1.8	1.71	1.95	1.82	1.65	2.16	1.82	1.8766667	COG1360	S:Function unknown	GO:0016020	CC:membrane;
BAB_RS17580	-	prolyl aminopeptidase	1.083	0.115491805	0.039229109	0.10254034	no	up	mRNA	288.57	263.22	282.71	277.83	284.25	289.59	278.167	283.89	COG0596	S:Function unknown	GO:0005737;GO:0005737;GO:0004177;GO:0004177;GO:0016829	CC:cytoplasm;CC:cytoplasm;MF:aminopeptidase activity;MF:aminopeptidase activity;MF:lyase activity;
BAB_RS22620	trkD	potassium transporter Kup	1.083	0.114585015	0.068260929	0.156547013	no	up	mRNA	77.72	70.25	76.06	78.2	77.08	72.87	74.6767	76.05	COG3158	P:Inorganic ion transport and metabolism	GO:0016021;GO:0005886;GO:0015079	CC:integral component of membrane;CC:plasma membrane;MF:potassium ion transmembrane transporter activity;
BAB_RS27590	-	diguanylate cyclase	1.083	0.11511808	0.092341441	0.19455282	no	up	mRNA	44.87	41.72	46.88	44.76	42.77	48.5	44.49	45.343333	COG2202;ENOG410XNMH	S:Function unknown;	GO:0016021	CC:integral component of membrane;
BAB_RS19265	-	cysteine--tRNA ligase	1.083	0.114532088	0.095689707	0.199643525	no	up	mRNA	110.14	99.01	102.33	111.34	102.76	103.41	103.827	105.83667	COG0215	J:Translation, ribosomal structure and biogenesis	GO:0006423;GO:0006423;GO:0005737;GO:0005737;GO:0004817;GO:0008270;GO:0046872;GO:0004601;GO:0005524;GO:0004817;GO:0008270;GO:0046872;GO:0005524	BP:cysteinyl-tRNA aminoacylation;BP:cysteinyl-tRNA aminoacylation;CC:cytoplasm;CC:cytoplasm;MF:cysteine-tRNA ligase activity;MF:zinc ion binding;MF:metal ion binding;MF:peroxidase activity;MF:ATP binding;MF:cysteine-tRNA ligase activity;MF:zinc ion binding;MF:metal ion binding;MF:ATP binding;
BAB_RS29520	-	quinone oxidoreductase	1.083	0.114688927	0.105067253	0.213929711	no	up	mRNA	160.96	144.19	150.02	144.54	159.72	161.8	151.723	155.35333	COG0604	S:Function unknown	GO:0008270;GO:0016491	MF:zinc ion binding;MF:oxidoreductase activity;
BAB_RS19080	-	pyridoxal phosphate-dependent aminotransferase	1.083	0.114605647	0.112793995	0.224957856	no	up	mRNA	51.16	48.79	56.94	55.95	52.29	52.06	52.2967	53.433333	COG0079	E:Amino acid transport and metabolism	GO:0009058;GO:0009058;GO:0030170;GO:0008483;GO:0008483;GO:0030170;GO:0004400	BP:biosynthetic process;BP:biosynthetic process;MF:pyridoxal phosphate binding;MF:transaminase activity;MF:transaminase activity;MF:pyridoxal phosphate binding;MF:histidinol-phosphate transaminase activity;
BAB_RS31220	-	NAD-dependent succinate-semialdehyde dehydrogenase	1.083	0.114932723	0.135254472	0.25898249	no	up	mRNA	66.71	63.49	64.79	65.46	71.97	61.44	64.9967	66.29	COG1012	L:Replication, recombination and repair	GO:0016620	MF:oxidoreductase activity, acting on the aldehyde or oxo group of donors, NAD or NADP as acceptor;
BAB_RS21760	-	alkaline phosphatase	1.083	0.114546106	0.426305174	0.589633459	no	up	mRNA	5.42	5.49	5.5	5.05	5.41	6.34	5.47	5.6	COG1785	P:Inorganic ion transport and metabolism	GO:0016791;GO:0004035	MF:phosphatase activity;MF:alkaline phosphatase activity;
BAB_RS29210	-	CoA transferase subunit B	1.083	0.115080814	0.47680167	0.639117132	no	up	mRNA	20.08	14.11	21.72	20.98	19.41	16.69	18.6367	19.026667	COG2057	I:Lipid transport and metabolism	GO:0008152;GO:0008152;GO:0008152;GO:0008410;GO:0016787;GO:0008410;GO:0008410;GO:0016787;GO:0047569	BP:metabolic process;BP:metabolic process;BP:metabolic process;MF:CoA-transferase activity;MF:hydrolase activity;MF:CoA-transferase activity;MF:CoA-transferase activity;MF:hydrolase activity;MF:3-oxoadipate CoA-transferase activity;
BAB_RS16215	-	hypothetical protein	1.083	0.114501161	0.482348125	0.642797398	no	up	mRNA	333.57	301.18	339.04	318.36	381.38	383.52	324.597	361.08667	------	------	------	------
BAB_RS32125	-	hypothetical protein	1.083	0.114949456	0.554665206	0.699976161	no	up	mRNA	29.44	15.19	31.74	23.51	28.1	25.27	25.4567	25.626667	------	------	------	------
BAB_RS24025	-	hypothetical protein	1.082	0.113314209	0.040104582	0.106054339	no	up	mRNA	169.69	167.24	184.15	172.2	181.31	177.21	173.693	176.90667	ENOG4111JUN	S:Function unknown	GO:0016021	CC:integral component of membrane;
BAB_RS18325	-	hypothetical protein	1.082	0.114273206	0.044057413	0.113517616	no	up	mRNA	304.61	281.46	309.67	309.52	313.76	291.83	298.58	305.03667	COG4233;COG4233	C:Energy production and conversion;O:Posttranslational modification, protein turnover, chaperones	GO:0005840	CC:ribosome;
BAB_RS27345	-	TldD/PmbA family protein	1.082	0.114051036	0.060013537	0.141781981	no	up	mRNA	69.44	67.08	73.59	72.41	70.3	71.62	70.0367	71.443333	COG0312	P:Inorganic ion transport and metabolism	GO:0008237	MF:metallopeptidase activity;
BAB_RS27260	-	class II fumarate hydratase	1.082	0.113577922	0.122641954	0.239543221	no	up	mRNA	128.44	130.94	127.35	121.25	135.69	137.92	128.91	131.62	COG0114	C:Energy production and conversion	GO:0006099;GO:0006106;GO:0006106;GO:0006099;GO:0045239;GO:0045239;GO:0004333;GO:0016491;GO:0004333	BP:tricarboxylic acid cycle;BP:fumarate metabolic process;BP:fumarate metabolic process;BP:tricarboxylic acid cycle;CC:tricarboxylic acid cycle enzyme complex;CC:tricarboxylic acid cycle enzyme complex;MF:fumarate hydratase activity;MF:oxidoreductase activity;MF:fumarate hydratase activity;
BAB_RS21310	-	phosphonate metabolism transcriptional regulator PhnF	1.082	0.113845662	0.136198892	0.259861663	no	up	mRNA	266.29	287.55	313.85	275.47	297.16	316.31	289.23	296.31333	COG2188	K:Transcription	GO:0006351;GO:0003700;GO:0003677	BP:transcription, DNA-templated;MF:transcription factor activity, sequence-specific DNA binding;MF:DNA binding;
BAB_RS28420	-	nickel-responsive transcriptional regulator NikR	1.082	0.113201612	0.216195697	0.36254529	no	up	mRNA	34.18	26.69	30.11	29.6	31.25	31.44	30.3267	30.763333	COG0864	K:Transcription	GO:0006355;GO:0006351;GO:0010045;GO:0010045;GO:0006351;GO:0016151;GO:0003700;GO:0003677;GO:0003677;GO:0003700;GO:0016151	BP:regulation of transcription, DNA-templated;BP:transcription, DNA-templated;BP:response to nickel cation;BP:response to nickel cation;BP:transcription, DNA-templated;MF:nickel cation binding;MF:transcription factor activity, sequence-specific DNA binding;MF:DNA binding;MF:DNA binding;MF:transcription factor activity, sequence-specific DNA binding;MF:nickel cation binding;
BAB_RS23540	-	peptide ABC transporter permease	1.082	0.113237305	0.275323682	0.430888938	no	up	mRNA	25.54	22.85	23.26	26.26	25.16	21.35	23.8833	24.256667	COG0601	P:Inorganic ion transport and metabolism	GO:0055085;GO:0055085;GO:0016021;GO:0005886;GO:0016021;GO:0005886	BP:transmembrane transport;BP:transmembrane transport;CC:integral component of membrane;CC:plasma membrane;CC:integral component of membrane;CC:plasma membrane;
BAB_RS27310	-	RNA pseudouridylate synthase	1.081	0.112076076	0.045002841	0.115306323	no	up	mRNA	172.26	158.14	177.96	177.65	172.08	166.86	169.453	172.19667	COG1187	J:Translation, ribosomal structure and biogenesis	GO:0001522;GO:0001522;GO:0009982;GO:0003723;GO:0016829;GO:0003723;GO:0009982	BP:pseudouridine synthesis;BP:pseudouridine synthesis;MF:pseudouridine synthase activity;MF:RNA binding;MF:lyase activity;MF:RNA binding;MF:pseudouridine synthase activity;
BAB_RS22535	-	DUF4198 domain-containing protein	1.081	0.112162541	0.055575383	0.134460621	no	up	mRNA	339.34	340.72	362.43	341.62	360.81	364.75	347.497	355.72667	COG5266	P:Inorganic ion transport and metabolism	GO:0016874	MF:ligase activity;
BAB_RS25385	-	diaminopimelate decarboxylase	1.081	0.1117462	0.113221984	0.225531454	no	up	mRNA	51.53	48.81	48.74	51.47	50.2	50.17	49.6933	50.613333	COG0019	M:Cell wall/membrane/envelope biogenesis	GO:0009089;GO:0009089;GO:0008836;GO:0030170;GO:0008836;GO:0030170;GO:0016787	BP:lysine biosynthetic process via diaminopimelate;BP:lysine biosynthetic process via diaminopimelate;MF:diaminopimelate decarboxylase activity;MF:pyridoxal phosphate binding;MF:diaminopimelate decarboxylase activity;MF:pyridoxal phosphate binding;MF:hydrolase activity;
BAB_RS24215	-	cell division protein ZapA	1.081	0.112351693	0.148520566	0.276956808	no	up	mRNA	303.43	275.14	309.13	297.11	327.76	299.91	295.9	308.26	COG3027	S:Function unknown	GO:0007049;GO:0051301;GO:0051301;GO:0007049;GO:0005737;GO:0005737	BP:cell cycle;BP:cell division;BP:cell division;BP:cell cycle;CC:cytoplasm;CC:cytoplasm;
BAB_RS26605	-	low specificity L-threonine aldolase	1.081	0.112443277	0.200492532	0.343197925	no	up	mRNA	37.64	36.94	41.18	43.18	35.99	39.11	38.5867	39.426667	COG2008	E:Amino acid transport and metabolism	GO:0006567;GO:0004793	BP:threonine catabolic process;MF:threonine aldolase activity;
BAB_RS16925	-	thiamine phosphate synthase	1.081	0.112354892	0.689954251	0.804946626	no	up	mRNA	9.69	3.9	8.78	7.1	8.39	6.9	7.45667	7.4633333	COG0352	H:Coenzyme transport and metabolism	GO:0009228;GO:0009229;GO:0009229;GO:0009228;GO:0046872;GO:0004789;GO:0004789;GO:0046872	BP:thiamine biosynthetic process;BP:thiamine diphosphate biosynthetic process;BP:thiamine diphosphate biosynthetic process;BP:thiamine biosynthetic process;MF:metal ion binding;MF:thiamine-phosphate diphosphorylase activity;MF:thiamine-phosphate diphosphorylase activity;MF:metal ion binding;
BAB_RS24165	-	hypothetical protein	1.08	0.110710795	0.088295438	0.188375327	no	up	mRNA	57.45	57.04	63.73	56.91	62.31	61.97	59.4067	60.396667	COG1360	N:Cell motility	GO:0016021;GO:0016021;GO:0016020	CC:integral component of membrane;CC:integral component of membrane;CC:membrane;
BAB_RS19650	-	porphobilinogen synthase	1.08	0.110374178	0.092338216	0.194673679	no	up	mRNA	139.98	122.59	146.36	144.98	137.47	133.19	136.317	138.54667	COG0113	H:Coenzyme transport and metabolism	GO:0006779;GO:0006779;GO:0004655;GO:0046872;GO:0046872;GO:0004655	BP:porphyrin-containing compound biosynthetic process;BP:porphyrin-containing compound biosynthetic process;MF:porphobilinogen synthase activity;MF:metal ion binding;MF:metal ion binding;MF:porphobilinogen synthase activity;
BAB_RS23500	-	methyltransferase	1.08	0.110826055	0.160191365	0.292108317	no	up	mRNA	56.91	58.4	69.75	65.8	64.54	57.53	61.6867	62.623333	COG5459	J:Translation, ribosomal structure and biogenesis	GO:0006412;GO:0008168	BP:translation;MF:methyltransferase activity;
BAB_RS29540	-	ABC transporter permease	1.08	0.11152336	0.338116476	0.502482997	no	up	mRNA	20.46	24.04	28.03	25.34	25.44	23.83	24.1767	24.87	COG1079	C:Energy production and conversion	GO:0008643;GO:0005886;GO:0016021;GO:0016021;GO:0005886;GO:0022857;GO:0022857	BP:carbohydrate transport;CC:plasma membrane;CC:integral component of membrane;CC:integral component of membrane;CC:plasma membrane;MF:transmembrane transporter activity;MF:transmembrane transporter activity;
BAB_RS26665	-	type IV secretion system protein virB5	1.08	0.111004579	0.440525716	0.606170932	no	up	mRNA	21.98	16.71	21.18	16.85	20.86	23.18	19.9567	20.296667	ENOG4111RX0	U:Intracellular trafficking, secretion, and vesicular transport	GO:0009405	BP:pathogenesis;
BAB_RS16470	-	NAD(P)-dependent oxidoreductase	1.079	0.109119358	0.059203721	0.141218676	no	up	mRNA	277.44	260.51	268.5	267.9	278.75	276.24	268.817	274.29667	ENOG410XQY9	S:Function unknown	GO:0016853;GO:0016491;GO:0016491	MF:isomerase activity;MF:oxidoreductase activity;MF:oxidoreductase activity;
BAB_RS18610	-	AGE family epimerase/isomerase	1.079	0.109979965	0.05968432	0.141733718	no	up	mRNA	231.62	226.04	229.96	227.91	242.01	230.54	229.207	233.48667	COG2942	G:Carbohydrate transport and metabolism	GO:0003824;GO:0003824;GO:0016853;GO:0004476	MF:catalytic activity;MF:catalytic activity;MF:isomerase activity;MF:mannose-6-phosphate isomerase activity;
BAB_RS24060	-	phosphoglucosamine mutase	1.079	0.109550453	0.070585443	0.160503205	no	up	mRNA	235.98	216.76	224.95	223.59	226.28	239.08	225.897	229.65	COG1109	E:Amino acid transport and metabolism	GO:0005975;GO:0005975;GO:0016021;GO:0000287;GO:0008966;GO:0016787;GO:0000287;GO:0008966	BP:carbohydrate metabolic process;BP:carbohydrate metabolic process;CC:integral component of membrane;MF:magnesium ion binding;MF:phosphoglucosamine mutase activity;MF:hydrolase activity;MF:magnesium ion binding;MF:phosphoglucosamine mutase activity;
BAB_RS21920	-	50S ribosomal protein L3	1.079	0.109095592	0.084105267	0.182464701	no	up	mRNA	930.74	766.07	871.38	853.28	877.33	888.93	856.063	873.18	COG0087	J:Translation, ribosomal structure and biogenesis	GO:0006412;GO:0006412;GO:0005840;GO:0005840;GO:0019843;GO:0003735;GO:0003735;GO:0019843	BP:translation;BP:translation;CC:ribosome;CC:ribosome;MF:rRNA binding;MF:structural constituent of ribosome;MF:structural constituent of ribosome;MF:rRNA binding;
BAB_RS25450	-	ABC transporter permease	1.079	0.109519319	0.0849306	0.183265291	no	up	mRNA	120.75	110.39	119.84	119.97	114.06	123.42	116.993	119.15	COG2177	D:Cell cycle control, cell division, chromosome partitioning	GO:0051301;GO:0016021	BP:cell division;CC:integral component of membrane;
BAB_RS22295	-	cobalt transporter	1.079	0.110015562	0.087192822	0.18701638	no	up	mRNA	3140.35	2909.57	3189.58	2947.63	3374.86	3208.89	3079.83	3177.1267	ENOG410XWV5;ENOG410ZZHN	S:Function unknown;P:Inorganic ion transport and metabolism	GO:0016021	CC:integral component of membrane;
BAB_RS25870	-	imidazole glycerol phosphate synthase cyclase subunit	1.079	0.110131088	0.215764032	0.362010357	no	up	mRNA	44.55	51.24	50.91	48.58	49.49	52.34	48.9	50.136667	COG0107	E:Amino acid transport and metabolism	GO:0000105;GO:0000105;GO:0000105;GO:0005737;GO:0005737;GO:0005737;GO:0000107;GO:0016829;GO:0000107;GO:0016829;GO:0016853;GO:0000107;GO:0016829	BP:histidine biosynthetic process;BP:histidine biosynthetic process;BP:histidine biosynthetic process;CC:cytoplasm;CC:cytoplasm;CC:cytoplasm;MF:imidazoleglycerol-phosphate synthase activity;MF:lyase activity;MF:imidazoleglycerol-phosphate synthase activity;MF:lyase activity;MF:isomerase activity;MF:imidazoleglycerol-phosphate synthase activity;MF:lyase activity;
BAB_RS32445	-	extensin	1.079	0.109952522	0.408698244	0.572426965	no	up	mRNA	23.3	23.59	24.41	25.45	19.71	27.96	23.7667	24.373333	COG3921	S:Function unknown	------	------
BAB_RS32385	-	hypothetical protein	1.079	0.109434018	0.744327161	0.845359904	no	up	mRNA	7.81	7.9	8.83	6.58	7.78	12.06	8.18	8.8066667	------	------	------	------
BAB_RS28110	-	ribose 5-phosphate isomerase B	1.078	0.108840992	0.083364615	0.181593564	no	up	mRNA	65.95	57.52	65.44	62.28	64.88	64.43	62.97	63.863333	COG0698	G:Carbohydrate transport and metabolism	GO:0005975;GO:0005975;GO:0005975;GO:0016853;GO:0004751;GO:0016853;GO:0004751	BP:carbohydrate metabolic process;BP:carbohydrate metabolic process;BP:carbohydrate metabolic process;MF:isomerase activity;MF:ribose-5-phosphate isomerase activity;MF:isomerase activity;MF:ribose-5-phosphate isomerase activity;
BAB_RS28475	-	acyl-CoA dehydrogenase	1.078	0.108759039	0.084426004	0.182667172	no	up	mRNA	184.82	169.54	175.93	169.22	185.57	183.83	176.763	179.54	COG1960	K:Transcription	GO:0008643;GO:0003995;GO:0016627;GO:0050660;GO:0043958;GO:0050660;GO:0016627	BP:carbohydrate transport;MF:acyl-CoA dehydrogenase activity;MF:oxidoreductase activity, acting on the CH-CH group of donors;MF:flavin adenine dinucleotide binding;MF:acryloyl-CoA reductase activity;MF:flavin adenine dinucleotide binding;MF:oxidoreductase activity, acting on the CH-CH group of donors;
BAB_RS18170	-	KR domain-containing protein	1.078	0.107828298	0.174748788	0.310031947	no	up	mRNA	86.83	74.02	76.47	85.06	77.09	78.55	79.1067	80.233333	COG1028	S:Function unknown	GO:0016491	MF:oxidoreductase activity;
BAB_RS20065	-	glyoxalase/bleomycin resistance protein/dioxygenase	1.078	0.108983459	0.193990565	0.334383951	no	up	mRNA	66.46	55.77	59.11	62.66	64.37	56.87	60.4467	61.3	COG2514	S:Function unknown	GO:0051213	MF:dioxygenase activity;
BAB_RS26765	-	DUF3380 domain-containing protein	1.078	0.107699713	0.323097918	0.485780819	no	up	mRNA	7.84	7.92	9.75	8.71	8.62	8.68	8.50333	8.67	ENOG4111MU4	S:Function unknown	------	------
BAB_RS25175	-	(deoxy)nucleoside triphosphate pyrophosphohydrolase	1.078	0.108749922	0.389670156	0.553986819	no	up	mRNA	35.49	28.96	33.16	29.22	35.79	34.69	32.5367	33.233333	COG0494	S:Function unknown	GO:0016787	MF:hydrolase activity;
BAB_RS24515	-	ATP synthase subunit beta	1.077	0.107064514	0.064599852	0.149538418	no	up	mRNA	850.06	747	847.89	813.9	837.75	826.94	814.983	826.19667	COG0055	C:Energy production and conversion	GO:0015986;GO:0015986;GO:0005886;GO:0045261;GO:0045261;GO:0005886;GO:0005524;GO:0046933;GO:0046933;GO:0016798;GO:0005524	BP:ATP synthesis coupled proton transport;BP:ATP synthesis coupled proton transport;CC:plasma membrane;CC:proton-transporting ATP synthase complex, catalytic core;CC:proton-transporting ATP synthase complex, catalytic core;CC:plasma membrane;MF:ATP binding;MF:proton-transporting ATP synthase activity, rotational mechanism;MF:proton-transporting ATP synthase activity, rotational mechanism;MF:hydrolase activity, acting on glycosyl bonds;MF:ATP binding;
BAB_RS16160	-	hypothetical protein	1.077	0.107064556	0.085647221	0.184316491	no	up	mRNA	118.97	104.38	116.09	115.8	113.01	115.94	113.147	114.91667	------	------	------	------
BAB_RS18180	-	colicin V biosynthesis protein	1.077	0.106439737	0.127564457	0.24631286	no	up	mRNA	266.24	263.45	262.01	278.35	251.32	279.68	263.9	269.78333	COG1286	S:Function unknown	GO:0009403;GO:0009403;GO:0016020;GO:0016021;GO:0016021	BP:toxin biosynthetic process;BP:toxin biosynthetic process;CC:membrane;CC:integral component of membrane;CC:integral component of membrane;
BAB_RS31000	-	2%2C3%2C4%2C5-tetrahydropyridine-2%2C6-dicarboxylate N-succinyltransferase	1.077	0.106859637	0.130359141	0.250804742	no	up	mRNA	79.52	72.96	77.69	83.02	75.29	75.44	76.7233	77.916667	COG2171	E:Amino acid transport and metabolism	GO:0009089;GO:0019877;GO:0009089;GO:0019877;GO:0019877;GO:0009089;GO:0005737;GO:0005737;GO:0005737;GO:0008666;GO:0008666;GO:0008666	BP:lysine biosynthetic process via diaminopimelate;BP:diaminopimelate biosynthetic process;BP:lysine biosynthetic process via diaminopimelate;BP:diaminopimelate biosynthetic process;BP:diaminopimelate biosynthetic process;BP:lysine biosynthetic process via diaminopimelate;CC:cytoplasm;CC:cytoplasm;CC:cytoplasm;MF:2,3,4,5-tetrahydropyridine-2,6-dicarboxylate N-succinyltransferase activity;MF:2,3,4,5-tetrahydropyridine-2,6-dicarboxylate N-succinyltransferase activity;MF:2,3,4,5-tetrahydropyridine-2,6-dicarboxylate N-succinyltransferase activity;
BAB_RS20700	-	DUF1508 domain-containing protein	1.077	0.106741042	0.158629956	0.290249458	no	up	mRNA	74.92	67.8	84.17	70.77	80.41	78.91	75.63	76.696667	COG3422	S:Function unknown	------	------
BAB_RS21480	-	triose-phosphate isomerase	1.077	0.107636251	0.177498071	0.313180286	no	up	mRNA	95.6	85.94	96.09	103.38	91.05	88.02	92.5433	94.15	COG0149	G:Carbohydrate transport and metabolism	GO:0006098;GO:0006096;GO:0006094;GO:0006094;GO:0006096;GO:0006098;GO:0006094;GO:0006096;GO:0005737;GO:0005737;GO:0005737;GO:0004807;GO:0004807;GO:0004807	BP:pentose-phosphate shunt;BP:glycolytic process;BP:gluconeogenesis;BP:gluconeogenesis;BP:glycolytic process;BP:pentose-phosphate shunt;BP:gluconeogenesis;BP:glycolytic process;CC:cytoplasm;CC:cytoplasm;CC:cytoplasm;MF:triose-phosphate isomerase activity;MF:triose-phosphate isomerase activity;MF:triose-phosphate isomerase activity;
BAB_RS26215	-	XRE family transcriptional regulator	1.076	0.106022041	0.093747322	0.196484113	no	up	mRNA	506.57	461.06	475.16	465.06	517.08	505.15	480.93	495.76333	COG1396	M:Cell wall/membrane/envelope biogenesis	GO:0043565	MF:sequence-specific DNA binding;
BAB_RS21505	-	molybdopterin molybdenumtransferase MoeA	1.076	0.105846356	0.127594977	0.246223821	no	up	mRNA	37.25	38.46	41.6	39.19	39.69	40.32	39.1033	39.733333	COG0303	H:Coenzyme transport and metabolism	GO:0032324;GO:0032324;GO:0016740	BP:molybdopterin cofactor biosynthetic process;BP:molybdopterin cofactor biosynthetic process;MF:transferase activity;
BAB_RS25760	-	transcription termination factor Rho	1.076	0.105012984	0.144536496	0.270942685	no	up	mRNA	328.81	280.42	296.34	323.94	293.03	300.15	301.857	305.70667	COG1158	K:Transcription	GO:0006353;GO:0006355;GO:0005524;GO:0004386;GO:0003723;GO:0008186	BP:DNA-templated transcription, termination;BP:regulation of transcription, DNA-templated;MF:ATP binding;MF:helicase activity;MF:RNA binding;MF:RNA-dependent ATPase activity;
BAB_RS20215	-	beta-N-acetylhexosaminidase	1.076	0.106338542	0.166888621	0.301243336	no	up	mRNA	82.21	76.64	79.18	86.67	74.2	80.78	79.3433	80.55	COG1472	G:Carbohydrate transport and metabolism	GO:0005975;GO:0102148;GO:0005975;GO:0004563;GO:0004553;GO:0004553	BP:carbohydrate metabolic process;BP:old GO;BP:carbohydrate metabolic process;MF:beta-N-acetylhexosaminidase activity;MF:hydrolase activity, hydrolyzing O-glycosyl compounds;MF:hydrolase activity, hydrolyzing O-glycosyl compounds;
BAB_RS22100	-	fructose-bisphosphatase class II	1.076	0.105089128	0.189973986	0.328694893	no	up	mRNA	34.16	30.49	33.03	35.08	30.51	33.32	32.56	32.97	COG1494	G:Carbohydrate transport and metabolism	GO:0006094;GO:0006071;GO:0006071;GO:0006094;GO:0042132;GO:0046872;GO:0042132;GO:0046872	BP:gluconeogenesis;BP:glycerol metabolic process;BP:glycerol metabolic process;BP:gluconeogenesis;MF:fructose 1,6-bisphosphate 1-phosphatase activity;MF:metal ion binding;MF:fructose 1,6-bisphosphate 1-phosphatase activity;MF:metal ion binding;
BAB_RS16830	-	3'(2')%2C5'-bisphosphate nucleotidase	1.076	0.106182618	0.425795346	0.589181933	no	up	mRNA	30.33	40.62	43.9	35.01	42.97	39.83	38.2833	39.27	COG1218	P:Inorganic ion transport and metabolism	GO:0046854;GO:0046854;GO:0016020;GO:0016020;GO:0000287;GO:0008441;GO:0000287	BP:phosphatidylinositol phosphorylation;BP:phosphatidylinositol phosphorylation;CC:membrane;CC:membrane;MF:magnesium ion binding;MF:3';MF:magnesium ion binding;
BAB_RS28595	-	iron-regulated protein FrpC	1.076	0.106019255	0.55711506	0.701140105	no	up	mRNA	4.7	5.91	5.37	5.6	5.19	5.65	5.32667	5.48	ENOG4111MZU	S:Function unknown	------	------
BAB_RS31590	-	lytic transglycosylase	1.076	0.106093443	0.790330956	0.871425313	no	up	mRNA	2.8	1.32	3.12	2.61	1.89	2.99	2.41333	2.4966667	COG0741	K:Transcription	------	------
BAB_RS18175	-	amidophosphoribosyltransferase	1.075	0.104856525	0.059899735	0.141825974	no	up	mRNA	262.4	253.94	276.84	276.65	267.22	259.47	264.393	267.78	COG0034	F:Nucleotide transport and metabolism	GO:0006189;GO:0006541;GO:0009116;GO:0009113;GO:0009113;GO:0006189;GO:0006541;GO:0009116;GO:0006189;GO:0006541;GO:0009116;GO:0009113;GO:0004044;GO:0016798;GO:0004044;GO:0004044	BP:'de novo' IMP biosynthetic process;BP:glutamine metabolic process;BP:nucleoside metabolic process;BP:purine nucleobase biosynthetic process;BP:purine nucleobase biosynthetic process;BP:'de novo' IMP biosynthetic process;BP:glutamine metabolic process;BP:nucleoside metabolic process;BP:'de novo' IMP biosynthetic process;BP:glutamine metabolic process;BP:nucleoside metabolic process;BP:purine nucleobase biosynthetic process;MF:amidophosphoribosyltransferase activity;MF:hydrolase activity, acting on glycosyl bonds;MF:amidophosphoribosyltransferase activity;MF:amidophosphoribosyltransferase activity;
BAB_RS22355	-	protein translocase subunit SecDF	1.075	0.104339084	0.098634272	0.204988302	no	up	mRNA	162.54	144.75	150.95	148.66	156.75	158.17	152.747	154.52667	COG0341	M:Cell wall/membrane/envelope biogenesis	GO:0043952;GO:0006605;GO:0065002;GO:0016021;GO:0005886;GO:0005622;GO:0015450	BP:protein transport by the Sec complex;BP:protein targeting;BP:intracellular protein transmembrane transport;CC:integral component of membrane;CC:plasma membrane;CC:intracellular;MF:P-P-bond-hydrolysis-driven protein transmembrane transporter activity;
BAB_RS26145	-	phosphate transport system regulatory protein PhoU	1.075	0.104616363	0.100009492	0.206245505	no	up	mRNA	107.18	100.76	109.24	103.11	111.44	107.83	105.727	107.46	COG0704	P:Inorganic ion transport and metabolism	GO:0030643;GO:0006817;GO:0045936;GO:0030643;GO:0045936;GO:0006817;GO:0005737;GO:0005737	BP:cellular phosphate ion homeostasis;BP:phosphate ion transport;BP:negative regulation of phosphate metabolic process;BP:cellular phosphate ion homeostasis;BP:negative regulation of phosphate metabolic process;BP:phosphate ion transport;CC:cytoplasm;CC:cytoplasm;
BAB_RS32465	-	hypothetical protein	1.075	0.103828014	0.327443264	0.490935701	no	up	mRNA	17.46	15.34	22.1	19.8	17.56	18.17	18.3	18.51	------	------	------	------
BAB_RS22175	-	precorrin-2 C(20)-methyltransferase	1.075	0.10384442	0.384517915	0.54884765	no	up	mRNA	18.36	19.14	19.89	21.18	18.92	18.53	19.13	19.543333	COG2243	H:Coenzyme transport and metabolism	GO:0009236;GO:0009236;GO:0030788;GO:0004471;GO:0008948;GO:0030788	BP:cobalamin biosynthetic process;BP:cobalamin biosynthetic process;MF:precorrin-2 C20-methyltransferase activity;MF:malate dehydrogenase;MF:oxaloacetate decarboxylase activity;MF:precorrin-2 C20-methyltransferase activity;
BAB_RS28885	-	alkyl hydroperoxide reductase AhpD	1.074	0.102372793	0.077051908	0.172041544	no	up	mRNA	1086.86	952.01	1012.15	1035.41	1007.57	1062.8	1017.01	1035.26	COG2128	S:Function unknown	GO:0006979;GO:0006979;GO:0006979;GO:0032843;GO:0008785;GO:0004601;GO:0051920;GO:0008785;GO:0032843;GO:0004601;GO:0051920;GO:0032843;GO:0051920;GO:0008785;GO:0004601	BP:response to oxidative stress;BP:response to oxidative stress;BP:response to oxidative stress;MF:hydroperoxide reductase activity;MF:alkyl hydroperoxide reductase activity;MF:peroxidase activity;MF:peroxiredoxin activity;MF:alkyl hydroperoxide reductase activity;MF:hydroperoxide reductase activity;MF:peroxidase activity;MF:peroxiredoxin activity;MF:hydroperoxide reductase activity;MF:peroxiredoxin activity;MF:alkyl hydroperoxide reductase activity;MF:peroxidase activity;
BAB_RS31215	-	GntR family transcriptional regulator	1.074	0.103107797	0.084900501	0.18332346	no	up	mRNA	103.57	97.77	101.86	100.07	102.02	104.9	101.067	102.33	ENOG4111XU0;ENOG410ZZTA	L:Replication, recombination and repair;	GO:0006351;GO:0003700;GO:0003677	BP:transcription, DNA-templated;MF:transcription factor activity, sequence-specific DNA binding;MF:DNA binding;
BAB_RS20250	-	5'-nucleotidase SurE	1.074	0.103201896	0.097081554	0.202022691	no	up	mRNA	114.79	111.06	115.58	115.08	112.96	118.74	113.81	115.59333	COG0496	F:Nucleotide transport and metabolism	GO:0005737;GO:0005737;GO:0005737;GO:0008253;GO:0046872;GO:0000166;GO:0046872;GO:0008253;GO:0000166;GO:0008253;GO:0046872;GO:0000166;GO:0016491	CC:cytoplasm;CC:cytoplasm;CC:cytoplasm;MF:5'-nucleotidase activity;MF:metal ion binding;MF:nucleotide binding;MF:metal ion binding;MF:5'-nucleotidase activity;MF:nucleotide binding;MF:5'-nucleotidase activity;MF:metal ion binding;MF:nucleotide binding;MF:oxidoreductase activity;
BAB_RS21810	-	30S ribosomal protein S13	1.074	0.102834809	0.124014231	0.24148953	no	up	mRNA	2200.69	1958.35	1987.06	2268.06	2067.59	2002.02	2048.7	2112.5567	COG0099	J:Translation, ribosomal structure and biogenesis	GO:0006412;GO:0006412;GO:0005840;GO:0005840;GO:0003735;GO:0000049;GO:0019843;GO:0019843;GO:0003735;GO:0000049	BP:translation;BP:translation;CC:ribosome;CC:ribosome;MF:structural constituent of ribosome;MF:tRNA binding;MF:rRNA binding;MF:rRNA binding;MF:structural constituent of ribosome;MF:tRNA binding;
BAB_RS24755	-	50S ribosomal protein L21	1.074	0.102712583	0.276137004	0.431741213	no	up	mRNA	1563.59	1266.83	1281.04	1551.19	1288.5	1343.67	1370.49	1394.4533	COG0261;COG3743	J:Translation, ribosomal structure and biogenesis;	GO:0006412;GO:0006412;GO:0005840;GO:0005840;GO:0019843;GO:0003735;GO:0003735;GO:0019843	BP:translation;BP:translation;CC:ribosome;CC:ribosome;MF:rRNA binding;MF:structural constituent of ribosome;MF:structural constituent of ribosome;MF:rRNA binding;
BAB_RS29135	-	ABC transporter ATP-binding protein	1.074	0.102788707	0.50673197	0.66184139	no	up	mRNA	9.03	7.46	10.73	8.6	9.79	9.25	9.07333	9.2133333	COG0410	F:Nucleotide transport and metabolism	GO:0016887;GO:0005524;GO:0005524;GO:0016887	MF:ATPase activity;MF:ATP binding;MF:ATP binding;MF:ATPase activity;
BAB_RS18780	-	hypothetical protein	1.074	0.102981247	0.683292139	0.799787848	no	up	mRNA	4.93	2.46	3.71	3.68	3.86	3.39	3.7	3.6433333	ENOG410XPU9	S:Function unknown	------	------
BAB_RS17295	-	UDP-N-acetylglucosamine 1-carboxyvinyltransferase	1.073	0.101823726	0.101029441	0.207549613	no	up	mRNA	237.87	230.62	231.5	246.26	227.88	234.78	233.33	236.30667	COG0766	M:Cell wall/membrane/envelope biogenesis	GO:0019277;GO:0071555;GO:0008360;GO:0051301;GO:0009252;GO:0007049;GO:0007049;GO:0051301;GO:0071555;GO:0009252;GO:0008360;GO:0019277;GO:0005737;GO:0005737;GO:0008760;GO:0008760	BP:UDP-N-acetylgalactosamine biosynthetic process;BP:cell wall organization;BP:regulation of cell shape;BP:cell division;BP:peptidoglycan biosynthetic process;BP:cell cycle;BP:cell cycle;BP:cell division;BP:cell wall organization;BP:peptidoglycan biosynthetic process;BP:regulation of cell shape;BP:UDP-N-acetylgalactosamine biosynthetic process;CC:cytoplasm;CC:cytoplasm;MF:UDP-N-acetylglucosamine 1-carboxyvinyltransferase activity;MF:UDP-N-acetylglucosamine 1-carboxyvinyltransferase activity;
BAB_RS23045	-	SIMPL domain-containing protein	1.073	0.101775112	0.139845716	0.265401231	no	up	mRNA	457.7	405.44	442.81	402.53	446.88	470.87	435.317	440.09333	COG2968	S:Function unknown	GO:0042597	CC:periplasmic space;
BAB_RS16355	-	DUF1223 domain-containing protein	1.073	0.102309736	0.214270648	0.360823686	no	up	mRNA	36.93	31.1	39.92	36.91	36.33	35.89	35.9833	36.376667	COG5429	S:Function unknown	------	------
BAB_RS27520	-	ABC transporter permease	1.073	0.102127644	0.729059655	0.83450968	no	up	mRNA	3.57	3.35	3.15	4.15	2.04	4.14	3.35667	3.4433333	ENOG4111FNG	G:Carbohydrate transport and metabolism	GO:0008643;GO:0016021;GO:0005886;GO:0016021;GO:0005886;GO:0022857;GO:0022857	BP:carbohydrate transport;CC:integral component of membrane;CC:plasma membrane;CC:integral component of membrane;CC:plasma membrane;MF:transmembrane transporter activity;MF:transmembrane transporter activity;
BAB_RS18970	-	DegP-like serine endoprotease	1.072	0.100408579	0.071032427	0.160950062	no	up	mRNA	202.92	194.04	220.05	198.26	210.83	213.89	205.67	207.66	COG0265	O:Posttranslational modification, protein turnover, chaperones	GO:0042597;GO:0042597;GO:0030288;GO:0004252;GO:0004252	CC:periplasmic space;CC:periplasmic space;CC:outer membrane-bounded periplasmic space;MF:serine-type endopeptidase activity;MF:serine-type endopeptidase activity;
BAB_RS29510	-	2-octaprenyl-6-methoxyphenyl hydroxylase	1.072	0.100815818	0.143082751	0.26947531	no	up	mRNA	68.98	67.63	74.96	65.81	75.11	72.91	70.5233	71.276667	COG0654	S:Function unknown	GO:0006744;GO:0006744;GO:0071949;GO:0016709;GO:0016301;GO:0071949;GO:0016709	BP:ubiquinone biosynthetic process;BP:ubiquinone biosynthetic process;MF:FAD binding;MF:oxidoreductase activity, acting on paired donors, with incorporation or reduction of molecular oxygen, NA;MF:kinase activity;MF:FAD binding;MF:oxidoreductase activity, acting on paired donors, with incorporation or reduction of molecular oxygen, NA;
BAB_RS30730	-	NAD(P)-dependent oxidoreductase	1.072	0.100674525	0.194611815	0.33527494	no	up	mRNA	61.94	52.25	59.09	57.59	59.68	58.1	57.76	58.456667	COG4221	G:Carbohydrate transport and metabolism	GO:0050255;GO:0016491;GO:0016491	MF:ribitol 2-dehydrogenase activity;MF:oxidoreductase activity;MF:oxidoreductase activity;
BAB_RS20460	-	alpha/beta hydrolase	1.072	0.100671684	0.197668945	0.339631187	no	up	mRNA	140.93	140.48	139.61	155.26	138.63	133.32	140.34	142.40333	COG2945	S:Function unknown	GO:0016787	MF:hydrolase activity;
BAB_RS26545	-	hypothetical protein	1.072	0.09979363	0.725496753	0.831023554	no	up	mRNA	10.61	5.87	7.02	9.35	7.69	6.19	7.83333	7.7433333	------	------	GO:0016021	CC:integral component of membrane;
BAB_RS22280	-	cobalamin biosynthesis protein CobW	1.071	0.098645006	0.142299258	0.268314269	no	up	mRNA	268.67	231.2	250.33	239.07	248.58	270	250.067	252.55	COG0523	S:Function unknown	GO:0009236	BP:cobalamin biosynthetic process;
BAB_RS31800	-	sulfatase	1.071	0.099045456	0.187211516	0.32549275	no	up	mRNA	37.95	33.99	34.37	35.46	36.75	34.85	35.4367	35.686667	COG3119	P:Inorganic ion transport and metabolism	GO:0008152;GO:0008152;GO:0008484;GO:0008484	BP:metabolic process;BP:metabolic process;MF:sulfuric ester hydrolase activity;MF:sulfuric ester hydrolase activity;
BAB_RS27255	-	DoxX family protein	1.071	0.099645884	0.197747254	0.33940274	no	up	mRNA	353.65	280.44	293.78	309.54	313.73	327.21	309.29	316.82667	COG2259	S:Function unknown	GO:0016021	CC:integral component of membrane;
BAB_RS17490	-	N-acetylglucosaminyltransferase	1.071	0.098636319	0.33502226	0.499038721	no	up	mRNA	7.96	7.45	9.71	8.05	9.03	8.23	8.37333	8.4366667	COG1215	M:Cell wall/membrane/envelope biogenesis	GO:0016021;GO:0016021;GO:0016740;GO:0016740;GO:0016757	CC:integral component of membrane;CC:integral component of membrane;MF:transferase activity;MF:transferase activity;MF:transferase activity, transferring glycosyl groups;
BAB_RS31375	-	ABC transporter permease	1.071	0.098995033	0.584157655	0.723274969	no	up	mRNA	7.04	4.9	7.3	6.65	6.02	6.76	6.41333	6.4766667	COG1176	S:Function unknown	GO:0055085;GO:0055085;GO:0016021;GO:0005886;GO:0016021;GO:0005886	BP:transmembrane transport;BP:transmembrane transport;CC:integral component of membrane;CC:plasma membrane;CC:integral component of membrane;CC:plasma membrane;
BAB_RS29940	-	NAD(P)/FAD-dependent oxidoreductase	1.07	0.097436008	0.083310035	0.18159779	no	up	mRNA	98.22	92.39	101.56	96.17	101.58	96.5	97.39	98.083333	COG1252	C:Energy production and conversion	GO:0016021;GO:0016021;GO:0016491;GO:0003954;GO:0004497;GO:0016491	CC:integral component of membrane;CC:integral component of membrane;MF:oxidoreductase activity;MF:NADH dehydrogenase activity;MF:monooxygenase activity;MF:oxidoreductase activity;
BAB_RS18425	-	polysaccharide deacetylase	1.07	0.098106984	0.086914566	0.186544088	no	up	mRNA	133.42	116.83	137.3	132.25	129.77	128.1	129.183	130.04	COG0726	G:Carbohydrate transport and metabolism	GO:0005975;GO:0016810	BP:carbohydrate metabolic process;MF:hydrolase activity, acting on carbon-nitrogen;
BAB_RS20240	-	twin-arginine translocase subunit TatC	1.07	0.097050062	0.122433108	0.239280764	no	up	mRNA	139.77	130.72	133.09	132.98	136.43	138.78	134.527	136.06333	COG0805	U:Intracellular trafficking, secretion, and vesicular transport	GO:0043953;GO:0043953;GO:0033281;GO:0005887;GO:0005887;GO:0033281;GO:0008320;GO:0008320	BP:protein transport by the Tat complex;BP:protein transport by the Tat complex;CC:TAT protein transport complex;CC:integral component of plasma membrane;CC:integral component of plasma membrane;CC:TAT protein transport complex;MF:protein transmembrane transporter activity;MF:protein transmembrane transporter activity;
BAB_RS16070	-	lytic murein transglycosylase	1.07	0.098083444	0.14472268	0.270975507	no	up	mRNA	65.8	64.39	64.8	64.55	68.33	63.95	64.9967	65.61	COG2951	M:Cell wall/membrane/envelope biogenesis	GO:0008152;GO:0016798	BP:metabolic process;MF:hydrolase activity, acting on glycosyl bonds;
BAB_RS30060	-	peptide deformylase	1.07	0.097140311	0.158380476	0.289958102	no	up	mRNA	87.71	79.95	82.24	82.2	86.03	84.39	83.3	84.206667	COG0242	J:Translation, ribosomal structure and biogenesis	GO:0006412;GO:0006412;GO:0046872;GO:0016805;GO:0042586;GO:0016805;GO:0046872;GO:0042586	BP:translation;BP:translation;MF:metal ion binding;MF:dipeptidase activity;MF:peptide deformylase activity;MF:dipeptidase activity;MF:metal ion binding;MF:peptide deformylase activity;
BAB_RS22170	-	precorrin-8X methylmutase	1.07	0.097924437	0.425160981	0.588811307	no	up	mRNA	24.28	24.66	26.61	23.76	24.43	29.03	25.1833	25.74	COG2082	H:Coenzyme transport and metabolism	GO:0009236;GO:0009236;GO:0016993;GO:0016993	BP:cobalamin biosynthetic process;BP:cobalamin biosynthetic process;MF:precorrin-8X methylmutase activity;MF:precorrin-8X methylmutase activity;
BAB_RS32925	-	hypothetical protein	1.07	0.097621696	0.449474783	0.614537224	no	up	mRNA	43.69	33.68	54.5	45.53	45.42	42.17	43.9567	44.373333	COG2009	C:Energy production and conversion	------	------
BAB_RS22300	-	cobyric acid synthase CobQ	1.07	0.097438712	0.463419319	0.626933167	no	up	mRNA	14.69	12.25	12.28	15.23	12.65	11.4	13.0733	13.093333	COG1492	H:Coenzyme transport and metabolism	GO:0009236;GO:0006541;GO:0009236;GO:0006541;GO:0003824;GO:0015420;GO:0015420	BP:cobalamin biosynthetic process;BP:glutamine metabolic process;BP:cobalamin biosynthetic process;BP:glutamine metabolic process;MF:catalytic activity;MF:cobalamin-transporting ATPase activity;MF:cobalamin-transporting ATPase activity;
BAB_RS26285	-	beta-ketoacyl-[acyl-carrier-protein] synthase I	1.069	0.095883265	0.100248083	0.206472493	no	up	mRNA	311.22	294.42	298.77	305.6	309.51	296.52	301.47	303.87667	COG0304	J:Translation, ribosomal structure and biogenesis	GO:0008152;GO:0008152;GO:0016740;GO:0016740;GO:0004315	BP:metabolic process;BP:metabolic process;MF:transferase activity;MF:transferase activity;MF:3-oxoacyl-[acyl-carrier-protein] synthase activity;
BAB_RS19950	-	lipoprotein-releasing system transmembrane subunit LolC	1.069	0.095686635	0.135279816	0.258876741	no	up	mRNA	124.96	122.05	119.59	121.93	122.93	124.88	122.2	123.24667	COG4591	M:Cell wall/membrane/envelope biogenesis	GO:0016021;GO:0005886;GO:0016021;GO:0005886;GO:0042954;GO:0042954	CC:integral component of membrane;CC:plasma membrane;CC:integral component of membrane;CC:plasma membrane;MF:lipoprotein transporter activity;MF:lipoprotein transporter activity;
BAB_RS23405	-	CCA tRNA nucleotidyltransferase	1.069	0.095865161	0.175883122	0.311528374	no	up	mRNA	63.5	57.19	63.66	58.77	66.89	59.71	61.45	61.79	COG0617	J:Translation, ribosomal structure and biogenesis	GO:0006396;GO:0006396;GO:0001680;GO:0006396;GO:0016779;GO:0004652;GO:0003723;GO:0005524;GO:0052927;GO:0016779;GO:0052928;GO:0052929;GO:0003723;GO:0005524;GO:0005524;GO:0016779;GO:0003723	BP:RNA processing;BP:RNA processing;BP:tRNA 3'-terminal CCA addition;BP:RNA processing;MF:nucleotidyltransferase activity;MF:polynucleotide adenylyltransferase activity;MF:RNA binding;MF:ATP binding;MF:CT;MF:nucleotidyltransferase activity;MF:CT;MF:AT;MF:RNA binding;MF:ATP binding;MF:ATP binding;MF:nucleotidyltransferase activity;MF:RNA binding;
BAB_RS26580	-	N-formylglutamate amidohydrolase	1.069	0.095751468	0.241028663	0.392909738	no	up	mRNA	142.53	130.82	163	131.37	148.15	159.8	145.45	146.44	COG3741	E:Amino acid transport and metabolism	GO:0016787;GO:0016787;GO:0050129	MF:hydrolase activity;MF:hydrolase activity;MF:N-formylglutamate deformylase activity;
BAB_RS28760	-	lectin-like protein BA14k	1.068	0.094867636	0.123863154	0.241341609	no	up	mRNA	2310.58	2212.19	2228.52	2132.13	2312.06	2462.02	2250.43	2302.07	ENOG41122WP	S:Function unknown	GO:0009405;GO:0009405;GO:0016021;GO:0016021;GO:0005886;GO:0030246	BP:pathogenesis;BP:pathogenesis;CC:integral component of membrane;CC:integral component of membrane;CC:plasma membrane;MF:carbohydrate binding;
BAB_RS19690	-	riboflavin synthase	1.068	0.095484348	0.15939719	0.290990438	no	up	mRNA	411.53	359.58	365.23	366.33	405.99	378.82	378.78	383.71333	COG0307	H:Coenzyme transport and metabolism	GO:0070207;GO:0009231;GO:1902444;GO:0004746;GO:0008870;GO:0004746	BP:protein homotrimerization;BP:riboflavin biosynthetic process;MF:riboflavin binding;MF:riboflavin synthase activity;MF:galactoside O-acetyltransferase activity;MF:riboflavin synthase activity;
BAB_RS28115	-	triose-phosphate isomerase	1.068	0.095119952	0.205360747	0.34911327	no	up	mRNA	62.11	61.8	69.5	62.22	65.74	67.96	64.47	65.306667	COG0149	G:Carbohydrate transport and metabolism	GO:0005975;GO:0006098;GO:0006096;GO:0006094;GO:0006096;GO:0006098;GO:0006094;GO:0005737;GO:0005737;GO:0004807;GO:0004807	BP:carbohydrate metabolic process;BP:pentose-phosphate shunt;BP:glycolytic process;BP:gluconeogenesis;BP:glycolytic process;BP:pentose-phosphate shunt;BP:gluconeogenesis;CC:cytoplasm;CC:cytoplasm;MF:triose-phosphate isomerase activity;MF:triose-phosphate isomerase activity;
BAB_RS22815	-	cell division protein FtsA	1.067	0.093907272	0.104755765	0.213566164	no	up	mRNA	259.82	257.54	286.25	255.51	274.43	278.63	267.87	269.52333	COG0849	D:Cell cycle control, cell division, chromosome partitioning	GO:0043093;GO:0043093;GO:0032153;GO:0009898;GO:0032153;GO:0009898	BP:FtsZ-dependent cytokinesis;BP:FtsZ-dependent cytokinesis;CC:cell division site;CC:cytoplasmic side of plasma membrane;CC:cell division site;CC:cytoplasmic side of plasma membrane;
BAB_RS23435	-	hypothetical protein	1.067	0.093512473	0.141028309	0.267329768	no	up	mRNA	42.09	43.45	44.61	43.33	44.6	42.89	43.3833	43.606667	ENOG410XPEB	S:Function unknown	GO:0016021	CC:integral component of membrane;
BAB_RS27000	-	ChbG/HpnK family deacetylase	1.067	0.093144643	0.382413573	0.547059132	no	up	mRNA	37.45	44.53	47.6	42.96	42.16	46.91	43.1933	44.01	COG3394	S:Function unknown	GO:0005975;GO:0016787;GO:0046872	BP:carbohydrate metabolic process;MF:hydrolase activity;MF:metal ion binding;
BAB_RS23070	-	DNA primase	1.066	0.091737076	0.162138157	0.295323072	no	up	mRNA	72.67	76.08	78.85	77.1	78.83	72.69	75.8667	76.206667	COG0358	L:Replication, recombination and repair	GO:1990077;GO:1990077;GO:0003677;GO:0003896;GO:0016787;GO:0008270;GO:0008270;GO:0003896;GO:0003677	CC:primosome complex;CC:primosome complex;MF:DNA binding;MF:DNA primase activity;MF:hydrolase activity;MF:zinc ion binding;MF:zinc ion binding;MF:DNA primase activity;MF:DNA binding;
BAB_RS24490	-	hydroxyproline-2-epimerase	1.066	0.092169792	0.163108485	0.296922132	no	up	mRNA	72.4	71.5	77.13	70.71	74.69	77.27	73.6767	74.223333	COG3938	E:Amino acid transport and metabolism	GO:0042116;GO:0047580;GO:0047580	BP:macrophage activation;MF:4-hydroxyproline epimerase activity;MF:4-hydroxyproline epimerase activity;
BAB_RS21475	-	preprotein translocase subunit SecG	1.066	0.092030914	0.300798991	0.459784566	no	up	mRNA	528.94	519.51	548.02	615.83	516.87	487.24	532.157	539.98	COG1314	U:Intracellular trafficking, secretion, and vesicular transport	GO:0009306;GO:0016021;GO:0015450	BP:protein secretion;CC:integral component of membrane;MF:P-P-bond-hydrolysis-driven protein transmembrane transporter activity;
BAB_RS24415	-	heme-binding protein	1.066	0.091725519	0.310037594	0.471662306	no	up	mRNA	197.57	198.64	210.18	207.83	227.09	183.07	202.13	205.99667	COG3193	S:Function unknown	------	------
BAB_RS27370	-	tetraacyldisaccharide 4'-kinase	1.066	0.091994725	0.349825433	0.514882783	no	up	mRNA	28.15	29.03	38.09	29.38	31.98	34.26	31.7567	31.873333	COG1663	M:Cell wall/membrane/envelope biogenesis	GO:0009245;GO:0009029;GO:0005524	BP:lipid A biosynthetic process;MF:tetraacyldisaccharide 4'-kinase activity;MF:ATP binding;
BAB_RS24280	-	MFS transporter	1.066	0.091738582	0.363486177	0.5298916	no	up	mRNA	57.28	65.93	72.1	64.11	65.14	70.01	65.1033	66.42	ENOG410Y0GE	S:Function unknown	------	------
BAB_RS22910	-	glycoside hydrolase	1.066	0.092808202	0.39114625	0.554612931	no	up	mRNA	17.03	14.49	15.62	15.14	15.69	16.46	15.7133	15.763333	COG3757	M:Cell wall/membrane/envelope biogenesis	GO:0016998;GO:0009253;GO:0003796	BP:cell wall macromolecule catabolic process;BP:peptidoglycan catabolic process;MF:lysozyme activity;
BAB_RS23985	-	ABC transporter ATP-binding protein	1.066	0.09169391	0.487376941	0.646015722	no	up	mRNA	11.38	9.81	12.99	12.03	12.33	9.91	11.3933	11.423333	COG1136	V:Defense mechanisms	GO:0046677;GO:0046677;GO:0016021;GO:0005886;GO:0016021;GO:0005886;GO:0015562;GO:0042895;GO:0016887;GO:0005524;GO:0042895;GO:0005524;GO:0016887;GO:0015562	BP:response to antibiotic;BP:response to antibiotic;CC:integral component of membrane;CC:plasma membrane;CC:integral component of membrane;CC:plasma membrane;MF:efflux transmembrane transporter activity;MF:antibiotic transporter activity;MF:ATPase activity;MF:ATP binding;MF:antibiotic transporter activity;MF:ATP binding;MF:ATPase activity;MF:efflux transmembrane transporter activity;
BAB_RS20365	-	DSBA oxidoreductase	1.065	0.090916926	0.141890344	0.268330592	no	up	mRNA	144.94	134.03	136.14	138.07	140.27	138.99	138.37	139.11	COG1651	E:Amino acid transport and metabolism	GO:0045454;GO:0045454;GO:0005623;GO:0005623;GO:0015035;GO:0015035	BP:cell redox homeostasis;BP:cell redox homeostasis;CC:cell;CC:cell;MF:protein disulfide oxidoreductase activity;MF:protein disulfide oxidoreductase activity;
BAB_RS16745	-	radical SAM protein	1.065	0.090423363	0.220978783	0.369025379	no	up	mRNA	48.81	50.71	53.94	54.25	49.25	50.95	51.1533	51.483333	COG0635	H:Coenzyme transport and metabolism	GO:0006782;GO:0006782;GO:0006782;GO:0005737;GO:0005737;GO:0005737;GO:0051539;GO:0004109;GO:0046872;GO:0016853;GO:0051539;GO:0004109;GO:0046872;GO:0004109;GO:0046872;GO:0051539	BP:protoporphyrinogen IX biosynthetic process;BP:protoporphyrinogen IX biosynthetic process;BP:protoporphyrinogen IX biosynthetic process;CC:cytoplasm;CC:cytoplasm;CC:cytoplasm;MF:4 iron, 4 sulfur cluster binding;MF:coproporphyrinogen oxidase activity;MF:metal ion binding;MF:isomerase activity;MF:4 iron, 4 sulfur cluster binding;MF:coproporphyrinogen oxidase activity;MF:metal ion binding;MF:coproporphyrinogen oxidase activity;MF:metal ion binding;MF:4 iron, 4 sulfur cluster binding;
BAB_RS27970	-	Lrp/AsnC family transcriptional regulator	1.065	0.091160641	0.259421463	0.413247972	no	up	mRNA	111.51	105.37	99.05	112.65	103.36	101.94	105.31	105.98333	ENOG4111X1F	K:Transcription	GO:0006351;GO:0005622;GO:0003700;GO:0043565	BP:transcription, DNA-templated;CC:intracellular;MF:transcription factor activity, sequence-specific DNA binding;MF:sequence-specific DNA binding;
BAB_RS29795	-	GntR family transcriptional regulator	1.065	0.090269379	0.397415144	0.56102586	no	up	mRNA	28.3	25.03	25.17	27.53	26.9	24.25	26.1667	26.226667	COG2188	G:Carbohydrate transport and metabolism	GO:0006351;GO:0006351;GO:0003700;GO:0003677;GO:0003677;GO:0003700	BP:transcription, DNA-templated;BP:transcription, DNA-templated;MF:transcription factor activity, sequence-specific DNA binding;MF:DNA binding;MF:DNA binding;MF:transcription factor activity, sequence-specific DNA binding;
BAB_RS25860	-	imidazole glycerol phosphate synthase subunit HisH	1.065	0.091231479	0.40459969	0.568172554	no	up	mRNA	38.3	36.63	47.96	41.2	40.24	43.44	40.9633	41.626667	COG0118	E:Amino acid transport and metabolism	GO:0000105;GO:0006541;GO:0000105;GO:0006541;GO:0006541;GO:0000105;GO:0006541;GO:0000105;GO:0005737;GO:0005737;GO:0005737;GO:0000107;GO:0016763;GO:0000107;GO:0016763	BP:histidine biosynthetic process;BP:glutamine metabolic process;BP:histidine biosynthetic process;BP:glutamine metabolic process;BP:glutamine metabolic process;BP:histidine biosynthetic process;BP:glutamine metabolic process;BP:histidine biosynthetic process;CC:cytoplasm;CC:cytoplasm;CC:cytoplasm;MF:imidazoleglycerol-phosphate synthase activity;MF:transferase activity, transferring pentosyl groups;MF:imidazoleglycerol-phosphate synthase activity;MF:transferase activity, transferring pentosyl groups;
BAB_RS17365	-	Urease accessory protein UreE 1	1.065	0.090346432	0.438376523	0.603989608	no	up	mRNA	61.2	48.93	51.81	49.02	60.86	52.91	53.98	54.263333	COG2371	O:Posttranslational modification, protein turnover, chaperones	GO:0006457;GO:0065003;GO:0018307;GO:0009405;GO:0019627;GO:0006457;GO:0065003;GO:0018307;GO:0019627;GO:0005737;GO:0005737;GO:0016151;GO:0051082;GO:0016151;GO:0051082	BP:protein folding;BP:macromolecular complex assembly;BP:enzyme active site formation;BP:pathogenesis;BP:urea metabolic process;BP:protein folding;BP:macromolecular complex assembly;BP:enzyme active site formation;BP:urea metabolic process;CC:cytoplasm;CC:cytoplasm;MF:nickel cation binding;MF:unfolded protein binding;MF:nickel cation binding;MF:unfolded protein binding;
BAB_RS26840	-	glycosyltransferase	1.065	0.090537424	0.764820815	0.858919706	no	up	mRNA	3.99	2.65	2.37	3.15	3.04	2.56	3.00333	2.9166667	COG1922	M:Cell wall/membrane/envelope biogenesis	GO:0009058;GO:0009058;GO:0009058;GO:0050087;GO:0016757;GO:0016757;GO:0016757	BP:biosynthetic process;BP:biosynthetic process;BP:biosynthetic process;MF:mannitol dehydrogenas;MF:transferase activity, transferring glycosyl groups;MF:transferase activity, transferring glycosyl groups;MF:transferase activity, transferring glycosyl groups;
BAB_RS19930	-	MBL fold metallo-hydrolase	1.064	0.089534328	0.086684035	0.186173667	no	up	mRNA	132.62	126.11	140.08	132.21	136.17	131.14	132.937	133.17333	COG0595	S:Function unknown	GO:0016787	MF:hydrolase activity;
BAB_RS29460	-	bifunctional N-acetylglucosamine-1-phosphate uridyltransferase/glucosamine-1-phosphate acetyltransferase	1.064	0.08927337	0.119046807	0.234229879	no	up	mRNA	217.13	198.14	204.57	205.96	210.07	205.46	206.613	207.16333	COG1207	M:Cell wall/membrane/envelope biogenesis	GO:0006048;GO:0000902;GO:0009245;GO:0071555;GO:0008360;GO:0009252;GO:0009103;GO:0000902;GO:0071555;GO:0009245;GO:0009103;GO:0009252;GO:0008360;GO:0006048;GO:0005737;GO:0005737;GO:0019134;GO:0000287;GO:0003977;GO:0019134;GO:0000287;GO:0003977	BP:UDP-N-acetylglucosamine biosynthetic process;BP:cell morphogenesis;BP:lipid A biosynthetic process;BP:cell wall organization;BP:regulation of cell shape;BP:peptidoglycan biosynthetic process;BP:lipopolysaccharide biosynthetic process;BP:cell morphogenesis;BP:cell wall organization;BP:lipid A biosynthetic process;BP:lipopolysaccharide biosynthetic process;BP:peptidoglycan biosynthetic process;BP:regulation of cell shape;BP:UDP-N-acetylglucosamine biosynthetic process;CC:cytoplasm;CC:cytoplasm;MF:glucosamine-1-phosphate N-acetyltransferase activity;MF:magnesium ion binding;MF:UDP-N-acetylglucosamine diphosphorylase activity;MF:glucosamine-1-phosphate N-acetyltransferase activity;MF:magnesium ion binding;MF:UDP-N-acetylglucosamine diphosphorylase activity;
BAB_RS23110	-	ABC transporter ATP-binding protein/permease	1.064	0.089894095	0.139197726	0.264483911	no	up	mRNA	104.81	96.29	102.53	95.08	103.93	105.12	101.21	101.37667	COG4178	S:Function unknown	GO:0016021;GO:0016021;GO:0042626;GO:0016740;GO:0005524;GO:0042626;GO:0005524	CC:integral component of membrane;CC:integral component of membrane;MF:ATPase activity, coupled to transmembrane movement of substances;MF:transferase activity;MF:ATP binding;MF:ATPase activity, coupled to transmembrane movement of substances;MF:ATP binding;
BAB_RS24685	-	RNA pyrophosphohydrolase	1.064	0.089114657	0.146087071	0.273211734	no	up	mRNA	124	118.89	133.09	121.25	129.23	127.7	125.327	126.06	COG0494	S:Function unknown	GO:0016787;GO:0016818;GO:0016787;GO:0016818	MF:hydrolase activity;MF:hydrolase activity, acting on acid anhydrides, in phosphorus-containing anhydrides;MF:hydrolase activity;MF:hydrolase activity, acting on acid anhydrides, in phosphorus-containing anhydrides;
BAB_RS20520	-	hypothetical protein	1.064	0.089014587	0.173326888	0.309388496	no	up	mRNA	221.81	186.72	204.34	195.15	214.11	204.47	204.29	204.57667	ENOG4111V07	S:Function unknown	------	------

BAB_RS24850	-	peptide chain release factor N(5)-glutamine methyltransferase	1.064	0.089496541	0.180864621	0.317724455	no	up	mRNA	125.5	119.49	116.62	122.05	121.65	119.97	120.537	121.22333	COG2890	J:Translation, ribosomal structure and biogenesis	GO:0018364;GO:0036009;GO:0102559;GO:0003676	BP:peptidyl-glutamine methylation;MF:protein-glutamine N-methyltransferase activity;MF:old GO;MF:nucleic acid binding;
BAB_RS29375	-	dihydroorotase	1.064	0.089039864	0.244271333	0.39618566	no	up	mRNA	49.48	47.15	52.72	54.14	49.25	46.36	49.7833	49.916667	COG0044	S:Function unknown	GO:0006221;GO:0004151;GO:0046872	BP:pyrimidine nucleotide biosynthetic process;MF:dihydroorotase activity;MF:metal ion binding;
BAB_RS26735	-	hypothetical protein	1.064	0.089573269	0.290849632	0.449494886	no	up	mRNA	66.89	67.75	75.91	63.86	73.66	75.04	70.1833	70.853333	COG0115	E:Amino acid transport and metabolism	GO:0008152;GO:0008152;GO:0016829;GO:0003824;GO:0016829;GO:0008483	BP:metabolic process;BP:metabolic process;MF:lyase activity;MF:catalytic activity;MF:lyase activity;MF:transaminase activity;
BAB_RS31935	-	hypothetical protein	1.064	0.089421916	0.321552514	0.484818502	no	up	mRNA	57.99	49.79	71.8	62.13	57.31	59.56	59.86	59.666667	------	------	------	------
BAB_RS26625	-	DUF459 domain-containing protein	1.064	0.089672029	0.381525981	0.546763147	no	up	mRNA	23.91	21.93	21.61	19.48	23.82	24.18	22.4833	22.493333	COG2845	S:Function unknown	------	------
BAB_RS24955	-	EVE domain-containing protein	1.064	0.089445833	0.389946236	0.554134125	no	up	mRNA	67.09	56.64	61.53	59.39	64.79	64.16	61.7533	62.78	COG2947	S:Function unknown	------	------
BAB_RS16635	-	DUF1049 domain-containing protein	1.064	0.089122786	0.397750988	0.56100699	no	up	mRNA	526.48	462.4	508.45	444.54	591.27	523.19	499.11	519.66667	ENOG4112876	S:Function unknown	GO:0005887	CC:integral component of plasma membrane;
BAB_RS27605	-	acetyltransferase	1.064	0.088837066	0.65071287	0.771490942	no	up	mRNA	5.73	5.86	5.81	4.9	5.33	7.39	5.8	5.8733333	ENOG4111NVD	S:Function unknown	GO:0016740	MF:transferase activity;
BAB_RS20530	-	protein-L-isoaspartate O-methyltransferase	1.063	0.088303222	0.125580909	0.243360351	no	up	mRNA	414.9	367.03	388.7	399.52	394.53	384.65	390.21	392.9	COG2518	S:Function unknown	GO:0004719	MF:protein-L-isoaspartat;
BAB_RS25115	-	succinate--CoA ligase subunit alpha	1.063	0.087715387	0.13819806	0.262895421	no	up	mRNA	1353.9	1195.03	1376.35	1243	1352.73	1342.11	1308.43	1312.6133	COG0074	C:Energy production and conversion	GO:0006099;GO:0006099;GO:0004775;GO:0048037;GO:0016853;GO:0000166;GO:0016853;GO:0016829;GO:0000166;GO:0048037;GO:0004775	BP:tricarboxylic acid cycle;BP:tricarboxylic acid cycle;MF:succinate-CoA ligas;MF:cofactor binding;MF:isomerase activity;MF:nucleotide binding;MF:isomerase activity;MF:lyase activity;MF:nucleotide binding;MF:cofactor binding;MF:succinate-CoA ligas;
BAB_RS21980	-	50S ribosomal protein L11	1.063	0.08846469	0.14935265	0.278024371	no	up	mRNA	1283.28	1153.75	1230.86	1308.85	1190.16	1198.74	1222.63	1232.5833	COG0080	J:Translation, ribosomal structure and biogenesis	GO:0006412;GO:0006412;GO:0006412;GO:0005840;GO:0005840;GO:0005840;GO:0070180;GO:0003735;GO:0003735;GO:0070180;GO:0019843;GO:0003735;GO:0070180;GO:0019843	BP:translation;BP:translation;BP:translation;CC:ribosome;CC:ribosome;CC:ribosome;MF:large ribosomal subunit rRNA binding;MF:structural constituent of ribosome;MF:structural constituent of ribosome;MF:large ribosomal subunit rRNA binding;MF:rRNA binding;MF:structural constituent of ribosome;MF:large ribosomal subunit rRNA binding;MF:rRNA binding;
BAB_RS20090	-	beta-ketoacyl-ACP synthase II	1.063	0.088355263	0.165154776	0.299290634	no	up	mRNA	82.62	81.9	90.52	87.82	81.54	86.53	85.0133	85.296667	COG0304	C:Energy production and conversion	GO:0008152;GO:0008152;GO:0016740;GO:0016740;GO:0004315	BP:metabolic process;BP:metabolic process;MF:transferase activity;MF:transferase activity;MF:3-oxoacyl-[acyl-carrier-protein] synthase activity;
BAB_RS18350	-	threonine synthase	1.063	0.088251407	0.167968154	0.302512151	no	up	mRNA	112.16	103.13	105.09	110.15	105.37	105.48	106.793	107	COG0498	E:Amino acid transport and metabolism	GO:0006520;GO:0006520;GO:0030170;GO:0004795;GO:0030170	BP:cellular amino acid metabolic process;BP:cellular amino acid metabolic process;MF:pyridoxal phosphate binding;MF:threonine synthase activity;MF:pyridoxal phosphate binding;
BAB_RS25020	-	GNAT family N-acetyltransferase	1.063	0.088373503	0.169995311	0.305820232	no	up	mRNA	1266.82	1179.79	1205.4	1124.67	1304.2	1260.49	1217.34	1229.7867	COG0454	C:Energy production and conversion	GO:0016740;GO:0016740;GO:0016746	MF:transferase activity;MF:transferase activity;MF:transferase activity, transferring acyl groups;
BAB_RS21555	-	outer membrane protein assembly factor BamA	1.063	0.087493494	0.188961117	0.32782509	no	up	mRNA	948.98	857.19	880.26	889.31	919.52	879.35	895.477	896.06	COG4775	M:Cell wall/membrane/envelope biogenesis	GO:0051205;GO:0043165;GO:0043165;GO:0051205;GO:0016021;GO:0009279;GO:0009279;GO:0016021	BP:protein insertion into membrane;BP:Gram-negative-bacterium-type cell outer membrane assembly;BP:Gram-negative-bacterium-type cell outer membrane assembly;BP:protein insertion into membrane;CC:integral component of membrane;CC:cell outer membrane;CC:cell outer membrane;CC:integral component of membrane;
BAB_RS18225	-	3-oxoacyl-[acyl-carrier-protein] reductase	1.063	0.088388119	0.208571617	0.353263365	no	up	mRNA	200.49	214.17	216.72	220.11	199.07	216.09	210.46	211.75667	ENOG410XNW1	S:Function unknown	GO:0006633;GO:0006633;GO:0051287;GO:0050089;GO:0004316;GO:0102132;GO:0102131;GO:0004316;GO:0051287	BP:fatty acid biosynthetic process;BP:fatty acid biosynthetic process;MF:NAD binding;MF:mannose isomerase activity;MF:3-oxoacyl-[acyl-carrier-protein] reductas;MF:3-oxo-pimeloyl-[acp] methyl ester reductase activity;MF:old GO;MF:3-oxoacyl-[acyl-carrier-protein] reductas;MF:NAD binding;
BAB_RS22520	-	Urease accessory protein UreG 2	1.063	0.088596213	0.215269175	0.361936086	no	up	mRNA	430.19	388.52	372.89	377.45	420.65	403.36	397.2	400.48667	COG0378	S:Function unknown	GO:0006807;GO:0006807;GO:0005737;GO:0005737;GO:0005525;GO:0003924;GO:0016151;GO:0016151;GO:0003924;GO:0005525	BP:nitrogen compound metabolic process;BP:nitrogen compound metabolic process;CC:cytoplasm;CC:cytoplasm;MF:GTP binding;MF:GTPase activity;MF:nickel cation binding;MF:nickel cation binding;MF:GTPase activity;MF:GTP binding;
BAB_RS24265	-	DUF1013 domain-containing protein	1.063	0.088689916	0.2727469	0.428526059	no	up	mRNA	556.35	454.94	464.65	517.21	468.38	495.76	491.98	493.78333	COG3820	S:Function unknown	------	------
BAB_RS28915	-	FAD-binding oxidoreductase	1.063	0.088796023	0.292700604	0.451053737	no	up	mRNA	43.07	42.21	49.93	46.7	47.85	41.12	45.07	45.223333	ENOG410XNXD	S:Function unknown	GO:0016491	MF:oxidoreductase activity;
BAB_RS16975	-	ABC transporter permease	1.063	0.087720573	0.297141089	0.455710892	no	up	mRNA	45.08	38.1	40.2	37.89	42.83	42.5	41.1267	41.073333	COG1174	E:Amino acid transport and metabolism	------	------
BAB_RS33155	-	formyltetrahydrofolate deformylase	1.063	0.087517341	0.732649038	0.836532111	no	up	mRNA	170.96	198.26	191.87	170.37	234.67	233.37	187.03	212.80333	------	------	------	------
BAB_RS24085	-	hypothetical protein	1.062	0.086505787	0.109334952	0.220246521	no	up	mRNA	906.88	828.26	897.04	913.58	863.83	884.03	877.393	887.14667	COG2885	S:Function unknown	GO:0007049;GO:0051301;GO:0009279;GO:0016021;GO:0016021;GO:0009279	BP:cell cycle;BP:cell division;CC:cell outer membrane;CC:integral component of membrane;CC:integral component of membrane;CC:cell outer membrane;
BAB_RS16410	-	DUF930 domain-containing protein	1.062	0.086910421	0.137774559	0.262711964	no	up	mRNA	151.43	146.88	161.46	160.18	149.16	151.22	153.257	153.52	ENOG4111YWI	S:Function unknown	------	------
BAB_RS22820	-	cell division protein FtsQ	1.062	0.086593647	0.139767438	0.265409443	no	up	mRNA	354.9	341.14	358.96	338.6	344.29	376.1	351.667	352.99667	COG1589	M:Cell wall/membrane/envelope biogenesis	GO:0090529;GO:0043093;GO:0090529;GO:0043093;GO:0032153;GO:0005887;GO:0032153;GO:0005887	BP:cell septum assembly;BP:FtsZ-dependent cytokinesis;BP:cell septum assembly;BP:FtsZ-dependent cytokinesis;CC:cell division site;CC:integral component of plasma membrane;CC:cell division site;CC:integral component of plasma membrane;
BAB_RS27660	-	tetratricopeptide repeat protein	1.062	0.086402317	0.19237864	0.332318586	no	up	mRNA	112.21	106.97	117.01	119.82	109.38	107.78	112.063	112.32667	COG0457	J:Translation, ribosomal structure and biogenesis	GO:0016740	MF:transferase activity;
BAB_RS16565	-	DNA-binding response regulator	1.062	0.086298306	0.240236048	0.392214645	no	up	mRNA	171.26	146.12	162.28	173.42	153.41	153.06	159.887	159.96333	COG4567	T:Signal transduction mechanisms	GO:0000160;GO:0005622;GO:0043565	BP:phosphorelay signal transduction system;CC:intracellular;MF:sequence-specific DNA binding;
BAB_RS22730	-	AsmA family protein	1.061	0.084903229	0.156152059	0.287351985	no	up	mRNA	50.45	50.73	58.24	54.79	53.41	50.67	53.14	52.956667	ENOG410XSXY	S:Function unknown	GO:0016021	CC:integral component of membrane;
BAB_RS22095	-	D-alanine--D-alanine ligase A	1.061	0.084998311	0.203448286	0.346963558	no	up	mRNA	247.68	205.6	219.06	222.34	225.25	224.23	224.113	223.94	COG1181	M:Cell wall/membrane/envelope biogenesis	GO:0071555;GO:0009252;GO:0008360;GO:0071555;GO:0008360;GO:0009252;GO:0071555;GO:0009252;GO:0008360;GO:0005737;GO:0005737;GO:0005737;GO:0005524;GO:0008716;GO:0016829;GO:0046872;GO:0008716;GO:0046872;GO:0005524;GO:0005524;GO:0008716;GO:0046872	BP:cell wall organization;BP:peptidoglycan biosynthetic process;BP:regulation of cell shape;BP:cell wall organization;BP:regulation of cell shape;BP:peptidoglycan biosynthetic process;BP:cell wall organization;BP:peptidoglycan biosynthetic process;BP:regulation of cell shape;CC:cytoplasm;CC:cytoplasm;CC:cytoplasm;MF:ATP binding;MF:D-alanine-D-alanine ligase activity;MF:lyase activity;MF:metal ion binding;MF:D-alanine-D-alanine ligase activity;MF:metal ion binding;MF:ATP binding;MF:D-alanine-D-alanine ligase activity;MF:metal ion binding;
BAB_RS22930	-	methyltransferase domain-containing protein	1.061	0.086044208	0.230164539	0.380606621	no	up	mRNA	2659.87	2593.58	2860.84	2800.2	2850.13	2476.34	2704.76	2708.89	COG0640	K:Transcription	GO:0003700;GO:0008168	MF:transcription factor activity, sequence-specific DNA binding;MF:methyltransferase activity;
BAB_RS24640	-	signal recognition particle protein	1.061	0.084779555	0.283325669	0.439134286	no	up	mRNA	43.49	41.83	45.13	48.02	41.99	40.29	43.4833	43.433333	COG0541	U:Intracellular trafficking, secretion, and vesicular transport	GO:0006614;GO:0006614;GO:0048500;GO:0048500;GO:0003924;GO:0008312;GO:0005525;GO:0008312;GO:0005525;GO:0003924	BP:SRP-dependent cotranslational protein targeting to membrane;BP:SRP-dependent cotranslational protein targeting to membrane;CC:signal recognition particle;CC:signal recognition particle;MF:GTPase activity;MF:7S RNA binding;MF:GTP binding;MF:7S RNA binding;MF:GTP binding;MF:GTPase activity;
BAB_RS30945	-	MFS transporter	1.061	0.085896708	0.455740854	0.619676413	no	up	mRNA	7.49	7.18	8.59	7.54	7.57	8.23	7.75333	7.78	ENOG410XNN3	J:Translation, ribosomal structure and biogenesis	GO:0055085;GO:0055085;GO:0016021;GO:0016021	BP:transmembrane transport;BP:transmembrane transport;CC:integral component of membrane;CC:integral component of membrane;
BAB_RS16995	-	sarcosine oxidase subunit beta family protein	1.061	0.085793088	0.53461166	0.684345524	no	up	mRNA	6	6.27	6.15	5.71	6.59	6.13	6.14	6.1433333	ENOG410XQMM	E:Amino acid transport and metabolism	GO:0046653;GO:0046653;GO:0008115;GO:0008115	BP:tetrahydrofolate metabolic process;BP:tetrahydrofolate metabolic process;MF:sarcosine oxidase activity;MF:sarcosine oxidase activity;
BAB_RS32500	-	hypothetical protein	1.061	0.085869861	0.924771214	0.954784676	no	up	mRNA	3.02	0	12.41	9.84	4.34	7.24	5.14333	7.14	------	------	------	------
BAB_RS24435	-	4-hydroxy-3-methylbut-2-en-1-yl diphosphate synthase (flavodoxin)	1.06	0.083904806	0.115483543	0.228759941	no	up	mRNA	170.04	153.33	172.81	168.15	166.21	160.66	165.393	165.00667	COG0821	I:Lipid transport and metabolism	GO:0019288;GO:0016114;GO:0016114;GO:0019288;GO:0019288;GO:0016114;GO:0051539;GO:0046429;GO:0005506;GO:0005506;GO:0046429;GO:0051539;GO:0051539;GO:0046429;GO:0016787;GO:0005506	BP:isopentenyl diphosphate biosynthetic process, methylerythritol 4-phosphate pathway;BP:terpenoid biosynthetic process;BP:terpenoid biosynthetic process;BP:isopentenyl diphosphate biosynthetic process, methylerythritol 4-phosphate pathway;BP:isopentenyl diphosphate biosynthetic process, methylerythritol 4-phosphate pathway;BP:terpenoid biosynthetic process;MF:4 iron, 4 sulfur cluster binding;MF:4-hydroxy-3-methylbut-2-en-1-yl diphosphate synthase activity;MF:iron ion binding;MF:iron ion binding;MF:4-hydroxy-3-methylbut-2-en-1-yl diphosphate synthase activity;MF:4 iron, 4 sulfur cluster binding;MF:4 iron, 4 sulfur cluster binding;MF:4-hydroxy-3-methylbut-2-en-1-yl diphosphate synthase activity;MF:hydrolase activity;MF:iron ion binding;
BAB_RS19870	-	NADH-quinone oxidoreductase subunit D	1.06	0.08352369	0.116356609	0.230205532	no	up	mRNA	434.11	401.99	439.72	437.85	415.7	421.08	425.273	424.87667	COG0649	C:Energy production and conversion	GO:0005886;GO:0005886;GO:0005886;GO:0005886;GO:0051287;GO:0048038;GO:0050136;GO:0016651;GO:0051287;GO:0050136;GO:0048038;GO:0051287;GO:0016651;GO:0048038;GO:0051287;GO:0048038;GO:0016651;GO:0050136	CC:plasma membrane;CC:plasma membrane;CC:plasma membrane;CC:plasma membrane;MF:NAD binding;MF:quinone binding;MF:NADH dehydrogenas;MF:oxidoreductase activity, acting on NA;MF:NAD binding;MF:NADH dehydrogenas;MF:quinone binding;MF:NAD binding;MF:oxidoreductase activity, acting on NA;MF:quinone binding;MF:NAD binding;MF:quinone binding;MF:oxidoreductase activity, acting on NA;MF:NADH dehydrogenas;
BAB_RS18190	-	replicative DNA helicase	1.06	0.083621311	0.141271608	0.267475355	no	up	mRNA	189.06	189.5	192.29	188.08	195.74	186.71	190.283	190.17667	COG0305	L:Replication, recombination and repair	GO:0006269;GO:0006269;GO:0006269;GO:1990077;GO:1990077;GO:1990077;GO:0003678;GO:0005524;GO:0003677;GO:0005524;GO:0003677;GO:0003678;GO:0016740;GO:0003677;GO:0003678;GO:0005524	BP:DNA replication, synthesis of RNA primer;BP:DNA replication, synthesis of RNA primer;BP:DNA replication, synthesis of RNA primer;CC:primosome complex;CC:primosome complex;CC:primosome complex;MF:DNA helicase activity;MF:ATP binding;MF:DNA binding;MF:ATP binding;MF:DNA binding;MF:DNA helicase activity;MF:transferase activity;MF:DNA binding;MF:DNA helicase activity;MF:ATP binding;
BAB_RS21050	-	threonine dehydratase	1.06	0.083868771	0.158778597	0.290190917	no	up	mRNA	84.56	75.91	82.3	80.72	80.71	80.93	80.9233	80.786667	COG1171	E:Amino acid transport and metabolism	GO:0009097;GO:0009097;GO:0004794;GO:0016740;GO:0030170;GO:0004794;GO:0030170	BP:isoleucine biosynthetic process;BP:isoleucine biosynthetic process;MF:L-threonine ammonia-lyase activity;MF:transferase activity;MF:pyridoxal phosphate binding;MF:L-threonine ammonia-lyase activity;MF:pyridoxal phosphate binding;
BAB_RS20160	-	nicotinate-nucleotide--dimethylbenzimidazole phosphoribosyltransferase	1.06	0.083829359	0.296794189	0.455613822	no	up	mRNA	120.07	134.95	124.4	122.02	133.22	124.94	126.473	126.72667	COG2038	H:Coenzyme transport and metabolism	GO:0009236;GO:0009163;GO:0009236;GO:0009163;GO:0015420;GO:0008939;GO:0015420;GO:0008939	BP:cobalamin biosynthetic process;BP:nucleoside biosynthetic process;BP:cobalamin biosynthetic process;BP:nucleoside biosynthetic process;MF:cobalamin-transporting ATPase activity;MF:nicotinate-nucleotide-dimethylbenzimidazole phosphoribosyltransferase activity;MF:cobalamin-transporting ATPase activity;MF:nicotinate-nucleotide-dimethylbenzimidazole phosphoribosyltransferase activity;
BAB_RS27730	-	hypothetical protein	1.06	0.083708751	0.329536309	0.493153312	no	up	mRNA	7.09	7.02	8.13	7.43	7.09	7.69	7.41333	7.4033333	COG2334	E:Amino acid transport and metabolism	GO:0008483;GO:0030170;GO:0030170;GO:0008483	MF:transaminase activity;MF:pyridoxal phosphate binding;MF:pyridoxal phosphate binding;MF:transaminase activity;
BAB_RS19005	-	EamA/RhaT family transporter	1.06	0.083618105	0.373677991	0.538639473	no	up	mRNA	32.56	27.22	33.21	28.52	34.39	29.6	30.9967	30.836667	COG0697;COG0697;ENOG410XQ0X	E:Amino acid transport and metabolism;G:Carbohydrate transport and metabolism;S:Function unknown	GO:0016021	CC:integral component of membrane;
BAB_RS19555	-	hypothetical protein	1.06	0.08401807	0.405822	0.568894453	no	up	mRNA	27.42	23.77	33.91	26.89	29.45	28.61	28.3667	28.316667	------	------	------	------
BAB_RS29035	-	iron ABC transporter permease	1.06	0.083762578	0.733087876	0.836141763	no	up	mRNA	6.28	14.43	10.46	10.04	11.15	10.85	10.39	10.68	COG4606	P:Inorganic ion transport and metabolism	GO:0016021;GO:0005886;GO:0005215	CC:integral component of membrane;CC:plasma membrane;MF:transporter activity;
BAB_RS19760	-	hypothetical protein	1.06	0.084516896	0.758396304	0.85439247	no	up	mRNA	198.77	117.03	117.75	120.81	149.62	185.26	144.517	151.89667			GO:0016740	MF:transferase activity;
BAB_RS26165	-	aminopeptidase	1.059	0.082278191	0.154586692	0.285288364	no	up	mRNA	282.07	268.16	318.95	277.12	289.6	298.34	289.727	288.35333	COG2309	E:Amino acid transport and metabolism	GO:0004177;GO:0004177;GO:0008237	MF:aminopeptidase activity;MF:aminopeptidase activity;MF:metallopeptidase activity;
BAB_RS27885	-	oxidoreductase	1.059	0.083028579	0.204276891	0.347638586	no	up	mRNA	89.91	87.61	92.94	82.13	93.44	94.17	90.1533	89.913333	COG0243	C:Energy production and conversion	GO:0043546;GO:0051539;GO:0030151;GO:0008863;GO:0051539;GO:0008863;GO:0030151;GO:0043546	MF:molybdopterin cofactor binding;MF:4 iron, 4 sulfur cluster binding;MF:molybdenum ion binding;MF:formate dehydrogenas;MF:4 iron, 4 sulfur cluster binding;MF:formate dehydrogenas;MF:molybdenum ion binding;MF:molybdopterin cofactor binding;
BAB_RS17690	-	invasion protein	1.059	0.082792396	0.213302919	0.359382423	no	up	mRNA	1250.53	1219.32	1266.45	1151.09	1317.24	1264.56	1245.43	1244.2967	COG5342	S:Function unknown	------	------
BAB_RS26270	-	methyltransferase	1.059	0.082516809	0.250177253	0.403929404	no	up	mRNA	38.32	35.57	38.2	38.56	37.18	36.13	37.3633	37.29	COG2813	J:Translation, ribosomal structure and biogenesis	GO:0008168	MF:methyltransferase activity;
BAB_RS22160	-	bifunctional cobalt-precorrin-7 (C(5))-methyltransferase/cobalt-precorrin-6B (C(15))-methyltransferase	1.059	0.082370198	0.482355082	0.642540165	no	up	mRNA	10.98	10.44	14.15	12.75	12.4	10.29	11.8567	11.813333	COG2242	H:Coenzyme transport and metabolism	GO:0009236;GO:0009236;GO:0009236;GO:0016021;GO:0008276;GO:0046025;GO:0047034;GO:0008276;GO:0046025;GO:0047034;GO:0008276	BP:cobalamin biosynthetic process;BP:cobalamin biosynthetic process;BP:cobalamin biosynthetic process;CC:integral component of membrane;MF:protein methyltransferase activity;MF:precorrin-6Y C5,15-methyltransferas;MF:15-hydroxyicosatetraenoate dehydrogenase activity;MF:protein methyltransferase activity;MF:precorrin-6Y C5,15-methyltransferas;MF:15-hydroxyicosatetraenoate dehydrogenase activity;MF:protein methyltransferase activity;
BAB_RS16555	-	IS5/IS1182 family transposase	1.059	0.0829721	0.49936669	0.655151153	no	up	mRNA	91.08	71.39	65.92	74.25	80.59	75.46	76.13	76.766667	COG3293	L:Replication, recombination and repair	------	------
BAB_RS31175	-	2-polyprenylphenol 6-hydroxylase	1.058	0.08154711	0.170848854	0.306497694	no	up	mRNA	85.82	86.19	91.28	88.79	89.11	84.74	87.7633	87.546667	COG0661	S:Function unknown	GO:0006744;GO:0006744;GO:0006744;GO:0016021;GO:0005886;GO:0016021;GO:0005886;GO:0005886;GO:0016021;GO:0005524;GO:0004672;GO:0005524;GO:0016787;GO:0004672;GO:0005524;GO:0004672	BP:ubiquinone biosynthetic process;BP:ubiquinone biosynthetic process;BP:ubiquinone biosynthetic process;CC:integral component of membrane;CC:plasma membrane;CC:integral component of membrane;CC:plasma membrane;CC:plasma membrane;CC:integral component of membrane;MF:ATP binding;MF:protein kinase activity;MF:ATP binding;MF:hydrolase activity;MF:protein kinase activity;MF:ATP binding;MF:protein kinase activity;
BAB_RS21495	-	indole-3-glycerol-phosphate synthase	1.058	0.081094078	0.185488503	0.323547535	no	up	mRNA	107.06	101.26	114.13	108.01	106.58	108.16	107.483	107.58333	COG0134	E:Amino acid transport and metabolism	GO:0000162;GO:0000162;GO:0004425;GO:0004425	BP:tryptophan biosynthetic process;BP:tryptophan biosynthetic process;MF:indole-3-glycerol-phosphate synthase activity;MF:indole-3-glycerol-phosphate synthase activity;
BAB_RS26050	-	phenylalanine--tRNA ligase subunit alpha	1.058	0.08165939	0.246749484	0.399599845	no	up	mRNA	55.59	51.26	59.72	57.5	56.44	52.22	55.5233	55.386667	COG0016	J:Translation, ribosomal structure and biogenesis	GO:0006432;GO:0006432;GO:0005737;GO:0005737;GO:0004826;GO:0000049;GO:0000287;GO:0046872;GO:0005524;GO:0004826;GO:0000049;GO:0000287;GO:0016740;GO:0046872;GO:0005524	BP:phenylalanyl-tRNA aminoacylation;BP:phenylalanyl-tRNA aminoacylation;CC:cytoplasm;CC:cytoplasm;MF:phenylalanine-tRNA ligase activity;MF:tRNA binding;MF:magnesium ion binding;MF:metal ion binding;MF:ATP binding;MF:phenylalanine-tRNA ligase activity;MF:tRNA binding;MF:magnesium ion binding;MF:transferase activity;MF:metal ion binding;MF:ATP binding;
BAB_RS18100	-	exodeoxyribonuclease VII small subunit	1.058	0.080662462	0.254533924	0.408296305	no	up	mRNA	190.58	166.21	168.73	173.76	183.55	168.98	175.173	175.43	COG1722	L:Replication, recombination and repair	GO:0006308;GO:0006308;GO:0005737;GO:0009318;GO:0005737;GO:0009318;GO:0008855;GO:0008855	BP:DNA catabolic process;BP:DNA catabolic process;CC:cytoplasm;CC:exodeoxyribonuclease VII complex;CC:cytoplasm;CC:exodeoxyribonuclease VII complex;MF:exodeoxyribonuclease VII activity;MF:exodeoxyribonuclease VII activity;
BAB_RS24985	-	acyl-CoA thioesterase II	1.058	0.081930338	0.367173686	0.533331398	no	up	mRNA	24.75	23.81	25.93	23.63	24.57	26.39	24.83	24.863333	COG1946	I:Lipid transport and metabolism	GO:0006637;GO:0006637;GO:0006637;GO:0047617;GO:0047617;GO:0047617;GO:0016740	BP:acyl-CoA metabolic process;BP:acyl-CoA metabolic process;BP:acyl-CoA metabolic process;MF:acyl-CoA hydrolase activity;MF:acyl-CoA hydrolase activity;MF:acyl-CoA hydrolase activity;MF:transferase activity;
BAB_RS16285	-	hypothetical protein	1.058	0.08180923	0.372935206	0.538293269	no	up	mRNA	80.91	73.9	101.79	82.31	87.24	87.04	85.5333	85.53	------	------	------	------
BAB_RS31390	-	hypothetical protein	1.058	0.08112065	0.382216463	0.547264482	no	up	mRNA	20.72	18.7	20.83	22.43	18.9	18.62	20.0833	19.983333	ENOG410XQXZ	S:Function unknown	------	------
BAB_RS22655	-	phosphoserine phosphatase SerB	1.058	0.08067694	0.666540331	0.785906085	no	up	mRNA	9.45	5.49	8.02	7.78	6.61	8.26	7.65333	7.55	COG0560	E:Amino acid transport and metabolism	GO:0004647	MF:phosphoserine phosphatase activity;
BAB_RS25055	-	NIPSNAP family protein	1.058	0.081795229	0.711644599	0.820127007	no	up	mRNA	18	25.11	23.23	23.27	23.08	23.94	22.1133	23.43	ENOG4111NIW	S:Function unknown	------	------
BAB_RS23430	-	DUF4159 domain-containing protein	1.057	0.079895549	0.153763378	0.283932031	no	up	mRNA	48.93	47.53	52.34	48.4	50.87	48.66	49.6	49.31	ENOG410XQC8	S:Function unknown	GO:0016021	CC:integral component of membrane;
BAB_RS21765	-	alanine--tRNA ligase	1.057	0.079453274	0.174203631	0.310092114	no	up	mRNA	82.81	80.59	89.85	86.24	78.82	86.62	84.4167	83.893333	COG0013	J:Translation, ribosomal structure and biogenesis	GO:0006419;GO:0006419;GO:0005737;GO:0005737;GO:0004813;GO:0000049;GO:0016740;GO:0008270;GO:0046872;GO:0005524;GO:0004813;GO:0000049;GO:0008270;GO:0046872;GO:0005524	BP:alanyl-tRNA aminoacylation;BP:alanyl-tRNA aminoacylation;CC:cytoplasm;CC:cytoplasm;MF:alanine-tRNA ligase activity;MF:tRNA binding;MF:transferase activity;MF:zinc ion binding;MF:metal ion binding;MF:ATP binding;MF:alanine-tRNA ligase activity;MF:tRNA binding;MF:zinc ion binding;MF:metal ion binding;MF:ATP binding;
BAB_RS19895	-	NADH-quinone oxidoreductase subunit I	1.057	0.080646001	0.204213537	0.347714941	no	up	mRNA	303.78	305.16	316.02	305.56	327.81	294.76	308.32	309.37667	COG1143	C:Energy production and conversion	GO:0005886;GO:0005886;GO:0005886;GO:0046872;GO:0051539;GO:0016651;GO:0005506;GO:0048038;GO:0050136;GO:0051539;GO:0046872;GO:0016651;GO:0048038;GO:0048038;GO:0051539;GO:0016651;GO:0005506;GO:0046872;GO:0050136	CC:plasma membrane;CC:plasma membrane;CC:plasma membrane;MF:metal ion binding;MF:4 iron, 4 sulfur cluster binding;MF:oxidoreductase activity, acting on NA;MF:iron ion binding;MF:quinone binding;MF:NADH dehydrogenas;MF:4 iron, 4 sulfur cluster binding;MF:metal ion binding;MF:oxidoreductase activity, acting on NA;MF:quinone binding;MF:quinone binding;MF:4 iron, 4 sulfur cluster binding;MF:oxidoreductase activity, acting on NA;MF:iron ion binding;MF:metal ion binding;MF:NADH dehydrogenas;
BAB_RS23715	-	KR domain-containing protein	1.057	0.080606746	0.233662902	0.385399848	no	up	mRNA	110.42	101.03	112.36	101.37	107.62	114.87	107.937	107.95333	COG1028;ENOG410XPR5	S:Function unknown;	GO:0008709;GO:0016491;GO:0016491	MF:cholate 7-alpha-dehydrogenase activity;MF:oxidoreductase activity;MF:oxidoreductase activity;
BAB_RS16805	-	methionine synthase	1.057	0.08057656	0.234150937	0.385610949	no	up	mRNA	1126.2	1055.73	1180.06	1170.82	1100.19	1069.76	1120.66	1113.59	COG0646	E:Amino acid transport and metabolism	GO:0042558;GO:0042558;GO:0031419;GO:0008705;GO:0008270;GO:0016491;GO:0031419;GO:0008705;GO:0008270	BP:pteridine-containing compound metabolic process;BP:pteridine-containing compound metabolic process;MF:cobalamin binding;MF:methionine synthase activity;MF:zinc ion binding;MF:oxidoreductase activity;MF:cobalamin binding;MF:methionine synthase activity;MF:zinc ion binding;
BAB_RS19730	-	3-oxoacyl-ACP synthase III	1.057	0.079768033	0.261152617	0.415593541	no	up	mRNA	145.75	135.77	145.96	154.86	138.22	133.11	142.493	142.06333	COG0332	I:Lipid transport and metabolism	GO:0006633;GO:0006633;GO:0005737;GO:0005737;GO:0004315;GO:0033818;GO:0016787;GO:0004315;GO:0033818	BP:fatty acid biosynthetic process;BP:fatty acid biosynthetic process;CC:cytoplasm;CC:cytoplasm;MF:3-oxoacyl-[acyl-carrier-protein] synthase activity;MF:beta-ketoacyl-acyl-carrier-protein synthase III activity;MF:hydrolase activity;MF:3-oxoacyl-[acyl-carrier-protein] synthase activity;MF:beta-ketoacyl-acyl-carrier-protein synthase III activity;
BAB_RS20425	-	hypothetical protein	1.057	0.080416043	0.315716611	0.478715182	no	up	mRNA	50.01	53.61	53.78	51.9	54.77	51.14	52.4667	52.603333	COG0739	L:Replication, recombination and repair	------	------
BAB_RS15970	-	ABC transporter permease	1.057	0.080098354	0.338113128	0.502710542	no	up	mRNA	66.45	65.39	58.66	62.96	64.87	62.23	63.5	63.353333	COG4239	P:Inorganic ion transport and metabolism	GO:0055085;GO:0055085;GO:0016021;GO:0005886;GO:0016021;GO:0005886	BP:transmembrane transport;BP:transmembrane transport;CC:integral component of membrane;CC:plasma membrane;CC:integral component of membrane;CC:plasma membrane;
BAB_RS19145	-	DNA-directed RNA polymerase subunit omega	1.057	0.080481469	0.379236169	0.544452999	no	up	mRNA	545.44	468.43	427.75	534.28	475.26	447.63	480.54	485.72333	COG1758	K:Transcription	GO:0006351;GO:0006351;GO:0003899;GO:0003677;GO:0003899;GO:0003677;GO:0016491	BP:transcription, DNA-templated;BP:transcription, DNA-templated;MF:DNA-directed RNA polymerase activity;MF:DNA binding;MF:DNA-directed RNA polymerase activity;MF:DNA binding;MF:oxidoreductase activity;
BAB_RS20120	-	DUF1045 domain-containing protein	1.057	0.079458629	0.478208279	0.639668277	no	up	mRNA	25.67	23.9	22.92	25.35	22.04	25.04	24.1633	24.143333	ENOG4111MI2	S:Function unknown	------	------
BAB_RS31300	-	peptide ABC transporter	1.057	0.080237363	0.668348369	0.78745996	no	up	mRNA	4.71	4.89	3.81	4.55	4.48	4.26	4.47	4.43	COG0601	S:Function unknown	GO:0055085;GO:0055085;GO:0016021;GO:0005886;GO:0016021;GO:0005886	BP:transmembrane transport;BP:transmembrane transport;CC:integral component of membrane;CC:plasma membrane;CC:integral component of membrane;CC:plasma membrane;
BAB_RS30540	-	MFS transporter	1.057	0.07960175	0.730508749	0.834978516	no	up	mRNA	1.97	1.65	2.53	1.88	2.45	1.77	2.05	2.0333333	COG0477	F:Nucleotide transport and metabolism	GO:0016021	CC:integral component of membrane;
BAB_RS33095	-	hypothetical protein	1.057	0.080561681	0.890349619	0.931215276	no	up	mRNA	21.07	7.19	4.92	11.54	6.33	12.71	11.06	10.193333	------	------	------	------
BAB_RS25565	-	cobaltochelatase subunit CobS	1.056	0.078009707	0.13588832	0.259577391	no	up	mRNA	283.18	261.33	275.38	268.9	275.83	271.74	273.297	272.15667	COG0714	H:Coenzyme transport and metabolism	GO:0051116;GO:0005524;GO:0016887;GO:0005524;GO:0016887	MF:cobaltochelatase activity;MF:ATP binding;MF:ATPase activity;MF:ATP binding;MF:ATPase activity;
BAB_RS20275	-	immunogenic membrane protein YaiC	1.056	0.079214349	0.174729292	0.310168628	no	up	mRNA	299.68	264.13	279.99	282.34	284.94	275.14	281.267	280.80667	COG1862	U:Intracellular trafficking, secretion, and vesicular transport	GO:0015031;GO:0016021;GO:0016021;GO:0005886	BP:protein transport;CC:integral component of membrane;CC:integral component of membrane;CC:plasma membrane;
BAB_RS23710	-	3-oxoacyl-ACP reductase	1.056	0.078678914	0.178063048	0.31360402	no	up	mRNA	203.7	179.04	194.14	187.19	191.78	196	192.293	191.65667	ENOG410XPR5	S:Function unknown	------	------
BAB_RS21650	-	L-serine ammonia-lyase	1.056	0.07841265	0.192461028	0.332282258	no	up	mRNA	106.03	102.87	113	107.44	111.17	101.77	107.3	106.79333	COG1760	E:Amino acid transport and metabolism	GO:0006094;GO:0003941;GO:0033794;GO:0051539	BP:gluconeogenesis;MF:L-serine ammonia-lyase activity;MF:sarcosine reductase activity;MF:4 iron, 4 sulfur cluster binding;
BAB_RS19245	-	NUDIX domain-containing protein	1.056	0.078976563	0.366673887	0.532846313	no	up	mRNA	99.8	105.34	106.22	100.22	107.57	108.54	103.787	105.44333	COG1051	F:Nucleotide transport and metabolism	GO:0016787	MF:hydrolase activity;
BAB_RS32135	-	hypothetical protein	1.056	0.077948036	0.543989758	0.691939467	no	up	mRNA	16.32	12.99	16.61	16.55	13.85	15.34	15.3067	15.246667	ENOG410ZIPJ;ENOG410XWW2	S:Function unknown;	GO:0016021	CC:integral component of membrane;
BAB_RS26745	-	oligoendopeptidase F	1.055	0.077340807	0.252388844	0.405462678	no	up	mRNA	96.7	86.03	90.91	83.73	94.09	93.83	91.2133	90.55	COG1164	E:Amino acid transport and metabolism	GO:0046872;GO:0004222;GO:0047389;GO:0046872;GO:0004222	MF:metal ion binding;MF:metalloendopeptidase activity;MF:glycerophosphocholine phosphodiesterase activity;MF:metal ion binding;MF:metalloendopeptidase activity;
BAB_RS27235	hisZ	ATP phosphoribosyltransferase regulatory subunit	1.055	0.077209987	0.373377536	0.538447946	no	up	mRNA	30.17	30.32	33.37	33.99	30.36	29.23	31.2867	31.193333	COG3705	E:Amino acid transport and metabolism	GO:0000105;GO:0000105;GO:0000105;GO:0005737;GO:0005737;GO:0005737;GO:0016757;GO:0004812	BP:histidine biosynthetic process;BP:histidine biosynthetic process;BP:histidine biosynthetic process;CC:cytoplasm;CC:cytoplasm;CC:cytoplasm;MF:transferase activity, transferring glycosyl groups;MF:aminoacyl-tRNA ligase activity;
BAB_RS23140	-	hypothetical protein	1.055	0.07767673	0.460812334	0.624458047	no	up	mRNA	20.81	19.27	26.78	21.64	24.49	20.14	22.2867	22.09	------	------	------	------
BAB_RS20420	-	IS5/IS1182 family transposase	1.055	0.077752427	0.467684686	0.631109154	no	up	mRNA	6.77	6.91	8.83	7.73	7.18	7.56	7.50333	7.49	COG3293	L:Replication, recombination and repair	GO:0006313;GO:0016021;GO:0003677;GO:0004803	BP:transposition, DNA-mediated;CC:integral component of membrane;MF:DNA binding;MF:transposase activity;
BAB_RS27610	-	aa3-type cytochrome c oxidase subunit IV	1.055	0.077529751	0.545027869	0.692437542	no	up	mRNA	971.68	967.37	826.05	862.06	1146.28	954.57	921.7	987.63667	ENOG410XUKR	S:Function unknown	GO:0016021;GO:0016021;GO:0004129	CC:integral component of membrane;CC:integral component of membrane;MF:cytochrome-c oxidase activity;
BAB_RS32850	ssrS	-	1.055	0.077776411	0.673462492	0.791164529	no	up	mRNA	117.98	117.89	158.91	132.88	150.93	140.23	131.593	141.34667	------	------	GO:0016021	CC:integral component of membrane;
BAB_RS30560	-	ABC transporter ATP-binding protein	1.055	0.077728108	0.824157056	0.893091609	no	up	mRNA	1.76	1.35	2.02	0.99	1.8	2.36	1.71	1.7166667	COG3842	G:Carbohydrate transport and metabolism	GO:0043190;GO:0043190;GO:0005524;GO:0016887;GO:0022857;GO:0022857;GO:0005524;GO:0016887	CC:ATP-binding cassett;CC:ATP-binding cassett;MF:ATP binding;MF:ATPase activity;MF:transmembrane transporter activity;MF:transmembrane transporter activity;MF:ATP binding;MF:ATPase activity;
BAB_RS21925	-	30S ribosomal protein S10	1.054	0.075383314	0.186835646	0.325191187	no	up	mRNA	1317.21	1119.92	1204.78	1216.52	1204.37	1265.55	1213.97	1228.8133	COG0051	J:Translation, ribosomal structure and biogenesis	GO:0006412;GO:0006412;GO:0005840;GO:0005840;GO:0003735;GO:0000049;GO:0003723;GO:0003735;GO:0000049	BP:translation;BP:translation;CC:ribosome;CC:ribosome;MF:structural constituent of ribosome;MF:tRNA binding;MF:RNA binding;MF:structural constituent of ribosome;MF:tRNA binding;
BAB_RS18765	-	transcriptional regulator	1.054	0.076491503	0.215041962	0.361743364	no	up	mRNA	1004.53	878.32	910.1	902.35	917.02	965.59	930.983	928.32	COG4957	K:Transcription	GO:0006355;GO:0006355;GO:0008270;GO:0003677;GO:0003677;GO:0008270	BP:regulation of transcription, DNA-templated;BP:regulation of transcription, DNA-templated;MF:zinc ion binding;MF:DNA binding;MF:DNA binding;MF:zinc ion binding;
BAB_RS18320	-	peroxiredoxin	1.054	0.076521083	0.224887142	0.373803615	no	up	mRNA	227.25	210.12	207.93	215.92	216.44	211.49	215.1	214.61667	COG0678	O:Posttranslational modification, protein turnover, chaperones	GO:0045454;GO:0045454;GO:0045454;GO:0005623;GO:0005623;GO:0005623;GO:0004601;GO:0016491;GO:0004601;GO:0016491	BP:cell redox homeostasis;BP:cell redox homeostasis;BP:cell redox homeostasis;CC:cell;CC:cell;CC:cell;MF:peroxidase activity;MF:oxidoreductase activity;MF:peroxidase activity;MF:oxidoreductase activity;
BAB_RS17575	-	hypothetical protein	1.054	0.075825949	0.235481435	0.387008619	no	up	mRNA	832.27	829.69	897.94	880.53	889.01	802.34	853.3	857.29333	ENOG41128E3;ENOG41122N6;ENOG410XX8H	S:Function unknown;	------	------
BAB_RS24145	-	sel1 repeat family protein	1.054	0.07584203	0.242657521	0.394564076	no	up	mRNA	62.32	60.39	65.8	64.82	62.71	59.94	62.8367	62.49	COG0790	S:Function unknown	------	------
BAB_RS22020	-	CBS domain-containing protein	1.054	0.076142789	0.268306807	0.423621509	no	up	mRNA	268.3	247.19	298.33	243.99	281.05	282.4	271.273	269.14667	COG0517	J:Translation, ribosomal structure and biogenesis	------	------
BAB_RS30390	-	phosphomannomutase	1.054	0.076253213	0.377040774	0.54227037	no	up	mRNA	48.77	51.51	63.03	50.6	55.8	56.12	54.4367	54.173333	COG1109	P:Inorganic ion transport and metabolism	GO:0005975;GO:0000287;GO:0004615;GO:0016868	BP:carbohydrate metabolic process;MF:magnesium ion binding;MF:phosphomannomutase activity;MF:intramolecular transferase activity, phosphotransferases;
BAB_RS16240	-	error-prone DNA polymerase	1.054	0.076017272	0.532042574	0.682688814	no	up	mRNA	3.59	4.46	4.13	3.83	4.2	4.12	4.06	4.05	COG0587	L:Replication, recombination and repair	GO:0006281;GO:0006260;GO:0006281;GO:0006260;GO:0006281;GO:0006260;GO:0005737;GO:0005737;GO:0005737;GO:0008408;GO:0003887;GO:0003676;GO:0008408;GO:0003887;GO:0003676;GO:0016491;GO:0008408;GO:0003887;GO:0003676	BP:DNA repair;BP:DNA replication;BP:DNA repair;BP:DNA replication;BP:DNA repair;BP:DNA replication;CC:cytoplasm;CC:cytoplasm;CC:cytoplasm;MF:3'-5' exonuclease activity;MF:DNA-directed DNA polymerase activity;MF:nucleic acid binding;MF:3'-5' exonuclease activity;MF:DNA-directed DNA polymerase activity;MF:nucleic acid binding;MF:oxidoreductase activity;MF:3'-5' exonuclease activity;MF:DNA-directed DNA polymerase activity;MF:nucleic acid binding;
BAB_RS24810	-	hypothetical protein	1.054	0.076409561	0.619859604	0.749570533	no	up	mRNA	31.77	30.81	48.2	40.31	37.81	33.1	36.9267	37.073333	------	------	------	------
BAB_RS19850	-	hypothetical protein	1.054	0.075275225	0.627237643	0.752263735	no	up	mRNA	50.66	41.97	46.09	55.18	41.73	43.63	46.24	46.846667	------	------	GO:0016021	CC:integral component of membrane;
BAB_RS20175	-	succinoglycan biosynthesis protein exoi	1.054	0.075455953	0.639876633	0.76314908	no	up	mRNA	17.48	12.29	14.68	16.64	12.59	14.74	14.8167	14.656667	COG1525	L:Replication, recombination and repair	GO:0016021	CC:integral component of membrane;
BAB_RS18260	-	invasion associated locus B family protein	1.053	0.075163282	0.172387638	0.308225642	no	up	mRNA	528.97	495.77	496.46	502.29	506.51	508.31	507.067	505.70333	COG5342	S:Function unknown	------	------
BAB_RS20280	-	protein translocase subunit SecDF	1.053	0.073855981	0.23556349	0.386945548	no	up	mRNA	126.58	115.17	120.69	119.68	113.42	125.97	120.813	119.69	COG0341	U:Intracellular trafficking, secretion, and vesicular transport	GO:0043952;GO:0065002;GO:0006605;GO:0006886;GO:0016021;GO:0005886;GO:0005622;GO:0016021;GO:0005622;GO:0005886;GO:0015450;GO:0015450	BP:protein transport by the Sec complex;BP:intracellular protein transmembrane transport;BP:protein targeting;BP:intracellular protein transport;CC:integral component of membrane;CC:plasma membrane;CC:intracellular;CC:integral component of membrane;CC:intracellular;CC:plasma membrane;MF:P-P-bond-hydrolysis-driven protein transmembrane transporter activity;MF:P-P-bond-hydrolysis-driven protein transmembrane transporter activity;
BAB_RS16490	-	ribonuclease D	1.053	0.075058306	0.237878077	0.389155937	no	up	mRNA	178.65	156.33	171.08	159.28	168.41	175.79	168.687	167.82667	COG0349	J:Translation, ribosomal structure and biogenesis	GO:0008408;GO:0003676;GO:0004525;GO:0008408;GO:0003676	MF:3'-5' exonuclease activity;MF:nucleic acid binding;MF:ribonuclease III activity;MF:3'-5' exonuclease activity;MF:nucleic acid binding;
BAB_RS20060	-	serine hydrolase family protein	1.053	0.074205331	0.289241877	0.447440611	no	up	mRNA	100.45	95.07	95.62	102.07	95.78	91.29	97.0467	96.38	COG3545	S:Function unknown	GO:0016787	MF:hydrolase activity;
BAB_RS25210	-	GNAT family N-acetyltransferase	1.053	0.074357919	0.31683569	0.479959016	no	up	mRNA	29.9	29.09	30.46	28.91	28.68	31.31	29.8167	29.633333	ENOG410ZPE3	S:Function unknown	------	------
BAB_RS18995	-	PAS domain-containing sensor histidine kinase	1.053	0.075076805	0.384808894	0.548288682	no	up	mRNA	41.57	39.39	41.46	35.56	40.47	45.38	40.8067	40.47	COG0642;COG0642;ENOG410XNMH;ENOG410XNMH	O:Posttranslational modification, protein turnover, chaperones;T:Signal transduction mechanisms;O:Posttranslational modification, protein turnover, chaperones;T:Signal transduction mechanisms	GO:0016021;GO:0005622;GO:0016021;GO:0005622;GO:0005524;GO:0000155;GO:0000155;GO:0005524	CC:integral component of membrane;CC:intracellular;CC:integral component of membrane;CC:intracellular;MF:ATP binding;MF:phosphorelay sensor kinase activity;MF:phosphorelay sensor kinase activity;MF:ATP binding;
BAB_RS28605	-	FAD-binding oxidoreductase	1.052	0.073232849	0.238314308	0.389472976	no	up	mRNA	99.55	95.39	103.66	100.73	103.16	91.87	99.5333	98.586667	ENOG410XWBT	C:Energy production and conversion	GO:0016491	MF:oxidoreductase activity;
BAB_RS29385	-	acyl-CoA dehydrogenase	1.052	0.073063467	0.250426488	0.403925857	no	up	mRNA	80.84	83.77	91.93	79.33	86.52	88.26	85.5133	84.703333	COG1960	F:Nucleotide transport and metabolism	GO:0003995;GO:0050660	MF:acyl-CoA dehydrogenase activity;MF:flavin adenine dinucleotide binding;
BAB_RS16055	-	30S ribosomal protein S1	1.052	0.073614426	0.251416306	0.404709715	no	up	mRNA	1254.54	1119.62	1174.4	1196.27	1160.76	1159.94	1182.85	1172.3233	COG0539	J:Translation, ribosomal structure and biogenesis	GO:0006412;GO:0006412;GO:0006412;GO:0005840;GO:0005840;GO:0005840;GO:0003735;GO:0003723;GO:0003735;GO:0003723;GO:0003735;GO:0003723;GO:0003735	BP:translation;BP:translation;BP:translation;CC:ribosome;CC:ribosome;CC:ribosome;MF:structural constituent of ribosome;MF:RNA binding;MF:RNA binding;MF:structural constituent of ribosome;MF:RNA binding;MF:structural constituent of ribosome;
BAB_RS28020	-	3-isopropylmalate dehydrogenase	1.052	0.072923672	0.255579855	0.409360955	no	up	mRNA	104.18	88.98	100.29	97.98	99.66	92.75	97.8167	96.796667	COG0473	E:Amino acid transport and metabolism	GO:0009098;GO:0009098;GO:0005737;GO:0005737;GO:0003862;GO:0000287;GO:0051287;GO:0051287;GO:0016829;GO:0000287;GO:0003862	BP:leucine biosynthetic process;BP:leucine biosynthetic process;CC:cytoplasm;CC:cytoplasm;MF:3-isopropylmalate dehydrogenase activity;MF:magnesium ion binding;MF:NAD binding;MF:NAD binding;MF:lyase activity;MF:magnesium ion binding;MF:3-isopropylmalate dehydrogenase activity;
BAB_RS23840	-	IclR family transcriptional regulator	1.052	0.072647277	0.338937124	0.503469709	no	up	mRNA	32.24	28.26	31.99	32.74	29.38	29.34	30.83	30.486667	COG1414	S:Function unknown	GO:0006355;GO:0006351;GO:0003677	BP:regulation of transcription, DNA-templated;BP:transcription, DNA-templated;MF:DNA binding;
BAB_RS23425	-	DUF58 domain-containing protein	1.052	0.072814785	0.490415676	0.648171767	no	up	mRNA	32.9	33.07	38.38	35.2	30.4	38.67	34.7833	34.756667	COG1721	S:Function unknown	------	------
BAB_RS31465	-	-	1.052	0.073564872	0.493521853	0.650137644	no	up	mRNA	307.97	307.68	277.3	250.38	334.59	302	297.65	295.65667	COG0048;ENOG4111UA7;ENOG4111VRG	J:Translation, ribosomal structure and biogenesis;S:Function unknown;S:Function unknown	------	------
BAB_RS17045	-	carbohydrate ABC transporter permease	1.052	0.072740165	0.496954097	0.653052562	no	up	mRNA	231.08	184.23	207.95	241.51	187.69	188.62	207.753	205.94	COG0395	G:Carbohydrate transport and metabolism	GO:0055085;GO:0055085;GO:0055085;GO:0008643;GO:0016021;GO:0005886;GO:0016021;GO:0005886;GO:0016021;GO:0005886	BP:transmembrane transport;BP:transmembrane transport;BP:transmembrane transport;BP:carbohydrate transport;CC:integral component of membrane;CC:plasma membrane;CC:integral component of membrane;CC:plasma membrane;CC:integral component of membrane;CC:plasma membrane;
BAB_RS25470	-	outer membrane lipoprotein carrier protein LolA	1.051	0.072222069	0.165759016	0.299878219	no	up	mRNA	394.08	356.32	384.83	371.19	371.31	383.57	378.41	375.35667	COG2834	M:Cell wall/membrane/envelope biogenesis	GO:0015031;GO:0042597	BP:protein transport;CC:periplasmic space;
BAB_RS24405	-	helicase	1.051	0.071587384	0.22049124	0.36840268	no	up	mRNA	89.16	92.23	98.01	93.51	94.53	88.3	93.1333	92.113333	COG0513	J:Translation, ribosomal structure and biogenesis	GO:0004386	MF:helicase activity;
BAB_RS21250	-	acriflavine resistance protein B	1.051	0.071545455	0.234051461	0.385644792	no	up	mRNA	243.89	221.77	235.99	219.72	243.05	230.89	233.883	231.22	COG0841	C:Energy production and conversion	GO:0016020;GO:0016021;GO:0016021;GO:0022857;GO:0022857	CC:membrane;CC:integral component of membrane;CC:integral component of membrane;MF:transmembrane transporter activity;MF:transmembrane transporter activity;
BAB_RS28640	-	glutamate--cysteine ligase	1.051	0.072255569	0.267501578	0.422765651	no	up	mRNA	64	54.11	62.15	60.48	57.42	60.25	60.0867	59.383333	COG3572	H:Coenzyme transport and metabolism	GO:0006750;GO:0006750;GO:0005524;GO:0004357;GO:0004357;GO:0016829;GO:0005524	BP:glutathione biosynthetic process;BP:glutathione biosynthetic process;MF:ATP binding;MF:glutamate-cysteine ligase activity;MF:glutamate-cysteine ligase activity;MF:lyase activity;MF:ATP binding;
BAB_RS21380	-	nitrogen regulation protein NR(I)	1.051	0.071707714	0.313736337	0.47638698	no	up	mRNA	157.08	147.02	149.82	163.2	143.64	142.82	151.307	149.88667	COG2204	T:Signal transduction mechanisms	GO:0009399;GO:0006808;GO:0006355;GO:0006351;GO:0009399;GO:0006355;GO:0006351;GO:0006808;GO:0005737;GO:0005737;GO:0005524;GO:0000156;GO:0043565;GO:0008134;GO:0043565;GO:0000156;GO:0008134;GO:0005524	BP:nitrogen fixation;BP:regulation of nitrogen utilization;BP:regulation of transcription, DNA-templated;BP:transcription, DNA-templated;BP:nitrogen fixation;BP:regulation of transcription, DNA-templated;BP:transcription, DNA-templated;BP:regulation of nitrogen utilization;CC:cytoplasm;CC:cytoplasm;MF:ATP binding;MF:phosphorelay response regulator activity;MF:sequence-specific DNA binding;MF:transcription factor binding;MF:sequence-specific DNA binding;MF:phosphorelay response regulator activity;MF:transcription factor binding;MF:ATP binding;
BAB_RS19170	-	MFS transporter	1.051	0.07211564	0.328544263	0.492127141	no	up	mRNA	49.39	40.39	50.37	49.28	44.63	44.25	46.7167	46.053333	ENOG410XPTC	G:Carbohydrate transport and metabolism	GO:0055085;GO:0055085;GO:0016021;GO:0016021	BP:transmembrane transport;BP:transmembrane transport;CC:integral component of membrane;CC:integral component of membrane;
BAB_RS23310	-	ATP-binding protein	1.051	0.071207755	0.357096598	0.522949576	no	up	mRNA	23.13	23.43	27.93	24.27	24.22	25.39	24.83	24.626667	COG0433	F:Nucleotide transport and metabolism	------	------
BAB_RS24780	-	IS6 family transposase IS2020	1.051	0.071258894	0.524323356	0.676295039	no	up	mRNA	14.81	14.61	13.82	13.76	13.24	15.91	14.4133	14.303333	COG3316	L:Replication, recombination and repair	GO:0015074	BP:DNA integration;
BAB_RS19860	-	NADH-quinone oxidoreductase subunit B	1.05	0.070826105	0.25852475	0.412228298	no	up	mRNA	417.88	413.91	431.62	445.16	411.16	405.54	421.137	420.62	COG0377	C:Energy production and conversion	GO:0005886;GO:0005886;GO:0005886;GO:0048038;GO:0051539;GO:0008137;GO:0005506;GO:0046872;GO:0051539;GO:0005506;GO:0008137;GO:0048038;GO:0051539;GO:0046872;GO:0008137;GO:0048038	CC:plasma membrane;CC:plasma membrane;CC:plasma membrane;MF:quinone binding;MF:4 iron, 4 sulfur cluster binding;MF:NADH dehydrogenas;MF:iron ion binding;MF:metal ion binding;MF:4 iron, 4 sulfur cluster binding;MF:iron ion binding;MF:NADH dehydrogenas;MF:quinone binding;MF:4 iron, 4 sulfur cluster binding;MF:metal ion binding;MF:NADH dehydrogenas;MF:quinone binding;
BAB_RS21270	-	tRNA preQ1(34) S-adenosylmethionine ribosyltransferase-isomerase QueA	1.05	0.070523441	0.267936521	0.423244858	no	up	mRNA	54.79	50.68	57.11	53.18	52.25	55.58	54.1933	53.67	COG0809	J:Translation, ribosomal structure and biogenesis	GO:0008616;GO:0008616;GO:0005737;GO:0005737;GO:0016853;GO:0016740;GO:0016740;GO:0016853	BP:queuosine biosynthetic process;BP:queuosine biosynthetic process;CC:cytoplasm;CC:cytoplasm;MF:isomerase activity;MF:transferase activity;MF:transferase activity;MF:isomerase activity;
BAB_RS20875	-	NAD+ synthase	1.05	0.070874439	0.26929202	0.424551159	no	up	mRNA	76.75	69.79	71.97	71.74	74.42	69.89	72.8367	72.016667	COG0171	H:Coenzyme transport and metabolism	GO:0009435;GO:0016491;GO:0003952;GO:0005524;GO:0008795	BP:NAD biosynthetic process;MF:oxidoreductase activity;MF:NAD+ synthas;MF:ATP binding;MF:NAD+ synthase activity;
BAB_RS16075	-	recombination protein RecR	1.05	0.070405916	0.276819579	0.432597912	no	up	mRNA	99.13	97.19	102.54	98.73	102.26	96.29	99.62	99.093333	COG0353	L:Replication, recombination and repair	GO:0006310;GO:0006281;GO:0006310;GO:0006281;GO:0003677;GO:0046872;GO:0046872;GO:0003677	BP:DNA recombination;BP:DNA repair;BP:DNA recombination;BP:DNA repair;MF:DNA binding;MF:metal ion binding;MF:metal ion binding;MF:DNA binding;
BAB_RS16210	-	FAA hydrolase family protein	1.05	0.070036132	0.349684532	0.514911274	no	up	mRNA	211.6	203.44	226.31	197.25	235.77	204.56	213.783	212.52667	COG0179	Q:Secondary metabolites biosynthesis, transport and catabolism	GO:0008152;GO:0008152;GO:0016787;GO:0047621;GO:0016787;GO:0047621;GO:0016853	BP:metabolic process;BP:metabolic process;MF:hydrolase activity;MF:acylpyruvate hydrolase activity;MF:hydrolase activity;MF:acylpyruvate hydrolase activity;MF:isomerase activity;
BAB_RS26785	-	molybdenum import ATP-binding protein ModC	1.05	0.070583178	0.368571162	0.53391305	no	up	mRNA	31.65	28.61	31.9	28.31	31.94	30.88	30.72	30.376667	COG4148	P:Inorganic ion transport and metabolism	GO:0008152;GO:0005886;GO:0005886;GO:0005886;GO:0042888;GO:0015412;GO:0005524;GO:0005524;GO:0015412;GO:0042888;GO:0042888;GO:0016798;GO:0015412;GO:0005524	BP:metabolic process;CC:plasma membrane;CC:plasma membrane;CC:plasma membrane;MF:molybdenum ion transmembrane transporter activity;MF:ATPase-coupled molybdate transmembrane transporter activity;MF:ATP binding;MF:ATP binding;MF:ATPase-coupled molybdate transmembrane transporter activity;MF:molybdenum ion transmembrane transporter activity;MF:molybdenum ion transmembrane transporter activity;MF:hydrolase activity, acting on glycosyl bonds;MF:ATPase-coupled molybdate transmembrane transporter activity;MF:ATP binding;
BAB_RS26470	-	DUF1109 domain-containing protein	1.05	0.070292901	0.404989706	0.568223548	no	up	mRNA	31.96	32.93	38.91	32.39	35.77	34.93	34.6	34.363333	COG4944	S:Function unknown	GO:0016021	CC:integral component of membrane;
BAB_RS29535	-	ABC transporter permease	1.05	0.070656858	0.426307735	0.589383284	no	up	mRNA	35.35	30.42	30.59	30.74	33.37	30.93	32.12	31.68	COG4603	C:Energy production and conversion	GO:0008643;GO:0016021;GO:0005886;GO:0016021;GO:0005886;GO:0022857;GO:0022857	BP:carbohydrate transport;CC:integral component of membrane;CC:plasma membrane;CC:integral component of membrane;CC:plasma membrane;MF:transmembrane transporter activity;MF:transmembrane transporter activity;
BAB_RS32710	-	hypothetical protein	1.05	0.070836944	0.45806999	0.621528243	no	up	mRNA	16.51	14.33	19.37	16.65	16.6	16.41	16.7367	16.553333	------	------	------	------
BAB_RS25880	-	type I pantothenate kinase	1.05	0.070518504	0.465680042	0.629196793	no	up	mRNA	23.83	17.79	22.41	20.45	21.17	21.35	21.3433	20.99	COG1072	E:Amino acid transport and metabolism	GO:0015937;GO:0015937;GO:0005737;GO:0005737;GO:0005524;GO:0004594;GO:0008168;GO:0004594;GO:0005524	BP:coenzyme A biosynthetic process;BP:coenzyme A biosynthetic process;CC:cytoplasm;CC:cytoplasm;MF:ATP binding;MF:pantothenate kinase activity;MF:methyltransferase activity;MF:pantothenate kinase activity;MF:ATP binding;
BAB_RS26905	-	HlyD family secretion protein	1.05	0.070722635	0.482445804	0.642394682	no	up	mRNA	87.59	77.52	82.91	94.43	79.7	71.31	82.6733	81.813333	COG1566	P:Inorganic ion transport and metabolism	GO:0055085;GO:0016021;GO:0005215	BP:transmembrane transport;CC:integral component of membrane;MF:transporter activity;
BAB_RS18775	-	hypothetical protein	1.05	0.070247966	0.850297098	0.909455584	no	up	mRNA	2.86	2.69	3.76	2.84	3.85	2.74	3.10333	3.1433333	COG0593	S:Function unknown	GO:0006270;GO:0006275;GO:0006270;GO:0006275;GO:0006270;GO:0006275;GO:0005737;GO:0005737;GO:0005737;GO:0005524;GO:0043565;GO:0003688;GO:0005524;GO:0043565;GO:0005524	BP:DNA replication initiation;BP:regulation of DNA replication;BP:DNA replication initiation;BP:regulation of DNA replication;BP:DNA replication initiation;BP:regulation of DNA replication;CC:cytoplasm;CC:cytoplasm;CC:cytoplasm;MF:ATP binding;MF:sequence-specific DNA binding;MF:DNA replication origin binding;MF:ATP binding;MF:sequence-specific DNA binding;MF:ATP binding;
BAB_RS16290	-	hypothetical protein	1.049	0.069578881	0.241053832	0.392751503	no	up	mRNA	1140.87	1064.19	1078.42	1067.86	1043.16	1171.83	1094.49	1094.2833	ENOG4111WBA	S:Function unknown	------	------
BAB_RS22165	-	precorrin-3B synthase	1.049	0.069446586	0.32969091	0.493154979	no	up	mRNA	59.35	56.5	61.08	62	54.05	59.1	58.9767	58.383333	COG0155	H:Coenzyme transport and metabolism	GO:0020037;GO:0046872;GO:0051539;GO:0047324;GO:0016491;GO:0051539;GO:0020037;GO:0046872;GO:0016491;GO:0046872;GO:0051539;GO:0020037;GO:0016491	MF:heme binding;MF:metal ion binding;MF:4 iron, 4 sulfur cluster binding;MF:phosphoenolpyruvate-glycerone phosphotransferase activity;MF:oxidoreductase activity;MF:4 iron, 4 sulfur cluster binding;MF:heme binding;MF:metal ion binding;MF:oxidoreductase activity;MF:metal ion binding;MF:4 iron, 4 sulfur cluster binding;MF:heme binding;MF:oxidoreductase activity;
BAB_RS21860	-	50S ribosomal protein L5	1.049	0.069379324	0.329457566	0.493265219	no	up	mRNA	674.25	557.31	655.97	671.92	598.3	609.41	629.177	626.54333	COG0094	J:Translation, ribosomal structure and biogenesis	GO:0006412;GO:0006412;GO:0005840;GO:0005840;GO:0003735;GO:0019843;GO:0000049;GO:0019843;GO:0003735;GO:0000049	BP:translation;BP:translation;CC:ribosome;CC:ribosome;MF:structural constituent of ribosome;MF:rRNA binding;MF:tRNA binding;MF:rRNA binding;MF:structural constituent of ribosome;MF:tRNA binding;
BAB_RS19100	-	porin	1.049	0.069490424	0.47670218	0.639250461	no	up	mRNA	14002.7	12510.8	12646.9	12061.35	13938.69	12783.07	13053.5	12927.703	ENOG4111F41	M:Cell wall/membrane/envelope biogenesis	GO:0006811;GO:0006811;GO:0009279;GO:0046930;GO:0009279;GO:0046930;GO:0015288;GO:0015288	BP:ion transport;BP:ion transport;CC:cell outer membrane;CC:pore complex;CC:cell outer membrane;CC:pore complex;MF:porin activity;MF:porin activity;
BAB_RS29190	-	4-hydroxybenzoate 3-monooxygenase	1.049	0.068402079	0.508461847	0.662753718	no	up	mRNA	16.95	14.31	14.75	15.55	13.34	16.43	15.3367	15.106667	COG0654	K:Transcription	GO:0043639;GO:0018659;GO:0071949	BP:benzoate catabolic process;MF:4-hydroxybenzoate 3-monooxygenase activity;MF:FAD binding;
BAB_RS24530	-	ATP synthase subunit delta	1.049	0.068745335	0.543926408	0.692132891	no	up	mRNA	218.7	166.53	173.31	211.59	170.59	171.51	186.18	184.56333	COG0712	C:Energy production and conversion	GO:0015986;GO:0015986;GO:0015986;GO:0045261;GO:0045261;GO:0005886;GO:0045261;GO:0005886;GO:0016798;GO:0046933;GO:0046933	BP:ATP synthesis coupled proton transport;BP:ATP synthesis coupled proton transport;BP:ATP synthesis coupled proton transport;CC:plasma membrane;CC:proton-transporting ATP synthase complex, catalytic core;CC:proton-transporting ATP synthase complex, catalytic core;CC:plasma membrane;CC:proton-transporting ATP synthase complex, catalytic core;CC:plasma membrane;MF:hydrolase activity, acting on glycosyl bonds;MF:proton-transporting ATP synthase activity, rotational mechanism;MF:proton-transporting ATP synthase activity, rotational mechanism;
BAB_RS19405	-	molybdopterin oxidoreductase family protein	1.048	0.06696268	0.263219255	0.418467821	no	up	mRNA	40.73	37.03	45.79	39.45	41.09	40.94	41.1833	40.493333	COG0243	C:Energy production and conversion	GO:0009055;GO:0051536;GO:0046872;GO:0043546;GO:0051536;GO:0046872;GO:0043546;GO:0009055	MF:electron carrier activity;MF:iron-sulfur cluster binding;MF:metal ion binding;MF:molybdopterin cofactor binding;MF:iron-sulfur cluster binding;MF:metal ion binding;MF:molybdopterin cofactor binding;MF:electron carrier activity;
BAB_RS29570	-	transcriptional regulator	1.048	0.068121864	0.321746984	0.484429737	no	up	mRNA	624.45	599.98	558.77	584.23	635.38	563.73	594.4	594.44667	COG1959	K:Transcription	GO:0003677	MF:DNA binding;
BAB_RS22210	-	ABC transporter substrate-binding protein	1.048	0.067419548	0.480201195	0.641266185	no	up	mRNA	13.38	13.23	15.86	14.33	13.38	14.38	14.1567	14.03	COG0683	S:Function unknown	------	------
BAB_RS20510	-	ATP-dependent helicase	1.048	0.067059357	0.487823894	0.646341514	no	up	mRNA	446.27	362.3	385.81	446.13	373.35	358.4	398.127	392.62667	COG0513	G:Carbohydrate transport and metabolism	GO:0005524;GO:0003676;GO:0004386;GO:0005524;GO:0004386;GO:0003676;GO:0003676;GO:0005524;GO:0004386	MF:ATP binding;MF:nucleic acid binding;MF:helicase activity;MF:ATP binding;MF:helicase activity;MF:nucleic acid binding;MF:nucleic acid binding;MF:ATP binding;MF:helicase activity;
BAB_RS27420	-	MerR family DNA-binding transcriptional regulator	1.048	0.066962367	0.524409933	0.676135278	no	up	mRNA	46.64	45.3	40.41	45.98	45.78	38.94	44.1167	43.566667	COG0789	C:Energy production and conversion	GO:0006355;GO:0003677	BP:regulation of transcription, DNA-templated;MF:DNA binding;
BAB_RS25320	-	electron transfer flavoprotein subunit alpha/FixB family protein	1.047	0.066707936	0.242479642	0.394474475	no	up	mRNA	173.87	157.86	178.15	158.9	171.74	172.79	169.96	167.81	COG2025	C:Energy production and conversion	GO:0009055;GO:0050660;GO:0050660;GO:0009055	MF:electron carrier activity;MF:flavin adenine dinucleotide binding;MF:flavin adenine dinucleotide binding;MF:electron carrier activity;
BAB_RS26235	-	transcription termination/antitermination protein NusA	1.047	0.065756298	0.281494156	0.437773826	no	up	mRNA	489.97	426.67	471.72	480.02	442.95	444.82	462.787	455.93	COG0195	K:Transcription	GO:0006353;GO:0042274;GO:0031564;GO:0006353;GO:0031564;GO:0031564;GO:0006353;GO:0005737;GO:0005737;GO:0005737;GO:0005840;GO:0003700;GO:0000166;GO:0003723;GO:0003700;GO:0000166;GO:0003723;GO:0003700;GO:0000166;GO:0003723;GO:0003746	BP:DNA-templated transcription, termination;BP:ribosomal small subunit biogenesis;BP:transcription antitermination;BP:DNA-templated transcription, termination;BP:transcription antitermination;BP:transcription antitermination;BP:DNA-templated transcription, termination;CC:cytoplasm;CC:cytoplasm;CC:cytoplasm;CC:ribosome;MF:transcription factor activity, sequence-specific DNA binding;MF:nucleotide binding;MF:RNA binding;MF:transcription factor activity, sequence-specific DNA binding;MF:nucleotide binding;MF:RNA binding;MF:transcription factor activity, sequence-specific DNA binding;MF:nucleotide binding;MF:RNA binding;MF:translation elongation factor activity;
BAB_RS16970	-	ABC transporter ATP-binding protein	1.047	0.06610544	0.313626371	0.476670544	no	up	mRNA	62.14	56.33	64.93	59.41	61.93	59.85	61.1333	60.396667	COG1125	E:Amino acid transport and metabolism	GO:0005524;GO:0016887	MF:ATP binding;MF:ATPase activity;

BAB_RS32670	-	cobalamin biosynthesis protein CbiG	1.047	0.066892652	0.417054602	0.580839374	no	up	mRNA	82.32	82.36	89.49	85.32	82.25	87.2	84.7233	84.923333	------	------	------	------
BAB_RS19235	-	hypothetical protein	1.047	0.066214329	0.424645576	0.588604934	no	up	mRNA	313.26	296.35	288.25	280.76	306.21	332.63	299.287	306.53333	ENOG4112560	S:Function unknown	[illegible]	[illegible]
BAB_RS27305	-	nucleoside deaminase	1.047	0.066630958	0.524487408	0.675963916	no	up	mRNA	16.07	16.53	16.77	17	15.43	16.56	16.4567	16.33	COG0590;COG0590	F:Nucleotide transport and metabolism;J:Translation, ribosomal structure and biogenesis	[illegible]	[illegible]
BAB_RS31705	-	aspartate aminotransferase family protein	1.047	0.066284612	0.626967815	0.752501901	no	up	mRNA	6.2	6.37	7.65	5.99	7.32	6.71	6.74	6.6733333	COG0161	E:Amino acid transport and metabolism	[illegible]	[illegible]
BAB_RS17845	-	aromatic acid exporter family protein	1.046	0.06548907	0.281721456	0.437915355	no	up	mRNA	214	214.63	240.6	207.04	226.25	227.94	223.077	220.41	COG4129	S:Function unknown	GO:0016021	CC:integral component of membrane;
BAB_RS19000	-	aminopeptidase N	1.046	0.064509078	0.293520104	0.452099757	no	up	mRNA	75.26	69.07	72.75	66.61	73.82	73.11	72.36	71.18	COG0308	E:Amino acid transport and metabolism	[illegible]	[illegible]
BAB_RS25485	-	DNA-binding response regulator	1.046	0.064707607	0.313733785	0.476608345	no	up	mRNA	473.75	392.31	423.17	428.47	423.77	419.47	429.743	423.90333	ENOG410XP65	T:Signal transduction mechanisms	[illegible]	[illegible]
BAB_RS23180	-	EamA/RhaT family transporter	1.046	0.064582209	0.318886787	0.482383827	no	up	mRNA	69.29	61.73	64.65	63.43	66.71	62.36	65.2233	64.166667	COG0697;ENOG4111KAW	S:Function unknown;	GO:0016021	CC:integral component of membrane;
BAB_RS27380	-	DNA mismatch repair protein MutL	1.046	0.065363527	0.321987203	0.484564349	no	up	mRNA	123.14	128.83	146.81	123.33	136.97	132.6	132.927	130.96667	COG0323	L:Replication, recombination and repair	[illegible]	[illegible]
BAB_RS17050	-	ABC transporter ATP-binding protein	1.046	0.064903414	0.361010713	0.527000191	no	up	mRNA	151.99	134.06	159.2	158.15	136.64	144.09	148.417	146.29333	COG3839;COG3842	G:Carbohydrate transport and metabolism;E:Amino acid transport and metabolism	[illegible]	[illegible]
BAB_RS20970	-	TIGR01620 family protein	1.046	0.064808529	0.372526485	0.538186869	no	up	mRNA	141.36	129.2	133.73	134.87	142.44	121.07	134.763	132.79333	COG3768	S:Function unknown	[illegible]	[illegible]
BAB_RS19320	-	DNA polymerase III subunit chi	1.046	0.064299404	0.376387014	0.541815177	no	up	mRNA	154.5	128	150.57	141.82	143.1	145.53	144.357	143.48333	COG2927	L:Replication, recombination and repair	[illegible]	[illegible]
BAB_RS17335	-	hypothetical protein	1.046	0.0653199	0.55500038	0.69984938	no	up	mRNA	18.61	17.16	18.71	16.01	19.82	17.86	18.16	17.896667	------	------	------	------
BAB_RS31235	-	ABC transporter ATP-binding protein	1.046	0.064456211	0.620923297	0.750009982	no	up	mRNA	23.65	23.29	25.38	27.68	24.49	19.17	24.1067	23.78	COG0444;COG1123	L:Replication, recombination and repair;S:Function unknown	[illegible]	[illegible]
BAB_RS33310	-	AraC family transcriptional regulator	1.046	0.06424952	0.714703217	0.822177385	no	up	mRNA	8.76	5.81	8.9	7.51	8.4	7.01	7.82333	7.64	ENOG411225V	K:Transcription	[illegible]	[illegible]
BAB_RS16185	-	Fe-S-binding ATPase	1.045	0.063016521	0.215381154	0.361934962	no	up	mRNA	69.83	63.66	70.82	65.7	68	66.9	68.1033	66.866667	COG0489	D:Cell cycle control, cell division, chromosome partitioning	[illegible]	[illegible]
BAB_RS25980	-	tryptophan synthase subunit beta	1.045	0.062893657	0.236927369	0.388193593	no	up	mRNA	267.87	272.7	293.21	273.91	276.76	271.26	277.927	273.97667	COG0133	E:Amino acid transport and metabolism	[illegible]	[illegible]
BAB_RS19780	-	heme A synthase	1.045	0.063148751	0.422663276	0.586363172	no	up	mRNA	29.05	25.02	27.56	27.63	27.06	25.44	27.21	26.71	COG1612	O:Posttranslational modification, protein turnover, chaperones	[illegible]	[illegible]
BAB_RS22530	-	urea transporter	1.045	0.063116251	0.476865327	0.638935903	no	up	mRNA	74.56	68.11	69.49	62.75	78.48	67.57	70.72	69.6	COG4413	E:Amino acid transport and metabolism	[illegible]	[illegible]
BAB_RS19075	-	DUF423 domain-containing protein	1.045	0.063315359	0.486786978	0.64576654	no	up	mRNA	57.96	59.89	72.61	56.33	67.85	63.79	63.4867	62.656667	COG2363	S:Function unknown	GO:0016021	CC:integral component of membrane;
BAB_RS20040	-	phosphoribosylformylglycinamidine synthase subunit PurS	1.045	0.063990748	0.489753609	0.648096518	no	up	mRNA	173.3	162.98	188.55	184.38	173.76	174.02	174.943	177.38667	COG1828	F:Nucleotide transport and metabolism	[illegible]	[illegible]
BAB_RS32505	-	hypothetical protein	1.045	0.063123073	0.574378801	0.7158569	no	up	mRNA	12.87	13.09	17.66	13.88	13.96	15.3	14.54	14.38	------	------	------	------
BAB_RS16900	-	hypothetical protein	1.045	0.064010078	0.758089461	0.854346348	no	up	mRNA	793.89	727.16	847.61	881.2	872.59	870.41	789.553	874.73333	------	------	------	------
BAB_RS25505	-	hypothetical protein	1.045	0.064173349	0.774237101	0.864658952	no	up	mRNA	27.09	18.39	16.33	21.77	18.15	20.87	20.6033	20.263333	------	------	------	------
BAB_RS17965	-	tetratricopeptide repeat protein	1.044	0.061562239	0.23358471	0.385468758	no	up	mRNA	235.25	221.22	235.86	229.77	229.46	221.13	230.777	226.78667	COG0457	S:Function unknown	------	------
BAB_RS30460	-	hypothetical protein	1.044	0.062705363	0.275014403	0.430824611	no	up	mRNA	634.15	582.75	580.5	575.95	588.08	616.42	599.133	593.48333	ENOG410YY9P	S:Function unknown	------	------
BAB_RS25405	-	histidinol-phosphate transaminase	1.044	0.061469178	0.283167895	0.439101567	no	up	mRNA	91.16	82.34	91.69	84.58	88.1	88.05	88.3967	86.91	COG0079	S:Function unknown	[illegible]	[illegible]
BAB_RS26705	-	KpsF/GutQ family sugar-phosphate isomerase	1.044	0.062249217	0.291998714	0.450837035	no	up	mRNA	93.76	89.8	100.22	95.1	95.42	88.62	94.5933	93.046667	COG0517	S:Function unknown	[illegible]	[illegible]
BAB_RS22465	-	ABC transporter substrate-binding protein	1.044	0.061978998	0.374049889	0.538933764	no	up	mRNA	79.31	81.66	86.73	84.37	82.31	78.24	82.5667	81.64	COG0614	P:Inorganic ion transport and metabolism	------	------
BAB_RS18820	-	hypothetical protein	1.044	0.062459795	0.469437678	0.632412268	no	up	mRNA	56.99	59.07	54.73	58.85	55.05	55.28	56.93	56.393333	COG5436	S:Function unknown	GO:0016021	CC:integral component of membrane;
BAB_RS17820	-	DSBA oxidoreductase	1.044	0.061930383	0.524650253	0.675902671	no	up	mRNA	23.23	19.19	23.12	18.65	21.94	23.63	21.8467	21.406667	COG1651	O:Posttranslational modification, protein turnover, chaperones	[illegible]	[illegible]
BAB_RS28195	-	aspartate aminotransferase family protein	1.044	0.062120102	0.613204769	0.745733127	no	up	mRNA	13.81	12.48	11.65	12.02	11.26	14	12.6467	12.426667	COG4992	S:Function unknown	[illegible]	[illegible]
BAB_RS18295	-	hypothetical protein	1.044	0.061777665	0.690468534	0.804962047	no	up	mRNA	22.07	25.05	26.08	23.39	22.33	28.25	24.4	24.656667	COG5349	S:Function unknown	GO:0016021	CC:integral component of membrane;
BAB_RS30700	-	regulatory protein NosR	1.044	0.062483452	0.81157026	0.88512398	no	up	mRNA	51.06	79.62	49.18	38.07	78.42	62.18	59.9533	59.556667	COG0348	C:Energy production and conversion	[illegible]	[illegible]
BAB_RS23285	-	peptidoglycan editing factor PgeF	1.043	0.060754129	0.340604067	0.505245091	no	up	mRNA	191.44	162.93	174.88	169.86	172.45	178.56	176.417	173.62333	COG1496	S:Function unknown	------	------
BAB_RS28675	-	iron ABC transporter permease	1.043	0.060718238	0.345948052	0.510344853	no	up	mRNA	64.71	59.92	70.04	63.7	62.59	65.41	64.89	63.9	COG0609	S:Function unknown	[illegible]	[illegible]
BAB_RS25525	-	membrane protein	1.043	0.060517168	0.594117779	0.731099358	no	up	mRNA	21.89	19.63	19.65	18.67	20.32	21.19	20.39	20.06	COG1738	S:Function unknown	[illegible]	[illegible]
BAB_RS27585	-	KR domain-containing protein	1.043	0.06045015	0.67798443	0.795313608	no	up	mRNA	14.65	15.08	21.22	15.44	17.4	17.87	16.9833	16.903333	ENOG410XNW1	S:Function unknown	[illegible]	[illegible]
BAB_RS28540	-	ABC transporter ATP-binding protein	1.043	0.06009149	0.707625239	0.817253736	no	up	mRNA	8.66	9.88	10.32	9.61	8.62	10.63	9.62	9.62	COG4608	S:Function unknown	[illegible]	[illegible]
BAB_RS30515	minC	septum site-determining protein MinC	1.042	0.058750284	0.227176919	0.376635419	no	up	mRNA	202.51	189.46	213.11	193.83	199.56	199.96	201.693	197.78333	COG0850	D:Cell cycle control, cell division, chromosome partitioning	[illegible]	[illegible]
BAB_RS17600	-	5-aminolevulinate synthase	1.042	0.058734556	0.274326286	0.430166109	no	up	mRNA	946.79	880.49	924.54	894.29	910.54	898.41	917.273	901.08	COG0156	H:Coenzyme transport and metabolism	[illegible]	[illegible]
BAB_RS29425	-	lipoate-protein ligase B	1.042	0.05923159	0.38950518	0.553997407	no	up	mRNA	49.12	48.96	55.1	52.01	50.43	48.25	51.06	50.23	COG0321	H:Coenzyme transport and metabolism	[illegible]	[illegible]
BAB_RS28045	-	organic hydroperoxide resistance protein	1.042	0.059287249	0.390291634	0.554379762	no	up	mRNA	63.33	65.14	66.2	61.62	67.88	61.93	64.89	63.81	COG1764	O:Posttranslational modification, protein turnover, chaperones	[illegible]	[illegible]
BAB_RS24890	-	glutaredoxin 3	1.042	0.059537031	0.547027032	0.694153971	no	up	mRNA	2103.6	1963.24	2064.88	2096.71	2262.92	2019.35	2043.91	2126.3267	COG0695	O:Posttranslational modification, protein turnover, chaperones	[illegible]	[illegible]
BAB_RS26835	-	hypothetical protein	1.042	0.058974512	0.591894148	0.728642106	no	up	mRNA	12.65	10.98	12.93	11.75	11.91	12.23	12.1867	11.963333	ENOG4111GBE	S:Function unknown	------	------
BAB_RS23335	-	MaoC family dehydratase	1.042	0.05895952	0.668558204	0.787418441	no	up	mRNA	22.05	25.7	20.97	22.13	24.07	21.86	22.9067	22.686667	COG2030	I:Lipid transport and metabolism	------	------
BAB_RS17475	-	multidrug efflux RND transporter permease subunit	1.041	0.057643854	0.269257205	0.424704663	no	up	mRNA	120.39	108.05	121.12	111.85	112.35	117.73	116.52	113.97667	COG0841	V:Defense mechanisms	[illegible]	[illegible]
BAB_RS25850	-	imidazoleglycerol-phosphate dehydratase	1.041	0.058274043	0.376866787	0.542262869	no	up	mRNA	50.87	43.44	53.41	50.62	47.77	45.93	49.24	48.106667	COG0131	E:Amino acid transport and metabolism	[illegible]	[illegible]
BAB_RS27360	-	DUF374 domain-containing protein	1.041	0.057893603	0.390485232	0.554409655	no	up	mRNA	182.11	176.59	201.47	194.8	174.07	183.23	186.723	184.03333	COG2121	S:Function unknown	------	------
BAB_RS17865	-	membrane protein	1.041	0.057817362	0.414940718	0.579150533	no	up	mRNA	137.57	130.95	132.88	140.81	125.13	130.19	133.8	132.04333	COG4649	S:Function unknown	GO:0016021	CC:integral component of membrane;
BAB_RS27955	-	RNA-binding transcriptional accessory protein	1.041	0.057820616	0.450915532	0.615459476	no	up	mRNA	27.85	25.86	28.31	23.94	28.35	27.98	27.34	26.756667	COG2183	K:Transcription	GO:0006139	[illegible]
BAB_RS21115	-	class I SAM-dependent methyltransferase	1.041	0.058580692	0.461860576	0.625351045	no	up	mRNA	40.89	41.43	40.08	40.96	40.7	38.83	40.8	40.163333	COG4976	S:Function unknown	[illegible]	[illegible]
BAB_RS28900	-	multicopper oxidase CueO	1.041	0.057319481	0.610488819	0.744120097	no	up	mRNA	11.97	9.18	12.24	10.26	10.43	11.91	11.13	10.866667	COG2132	Q:Secondary metabolites biosynthesis, transport and catabolism	[illegible]	[illegible]
BAB_RS32855	-	NAD(P)-dependent oxidoreductase	1.041	0.057305549	0.84231231	0.905133596	no	up	mRNA	23.23	13.07	17.36	18.99	19.22	15.33	17.8867	17.846667	COG1028	I:Lipid transport and metabolism	------	------
BAB_RS19665	-	MarR family transcriptional regulator	1.04	0.056041823	0.344618115	0.508616446	no	up	mRNA	151.77	143.27	149.34	140.67	145.06	151.28	148.127	145.67	ENOG4111FPQ	K:Transcription	[illegible]	[illegible]
BAB_RS25170	-	hypothetical protein	1.04	0.056989462	0.405246582	0.568335779	no	up	mRNA	370.08	330.37	336.25	327.77	340.23	371.86	345.567	346.62	ENOG4112BTW	S:Function unknown	GO:0016021	CC:integral component of membrane;
BAB_RS22270	-	cob(I)yrinic acid a%2Cc-diamide adenosyltransferase	1.04	0.056863232	0.488392359	0.646827968	no	up	mRNA	140.98	117.61	115.38	120.09	126.12	121.24	124.657	122.48333	COG2109	H:Coenzyme transport and metabolism	[illegible]	[illegible]
BAB_RS20715	-	queuine tRNA-ribosyltransferase	1.04	0.056227749	0.750455376	0.848719861	no	up	mRNA	8.93	9.85	13.39	10.62	11.06	10.53	10.7233	10.736667	COG0343	J:Translation, ribosomal structure and biogenesis	[illegible]	[illegible]
BAB_RS22040	-	gamma carbonic anhydrase family protein	1.039	0.055742231	0.307550582	0.469434689	no	up	mRNA	106.23	98.03	109.94	104.62	103.2	99.7	104.733	102.50667	COG0663	S:Function unknown	[illegible]	[illegible]
BAB_RS23475	-	anthranilate synthase component I	1.039	0.054959926	0.378924979	0.544249422	no	up	mRNA	72.17	70.12	72.42	73.96	66.77	69.31	71.57	70.013333	COG0512	E:Amino acid transport and metabolism	[illegible]	[illegible]
BAB_RS30875	-	hypothetical protein	1.039	0.05536557	0.401943465	0.565183524	no	up	mRNA	72.17	65.33	70.36	69.97	64.21	69.49	69.2867	67.89	------	------	GO:0016021	CC:integral component of membrane;
BAB_RS20975	-	hypothetical protein	1.039	0.054594656	0.417964841	0.581602873	no	up	mRNA	63.64	65.2	67.25	66.85	64.92	60.31	65.3633	64.026667	COG3106	S:Function unknown	------	------
BAB_RS25130	-	cell division protein ZapE	1.039	0.055436432	0.455149733	0.619659362	no	up	mRNA	58	50.77	55.11	55.2	49.35	55.85	54.6267	53.466667	COG1485	S:Function unknown	GO:0005524	[illegible]
BAB_RS31140	-	coniferyl aldehyde dehydrogenase	1.039	0.055599384	0.461685724	0.625377838	no	up	mRNA	36.27	29.53	33.34	32.79	32.02	31.89	33.0467	32.233333	COG1012	S:Function unknown	[illegible]	[illegible]
BAB_RS21580	-	UMP kinase	1.039	0.054766007	0.484912607	0.643811655	no	up	mRNA	78.04	70.57	79.09	80.83	68.82	73.98	75.9	74.543333	COG0528	F:Nucleotide transport and metabolism	[illegible]	[illegible]
BAB_RS24940	-	tRNA (adenosine(37)-N6)-threonylcarbamoyltransferase complex transferase subunit TsaD	1.039	0.054840548	0.505431066	0.66067942	no	up	mRNA	35.24	37.96	37.35	37.75	34.27	36.72	36.85	36.246667	COG0533	O:Posttranslational modification, protein turnover, chaperones	[illegible]	[illegible]
BAB_RS28230	-	transporter	1.039	0.055621984	0.523317649	0.676083477	no	up	mRNA	59.37	60.58	58.2	61.36	61.06	52.53	59.3833	58.316667	COG3586	S:Function unknown	[illegible]	[illegible]
BAB_RS24240	-	hypothetical protein	1.039	0.055354933	0.810010794	0.88402333	no	up	mRNA	16.8	11.83	17.85	12.97	17.84	15.62	15.4933	15.476667	ENOG410XV7D	S:Function unknown	GO:0016021	CC:integral component of membrane;
BAB_RS25255	-	amino acid ABC transporter permease	1.039	0.054733292	0.940879095	0.960916889	no	up	mRNA	0.21	0.61	0.82	0.52	0.22	1.1	0.54667	0.6133333	COG0765	E:Amino acid transport and metabolism	[illegible]	[illegible]
BAB_RS20485	-	cysteine desulfurase	1.038	0.054059965	0.360952661	0.527154955	no	up	mRNA	80.35	70.54	81.81	76.76	76.04	74.58	77.5667	75.793333	COG0520	E:Amino acid transport and metabolism	[illegible]	[illegible]
BAB_RS33490	-	hypothetical protein	1.038	0.053486798	0.375364779	0.540585852	no	up	mRNA	44.38	40.06	45.21	42.47	43.77	40.2	43.2167	42.146667	COG0251	S:Function unknown	------	------
BAB_RS30395	-	mannose-1-phosphate guanylyltransferase/mannose-6-phosphate isomerase	1.038	0.053875692	0.38336016	0.548169201	no	up	mRNA	112.71	99.46	107.57	99.12	108.94	104.4	106.58	104.15333	COG0836	P:Inorganic ion transport and metabolism	[illegible]	[illegible]
BAB_RS17945	-	Na+/H+ antiporter NhaA	1.038	0.053196019	0.394620141	0.558060966	no	up	mRNA	214.32	192.15	194.5	195.66	197.75	193.65	200.323	195.68667	COG0616;COG0616;COG3004	O:Posttranslational modification, protein turnover, chaperones;U:Intracellular trafficking, secretion, and vesicular transport;P:Inorganic ion transport and metabolism	[illegible]	[illegible]
BAB_RS17850	-	MFS transporter	1.038	0.054450627	0.494361854	0.65044416	no	up	mRNA	35.15	39.32	38.49	38.18	35.76	37	37.6533	36.98	ENOG410XNVC	G:Carbohydrate transport and metabolism	GO:0016021	CC:integral component of membrane;
BAB_RS17415	-	transcriptional regulator	1.038	0.054495205	0.530508766	0.681537251	no	up	mRNA	32.55	29.02	33.75	28.38	30.72	34.25	31.7733	31.116667	COG1309	K:Transcription	[illegible]	[illegible]
BAB_RS17110	-	hypothetical protein	1.038	0.053232125	0.554675296	0.699714065	no	up	mRNA	188.52	157.11	147.56	152.15	172.12	161.06	164.397	161.77667	------	------	------	------
BAB_RS23865	-	DNA-3-methyladenine glycosylase 2 family protein	1.038	0.054280262	0.624582471	0.752449749	no	up	mRNA	36.81	44.83	46.35	40.56	45.6	40.58	42.6633	42.246667	COG0122	L:Replication, recombination and repair	[illegible]	[illegible]
BAB_RS20685	-	hypothetical protein	1.038	0.053423998	0.633594846	0.757343839	no	up	mRNA	25.48	25.76	25.52	26.44	25.92	23.15	25.5867	25.17	ENOG410YZ7C	S:Function unknown	------	------
BAB_RS22600	-	PLP-dependent aminotransferase family protein	1.037	0.052565824	0.343804627	0.508116038	no	up	mRNA	567.43	518.95	558.35	513.88	536.22	556.49	548.243	535.53	COG1167	K:Transcription	[illegible]	[illegible]
BAB_RS26150	-	phosphate regulon transcriptional regulatory protein PhoB	1.037	0.052375003	0.439818838	0.605457552	no	up	mRNA	116.14	108.28	117.62	104.88	120.94	109.1	114.013	111.64	COG0745	P:Inorganic ion transport and metabolism	[illegible]	[illegible]
BAB_RS23610	-	DNA-binding response regulator	1.037	0.053025579	0.466170391	0.629594564	no	up	mRNA	961.37	826.7	838.67	854.54	825.29	888.94	875.58	856.25667	COG0745	S:Function unknown	[illegible]	[illegible]
BAB_RS22180	-	precorrin-3B C(17)-methyltransferase	1.037	0.052521619	0.547391075	0.694341699	no	up	mRNA	11.97	11.26	12.96	11.46	12.28	11.57	12.0633	11.77	COG2073	H:Coenzyme transport and metabolism	[illegible]	[illegible]
BAB_RS31385	-	glutamate-1-semialdehyde 2%2C1-aminomutase	1.037	0.051898665	0.625901631	0.752065049	no	up	mRNA	18.85	15.14	18.83	18.48	17.71	15.13	17.6067	17.106667	COG0001	H:Coenzyme transport and metabolism	[illegible]	[illegible]
BAB_RS23970	-	energy transducer TonB	1.037	0.05269218	0.729663219	0.834605882	no	up	mRNA	31.1	36.97	24.84	30.74	30.44	30.11	30.97	30.43	COG0810;ENOG410XUDZ	M:Cell wall/membrane/envelope biogenesis;S:Function unknown	------	------
BAB_RS32160	-	hypothetical protein	1.037	0.051791412	0.750693323	0.848690235	no	up	mRNA	13.56	14.38	18.43	17.94	14.36	13.71	15.4567	15.336667	------	------	------	------
BAB_RS19475	-	exonuclease	1.036	0.050517312	0.395028956	0.558393329	no	up	mRNA	42.92	40.1	46.13	41.98	42.82	41.06	43.05	41.953333	ENOG4111MFJ	S:Function unknown	[illegible]	[illegible]
BAB_RS21150	-	threonine--tRNA ligase	1.036	0.050734517	0.399194304	0.562795655	no	up	mRNA	135.37	122.38	134.94	136.4	124.06	122.09	130.897	127.51667	COG0441	J:Translation, ribosomal structure and biogenesis	[illegible]	[illegible]
BAB_RS28100	-	fructose 1%2C6-bisphosphatase	1.036	0.051142269	0.417617609	0.581371481	no	up	mRNA	66.22	62.17	65.54	64.75	62.98	61.85	64.6433	63.193333	COG0158	G:Carbohydrate transport and metabolism	[illegible]	[illegible]
BAB_RS22705	-	TetR/AcrR family transcriptional regulator	1.036	0.051489996	0.551380889	0.698024743	no	up	mRNA	53.4	48.05	57.38	45.75	55.04	54.53	52.9433	51.773333	ENOG41120HE	K:Transcription	[illegible]	[illegible]
BAB_RS16610	-	DNA mismatch repair protein MutS	1.035	0.049032591	0.367949222	0.533734018	no	up	mRNA	26.37	24.65	28.52	25.88	25.58	25.84	26.5133	25.766667	COG0249	L:Replication, recombination and repair	[illegible]	[illegible]
BAB_RS27375	-	DUF2093 domain-containing protein	1.035	0.049156065	0.39279534	0.556460066	no	up	mRNA	433.47	404.54	418.42	384.31	431.34	414.76	418.81	410.13667	COG3908	S:Function unknown	------	------
BAB_RS21685	-	NADPH-dependent 7-cyano-7-deazaguanine reductase QueF	1.035	0.048991674	0.403753927	0.567232779	no	up	mRNA	209.38	196.57	218.82	214.78	191.2	203.38	208.257	203.12	COG0780	S:Function unknown	[illegible]	[illegible]
BAB_RS19160	-	Crp/Fnr family transcriptional regulator	1.035	0.049258869	0.455547553	0.619938284	no	up	mRNA	220.98	213.16	223.91	218.94	227.47	197.17	219.35	214.52667	COG0664	F:Nucleotide transport and metabolism	[illegible]	[illegible]
BAB_RS18385	-	HAD family phosphatase	1.035	0.049607217	0.516819063	0.670112852	no	up	mRNA	19.36	18.61	22.49	19.87	18.86	20.29	20.1533	19.673333	COG1011	S:Function unknown	[illegible]	[illegible]
BAB_RS30990	-	pyrimidine 5'-nucleotidase	1.035	0.049294154	0.531165646	0.681835885	no	up	mRNA	41.73	39.55	38.72	39.43	39.74	37.94	40	39.036667	COG1011	E:Amino acid transport and metabolism	[illegible]	[illegible]
BAB_RS23400	-	aspartyl/asparaginyl beta-hydroxylase domain-containing protein	1.035	0.048938961	0.557283533	0.701077522	no	up	mRNA	286.7	240.91	236.11	233.24	269.76	245.06	254.573	249.35333	COG3555	O:Posttranslational modification, protein turnover, chaperones	GO:0018193	[illegible]
BAB_RS24300	-	5-(carboxyamino)imidazole ribonucleotide mutase	1.035	0.049855616	0.626734139	0.752502537	no	up	mRNA	80.61	67.6	70.98	80.57	68.49	65.49	73.0633	71.516667	COG0041	F:Nucleotide transport and metabolism	[illegible]	[illegible]
BAB_RS26070	-	molecular chaperone DnaJ	1.034	0.047769992	0.39545329	0.558501723	no	up	mRNA	379.3	370.19	396.45	378.1	387.4	351.24	381.98	372.24667	COG0484	O:Posttranslational modification, protein turnover, chaperones	[illegible]	[illegible]
BAB_RS24230	-	type I glyceraldehyde-3-phosphate dehydrogenase	1.034	0.048688856	0.401391968	0.56515004	no	up	mRNA	508.42	462.83	471.16	451.94	473.75	483.46	480.803	469.71667	COG0057	G:Carbohydrate transport and metabolism	[illegible]	[illegible]
BAB_RS30360	-	catalase	1.034	0.048507714	0.44683686	0.61223319	no	up	mRNA	680.12	635.68	668.48	612.04	627.3	694.16	661.427	644.5	COG0753	P:Inorganic ion transport and metabolism	[illegible]	[illegible]
BAB_RS19545	-	SH3 domain-containing protein	1.034	0.048494328	0.489469808	0.647987842	no	up	mRNA	99.43	102.18	114.45	94.28	108.74	105.19	105.353	102.73667	COG4991	S:Function unknown	------	------
BAB_RS27275	-	molecular chaperone GroES	1.034	0.047728335	0.50042067	0.656265965	no	up	mRNA	1575.23	1448.58	1446.22	1317.66	1594.8	1482.27	1490.01	1464.91	COG0234	O:Posttranslational modification, protein turnover, chaperones	[illegible]	[illegible]
BAB_RS30420	-	glutaminase	1.034	0.047759625	0.503050045	0.658370588	no	up	mRNA	50.08	50.87	54.79	51.04	53.17	47.81	51.9133	50.673333	COG2066	E:Amino acid transport and metabolism	[illegible]	[illegible]
BAB_RS21950	-	hypothetical protein	1.034	0.048820291	0.55875186	0.701825537	no	up	mRNA	292.75	248.08	236.48	270.23	238.13	259.27	259.103	255.87667	ENOG4111YS0;ENOG4112D5C	T:Signal transduction mechanisms;S:Function unknown	------	------
BAB_RS20400	-	peptide chain release factor 2	1.034	0.048567208	0.583672208	0.723510341	no	up	mRNA	192.24	164.45	178.27	198.17	161.03	161.83	178.32	173.67667	COG1186	J:Translation, ribosomal structure and biogenesis	[illegible]	[illegible]
BAB_RS31310	-	ABC transporter ATP-binding protein	1.034	0.048643921	0.688261614	0.803555439	no	up	mRNA	12.23	12.68	12.27	11.05	12.28	13.06	12.3933	12.13	COG0444	S:Function unknown	[illegible]	[illegible]
BAB_RS27195	-	glycolate oxidase subunit GlcE	1.034	0.048135513	0.691704959	0.805526653	no	up	mRNA	15.54	19.9	21.33	17.34	19.81	18.68	18.9233	18.61	COG0277	S:Function unknown	[illegible]	[illegible]
BAB_RS16015	-	acetyl/propionyl/methylcrotonyl-CoA carboxylase subunit alpha	1.034	0.047707352	0.774669947	0.864241159	no	up	mRNA	5.2	6.72	8.7	7.53	6.9	5.86	6.87333	6.7633333	COG4770	I:Lipid transport and metabolism	[illegible]	[illegible]
BAB_RS33315	-	hypothetical protein	1.034	0.047932811	0.941132189	0.960869947	no	up	mRNA	44.14	9.81	17.59	22.52	34.5	8.39	23.8467	21.803333	------	------	------	------
BAB_RS23650	-	histidine kinase	1.033	0.046896276	0.362307625	0.528653224	no	up	mRNA	125.53	125.05	133.58	121.19	125.62	126.58	128.053	124.46333	COG0642;ENOG410XNMH	S:Function unknown;	[illegible]	[illegible]
BAB_RS25970	-	tryptophan synthase subunit alpha	1.033	0.04715257	0.390687009	0.55420634	no	up	mRNA	216.89	193.87	219.3	208.65	205.85	200.12	210.02	204.87333	COG0159	E:Amino acid transport and metabolism	[illegible]	[illegible]
BAB_RS25085	-	dihydrolipoyl dehydrogenase	1.033	0.047242499	0.445023139	0.610789981	no	up	mRNA	463.92	417.58	443.87	401.56	456.46	432.7	441.79	430.24	COG1249	S:Function unknown	[illegible]	[illegible]
BAB_RS25710	-	ABC transporter substrate-binding protein	1.033	0.047014039	0.688635726	0.803700178	no	up	mRNA	81.32	62.76	58.24	69.48	64.46	62.31	67.44	65.416667	COG2984	S:Function unknown	------	------
BAB_RS24365	-	thiamine import ATP-binding protein ThiQ	1.033	0.046552549	0.777363119	0.866343288	no	up	mRNA	10.94	12.47	15.07	10.89	15.16	11.69	12.8267	12.58	COG3840	H:Coenzyme transport and metabolism	[illegible]	[illegible]
BAB_RS17980	-	glycine--tRNA ligase subunit beta	1.032	0.04605704	0.343280812	0.507809046	no	up	mRNA	91.2	86.98	95.76	88.92	88.48	88.67	91.3133	88.69	COG0751	J:Translation, ribosomal structure and biogenesis	[illegible]	[illegible]
BAB_RS28570	fabG	3-oxoacyl-ACP reductase	1.032	0.045953328	0.474471888	0.637057324	no	up	mRNA	119.81	111.68	113.35	106.57	114.2	115.64	114.947	112.13667	COG1028	G:Carbohydrate transport and metabolism	GO:0051301	[illegible]
BAB_RS21695	-	DUF924 domain-containing protein	1.032	0.045435907	0.64080937	0.763977924	no	up	mRNA	18.37	19.6	25.31	20.58	21	20.08	21.0933	20.553333	COG3803	S:Function unknown	GO:0016021	CC:integral component of membrane;
BAB_RS28295	-	nitronate monooxygenase	1.032	0.045790613	0.790565616	0.871385017	no	up	mRNA	6.6	6.43	9.45	7.84	7.7	6.51	7.49333	7.35	COG2070	S:Function unknown	[illegible]	[illegible]
BAB_RS21975	-	50S ribosomal protein L1	1.031	0.04414649	0.445494878	0.610915511	no	up	mRNA	1009.41	887.79	928.32	944.91	909.32	900.45	941.84	918.22667	COG0081	J:Translation, ribosomal structure and biogenesis	[illegible]	[illegible]
BAB_RS26220	-	methionine adenosyltransferase	1.031	0.044605865	0.484814356	0.643947303	no	up	mRNA	95.89	84.1	89.45	90.07	87.41	84.08	89.8133	87.186667	COG0192	H:Coenzyme transport and metabolism	[illegible]	[illegible]
BAB_RS27635	-	HAD family phosphatase	1.031	0.044198213	0.48724965	0.646113548	no	up	mRNA	140.8	132.57	142.63	140.74	137.99	126.66	138.667	135.13	COG0637	E:Amino acid transport and metabolism	[illegible]	[illegible]
BAB_RS25090	-	hypothetical protein	1.031	0.043412359	0.507226783	0.662218469	no	up	mRNA	125.92	115.89	115.84	115.72	118.54	114.52	119.217	116.26	ENOG4112173	S:Function unknown	------	------
BAB_RS27665	-	glyoxalase	1.031	0.044399396	0.50807715	0.662521056	no	up	mRNA	70.21	70.17	83.26	71.1	76.01	70.07	74.5467	72.393333	ENOG4111V4S;ENOG410Y4CG;COG0346	S:Function unknown;	GO:0051213	[illegible]
BAB_RS19770	-	chromosome partitioning protein ParA	1.031	0.04386168	0.551816388	0.698026006	no	up	mRNA	37.55	37.4	39.33	35.66	37.05	38.68	38.0933	37.13	COG0489;COG3206	M:Cell wall/membrane/envelope biogenesis;	------	------
BAB_RS21035	-	competence/damage-inducible protein A	1.031	0.043399789	0.553384124	0.699183323	no	up	mRNA	163.54	148.76	148.07	137.71	158.82	151.75	153.457	149.42667	COG1058	H:Coenzyme transport and metabolism	------	------
BAB_RS16750	-	16S rRNA (cytidine(1402)-2'-O)-methyltransferase	1.031	0.043391983	0.623393087	0.752144945	no	up	mRNA	38.39	42.09	45.81	41.78	43.02	38.6	42.0967	41.133333	COG0313	S:Function unknown	[illegible]	[illegible]
BAB_RS29660	-	UDP-glucose 4-epimerase GalE	1.031	0.044619834	0.864295544	0.916495572	no	up	mRNA	1.93	1.57	1.88	1.68	1.76	1.78	1.79333	1.74	COG1087	M:Cell wall/membrane/envelope biogenesis	[illegible]	[illegible]
BAB_RS23980	-	ABC transporter ATP-binding protein	1.031	0.044140737	0.872184975	0.920607859	no	up	mRNA	2.94	2.75	5.09	3.01	4.03	3.67	3.59333	3.57	COG0577;COG1136	V:Defense mechanisms;	[illegible]	[illegible]
BAB_RS17855	-	peptide transporter	1.03	0.042532822	0.419566947	0.583326959	no	up	mRNA	88.76	79.64	89.19	82.01	85.43	81.98	85.8633	83.14	COG1133	I:Lipid transport and metabolism	[illegible]	[illegible]
BAB_RS24185	-	GntR family transcriptional regulator	1.03	0.042997082	0.544628316	0.692203631	no	up	mRNA	26.57	23.62	29.37	25.66	26.4	24.99	26.52	25.683333	COG1802	K:Transcription	[illegible]	[illegible]
BAB_RS16270	-	LysE family translocator	1.03	0.042831308	0.596992615	0.733513297	no	up	mRNA	99.91	83.88	86.81	90.96	89.9	81.92	90.2	87.593333	COG1280	E:Amino acid transport and metabolism	[illegible]	[illegible]
BAB_RS23000	-	hypothetical protein	1.03	0.042889345	0.717379924	0.824666081	no	up	mRNA	55.24	48.38	50.24	50.83	53.52	47.38	51.2867	50.576667	------	------	------	------
BAB_RS29770	-	carbohydrate ABC transporter permease	1.03	0.042447324	0.724538357	0.830517923	no	up	mRNA	14.31	12.12	15.49	13.51	13.47	13.83	13.9733	13.603333	COG0395	S:Function unknown	[illegible]	[illegible]
BAB_RS28670	-	ABC transporter substrate-binding protein	1.029	0.040766722	0.477755793	0.639329181	no	up	mRNA	175.63	172.03	175.49	174.72	167.53	166.08	174.383	169.44333	COG0614	S:Function unknown	------	------
BAB_RS18160	-	phosphatidylserine decarboxylase family protein	1.029	0.041208191	0.485900478	0.644856768	no	up	mRNA	143.52	133.81	144.97	140.96	137.48	132.7	140.767	137.04667	COG0688	I:Lipid transport and metabolism	[illegible]	[illegible]
BAB_RS21435	-	pyruvate dehydrogenase complex E1 component subunit beta	1.029	0.04191747	0.490165466	0.648107672	no	up	mRNA	373.6	337.51	368.63	321.98	357.37	368.09	359.913	349.14667	COG0508	C:Energy production and conversion	[illegible]	[illegible]
BAB_RS30415	-	acid stress chaperone HdeA	1.029	0.041527584	0.492326069	0.649894684	no	up	mRNA	11527.5	10436.1	9926.01	9793.98	11331.23	10677.08	10629.8	10600.763	ENOG41128BT	O:Posttranslational modification, protein turnover, chaperones	[illegible]	[illegible]
BAB_RS27250	-	hypothetical protein	1.029	0.040873251	0.746956935	0.846849905	no	up	mRNA	18.54	14.07	18.12	13.79	17.83	17.27	16.91	16.296667	------	------	------	------
BAB_RS20915	-	ABC transporter	1.028	0.039883298	0.53431934	0.684516762	no	up	mRNA	237.68	217.27	228.87	213.35	238.63	213.77	227.94	221.91667	COG3218	S:Function unknown	------	------
BAB_RS17025	-	amidohydrolase	1.028	0.040210586	0.534286539	0.684747766	no	up	mRNA	76.81	69.12	82.07	70.65	71.67	78.44	76	73.586667	COG3618	S:Function unknown	[illegible]	[illegible]
BAB_RS24875	-	EamA/RhaT family transporter	1.028	0.039666542	0.599845946	0.735612604	no	up	mRNA	167.31	150.4	202.23	157.6	174.18	170.16	173.313	167.31333	ENOG410Y63J	S:Function unknown	GO:0016021	CC:integral component of membrane;
BAB_RS16705	-	adenosine kinase	1.028	0.040510641	0.602422713	0.736804026	no	up	mRNA	46.6	42.21	45.39	46.12	44.72	38.94	44.7333	43.26	COG0524	E:Amino acid transport and metabolism	[illegible]	[illegible]
BAB_RS25285	-	GntR family transcriptional regulator	1.028	0.039220645	0.690762361	0.805012502	no	up	mRNA	44.05	37.32	39.4	35.61	36.58	44.61	40.2567	38.933333	COG1802	E:Amino acid transport and metabolism	[illegible]	[illegible]
BAB_RS25390	-	TIGR02302 family protein	1.027	0.037770492	0.456430334	0.619826992	no	up	mRNA	120.33	120.82	130.49	117.74	119.78	121.42	123.88	119.64667	ENOG410XPAM	S:Function unknown	GO:0016021	CC:integral component of membrane;
BAB_RS24340	-	ABC transporter ATP-binding protein	1.027	0.038348658	0.518732158	0.671509438	no	up	mRNA	47.78	41.48	50.03	45.89	45.42	42.92	46.43	44.743333	COG0488	S:Function unknown	[illegible]	[illegible]

BAB_RS31835	-	branched-chain amino acid ABC transporter substrate-binding protein	1.027	0.038193362	0.535276105	0.684923189	no	up	mRNA	494.91	439.16	468.96	420.76	466.59	470.97	467.677	452.77333	ENOG410XP0X	E:Amino acid transport and metabolism	GO:0006865	BP:amino acid transport;
BAB_RS21960	-	DNA-directed RNA polymerase subunit beta	1.027	0.038227233	0.571200944	0.713557012	no	up	mRNA	1003.57	865.59	945.51	906.31	893.7	916.8	938.223	905.60333	COG0085	K:Transcription	GO:0006351;GO:0006351;GO:0006351;GO:0003677;GO:0003899;GO:0032549;GO:0016491;GO:0003899;GO:0003677;GO:0032549;GO:0003899;GO:0032549;GO:0003677	BP:transcription, DNA-templated;BP:transcription, DNA-templated;BP:transcription, DNA-templated;MF:DNA binding;MF:DNA-directed RNA polymerase activity;MF:ribonucleoside binding;MF:oxidoreductase activity;MF:DNA-directed RNA polymerase activity;MF:DNA binding;MF:ribonucleoside binding;MF:DNA-directed RNA polymerase activity;MF:ribonucleoside binding;MF:DNA binding;
BAB_RS16455	-	PhzF family phenazine biosynthesis protein	1.027	0.038368186	0.583747703	0.723324864	no	up	mRNA	75.74	72.32	83.35	68.39	78.59	77.22	77.1367	74.733333	COG0384	S:Function unknown	GO:0009058;GO:0003824	BP:biosynthetic process;MF:catalytic activity;
BAB_RS18125	-	1-deoxy-D-xylulose-5-phosphate synthase	1.027	0.038265036	0.583465508	0.723533261	no	up	mRNA	51.86	55.95	59.13	54.25	56.32	51.03	55.6467	53.866667	COG1154	H:Coenzyme transport and metabolism	GO:0052865;GO:0016114;GO:0009228;GO:0052865;GO:0009228;GO:0016114;GO:0052865;GO:0016114;GO:0009228;GO:0008661;GO:0002 87;GO:0030976;GO:0008661;GO:000287;GO:0030976;GO:0008661;GO:0030976;GO:0000287;GO:0046872	BP:1-deoxy-D-xylulose 5-phosphate biosynthetic process;BP:terpenoid biosynthetic process;BP:thiamine biosynthetic process;BP:1-deoxy-D-xylulose 5-phosphate biosynthetic process;BP:thiamine biosynthetic process;BP:terpenoid biosynthetic process;BP:1-deoxy-D-xylulose 5-phosphate biosynthetic process;BP:terpenoid biosynthetic process;BP:thiamine biosynthetic process;MF:1-deoxy-D-xylulose-5-phosphate synthase activity;MF:magnesium ion binding;MF:thiamine pyrophosphate binding;MF:1-deoxy-D-xylulose-5-phosphate synthase activity;MF:magnesium ion binding;MF:thiamine pyrophosphate binding;MF:1-deoxy-D-xylulose-5-phosphate synthase activity;MF:thiamine pyrophosphate binding;MF:magnesium ion binding;MF:metal
BAB_RS31485	-	zinc ABC transporter substrate-binding protein	1.027	0.038876222	0.596753501	0.733499999	no	up	mRNA	69.05	69.69	66.7	68.11	63.39	68.06	68.48	66.52	COG4531	P:Inorganic ion transport and metabolism	GO:0006829;GO:0009405;GO:0030001;GO:0006829;GO:0042597;GO:0046872;GO:0046872;GO:0046872	BP:zinc II ion transport;BP:pathogenesis;BP:metal ion transport;BP:zinc II ion transport;CC:periplasmic space;MF:metal ion binding;MF:metal ion binding;MF:metal ion binding;
BAB_RS18115	-	DUF937 domain-containing protein	1.027	0.0378521	0.645505843	0.767867558	no	up	mRNA	649.05	676.82	748.84	594.54	737.33	693.44	691.57	675.10333	COG3753	S:Function unknown	GO:0005840	CC:ribosome;
BAB_RS32985	-	hypothetical protein	1.027	0.037972375	0.6620758	0.782364673	no	up	mRNA	20.37	18.84	24.81	20.52	22.03	19.2	21.34	20.583333	------	------	------	------
BAB_RS29975	-	MFS transporter	1.027	0.038496878	0.712882529	0.820670571	no	up	mRNA	19.42	23.51	22.5	22.97	19.61	21.15	21.81	21.243333	ENOG410XPP0	P:Inorganic ion transport and metabolism	GO:0055085;GO:0016021	BP:transmembrane transport;CC:integral component of membrane;
BAB_RS17040	-	sugar ABC transporter permease	1.027	0.038490765	0.720958284	0.827595201	no	up	mRNA	158.55	142.2	152.07	174.01	138.65	125.97	150.94	146.21	ENOG4111EY7	G:Carbohydrate transport and metabolism	GO:0055085;GO:0055085;GO:0016021;GO:0005886;GO:0016021;GO:0005886	BP:transmembrane transport;BP:transmembrane transport;CC:integral component of membrane;CC:plasma membrane;CC:integral component of membrane;CC:plasma membrane;
BAB_RS25290	-	L-asparaginase	1.027	0.037979793	0.805101279	0.880759418	no	up	mRNA	173.36	169.75	165.78	131.26	146.55	216.71	169.63	164.84	COG0252	E:Amino acid transport and metabolism	GO:0004067	MF:asparaginase activity;
BAB_RS31730	-	aldehyde dehydrogenase	1.027	0.038648445	0.861696307	0.916461514	no	up	mRNA	2.03	3.3	3.15	2.37	3.2	2.78	2.82667	2.7833333	COG1012	S:Function unknown	GO:0016620;GO:0004029;GO:0016620	MF:oxidoreductase activity, acting on the aldehyde or oxo group of donors, NAD or NADP as acceptor;MF:aldehyde dehydrogenas;MF:oxidoreductase activity, acting on the aldehyde or oxo group of donors, NAD or NADP as acceptor;
BAB_RS19560	-	cystathionine beta-lyase	1.026	0.03679787	0.552002838	0.697712478	no	up	mRNA	102.32	102.92	104.34	94.82	100.48	104.34	103.193	99.88	COG0626	S:Function unknown	GO:0006520;GO:0006520;GO:0006520;GO:0005737;GO:0005737;GO:0005737;GO:0004121;GO:0030170;GO:0004121;GO:0030170;GO:0004121;GO:0047396;GO:0030170	BP:cellular amino acid metabolic process;BP:cellular amino acid metabolic process;BP:cellular amino acid metabolic process;CC:cytoplasm;CC:cytoplasm;CC:cytoplasm;MF:cystathionine beta-lyase activity;MF:pyridoxal phosphate binding;MF:cystathionine beta-lyase activity;MF:pyridoxal phosphate binding;MF:cystathionine beta-lyase activity;MF:glycosylphosphatidylinositol diacylglycerol-lyase activity;MF:pyridoxal phosphate binding;
BAB_RS17370	-	Urease accessory protein UreF 1	1.026	0.037669146	0.680780981	0.798302661	no	up	mRNA	47.63	53.73	56.4	50.09	50.84	53.53	52.5867	51.486667	COG0830	O:Posttranslational modification, protein turnover, chaperones	GO:0006807;GO:0006807;GO:0009405;GO:0005737;GO:0005737;GO:0016151;GO:0016151	BP:nitrogen compound metabolic process;BP:nitrogen compound metabolic process;BP:pathogenesis;CC:cytoplasm;CC:cytoplasm;MF:nickel cation binding;MF:nickel cation binding;
BAB_RS27905	-	AcrB/AcrD/AcrF family protein	1.025	0.036242858	0.508800374	0.662926034	no	up	mRNA	63.88	58.66	64.05	60.27	57.57	62.07	62.1967	59.97	COG0841	M:Cell wall/membrane/envelope biogenesis	GO:0016020;GO:0016021;GO:0022857	CC:membrane;CC:integral component of membrane;MF:transmembrane transporter activity;
BAB_RS25150	-	signal recognition particle-docking protein FtsY	1.025	0.03546011	0.542473643	0.691379538	no	up	mRNA	87.41	79.15	87.39	83.94	82.49	78.61	84.65	81.68	COG0552	U:Intracellular trafficking, secretion, and vesicular transport	GO:0006614;GO:0006614;GO:0051301;GO:0005737;GO:0031226;GO:0005737;GO:0031226;GO:0005525;GO:0003924;GO:0003924;GO:0005525	BP:SRP-dependent cotranslational protein targeting to membrane;BP:SRP-dependent cotranslational protein targeting to membrane;BP:cell division;CC:cytoplasm;CC:intrinsic component of plasma membrane;CC:cytoplasm;CC:intrinsic component of plasma membrane;MF:GTP binding;MF:GTPase activity;MF:GTPase activity;MF:GTP binding;
BAB_RS21430	-	pyruvate dehydrogenase complex dihydrolipoamide acetyltransferase	1.025	0.035717503	0.574875116	0.715920057	no	up	mRNA	305.11	283.35	291.73	260.9	291.94	298.19	293.397	283.67667	COG0508	C:Energy production and conversion	GO:0006090;GO:0006090;GO:0006090;GO:0045254;GO:0045254;GO:0045254;GO:0008168;GO:0004742;GO:0004742;GO:0004742	BP:pyruvate metabolic process;BP:pyruvate metabolic process;BP:pyruvate metabolic process;CC:pyruvate dehydrogenase complex;CC:pyruvate dehydrogenase complex;CC:pyruvate dehydrogenase complex;MF:methyltransferase activity;MF:dihydrolipoyllysine-residue acetyltransferase activity;MF:dihydrolipoyllysine-residue acetyltransferase activity;MF:dihydrolipoyllysine-residue acetyltransferase activity;
BAB_RS19380	-	glutathione S-transferase	1.025	0.03516717	0.585044308	0.72353632	no	up	mRNA	122.06	118.92	117.61	116.55	116.71	115.29	119.53	116.18333	COG0625	L:Replication, recombination and repair	GO:0016740;GO:0016740;GO:0004812	MF:transferase activity;MF:transferase activity;MF:aminoacyl-tRNA ligase activity;
BAB_RS23695	-	isocitrate lyase	1.025	0.036114094	0.602243864	0.736865778	no	up	mRNA	139.12	125.02	136.31	116.14	136.85	133.77	133.483	128.92	COG2224	C:Energy production and conversion	GO:0019752;GO:0019752;GO:0004451;GO:0004451;GO:0051864	BP:carboxylic acid metabolic process;BP:carboxylic acid metabolic process;MF:isocitrate lyase activity;MF:isocitrate lyase activity;MF:histone demethylase activit;
BAB_RS19195	-	ribonuclease III	1.025	0.035087071	0.630151086	0.754350015	no	up	mRNA	137.8	128.82	139.81	137.62	118.53	137.79	135.477	131.31333	COG0571	K:Transcription	GO:0006397;GO:0016075;GO:0006364;GO:0008033;GO:0008033;GO:0016075;GO:0006364;GO:0006397;GO:0005737;GO:0005737;GO:0004672;GO:0004525;GO:0019843;GO:0004525;GO:0019843;GO:0046872	BP:mRNA processing;BP:rRNA catabolic process;BP:rRNA processing;BP:tRNA processing;BP:tRNA processing;BP:rRNA catabolic process;BP:rRNA processing;BP:mRNA processing;CC:cytoplasm;CC:cytoplasm;MF:metal ion binding;MF:ribonuclease III activity;MF:rRNA binding;MF:ribonuclease III activity;MF:rRNA binding;MF:metal ion binding;
BAB_RS17275	-	low molecular weight phosphatase family protein	1.025	0.036230117	0.644681852	0.767455647	no	up	mRNA	147.64	119.94	143.19	130.38	141.69	126.86	136.923	132.97667	COG0394	J:Translation, ribosomal structure and biogenesis	------	------
BAB_RS18055	-	histidine phosphatase family protein	1.025	0.035510005	0.649984141	0.770911423	no	up	mRNA	43.36	37.58	39.81	39.06	38.78	38.77	40.25	38.87	COG0406	G:Carbohydrate transport and metabolism	------	------
BAB_RS25440	-	1-acyl-sn-glycerol-3-phosphate acyltransferase	1.025	0.036035128	0.655896276	0.775918533	no	up	mRNA	58.59	51.25	53.36	56.94	50.7	49.91	54.4	52.516667	COG0204	I:Lipid transport and metabolism	GO:0008152;GO:0008152;GO:0008152;GO:0008152;GO:0016021;GO:0016021;GO:0016021;GO:0016021;GO:0016746;GO:0003841;GO:0016746;GO:0003841;GO:0016746	BP:metabolic process;BP:metabolic process;BP:metabolic process;BP:metabolic process;CC:integral component of membrane;CC:integral component of membrane;CC:integral component of membrane;CC:integral component of membrane;MF:transferase activity, transferring acyl groups;MF:1-acylglycerol-3-phosphate O-acyltransferase activity;MF:transferase activity, transferring acyl groups;MF:1-acylglycerol-3-phosphate O-acyltransferase activity;MF:transferase activity, transferring acyl groups;
BAB_RS32830	-	hypothetical protein	1.025	0.035474637	0.770682479	0.862487916	no	up	mRNA	12.56	12.82	18.53	13.74	14.32	14.5	14.6367	14.186667	------	------	------	------
BAB_RS31580	-	flagellar hook protein FlgE	1.025	0.035722657	0.786423935	0.870702999	no	up	mRNA	7.37	8.07	7.74	8.01	7.01	7.5	7.72667	7.5066667	COG1749	N:Cell motility	GO:0071973;GO:0009425	BP:bacterial-type flagellum-dependent cell motility;CC:bacterial-type flagellum basal body;
BAB_RS26925	-	flagellar biosynthesis protein FlhB	1.025	0.035814984	0.829116	0.89544528	no	up	mRNA	5.73	4.31	5.79	5.65	4.53	5.09	5.27667	5.09	COG1377	N:Cell motility	GO:0009306;GO:0009306;GO:0044780;GO:0016021;GO:0016021;GO:0005886	BP:protein secretion;BP:protein secretion;BP:bacterial-type flagellum assembly;CC:integral component of membrane;CC:integral component of membrane;CC:plasma membrane;
BAB_RS33000	-	hypothetical protein	1.025	0.03591447	0.873099135	0.921270121	no	up	mRNA	21.05	13.18	18.72	12.08	15.83	23.05	17.65	16.986667	------	------	GO:0016021	CC:integral component of membrane;
BAB_RS28550	-	bifunctional methylenetetrahydrofolate dehydrogenase/methenyltetrahydrofolate cyclohydrolase	1.024	0.034389266	0.621028178	0.749854767	no	up	mRNA	36.53	31.49	38.36	35.74	34.72	31.76	35.46	34.073333	COG0190	H:Coenzyme transport and metabolism	GO:0000105;GO:0009086;GO:0006164;GO:0035999;GO:0035999;GO:0006164;GO:0000105;GO:0009086;GO:0004477;GO:0004488;GO:0004477;GO:0004488	BP:histidine biosynthetic process;BP:methionine biosynthetic process;BP:purine nucleotide biosynthetic process;BP:tetrahydrofolate interconversion;BP:tetrahydrofolate interconversion;BP:purine nucleotide biosynthetic process;BP:histidine biosynthetic process;BP:methionine biosynthetic process;MF:methenyltetrahydrofolate cyclohydrolase activity;MF:methylenetetrahydrofolate dehydrogenase activity;MF:methenyltetrahydrofolate cyclohydrolase activity;MF:methylenetetrahydrofolate dehydrogenas;
BAB_RS17970	-	glycine--tRNA ligase subunit alpha	1.024	0.03472485	0.625741033	0.752434857	no	up	mRNA	117.83	111.71	112.62	118.31	103.39	109.25	114.053	110.31667	COG0752	J:Translation, ribosomal structure and biogenesis	GO:0006426;GO:0006426;GO:0005737;GO:0005737;GO:0005524;GO:0004820;GO:0004820;GO:0005524	BP:glycyl-tRNA aminoacylation;BP:glycyl-tRNA aminoacylation;CC:cytoplasm;CC:cytoplasm;MF:ATP binding;MF:glycine-tRNA ligase activity;MF:glycine-tRNA ligase activity;MF:ATP binding;
BAB_RS18305	-	4-hydroxy-3-methylbut-2-enyl diphosphate reductase	1.024	0.034608119	0.641663432	0.764712392	no	up	mRNA	48.39	46.44	54.01	51.08	47.09	45.82	49.6133	47.996667	COG0761	I:Lipid transport and metabolism	GO:0016114;GO:0050992;GO:0019288;GO:0016114;GO:0050992;GO:0019288;GO:0050992;GO:0019288;GO:0050992;GO:0019288;GO:0016114;GO:0051539;GO:0051745;GO:0046872;GO:0051539;GO:0051745;GO:0046872;GO:0051539;GO:0051745;GO:0046872;GO:0051539;GO:0051745;GO:0046872	BP:terpenoid biosynthetic process;BP:dimethylallyl diphosphate biosynthetic process;BP:isopentenyl diphosphate biosynthetic process, methylerythritol 4-phosphate pathway;BP:terpenoid biosynthetic process;BP:dimethylallyl diphosphate biosynthetic process;BP:isopentenyl diphosphate biosynthetic process, methylerythritol 4-phosphate pathway;BP:dimethylallyl diphosphate biosynthetic process;BP:isopentenyl diphosphate biosynthetic process, methylerythritol 4-phosphate pathway;BP:dimethylallyl diphosphate biosynthetic process;BP:isopentenyl diphosphate biosynthetic process, methylerythritol 4-phosphate pathway;BP:terpenoid biosynthetic process;MF:4 iron, 4 sulfur cluster binding;MF:4-hydroxy-3-methylbut-2-en-1-yl diphosphate reductase activity;MF:metal ion binding;MF:4 iron, 4 sulfur cluster binding;MF:4-hydroxy-3-methylbut-2-en-1-yl diphosphate reductase activity;MF:metal ion binding;MF:4 iron, 4 sulfur cluster binding;MF:4-hydroxy-3-methylbut-2-en-1-yl diphosphate reductase activity;MF:metal ion binding;MF:4 iron, 4 sulfur cluster binding;MF:4-hydroxy-3-methylbut-2-en-1-yl diphosphate reductase activity;MF:metal ion binding;
BAB_RS28990	-	ribosomal RNA large subunit methyltransferase E	1.024	0.034741581	0.667522446	0.786775355	no	up	mRNA	82.56	78.62	97.61	83.98	87.48	79.82	86.2633	83.76	COG0293	J:Translation, ribosomal structure and biogenesis	GO:0006364;GO:0001510;GO:0051301;GO:0005737;GO:0005737;GO:0008650;GO:0008168;GO:0008650	BP:rRNA processing;BP:RNA methylation;BP:cell division;CC:cytoplasm;CC:cytoplasm;MF:rRN;MF:methyltransferase activity;MF:rRN;
BAB_RS22365	-	lysylphosphatidylglycerol synthetase family protein	1.024	0.033539319	0.693163814	0.806640831	no	up	mRNA	171.52	144.22	139.62	153.15	145.57	140.74	151.787	146.48667	COG0392	S:Function unknown	GO:0016021	CC:integral component of membrane;
BAB_RS21340	-	ATP-dependent Clp protease ATP-binding subunit ClpX	1.023	0.033387149	0.551974724	0.697951511	no	up	mRNA	938.35	900.47	946.55	862.54	901.82	921.63	928.457	895.33	COG1219	O:Posttranslational modification, protein turnover, chaperones	GO:0006457;GO:0051082;GO:0008270;GO:0008233;GO:0046983;GO:0005524	BP:protein folding;MF:unfolded protein binding;MF:zinc ion binding;MF:peptidase activity;MF:protein dimerization activity;MF:ATP binding;
BAB_RS22490	-	glycoside hydrolase family 10 protein	1.023	0.033127332	0.604964206	0.738787532	no	up	mRNA	35.45	31.91	36.74	32.7	34.77	32.69	34.7	33.386667	COG1649	S:Function unknown	GO:0003824;GO:0016787;GO:0003824	MF:catalytic activity;MF:hydrolase activity;MF:catalytic activity;
BAB_RS26155	-	cell wall hydrolase	1.023	0.032988518	0.61601549	0.74801881	no	up	mRNA	596.32	623.69	687.84	583.75	600.59	655.87	635.95	613.40333	COG3773	P:Inorganic ion transport and metabolism	GO:0016787	MF:hydrolase activity;
BAB_RS21735	-	glutamate racemase	1.023	0.032907383	0.618503541	0.749058378	no	up	mRNA	83.76	86.59	90.13	80.68	88.1	83.25	86.8267	84.01	COG0796	M:Cell wall/membrane/envelope biogenesis	GO:0071555;GO:0008360;GO:0009252;GO:0071555;GO:0009252;GO:0008360;GO:0008881;GO:0008881	BP:cell wall organization;BP:regulation of cell shape;BP:peptidoglycan biosynthetic process;BP:cell wall organization;BP:peptidoglycan biosynthetic process;BP:regulation of cell shape;MF:glutamate racemase activity;MF:glutamate racemase activity;
BAB_RS25465	-	aquaporin	1.023	0.032158277	0.637574939	0.7609689	no	up	mRNA	408.03	359.93	372.36	348.93	353.05	400.53	380.107	367.50333	COG0580	G:Carbohydrate transport and metabolism	GO:0016021;GO:0005886;GO:0015250	CC:integral component of membrane;CC:plasma membrane;MF:water channel activity;
BAB_RS25305	-	universal stress protein	1.023	0.033263873	0.666270158	0.785875924	no	up	mRNA	2266.56	2244.31	2443.37	2005.56	2301.13	2495.46	2318.08	2267.3833	COG0589	G:Carbohydrate transport and metabolism	GO:0006950;GO:0006950;GO:0005737;GO:0005737	BP:response to stress;BP:response to stress;CC:cytoplasm;CC:cytoplasm;
BAB_RS18025	-	phosphoribosylamine--glycine ligase	1.023	0.032533452	0.670364162	0.788678159	no	up	mRNA	33.91	28.05	31.87	31.22	28.82	30.11	31.2767	30.05	COG0151	F:Nucleotide transport and metabolism	GO:0009113;GO:0006189;GO:0016798;GO:0005524;GO:0046872;GO:0004637	BP:purine nucleobase biosynthetic process;BP:'de novo' IMP biosynthetic process;MF:hydrolase activity, acting on glycosyl bonds;MF:ATP binding;MF:metal ion binding;MF:phosphoribosylamine-glycine ligase activity;
BAB_RS27490	-	hypothetical protein	1.023	0.033154166	0.702572035	0.813757732	no	up	mRNA	16.45	15.24	17.42	14.92	16.11	16.32	16.37	15.783333	ENOG4111EZU	S:Function unknown	------	------
BAB_RS22035	-	hypothetical protein	1.023	0.032312091	0.703422536	0.814155839	no	up	mRNA	57.33	53.67	52.61	50.65	49.06	58.19	54.5367	52.633333	COG3672	S:Function unknown	------	------
BAB_RS19395	-	23S rRNA (adenine(2030)-N(6))-methyltransferase RlmJ	1.023	0.032587511	0.712703195	0.820758196	no	up	mRNA	52.13	44.69	45.21	48.68	45.63	42.32	47.3433	45.543333	COG2961	S:Function unknown	GO:0070475;GO:0070475;GO:0003723;GO:0036307;GO:0036307;GO:0003723	BP:rRNA base methylation;BP:rRNA base methylation;MF:RNA binding;MF:23S rRN;MF:23S rRN;MF:RNA binding;
BAB_RS27355	-	DUF4170 domain-containing protein	1.023	0.032614297	0.796867435	0.875927153	no	up	mRNA	536.68	536.43	545.65	620.35	496.6	541.24	539.587	552.73	ENOG41122CC	S:Function unknown	------	------
BAB_RS16915	-	thiaminase II	1.023	0.033386102	0.861072428	0.916101228	no	up	mRNA	9.91	7.2	7.47	7.34	7.22	8.98	8.19333	7.8466667	COG0819	K:Transcription	GO:0009228;GO:0009229;GO:0009229;GO:0009228;GO:0050334;GO:0050334	BP:thiamine biosynthetic process;BP:thiamine diphosphate biosynthetic process;BP:thiamine diphosphate biosynthetic process;BP:thiamine biosynthetic process;MF:thiaminase activity;MF:thiaminase activity;
BAB_RS16415	-	formate transporter	1.022	0.030917917	0.572997663	0.714967569	no	up	mRNA	142.74	125.12	142.84	133.36	129.2	132.55	136.9	131.70333	ENOG410ZGAD	S:Function unknown	------	------
BAB_RS21770	-	DNA recombination/repair protein RecA	1.022	0.03189652	0.590865856	0.727934048	no	up	mRNA	184.56	168.2	178.49	158.87	176.65	176.3	177.083	170.60667	COG0468	L:Replication, recombination and repair	GO:0006310;GO:0006281;GO:0009432;GO:0006310;GO:0006281;GO:0009432;GO:0006310;GO:0006281;GO:0009432;GO:0005737;GO:0005737;GO:0005737;GO:0008094;GO:0003684;GO:0003697;GO:0005524;GO:0005524;GO:0008094;GO:0003697;GO:0005524;GO:0003684;GO:0008094;GO:0003697	BP:DNA recombination;BP:DNA repair;BP:SOS response;BP:DNA recombination;BP:DNA repair;BP:SOS response;BP:DNA recombination;BP:DNA repair;BP:SOS response;CC:cytoplasm;CC:cytoplasm;CC:cytoplasm;MF:DNA-dependent ATPase activity;MF:damaged DNA binding;MF:single-stranded DNA binding;MF:ATP binding;MF:ATP binding;MF:DNA-dependent ATPase activity;MF:single-stranded DNA binding;MF:ATP binding;MF:damaged DNA binding;MF:DNA-dependent ATPase activity;MF:single-stranded DNA binding;
BAB_RS23875	-	malate synthase G	1.022	0.031693428	0.646848091	0.7688949	no	up	mRNA	56.48	53.39	53.26	48.75	53.53	54.74	54.3767	52.34	COG2225	C:Energy production and conversion	GO:0006097;GO:0006099;GO:0006099;GO:0006097;GO:0005737;GO:0005737;GO:0004474;GO:0046872;GO:0046872;GO:0004474	BP:glyoxylate cycle;BP:tricarboxylic acid cycle;BP:tricarboxylic acid cycle;BP:glyoxylate cycle;CC:cytoplasm;CC:cytoplasm;MF:malate synthase activity;MF:metal ion binding;MF:metal ion binding;MF:malate synthase activity;
BAB_RS24920	-	heme biosynthesis protein HemY	1.022	0.031079645	0.649112604	0.770162038	no	up	mRNA	404.1	410.55	470.06	375.98	434.18	426.17	428.237	412.11	COG3898	S:Function unknown	GO:0016021	CC:integral component of membrane;
BAB_RS31805	-	bicyclomycin resistance protein	1.022	0.031144058	0.664524903	0.784393282	no	up	mRNA	53.73	46.63	49.24	47.52	48.43	48.03	49.8667	47.993333	COG0715	P:Inorganic ion transport and metabolism	GO:0016020;GO:0042597;GO:0016020;GO:0042626;GO:0042626	CC:membrane;CC:periplasmic space;CC:membrane;MF:ATPase activity, coupled to transmembrane movement of substances;MF:ATPase activity, coupled to transmembrane movement of substances;
BAB_RS18415	-	glyoxalase	1.022	0.031215167	0.665569176	0.785337409	no	up	mRNA	71.64	75.72	76.03	73.5	72.4	70.71	74.4633	72.203333	COG3324	S:Function unknown	GO:0051213	MF:dioxygenase activity;
BAB_RS26635	-	P-type DNA transfer ATPase VirB11	1.022	0.031090591	0.682537806	0.799487411	no	up	mRNA	32.79	30.48	34.3	29.38	32.57	32.08	32.5233	31.343333	COG0630	U:Intracellular trafficking, secretion, and vesicular transport	GO:0009405;GO:0044097;GO:0044097;GO:0044097;GO:0005737;GO:0043684;GO:0043684;GO:0043684;GO:0005524	BP:pathogenesis;BP:secretion by the type IV secretion system;BP:secretion by the type IV secretion system;BP:secretion by the type IV secretion system;CC:cytoplasm;CC:type IV secretion system complex;CC:type IV secretion system complex;CC:type IV secretion system complex;MF:ATP binding;
BAB_RS22140	-	DNA/RNA nuclease SfsA	1.022	0.03139997	0.699670401	0.811567147	no	up	mRNA	43.54	36.29	41.91	41.16	36.89	39.14	40.58	39.063333	COG1489	S:Function unknown	------	------
BAB_RS23060	-	hypothetical protein	1.022	0.031211824	0.784588158	0.869269569	no	up	mRNA	298.18	313.43	289.92	324.05	316.54	273.86	300.51	304.81667	COG5453	S:Function unknown	------	------
BAB_RS30150	-	sugar ABC transporter substrate-binding protein	1.022	0.031029872	0.811378849	0.885215701	no	up	mRNA	6.32	5.04	5.48	5.12	5.48	5.48	5.61333	5.36	COG1596	S:Function unknown	GO:0016020;GO:0016020;GO:0015159;GO:0015159	CC:membrane;CC:membrane;MF:polysaccharide transmembrane transporter activity;MF:polysaccharide transmembrane transporter activity;
BAB_RS17875	-	Hsp70 family protein	1.022	0.030937115	0.813165918	0.885962053	no	up	mRNA	6.56	6.96	6.52	6.94	5.92	6.52	6.68	6.46	COG0443	O:Posttranslational modification, protein turnover, chaperones	------	------
BAB_RS20565	-	oxidoreductase	1.021	0.029904685	0.582218023	0.722544036	no	up	mRNA	539.62	486.31	517.26	486.65	489.21	508.39	514.397	494.75	COG2084	I:Lipid transport and metabolism	GO:0051287;GO:0004616;GO:0008442;GO:0051287;GO:0004616;GO:0004616	MF:NAD binding;MF:phosphogluconate dehydrogenas;MF:3-hydroxyisobutyrate dehydrogenase activity;MF:NAD binding;MF:phosphogluconate dehydrogenas;MF:phosphogluconate dehydrogenas;
BAB_RS15960	-	glyoxylate/hydroxypyruvate reductase A	1.021	0.030373114	0.55852878	0.723856857	no	up	mRNA	105.42	100.7	111.88	96.11	105.86	103.79	106	101.92	COG0111	E:Amino acid transport and metabolism	GO:0055114;GO:0051287	BP:oxidation-reduction process;MF:NAD binding;
BAB_RS30370	-	cystathionine gamma-synthase	1.021	0.029768509	0.589850238	0.727799083	no	up	mRNA	117.23	106.55	117.43	107.73	108.54	112.2	113.737	109.49	COG3258	C:Energy production and conversion	GO:0046872;GO:0020037;GO:0009055;GO:0009055;GO:0020037;GO:0046872;GO:0009055;GO:0020037	MF:metal ion binding;MF:heme binding;MF:electron carrier activity;MF:electron carrier activity;MF:heme binding;MF:metal ion binding;MF:electron carrier activity;MF:heme binding;
BAB_RS28500	-	lysine--tRNA ligase	1.021	0.030005831	0.629820306	0.754235051	no	up	mRNA	37.27	34.66	36.72	36.67	33.73	33.92	36.2167	34.773333	COG1384	J:Translation, ribosomal structure and biogenesis	GO:0006430;GO:0006430;GO:0005737;GO:0005737;GO:0004824;GO:0000049;GO:0005524;GO:0016491;GO:0005524;GO:0004824;GO:0000049	BP:lysyl-tRNA aminoacylation;BP:lysyl-tRNA aminoacylation;CC:cytoplasm;CC:cytoplasm;MF:lysine-tRNA ligase activity;MF:tRNA binding;MF:ATP binding;MF:oxidoreductase activity;MF:ATP binding;MF:lysine-tRNA ligase activity;MF:tRNA binding;
BAB_RS31250	-	IclR family transcriptional regulator	1.021	0.029311882	0.642916106	0.765921189	no	up	mRNA	82.11	78.93	88.51	79.95	83.03	77.77	83.1833	80.25	COG1414	S:Function unknown	GO:0006355;GO:0006351;GO:0006355;GO:0006351;GO:0003677;GO:0003677	BP:regulation of transcription, DNA-templated;BP:transcription, DNA-templated;BP:regulation of transcription, DNA-templated;BP:transcription, DNA-templated;MF:DNA binding;MF:DNA binding;
BAB_RS21805	-	30S ribosomal protein S11	1.021	0.029814328	0.693820496	0.806820576	no	up	mRNA	585.3	510.45	560.28	593.71	501.29	514.29	552.01	536.43	COG0100	J:Translation, ribosomal structure and biogenesis	GO:0006412;GO:0006412;GO:0006412;GO:0005840;GO:0005840;GO:0005840;GO:0019843;GO:0003735;GO:0003735;GO:0019843;GO:0003735;GO:0019843	BP:translation;BP:translation;BP:translation;CC:ribosome;CC:ribosome;CC:ribosome;MF:rRNA binding;MF:structural constituent of ribosome;MF:structural constituent of ribosome;MF:rRNA binding;MF:structural constituent of ribosome;MF:rRNA binding;
BAB_RS22500	-	urease subunit beta	1.021	0.029547839	0.782402039	0.868944954	no	up	mRNA	100.65	91.03	92.92	77.64	93.51	105.41	94.8667	92.186667	COG0832	S:Function unknown	GO:0043419;GO:0043419;GO:0005737;GO:0005737;GO:0009039;GO:0009039	BP:urea catabolic process;BP:urea catabolic process;CC:cytoplasm;CC:cytoplasm;MF:urease activity;MF:urease activity;
BAB_RS27620	-	NAD(P) transhydrogenase subunit alpha	1.021	0.029365807	0.807159085	0.882109572	no	up	mRNA	158.27	117.29	106.58	117.05	130.47	121.07	127.38	122.86333	COG3288	C:Energy production and conversion	GO:0016021;GO:0016021;GO:0016021;GO:0008750;GO:0008750;GO:0003957	CC:integral component of membrane;CC:integral component of membrane;CC:integral component of membrane;MF:NA;MF:NA;MF:NA;
BAB_RS28770	-	glutamine synthetase	1.021	0.030665244	0.855539665	0.912026855	no	up	mRNA	5.3	3.72	3.92	4.15	4.13	3.97	4.31333	4.0833333	COG0174	C:Energy production and conversion	GO:0006542;GO:0004356	BP:glutamine biosynthetic process;MF:glutamate-ammonia ligase activity;
BAB_RS26955	-	flagellar motor switch protein FliM	1.021	0.030149951	0.937814042	0.959005894	no	up	mRNA	2.46	1.91	2.14	1.08	2.28	2.88	2.17	2.08	COG1868	N:Cell motility	------	------
BAB_RS25995	-	M48 family peptidase	1.02	0.029116299	0.648490331	0.769707955	no	up	mRNA	312.58	317.04	357.23	294.7	331.39	326.36	328.95	317.48333	COG1451	S:Function unknown	------	------
BAB_RS25325	-	electron transfer flavoprotein subunit beta/FixA family protein	1.02	0.028801985	0.652458163	0.772989704	no	up	mRNA	218.14	211.58	226.49	194.57	226.9	211.18	218.737	210.88333	COG2086	C:Energy production and conversion	GO:0009055	MF:electron carrier activity;
BAB_RS17735	-	xanthine dehydrogenase small subunit	1.02	0.028346777	0.684428668	0.800534878	no	up	mRNA	49.06	49.26	54.9	52.3	48.69	46.35	51.0733	49.113333	COG4630	F:Nucleotide transport and metabolism	GO:0051537;GO:0009055;GO:0050660;GO:0046872;GO:0016614;GO:0004854;GO:0004855;GO:0051537;GO:0004662;GO:0009055;GO:0050660;GO:0046872;GO:0016614;GO:0004854;GO:0004855;GO:0050660;GO:0016614;GO:0051537;GO:0004855;GO:0004854;GO:0046872;GO:0009055	MF:2 iron, 2 sulfur cluster binding;MF:electron carrier activity;MF:flavin adenine dinucleotide binding;MF:metal ion binding;MF:oxidoreductase activity, acting on CH-OH group of donors;MF:xanthine dehydrogenase activity;MF:xanthine oxidase activity;MF:2 iron, 2 sulfur cluster binding;MF:CAAX-protein geranylgeranyltransferase activity;MF:electron carrier activity;MF:flavin adenine dinucleotide binding;MF:metal ion binding;MF:oxidoreductase activity, acting on CH-OH group of donors;MF:xanthine dehydrogenase activity;MF:xanthine oxidase activity;MF:flavin adenine dinucleotide binding;MF:oxidoreductase activity, acting on CH-OH group of donors;MF:2 iron, 2 sulfur cluster binding;MF:xanthine oxidase activity;MF:xanthine
BAB_RS30305	-	phosphoketolase	1.02	0.028121404	0.694731501	0.807587667	no	up	mRNA	97.37	93.19	89.99	83.87	89.69	96.03	93.5167	89.863333	COG3957	G:Carbohydrate transport and metabolism	GO:0005975;GO:0016832	BP:carbohydrate metabolic process;MF:aldehyde-lyase activity;
BAB_RS20000	-	inositol monophosphatase	1.02	0.028620073	0.713427604	0.821003901	no	up	mRNA	41.55	35.88	39.16	36.09	36.62	39.36	38.8633	37.356667	COG0483	G:Carbohydrate transport and metabolism	GO:0046854	BP:phosphatidylinositol phosphorylation;
BAB_RS17840	-	ArsC family reductase	1.02	0.029136187	0.759993543	0.85559189	no	up	mRNA	103.62	90.05	82.68	82.37	94.66	89.08	92.1167	88.703333	COG1393	P:Inorganic ion transport and metabolism	GO:0016491	MF:oxidoreductase activity;
BAB_RS29180	-	3-oxoadipate enol-lactonase	1.02	0.028169825	0.762237582	0.856917198	no	up	mRNA	57.78	56.27	57.75	47.46	59.72	58.44	57.2667	55.206667	COG0596	S:Function unknown	GO:0042952;GO:0042952;GO:0016757;GO:0047570;GO:0016757	BP:beta-ketoadipate pathway;BP:beta-ketoadipate pathway;MF:transferase activity, transferring glycosyl groups;MF:3-oxoadipate enol-lactonase activity;MF:3-oxoadipate enol-lactonase activity;MF:transferase activity, transferring glycosyl groups;
BAB_RS25430	-	YggT family protein	1.02	0.028733793	0.778550697	0.8670653	no	up	mRNA	76.54	57.41	67.39	71.88	61.65	59.33	67.1133	64.286667	COG0762	S:Function unknown	GO:0016021;GO:0016021;GO:0016020	CC:integral component of membrane;CC:integral component of membrane;CC:membrane;
BAB_RS22980	-	hybrid-cluster NAD(P)-dependent oxidoreductase	1.02	0.02830264	0.783095139	0.868813771	no	up	mRNA	10.51	11.11	12.63	10.99	11.13	10.95	11.4167	11.023333	COG1018	P:Inorganic ion transport and metabolism	GO:0051537;GO:0009055;GO:0046872;GO:0046872;GO:0051213;GO:0009055;GO:0051537	MF:2 iron, 2 sulfur cluster binding;MF:electron carrier activity;MF:metal ion binding;MF:metal ion binding;MF:dioxygenase activity;MF:electron carrier activity;MF:2 iron, 2 sulfur cluster binding;
BAB_RS30095	-	acetyl-CoA C-acyltransferase	1.02	0.029083888	0.874652657	0.922606365	no	up	mRNA	3.44	3.83	4.28	3.91	4.12	3.12	3.85	3.7166667	COG0183	S:Function unknown	GO:0008152;GO:0008152;GO:0003985;GO:0016301;GO:0016747;GO:0016747	BP:metabolic process;BP:metabolic process;MF:acetyl-CoA C-acetyltransferase activity;MF:kinase activity;MF:transferase activity, transferring acyl groups other than amino-acyl groups;MF:transferase activity, transferring acyl groups other than amino-acyl groups;
BAB_RS30660	-	pseudoazurin	1.02	0.02788672	0.887252385	0.928882996	no	up	mRNA	256.39	282.84	199.44	169.57	309.37	238.42	246.223	239.12	COG3794	C:Energy production and conversion	GO:0005507;GO:0009055	MF:copper ion binding;MF:electron carrier activity;
BAB_RS16085	-	DNA polymerase III subunit gamma/tau	1.019	0.027406786	0.582767584	0.722946813	no	up	mRNA	156.45	153.42	167.55	148.22	157.36	152.58	159.14	152.72	COG2812	L:Replication, recombination and repair	GO:0006260;GO:0006260;GO:0009360;GO:0009360;GO:0003887;GO:0003677;GO:0005524;GO:0016491;GO:0005524;GO:0003677;GO:0003887	BP:DNA replication;BP:DNA replication;CC:DNA polymerase III complex;CC:DNA polymerase III complex;MF:DNA-directed DNA polymerase activity;MF:DNA binding;MF:ATP binding;MF:oxidoreductase activity;MF:ATP binding;MF:DNA binding;MF:DNA-directed DNA polymerase activity;
BAB_RS22665	-	PDZ domain-containing protein	1.019	0.027417379	0.646850262	0.768613126	no	up	mRNA	203.38	180.06	184.98	181.45	185.68	178.58	189.473	181.90333	COG0265	E:Amino acid transport and metabolism	GO:0042597;GO:0004252	CC:periplasmic space;MF:serine-type endopeptidase activity;
BAB_RS21265	-	tRNA guanosine(34) transglycosylase Tgt	1.019	0.026859657	0.693543427	0.80679038	no	up	mRNA	36.9	34.98	39.28	38.03	34.52	34.09	37.0533	35.546667	COG0343	J:Translation, ribosomal structure and biogenesis	GO:0008616;GO:0008616;GO:0101030;GO:0016491;GO:0046872;GO:0008479;GO:0101030;GO:0046872;GO:0016491;GO:0008479	BP:queuosine biosynthetic process;BP:queuosine biosynthetic process;BP:old GO;MF:oxidoreductase activity;MF:metal ion binding;MF:queuine tRNA-ribosyltransferase activity;MF:old GO;MF:metal ion binding;MF:oxidoreductase activity;MF:queuine tRNA-ribosyltransferase activity;
BAB_RS30380	-	chloride channel protein	1.019	0.027330679	0.708433611	0.817599566	no	up	mRNA	38.07	37.07	36.43	35.42	34.65	37.19	37.19	35.753333	COG0038	P:Inorganic ion transport and metabolism	GO:0016021;GO:0005886;GO:0005247	CC:integral component of membrane;CC:plasma membrane;MF:voltage-gated chloride channel activity;
BAB_RS29915	-	glycosyl hydrolase	1.019	0.027748675	0.76551536	0.859099144	no	up	mRNA	7.65	6.73	8.03	6.71	7.46	7.27	7.47	7.1466667	COG3757	E:Amino acid transport and metabolism	GO:0016998;GO:0009253;GO:0003796	BP:cell wall macromolecule catabolic process;BP:peptidoglycan catabolic process;MF:lysozyme activity;
BAB_RS26400	-	ABC transporter permease	1.019	0.027713806	0.773768348	0.864435919	no	up	mRNA	21.51	19.98	20.83	17.93	20.53	21.41	20.7733	19.956667	COG4603	S:Function unknown	GO:0008643;GO:0016021;GO:0005886;GO:0016021;GO:0005886;GO:0022857;GO:0022857	BP:carbohydrate transport;CC:integral component of membrane;CC:plasma membrane;CC:integral component of membrane;CC:plasma membrane;MF:transmembrane transporter activity;MF:transmembrane transporter activity;
BAB_RS31200	-	hypothetical protein	1.019	0.02659337	0.802010416	0.879174161	no	up	mRNA	105.6	125.01	113.97	110.22	114.55	116.61	114.86	113.79333	ENOG410Y2TH;ENOG4112DBH	S:Function unknown;	GO:0016021	CC:integral component of membrane;
BAB_RS16010	-	hydroxymethylglutaryl-CoA lyase	1.019	0.02694122	0.812297502	0.885616517	no	up	mRNA	27.29	24.37	31.71	29.65	26.58	24.08	27.79	26.77	COG0119	E:Amino acid transport and metabolism	GO:0005829;GO:0005829;GO:0004419;GO:0004419	CC:cytosol;CC:cytosol;MF:hydroxymethylglutaryl-CoA lyase activity;MF:hydroxymethylglutaryl-CoA lyase activity;
BAB_RS27960	-	transcriptional regulator	1.019	0.0269422	0.859973472	0.915235099	no	up	mRNA	37.99	38.57	29.87	33.54	34.53	35.72	35.4767	34.596667	COG0640;ENOG4111VSN	K:Transcription;	GO:0003700	MF:transcription factor activity, sequence-specific DNA binding;
BAB_RS28465	-	hypothetical protein	1.019	0.026699998	0.878210516	0.925144389	no	up	mRNA	38.9	41.37	35.18	29.12	45.64	37.39	38.4833	37.383333	------	------	------	------
BAB_RS28095	-	hypothetical protein	1.019	0.027817575	0.916334358	0.947596489	no	up	mRNA	232.06	177.75	225.72	199.18	245.24	244.8	211.843	229.74	------	------	------	------
BAB_RS24970	-	PLP-dependent aminotransferase family protein	1.018	0.025283935	0.767781707	0.861041056	no	up	mRNA	20.84	18.64	24.94	20.53	19.23	21.88	21.4733	20.546667	COG1167	S:Function unknown	GO:0006351;GO:0006351;GO:0008483;GO:0003700;GO:0003824;GO:0003677;GO:0030170;GO:0003824;GO:0003677;GO:0003700;GO:0030170	BP:transcription, DNA-templated;BP:transcription, DNA-templated;MF:transaminase activity;MF:transcription factor activity, sequence-specific DNA binding;MF:catalytic activity;MF:DNA binding;MF:pyridoxal phosphate binding;MF:catalytic activity;MF:DNA binding;MF:transcription factor activity, sequence-specific DNA binding;MF:pyridoxal phosphate binding;
BAB_RS21145	-	hypothetical protein	1.017	0.024708095	0.697705347	0.809580093	no	up	mRNA	101.27	107.28	107.61	99.16	99.65	104.93	105.387	101.24667	ENOG410Z410	S:Function unknown	------	------
BAB_RS25600	-	site-specific tyrosine recombinase XerD	1.017	0.02501001	0.717366501	0.824945801	no	up	mRNA	47.8	43.62	48.1	46.75	43.2	43.94	46.5067	44.63	COG4974	S:Function unknown	GO:0007049;GO:0051301;GO:0007059;GO:0015074;GO:0007049;GO:0051301;GO:0007059;GO:0006313;GO:0006313;GO:0007049;GO:0007059;GO:0051301;GO:0005737;GO:0005737;GO:0005737;GO:0003677;GO:0009009;GO:0003677;GO:0009037;GO:0009037;GO:0003677	BP:cell cycle;BP:cell division;BP:chromosome segregation;BP:DNA integration;BP:cell cycle;BP:cell division;BP:chromosome segregation;BP:transposition, DNA-mediated;BP:transposition, DNA-mediated;BP:cell cycle;BP:chromosome segregation;BP:cell division;CC:cytoplasm;CC:cytoplasm;CC:cytoplasm;MF:DNA binding;MF:site-specific recombinase activity;MF:DNA binding;MF:tyrosine-based site-specific recombinase activity;MF:tyrosine-based site-specific recombinase activity;MF:DNA binding;
BAB_RS20790	-	TatD family deoxyribonuclease	1.017	0.02459082	0.827101142	0.894471884	no	up	mRNA	61.91	49.93	64.27	65.47	51.42	52.17	58.7033	56.353333	COG0084	L:Replication, recombination and repair	GO:0016888	MF:endodeoxyribonuclease activity, producing 5'-phosphomonoesters;
BAB_RS29485	-	FosX/FosE/FosI family fosfomycin resistance thiol transferase	1.017	0.024856802	0.836545612	0.901348441	no	up	mRNA	19.43	16.07	24.55	18.07	20.32	19.13	20.0167	19.173333	ENOG4111SM5	S:Function unknown	GO:0016740	MF:transferase activity;
BAB_RS24400	-	RNA-binding S4 domain-containing protein	1.017	0.024710997	0.853684895	0.911258993	no	up	mRNA	62.95	48.83	52.88	58.03	54.49	46.97	54.8867	53.163333	COG1188	J:Translation, ribosomal structure and biogenesis	GO:0003723	MF:RNA binding;
BAB_RS20095	-	beta-ketoacyl-ACP synthase II	1.016	0.02338778	0.705596494	0.816084066	no	up	mRNA	86.68	78.65	85.47	81.92	75.83	82.5	83.6	80.083333	COG0304	C:Energy production and conversion	GO:0008152;GO:0008152;GO:0003824;GO:0003824;GO:0004315	BP:metabolic process;BP:metabolic process;MF:catalytic activity;MF:catalytic activity;MF:3-oxoacyl-[acyl-carrier-protein] synthase activity;
BAB_RS18525	-	DegT/DnrJ/EryC1/StrS aminotransferase family protein	1.016	0.022296298	0.709505821	0.818542981	no	up	mRNA	497.11	456.56	469.61	478.96	437.05	448.31	474.427	454.77333	COG0399	E:Amino acid transport and metabolism	GO:0003824;GO:0008483;GO:0003983;GO:0003824	MF:catalytic activity;MF:transaminase activity;MF:transaminase activity;MF:catalytic activity;
BAB_RS20220	-	segregation/condensation protein A	1.016	0.023399309	0.782765309	0.869048009	no	up	mRNA	109.98	107.32	127.62	98.37	111.48	121.88	114.973	110.57667	COG1354	S:Function unknown	GO:0007059;GO:0051301;GO:0007049;GO:0007049;GO:0051301;GO:0007059;GO:0007059;GO:0007049;GO:0051301;GO:0005737;GO:0005737;GO:0005737	BP:chromosome segregation;BP:cell division;BP:cell cycle;BP:cell cycle;BP:cell division;BP:chromosome segregation;BP:chromosome segregation;BP:cell cycle;BP:cell division;CC:cytoplasm;CC:cytoplasm;CC:cytoplasm;
BAB_RS21720	-	hypothetical protein	1.016	0.022274088	0.784155117	0.869089476	no	up	mRNA	454.74	394.36	415.53	352.06	430.58	429.57	421.543	404.07	ENOG410ZGE6	S:Function unknown	------	------
BAB_RS21165	-	iron-sulfur cluster assembly scaffold protein	1.015	0.021139771	0.664031448	0.784387148	no	up	mRNA	146.77	141.56	156.3	137.48	145.48	141.85	148.21	141.60333	COG0822	C:Energy production and conversion	GO:0016226;GO:0005506;GO:0051536	BP:iron-sulfur cluster assembly;MF:iron ion binding;MF:iron-sulfur cluster binding;
BAB_RS21610	-	glycerophosphoryl diester phosphodiesterase	1.015	0.021364638	0.737565381	0.839758175	no	up	mRNA	194.25	175.31	192.79	167.51	180.07	192.16	187.45	179.91333	COG0584	C:Energy production and conversion	GO:0006629;GO:0006629;GO:0008889;GO:0008889	BP:lipid metabolic process;BP:lipid metabolic process;MF:glycerophosphodiester phosphodiesterase activity;MF:glycerophosphodiester phosphodiesterase activity;
BAB_RS18270	-	cytochrome c oxidase subunit I	1.015	0.021708868	0.760880768	0.856290685	no	up	mRNA	417.59	362.28	376.15	340.05	376.44	388.76	385.34	368.41667	COG0843	S:Function unknown	GO:0015990;GO:0009060;GO:0006119;GO:0015990;GO:0006119;GO:0009060;GO:0015990;GO:0009060;GO:0006119;GO:0009060;GO:0015990;GO:0006119;GO:0016021;GO:0045277;GO:0005886;GO:0016021;GO:0005886;GO:0045277;GO:0016021;GO:0045277;GO:0016021;GO:0005886;GO:0045277;GO:0004129;GO:0016787;GO:0020037;GO:0046872;GO:0004129;GO:0020037;GO:0046872;GO:0004129;GO:0020037;GO:0046872;GO:0004129;GO:0020037;GO:0046872	BP:electron transport coupled proton transport;BP:aerobic respiration;BP:oxidative phosphorylation;BP:electron transport coupled proton transport;BP:oxidative phosphorylation;BP:aerobic respiration;BP:electron transport coupled proton transport;BP:aerobic respiration;BP:oxidative phosphorylation;BP:aerobic respiration;BP:electron transport coupled proton transport;BP:oxidative phosphorylation;CC:integral component of membrane;CC:respiratory chain complex IV;CC:plasma membrane;CC:integral component of membrane;CC:plasma membrane;CC:respiratory chain complex IV;CC:integral component of membrane;CC:respiratory chain complex IV;CC:integral component of membrane;CC:plasma membrane;CC:respiratory chain complex IV;MF:cytochrome-c oxidase activity;MF:hydrolase activity;MF:heme binding;MF:metal ion binding;MF:cytochrome-c oxidase activity;MF:heme binding;MF:metal ion binding;MF:cytochrome-c oxidase activity;MF:heme binding;MF:metal ion binding;MF:cytochrome-c oxidase activity;MF:heme binding;MF:metal ion binding;
BAB_RS25435	-	inorganic pyrophosphatase	1.015	0.020783194	0.779926224	0.86769493	no	up	mRNA	576.67	495.41	519.37	559.26	476.1	498.26	530.483	511.20667	COG0221	C:Energy production and conversion	GO:0006796;GO:0006796;GO:0005737;GO:0005737;GO:0004427;GO:0000287;GO:0000287;GO:0004427	BP:phosphate-containing compound metabolic process;BP:phosphate-containing compound metabolic process;CC:cytoplasm;CC:cytoplasm;MF:inorganic diphosphatase activity;MF:magnesium ion binding;MF:magnesium ion binding;MF:inorganic diphosphatase activity;
BAB_RS20005	-	Bcr/CflA family drug resistance efflux transporter	1.015	0.020800622	0.797302826	0.876106012	no	up	mRNA	53.18	45.77	45.85	48.46	45.48	44.18	48.2667	46.04	ENOG410XNNX	P:Inorganic ion transport and metabolism	GO:0016021;GO:0016021;GO:0015238;GO:0015238	CC:integral component of membrane;CC:integral component of membrane;MF:drug transmembrane transporter activity;MF:drug transmembrane transporter activity;
BAB_RS22275	-	cobaltochelatase subunit CobN	1.014	0.019466108	0.76881524	0.861598663	no	up	mRNA	62.02	68.65	73.84	63.47	64.21	67.5	68.17	65.06	COG1429	H:Coenzyme transport and metabolism	GO:0009236;GO:0009236;GO:0051116;GO:0047519;GO:0051116	BP:cobalamin biosynthetic process;BP:cobalamin biosynthetic process;MF:cobaltochelatase activity;MF:quinate dehydrogenas;MF:cobaltochelatase activity;
BAB_RS21565	-	phosphatidate cytidylyltransferase	1.014	0.019390059	0.772888639	0.86375346	no	up	mRNA	65.76	62.38	70.29	60.03	67.15	62.66	66.1433	63.28	COG0575	I:Lipid transport and metabolism	GO:0016024;GO:0016024;GO:0016021;GO:0005886;GO:0016021;GO:0004605;GO:0004605	BP:CDP-diacylglycerol biosynthetic process;BP:CDP-diacylglycerol biosynthetic process;CC:integral component of membrane;CC:plasma membrane;CC:integral component of membrane;MF:phosphatidate cytidylyltransferase activity;MF:phosphatidate cytidylyltransferase activity;
BAB_RS23585	-	DUF1153 domain-containing protein	1.014	0.020423643	0.783185276	0.868613839	no	up	mRNA	5669.18	5940.29	5337.63	5512.37	5303.31	6098.72	5649.03	5638.1333	ENOG4111NMJ	S:Function unknown	GO:0043565	MF:sequence-specific DNA binding;
BAB_RS29400	-	Asp-tRNA(Asn)/Glu-tRNA(Gln) amidotransferase GatCAB subunit C	1.014	0.019445061	0.797617255	0.875852441	no	up	mRNA	292.58	264.63	278.72	293.6	259.64	260.72	278.643	271.32	COG0721	J:Translation, ribosomal structure and biogenesis	GO:0006450;GO:0006412;GO:0006450;GO:0006412;GO:0006450;GO:0006412;GO:0016787;GO:0016740;GO:0050567;GO:0016874;GO:0005524;GO:0005524;GO:0016874;GO:0005524;GO:0050567;GO:0016740	BP:regulation of translational fidelity;BP:translation;BP:regulation of translational fidelity;BP:translation;BP:regulation of translational fidelity;BP:translation;MF:hydrolase activity;MF:transferase activity;MF:glutaminyl-tRNA synthas;MF:ligase activity;MF:ATP binding;MF:ATP binding;MF:ligase activity;MF:ATP binding;MF:glutaminyl-tRNA synthas;MF:transferase activity;
BAB_RS33550	-	heavy metal translocating P-type ATPase	1.014	0.019507305	0.834860275	0.900438424	no	up	mRNA	13.67	14.17	17.05	13.98	15.42	13.51	14.9633	14.303333	COG2217	E:Amino acid transport and metabolism	GO:0016021;GO:0005886;GO:0016021;GO:0005886;GO:0019829;GO:0047381;GO:0000166;GO:0019829;GO:0000166	CC:integral component of membrane;CC:plasma membrane;CC:integral component of membrane;CC:plasma membrane;MF:cation-transporting ATPase activity;MF:dodecanoyl-[acyl-carrier-protein] hydrolase activity;MF:nucleotide binding;MF:cation-transporting ATPase activity;MF:nucleotide binding;
BAB_RS30180	-	putative N-acetylmannosamine-6-phosphate 2-epimerase	1.014	0.019726308	0.936814229	0.95889905	no	up	mRNA	2.71	1.45	2.92	1.88	2.54	2.23	2.36	2.2166667	COG3010	G:Carbohydrate transport and metabolism	GO:0005975;GO:0006051;GO:0019262;GO:0019262;GO:0005975;GO:0006051;GO:0047465;GO:0047465;GO:0016301	BP:carbohydrate metabolic process;BP:N-acetylmannosamine metabolic process;BP:N-acetylneuraminate catabolic process;BP:N-acetylneuraminate catabolic process;BP:carbohydrate metabolic process;BP:N-acetylmannosamine metabolic process;MF:N-acylglucosamine-6-phosphate 2-epimerase activity;MF:N-acylglucosamine-6-phosphate 2-epimerase activity;MF:kinase activity;
BAB_RS17500	-	two-component sensor histidine kinase	1.013	0.019182166	0.748614581	0.847532998	no	up	mRNA	30.37	29.05	33.54	29.33	29.92	29.4	30.9867	29.55	ENOG410XNMH	T:Signal transduction mechanisms	GO:0016021;GO:0005622;GO:0016021;GO:0005622;GO:0005524;GO:0000155;GO:0000155;GO:0005524	CC:integral component of membrane;CC:intracellular;CC:integral component of membrane;CC:intracellular;MF:ATP binding;MF:phosphorelay sensor kinase activity;MF:phosphorelay sensor kinase activity;MF:ATP binding;
BAB_RS19360	-	CDP-diacylglycerol--glycerol-3-phosphate 3-phosphatidyltransferase	1.013	0.01858038	0.764953616	0.858768682	no	up	mRNA	280.29	235.42	264.05	240.52	251.79	256.53	259.92	249.61333	COG0558	I:Lipid transport and metabolism	GO:0008654;GO:0008654;GO:0016021;GO:0016021;GO:0008444;GO:0008444	BP:phospholipid biosynthetic process;BP:phospholipid biosynthetic process;CC:integral component of membrane;CC:integral component of membrane;MF:CDP-diacylglycerol-glycerol-3-phosphate 3-phosphatidyltransferase activity;MF:CDP-diacylglycerol-glycerol-3-phosphate 3-phosphatidyltransferase activity;
BAB_RS24430	-	polyprenyl synthetase family protein	1.013	0.019129017	0.770006358	0.862331972	no	up	mRNA	37.43	36	40.96	38.78	34.8	35.52	38.13	36.366667	COG0142	J:Translation, ribosomal structure and biogenesis	GO:0008299;GO:0008299;GO:0016740;GO:0004337;GO:0016740	BP:isoprenoid biosynthetic process;BP:isoprenoid biosynthetic process;MF:transferase activity;MF:geranyltranstransferase activity;MF:transferase activity;
BAB_RS27775	-	dihydroxy-acid dehydratase	1.013	0.018511883	0.778792285	0.867033823	no	up	mRNA	252.27	215.39	239.53	208.27	240.41	225.22	235.73	224.63333	COG0129	S:Function unknown	GO:0008152;GO:0008152;GO:0008152;GO:0016829;GO:0016829;GO:0004160;GO:0016829;GO:0008869	BP:metabolic process;BP:metabolic process;BP:metabolic process;MF:lyase activity;MF:lyase activity;MF:dihydroxy-acid dehydratase activity;MF:lyase activity;MF:galactonate dehydratase activity;
BAB_RS29900	-	ketoacyl-ACP synthase III	1.013	0.018068014	0.868033896	0.917733764	no	up	mRNA	19.33	23.66	22.27	21.02	21.2	20.58	21.7533	20.933333	COG0332	H:Coenzyme transport and metabolism	GO:0006633;GO:0006633;GO:0004315;GO:0004315	BP:fatty acid biosynthetic process;BP:fatty acid biosynthetic process;MF:3-oxoacyl-[acyl-carrier-protein] synthase activity;MF:3-oxoacyl-[acyl-carrier-protein] synthase activity;
BAB_RS30225	-	gfo/Idh/MocA family oxidoreductase	1.013	0.019075293	0.899314832	0.936932087	no	up	mRNA	8.13	6.47	8.45	6.5	7.61	7.87	7.68333	7.3266667	ENOG4111K6V	S:Function unknown	GO:0016491	MF:oxidoreductase activity;
BAB_RS26205	-	HlyC/CorC family transporter	1.012	0.016981576	0.750849347	0.848568045	no	up	mRNA	544.14	553.17	610.01	531.3	548.36	550.81	569.107	543.49	COG1253	P:Inorganic ion transport and metabolism	GO:0050660;GO:0016614	MF:flavin adenine dinucleotide binding;MF:oxidoreductase activity, acting on CH-OH group of donors;
BAB_RS29630	-	hypothetical protein	1.012	0.01760559	0.788038493	0.870989913	no	up	mRNA	114.45	113.06	128.46	120.22	106.4	113.23	118.657	113.28333	------	------	------	------
BAB_RS21955	-	DNA-directed RNA polymerase subunit beta'	1.012	0.01702577	0.792469176	0.872585148	no	up	mRNA	901.22	813.37	871.11	789.46	851.96	819.14	861.9	820.18667	COG0086	K:Transcription	GO:0006351;GO:0006351;GO:0003677;GO:0003899;GO:0003899;GO:0016491;GO:0003677	BP:transcription, DNA-templated;BP:transcription, DNA-templated;MF:DNA binding;MF:DNA-directed RNA polymerase activity;MF:DNA-directed RNA polymerase activity;MF:oxidoreductase activity;MF:DNA binding;
BAB_RS19790	-	agmatinase	1.012	0.016853688	0.817157658	0.888804183	no	up	mRNA	59.17	61.3	70.23	58.25	60.77	63.52	63.5667	60.846667	COG0010	E:Amino acid transport and metabolism	GO:0004177;GO:0046872;GO:0016813;GO:0004177;GO:0008783;GO:0046872;GO:0016813	MF:aminopeptidase activity;MF:metal ion binding;MF:hydrolase activity, acting on carbon-nitroge;MF:aminopeptidase activity;MF:agmatinase activity;MF:metal ion binding;MF:hydrolase activity, acting on carbon-nitroge;
BAB_RS23880	-	N-acetyltransferase	1.012	0.017423193	0.839112967	0.90260126	no	up	mRNA	49	44.75	60.85	50.11	50.98	45.97	51.5333	49.02	COG1247	C:Energy production and conversion	GO:0016740;GO:0016740;GO:0046872;GO:0016746	MF:transferase activity;MF:transferase activity;MF:metal ion binding;MF:transferase activity, transferring acyl groups;
BAB_RS24680	-	GlsB/YeaQ/YmgE family stress response membrane protein	1.012	0.01725659	0.914180724	0.947198537	no	up	mRNA	192.25	181.9	165.55	155.08	174.68	220.23	179.9	183.33	------	------	------	------
BAB_RS31315	-	ABC transporter ATP-binding protein	1.012	0.016502263	0.933661767	0.956586498	no	up	mRNA	5.39	5.86	4.47	5.48	4.27	5.3	5.24	5.0166667	COG4608	S:Function unknown	GO:0015031;GO:0015833;GO:0015833;GO:0005886;GO:0005524;GO:0016887;GO:0005524;GO:0016887	BP:protein transport;BP:peptide transport;BP:peptide transport;CC:plasma membrane;MF:ATP binding;MF:ATPase activity;MF:ATP binding;MF:ATPase activity;
BAB_RS29130	-	adenine deaminase	1.011	0.015711942	0.783378838	0.868528711	no	up	mRNA	78.25	72.9	74.83	70.07	70.94	74.02	75.3267	71.676667	COG1001	F:Nucleotide transport and metabolism	GO:0006146;GO:0000034	BP:adenine catabolic process;MF:adenine deaminase activity;
BAB_RS16640	-	integration host factor subunit beta	1.011	0.015178192	0.838505715	0.902552382	no	up	mRNA	761.65	682.79	672.99	659.84	695.98	753.74	705.81	703.18667	COG0776	L:Replication, recombination and repair	GO:0006310;GO:0006355;GO:0006351;GO:0006417;GO:0006310;GO:0006355;GO:0006417;GO:0006351;GO:0005694;GO:0005694;GO:0003677;GO:0003677	BP:DNA recombination;BP:regulation of transcription, DNA-templated;BP:transcription, DNA-templated;BP:regulation of translation;BP:DNA recombination;BP:regulation of transcription, DNA-templated;BP:regulation of translation;BP:transcription, DNA-templated;CC:chromosome;CC:chromosome;MF:DNA binding;MF:DNA binding;
BAB_RS30110	-	transcriptional regulator	1.011	0.016188747	0.852880234	0.911005383	no	up	mRNA	30.31	27.62	28.15	28.67	27.26	26.08	28.6933	27.336667	ENOG410XR9S	K:Transcription	GO:0006351;GO:0006351;GO:0003700;GO:0003677;GO:0003677;GO:0003700	BP:transcription, DNA-templated;BP:transcription, DNA-templated;MF:transcription factor activity, sequence-specific DNA binding;MF:DNA binding;MF:DNA binding;MF:transcription factor activity, sequence-specific DNA binding;
BAB_RS27240	hisG	ATP phosphoribosyltransferase catalytic subunit HisG	1.011	0.015243823	0.879035722	0.924800843	no	up	mRNA	41.83	33.11	39.36	38.22	34.3	36.51	38.1	36.343333	COG0040	E:Amino acid transport and metabolism	GO:0000105;GO:0000105;GO:0000105;GO:0005737;GO:0005737;GO:0005737;GO:0005524;GO:0003879;GO:0003879;GO:0016829;GO:0005524;GO:0003879;GO:0005524	BP:histidine biosynthetic process;BP:histidine biosynthetic process;BP:histidine biosynthetic process;CC:cytoplasm;CC:cytoplasm;CC:cytoplasm;MF:ATP binding;MF:ATP phosphoribosyltransferase activity;MF:ATP phosphoribosyltransferase activity;MF:lyase activity;MF:ATP binding;MF:ATP phosphoribosyltransferase activity;MF:ATP binding;
BAB_RS31510	-	hypothetical protein	1.011	0.015151714	0.890385369	0.930949623	no	up	mRNA	10.26	11.45	12.1	11.3	10.44	10.68	11.27	10.806667	ENOG41124RZ	S:Function unknown	GO:0016021	CC:integral component of membrane;
BAB_RS29590	-	hypothetical protein	1.011	0.015625209	0.901705182	0.93911791	no	up	mRNA	22.43	18.71	20.33	19.57	18.52	20.71	20.49	19.6	------	------	------	------
BAB_RS19775	-	DUF2842 domain-containing protein	1.011	0.016051149	0.906235178	0.942003762	no	up	mRNA	16.16	12.94	16.1	13.46	15.41	14.2	15.0667	14.356667	ENOG41123RH	S:Function unknown	GO:0016021	CC:integral component of membrane;
BAB_RS21795	-	50S ribosomal protein L17	1.01	0.01494816	0.796039316	0.875615996	no	up	mRNA	822.69	786.87	859.23	806.04	809.26	777.73	822.93	797.67667	COG0203	J:Translation, ribosomal structure and biogenesis	GO:0006412;GO:0005840;GO:0003735	BP:translation;CC:ribosome;MF:structural constituent of ribosome;
BAB_RS29845	-	monovalent cation/H+ antiporter subunit D	1.01	0.013992037	0.814382134	0.886986372	no	up	mRNA	64.61	66.76	71.53	63.74	65.7	63.76	67.6333	64.4	COG0651;COG0651	C:Energy production and conversion;P:Inorganic ion transport and metabolism	GO:0016021	CC:integral component of membrane;
BAB_RS17470	-	efflux RND transporter periplasmic adaptor subunit	1.01	0.014657837	0.852249313	0.91093714	no	up	mRNA	103.5	87.36	89.82	94.43	84.37	88.19	93.56	88.996667	ENOG410XNVN	V:Defense mechanisms	GO:0055085;GO:0016020;GO:0005215	BP:transmembrane transport;CC:membrane;MF:transporter activity;
BAB_RS20045	-	phosphoribosylaminoimidazolesuccinocarboxamide synthase	1.01	0.014368714	0.864218784	0.917019469	no	up	mRNA	546.2	447.63	454.68	494.81	457.85	428	482.837	460.22	COG0152	F:Nucleotide transport and metabolism	GO:0009236;GO:0006189;GO:0009236;GO:0006189;GO:0004639;GO:0005524;GO:0016746;GO:0004639;GO:0005524	BP:cobalamin biosynthetic process;BP:'de novo' IMP biosynthetic process;BP:cobalamin biosynthetic process;BP:'de novo' IMP biosynthetic process;MF:phosphoribosylaminoimidazolesuccinocarboxamide synthase activity;MF:ATP binding;MF:transferase activity, transferring acyl groups;MF:phosphoribosylaminoimidazolesuccinocarboxamide synthase activity;MF:ATP binding;
BAB_RS27425	-	two-component sensor histidine kinase	1.01	0.014356318	0.921442174	0.952571977	no	up	mRNA	25.48	31.5	26.87	23.29	32.57	24.15	27.95	26.67	ENOG410XS4R	T:Signal transduction mechanisms	GO:0016021;GO:0005622;GO:0000155;GO:0005524	CC:integral component of membrane;CC:intracellular;MF:phosphorelay sensor kinase activity;MF:ATP binding;
BAB_RS17180	-	hypothetical protein	1.01	0.01463403	0.969639907	0.980627328	no	up	mRNA	18.83	7.85	7.36	10.8	9.28	10.98	11.3467	10.353333	------	------	------	------
BAB_RS27530	-	protein-methionine-sulfoxide reductase heme-binding subunit YedZ	1.009	0.013245266	0.818704912	0.88958366	no	up	mRNA	145.19	135.98	142.54	137.8	130.41	135.85	141.237	134.68667	COG2717	P:Inorganic ion transport and metabolism	GO:0030091;GO:0030091;GO:0055114;GO:0005887;GO:0005887;GO:0009055;GO:0020037;GO:0010181;GO:0046872;GO:0020037;GO:0010181;GO:0046872;GO:0009055	BP:protein repair;BP:protein repair;BP:oxidation-reduction process;CC:integral component of plasma membrane;CC:integral component of plasma membrane;MF:electron carrier activity;MF:heme binding;MF:FMN binding;MF:metal ion binding;MF:heme binding;MF:FMN binding;MF:metal ion binding;MF:electron carrier activity;
BAB_RS22790	-	DNA ligase (NAD(+)) LigA	1.009	0.013598236	0.824562654	0.89292882	no	up	mRNA	49.46	46.71	54.87	45.52	50.54	47.27	50.3467	47.776667	COG0272	L:Replication, recombination and repair	GO:0006281;GO:0006260;GO:0006260;GO:0006281;GO:0006260;GO:0006281;GO:0003911;GO:0046872;GO:0016491;GO:0046872;GO:0003911;GO:0016491;GO:0046872;GO:0003911	BP:DNA repair;BP:DNA replication;BP:DNA replication;BP:DNA repair;BP:DNA replication;BP:DNA repair;MF:DNA ligas;MF:metal ion binding;MF:oxidoreductase activity;MF:metal ion binding;MF:DNA ligas;MF:oxidoreductase activity;MF:metal ion binding;MF:DNA ligas;
BAB_RS22025	-	rhomboid family intramembrane serine protease	1.009	0.013099036	0.847300126	0.908366802	no	up	mRNA	107.1	93.95	104.75	97.02	103.4	90.25	101.933	96.89	COG0705	S:Function unknown	GO:0016021;GO:0004252	CC:integral component of membrane;MF:serine-type endopeptidase activity;

BAB_RS20235	-	twin-arginine translocase subunit TatB	1.009	0.01306729	0.855668909	0.911862092	no	up	mRNA	377.96	302.44	325.92	320.93	331.1	308.89	335.44	320.30667	COG1826	U:Intracellular trafficking, secretion, and vesicular transport	GO:0009306;GO:0043953;GO:0005887;GO:0033281;GO:0008320	BP:protein secretion;BP:protein transport by the Tat complex;CC:integral component of plasma membrane;CC:TAT protein transport complex;MF:protein transmembrane transporter activity;
BAB_RS25585	-	3-dehydroquinate synthase	1.009	0.013430876	0.864242364	0.916741736	no	up	mRNA	36.16	39.32	38.8	37.52	36.92	34.59	38.0933	36.343333	COG0337	E:Amino acid transport and metabolism	GO:0009423;GO:0009073;GO:0009423;GO:0009073;GO:0009073;GO:0009423;GO:0005737;GO:0005737;GO:0005737;GO:0000166;GO:0003856;GO:0046872;GO:0016301;GO:0000166;GO:0016491;GO:0003856;GO:0046872;GO:0003856;GO:0046872;GO:0000166	BP:chorismate biosynthetic process;BP:aromatic amino acid family biosynthetic process;BP:chorismate biosynthetic process;BP:aromatic amino acid family biosynthetic process;BP:aromatic amino acid family biosynthetic process;BP:chorismate biosynthetic process;CC:cytoplasm;CC:cytoplasm;CC:cytoplasm;MF:nucleotide binding;MF:3-dehydroquinate synthase activity;MF:metal ion binding;MF:kinase activity;MF:nucleotide binding;MF:oxidoreductase activity;MF:3-dehydroquinate synthase activity;MF:metal ion binding;MF:3-dehydroquinate synthase activity;MF:metal ion binding;MF:nucleotide binding;
BAB_RS22555	-	cobalt ABC transporter ATP-binding protein	1.009	0.012592342	0.867651673	0.918236108	no	up	mRNA	26.7	28.26	31.95	26.88	29.39	26.35	28.97	27.54	COG1122	P:Inorganic ion transport and metabolism	GO:0005886;GO:0005524;GO:0016887;GO:0005524;GO:0016887	CC:plasma membrane;MF:ATP binding;MF:ATPase activity;MF:ATP binding;MF:ATPase activity;
BAB_RS23595	-	hypothetical protein	1.009	0.012359482	0.868991517	0.918443994	no	up	mRNA	65.93	65.76	68.36	69.17	58.47	62.74	66.6833	63.46	ENOG4111X3Z	S:Function unknown	GO:0016021	CC:integral component of membrane;
BAB_RS32805	-	hypothetical protein	1.009	0.012507392	0.929531357	0.955401232	no	up	mRNA	4.94	3.97	4.85	4.93	4.05	4.03	4.58667	4.3366667	------	------	------	------
BAB_RS18005	-	hypothetical protein	1.009	0.012802324	0.930432877	0.956022013	no	up	mRNA	10.94	12.42	11.3	10.42	11.57	11.24	11.5533	11.076667	ENOG4111KU4	S:Function unknown	------	------
BAB_RS19880	-	NADH-quinone oxidoreductase subunit NuoF	1.008	0.011494773	0.824015419	0.893239386	no	up	mRNA	473.8	462.2	531.61	456.55	472.87	464.47	489.203	464.63	COG1894	C:Energy production and conversion	GO:0046872;GO:0051539;GO:0051287;GO:0010181;GO:0008137;GO:0048038	MF:metal ion binding;MF:4 iron, 4 sulfur cluster binding;MF:NAD binding;MF:FMN binding;MF:NADH dehydrogenas;MF:quinone binding;
BAB_RS21775	-	cell envelope biogenesis protein OmpA	1.008	0.011793351	0.835377769	0.900392074	no	up	mRNA	261.24	249.48	263.73	255.7	245.31	239.43	258.15	246.81333	COG2885	L:Replication, recombination and repair	GO:0016021;GO:0009279;GO:0009279;GO:0016021	CC:integral component of membrane;CC:cell outer membrane;CC:cell outer membrane;CC:integral component of membrane;
BAB_RS24235	-	phosphoglycerate kinase	1.008	0.010843443	0.863818217	0.916897236	no	up	mRNA	234.53	215.54	225.22	196.25	218.76	226.59	225.097	213.86667	COG0126	G:Carbohydrate transport and metabolism	GO:0006096;GO:0006096;GO:0005737;GO:0005737;GO:0004618;GO:0005524;GO:0005524;GO:0004618	BP:glycolytic process;BP:glycolytic process;CC:cytoplasm;CC:cytoplasm;MF:phosphoglycerate kinase activity;MF:ATP binding;MF:ATP binding;MF:phosphoglycerate kinase activity;
BAB_RS23605	-	histidine phosphotransferase	1.008	0.0116993	0.867956711	0.917954218	no	up	mRNA	102.88	100.02	95.76	91.38	98.61	94.86	99.5533	94.95	COG5385	S:Function unknown	GO:0018197;GO:0000160;GO:0005737;GO:0016740;GO:0042803;GO:0004672	BP:peptidyl-aspartic acid modification;BP:phosphorelay signal transduction system;CC:cytoplasm;MF:transferase activity;MF:protein homodimerization activity;MF:protein kinase activity;
BAB_RS25755	-	thioredoxin domain-containing protein	1.008	0.012109477	0.874973107	0.922641481	no	up	mRNA	19.55	18.04	21.85	17.48	20.05	18.82	19.8133	18.783333	COG1331	O:Posttranslational modification, protein turnover, chaperones	GO:0003824	MF:catalytic activity;
BAB_RS30595	-	respiratory nitrate reductase subunit gamma	1.008	0.011037732	0.878873633	0.925236233	no	up	mRNA	337.05	303.51	311.25	286.77	286.89	334.67	317.27	302.77667	COG2181	C:Energy production and conversion	GO:0016021;GO:0009325;GO:0016021;GO:0009325;GO:0016021;GO:0009325;GO:0008940;GO:0008940;GO:0004475;GO:0008940	CC:integral component of membrane;CC:nitrate reductase complex;CC:integral component of membrane;CC:nitrate reductase complex;CC:integral component of membrane;CC:nitrate reductase complex;MF:nitrate reductase activity;MF:nitrate reductase activity;MF:mannose-1-phosphate guanylyltransferase activity;MF:nitrate reductase activity;
BAB_RS26305	-	hypothetical protein	1.008	0.011970368	0.884241164	0.927846787	no	up	mRNA	93.16	94.43	116.97	102.39	95.49	92.38	101.52	96.753333	COG3807	S:Function unknown	GO:0016021	CC:integral component of membrane;
BAB_RS21135	-	flavin reductase family protein	1.008	0.01110807	0.889876326	0.931023326	no	up	mRNA	96.77	81.99	91.64	77.3	89.76	90.36	90.1333	85.806667	COG1853	S:Function unknown	GO:0010181;GO:0004497;GO:0004497;GO:0010181;GO:0010181	MF:FMN binding;MF:monooxygenase activity;MF:monooxygenase activity;MF:FMN binding;MF:FMN binding;
BAB_RS19070	-	hypothetical protein	1.007	0.010258529	0.847297733	0.908667429	no	up	mRNA	557.78	537.27	557.71	507.2	519.92	542.72	550.92	523.28	ENOG41120FG	S:Function unknown	GO:0016021	CC:integral component of membrane;
BAB_RS16450	-	nicotinate phosphoribosyltransferase	1.007	0.009793789	0.862704217	0.916623231	no	up	mRNA	57.93	51.82	59.66	54.53	51.93	53.91	56.47	53.456667	COG1488	H:Coenzyme transport and metabolism	GO:0009435;GO:0019357;GO:0009435;GO:0019357;GO:0009435;GO:0019357;GO:0004514;GO:0004516;GO:0004516;GO:0004514;GO:0008168;GO:0004516;GO:0004514	BP:NAD biosynthetic process;BP:nicotinate nucleotide biosynthetic process;BP:NAD biosynthetic process;BP:nicotinate nucleotide biosynthetic process;BP:NAD biosynthetic process;BP:nicotinate nucleotide biosynthetic process;MF:nicotinate-nucleotide diphosphorylas;MF:nicotinate phosphoribosyltransferase activity;MF:nicotinate phosphoribosyltransferase activity;MF:nicotinate-nucleotide diphosphorylas;MF:methyltransferase activity;MF:nicotinate phosphoribosyltransferase activity;MF:nicotinate-nucleotide diphosphorylas;
BAB_RS19645	-	RDD family protein	1.007	0.009483681	0.865888135	0.917276155	no	up	mRNA	565.77	569.6	587.25	535.25	556.78	566.46	574.207	552.83	COG1714	S:Function unknown	GO:0016021	CC:integral component of membrane;
BAB_RS24050	-	bile acid:sodium symporter family protein	1.007	0.010317857	0.879526269	0.924711356	no	up	mRNA	123.56	104.75	109.46	108.74	104.99	106.63	112.59	106.78667	COG0385	E:Amino acid transport and metabolism	GO:0016021	CC:integral component of membrane;
BAB_RS22940	-	MFS transporter	1.007	0.009433449	0.878311289	0.924947287	no	up	mRNA	125.59	111.71	113.95	109.61	110.16	113.15	117.083	110.97333	ENOG410XP7I	G:Carbohydrate transport and metabolism	GO:0055085;GO:0055085;GO:0016021;GO:0016021;GO:0016021;GO:0005215;GO:0022857;GO:0005215	BP:transmembrane transport;BP:transmembrane transport;CC:integral component of membrane;CC:integral component of membrane;CC:integral component of membrane;MF:transporter activity;MF:transmembrane transporter activity;MF:transporter activity;
BAB_RS25445	-	YdcF family protein	1.007	0.009481755	0.879021477	0.925088767	no	up	mRNA	150.51	156.9	154.53	145.99	145.51	148.75	153.98	146.75	COG1434	I:Lipid transport and metabolism	GO:0016021	CC:integral component of membrane;
BAB_RS19185	-	holo-ACP synthase	1.007	0.01015606	0.88195023	0.926047741	no	up	mRNA	138.3	128.38	140.58	132.46	125.02	133.73	135.753	130.40333	COG0736	I:Lipid transport and metabolism	GO:0006633;GO:0006633;GO:0005737;GO:0005737;GO:0008897;GO:0000287;GO:0016491;GO:0008897;GO:0000287	BP:fatty acid biosynthetic process;BP:fatty acid biosynthetic process;CC:cytoplasm;CC:cytoplasm;MF:holo-[acyl-carrier-protein] synthase activity;MF:magnesium ion binding;MF:oxidoreductase activity;MF:holo-[acyl-carrier-protein] synthase activity;MF:magnesium ion binding;
BAB_RS24200	-	TerC family protein	1.007	0.010154937	0.905241499	0.941580103	no	up	mRNA	22.3	21.93	27.76	23.69	23.23	21.38	23.9967	22.766667	COG0861	S:Function unknown	GO:0016021	CC:integral component of membrane;
BAB_RS32255	-	transposase	1.007	0.009620706	0.946164436	0.964475359	no	up	mRNA	23.49	18.3	24.41	24.57	20.26	17.93	22.0667	20.92	COG3293	M:Cell wall/membrane/envelope biogenesis	GO:0006313;GO:0004803;GO:0003677	BP:transposition, DNA-mediated;MF:transposase activity;MF:DNA binding;
BAB_RS32065	-	multidrug ABC transporter ATP-binding protein	1.006	0.009299918	0.890736604	0.931013894	no	up	mRNA	361.95	343.74	404.49	319.83	373.29	355.91	370.06	349.67667	COG1132	V:Defense mechanisms	------	------
BAB_RS30590	-	nitrate reductase molybdenum cofactor assembly chaperone	1.006	0.008173662	0.908670646	0.943619517	no	up	mRNA	334.8	335.27	335.95	315.92	294.22	349.74	335.34	319.96	COG2180	C:Energy production and conversion	GO:0051131;GO:0051131;GO:0051082;GO:0051082	BP:chaperone-mediated protein complex assembly;BP:chaperone-mediated protein complex assembly;MF:unfolded protein binding;MF:unfolded protein binding;
BAB_RS24770	-	helix-turn-helix domain-containing protein	1.006	0.008068	0.927513621	0.955160662	no	up	mRNA	18.38	17.53	16.96	15.33	16.85	17.85	17.6233	16.676667	COG1396	L:Replication, recombination and repair	GO:0043565	MF:sequence-specific DNA binding;
BAB_RS28325	-	sulfite exporter TauE/SafE family protein	1.006	0.008538595	0.933349129	0.956571212	no	up	mRNA	33.12	30.39	38.12	34.39	28.21	34.19	33.8767	32.263333	COG0730	S:Function unknown	GO:0016021;GO:0005886	CC:integral component of membrane;CC:plasma membrane;
BAB_RS27200	-	glycolate oxidase iron-sulfur subunit	1.006	0.009074071	0.940717855	0.961363381	no	up	mRNA	12.66	13.4	15.88	13.64	14.71	11.51	13.98	13.286667	COG0247	C:Energy production and conversion	GO:0051536	MF:iron-sulfur cluster binding;
BAB_RS21705	-	acyl-CoA carboxylase subunit beta	1.006	0.009254733	0.95418235	0.969572388	no	up	mRNA	4.31	6.26	6.13	5.39	5.38	5.32	5.56667	5.3633333	COG4799	K:Transcription	GO:0006633;GO:0006633;GO:0006633;GO:0006633;GO:0009317;GO:0009317;GO:0009317;GO:0009317;GO:0004658;GO:0016740;GO:0003989;GO:0003989;GO:0004658;GO:0016740;GO:0003989;GO:0003989;GO:0016740	BP:fatty acid biosynthetic process;BP:fatty acid biosynthetic process;BP:fatty acid biosynthetic process;BP:fatty acid biosynthetic process;CC:acetyl-CoA carboxylase complex;CC:acetyl-CoA carboxylase complex;CC:acetyl-CoA carboxylase complex;CC:acetyl-CoA carboxylase complex;MF:propionyl-CoA carboxylase activity;MF:transferase activity;MF:acetyl-CoA carboxylase activity;MF:acetyl-CoA carboxylase activity;MF:propionyl-CoA carboxylase activity;MF:transferase activity;MF:acetyl-CoA carboxylase activity;MF:acetyl-CoA carboxylase activity;MF:transferase activity;
BAB_RS32420	-	hypothetical protein	1.006	0.008308716	0.990259874	0.994282805	no	up	mRNA	19.49	51.35	56.69	37.48	36.36	85.18	42.51	53.006667	------	------	------	------
BAB_RS19150	-	GTP pyrophosphokinase rsh	1.005	0.007857929	0.877573071	0.924776083	no	up	mRNA	64.34	60.81	69.38	60.43	61.35	62.14	64.8433	61.306667	COG0317;COG0317	K:Transcription;T:Signal transduction mechanisms	GO:0015969;GO:0015969;GO:0008728;GO:0016301;GO:0005524;GO:0005525	BP:guanosine tetraphosphate metabolic process;BP:guanosine tetraphosphate metabolic process;MF:GTP diphosphokinase activity;MF:kinase activity;MF:ATP binding;MF:GTP binding;
BAB_RS19910	-	NADH-quinone oxidoreductase subunit L	1.005	0.006662429	0.905612005	0.941660638	no	up	mRNA	549.39	494.8	525.92	480.54	512.53	492.18	523.37	495.08333	COG1009	C:Energy production and conversion	GO:0042773;GO:0016021;GO:0008137	BP:ATP synthesis coupled electron transport;CC:integral component of membrane;MF:NADH dehydrogenas;
BAB_RS20590	-	cyclic pyranopterin monophosphate synthase	1.005	0.007200275	0.910178096	0.944574361	no	up	mRNA	394.78	362.95	369.88	377.85	356.41	335.12	375.87	356.46	COG2896	H:Coenzyme transport and metabolism	GO:0006777;GO:0019008;GO:0051539;GO:1904047;GO:0061798;GO:0046872;GO:0005525	BP:Mo-molybdopterin cofactor biosynthetic process;CC:molybdopterin synthase complex;MF:4 iron, 4 sulfur cluster binding;MF:S-adenosyl-L-methionine binding;MF:GTP 3',8'-cyclase activity;MF:metal ion binding;MF:GTP binding;
BAB_RS16140	-	SH3 domain-containing protein	1.005	0.007755501	0.950180693	0.967035339	no	up	mRNA	24.34	29.05	26.77	27.58	23.73	25.7	26.72	25.67	ENOG410Y84G	S:Function unknown	------	------
BAB_RS28400	-	ABC transporter ATP-binding protein	1.005	0.006505726	0.954415015	0.969502195	no	up	mRNA	11.6	11.37	12.13	12.13	10.8	10.37	11.7	11.1	COG3839	S:Function unknown	GO:0043190;GO:0043190;GO:0005524;GO:0016887;GO:0022857;GO:0022857;GO:0005524;GO:0016887	CC:ATP-binding cassett;CC:ATP-binding cassett;MF:ATP binding;MF:ATPase activity;MF:transmembrane transporter activity;MF:transmembrane transporter activity;MF:ATP binding;MF:ATPase activity;
BAB_RS28695	-	carbohydrate ABC transporter permease	1.005	0.006978087	0.955795886	0.969984896	no	up	mRNA	25.46	22.11	21.71	18.6	24.17	22.62	23.0933	21.796667	COG0395	S:Function unknown	GO:0055085;GO:0008643;GO:0055085;GO:0016021;GO:0005886;GO:0016021;GO:0005886;GO:0008168	BP:transmembrane transport;BP:carbohydrate transport;BP:transmembrane transport;CC:integral component of membrane;CC:plasma membrane;CC:integral component of membrane;CC:plasma membrane;MF:methyltransferase activity;
BAB_RS18595	-	transposase	1.004	0.005574847	0.923635572	0.953918705	no	up	mRNA	70.34	63.54	74.13	66.66	64.52	65.48	69.3367	65.553333	ENOG41123IT;COG3293	L:Replication, recombination and repair;	GO:0003677	MF:DNA binding;
BAB_RS32980	-	hypothetical protein	1.004	0.006163445	0.923498482	0.954083802	no	up	mRNA	27.36	23.72	27.36	25.99	24.9	23.02	26.1467	24.636667	COG3468;COG3468;ENOG410ZITU	M:Cell wall/membrane/envelope biogenesis;U:Intracellular trafficking, secretion, and vesicular transport;S:Function unknown	GO:0019867	CC:outer membrane;
BAB_RS30490	-	DUF3597 domain-containing protein	1.004	0.005306742	0.925210635	0.954931504	no	up	mRNA	4624.35	4502.1	4558.26	4176.55	4363	4518.54	4561.57	4352.6967	ENOG4111WV7	S:Function unknown	------	------
BAB_RS21295	-	DNA gyrase subunit A	1.004	0.005127284	0.927312254	0.955259465	no	up	mRNA	354.59	323.36	336.98	312.71	323.97	322	338.31	319.56	COG0188	H:Coenzyme transport and metabolism	GO:0006265;GO:0006261;GO:0006265;GO:0006261;GO:0005694;GO:0005737;GO:0005737;GO:0005694;GO:0005524;GO:0003677;GO:0003918;GO:0003677;GO:0003918;GO:0005524	BP:DNA topological change;BP:DNA-dependent DNA replication;BP:DNA topological change;BP:DNA-dependent DNA replication;CC:chromosome;CC:cytoplasm;CC:cytoplasm;CC:chromosome;MF:ATP binding;MF:DNA binding;MF:DNA topoisomerase type I;MF:DNA binding;MF:DNA topoisomerase type I;MF:ATP binding;
BAB_RS29700	-	DNA oxidative demethylase AlkB	1.004	0.006078595	0.931088591	0.956084258	no	up	mRNA	40	35.24	41.11	38.31	35.4	36.32	38.7833	36.676667	COG3145	L:Replication, recombination and repair	GO:0008168;GO:0016491;GO:0051213;GO:0016491;GO:0016491	MF:methyltransferase activity;MF:oxidoreductase activity;MF:dioxygenase activity;MF:oxidoreductase activity;MF:oxidoreductase activity;
BAB_RS24120	-	Holliday junction branch migration protein RuvA	1.004	0.005841756	0.933234163	0.956758572	no	up	mRNA	115.16	111.47	119.29	111.2	102.69	116.02	115.307	109.97	COG0632	L:Replication, recombination and repair	GO:0006310;GO:0006281;GO:0009432;GO:0006310;GO:0006281;GO:0009432;GO:0006310;GO:0006281;GO:0009432;GO:0009379;GO:0009379;GO:0009378;GO:0016740;GO:0003677;GO:0005524;GO:0005524;GO:0003677;GO:0009378;GO:0009378;GO:0003677;GO:0005524	BP:DNA recombination;BP:DNA repair;BP:SOS response;BP:DNA recombination;BP:DNA repair;BP:SOS response;BP:DNA recombination;BP:DNA repair;BP:SOS response;CC:Holliday junction helicase complex;CC:Holliday junction helicase complex;MF:four-way junction helicase activity;MF:transferase activity;MF:DNA binding;MF:ATP binding;MF:ATP binding;MF:DNA binding;MF:four-way junction helicase activity;MF:four-way junction helicase activity;MF:DNA binding;MF:ATP binding;
BAB_RS25845	-	HslU--HslV peptidase proteolytic subunit	1.004	0.005619985	0.93260573	0.957030409	no	up	mRNA	231.97	218.81	234.98	231.06	220.31	200.38	228.587	217.25	COG5405	O:Posttranslational modification, protein turnover, chaperones	GO:0051603;GO:0051603;GO:0051603;GO:0005839;GO:0009376;GO:0009376;GO:0005839;GO:0009376;GO:0005839;GO:0046872;GO:0004298;GO:0004298;GO:0046872;GO:0046872;GO:0004298	BP:proteolysis involved in cellular protein catabolic process;BP:proteolysis involved in cellular protein catabolic process;BP:proteolysis involved in cellular protein catabolic process;CC:proteasome core complex;CC:HslUV protease complex;CC:HslUV protease complex;CC:proteasome core complex;CC:HslUV protease complex;CC:proteasome core complex;MF:metal ion binding;MF:threonine-type endopeptidase activity;MF:threonine-type endopeptidase activity;MF:metal ion binding;MF:metal ion binding;MF:threonine-type endopeptidase activity;
BAB_RS27015	-	AraC family transcriptional regulator	1.004	0.005488498	0.955659757	0.970153175	no	up	mRNA	25.17	25.32	23.7	23.15	21.35	25.88	24.73	23.46	ENOG4111NRR	K:Transcription	GO:0006351;GO:0003700;GO:0043565	BP:transcription, DNA-templated;MF:transcription factor activity, sequence-specific DNA binding;MF:sequence-specific DNA binding;
BAB_RS28270	-	hypothetical protein	1.004	0.005797431	0.970395248	0.980773806	no	up	mRNA	15.24	16.09	20.73	14.75	16.09	19.32	17.3533	16.72	------	------	------	------
BAB_RS23075	-	Na/Pi cotransporter family protein	1.003	0.004074393	0.940773097	0.961114136	no	up	mRNA	175.62	159.22	175.29	154.84	169.93	156.38	170.043	160.38333	COG1283	P:Inorganic ion transport and metabolism	GO:0016021;GO:0016021;GO:0015321;GO:0015321	CC:integral component of membrane;CC:integral component of membrane;MF:sodium-dependent phosphate transmembrane transporter activity;MF:sodium-dependent phosphate transmembrane transporter activity;
BAB_RS32525	-	hypothetical protein	1.003	0.004017768	0.949254986	0.966399325	no	up	mRNA	347.13	324.21	314.25	307.44	309.12	315.86	328.53	310.80667	------	------	------	------
BAB_RS26670	-	type IV secretion system protein VirB4	1.003	0.00427033	0.95939033	0.972404142	no	up	mRNA	9.81	8.6	9.51	8.99	8.46	8.85	9.30667	8.7666667	COG3451	U:Intracellular trafficking, secretion, and vesicular transport	GO:0009405;GO:0005524;GO:0005524	BP:pathogenesis;MF:ATP binding;MF:ATP binding;
BAB_RS26130	-	phosphate ABC transporter permease subunit PstC	1.002	0.003589015	0.955617406	0.970416791	no	up	mRNA	45.05	38.86	42.49	39.49	39.64	39.99	42.1333	39.706667	COG0573	P:Inorganic ion transport and metabolism	GO:0006817;GO:0006817;GO:0016021;GO:0005886;GO:0016021;GO:0005886;GO:0005315;GO:0005315	BP:phosphate ion transport;BP:phosphate ion transport;CC:integral component of membrane;CC:plasma membrane;CC:integral component of membrane;CC:plasma membrane;MF:inorganic phosphate transmembrane transporter activity;MF:inorganic phosphate transmembrane transporter activity;
BAB_RS17465	-	TetR family transcriptional regulator	1.002	0.003293107	0.959373205	0.972693628	no	up	mRNA	119.16	108.16	122.68	105.24	116.03	110.82	116.667	110.69667	COG1309;COG1309	I:Lipid transport and metabolism;Q:Secondary metabolites biosynthesis, transport and catabolism	GO:0006355;GO:0006351;GO:0006355;GO:0006351;GO:0003677;GO:0003677	BP:regulation of transcription, DNA-templated;BP:transcription, DNA-templated;BP:regulation of transcription, DNA-templated;BP:transcription, DNA-templated;MF:DNA binding;MF:DNA binding;
BAB_RS31095	-	glucokinase	1.002	0.002506168	0.960601639	0.97301799	no	up	mRNA	251.22	224.21	259.95	227.34	231.67	235.65	245.127	231.55333	COG0837	G:Carbohydrate transport and metabolism	GO:0006096;GO:0006096;GO:0005737;GO:0005737;GO:0004340;GO:0005524;GO:0005524;GO:0004340	BP:glycolytic process;BP:glycolytic process;CC:cytoplasm;CC:cytoplasm;MF:glucokinase activity;MF:ATP binding;MF:ATP binding;MF:glucokinase activity;
BAB_RS23245	-	DNA-binding response regulator	1.002	0.00265354	0.960460169	0.973181495	no	up	mRNA	257.21	246.71	265.84	233.68	248.16	248.44	256.587	243.42667	COG0745	K:Transcription	GO:0000160;GO:0006355;GO:0006351;GO:0000160;GO:0006351;GO:0006355;GO:0005622;GO:0005622;GO:0003677;GO:0003677	BP:phosphorelay signal transduction system;BP:regulation of transcription, DNA-templated;BP:transcription, DNA-templated;BP:phosphorelay signal transduction system;BP:transcription, DNA-templated;BP:regulation of transcription, DNA-templated;CC:intracellular;CC:intracellular;MF:DNA binding;MF:DNA binding;
BAB_RS31885	-	plasmid partitioning protein RepB	1.002	0.00281015	0.963777387	0.975927118	no	up	mRNA	115.87	104.07	116.2	111.6	105.56	100.05	112.047	105.73667	COG1475	E:Amino acid transport and metabolism	GO:0003677	MF:DNA binding;
BAB_RS26560	-	two-component sensor histidine kinase	1.002	0.003253098	0.967858431	0.979442248	no	up	mRNA	25.4	24.24	25.91	22.64	23.87	24.95	25.1833	23.82	ENOG410XNMH	S:Function unknown	GO:0016021;GO:0005622;GO:0016021;GO:0005622;GO:0005524;GO:0000155;GO:0000155;GO:0005524	CC:integral component of membrane;CC:intracellular;CC:integral component of membrane;CC:intracellular;MF:ATP binding;MF:phosphorelay sensor kinase activity;MF:phosphorelay sensor kinase activity;MF:ATP binding;
BAB_RS30175	-	N-acetylneuraminate epimerase	1.002	0.002642461	0.978727055	0.98764134	no	up	mRNA	19.39	19.32	17.61	18.28	16.94	18.01	18.7733	17.743333	COG3055	S:Function unknown	GO:0005975;GO:0005975;GO:0042597;GO:0042597;GO:0016857;GO:0016857;GO:0016853	BP:carbohydrate metabolic process;BP:carbohydrate metabolic process;CC:periplasmic space;CC:periplasmic space;MF:racemase and epimerase activity, acting on carbohydrates and derivatives;MF:racemase and epimerase activity, acting on carbohydrates and derivatives;MF:isomerase activity;
BAB_RS22185	-	hypothetical protein	1.002	0.002274692	0.986570913	0.991818631	no	up	mRNA	111.7	87.98	92	105.44	96.99	73.22	97.2267	91.883333	------	------	------	------
BAB_RS18015	-	acyl-CoA dehydrogenase	1.001	0.002042829	0.982848758	0.990556167	no	up	mRNA	17.37	15.76	16.6	14.24	15.07	17.56	16.5767	15.623333	COG1960	I:Lipid transport and metabolism	GO:0003995;GO:0016627;GO:0050660;GO:0043958	MF:acyl-CoA dehydrogenase activity;MF:oxidoreductase activity, acting on the CH-CH group of donors;MF:flavin adenine dinucleotide binding;MF:acryloyl-CoA reductase activity;
BAB_RS25865	-	1-(5-phosphoribosyl)-5-[(5-phosphoribosylamino)methylideneamino]imidazole-4-carboxamide isomerase	1.001	0.001853311	0.982596583	0.990612746	no	up	mRNA	44.99	44.57	43.71	44.04	41.48	40.88	44.4233	42.133333	COG0106	E:Amino acid transport and metabolism	GO:0000105;GO:0000105;GO:0005737;GO:0005737;GO:0003949;GO:0016787;GO:0003949	BP:histidine biosynthetic process;BP:histidine biosynthetic process;CC:cytoplasm;CC:cytoplasm;MF:1;MF:hydrolase activity;MF:1;
BAB_RS19440	-	3-oxoacyl-ACP reductase	1.001	0.001319208	0.987087227	0.992027294	no	up	mRNA	49.09	42.64	43.23	44.58	42.07	40.44	44.9867	42.363333	ENOG410XQFJ	S:Function unknown	------	------
BAB_RS17530	-	O-succinylhomoserine sulfhydrylase	1.001	0.001143087	0.989803104	0.994134846	no	up	mRNA	16.23	15.4	18.67	17.05	15.94	14.39	16.7667	15.793333	COG0626	E:Amino acid transport and metabolism	GO:0071266;GO:0071268;GO:0071266;GO:0071268;GO:0016765;GO:0016829;GO:0030170;GO:0016829;GO:0030170;GO:0016765	BP:'de novo' L-methionine biosynthetic process;BP:homocysteine biosynthetic process;BP:'de novo' L-methionine biosynthetic process;BP:homocysteine biosynthetic process;MF:transferase activity, transferring alkyl or aryl;MF:lyase activity;MF:pyridoxal phosphate binding;MF:lyase activity;MF:pyridoxal phosphate binding;MF:transferase activity, transferring alkyl or ary;
BAB_RS26015	-	alpha/beta hydrolase	1.001	0.000935963	0.990530872	0.994244202	no	up	mRNA	92.94	81.3	86.44	77.68	90.26	78.02	86.8933	81.986667	COG0596	Q:Secondary metabolites biosynthesis, transport and catabolism	GO:0016787	MF:hydrolase activity;
BAB_RS21915	-	50S ribosomal protein L4	1	0.000686118	0.988767467	0.993405213	no	up	mRNA	1238.93	1167.19	1273.84	1133.03	1198.67	1162.65	1226.65	1164.7833	COG0088	J:Translation, ribosomal structure and biogenesis	GO:0006412;GO:0006412;GO:0005840;GO:0005840;GO:0019843;GO:0003735;GO:0003735;GO:0019843	BP:translation;BP:translation;CC:ribosome;CC:ribosome;MF:rRNA binding;MF:structural constituent of ribosome;MF:structural constituent of ribosome;MF:rRNA binding;
BAB_RS20705	-	hypothetical protein	1	0.000601427	0.995412495	0.996653271	no	up	mRNA	48.73	44.02	52.69	40.6	46.93	50.79	48.48	46.106667	ENOG410XVGJ	S:Function unknown	------	------
BAB_RS30725	-	ribulokinase	1	0.000381475	0.995141036	0.996692066	no	up	mRNA	38.58	34.26	38.82	35.64	34	35.43	37.22	35.023333	COG1069	G:Carbohydrate transport and metabolism	GO:0005975;GO:0005975;GO:0016301;GO:0016773;GO:0016773;GO:0016301;GO:0019150;GO:0016757;GO:0019150;GO:0016773;GO:0016301	BP:carbohydrate metabolic process;BP:carbohydrate metabolic process;MF:kinase activity;MF:phosphotransferase activity, alcohol group as acceptor;MF:phosphotransferase activity, alcohol group as acceptor;MF:kinase activity;MF:D-ribulokinase activity;MF:transferase activity, transferring glycosyl groups;MF:D-ribulokinase activity;MF:phosphotransferase activity, alcohol group as acceptor;MF:kinase activity;
BAB_RS26350	-	30S ribosomal protein S20	1	0.000671892	0.995085056	0.996946768	no	up	mRNA	5093.2	5482.88	5589.39	5850.44	5261.37	4844.79	5388.49	5318.8667	COG0268	J:Translation, ribosomal structure and biogenesis	GO:0006412;GO:0006412;GO:0005840;GO:0005840;GO:0019843;GO:0003735;GO:0003735;GO:0019843	BP:translation;BP:translation;CC:ribosome;CC:ribosome;MF:rRNA binding;MF:structural constituent of ribosome;MF:structural constituent of ribosome;MF:rRNA binding;
BAB_RS29880	-	biotin synthase BioB	1	0.000705106	0.994680977	0.9971638	no	up	mRNA	178.45	168.71	161.29	184.68	159.32	136.17	169.483	160.05667	COG0502	H:Coenzyme transport and metabolism	GO:0009102;GO:0009102;GO:0004076;GO:0005506;GO:0051537;GO:0051539;GO:0051537;GO:0051539;GO:0004076;GO:0005506	BP:biotin biosynthetic process;BP:biotin biosynthetic process;MF:biotin synthase activity;MF:iron ion binding;MF:2 iron, 2 sulfur cluster binding;MF:4 iron, 4 sulfur cluster binding;MF:2 iron, 2 sulfur cluster binding;MF:4 iron, 4 sulfur cluster binding;MF:biotin synthase activity;MF:iron ion binding;
BAB_RS20990	-	type IV secreted protein VceC	1	0.000207071	0.997013864	0.997634863	no	up	mRNA	267.99	248.7	255.57	234.14	242.33	251.97	257.42	242.81333	ENOG41125G1	S:Function unknown	GO:0044781;GO:0016021	BP:bacterial-type flagellum organization;CC:integral component of membrane;
BAB_RS26180	-	ribosomal-protein-alanine N-acetyltransferase	1	5.37283E-05	0.999678658	0.999678658	no	up	mRNA	48.89	48.74	56.71	41.43	56.83	49.34	51.4467	49.2	COG0456	S:Function unknown	GO:0006474;GO:0006474;GO:0006474;GO:0016407;GO:0003864;GO:0016407;GO:0008999;GO:0016407	BP:N-terminal protein amino acid acetylation;BP:N-terminal protein amino acid acetylation;BP:N-terminal protein amino acid acetylation;MF:acetyltransferase activity;MF:3-methyl-2-oxobutanoate hydroxymethyltransferase activity;MF:acetyltransferase activity;MF:ribosomal-protein-alanine N-acetyltransferase activity;MF:acetyltransferase activity;

Genes down-regulated in 2308Δ*vdtR* comparing to 2308

Gene_id	Gene name	Gene description	FC(2308ΔvdtR/2308)	Log2FC(vdtR/S2308)	Pvalue	Padjust	Significant	Regulate	Type	2308_1	2308_2	2308_3	2308ΔvdtR_1	2308ΔvdtR_2	2308ΔvdtR_3	2308	**2308ΔvdtR**	COG_id	COG_Functional Categories	GO_id	GO_term
BAB_RS21730	-	hypothetical protein	0.429	-1.221047417	1.95383E-09	4.48404E-07	yes	down	mRNA	403.83	375.95	382.09	148.99	152.33	169.73	387.29	157.016667	COG2358	P:Inorganic ion transport and metabolism	------	------
BAB_RS27705	-	ABC transporter ATP-binding protein	0.296	-1.757959254	9.10789E-09	1.62576E-06	yes	down	mRNA	47.86	38.9	46.26	11.06	12.7	13.27	44.34	12.3433333	COG0411	P:Inorganic ion transport and metabolism	GO:0005524;GO:0016887;GO:0016887;GO:0005524	MF:ATP binding;MF:ATPase activity;MF:ATPase activity;MF:ATP binding;
BAB_RS27710	-	ABC transporter permease	0.348	-1.523505099	1.10124E-08	1.86226E-06	yes	down	mRNA	179.21	174.79	171.63	49.5	57.2	66	175.21	57.5666667	ENOG410XNWR	E:Amino acid transport and metabolism	------	------
BAB_RS24665	-	methylmalonate-semialdehyde dehydrogenase (CoA acylating)	0.295	-1.760875496	3.06071E-08	4.27568E-06	yes	down	mRNA	1303.05	1215.65	1256.57	289.96	329.52	431.32	1258.42	350.266667	COG1012	S:Function unknown	GO:0019179;GO:0004491;GO:0004491	MF:dTDP-4-amino-4,6-dideoxy-D-glucose transaminase activity;MF:methylmalonate-semialdehyde dehydrogenas;MF:methylmalonate-semialdehyde dehydrogenas;
BAB_RS27690	-	branched-chain amino acid ABC transporter permease	0.467	-1.099473411	5.48158E-08	6.77396E-06	yes	down	mRNA	83.63	74.77	74.67	32.04	36.74	33.63	77.69	34.1366667	COG4177	P:Inorganic ion transport and metabolism	GO:0016021;GO:0005886;GO:0022857	CC:integral component of membrane;CC:plasma membrane;MF:transmembrane transporter activity;
BAB_RS19575	-	amino acid ABC transporter substrate-binding protein	0.441	-1.182145793	1.02901E-07	1.10207E-05	yes	down	mRNA	97.55	81.05	82.13	37.53	37.53	33.07	86.91	36.0433333	COG0834	E:Amino acid transport and metabolism	------	------
BAB_RS24660	-	hypothetical protein	0.474	-1.078160281	1.63713E-07	1.64378E-05	yes	down	mRNA	281.1	284.02	309.57	120.19	123.87	151.12	291.563	131.726667	ENOG4112CCK	S:Function unknown	GO:0016021	CC:integral component of membrane;
BAB_RS27700	-	ABC transporter ATP-binding protein	0.364	-1.459922617	2.57916E-07	2.07171E-05	yes	down	mRNA	78.64	65.95	58.65	20.63	24.59	24.28	67.7467	23.1666667	COG0410	P:Inorganic ion transport and metabolism	GO:0005524;GO:0016887;GO:0016301;GO:0005524;GO:0016887	MF:ATP binding;MF:ATPase activity;MF:kinase activity;MF:ATP binding;MF:ATPase activity;
BAB_RS27685	-	choline dehydrogenase	0.453	-1.141833898	1.44989E-06	8.78962E-05	yes	down	mRNA	31.71	28.19	24.37	11.87	12.71	11.23	28.09	11.9366667	COG2303	P:Inorganic ion transport and metabolism	GO:0050660;GO:0016614	MF:flavin adenine dinucleotide binding;MF:oxidoreductase activity, acting on CH-OH group of donors;
BAB_RS30270	-	branched-chain amino acid ABC transporter permease	0.469	-1.091134693	4.56679E-05	0.001146335	yes	down	mRNA	14.51	16.29	13.36	5.71	6.26	7.71	14.72	6.56	COG0559	I:Lipid transport and metabolism	GO:0016021;GO:0005886;GO:0022857	CC:integral component of membrane;CC:plasma membrane;MF:transmembrane transporter activity;
BAB_RS33390	-	hypothetical protein	0.438	-1.189663221	0.000392262	0.00443781	yes	down	mRNA	24.91	18.68	23.05	8.3	9.75	10.11	22.2133	9.38666667	------	------	------	------
BAB_RS22440	-	ABC transporter permease	0.464	-1.106709124	0.000692785	0.006470688	yes	down	mRNA	20.31	14.06	19.84	5.68	8.3	9.95	18.07	7.97666667	COG1172	G:Carbohydrate transport and metabolism	GO:0016021;GO:0005886;GO:0022857	CC:integral component of membrane;CC:plasma membrane;MF:transmembrane transporter activity;
BAB_RS21725	-	DUF3394 domain-containing protein	0.516	-0.953684164	7.42976E-09	1.40423E-06	no	down	mRNA	146.64	133.54	139.35	64.68	69.03	70.17	139.843	67.96	COG4666	S:Function unknown	GO:0016021	CC:integral component of membrane;
BAB_RS27245	-	glucose/galactose transporter	0.563	-0.828008298	2.207E-08	3.54555E-06	no	down	mRNA	467.38	431.53	439.5	236.8	240.61	233.37	446.137	236.926667	COG0738	G:Carbohydrate transport and metabolism	GO:0016021;GO:0005886;GO:0005355;GO:0005354	CC:integral component of membrane;CC:plasma membrane;MF:glucose transmembrane transporter activity;MF:galactose transmembrane transporter activity;
BAB_RS22445	-	LacI family transcriptional regulator	0.517	-0.951363352	5.94472E-08	7.07422E-06	no	down	mRNA	109.73	92.41	103.25	46.76	49.43	52.58	101.797	49.59	COG1879	G:Carbohydrate transport and metabolism	GO:0008643	BP:carbohydrate transport;
BAB_RS22480	-	multidrug ABC transporter ATP-binding protein	0.602	-0.73242476	8.75397E-08	9.6988E-06	no	down	mRNA	67.52	64.98	72.95	40.29	37.01	39.01	68.4833	38.77	COG0842	V:Defense mechanisms	GO:0016021;GO:0005524;GO:0016887	CC:integral component of membrane;MF:ATP binding;MF:ATPase activity;
BAB_RS28855	-	5-dehydro-2-deoxygluconokinase	0.527	-0.924600216	1.27797E-07	1.32456E-05	no	down	mRNA	19.31	18.08	21.13	9.42	9.89	9.69	19.5067	9.66666667	COG0524;COG3892	E:Amino acid transport and metabolism;G:Carboh	GO:0016773;GO:0016301	MF:phosphotransferase activity, alcohol group as acceptor;MF:kinase activity;
BAB_RS18670	-	betaine-aldehyde dehydrogenase	0.641	-0.640790934	1.88231E-07	1.67996E-05	no	down	mRNA	228.82	211.37	233.96	129.74	133.58	143.76	224.717	135.693333	COG1012	K:Transcription	GO:0019285;GO:0019285;GO:0046872;GO:0008802;GO:0008802;GO:00	BP:glycine betaine biosynthetic process from choline;BP:glycine betaine biosynthetic process from choline;MF:metal ion binding;MF:betaine-aldehyde dehydrogenase activity;MF:betaine-aldehyde dehydrogenase activity;MF:metal ion binding;
BAB_RS18675	-	choline dehydrogenase	0.598	-0.74076775	1.72799E-07	1.68244E-05	no	down	mRNA	291.07	293.27	331.09	165.34	166.07	184.59	305.143	172	COG2303	E:Amino acid transport and metabolism	------	------
BAB_RS24460	-	branched chain amino acid ABC transporter substrate-binding protein	0.513	-0.961582875	2.15449E-07	1.82168E-05	no	down	mRNA	117.43	94.29	98.48	49.63	49.31	50.93	103.4	49.9566667	COG0683	C:Energy production and conversion	GO:0006865	BP:amino acid transport;
BAB_RS28955	-	sugar-binding periplasmic protein	0.624	-0.680094863	3.29642E-07	2.46312E-05	no	down	mRNA	981.14	888.89	933.99	528.35	540.64	580.21	934.673	549.733333	COG1653;ENOG410XQ0Q	C:Energy production and conversion;G:Carbohydr	GO:0008643;GO:0042597	BP:carbohydrate transport;CC:periplasmic space;
BAB_RS27695	-	branched-chain amino acid ABC transporter permease	0.532	-0.91040724	4.18138E-07	2.98551E-05	no	down	mRNA	100.65	89.56	95.42	43.42	46.81	53.36	95.21	47.8633333	COG0559	P:Inorganic ion transport and metabolism	GO:0016021;GO:0005886;GO:0022857	CC:integral component of membrane;CC:plasma membrane;MF:transmembrane transporter activity;
BAB_RS16880	-	LacI family DNA-binding transcriptional regulator	0.558	-0.842865841	4.55366E-07	3.18063E-05	no	down	mRNA	122.51	99.83	106.6	59.7	56.98	55.98	109.647	57.5533333	COG1609	K:Transcription	GO:0006355;GO:0006351;GO:0006355;GO:0006351;GO:0003677;GO:00	BP:regulation of transcription, DNA-templated;BP:transcription, DNA-templated;BP:regulation of transcription, DNA-templated;BP:transcription, DNA-templated;MF:DNA binding;MF:DNA binding;
BAB_RS22485	-	glycoside hydrolase family 43	0.701	-0.513035275	8.6475E-07	5.91158E-05	no	down	mRNA	293.42	270.11	294.87	194.25	190.28	182.4	286.133	188.976667	ENOG410XPU6	U:Intracellular trafficking, secretion, and vesicular t	GO:0009306;GO:0016021	BP:protein secretion;CC:integral component of membrane;
BAB_RS23465	-	2-isopropylmalate synthase	0.67	-0.578659996	1.17652E-06	7.71458E-05	no	down	mRNA	409.32	355.37	394.66	254.82	237.54	237.57	386.45	243.31	COG0119	S:Function unknown	GO:0009098;GO:0009098;GO:0009098;GO:0016829;GO:0003852;GO:00	BP:leucine biosynthetic process;BP:leucine biosynthetic process;BP:leucine biosynthetic process;MF:lyase activity;MF:2-isopropylmalate synthase activity;MF:2-isopropylmalate synthase activity;MF:2-isopropylmalate synthase activity;MF:lyase activity;
BAB_RS16875	-	DUF779 domain-containing protein	0.687	-0.541345817	1.48176E-06	8.65617E-05	no	down	mRNA	782.31	705.28	704.9	474.84	465.7	498.62	730.83	479.72	COG3564	S:Function unknown	------	------
BAB_RS30265	-	branched-chain amino acid ABC transporter ATP-binding protein	0.555	-0.850219893	1.39318E-06	8.77704E-05	no	down	mRNA	18.08	16.27	16.7	8.56	9.19	8.83	17.0167	8.86	COG0411;COG0559	I:Lipid transport and metabolism;	GO:0016021;GO:0005886;GO:0005524;GO:0022857;GO:0016887	CC:integral component of membrane;CC:plasma membrane;MF:ATP binding;MF:transmembrane transporter activity;MF:ATPase activity;
BAB_RS19165	-	oxygen-independent coproporphyrinogen III oxidase	0.696	-0.523167387	1.3813E-06	8.87624E-05	no	down	mRNA	641.46	587.75	625.54	403.39	406.16	404.98	618.25	404.843333	COG0635	F:Nucleotide transport and metabolism	GO:0006782;GO:0006782;GO:0006782;GO:0005737;GO:0005737;GO:00	BP:protoporphyrinogen IX biosynthetic process;BP:protoporphyrinogen IX biosynthetic process;BP:protoporphyrinogen IX biosynthetic process;CC:cytoplasm;CC:cytoplasm;CC:cytoplasm;MF:4 iron, 4 sulfur cluster binding;MF:coproporphyrinogen oxidase activity;MF:isomerase activity;MF:metal ion binding;MF:4 iron, 4 sulfur cluster binding;MF:coproporphyrinogen oxidase activity;MF:metal ion binding;MF:isomerase activity;MF:4 iron, 4 sulfur cluster binding;MF:coproporphyrinogen oxidase activity;MF:metal ion binding;
BAB_RS33145	-	sugar ABC transporter substrate-binding protein	0.65	-0.622077668	1.7276E-06	9.91213E-05	no	down	mRNA	265.25	232.21	251.78	140.4	162.69	155.89	249.747	152.993333	COG4213	G:Carbohydrate transport and metabolism	------	------
BAB_RS30710	-	ABC transporter ATP-binding protein/permease	0.689	-0.537604156	1.85104E-06	0.000100803	no	down	mRNA	49.37	46.26	51.93	31.11	32.95	31.55	49.1867	31.87	COG4178	C:Energy production and conversion	GO:0016021;GO:0005524;GO:0042626	CC:integral component of membrane;MF:ATP binding;MF:ATPase activity, coupled to transmembrane movement of substances;
BAB_RS23790	-	sugar ABC transporter ATP-binding protein	0.649	-0.623387509	1.88596E-06	0.000100993	no	down	mRNA	108.26	103.2	107.35	59.52	66.67	68.86	106.27	65.0166667	COG1129	G:Carbohydrate transport and metabolism	GO:0005524;GO:0016887	MF:ATP binding;MF:ATPase activity;
BAB_RS29735	-	dihydrolipoyl dehydrogenase	0.685	-0.546627075	3.20309E-06	0.000155932	no	down	mRNA	127.89	130.72	127.71	80.56	84.26	84.72	128.773	83.18	COG1249	S:Function unknown	GO:0045454;GO:0005623;GO:0004148;GO:0009055;GO:0050660	BP:cell redox homeostasis;CC:cell;MF:dihydrolipoyl dehydrogenase activity;MF:electron carrier activity;MF:flavin adenine dinucleotide binding;
BAB_RS29280	-	hypothetical protein	0.68	-0.555660722	3.98432E-06	0.000188259	no	down	mRNA	453.88	417.36	401.25	264.42	272.81	284.16	424.163	273.796667	ENOG410Z0SY	S:Function unknown	------	------
BAB_RS28585	-	branched-chain amino acid ABC transporter permease	0.675	-0.566315503	4.53799E-06	0.000211312	no	down	mRNA	385.47	329.64	334.08	225.26	225.74	217.65	349.73	222.883333	COG1296	E:Amino acid transport and metabolism	GO:0016021	CC:integral component of membrane;
BAB_RS17630	-	CidA/LrgA family protein	0.746	-0.423171154	4.75001E-06	0.000214955	no	down	mRNA	2680.59	2589.61	2686.99	1906.71	1849.94	1920.72	2652.4	1892.45667	COG1380	S:Function unknown	GO:0016021;GO:0005886	CC:integral component of membrane;CC:plasma membrane;
BAB_RS30485	-	ABC transporter substrate-binding protein	0.712	-0.491055493	5.09868E-06	0.000227529	no	down	mRNA	196.01	184.35	188.26	120.92	130.96	129.81	189.54	127.23	COG0687	S:Function unknown	------	------
BAB_RS23745	-	PepSY domain-containing protein	0.679	-0.557906564	5.51349E-06	0.00023939	no	down	mRNA	1199.43	992.17	1054.96	688.4	687.09	703.92	1082.19	693.136667	ENOG410Y23I	S:Function unknown	------	------
BAB_RS19830	-	hypothetical protein	0.684	-0.547692656	5.78812E-06	0.0002447	no	down	mRNA	91.05	88.97	91.91	61.11	56.47	58.38	90.6433	58.6533333	------	------	------	------
BAB_RS22415	-	calcium-binding protein	0.714	-0.485032068	6.18825E-06	0.000258219	no	down	mRNA	531.2	485.01	530.02	344.4	326.41	369.83	515.41	346.88	ENOG410ZYDA;ENOG410XUN8	S:Function unknown;	GO:0005509	MF:calcium ion binding;
BAB_RS23485	-	AsnC family transcriptional regulator	0.635	-0.654479523	6.387E-06	0.000263095	no	down	mRNA	68.39	60.43	60.63	37.5	36.37	39.69	63.15	37.8533333	COG1522	E:Amino acid transport and metabolism	GO:0006351;GO:0005622;GO:0003700;GO:0043565	BP:transcription, DNA-templated;CC:intracellular;MF:transcription factor activity, sequence-specific DNA binding;MF:sequence-specific DNA binding;
BAB_RS24475	-	branched-chain amino acid ABC transporter ATP-binding protein	0.706	-0.501244785	7.30737E-06	0.000293482	no	down	mRNA	84.4	76.67	79.67	53.22	54.65	52.33	80.2467	53.4	COG3743;COG0411	E:Amino acid transport and metabolism;	GO:0005524;GO:0016887	MF:ATP binding;MF:ATPase activity;
BAB_RS26110	-	hypothetical protein	0.694	-0.526539441	7.83261E-06	0.000306904	no	down	mRNA	101.7	99.91	99.82	63.93	65.75	68.34	100.477	66.0066667	COG3672	O:Posttranslational modification, protein turnover,	------	------
BAB_RS26890	-	iron ABC transporter substrate-binding protein	0.726	-0.462511528	9.17053E-06	0.000350773	no	down	mRNA	333.37	294.2	307.33	203.95	220.38	215.4	311.633	213.243333	COG1840	P:Inorganic ion transport and metabolism	------	------
BAB_RS28510	-	carbonic anhydrase	0.648	-0.626063825	9.1602E-06	0.000354599	no	down	mRNA	219.04	185.32	194.94	110.1	131.23	125.25	199.767	122.193333	COG0288	S:Function unknown	GO:0015976;GO:0015976;GO:0004089;GO:0008270;GO:0004089;GO:00	BP:carbon utilization;BP:carbon utilization;MF:carbonate dehydratase activity;MF:zinc ion binding;MF:carbonate dehydratase activity;MF:zinc ion binding;
BAB_RS23760	-	glycerone kinase	0.699	-0.517471452	1.02175E-05	0.00038173	no	down	mRNA	62.72	59.09	61.24	38.24	42.06	40.21	61.0167	40.17	COG2376	G:Carbohydrate transport and metabolism	GO:0030246;GO:0016301	MF:carbohydrate binding;MF:kinase activity;
BAB_RS26885	-	acriflavin resistance protein	0.652	-0.616523122	1.07624E-05	0.000392949	no	down	mRNA	52.99	51.19	50.85	30.59	30.57	34.42	51.6767	31.86	COG4132	S:Function unknown	GO:0055085;GO:0055085;GO:0016021;GO:0005886;GO:0016021;GO:00	BP:transmembrane transport;BP:transmembrane transport;CC:integral component of membrane;CC:plasma membrane;CC:integral component of membrane;CC:plasma membrane;
BAB_RS23355	-	ubiquinol-cytochrome c reductase iron-sulfur subunit	0.749	-0.417762213	1.21263E-05	0.000428152	no	down	mRNA	1048.16	1008.28	1097.82	728.21	792.99	723.16	1051.42	748.12	COG0723	C:Energy production and conversion	GO:0016021;GO:0016020;GO:0016021;GO:0008121;GO:0051537;GO:00	CC:integral component of membrane;CC:membrane;CC:integral component of membrane;MF:ubiquinol-cytochrome-c reductase activity;MF:2 iron, 2 sulfur cluster binding;MF:2 iron, 2 sulfur cluster binding;MF:ubiquinol-cytochrome-c reductase activity;
BAB_RS30300	-	acetate/propionate family kinase	0.65	-0.621216965	1.64694E-05	0.000557014	no	down	mRNA	52	51.97	48.82	29.61	30.1	34.09	50.93	31.2666667	COG0282	C:Energy production and conversion	GO:0006085;GO:0006082;GO:0005737;GO:0008776;GO:0000287;GO:00	BP:acetyl-CoA biosynthetic process;BP:organic acid metabolic process;CC:cytoplasm;MF:acetate kinase activity;MF:magnesium ion binding;MF:ATP binding;MF:oxidoreductase activity;
BAB_RS29825	-	GbsR/MarR family transcriptional regulator	0.724	-0.466190187	1.63121E-05	0.000557561	no	down	mRNA	396.52	360.37	387.68	250.98	283.06	253.75	381.523	262.596667	COG1510	K:Transcription	GO:0006351;GO:0006351;GO:0003700;GO:0003677;GO:0003677;GO:00	BP:transcription, DNA-templated;BP:transcription, DNA-templated;MF:transcription factor activity, sequence-specific DNA binding;MF:DNA binding;MF:DNA binding;MF:transcription factor activity, sequence-specific DNA binding;
BAB_RS18680	-	transcriptional regulator	0.61	-0.712841374	1.6784E-05	0.000561739	no	down	mRNA	142.24	128.9	126.37	65.98	78.13	85.93	132.503	76.68	COG1309	E:Amino acid transport and metabolism	GO:0006355;GO:0045892;GO:0006351;GO:0019285;GO:0019285;GO:00	BP:regulation of transcription, DNA-templated;BP:negative regulation of transcription, DNA-templated;BP:transcription, DNA-templated;BP:glycine betaine biosynthetic process from choline;BP:glycine betaine biosynthetic process from choline;BP:negative regulation of transcription, DNA-templated;BP:transcription, DNA-templated;BP:regulation of transcription, DNA-templated;BP:glycine betaine biosynthetic process from choline;BP:transcription, DNA-templated;MF:transcription factor activity, sequence-specific DNA binding;MF:DNA binding;MF:DNA binding;MF:transcription factor activity, sequence-specific DNA binding;MF:DNA binding;
BAB_RS24470	-	ABC transporter ATP-binding protein	0.684	-0.548677038	1.98081E-05	0.000630134	no	down	mRNA	77.21	75.48	74.63	47.65	52	47.33	75.7733	48.9933333	COG0410	E:Amino acid transport and metabolism	GO:0016887;GO:0005524;GO:0015658;GO:0005524;GO:0016887;GO:00	MF:ATPase activity;MF:ATP binding;MF:branched-chain amino acid transmembrane transporter activity;MF:ATP binding;MF:ATPase activity;MF:branched-chain amino acid transmembrane transporter activity;
BAB_RS30745	-	ABC transporter substrate-binding protein	0.721	-0.472461804	1.96649E-05	0.000638217	no	down	mRNA	531.59	468.22	482.95	350.97	338.52	317.85	494.253	335.78	COG4213	G:Carbohydrate transport and metabolism	GO:0008643	BP:carbohydrate transport;
BAB_RS25915	-	PTS fructose transporter subunit IIA	0.705	-0.503422716	2.21317E-05	0.000690381	no	down	mRNA	677.3	559.97	538.73	392.87	395.13	411.24	592	399.746667	COG2893	G:Carbohydrate transport and metabolism	GO:0009401;GO:0009401;GO:0009401;GO:0016021;GO:0016021;GO:00	BP:phosphoenolpyruvate-dependent sugar phosphotransferase system;BP:phosphoenolpyruvate-dependent sugar phosphotransferase system;BP:phosphoenolpyruvate-dependent sugar phosphotransferase system;CC:integral component of membrane;CC:integral component of membrane;CC:integral component of membrane;MF:D-glucosamine PTS permease activity;MF:protein-;MF:D-glucosamine PTS permease activity;MF:protein-;
BAB_RS28860	-	MurR/RpiR family transcriptional regulator	0.6	-0.738129948	2.3247E-05	0.000698061	no	down	mRNA	34.8	28.85	31.71	19.23	16.8	17.85	31.7867	17.96	COG1737	G:Carbohydrate transport and metabolism	GO:1901135;GO:0006351;GO:1901135;GO:0006351;GO:0097367;GO:00	BP:carbohydrate derivative metabolic process;BP:transcription, DNA-templated;BP:carbohydrate derivative metabolic process;BP:transcription, DNA-templated;MF:carbohydrate derivative binding;MF:DNA binding;MF:transcription factor activity, sequence-specific DNA binding;MF:carbohydrate derivative binding;MF:transcription factor activity, sequence-specific DNA binding;MF:DNA binding;
BAB_RS24485	-	branched-chain amino acid ABC transporter permease	0.707	-0.499954732	2.41635E-05	0.000718864	no	down	mRNA	178.63	153.76	154.42	107.75	110.5	106.21	162.27	108.153333	COG0559	E:Amino acid transport and metabolism	GO:0016021;GO:0005886;GO:0022857	CC:integral component of membrane;CC:plasma membrane;MF:transmembrane transporter activity;
BAB_RS27800	-	sugar ABC transporter ATP-binding protein	0.7	-0.514310566	2.93274E-05	0.000833885	no	down	mRNA	42.45	37.31	38.44	26.85	26.13	24.79	39.4	25.9233333	COG1129	G:Carbohydrate transport and metabolism	GO:0008643;GO:0005524;GO:0016887;GO:0016887;GO:0005524	BP:carbohydrate transport;MF:ATP binding;MF:ATPase activity;MF:ATPase activity;MF:ATP binding;
BAB_RS28820	-	iron ABC transporter substrate-binding protein	0.654	-0.612818683	3.05494E-05	0.000853525	no	down	mRNA	74.57	62.19	65.83	40.72	45.68	38.04	67.53	41.48	COG1840	E:Amino acid transport and metabolism	------	------
BAB_RS16400	-	response regulator	0.775	-0.36790567	3.37457E-05	0.000926709	no	down	mRNA	1285.72	1122.46	1164.69	880.41	857.12	920.12	1190.96	885.883333	COG0784	T:Signal transduction mechanisms	GO:0000160;GO:0005622	BP:phosphorelay signal transduction system;CC:intracellular;
BAB_RS17425	-	NAD-dependent dihydropyrimidine dehydrogenase subunit PreA	0.707	-0.500935278	3.52811E-05	0.000960663	no	down	mRNA	27.33	25.09	28.56	17.11	17.47	19.33	26.9933	17.97	COG1146	C:Energy production and conversion	GO:0005737;GO:0005737;GO:0005737;GO:0016627;GO:0016627;GO:00	CC:cytoplasm;CC:cytoplasm;CC:cytoplasm;MF:oxidoreductase activity, acting on the CH-CH group of donors;MF:oxidoreductase activity, acting on the CH-CH group of donors;MF:oxidoreductase activity, acting on the CH-CH group of donors;MF:carbohydrate binding;
BAB_RS27765	-	hypothetical protein	0.769	-0.378528909	4.03303E-05	0.001062142	no	down	mRNA	156.72	153.64	178.12	120.02	117	117.3	162.827	118.106667	COG3802	S:Function unknown	GO:0008152;GO:0008643;GO:0008643;GO:0008152;GO:0003824;GO:00	BP:metabolic process;BP:carbohydrate transport;BP:carbohydrate transport;BP:metabolic process;MF:catalytic activity;MF:catalytic activity;
BAB_RS19925	-	biotin--[acetyl-CoA-carboxylase] ligase	0.749	-0.416926939	4.32965E-05	0.001121868	no	down	mRNA	113.66	109.91	123.51	84.06	84.31	77.02	115.693	81.7966667	COG0340	H:Coenzyme transport and metabolism	GO:0006464;GO:0006464;GO:0004077;GO:0004077;GO:0004111	BP:cellular protein modification process;BP:cellular protein modification process;MF:biotin-[acetyl-CoA-carboxylase] ligase activity;MF:biotin-[acetyl-CoA-carboxylase] ligase activity;MF:creatine kinase activity;
BAB_RS23685	-	Cro/CI family transcriptional regulator	0.803	-0.316694823	4.89836E-05	0.00121065	no	down	mRNA	356.45	342.03	383.43	269.97	280.27	267.99	360.637	272.743333	COG3800;COG1396	E:Amino acid transport and metabolism;	GO:0006355;GO:0043565	BP:regulation of transcription, DNA-templated;MF:sequence-specific DNA binding;
BAB_RS17655	-	ferredoxin--NADP reductase	0.677	-0.563460041	5.17174E-05	0.001230873	no	down	mRNA	66.28	63.42	73.16	41.2	39.13	49.27	67.62	43.2	COG1018	C:Energy production and conversion	GO:0004324	MF:ferredoxin-NADP+ reductase activity;
BAB_RS18395	-	DUF721 domain-containing protein	0.743	-0.429156289	5.13797E-05	0.001231962	no	down	mRNA	71.83	65.76	73.52	46.94	50.1	51.04	70.37	49.36	COG5389	S:Function unknown	------	------
BAB_RS29285	-	endonuclease V	0.703	-0.509041331	5.10053E-05	0.00123218	no	down	mRNA	107.55	95.02	99.06	66.37	60.58	72.77	100.543	66.5733333	ENOG4111P9T	L:Replication, recombination and repair	GO:0004519;GO:0004519;GO:0033892	MF:endonuclease activity;MF:endonuclease activity;MF:deoxyribonucleas;
BAB_RS23785	-	sugar ABC transporter permease	0.738	-0.439164778	5.35466E-05	0.001237734	no	down	mRNA	183.5	179.23	178.26	116.06	134.65	125.97	180.33	125.56	COG1172	G:Carbohydrate transport and metabolism	GO:0016021;GO:0005886;GO:0022857	CC:integral component of membrane;CC:plasma membrane;MF:transmembrane transporter activity;
BAB_RS20555	-	transporter	0.532	-0.909762266	5.26458E-05	0.001243758	no	down	mRNA	13.39	13.35	12.98	7.26	6.91	5.72	13.24	6.63	COG2067	I:Lipid transport and metabolism	------	------
BAB_RS19450	-	L-lactate permease	0.715	-0.483828138	6.00418E-05	0.001339684	no	down	mRNA	21.28	18.48	21.09	13.03	14.26	13.54	20.2833	13.61	COG1620	C:Energy production and conversion	GO:0005887;GO:0015129	CC:integral component of plasma membrane;MF:lactate transmembrane transporter activity;
BAB_RS30930	fabG	3-oxoacyl-ACP reductase	0.775	-0.36740079	6.38042E-05	0.001404129	no	down	mRNA	505.52	436.17	452.78	333.53	344.87	343.74	464.823	340.713333	COG1028	J:Translation, ribosomal structure and biogenesis	GO:0004316;GO:0102132;GO:0102131	MF:3-oxoacyl-[acyl-carrier-protein] reductas;MF:3-oxo-pimeloyl-[acp] methyl ester reductase activity;MF:old GO;
BAB_RS28740	-	proline/glycine betaine ABC transporter ATP-binding protein ProV	0.752	-0.41159121	6.90917E-05	0.001460471	no	down	mRNA	162.01	154.72	152.83	106.09	109.03	118.08	156.52	111.066667	COG4175	S:Function unknown	GO:0031460;GO:0031460;GO:0016020;GO:0016020;GO:0016887;GO:00	BP:glycine betaine transport;BP:glycine betaine transport;CC:membrane;CC:membrane;MF:ATPase activity;MF:ATP binding;MF:ATP binding;MF:ATPase activity;
BAB_RS29340	-	NUDIX hydrolase	0.751	-0.412895973	7.19764E-05	0.001501689	no	down	mRNA	138.58	116.59	136.72	93.75	93.9	90.39	130.63	92.68	COG0494	G:Carbohydrate transport and metabolism	GO:0016787	MF:hydrolase activity;
BAB_RS28865	-	inositol 2-dehydrogenase	0.551	-0.859871305	7.1847E-05	0.001508788	no	down	mRNA	18.78	16.14	19.02	8.83	7.99	11.33	17.98	9.38333333	COG0673	G:Carbohydrate transport and metabolism	GO:0050112;GO:0016491;GO:0050112;GO:0016491	MF:inositol 2-dehydrogenase activity;MF:oxidoreductase activity;MF:inositol 2-dehydrogenase activity;MF:oxidoreductase activity;
BAB_RS21095	-	HlyD family secretion protein	0.69	-0.535250613	8.15041E-05	0.001636705	no	down	mRNA	87.77	93.53	89.98	53.52	61.28	63.17	90.4267	59.3233333	COG1566	V:Defense mechanisms	------	------
BAB_RS26200	-	rRNA maturation RNase YbeY	0.708	-0.498053624	8.06204E-05	0.001639451	no	down	mRNA	500.2	406.88	399.18	297.94	296.6	281.44	435.42	291.993333	COG0319	S:Function unknown	GO:0006364;GO:0006364;GO:0006364;GO:0005737;GO:0005737;GO:00	BP:rRNA processing;BP:rRNA processing;BP:rRNA processing;CC:cytoplasm;CC:cytoplasm;CC:cytoplasm;MF:endoribonuclease activity;MF:metalloendopeptidase activity;MF:zinc ion binding;MF:endoribonuclease activity;MF:kinase activity;MF:metalloendopeptidase activity;MF:zinc ion binding;MF:endonuclease activity;MF:metal ion binding;MF:metalloendopeptidase activity;
BAB_RS26760	-	ketohydroxyglutarate aldolase	0.764	-0.389003519	8.35704E-05	0.001667775	no	down	mRNA	84.89	80.79	87.07	60.99	62.09	59.49	84.25	60.8566667	COG0800	G:Carbohydrate transport and metabolism	GO:0008152;GO:0008152;GO:0016829;GO:0015020;GO:0016829	BP:metabolic process;BP:metabolic process;MF:lyase activity;MF:glucuronosyltransferase activity;MF:lyase activity;
BAB_RS23250	-	two-component sensor histidine kinase	0.725	-0.464788779	8.99234E-05	0.001761731	no	down	mRNA	77.94	76.47	72.05	48.22	52.29	54.32	75.4867	51.61	ENOG410XTWD	T:Signal transduction mechanisms	GO:0016021;GO:0005622;GO:0016021;GO:0005622;GO:0005524;GO:00	CC:integral component of membrane;CC:intracellular;CC:integral component of membrane;CC:intracellular;MF:ATP binding;MF:phosphorelay sensor kinase activity;MF:phosphorelay sensor kinase activity;MF:ATP binding;
BAB_RS18265	-	cytochrome c oxidase subunit II	0.799	-0.323921029	9.24367E-05	0.001789151	no	down	mRNA	977.53	901.8	1004.2	711.52	765.91	697.38	961.177	724.936667	COG1622	S:Function unknown	GO:0016021;GO:0005886;GO:0070469;GO:0016021;GO:0005886;GO:00	CC:integral component of membrane;CC:plasma membrane;CC:respiratory chain;CC:integral component of membrane;CC:plasma membrane;CC:respiratory chain;MF:copper ion binding;MF:cytochrome-c oxidase activity;MF:hydrolase activity;MF:copper ion binding;MF:cytochrome-c oxidase activity;
BAB_RS22305	-	acyl-CoA dehydrogenase	0.664	-0.590251638	9.41086E-05	0.001789178	no	down	mRNA	116.06	111.83	127.46	63.7	72.28	86.89	118.45	74.29	COG1960	H:Coenzyme transport and metabolism	GO:0006629;GO:0006629;GO:0003995;GO:0050660;GO:0003995;GO:00	BP:lipid metabolic process;BP:lipid metabolic process;MF:acyl-CoA dehydrogenase activity;MF:flavin adenine dinucleotide binding;MF:acyl-CoA dehydrogenase activity;MF:flavin adenine dinucleotide binding;MF:butyryl-CoA dehydrogenase activity;
BAB_RS22340	-	hypothetical protein	0.706	-0.502866083	9.87386E-05	0.001855246	no	down	mRNA	128.4	102.97	110.8	78.71	70.99	78.21	114.057	75.97	ENOG410XXSD;ENOG410Y1F3	S:Function unknown;	------	------
BAB_RS22725	-	hypothetical protein	0.642	-0.638344935	0.000105023	0.001939306	no	down	mRNA	37.09	35.4	40.98	22.23	20.73	26.3	37.8233	23.0866667	COG4321	S:Function unknown	GO:0016740	MF:transferase activity;
BAB_RS29750	-	3-methyl-2-oxobutanoate dehydrogenase (2-methylpropanoyl-transferring) subunit alpha	0.782	-0.354993552	0.000107298	0.001947728	no	down	mRNA	1599.71	1496.14	1567.12	1078.46	1185.98	1174.2	1554.32	1146.21333	COG1071	C:Energy production and conversion	GO:0009083;GO:0003863;GO:0003826	BP:branched-chain amino acid catabolic process;MF:3-methyl-2-oxobutanoate dehydrogenas;MF:alpha-ketoacid dehydrogenase activity;
BAB_RS26795	-	hypothetical protein	0.644	-0.635792248	0.000119818	0.002103687	no	down	mRNA	59.56	67.65	65.61	37.97	37.59	43.4	64.2733	39.6533333	ENOG410Y75U	S:Function unknown	GO:0016021	CC:integral component of membrane;
BAB_RS20380	-	ribonuclease E/G	0.786	-0.347086366	0.000123454	0.002121166	no	down	mRNA	314.58	302.74	299.61	225.05	223.88	230.13	305.643	226.353333	COG1530	J:Translation, ribosomal structure and biogenesis	GO:0008033;GO:0006364;GO:0006402;GO:0006402;GO:0006364;GO:00	BP:tRNA processing;BP:rRNA processing;BP:mRNA catabolic process;BP:mRNA catabolic process;BP:rRNA processing;BP:tRNA processing;CC:cytoplasm;CC:cytoplasmic side of plasma membrane;CC:cytoplasmic side of plasma membrane;MF:ribonuclease E activity;MF:magnesium ion binding;MF:endoribonuclease activity;MF:zinc ion binding;MF:RNA binding;MF:endoribonuclease activity;MF:magnesium ion binding;MF:ribonuclease E activity;MF:RNA binding;MF:zinc ion binding;
BAB_RS33465	-	3-octaprenyl-4-hydroxybenzoate carboxy-lyase	0.633	-0.660347439	0.000123168	0.002127626	no	down	mRNA	41.62	47.72	44.22	22.76	30.51	26.51	44.52	26.5933333	COG0043	H:Coenzyme transport and metabolism	GO:0016831	MF:carboxy-lyase activity;
BAB_RS32315	-	hypothetical protein	0.673	-0.571340832	0.000127321	0.002175964	no	down	mRNA	16.83	15.74	22.69	11.57	12.07	11.22	18.42	11.62	COG1309	K:Transcription	------	------
BAB_RS26480	-	branched-chain amino acid ABC transporter substrate-binding protein	0.691	-0.532948334	0.000130445	0.002194347	no	down	mRNA	35.69	32.51	33.42	21.63	20.04	24.6	33.8733	22.09	COG0683	S:Function unknown	GO:0006865	BP:amino acid transport;
BAB_RS24785	-	hypothetical protein	0.782	-0.354405143	0.00014019	0.002309892	no	down	mRNA	63.27	56.36	59.57	43.41	43.64	44.86	59.7333	43.97	ENOG410XRTK	S:Function unknown	------	------
BAB_RS20740	-	hypothetical protein	0.718	-0.477588859	0.000139742	0.002314392	no	down	mRNA	54.14	46.18	47.14	32.96	31.06	35.63	49.1533	33.2166667	------	------	------	------
BAB_RS18795	-	hypothetical protein	0.661	-0.596497093	0.000152052	0.002442711	no	down	mRNA	71.64	76.54	69.41	46.45	41.67	49.42	72.53	45.8466667	ENOG410ZQU6	S:Function unknown	------	------
BAB_RS20575	-	ABC transporter substrate-binding protein	0.811	-0.302573759	0.000159086	0.002517943	no	down	mRNA	712.93	641.32	657.58	522.96	500.66	525.6	670.61	516.406667	COG0834	I:Lipid transport and metabolism	------	------
BAB_RS28825	-	iron ABC transporter permease	0.717	-0.480266753	0.000158725	0.002524665	no	down	mRNA	13.59	13.59	14.11	9.86	9.41	8.59	13.7633	9.28666667	COG1178	E:Amino acid transport and metabolism	GO:0055085;GO:0016021;GO:0005886	BP:transmembrane transport;CC:integral component of membrane;CC:plasma membrane;
BAB_RS28960	-	sugar ABC transporter permease	0.722	-0.469960633	0.000165597	0.00255799	no	down	mRNA	242.5	197.06	196.72	141.1	141.62	150.38	212.093	144.366667	ENOG410XRGU	G:Carbohydrate transport and metabolism	GO:0055085;GO:0016021;GO:0005886	BP:transmembrane transport;CC:integral component of membrane;CC:plasma membrane;
BAB_RS21530	-	lipid-A-disaccharide synthase	0.773	-0.371612659	0.000165259	0.002565114	no	down	mRNA	52.46	47.04	52.44	35.38	36.43	38.82	50.6467	36.8766667	COG0763	M:Cell wall/membrane/envelope biogenesis	GO:0009245;GO:0009245;GO:0016798;GO:0008915;GO:0008915	BP:lipid A biosynthetic process;BP:lipid A biosynthetic process;MF:hydrolase activity, acting on glycosyl bonds;MF:lipid-A-disaccharide synthase activity;MF:lipid-A-disaccharide synthase activity;
BAB_RS28775	-	N-formylglutamate amidohydrolase	0.721	-0.472100682	0.000176073	0.002706813	no	down	mRNA	41.33	37.65	45.02	28.58	27.07	28.96	41.3333	28.2033333	COG3931	C:Energy production and conversion	GO:0016787	MF:hydrolase activity;
BAB_RS18665	-	AraC family transcriptional regulator	0.763	-0.38939364	0.000181208	0.002772486	no	down	mRNA	77.75	74.7	78.26	58.6	52.5	55.5	76.9033	55.5333333	ENOG4111NRR	K:Transcription	------	------
BAB_RS20225	-	SMC-Scp complex subunit ScpB	0.812	-0.301220385	0.00018422	0.00277887	no	down	mRNA	149.9	139.69	154.76	112.12	114.75	113.89	148.117	113.586667	COG1386	K:Transcription	GO:0051301;GO:0051304;GO:0051304;GO:0051301;GO:0005737;GO:00	BP:cell division;BP:chromosome separation;BP:chromosome separation;BP:cell division;CC:cytoplasm;CC:cytoplasm;
BAB_RS17300	-	hypothetical protein	0.758	-0.399976535	0.000183401	0.002779563	no	down	mRNA	175.8	164.9	165.68	114.52	129.37	122.49	168.793	122.126667	ENOG410ZAU1	S:Function unknown	------	------
BAB_RS23755	-	dihydroxyacetone kinase	0.603	-0.730393063	0.000182954	0.002785926	no	down	mRNA	19.97	16.58	20.5	10.02	12.54	9.63	19.0167	10.73	COG2376	G:Carbohydrate transport and metabolism	------	------
BAB_RS27760	-	hypothetical protein	0.764	-0.38913445	0.000185557	0.002785949	no	down	mRNA	823.4	670.96	669.36	548.37	520.32	538.81	721.24	535.833333	------	------	GO:0008643	BP:carbohydrate transport;
BAB_RS30350	-	disulfide bond formation protein B	0.757	-0.401447835	0.000190899	0.00282654	no	down	mRNA	118.96	113.09	107.24	77.92	80.35	84.49	113.097	80.92	COG1495	F:Nucleotide transport and metabolism	GO:0016021;GO:0005886;GO:0015035	CC:integral component of membrane;CC:plasma membrane;MF:protein disulfide oxidoreductase activity;
BAB_RS33020	-	hypothetical protein	0.737	-0.44121424	0.000191817	0.0028271	no	down	mRNA	232.59	230.43	227.85	152.27	155.47	181.01	230.29	162.916667	------	------	------	------
BAB_RS18060	-	cold-shock protein	0.78	-0.357585951	0.000193365	0.002836901	no	down	mRNA	51897.6	48418.9	44990.6	36009.02	38710.88	37353.21	48435.7	37357.7033	COG1278	K:Transcription	GO:0006355;GO:0006355;GO:0005737;GO:0005737;GO:0003677;GO:00	BP:regulation of transcription, DNA-templated;BP:regulation of transcription, DNA-templated;CC:cytoplasm;CC:cytoplasm;MF:DNA binding;MF:DNA binding;
BAB_RS30630	-	U32 family peptidase	0.73	-0.454221357	0.000195372	0.002853317	no	down	mRNA	309.76	316.7	291.76	189.3	229.05	215.89	306.073	211.413333	COG0826	O:Posttranslational modification, protein turnover,	GO:0008233	MF:peptidase activity;
BAB_RS20900	-	STAS domain-containing protein	0.781	-0.355692779	0.000209081	0.003012458	no	down	mRNA	101.05	94.74	96.49	67.29	72.91	75.15	97.4267	71.7833333	COG0767	Q:Secondary metabolites biosynthesis, transport a	GO:0043190	CC:ATP-binding cassett;
BAB_RS17390	-	efflux RND transporter periplasmic adaptor subunit	0.796	-0.329092582	0.000215656	0.003065946	no	down	mRNA	115	114.64	115.38	88.11	83.74	87.46	115.007	86.4366667	COG0845	M:Cell wall/membrane/envelope biogenesis	GO:0055085;GO:0016020;GO:0005215	BP:transmembrane transport;CC:membrane;MF:transporter activity;
BAB_RS33190	-	hypothetical protein	0.705	-0.504941116	0.000214353	0.003074628	no	down	mRNA	772.69	705.12	565.78	484.91	447.54	492.43	681.197	474.96	------	------	------	------
BAB_RS30410	-	hypothetical protein	0.804	-0.315619162	0.000219359	0.00310485	no	down	mRNA	137.97	122.38	130.7	101.4	100.05	94.15	130.35	98.5333333	------	------	GO:0016021	CC:integral component of membrane;
BAB_RS30740	-	sugar ABC transporter ATP-binding protein	0.703	-0.507688056	0.000225303	0.003161132	no	down	mRNA	65.36	51.45	56.17	39.63	39.75	34.79	57.66	38.0566667	COG1129	G:Carbohydrate transport and metabolism	GO:0005524;GO:0016887	MF:ATP binding;MF:ATPase activity;
BAB_RS23440	-	N-acetyltransferase	0.676	-0.564427605	0.000226676	0.003166568	no	down	mRNA	72.02	65.54	68.44	49.52	45.14	36.46	68.6667	43.7066667	COG3153	S:Function unknown	GO:0016740;GO:0016746	MF:transferase activity;MF:transferase activity, transferring acyl groups;
BAB_RS23740	-	patatin	0.736	-0.442797156	0.000229374	0.003176636	no	down	mRNA	89.83	84.42	78.14	58.48	61.53	54.96	84.13	58.3233333	ENOG410Z2XG	S:Function unknown	GO:0016042;GO:0016787	BP:lipid catabolic process;MF:hydrolase activity;
BAB_RS25990	-	YdcF family protein	0.796	-0.329685573	0.00023103	0.003185829	no	down	mRNA	279.83	257.55	274.61	210.77	209.79	191.42	270.663	203.993333	COG1434	E:Amino acid transport and metabolism	------	------
BAB_RS19825	-	hypothetical protein	0.795	-0.33040414	0.000232545	0.003193023	no	down	mRNA	297.63	292.35	304.21	222.34	232.84	226.2	298.063	227.126667	ENOG41124QQ	S:Function unknown	------	------
BAB_RS29265	-	DNA polymerase III subunit epsilon	0.759	-0.398564657	0.000238419	0.003245929	no	down	mRNA	54.49	48.65	51.84	37.7	37.03	36.19	51.66	36.9733333	COG0847	S:Function unknown	GO:0006260;GO:0006260;GO:0043885;GO:0003677;GO:0003887;GO:00	BP:DNA replication;BP:DNA replication;MF:carbon-monoxide dehydrogenas;MF:DNA binding;MF:DNA-directed DNA polymerase activity;MF:exonuclease activity;MF:DNA-directed DNA polymerase activity;MF:DNA binding;
BAB_RS22285	-	bifunctional adenosylcobinamide kinase/adenosylcobinamide-phosphate guanylyltransferase	0.696	-0.522891158	0.000248559	0.003286503	no	down	mRNA	107.52	127.57	122.46	73.45	82.92	81.96	119.183	79.4433333	COG2087	H:Coenzyme transport and metabolism	GO:0009236;GO:0008820;GO:0043752;GO:0005524;GO:0005525	BP:cobalamin biosynthetic process;MF:cobinamide phosphate guanylyltransferase activity;MF:adenosylcobinamide kinase activity;MF:ATP binding;MF:GTP binding;
BAB_RS29745	-	alpha-ketoacid dehydrogenase subunit beta	0.816	-0.292783544	0.000256175	0.003305578	no	down	mRNA	765.09	730.03	747.24	549.14	596.52	582.98	747.453	576.213333	COG0022	C:Energy production and conversion	GO:0008152;GO:0003824;GO:0003863	BP:metabolic process;MF:catalytic activity;MF:3-methyl-2-oxobutanoate dehydrogenas;
BAB_RS17715	-	ABC transporter substrate-binding protein	0.687	-0.541865394	0.000246579	0.003314885	no	down	mRNA	27.55	22.07	27.66	17.2	15.15	17.63	25.76	16.66	COG4663	Q:Secondary metabolites biosynthesis, transport a	GO:0055085;GO:0030288;GO:0046872	BP:transmembrane transport;CC:outer membrane-bounded periplasmic space;MF:metal ion binding;
BAB_RS29820	-	thiol reductant ABC exporter subunit CydD	0.8	-0.322656248	0.000267605	0.003425556	no	down	mRNA	151.61	150.65	155.35	106.62	121.16	117.13	152.537	114.97	COG4988	V:Defense mechanisms	GO:0042883;GO:0042883;GO:0016021;GO:0016021;GO:0005524;GO:00	BP:cysteine transport;BP:cysteine transport;CC:integral component of membrane;CC:integral component of membrane;MF:ATP binding;MF:ATPase activity, coupled to transmembrane movement of substances;MF:ATPase activity, coupled to transmembrane movement of substances;MF:ATP binding;
BAB_RS28715	-	ATP-binding protein	0.734	-0.447008409	0.000308325	0.003839717	no	down	mRNA	29.16	26.17	28.09	19.54	18.31	19.87	27.8067	19.24	COG3839;COG3842	S:Function unknown;	GO:0043190;GO:0043190;GO:0043190;GO:0005524;GO:0016887;GO:00	CC:ATP-binding cassett;CC:ATP-binding cassett;CC:ATP-binding cassett;MF:ATP binding;MF:ATPase activity;MF:transmembrane transporter activity;MF:transmembrane transporter activity;MF:ATP binding;MF:ATPase activity;MF:transmembrane transporter activity;MF:ATPase activity;MF:ATP binding;
BAB_RS24455	-	branched chain amino acid ABC transporter substrate-binding protein	0.799	-0.323131429	0.000324425	0.003948403	no	down	mRNA	696.48	656.15	661.14	479.02	504.25	534.89	671.257	506.053333	COG0683	C:Energy production and conversion	GO:0006865;GO:0006865;GO:0016021	BP:amino acid transport;BP:amino acid transport;CC:integral component of membrane;
BAB_RS21535	-	DUF1009 domain-containing protein	0.771	-0.376089903	0.000341922	0.004114592	no	down	mRNA	83.89	88.09	84.06	64.13	60.92	61.79	85.3467	62.28	COG3494	S:Function unknown	GO:0016779	MF:nucleotidyltransferase activity;
BAB_RS21780	-	membrane protein	0.767	-0.383581439	0.000349222	0.004125184	no	down	mRNA	150.85	153.35	166.5	106.76	111.29	124.9	156.9	114.316667	COG4764	S:Function unknown	------	------
BAB_RS23490	-	MarR family transcriptional regulator	0.84	-0.251010846	0.000370735	0.004284795	no	down	mRNA	2272.88	2142.68	2223.52	1790.78	1807.66	1776.1	2213.03	1791.51333	ENOG41122MC;COG1846	K:Transcription;	GO:0006351;GO:0006351;GO:0003700;GO:0003677;GO:0003677;GO:00	BP:transcription, DNA-templated;BP:transcription, DNA-templated;MF:transcription factor activity, sequence-specific DNA binding;MF:DNA binding;MF:DNA binding;MF:transcription factor activity, sequence-specific DNA binding;
BAB_RS22900	-	molecular chaperone DjlA	0.758	-0.400119464	0.00037291	0.004294475	no	down	mRNA	69.31	69.99	65.97	46.53	50.16	50.56	68.4233	49.0833333	COG1076	O:Posttranslational modification, protein turnover,	------	------
BAB_RS21110	-	alcohol dehydrogenase	0.805	-0.313412841	0.000378979	0.004348786	no	down	mRNA	235.2	230.59	260.08	168.06	187.13	194.74	241.957	183.31	COG1064	E:Amino acid transport and metabolism	GO:0004714;GO:0008270;GO:0016491;GO:0016491;GO:0008270	MF:transmembrane receptor protein tyrosine kinase activity;MF:zinc ion binding;MF:oxidoreductase activity;MF:oxidoreductase activity;MF:zinc ion binding;
BAB_RS16220	-	hypothetical protein	0.651	-0.619468356	0.000388464	0.004426005	no	down	mRNA	88.68	81.69	83.89	57.48	52.7	50.26	84.7533	53.48	------	------	GO:0016021	CC:integral component of membrane;
BAB_RS23780	-	sugar ABC transporter substrate-binding protein	0.76	-0.396235077	0.000393988	0.004441694	no	down	mRNA	375.22	326.88	341.37	219.99	257.54	271.01	347.823	249.513333	COG1879	G:Carbohydrate transport and metabolism	------	------
BAB_RS18360	-	EAL domain-containing protein	0.793	-0.334699653	0.000410525	0.004579918	no	down	mRNA	69.33	68.28	66.99	53.15	49.25	50.55	68.2	50.9833333	ENOG410XNMH;COG4943	S:Function unknown;T:Signal transduction mechar	------	------
BAB_RS29740	-	2-oxo acid dehydrogenase subunit E2	0.836	-0.258625906	0.000417464	0.004641222	no	down	mRNA	514.89	504.31	549.25	394.27	427.56	414.43	522.817	412.086667	COG0508	S:Function unknown	GO:0008152;GO:0016746	BP:metabolic process;MF:transferase activity, transferring acyl groups;
BAB_RS22380	-	sulfate ABC transporter permease subunit CysW	0.617	-0.697403158	0.000423824	0.004679544	no	down	mRNA	90.49	83.76	79.98	48.49	38.75	60.94	84.7433	49.3933333	COG4208	S:Function unknown	GO:0016021;GO:0016021;GO:0015419;GO:0015419	CC:integral component of membrane;CC:integral component of membrane;MF:ATPase-coupled sulfate transmembrane transporter activity;MF:ATPase-coupled sulfate transmembrane transporter activity;
BAB_RS20135	-	amino acid ABC transporter substrate-binding protein	0.697	-0.520349486	0.000428242	0.004696042	no	down	mRNA	100.83	78.76	76.33	52.74	57.02	58.03	85.3067	55.93	COG0834	P:Inorganic ion transport and metabolism	GO:0071705;GO:0016020;GO:0030288;GO:0016020;GO:0004970;GO:00	BP:nitrogen compound transport;CC:membrane;CC:outer membrane-bounded periplasmic space;CC:membrane;MF:ionotropic glutamate receptor activity;MF:ionotropic glutamate receptor activity;
BAB_RS26790	-	N-acetyltransferase	0.799	-0.323336901	0.000430935	0.004709507	no	down	mRNA	108.78	97.75	107.05	77.73	81.9	77.54	104.527	79.0566667	COG1670	P:Inorganic ion transport and metabolism	GO:0016740	MF:transferase activity;
BAB_RS31135	-	lysoplasmalogenase	0.653	-0.614486777	0.000456701	0.004940677	no	down	mRNA	18.53	18.48	18.93	11.07	11.28	12.34	18.6467	11.5633333	COG3714	S:Function unknown	GO:0016021	CC:integral component of membrane;
BAB_RS24655	-	pyridoxal kinase PdxY	0.787	-0.345970873	0.0004646	0.005009263	no	down	mRNA	90.22	94.07	105.76	69.3	76.01	70.28	96.6833	71.8633333	COG2240	H:Coenzyme transport and metabolism	GO:0009443;GO:0009443;GO:0009443;GO:0016829;GO:0005524;GO:00	BP:pyridoxal 5'-phosphate salvage;BP:pyridoxal 5'-phosphate salvage;BP:pyridoxal 5'-phosphate salvage;MF:lyase activity;MF:ATP binding;MF:pyridoxal kinase activity;MF:ATP binding;MF:pyridoxal kinase activity;MF:lyase activity;MF:ATP binding;MF:pyridoxal kinase activity;
BAB_RS22590	-	endonuclease	0.803	-0.316568316	0.00047019	0.00501901	no	down	mRNA	90.56	86.76	86.07	64.31	66.99	68.31	87.7967	66.5366667	ENOG41102QR	S:Function unknown	GO:0004519;GO:0004527	MF:endonuclease activity;MF:exonuclease activity;
BAB_RS30480	-	ABC transporter ATP-binding protein	0.703	-0.50804832	0.000472299	0.005024825	no	down	mRNA	25.61	29.78	31.53	17.78	20.65	19.48	28.9733	19.3033333	COG3842	S:Function unknown	GO:0043190;GO:0043190;GO:0015417;GO:0005524;GO:0005524;GO:00	CC:ATP-binding cassett;CC:ATP-binding cassett;MF:polyamine-transporting ATPase activity;MF:ATP binding;MF:ATP binding;MF:polyamine-transporting ATPase activity;
BAB_RS21615	-	GNAT family N-acetyltransferase	0.667	-0.584500768	0.000554251	0.005671369	no	down	mRNA	21.51	18.08	17.79	12.39	10.58	13.03	19.1267	12	COG3146	S:Function unknown	------	------
BAB_RS24465	-	hypothetical protein	0.706	-0.502026521	0.000561602	0.005710212	no	down	mRNA	129.62	127.73	121.9	80.82	94.47	84.08	126.417	86.4566667	ENOG41125H1	S:Function unknown	GO:0016021	CC:integral component of membrane;
BAB_RS28220	-	IS5/IS1182 family transposase	0.737	-0.440356328	0.000569167	0.005714793	no	down	mRNA	112.63	111.86	107.48	86.78	71.93	73.87	110.657	77.5266667	COG3293	L:Replication, recombination and repair	------	------
BAB_RS15995	-	hypothetical protein	0.652	-0.616032452	0.000567498	0.005733875	no	down	mRNA	152.16	133.49	122.82	101.04	77.98	75.78	136.157	84.9333333	------	------	GO:0016021	CC:integral component of membrane;
BAB_RS21315	-	hypothetical protein	0.724	-0.465513747	0.000566994	0.005746852	no	down	mRNA	250.6	278.74	293.31	173.16	204.5	198.93	274.217	192.196667	------	------	------	------
BAB_RS30755	-	transcriptional regulator	0.719	-0.476793519	0.000586988	0.005857116	no	down	mRNA	24.45	24.83	26.5	16.73	15.6	19.27	25.26	17.2	COG1609	G:Carbohydrate transport and metabolism	GO:0006355;GO:0006351;GO:0006355;GO:0006351;GO:0003677;GO:00	BP:regulation of transcription, DNA-templated;BP:transcription, DNA-templated;BP:regulation of transcription, DNA-templated;BP:transcription, DNA-templated;MF:DNA binding;MF:DNA binding;
BAB_RS28970	-	carbohydrate ABC transporter permease	0.636	-0.653984806	0.000593164	0.005900421	no	down	mRNA	25.14	19.68	25.2	13.61	15.27	13.03	23.34	13.97	COG0395	G:Carbohydrate transport and metabolism	GO:0055085;GO:0008643;GO:0055085;GO:0016021;GO:0005886;GO:00	BP:transmembrane transport;BP:carbohydrate transport;BP:transmembrane transport;CC:integral component of membrane;CC:plasma membrane;CC:integral component of membrane;CC:plasma membrane;
BAB_RS30575	-	MFS transporter	0.776	-0.365354392	0.000600949	0.005959408	no	down	mRNA	662.69	703.56	621.33	463.56	499.18	499.17	662.527	487.303333	COG2223	P:Inorganic ion transport and metabolism	GO:0016021;GO:0015112;GO:0015113	CC:integral component of membrane;MF:nitrate transmembrane transporter activity;MF:nitrite transmembrane transporter activity;
BAB_RS17430	-	FAD-dependent oxidoreductase	0.79	-0.340747223	0.000623696	0.006147039	no	down	mRNA	59.69	54.77	62.59	40.98	42.42	48.29	59.0167	43.8966667	COG0493	E:Amino acid transport and metabolism	GO:0051536;GO:0016491	MF:iron-sulfur cluster binding;MF:oxidoreductase activity;
BAB_RS18040	-	hypothetical protein	0.784	-0.351347001	0.000626403	0.006154845	no	down	mRNA	391.33	357.02	354.56	262.78	256.02	301.94	367.637	273.58	ENOG41128VK	S:Function unknown	GO:0016021	CC:integral component of membrane;
BAB_RS23450	-	OmpW family protein	0.799	-0.324606861	0.000644222	0.006234592	no	down	mRNA	4179.46	3800.78	3801.78	2731.28	3139.95	3049.84	3927.34	2973.69	COG3047	M:Cell wall/membrane/envelope biogenesis	GO:0019867	CC:outer membrane;
BAB_RS30640	-	ABC transporter ATP-binding protein	0.672	-0.57311796	0.000650528	0.006239242	no	down	mRNA	24.82	21.68	19.88	13.67	12.8	15.54	22.1267	14.0033333	COG1116	O:Posttranslational modification, protein turnover,	GO:0005524;GO:0016887;GO:0016887;GO:0005524	MF:ATP binding;MF:ATPase activity;MF:ATPase activity;MF:ATP binding;
BAB_RS21075	-	N-acetyltransferase	0.837	-0.257359317	0.000674628	0.006394042	no	down	mRNA	82.83	75.35	83.28	60.38	65.64	63.94	80.4867	63.32	ENOG4111IIV;COG2897	S:Function unknown;	GO:0016740	MF:transferase activity;
BAB_RS26850	-	glycosyl transferase	0.773	-0.370556791	0.000686107	0.006464699	no	down	mRNA	53.23	51.96	51.43	40.16	35.31	39.02	52.2067	38.1633333	COG0463	M:Cell wall/membrane/envelope biogenesis	GO:0016740	MF:transferase activity;
BAB_RS20905	-	ABC transporter ATP-binding protein	0.825	-0.278256589	0.000690744	0.006470436	no	down	mRNA	160.67	152.03	165.43	117.02	130.18	125.46	159.377	124.22	COG1127	Q:Secondary metabolites biosynthesis, transport a	GO:0016887;GO:0005524;GO:0005524;GO:0016887	MF:ATPase activity;MF:ATP binding;MF:ATP binding;MF:ATPase activity;
BAB_RS31330	-	ABC transporter substrate-binding protein	0.663	-0.593365766	0.000702154	0.006520288	no	down	mRNA	13.38	10.36	11.37	7.16	8.07	6.46	11.7033	7.23	COG0747;ENOG410Y6NK	K:Transcription;S:Function unknown	GO:0055085;GO:0055085;GO:0043190;GO:0043190	BP:transmembrane transport;BP:transmembrane transport;CC:ATP-binding cassett;CC:ATP-binding cassett;
BAB_RS22905	-	hypothetical protein	0.8	-0.32226621	0.000700168	0.006520695	no	down	mRNA	266.35	257.53	276.12	212.83	209.25	186.63	266.667	202.903333	------	------	------	------
BAB_RS30920	-	ABC transporter permease	0.805	-0.313432561	0.000743582	0.006711035	no	down	mRNA	132.07	139.62	145.01	99.7	111.46	106.09	138.9	105.75	COG0601	J:Translation, ribosomal structure and biogenesis	GO:0055085;GO:0016021;GO:0005886	BP:transmembrane transport;CC:integral component of membrane;CC:plasma membrane;
BAB_RS19820	-	hypothetical protein	0.754	-0.407257502	0.000743168	0.006726193	no	down	mRNA	83.56	68.38	72.53	54.22	51.12	54.75	74.8233	53.3633333	------	------	GO:0016021	CC:integral component of membrane;
BAB_RS24605	-	MFS transporter	0.804	-0.314232486	0.000747976	0.006731783	no	down	mRNA	46.63	41.72	44.78	32.05	33.73	35.02	44.3767	33.6	COG2270	G:Carbohydrate transport and metabolism	GO:0016021	CC:integral component of membrane;
BAB_RS17395	-	AcrB/AcrD/AcrF family protein	0.85	-0.234588302	0.000754743	0.006754847	no	down	mRNA	64.56	60.39	65.12	50.45	51.7	49.75	63.3567	50.6333333	COG0841	P:Inorganic ion transport and metabolism	GO:0016021;GO:0022857	CC:integral component of membrane;MF:transmembrane transporter activity;

BAB_RS26490	-	branched-chain amino acid ABC transporter permease	0.649	-0.622870033	0.000764326	0.006802714	no	down	mRNA	8.51	8.31	8.38	4.66	5.21	5.56	8.4	5.14333333	COG4177	S:Function unknown	GO:0016021;GO:0005886;GO:0022857	CC:integral component of membrane;CC:plasma membrane;MF:transmembrane transporter activity;
BAB_RS32575	-	EmrB/QacA family drug resistance transporter	0.695	-0.524117048	0.000763873	0.006817571	no	down	mRNA	26.04	21.84	23.97	12.86	17.09	16.92	23.95	15.6233333	ENOG410XNN3	P:Inorganic ion transport and metabolism	GO:0016021;GO:0005886;GO:0022857	CC:integral component of membrane;CC:plasma membrane;MF:transmembrane transporter activity;
BAB_RS31975	-	hypothetical protein	0.81	-0.304597665	0.000783211	0.00693239	no	down	mRNA	34.93	32.25	35.3	24.89	24.8	28.33	34.16	26.0066667	COG5295;COG5295	U:Intracellular trafficking, secretion, and vesicular	GO:0009405;GO:0019867	BP:pathogenesis;CC:outer membrane;
BAB_RS32910	-	hypothetical protein	0.762	-0.391804314	0.000829297	0.007240571	no	down	mRNA	1035.58	874.5	830.04	723.64	605.56	661.51	913.373	663.57	------	------	------	------
BAB_RS20630	-	GlsB/YeaQ/YmgE family stress response membrane protein	0.843	-0.246874763	0.000863467	0.007457848	no	down	mRNA	635.95	607.14	604.08	483.74	479.14	515.29	615.723	492.723333	COG2261	C:Energy production and conversion	GO:0016021	CC:integral component of membrane;
BAB_RS30905	-	ABC transporter ATP-binding protein	0.807	-0.309891811	0.000912081	0.007835602	no	down	mRNA	91.76	83.73	101.32	70.45	72.97	67.46	92.27	70.2933333	COG4608	J:Translation, ribosomal structure and biogenesis	GO:0015833;GO:0015833;GO:0005524;GO:0016887;GO:0005524;GO:00	BP:peptide transport;BP:peptide transport;MF:ATP binding;MF:ATPase activity;MF:ATP binding;MF:ATPase activity;
BAB_RS28145	-	DUF2291 domain-containing protein	0.814	-0.296390135	0.000920931	0.007890533	no	down	mRNA	230.28	193.03	203.24	157.54	162.08	161.68	208.85	160.433333	COG5618	S:Function unknown	GO:0016021;GO:0016021;GO:0005524	CC:integral component of membrane;CC:integral component of membrane;MF:ATP binding;
BAB_RS22060	-	DUF192 domain-containing protein	0.829	-0.270069392	0.00094549	0.008036665	no	down	mRNA	322.23	304.05	299.74	232.7	252.01	247.28	308.673	243.996667	COG1430	S:Function unknown	------	------
BAB_RS23010	-	hypothetical protein	0.784	-0.351512649	0.000962166	0.008114016	no	down	mRNA	221.81	210.67	196.17	145.08	153.4	167.61	209.55	155.363333	ENOG4111V1M	S:Function unknown	------	------
BAB_RS25300	-	methionine gamma-lyase	0.809	-0.305122989	0.000971787	0.008131124	no	down	mRNA	50.91	46.97	50.01	35.49	36.58	40.64	49.2967	37.57	COG0626	G:Carbohydrate transport and metabolism	GO:0003824;GO:0030170;GO:0003962;GO:0003824;GO:0016829;GO:00	MF:catalytic activity;MF:pyridoxal phosphate binding;MF:cystathionine gamma-synthase activity;MF:catalytic activity;MF:lyase activity;MF:pyridoxal phosphate binding;MF:cystathionine gamma-synthase activity;
BAB_RS19250	-	SOS response-associated peptidase	0.718	-0.476980012	0.000970102	0.008138221	no	down	mRNA	28.94	28.03	29.91	21.13	20.67	17.09	28.96	19.63	COG2135	S:Function unknown	GO:0008233	MF:peptidase activity;
BAB_RS30910	-	ABC transporter ATP-binding protein	0.815	-0.295934965	0.001003506	0.0082886	no	down	mRNA	139.5	141.52	160.71	107.64	120.37	111.79	147.243	113.266667	COG0444	J:Translation, ribosomal structure and biogenesis	GO:0015833;GO:0015833;GO:0005524;GO:0016887;GO:0016887;GO:00	BP:peptide transport;BP:peptide transport;MF:ATP binding;MF:ATPase activity;MF:ATPase activity;MF:ATP binding;
BAB_RS32650	-	hypothetical protein	0.673	-0.571115063	0.001006131	0.008288972	no	down	mRNA	90.39	98.91	115.15	73.26	64.88	60.06	101.483	66.0666667	COG1760	E:Amino acid transport and metabolism	GO:0006094;GO:0006094;GO:0051539;GO:0003941;GO:0003941;GO:00	BP:gluconeogenesis;BP:gluconeogenesis;MF:4 iron, 4 sulfur cluster binding;MF:L-serine ammonia-lyase activity;MF:L-serine ammonia-lyase activity;MF:4 iron, 4 sulfur cluster binding;
BAB_RS21750	-	hypothetical protein	0.816	-0.29342537	0.001013139	0.008325358	no	down	mRNA	208.18	188.53	206.13	142.66	160.72	163.18	200.947	155.52	COG4321	S:Function unknown	GO:0016787	MF:hydrolase activity;
BAB_RS20305	-	DUF2157 domain-containing protein	0.833	-0.262828246	0.001050795	0.008590847	no	down	mRNA	94.45	83.97	92.38	73.48	69.67	69.37	90.2667	70.84	COG4872	S:Function unknown	GO:0016021	CC:integral component of membrane;
BAB_RS21990	-	preprotein translocase subunit SecE	0.833	-0.26391169	0.001109429	0.008978833	no	down	mRNA	1512.94	1273.83	1374.16	1112.21	1112.87	1053.84	1386.98	1092.97333	COG0690	U:Intracellular trafficking, secretion, and vesicular	GO:0009306;GO:0006605;GO:0065002;GO:0043952;GO:0065002;GO:00	BP:protein secretion;BP:protein targeting;BP:intracellular protein transmembrane transport;BP:protein transport by the Sec complex;BP:intracellular protein transmembrane transport;BP:protein secretion;BP:protein targeting;BP:protein transport by the Sec complex;CC:intracellular;CC:integral component of membrane;CC:plasma membrane;CC:integral component of membrane;CC:intracellular;CC:plasma membrane;MF:P-P-bond-hydrolysis-driven protein transmembrane transporter activity;MF:P-P-bond-hydrolysis-driven protein transmembrane transporter activity;
BAB_RS21965	-	50S ribosomal protein L7/L12	0.796	-0.328472535	0.001142002	0.009196125	no	down	mRNA	2631.12	2105.67	2086.6	1758.37	1805.14	1628.21	2274.46	1730.57333	COG0222	J:Translation, ribosomal structure and biogenesis	GO:0006412;GO:0005840;GO:0003735	BP:translation;CC:ribosome;MF:structural constituent of ribosome;
BAB_RS29920	-	sodium:alanine symporter family protein	0.833	-0.26328314	0.00115372	0.009267258	no	down	mRNA	101.66	100.38	121.22	82.75	84.24	86.35	107.753	84.4466667	COG1115	E:Amino acid transport and metabolism	GO:0016021;GO:0005886;GO:0015655	CC:integral component of membrane;CC:plasma membrane;MF:alanin;
BAB_RS29000	-	amino acid ABC transporter ATP-binding protein	0.78	-0.357718422	0.001157367	0.009273364	no	down	mRNA	135.13	120.03	119.38	82.89	94.57	98.83	124.847	92.0966667	COG1126	J:Translation, ribosomal structure and biogenesis	GO:0015424;GO:0005524	MF:amino acid-transporting ATPase activity;MF:ATP binding;
BAB_RS32685	-	hypothetical protein	0.748	-0.419044616	0.001173124	0.009376239	no	down	mRNA	23.67	19.37	26.6	16.57	17.3	14.95	23.2133	16.2733333	------	------	GO:0016021	CC:integral component of membrane;
BAB_RS22400	-	TerC family protein	0.845	-0.242660586	0.001185099	0.009378631	no	down	mRNA	130.04	122.4	126.03	96	101.18	104.09	126.157	100.423333	COG0861	P:Inorganic ion transport and metabolism	GO:0016021;GO:0016021;GO:0050660;GO:0016614;GO:0050660;GO:00	CC:integral component of membrane;CC:integral component of membrane;MF:flavin adenine dinucleotide binding;MF:oxidoreductase activity, acting on CH-OH group of donors;MF:flavin adenine dinucleotide binding;MF:oxidoreductase activity, acting on CH-OH group of donors;
BAB_RS24480	-	high-affinity branched-chain amino acid ABC transporter permease LivM	0.727	-0.459151021	0.001218875	0.009575171	no	down	mRNA	83.85	65.11	62.74	50.18	47.48	47.02	70.5667	48.2266667	COG4177	E:Amino acid transport and metabolism	GO:0016021;GO:0005886;GO:0022857	CC:integral component of membrane;CC:plasma membrane;MF:transmembrane transporter activity;
BAB_RS24860	-	phosphoenolpyruvate--protein phosphotransferase	0.847	-0.23953635	0.001242035	0.00959293	no	down	mRNA	239.12	243.31	251.89	187.27	202.48	196.1	244.773	195.283333	COG3605	T:Signal transduction mechanisms	GO:0009401;GO:0016310;GO:0009401;GO:0016310;GO:0008965;GO:00	BP:phosphoenolpyruvate-dependent sugar phosphotransferase system;BP:phosphorylation;BP:phosphoenolpyruvate-dependent sugar phosphotransferase system;BP:phosphorylation;MF:phosphoenolpyruvate-protein phosphotransferase activity;MF:transferase activity, transferring phosphorus-containing groups;MF:transferase activity, transferring phosphorus-containing groups;MF:phosphoenolpyruvate-protein phosphotransferase activity
BAB_RS20385	-	hypothetical protein	0.811	-0.302491462	0.00123978	0.009598589	no	down	mRNA	30.18	31.49	37.38	25.39	25.63	24.52	33.0167	25.18	------	------	------	------
BAB_RS30935	-	aldehyde dehydrogenase	0.865	-0.20889306	0.001252556	0.009627902	no	down	mRNA	225.22	199.58	226.63	175.7	177.89	176.88	217.143	176.823333	COG1012	J:Translation, ribosomal structure and biogenesis	GO:0016620;GO:0004029;GO:0016620	MF:oxidoreductase activity, acting on the aldehyde or oxo group of donors, NAD or NADP as acceptor;MF:aldehyde dehydrogenas;MF:oxidoreductase activity, acting on the aldehyde or oxo group of donors, NAD or NADP as acceptor;
BAB_RS20100	-	acyl carrier protein	0.716	-0.48187198	0.001264919	0.009676633	no	down	mRNA	603.47	482.21	415.74	390.11	329.4	312.6	500.473	344.036667	COG0236	C:Energy production and conversion	GO:0006633;GO:0009245;GO:0036104;GO:0005737	BP:fatty acid biosynthetic process;BP:lipid A biosynthetic process;BP:Kdo2-lipid A biosynthetic process;CC:cytoplasm;
BAB_RS32795	-	ABC transporter substrate-binding protein	0.803	-0.31572113	0.001301316	0.009791873	no	down	mRNA	439.69	368.53	377.51	284.45	313.81	299.27	395.243	299.176667	COG0747	E:Amino acid transport and metabolism	GO:0055085;GO:0055085;GO:0043190;GO:0043190	BP:transmembrane transport;BP:transmembrane transport;CC:ATP-binding cassett;CC:ATP-binding cassett;
BAB_RS30925	-	ABC transporter substrate-binding protein	0.844	-0.244094099	0.001299439	0.009800702	no	down	mRNA	475.6	417.81	441.37	349.22	363.74	348.89	444.927	353.95	COG0747	J:Translation, ribosomal structure and biogenesis	GO:0055085;GO:0055085;GO:0043190;GO:0043190	BP:transmembrane transport;BP:transmembrane transport;CC:ATP-binding cassett;CC:ATP-binding cassett;
BAB_RS17770	-	cbb3-type cytochrome oxidase assembly protein CcoS	0.801	-0.32015895	0.001322831	0.009861381	no	down	mRNA	51.37	51.05	55.77	42.6	38.86	38.37	52.73	39.9433333	ENOG410XUJA;ENOG410ZXNY	P:Inorganic ion transport and metabolism;	GO:0016021	CC:integral component of membrane;
BAB_RS16535	-	regulator	0.735	-0.44396828	0.001320735	0.009868657	no	down	mRNA	471.64	544.29	537.11	351.05	381.17	395.52	517.68	375.913333	COG1937;COG1643	S:Function unknown;L:Replication, recombination	GO:0006355;GO:0006355;GO:0046872;GO:0003677;GO:0003677;GO:00	BP:regulation of transcription, DNA-templated;BP:regulation of transcription, DNA-templated;MF:metal ion binding;MF:DNA binding;MF:DNA binding;MF:metal ion binding;
BAB_RS22435	-	ABC transporter permease	0.579	-0.787689261	0.001319772	0.009884443	no	down	mRNA	10.05	8.21	11.24	4.39	6.16	5.51	9.83333	5.35333333	COG1172	S:Function unknown	------	------
BAB_RS26510	-	NAD(P)-dependent oxidoreductase	0.608	-0.717123138	0.001329171	0.009885713	no	down	mRNA	15.64	14.55	14.65	7.76	10.53	7.23	14.9467	8.50666667	ENOG4111H6U	I:Lipid transport and metabolism	GO:0000166;GO:0004316;GO:0102132;GO:0102131;GO:0016491;GO:00	MF:nucleotide binding;MF:3-oxoacyl-[acyl-carrier-protein] reductas;MF:3-oxo-pimeloyl-[acp] methyl ester reductase activity;MF:old GO;MF:oxidoreductase activity;MF:oxidoreductase activity;
BAB_RS22045	-	DUF3126 domain-containing protein	0.702	-0.511158543	0.001363915	0.010097374	no	down	mRNA	1099.61	1182.01	972.79	848.36	732.76	710.37	1084.8	763.83	ENOG411263I	S:Function unknown	------	------
BAB_RS16870	-	alcohol dehydrogenase AdhP	0.843	-0.246237314	0.001382168	0.010162256	no	down	mRNA	2185.01	1901.06	2055.48	1610.85	1617.94	1656.79	2047.18	1628.52667	COG1064	C:Energy production and conversion	GO:0016491;GO:0008270;GO:0000166;GO:0008270;GO:0004022;GO:00	MF:oxidoreductase activity;MF:zinc ion binding;MF:nucleotide binding;MF:zinc ion binding;MF:alcohol dehydrogenas;MF:oxidoreductase activity;
BAB_RS27895	-	efflux RND transporter periplasmic adaptor subunit	0.835	-0.26007256	0.001381505	0.010180675	no	down	mRNA	71.06	69.62	70.77	55.24	55.46	55.97	70.4833	55.5566667	ENOG410Z0T4	M:Cell wall/membrane/envelope biogenesis	GO:0055085;GO:0055085;GO:0016020;GO:0016020;GO:0005215;GO:00	BP:transmembrane transport;BP:transmembrane transport;CC:membrane;CC:membrane;MF:transporter activity;MF:transporter activity;
BAB_RS28120	-	DeoR/GlpR transcriptional regulator	0.672	-0.573818193	0.001391186	0.010205206	no	down	mRNA	22.37	19.32	19.65	13.53	14.13	10.96	20.4467	12.8733333	COG1349	G:Carbohydrate transport and metabolism	GO:0006351;GO:0005622;GO:0003700;GO:0003677	BP:transcription, DNA-templated;CC:intracellular;MF:transcription factor activity, sequence-specific DNA binding;MF:DNA binding;
BAB_RS20550	-	hypothetical protein	0.609	-0.715992346	0.001406815	0.010249654	no	down	mRNA	41.82	42.79	45.75	21.18	32.02	22.41	43.4533	25.2033333	COG2067	I:Lipid transport and metabolism	------	------
BAB_RS22450	-	gfo/Idh/MocA family oxidoreductase	0.804	-0.315088831	0.001410628	0.010254181	no	down	mRNA	42.32	37.75	43.27	28.19	33.08	31.95	41.1133	31.0733333	COG0673	G:Carbohydrate transport and metabolism	GO:0050112;GO:0016491;GO:0016491	MF:inositol 2-dehydrogenase activity;MF:oxidoreductase activity;MF:oxidoreductase activity;
BAB_RS17585	-	DNA-binding response regulator	0.854	-0.228198464	0.001404659	0.010257204	no	down	mRNA	61.55	59.44	65.35	51.17	50.25	48.22	62.1133	49.88	COG2197	S:Function unknown	GO:0000160;GO:0006355;GO:0006351;GO:0000160;GO:0006351;GO:00	BP:phosphorelay signal transduction system;BP:regulation of transcription, DNA-templated;BP:transcription, DNA-templated;BP:phosphorelay signal transduction system;BP:transcription, DNA-templated;BP:regulation of transcription, DNA-templated;CC:intracellular;CC:intracellular;MF:DNA binding;MF:DNA binding;
BAB_RS17065	-	gfo/Idh/MocA family oxidoreductase	0.846	-0.241510345	0.001419448	0.010295003	no	down	mRNA	131.83	114.96	131.66	99.98	98.41	103.17	126.15	100.52	COG0673	S:Function unknown	GO:0016491	MF:oxidoreductase activity;
BAB_RS30650	-	ABC transporter substrate-binding protein	0.771	-0.37504225	0.001445178	0.010364634	no	down	mRNA	50.51	39.61	43.14	30.88	33.35	32.18	44.42	32.1366667	COG0715	O:Posttranslational modification, protein turnover,	------	------
BAB_RS19570	-	hypothetical protein	0.581	-0.7844753	0.001456278	0.010397826	no	down	mRNA	200.49	219.37	219.21	92.36	147.89	134.55	213.023	124.933333	COG0834	I:Lipid transport and metabolism	------	------
BAB_RS23775	-	D-threitol dehydrogenase	0.815	-0.295987699	0.001496824	0.01059316	no	down	mRNA	135.74	120.46	129.27	90.44	105.73	100.17	128.49	98.78	COG1028	G:Carbohydrate transport and metabolism	GO:0016491	MF:oxidoreductase activity;
BAB_RS32395	-	transcriptional regulator	0.781	-0.356994993	0.001496354	0.010636692	no	down	mRNA	63.67	56.92	59.31	47.89	43.15	41.63	59.9667	44.2233333	------	------	GO:0043565	MF:sequence-specific DNA binding;
BAB_RS22405	-	DedA family protein	0.761	-0.394000322	0.001580326	0.011086432	no	down	mRNA	201.94	169.19	167.56	143.04	125.47	117.72	179.563	128.743333	COG0586	P:Inorganic ion transport and metabolism	GO:0016021	CC:integral component of membrane;
BAB_RS23675	-	ABC transporter ATP-binding protein	0.808	-0.308287668	0.001578837	0.011100224	no	down	mRNA	62.53	52.19	57.24	41.54	46.23	42.7	57.32	43.49	COG3842	P:Inorganic ion transport and metabolism	GO:0043190;GO:0043190;GO:0015417;GO:0005524;GO:0005524;GO:00	CC:ATP-binding cassett;CC:ATP-binding cassett;MF:polyamine-transporting ATPase activity;MF:ATP binding;MF:ATP binding;MF:polyamine-transporting ATPase activity;
BAB_RS19600	-	FAD-binding protein	0.853	-0.229005756	0.001577579	0.011115708	no	down	mRNA	170.71	172.23	181.65	146.81	136.61	138.2	174.863	140.54	COG0654;COG0654	C:Energy production and conversion;H:Coenzyme	GO:0071949;GO:0004497	MF:FAD binding;MF:monooxygenase activity;
BAB_RS16585	-	universal stress protein A	0.831	-0.267772247	0.001598131	0.011186917	no	down	mRNA	125.09	117.5	120.22	97.25	95.56	92.87	120.937	95.2266667	COG0589	T:Signal transduction mechanisms	------	------
BAB_RS30785	-	hypothetical protein	0.824	-0.279842594	0.001646998	0.011454125	no	down	mRNA	194.42	194.28	206.69	140.64	167.49	154.72	198.463	154.283333	ENOG410Y07C	Q:Secondary metabolites biosynthesis, transport a	GO:0016021	CC:integral component of membrane;
BAB_RS23515	-	choline ABC transporter permease subunit	0.796	-0.328622587	0.001662049	0.011508972	no	down	mRNA	51.65	51.12	49.72	38.14	36.1	40.63	50.83	38.29	COG4176	E:Amino acid transport and metabolism	GO:0055085;GO:0016021;GO:0005886	BP:transmembrane transport;CC:integral component of membrane;CC:plasma membrane;
BAB_RS22310	-	3-hydroxyisobutyrate dehydrogenase	0.823	-0.280211387	0.001707954	0.011725764	no	down	mRNA	124.48	117.7	126.96	85.63	100.57	100.19	123.047	95.4633333	COG2084	H:Coenzyme transport and metabolism	GO:0006574;GO:0006574;GO:0008442;GO:0051287;GO:0004616;GO:00	BP:valine catabolic process;BP:valine catabolic process;MF:3-hydroxyisobutyrate dehydrogenase activity;MF:NAD binding;MF:phosphogluconate dehydrogenas;MF:NAD binding;MF:3-hydroxyisobutyrate dehydrogenase activity;MF:phosphogluconate dehydrogenas;
BAB_RS30750	-	hypothetical protein	0.764	-0.387499405	0.001714748	0.011747302	no	down	mRNA	25.14	23.78	30.61	20.09	18.03	19.33	26.51	19.15	------	------	------	------
BAB_RS18935	-	DNA-binding response regulator	0.831	-0.266625869	0.001775928	0.012089102	no	down	mRNA	115.26	108.97	118.29	86.57	88.14	95.28	114.173	89.9966667	ENOG4111FPD	T:Signal transduction mechanisms	GO:0000160;GO:0006355;GO:0006351;GO:0000160;GO:0008643;GO:00	BP:phosphorelay signal transduction system;BP:regulation of transcription, DNA-templated;BP:transcription, DNA-templated;BP:phosphorelay signal transduction system;BP:carbohydrate transport;BP:regulation of transcription, DNA-templated;BP:transcription, DNA-templated;BP:phosphorelay signal transduction system;BP:transcription, DNA-templated;BP:regulation of transcription, DNA-templated;CC:intracellular;CC:intracellular;MF:DNA binding;MF:DNA binding;
BAB_RS29185	-	pca operon transcription factor PcaQ	0.834	-0.262164707	0.001828139	0.012365917	no	down	mRNA	84.82	77.73	79.8	65.34	62.29	62.98	80.7833	63.5366667	ENOG410YQ2R	K:Transcription	GO:0019619;GO:0045893;GO:0006351;GO:0019619;GO:0006351;GO:00	BP:3,4-dihydroxybenzoate catabolic process;BP:positive regulation of transcription, DNA-templated;BP:transcription, DNA-templated;BP:3,4-dihydroxybenzoate catabolic process;BP:transcription, DNA-templated;BP:positive regulation of transcription, DNA-templated;MF:DNA binding;MF:transcription factor activity, sequence-specific DNA binding;MF:transcription factor activity, sequence-specific DNA binding;MF:DNA binding;
BAB_RS16365	-	cytochrome c biogenesis ATP-binding export protein CcmA	0.825	-0.27681911	0.001854668	0.012388873	no	down	mRNA	93.07	88.76	88.65	67.14	72.55	71.47	90.16	70.3866667	COG4133	O:Posttranslational modification, protein turnover,	GO:0017004;GO:0017004;GO:0005886;GO:0005886;GO:0103115;GO:00	BP:cytochrome complex assembly;BP:cytochrome complex assembly;CC:plasma membrane;CC:plasma membrane;MF:protoheme IX ABC transporter activity;MF:heme-transporting ATPase activity;MF:ATP binding;MF:ATP binding;MF:heme-transporting ATPase activity;MF:protoheme IX ABC transporter activity;
BAB_RS30260	livF	ABC transporter ATP-binding protein	0.642	-0.638657518	0.001877497	0.012463634	no	down	mRNA	13.5	14.31	12.99	7.21	8.9	8.68	13.6	8.26333333	COG0410	I:Lipid transport and metabolism	GO:0016887;GO:0005524;GO:0005524;GO:0016887	MF:ATPase activity;MF:ATP binding;MF:ATP binding;MF:ATPase activity;
BAB_RS28310	-	DNA ligase-associated DEXH box helicase	0.827	-0.274395333	0.001985473	0.01301903	no	down	mRNA	16.15	16.12	18.39	13.03	12.88	13.55	16.8867	13.1533333	COG1201	L:Replication, recombination and repair	GO:0005840;GO:0003676;GO:0005524;GO:0004386;GO:0016818;GO:00	CC:ribosome;MF:nucleic acid binding;MF:ATP binding;MF:helicase activity;MF:hydrolase activity, acting on acid anhydrides, in phosphorus-containing anhydrides;MF:nucleic acid binding;MF:ATP binding;MF:helicase activity;
BAB_RS19685	-	bifunctional diaminohydroxyphosphoribosylaminopyrimidine deaminase/5-amino-6-(5-phosphoribosylamino)uracil reductase RibD	0.853	-0.229051595	0.002016536	0.013195787	no	down	mRNA	144.39	146.53	161.06	118.55	124.2	121.35	150.66	121.366667	COG0117	H:Coenzyme transport and metabolism	GO:0009231;GO:0008703;GO:0008835;GO:0008270	BP:riboflavin biosynthetic process;MF:5-amino-6;MF:diaminohydroxyphosphoribosylaminopyrimidine deaminase activity;MF:zinc ion binding;
BAB_RS28610	-	diversity-generating retroelement protein bAvd family protein	0.807	-0.308890471	0.002072779	0.01353626	no	down	mRNA	258.16	240.33	213.57	178.47	192.75	179.95	237.353	183.723333	ENOG41121RZ	S:Function unknown	GO:0005840;GO:0016491	CC:ribosome;MF:oxidoreductase activity;
BAB_RS24575	-	acetyl-coenzyme A synthetase	0.812	-0.300365558	0.002195772	0.014195201	no	down	mRNA	139.7	124.58	134.01	89.18	105.87	109.31	132.763	101.453333	COG0365	E:Amino acid transport and metabolism	GO:0019427;GO:0019427;GO:0019427;GO:0003987;GO:0016208;GO:00	BP:acetyl-CoA biosynthetic process from acetate;BP:acetyl-CoA biosynthetic process from acetate;BP:acetyl-CoA biosynthetic process from acetate;MF:acetate-CoA ligase activity;MF:AMP binding;MF:ATP binding;MF:metal ion binding;MF:acetate-CoA ligase activity;MF:AMP binding;MF:ATP binding;MF:metal ion binding;MF:acetate-CoA ligase activity;MF:AMP binding;MF:ATP binding;MF:metal ion binding;MF:oxidoreductase activity;
BAB_RS29980	-	MarR family transcriptional regulator	0.784	-0.351862227	0.00221119	0.014209108	no	down	mRNA	167.72	153.02	143.21	114.2	125.8	105.06	154.65	115.02	COG1846;ENOG41120VD	P:Inorganic ion transport and metabolism;K:Trans	GO:0006351;GO:0006351;GO:0003700;GO:0003677;GO:0003677;GO:00	BP:transcription, DNA-templated;BP:transcription, DNA-templated;MF:transcription factor activity, sequence-specific DNA binding;MF:DNA binding;MF:DNA binding;MF:transcription factor activity, sequence-specific DNA binding;
BAB_RS17660	-	superoxide dismutase	0.703	-0.5084694	0.002383566	0.015195235	no	down	mRNA	842.41	958.34	869.43	627.66	534.88	719.42	890.06	627.32	COG0605	P:Inorganic ion transport and metabolism	GO:0004784;GO:0046872;GO:0046872;GO:0004784	MF:superoxide dismutase activity;MF:metal ion binding;MF:metal ion binding;MF:superoxide dismutase activity;
BAB_RS19740	-	MerR family transcriptional regulator	0.873	-0.195808304	0.002407594	0.015257593	no	down	mRNA	276.16	259.58	293.44	217.75	235.37	229.21	276.393	227.443333	COG0789	I:Lipid transport and metabolism	GO:0006355;GO:0006355;GO:0003677;GO:0003677	BP:regulation of transcription, DNA-templated;BP:regulation of transcription, DNA-templated;MF:DNA binding;MF:DNA binding;
BAB_RS21305	-	ribose 1%2C5-bisphosphate phosphokinase PhnN	0.828	-0.272607029	0.002413705	0.015266212	no	down	mRNA	60.69	59.33	62.68	47.85	50.56	44.15	60.9	47.52	COG3709	P:Inorganic ion transport and metabolism	GO:0006015;GO:0019634;GO:0019634;GO:0006015;GO:0019634;GO:00	BP:5-phosphoribose 1-diphosphate biosynthetic process;BP:organic phosphonate metabolic process;BP:organic phosphonate metabolic process;BP:5-phosphoribose 1-diphosphate biosynthetic process;BP:organic phosphonate metabolic process;BP:5-phosphoribose 1-diphosphate biosynthetic process;MF:ATP binding;MF:ribose 1,5-bisphosphate phosphokinase activity;MF:ribose 1,5-bisphosphate phosphokinase activity;MF:ATP binding;MF:ribose 1,5-bisphosphate phosphokinase activity;MF:ATP binding;
BAB_RS21020	-	NUDIX domain-containing protein	0.873	-0.196258276	0.002420966	0.015282053	no	down	mRNA	197.35	182.55	205.35	160.4	156.84	164.32	195.083	160.52	COG0494	F:Nucleotide transport and metabolism	GO:0016787;GO:0004081	MF:hydrolase activity;MF:bi;
BAB_RS23920	-	response regulator	0.819	-0.288309805	0.002457121	0.015419394	no	down	mRNA	2997.79	2582.72	2584	2013.65	2103.31	2199.26	2721.5	2105.40667	COG0784;ENOG410YE83	T:Signal transduction mechanisms;	GO:0000160;GO:0000160;GO:0005622;GO:0005622;GO:0046872	BP:phosphorelay signal transduction system;BP:phosphorelay signal transduction system;CC:intracellular;CC:intracellular;MF:metal ion binding;
BAB_RS31845	-	branched-chain amino acid ABC transporter permease	0.719	-0.475473389	0.002516736	0.015732047	no	down	mRNA	37.99	33.82	38.44	24.22	26.76	24.58	36.75	25.1866667	COG0559	E:Amino acid transport and metabolism	GO:0016021;GO:0005886;GO:0022857	CC:integral component of membrane;CC:plasma membrane;MF:transmembrane transporter activity;
BAB_RS22205	-	flavin reductase	0.763	-0.391044254	0.002551188	0.015824258	no	down	mRNA	108.95	88.61	106.63	77.48	71.07	73.7	101.397	74.0833333	COG1853	S:Function unknown	GO:0010181;GO:0004497	MF:FMN binding;MF:monooxygenase activity;
BAB_RS17200	-	transcriptional regulator	0.806	-0.312010247	0.002543395	0.015837069	no	down	mRNA	178.61	150.95	148.75	125.95	118.42	122.67	159.437	122.346667	COG2856;ENOG410Y0U9	E:Amino acid transport and metabolism;S:Functior	GO:0043565;GO:0003677	MF:sequence-specific DNA binding;MF:DNA binding;
BAB_RS26405	-	ABC transporter ATP-binding protein	0.796	-0.328489621	0.00256876	0.015871975	no	down	mRNA	35.22	36.25	33.71	24.77	26.08	28.23	35.06	26.36	COG3845	S:Function unknown	GO:0008643;GO:0016887;GO:0005524;GO:0005524;GO:0016887	BP:carbohydrate transport;MF:ATPase activity;MF:ATP binding;MF:ATP binding;MF:ATPase activity;
BAB_RS18465	-	polysaccharide biosynthesis protein	0.858	-0.221452665	0.002566355	0.015887666	no	down	mRNA	55.11	49.72	52.94	42.28	41.88	43.09	52.59	42.4166667	COG1086	M:Cell wall/membrane/envelope biogenesis	GO:0016021	CC:integral component of membrane;
BAB_RS20850	-	ribose-5-phosphate isomerase	0.851	-0.232807394	0.002581202	0.015887743	no	down	mRNA	171.74	152.49	159.44	132.24	126.99	129.12	161.223	129.45	COG0120	G:Carbohydrate transport and metabolism	GO:0009052;GO:0009052;GO:0009052;GO:0009052;GO:0016787;GO:00	BP:pentose-phosphate shunt, non-oxidative branch;BP:pentose-phosphate shunt, non-oxidative branch;BP:pentose-phosphate shunt, non-oxidative branch;BP:pentose-phosphate shunt, non-oxidative branch;MF:hydrolase activity;MF:ribose-5-phosphate isomerase activity;MF:ribose-5-phosphate isomerase activity;MF:hydrolase activity;MF:ribose-5-phosphate isomerase activity;MF:ribose-5-phosphate isomerase activity;
BAB_RS22795	-	DNA repair protein RecN	0.831	-0.266832031	0.002577961	0.015898252	no	down	mRNA	77.34	78.64	86.97	58.09	67.44	64.74	80.9833	63.4233333	COG0497	L:Replication, recombination and repair	GO:0006310;GO:0006281;GO:0006310;GO:0006281;GO:0005524;GO:00	BP:DNA recombination;BP:DNA repair;BP:DNA recombination;BP:DNA repair;MF:ATP binding;MF:ATP binding;
BAB_RS25920	-	HPr family phosphocarrier protein	0.772	-0.374134535	0.002636798	0.016106522	no	down	mRNA	190.4	151.07	174	133.53	120.39	129.47	171.823	127.796667	COG1925	G:Carbohydrate transport and metabolism	GO:0009401;GO:0009401;GO:0009401;GO:0005737;GO:0005737;GO:00	BP:phosphoenolpyruvate-dependent sugar phosphotransferase system;BP:phosphoenolpyruvate-dependent sugar phosphotransferase system;BP:phosphoenolpyruvate-dependent sugar phosphotransferase system;CC:cytoplasm;CC:cytoplasm;CC:cytoplasm;MF:transferase activity;MF:transferase activity;MF:kinase activity;
BAB_RS25475	-	exodeoxyribonuclease III	0.841	-0.249337347	0.002648023	0.016144396	no	down	mRNA	69.89	61.89	70.38	52.96	54.57	52.9	67.3867	53.4766667	COG0708	M:Cell wall/membrane/envelope biogenesis	GO:0006281;GO:0006281;GO:0006281;GO:0005622;GO:0005622;GO:00	BP:DNA repair;BP:DNA repair;BP:DNA repair;CC:intracellular;CC:intracellular;MF:DNA binding;MF:endonuclease activity;MF:exodeoxyribonuclease III activity;MF:endonuclease activity;MF:exodeoxyribonuclease III activity;MF:DNA binding;MF:endonuclease activity;MF:exodeoxyribonuclease III activity;MF:DNA binding;
BAB_RS16430	-	sulfate ABC transporter permease subunit CysT	0.781	-0.35681936	0.002655455	0.016159045	no	down	mRNA	41.32	34.91	40.28	25.72	29.98	30.01	38.8367	28.57	COG0555	P:Inorganic ion transport and metabolism	GO:0016021;GO:0015419	CC:integral component of membrane;MF:ATPase-coupled sulfate transmembrane transporter activity;
BAB_RS33480	-	hypothetical protein	0.832	-0.266192535	0.00267649	0.016256264	no	down	mRNA	196.11	190.39	214.46	150.91	169.59	154.72	200.32	158.406667	COG0154	J:Translation, ribosomal structure and biogenesis	------	------
BAB_RS21895	-	50S ribosomal protein L22	0.861	-0.215477141	0.002694305	0.016333588	no	down	mRNA	1217.31	1030.12	1083.21	885.72	946.6	924.52	1110.21	918.946667	COG0091	J:Translation, ribosomal structure and biogenesis	GO:0006412;GO:0015934;GO:0003735;GO:0019843	BP:translation;CC:large ribosomal subunit;MF:structural constituent of ribosome;MF:rRNA binding;
BAB_RS20645	-	site-specific integrase	0.787	-0.346325669	0.002746117	0.016522983	no	down	mRNA	81.28	79.17	69.3	59.2	54.46	57.05	76.5833	56.9033333	COG0582	C:Energy production and conversion	GO:0006310;GO:0015074;GO:0015074;GO:0006310;GO:0003677;GO:00	BP:DNA recombination;BP:DNA integration;BP:DNA integration;BP:DNA recombination;MF:DNA binding;MF:DNA binding;
BAB_RS16770	-	nitrite/sulfite reductase	0.726	-0.461398886	0.002766364	0.016613697	no	down	mRNA	14.48	12.56	14.63	9.03	8.28	11.24	13.89	9.51666667	COG0155	C:Energy production and conversion	GO:0046872;GO:0004783;GO:0051539;GO:0020037;GO:0016491;GO:00	MF:metal ion binding;MF:sulfite reductas;MF:4 iron, 4 sulfur cluster binding;MF:heme binding;MF:oxidoreductase activity;MF:4 iron, 4 sulfur cluster binding;MF:heme binding;MF:metal ion binding;MF:oxidoreductase activity;
BAB_RS28705	-	bicyclomycin resistance protein	0.832	-0.265592417	0.002812295	0.016826639	no	down	mRNA	60.5	57.17	56.89	44.23	44.81	47.93	58.1867	45.6566667	ENOG4111KWE	G:Carbohydrate transport and metabolism	GO:0055085;GO:0055085;GO:0042597	BP:transmembrane transport;BP:transmembrane transport;CC:periplasmic space;
BAB_RS20290	-	phytoene/squalene synthase family protein	0.831	-0.267183892	0.002836159	0.016937881	no	down	mRNA	55.62	48.73	52.73	40.04	41.55	41.42	52.36	41.0033333	COG1562	I:Lipid transport and metabolism	GO:0009058;GO:0016740	BP:biosynthetic process;MF:transferase activity;
BAB_RS16760	-	uroporphyrinogen-III C-methyltransferase	0.614	-0.704572648	0.002884428	0.01719419	no	down	mRNA	18.27	20.29	23.17	9.9	9.73	16.49	20.5767	12.04	COG1648	H:Coenzyme transport and metabolism	GO:0009236;GO:0019354;GO:0009236;GO:0019354;GO:0045552;GO:00	BP:cobalamin biosynthetic process;BP:siroheme biosynthetic process;BP:cobalamin biosynthetic process;BP:siroheme biosynthetic process;MF:dihydrokaempferol 4-reductase activity;MF:NAD binding;MF:precorrin-2 dehydrogenase activity;MF:sirohydrochlorin ferrochelatase activity;MF:uroporphyrin-III C-methyltransferase activity;MF:NAD binding;MF:precorrin-2 dehydrogenase activity;MF:sirohydrochlorin ferrochelatase activity;MF:uroporphyrin-III C-methyltransferase activity;
BAB_RS29330	-	50S ribosomal protein L33	0.695	-0.524486852	0.002900865	0.017260148	no	down	mRNA	343.04	290.63	362.73	273.56	205.19	177.98	332.133	218.91	COG0267	J:Translation, ribosomal structure and biogenesis	GO:0006412;GO:0005840;GO:0003735	BP:translation;CC:ribosome;MF:structural constituent of ribosome;
BAB_RS30955	-	DUF4105 domain-containing protein	0.821	-0.28534671	0.002912778	0.017267075	no	down	mRNA	62.89	54.53	57.76	43.24	48.78	43.09	58.3933	45.0366667	ENOG410XT9F	S:Function unknown	GO:0016021	CC:integral component of membrane;
BAB_RS28995	-	Ppx/GppA family phosphatase	0.84	-0.251635557	0.002987204	0.017643174	no	down	mRNA	70.92	68.53	78.61	61.92	55.57	54.96	72.6867	57.4833333	COG0248	J:Translation, ribosomal structure and biogenesis	------	------
BAB_RS27170	-	DUF465 domain-containing protein	0.865	-0.209682463	0.003087289	0.018101201	no	down	mRNA	2799.81	2508.13	2788.93	2145.12	2180.43	2285.47	2698.96	2203.67333	COG2841	S:Function unknown	------	------
BAB_RS16225	-	hypothetical protein	0.751	-0.413651949	0.003102726	0.018158575	no	down	mRNA	1447	1016.76	957.25	844.86	839.38	854.39	1140.34	846.21	ENOG410XYJX	S:Function unknown	------	------
BAB_RS22420	-	RbsD/FucU transporter	0.871	-0.199153689	0.003116691	0.018207145	no	down	mRNA	219.59	204.41	235.04	172.31	186.74	182.17	219.68	180.406667	COG4154	G:Carbohydrate transport and metabolism	GO:0005996;GO:0005996;GO:0016853;GO:0048029;GO:0016853;GO:00	BP:monosaccharide metabolic process;BP:monosaccharide metabolic process;MF:isomerase activity;MF:monosaccharide binding;MF:isomerase activity;MF:monosaccharide binding;
BAB_RS22475	-	ABC transporter permease	0.687	-0.541597404	0.003155975	0.018369833	no	down	mRNA	8.16	7.89	9.5	6.08	5.6	4.89	8.51667	5.52333333	COG0842	V:Defense mechanisms	GO:0055085;GO:0043190	BP:transmembrane transport;CC:ATP-binding cassett;
BAB_RS20775	-	thymidylate kinase	0.783	-0.353167259	0.003190585	0.018470902	no	down	mRNA	38.69	34.15	37.78	25.3	27.72	28.86	36.8733	27.2933333	COG0125	F:Nucleotide transport and metabolism	GO:0006235;GO:0006233;GO:0016787;GO:0004798;GO:0005524	BP:dTTP biosynthetic process;BP:dTDP biosynthetic process;MF:hydrolase activity;MF:thymidylate kinase activity;MF:ATP binding;
BAB_RS30275	-	branched-chain amino acid ABC transporter substrate-binding protein	0.54	-0.88990403	0.003230011	0.01859861	no	down	mRNA	39.55	35.84	36.78	13.46	14.63	29.4	37.39	19.1633333	COG0683	E:Amino acid transport and metabolism	GO:0006865	BP:amino acid transport;
BAB_RS28600	-	MarR family transcriptional regulator	0.863	-0.212266586	0.003279148	0.018780576	no	down	mRNA	86.65	79.68	91.23	70.36	69.76	69.52	85.8533	69.88	ENOG4111UVT	K:Transcription	GO:0006351;GO:0003700;GO:0003677	BP:transcription, DNA-templated;MF:transcription factor activity, sequence-specific DNA binding;MF:DNA binding;
BAB_RS28850	-	3D-(3%2C5/4)-trihydroxycyclohexane-1%2C2-dione acylhydrolase (decyclizing)	0.839	-0.254073023	0.003274182	0.01878562	no	down	mRNA	33.88	34.27	37.48	26.12	28.27	29.1	35.21	27.83	COG3962	E:Amino acid transport and metabolism	GO:0019310;GO:0019310;GO:0016823;GO:0000287;GO:0030976;GO:00	BP:inositol catabolic process;BP:inositol catabolic process;MF:hydrolase activity, acting on acid carbon-carbon bonds, in ketonic substances;MF:magnesium ion binding;MF:thiamine pyrophosphate binding;MF:hydrolase activity, acting on acid carbon-carbon bonds, in ketonic substances;MF:magnesium ion binding;MF:thiamine pyrophosphate binding;
BAB_RS32155	-	hypothetical protein	0.829	-0.270121198	0.003311314	0.018830532	no	down	mRNA	44.74	41.04	43.48	34.07	32.34	34.75	43.0867	33.72	------	------	------	------
BAB_RS26310	-	D-glycerate dehydrogenase	0.872	-0.196991894	0.003327243	0.018887688	no	down	mRNA	95.05	89.74	94.78	74.65	76.08	78.87	93.19	76.5333333	COG1052	C:Energy production and conversion	GO:0008873;GO:0051287;GO:0016616;GO:0008873;GO:0051287;GO:00	MF:gluconate 2-dehydrogenase activity;MF:NAD binding;MF:oxidoreductase activity, acting on the CH-OH group of donors, NAD or NADP as acceptor;MF:gluconate 2-dehydrogenase activity;MF:NAD binding;MF:NAD binding;MF:oxidoreductase activity, acting on the CH-OH group of donors, NAD or NADP as acceptor;
BAB_RS28560	-	6-phosphogluconolactonase	0.836	-0.258982559	0.003339129	0.018921728	no	down	mRNA	162.14	153.96	158.68	113.51	128.51	133.32	158.26	125.113333	COG0363	G:Carbohydrate transport and metabolism	GO:0005975;GO:0006098;GO:0006098;GO:0005975;GO:0017057;GO:00	BP:carbohydrate metabolic process;BP:pentose-phosphate shunt;BP:pentose-phosphate shunt;BP:carbohydrate metabolic process;MF:6-phosphogluconolactonase activity;MF:6-phosphogluconolactonase activity;MF:isomerase activity;
BAB_RS22195	-	phospholipase	0.702	-0.510482396	0.003372254	0.019042269	no	down	mRNA	9.7	10.26	10.19	6.5	7.05	6.44	10.05	6.66333333	ENOG41111DH	S:Function unknown	------	------
BAB_RS29545	-	BMP family ABC transporter substrate-binding protein	0.793	-0.334394516	0.003368552	0.019054854	no	down	mRNA	57.41	51.41	49.39	40.08	36.21	42.05	52.7367	39.4466667	COG1744	C:Energy production and conversion	GO:0005886	CC:plasma membrane;
BAB_RS18070	-	hypothetical protein	0.852	-0.230837938	0.003401948	0.019109194	no	down	mRNA	1604.05	1360.11	1415.12	1188.09	1150.79	1191.62	1459.76	1176.83333	------	------	------	------
BAB_RS29100	-	SLC13 family permease	0.853	-0.228981462	0.003397358	0.019116832	no	down	mRNA	28.87	26.92	29.51	22.54	23.51	22.39	28.4333	22.8133333	COG0471	P:Inorganic ion transport and metabolism	GO:0006813;GO:0006814;GO:0006813;GO:0006814;GO:0016021;GO:00	BP:potassium ion transport;BP:sodium ion transport;BP:potassium ion transport;BP:sodium ion transport;CC:integral component of membrane;CC:integral component of membrane;MF:cation transmembrane transporter activity;MF:cation transmembrane transporter activity;
BAB_RS19805	-	PaaI family thioesterase	0.68	-0.556618491	0.003423568	0.019197075	no	down	mRNA	27.51	19.32	23.83	15.86	15.58	13.62	23.5533	15.02	COG2050	Q:Secondary metabolites biosynthesis, transport a	------	------
BAB_RS25570	-	J domain-containing protein	0.87	-0.200521384	0.003543662	0.019766988	no	down	mRNA	564.86	497.98	524.37	419.06	442.98	443.88	529.07	435.306667	COG2214	H:Coenzyme transport and metabolism	------	------
BAB_RS21830	-	50S ribosomal protein L30	0.806	-0.31026949	0.003538215	0.01977093	no	down	mRNA	1081.27	908.82	1073.71	809.81	828.45	770.38	1021.27	802.88	COG1841	J:Translation, ribosomal structure and biogenesis	GO:0006412;GO:0006412;GO:0015934;GO:0015934;GO:0003735;GO:00	BP:translation;BP:translation;CC:large ribosomal subunit;CC:large ribosomal subunit;MF:structural constituent of ribosome;MF:structural constituent of ribosome;
BAB_RS31840	-	branched-chain amino acid ABC transporter permease	0.777	-0.364253357	0.003551325	0.0197754	no	down	mRNA	151.53	119.18	125.81	92.32	108.56	90.18	132.173	97.02	COG0559	E:Amino acid transport and metabolism	GO:0016021;GO:0005886;GO:0022857	CC:integral component of membrane;CC:plasma membrane;MF:transmembrane transporter activity;
BAB_RS19050	-	DUF2147 domain-containing protein	0.782	-0.355112771	0.003534921	0.019786934	no	down	mRNA	374.53	360.67	420.71	283.65	311.88	287.51	385.303	294.346667	COG4731	S:Function unknown	GO:0005524	MF:ATP binding;
BAB_RS26525	-	pyruvate dehydrogenase complex dihydrolipoamide acetyltransferase	0.663	-0.593250663	0.003564721	0.019815654	no	down	mRNA	4.47	4.36	4.54	2.5	2.8	3.06	4.45667	2.78666667	COG0508	S:Function unknown	GO:0006090;GO:0006090;GO:0006090;GO:0045254;GO:0045254;GO:00	BP:pyruvate metabolic process;BP:pyruvate metabolic process;BP:pyruvate metabolic process;CC:pyruvate dehydrogenase complex;CC:pyruvate dehydrogenase complex;CC:pyruvate dehydrogenase complex;MF:methyltransferase activity;MF:dihydrolipoyllysine-residue acetyltransferase activity;MF:dihydrolipoyllysine-residue acetyltransferase activity;MF:dihydrolipoyllysine-residue acetyltransferase activity;
BAB_RS28415	-	altronate dehydratase	0.819	-0.288792878	0.003583042	0.019883098	no	down	mRNA	20.74	18.43	19.39	15.69	14.77	14.58	19.52	15.0133333	COG2721	G:Carbohydrate transport and metabolism	GO:0016787;GO:0016829;GO:0008867;GO:0016829	MF:hydrolase activity;MF:lyase activity;MF:galactarate dehydratase activity;MF:lyase activity;
BAB_RS32690	-	hypothetical protein	0.685	-0.545044177	0.00361948	0.02005067	no	down	mRNA	1964.83	2197.35	2321.44	1424.74	1630.47	1479.03	2161.21	1511.41333	------	------	------	------
BAB_RS21550	-	UDP-3-O-(3-hydroxymyristoyl)glucosamine N-acyltransferase	0.874	-0.19409434	0.003644836	0.02005284	no	down	mRNA	171.05	167.35	177.1	141.81	139.54	144.27	171.833	141.873333	COG1044	M:Cell wall/membrane/envelope biogenesis	GO:0009245;GO:0009245;GO:0016853;GO:0016410;GO:0016410	BP:lipid A biosynthetic process;BP:lipid A biosynthetic process;MF:isomerase activity;MF:N-acyltransferase activity;MF:N-acyltransferase activity;
BAB_RS26115	-	septal ring lytic transglycosylase RlpA family protein	0.827	-0.274614363	0.003689621	0.020195492	no	down	mRNA	670.62	593.19	561.71	507.04	451.98	473.25	608.507	477.423333	COG0797	S:Function unknown	GO:0071555;GO:0000270;GO:0071555;GO:0000270;GO:0016829;GO:00	BP:cell wall organization;BP:peptidoglycan metabolic process;BP:cell wall organization;BP:peptidoglycan metabolic process;MF:lyase activity;MF:lytic endotransglycosylase activity;MF:lyase activity;MF:lytic endotransglycosylase activity;
BAB_RS22055	-	alpha/beta hydrolase	0.864	-0.210414043	0.003737493	0.020422729	no	down	mRNA	175.15	156.14	182.04	137.67	142.39	139.33	171.11	139.796667	COG0596	E:Amino acid transport and metabolism	GO:0016787;GO:0016787;GO:0004601;GO:0004177	MF:hydrolase activity;MF:hydrolase activity;MF:peroxidase activity;MF:aminopeptidase activity;
BAB_RS23800	-	choline dehydrogenase	0.814	-0.296483998	0.003763992	0.020497808	no	down	mRNA	70.3	66.4	65.85	49.52	57.77	47.92	67.5167	51.7366667	COG2303	S:Function unknown	GO:0050660;GO:0016614	MF:flavin adenine dinucleotide binding;MF:oxidoreductase activity, acting on CH-OH group of donors;
BAB_RS24175	-	50S ribosomal protein L31	0.733	-0.447722769	0.003805894	0.020690927	no	down	mRNA	1973.1	1680.48	1599.95	1508.93	1086.52	1091.52	1751.18	1228.99	COG0254	J:Translation, ribosomal structure and biogenesis	GO:0006412;GO:0006412;GO:0005840;GO:0005840;GO:0019843;GO:00	BP:translation;BP:translation;CC:ribosome;CC:ribosome;MF:rRNA binding;MF:structural constituent of ribosome;MF:structural constituent of ribosome;MF:rRNA binding;
BAB_RS19385	-	ribonuclease T(2)	0.763	-0.389756109	0.003850936	0.020865191	no	down	mRNA	33.82	29.58	35.82	23.53	21.93	26.38	33.0733	23.9466667	COG3719	J:Translation, ribosomal structure and biogenesis	GO:0033897;GO:0003723	MF:ribonuclease T2 activity;MF:RNA binding;
BAB_RS19935	-	methylmalonyl-CoA epimerase	0.847	-0.238869484	0.003907578	0.02110092	no	down	mRNA	259.47	252.9	246.55	197.42	213.09	207.73	252.973	206.08	COG0346	S:Function unknown	GO:0004493;GO:0016787;GO:0051213;GO:0004493;GO:0004462	MF:methylmalonyl-CoA epimerase activity;MF:hydrolase activity;MF:dioxygenase activity;MF:methylmalonyl-CoA epimerase activity;MF:lactoylglutathione lyase activity;
BAB_RS26325	-	MarR family transcriptional regulator	0.735	-0.444259786	0.003999348	0.021380875	no	down	mRNA	79.57	81.85	81.64	56.89	62.89	53.51	81.02	57.7633333	ENOG4111XUF	K:Transcription	GO:0006351;GO:0006351;GO:0003700;GO:0003677;GO:0003677;GO:00	BP:transcription, DNA-templated;BP:transcription, DNA-templated;MF:transcription factor activity, sequence-specific DNA binding;MF:DNA binding;MF:DNA binding;MF:transcription factor activity, sequence-specific DNA binding;
BAB_RS20580	-	molybdopterin-guanine dinucleotide biosynthesis protein B	0.847	-0.239053641	0.004040548	0.021458317	no	down	mRNA	295.25	269.13	271.89	227.12	234.82	208.2	278.757	223.38	COG1763	H:Coenzyme transport and metabolism	GO:0006777;GO:0006777;GO:0005525;GO:0005525	BP:Mo-molybdopterin cofactor biosynthetic process;BP:Mo-molybdopterin cofactor biosynthetic process;MF:GTP binding;MF:GTP binding;
BAB_RS16775	-	phosphoadenylyl-sulfate reductase	0.666	-0.587048877	0.004051696	0.021482014	no	down	mRNA	11.34	8.92	9.57	5.89	6.19	6.53	9.94333	6.20333333	COG0175	P:Inorganic ion transport and metabolism	GO:0070814;GO:0019379;GO:0070814;GO:0019379;GO:0005737;GO:00	BP:hydrogen sulfide biosynthetic process;BP:sulfate assimilation, phosphoadenylyl sulfate reduction by phosphoadenylyl-sulfate reductas;BP:hydrogen sulfide biosynthetic process;BP:sulfate assimilation, phosphoadenylyl sulfate reduction by phosphoadenylyl-sulfate reductas;CC:cytoplasm;CC:cytoplasm;MF:phosphoadenylyl-sulfate reductas;MF:hydrolase activity, acting on glycosyl bonds;MF:phosphoadenylyl-sulfate reductas;
BAB_RS18925	-	hypothetical protein	0.733	-0.447795325	0.004105943	0.02169802	no	down	mRNA	108.2	85.57	76.58	62.38	61.32	65.38	90.1167	63.0266667	ENOG410Y2MW	S:Function unknown	GO:0016021	CC:integral component of membrane;
BAB_RS16600	-	bifunctional uridylyltransferase/uridylyl-removing protein	0.852	-0.231254301	0.004178467	0.022045014	no	down	mRNA	50.97	48.38	52.03	37.26	40.9	43.18	50.46	40.4466667	COG2844	O:Posttranslational modification, protein turnover,	GO:0006808;GO:0006807;GO:0006807;GO:0016787;GO:0008773;GO:00	BP:regulation of nitrogen utilization;BP:nitrogen compound metabolic process;BP:nitrogen compound metabolic process;MF:hydrolase activity;MF:[protein-PII] uridylyltransferase activity;MF:phosphoric diester hydrolase activity;MF:[protein-PII] uridylyltransferase activity;MF:hydrolase activity;
BAB_RS29015	-	5-oxoprolinase	0.881	-0.182553898	0.004242702	0.022310643	no	down	mRNA	39.71	37.02	41.84	32.73	32.64	32.82	39.5233	32.73	COG0146;COG0146	E:Amino acid transport and metabolism;Q:Second	GO:0016787	MF:hydrolase activity;
BAB_RS28710	-	SDR family oxidoreductase	0.812	-0.300461249	0.004302817	0.022516208	no	down	mRNA	61.03	61.52	60.7	44.84	51.42	44.4	61.0833	46.8866667	ENOG4111GP3	S:Function unknown	GO:0016491	MF:oxidoreductase activity;
BAB_RS19660	-	hypothetical protein	0.742	-0.430900875	0.004362693	0.022755409	no	down	mRNA	47.44	55.81	53.8	31.88	40.79	38.45	52.35	37.04	ENOG4112797	S:Function unknown	GO:0016021	CC:integral component of membrane;
BAB_RS16665	-	RNA polymerase sigma-54 factor	0.768	-0.380463017	0.00439555	0.02288963	no	down	mRNA	19.57	16.29	16.06	11.73	13.39	12.2	17.3067	12.44	COG1508	K:Transcription	GO:0006352;GO:0006352;GO:0016987;GO:0001216;GO:0003677;GO:00	BP:DNA-templated transcription, initiation;BP:DNA-templated transcription, initiation;MF:sigma factor activity;MF:bacterial-type RNA polymerase transcriptional activator activity, sequence-specific DNA binding;MF:DNA binding;MF:DNA-directed RNA polymerase activity;MF:DNA-directed RNA polymerase activity;MF:DNA binding;MF:bacterial-type RNA polymerase transcriptional activator activity, sequence-specific DNA binding;MF:sigma factor activity;
BAB_RS20310	-	hypothetical protein	0.842	-0.248915294	0.004453149	0.023077365	no	down	mRNA	83.26	79.59	77.49	62.47	64.98	63.77	80.1133	63.74	COG4929	S:Function unknown	------	------
BAB_RS31475	-	Rrf2 family transcriptional regulator	0.865	-0.209062944	0.004516638	0.023331124	no	down	mRNA	95.23	85.15	98.11	73	78.13	75.92	92.83	75.6833333	COG1959	S:Function unknown	GO:0003677	MF:DNA binding;
BAB_RS25695	-	LacI family DNA-binding transcriptional regulator	0.667	-0.583925156	0.004556584	0.023499687	no	down	mRNA	5.4	6.17	5.96	3.5	3.78	3.8	5.84333	3.69333333	COG1609	S:Function unknown	GO:0006355;GO:0006351;GO:0006355;GO:0006351;GO:0003677;GO:00	BP:regulation of transcription, DNA-templated;BP:transcription, DNA-templated;BP:regulation of transcription, DNA-templated;BP:transcription, DNA-templated;MF:DNA binding;MF:DNA binding;
BAB_RS26475	-	FadR family transcriptional regulator	0.827	-0.274000981	0.004588129	0.023624452	no	down	mRNA	80.39	77.38	78.98	58.87	57.9	68.33	78.9167	61.7	COG2186	S:Function unknown	GO:0006351;GO:0006351;GO:0003700;GO:0003677;GO:0003677;GO:00	BP:transcription, DNA-templated;BP:transcription, DNA-templated;MF:transcription factor activity, sequence-specific DNA binding;MF:DNA binding;MF:DNA binding;MF:transcription factor activity, sequence-specific DNA binding;
BAB_RS30465	-	hypothetical protein	0.789	-0.341907017	0.004615498	0.02368945	no	down	mRNA	246.63	234.96	278.55	193.94	199.52	190.94	253.38	194.8	------	------	------	------
BAB_RS25160	-	hydroxyacylglutathione hydrolase	0.821	-0.284473552	0.004693843	0.02397666	no	down	mRNA	49.42	48.01	53.85	40.75	40.61	36.07	50.4267	39.1433333	COG0491;ENOG4111FEZ	D:Cell cycle control, cell division, chromosome par	GO:0019243;GO:0019243;GO:0004416;GO:0046872;GO:0046872;GO:00	BP:methylglyoxal catabolic process to D-lactate via S-lactoyl-glutathione;BP:methylglyoxal catabolic process to D-lactate via S-lactoyl-glutathione;MF:hydroxyacylglutathione hydrolase activity;MF:metal ion binding;MF:metal ion binding;MF:hydroxyacylglutathione hydrolase activity;
BAB_RS20405	-	IS5/IS1182 family transposase	0.761	-0.394073176	0.004756919	0.024107226	no	down	mRNA	52.1	52.22	56.89	44.16	35.76	37.21	53.7367	39.0433333	COG3293	L:Replication, recombination and repair	------	------
BAB_RS29325	-	PleD family two-component system response regulator	0.838	-0.254205261	0.004940884	0.02468905	no	down	mRNA	36.42	31.69	39.25	26.66	27.8	30.16	35.7867	28.2066667	ENOG410XNMH	T:Signal transduction mechanisms	GO:0000160;GO:0005622	BP:phosphorelay signal transduction system;CC:intracellular;
BAB_RS31430	-	IS3 family transposase	0.785	-0.350017707	0.004984791	0.024831216	no	down	mRNA	24.79	22.19	22.91	19.46	16.09	16.03	23.2967	17.1933333	COG2801	L:Replication, recombination and repair	GO:0015074;GO:0006313;GO:0015074;GO:0003677;GO:0004803;GO:00	BP:DNA integration;BP:transposition, DNA-mediated;BP:DNA integration;MF:DNA binding;MF:transposase activity;MF:nucleic acid binding;
BAB_RS21080	-	acetyl-CoA hydrolase	0.869	-0.202684101	0.00503852	0.025060006	no	down	mRNA	115.29	102.31	108.71	84.97	92.24	89.38	108.77	88.8633333	COG0427	C:Energy production and conversion	GO:0006084;GO:0006084;GO:0016740;GO:0016787;GO:0016740;GO:00	BP:acetyl-CoA metabolic process;BP:acetyl-CoA metabolic process;MF:transferase activity;MF:hydrolase activity;MF:transferase activity;MF:acetate CoA-transferase activity;
BAB_RS30315	-	iron permease FTR1	0.822	-0.283351365	0.005073895	0.025196946	no	down	mRNA	296.32	306.26	274.82	210.08	246.04	226.94	292.467	227.686667	COG0672	P:Inorganic ion transport and metabolism	GO:0033573;GO:0005381	CC:high-affinity iron permease complex;MF:iron ion transmembrane transporter activity;
BAB_RS21835	-	30S ribosomal protein S5	0.876	-0.190619715	0.005085026	0.025213252	no	down	mRNA	1195.54	1078.83	1179.8	923.51	1021.83	931.04	1151.39	958.793333	COG0098	J:Translation, ribosomal structure and biogenesis	GO:0006412;GO:0006412;GO:0006412;GO:0006412;GO:0015935;GO:00	BP:translation;BP:translation;BP:translation;BP:translation;CC:small ribosomal subunit;CC:small ribosomal subunit;CC:small ribosomal subunit;CC:small ribosomal subunit;MF:structural constituent of ribosome;MF:rRNA binding;MF:rRNA binding;MF:structural constituent of ribosome;MF:structural constituent of ribosome;MF:rRNA binding;MF:structural constituent of ribosome;MF:rRNA binding;
BAB_RS32915	-	hypothetical protein	0.793	-0.333845401	0.005171505	0.02552388	no	down	mRNA	170.53	137.15	133.51	108.43	117.73	103.42	147.063	109.86	COG4103	G:Carbohydrate transport and metabolism	------	------
BAB_RS31505	-	PilZ domain-containing protein	0.839	-0.252473864	0.00518784	0.02556523	no	down	mRNA	83.21	74.54	83.51	60.36	61.65	69.42	80.42	63.81	ENOG410XXR3	U:Intracellular trafficking, secretion, and vesicular	GO:0035438	MF:cyclic-di-GMP binding;
BAB_RS33335	-	hypothetical protein	0.763	-0.390292261	0.005221575	0.025692068	no	down	mRNA	281.65	228.08	315.59	172.04	218.22	210.23	275.107	200.163333	------	------	------	------
BAB_RS22525	-	urease accessory protein ureD 2	0.848	-0.238090726	0.00535796	0.026282636	no	down	mRNA	106.96	97.09	95.71	80.09	78.3	81.57	99.92	79.9866667	COG0829	S:Function unknown	GO:0006807;GO:0006807;GO:0005737;GO:0005737;GO:0016151;GO:00	BP:nitrogen compound metabolic process;BP:nitrogen compound metabolic process;CC:cytoplasm;CC:cytoplasm;MF:nickel cation binding;MF:nickel cation binding;
BAB_RS18435	-	DUF1499 domain-containing protein	0.863	-0.213063093	0.005392724	0.026332558	no	down	mRNA	49.12	48.73	53.5	41.4	39.99	41.81	50.45	41.0666667	ENOG4111R8M;ENOG410XQW8	S:Function unknown;	GO:0016021	CC:integral component of membrane;
BAB_RS21970	-	50S ribosomal protein L10	0.872	-0.19693353	0.005659774	0.027182141	no	down	mRNA	2403.78	2277.1	2407.21	1979.12	2032.04	1839.4	2362.7	1950.18667	COG0244	J:Translation, ribosomal structure and biogenesis	GO:0042254;GO:0006412;GO:0042254;GO:0006412;GO:0042254;GO:00	BP:ribosome biogenesis;BP:translation;BP:ribosome biogenesis;BP:translation;BP:ribosome biogenesis;BP:translation;CC:ribosome;CC:ribosome;CC:ribosome;MF:rRNA binding;MF:structural constituent of ribosome;MF:large ribosomal subunit rRNA binding;MF:rRNA binding;MF:structural constituent of ribosome;MF:large ribosomal subunit rRNA binding;MF:structural constituent of ribosome;
BAB_RS23055	-	YcgN family cysteine cluster protein	0.824	-0.279880784	0.00561814	0.027185365	no	down	mRNA	41.71	37.14	46.05	32.59	32.46	32.14	41.6333	32.3966667	COG2983	S:Function unknown	------	------
BAB_RS16650	-	LPS export ABC transporter periplasmic protein LptC	0.865	-0.209656575	0.005645579	0.027195271	no	down	mRNA	339.92	304.36	327.38	279.66	267.15	245.8	323.887	264.203333	COG5375	S:Function unknown	GO:0005887;GO:0015221	CC:integral component of plasma membrane;MF:lipopolysaccharide transmembrane transporter activity;

BAB_RS23890	-	RluA family pseudouridine synthase	0.858	-0.221220187	0.005654739	0.027198619	no	down	mRNA	81.59	73.79	79.28	62.22	59.58	67.72	78.22	63.1733333	COG0564	C:Energy production and conversion	GO:0001522;GO:0001522;GO:0016787;GO:0003723;GO:0009982;GO:00	BP:pseudouridine synthesis;BP:pseudouridine synthesis;MF:hydrolase activity;MF:RNA binding;MF:pseudouridine synthase activity;MF:hydrolase activity;MF:lyase activity;MF:RNA binding;MF:pseudouridine synthase activity;
BAB_RS21815	-	adenylate kinase	0.864	-0.210968751	0.005632137	0.027212113	no	down	mRNA	481.47	434.22	457.38	386.21	389.62	345.28	457.69	373.703333	COG0563	F:Nucleotide transport and metabolism	GO:0044209;GO:0044209;GO:0044209;GO:0005737;GO:0005737;GO:00	BP:AMP salvage;BP:AMP salvage;BP:AMP salvage;CC:cytoplasm;CC:cytoplasm;CC:cytoplasm;MF:lyase activity;MF:adenylate kinase activity;MF:ATP binding;MF:adenylate kinase activity;MF:ATP binding;MF:lyase activity;MF:ATP binding;MF:adenylate kinase activity;
BAB_RS27770	-	gfo/Idh/MocA family oxidoreductase	0.855	-0.225932597	0.005641496	0.027216406	no	down	mRNA	122.74	112.32	132.48	91.61	105.1	99.61	122.513	98.7733333	COG0673	S:Function unknown	GO:0016491;GO:0019151;GO:0016491;GO:0019151	MF:oxidoreductase activity;MF:galactose 1-dehydrogenase activity;MF:oxidoreductase activity;MF:galactose 1-dehydrogenase activity;
BAB_RS22370	-	serine hydrolase family protein	0.82	-0.286060925	0.005694451	0.027307869	no	down	mRNA	73.77	68.02	68.77	52.43	54.2	57.5	70.1867	54.71	COG3545	S:Function unknown	GO:0016787	MF:hydrolase activity;
BAB_RS26520	-	MFS transporter	0.764	-0.38764127	0.005756579	0.027523641	no	down	mRNA	5.12	4.97	5.72	3.8	3.62	3.99	5.27	3.80333333	COG1071	S:Function unknown	GO:0016624;GO:0003863	MF:oxidoreductase activity, acting on the aldehyde or oxo group of donors, disulfide as acceptor;MF:3-methyl-2-oxobutanoate dehydrogenas;
BAB_RS23770	-	dihydroxyacetone kinase subunit DhaK	0.826	-0.275563617	0.005767776	0.027536201	no	down	mRNA	84.09	76.84	74.99	55.8	64.69	63.29	78.64	61.26	COG2376	G:Carbohydrate transport and metabolism	GO:0006071;GO:0004371	BP:glycerol metabolic process;MF:glycerone kinase activity;
BAB_RS30780	-	isoprenylcysteine carboxylmethyltransferase family protein	0.863	-0.212332594	0.005756175	0.027562727	no	down	mRNA	371.63	362.86	356.1	295.06	315.3	284.19	363.53	298.183333	COG2020	Q:Secondary metabolites biosynthesis, transport a	GO:0016021;GO:0016021;GO:0004671;GO:0004671	CC:integral component of membrane;CC:integral component of membrane;MF:protein C-terminal S-isoprenylcysteine carboxyl O-methyltransferase activity;MF:protein C-terminal S-isoprenylcysteine carboxyl O-methyltransferase activity;
BAB_RS24115	-	Holliday junction branch migration DNA helicase RuvB	0.858	-0.22175927	0.005888998	0.027907597	no	down	mRNA	68.76	71.45	72.27	56.96	57.42	57.96	70.8267	57.4466667	COG2255	L:Replication, recombination and repair	GO:0006310;GO:0006281;GO:0009432;GO:0006310;GO:0006281;GO:00	BP:DNA recombination;BP:DNA repair;BP:SOS response;BP:DNA recombination;BP:DNA repair;BP:SOS response;BP:DNA recombination;BP:DNA repair;BP:SOS response;MF:four-way junction helicase activity;MF:transferase activity;MF:DNA binding;MF:ATP binding;MF:four-way junction helicase activity;MF:DNA binding;MF:ATP binding;MF:ATP binding;MF:DNA binding;MF:four-way junction helicase activity;
BAB_RS25240	-	LysR family transcriptional regulator	0.637	-0.650100199	0.00592196	0.028022469	no	down	mRNA	22.6	15.05	18.23	8.06	11.34	13.89	18.6267	11.0966667	COG0583	S:Function unknown	GO:0006351;GO:0003700;GO:0003677	BP:transcription, DNA-templated;MF:transcription factor activity, sequence-specific DNA binding;MF:DNA binding;
BAB_RS17775	-	cadmium-translocating P-type ATPase	0.892	-0.165666406	0.005989329	0.028216592	no	down	mRNA	221.53	210.97	229.13	187.98	179.92	187.23	220.543	185.043333	COG2217	P:Inorganic ion transport and metabolism	GO:0030001;GO:0030001;GO:0016021;GO:0005886;GO:0016021;GO:00	BP:metal ion transport;BP:metal ion transport;CC:integral component of membrane;CC:plasma membrane;CC:integral component of membrane;CC:plasma membrane;MF:cation-transporting ATPase activity;MF:nucleotide binding;MF:metal ion binding;MF:dodecanoyl-[acyl-carrier-protein] hydrolase activity;MF:cation-transporting ATPase activity;MF:nucleotide binding;MF:metal ion binding;
BAB_RS26085	-	DUF1330 domain-containing protein	0.844	-0.245526545	0.005985332	0.028239167	no	down	mRNA	46.87	43.1	48.27	33.51	38.36	38	46.08	36.6233333	COG5470	S:Function unknown	------	------
BAB_RS18075	-	DUF374 domain-containing protein	0.885	-0.176901907	0.006026537	0.028267536	no	down	mRNA	175.77	164.29	179.64	145.29	139.97	148.39	173.233	144.55	COG2121	S:Function unknown	------	------
BAB_RS26290	-	beta-hydroxydecanoyl-ACP dehydratase	0.892	-0.165023499	0.006018603	0.028271594	no	down	mRNA	200.74	190.5	203.6	166.74	166.56	167.18	198.28	166.826667	COG0764	J:Translation, ribosomal structure and biogenesis	GO:0006633;GO:0006633;GO:0005737;GO:0005737;GO:0008693;GO:00	BP:fatty acid biosynthetic process;BP:fatty acid biosynthetic process;CC:cytoplasm;CC:cytoplasm;MF:3-hydroxydecanoyl-[acyl-carrier-protein] dehydratase activity;MF:3-hydroxyoctanoyl-[acyl-carrier-protein] dehydratase activity;MF:trans-2-decenoyl-acyl-carrier-protein isomerase activity;MF:transferase activity, transferring glycosyl groups;MF:3-hydroxyoctanoyl-[acyl-carrier-protein] dehydratase activity;MF:3-hydroxydecanoyl-[acyl-carrier-protein] dehydratase activity;MF:trans-2-decenoyl-acyl-carrier-protein isomerase activity;
BAB_RS26230	-	ribosome maturation factor	0.84	-0.251349468	0.006012569	0.028284601	no	down	mRNA	148.82	131.73	148.12	123.88	109.85	107.15	142.89	113.626667	COG0779	S:Function unknown	GO:0042274;GO:0042274;GO:0005737;GO:0005737	BP:ribosomal small subunit biogenesis;BP:ribosomal small subunit biogenesis;CC:cytoplasm;CC:cytoplasm;
BAB_RS31075	-	tRNA epoxyqueuosine(34) reductase QueG	0.806	-0.311015969	0.006039884	0.028288846	no	down	mRNA	28.44	25.57	30.82	21.99	19.38	23.22	28.2767	21.53	COG1600	C:Energy production and conversion	GO:0008033;GO:0008616;GO:0008616;GO:0008033;GO:0005737;GO:00	BP:tRNA processing;BP:queuosine biosynthetic process;BP:queuosine biosynthetic process;BP:tRNA processing;CC:cytoplasm;CC:cytoplasm;MF:epoxyqueuosine reductase activity;MF:4 iron, 4 sulfur cluster binding;MF:metal ion binding;MF:4 iron, 4 sulfur cluster binding;MF:epoxyqueuosine reductase activity;MF:metal ion binding;
BAB_RS16110	-	cytochrome c family protein	0.882	-0.180994492	0.006084381	0.028455774	no	down	mRNA	313.34	285.96	305.88	261.75	249.38	241.97	301.727	251.033333	COG3474	C:Energy production and conversion	GO:0046872;GO:0020037;GO:0009055	MF:metal ion binding;MF:heme binding;MF:electron carrier activity;
BAB_RS29010	-	amino acid ABC transporter substrate-binding protein	0.861	-0.21567066	0.006150944	0.028683576	no	down	mRNA	327.99	285.86	294.77	238.64	240.31	260.34	302.873	246.43	ENOG410XSV7;COG0834	E:Amino acid transport and metabolism;	------	------
BAB_RS16845	-	DNA-binding response regulator	0.726	-0.462735756	0.006178637	0.028770957	no	down	mRNA	15.25	17.64	19.4	12.9	11.71	11.65	17.43	12.0866667	COG2197;ENOG4111ZEJ	T:Signal transduction mechanisms;	GO:0006355;GO:0006351;GO:0006355;GO:0006351;GO:0003677;GO:00	BP:regulation of transcription, DNA-templated;BP:transcription, DNA-templated;BP:regulation of transcription, DNA-templated;BP:transcription, DNA-templated;MF:DNA binding;MF:DNA binding;
BAB_RS21000	-	aminoacetone oxidase family FAD-binding enzyme	0.805	-0.313150384	0.006262734	0.028952753	no	down	mRNA	39.5	44.8	41.42	29.16	33.32	33.3	41.9067	31.9266667	COG2081	S:Function unknown	------	------
BAB_RS17750	-	LysR family transcriptional regulator	0.722	-0.469611202	0.006273669	0.028961634	no	down	mRNA	20.21	18.99	21.04	16.77	11.82	12.58	20.08	13.7233333	ENOG410XNQA	K:Transcription	GO:0006351;GO:0006351;GO:0003700;GO:0003677;GO:0003677;GO:00	BP:transcription, DNA-templated;BP:transcription, DNA-templated;MF:transcription factor activity, sequence-specific DNA binding;MF:DNA binding;MF:DNA binding;MF:transcription factor activity, sequence-specific DNA binding;
BAB_RS31585	-	flagellar transcriptional regulator FtcR	0.746	-0.423566493	0.006334492	0.029200462	no	down	mRNA	30.03	25.85	31.34	19.51	17.96	24.23	29.0733	20.5666667	ENOG410XRGP	K:Transcription	GO:0000160;GO:0006355;GO:0006351;GO:0000160;GO:0006351;GO:00	BP:phosphorelay signal transduction system;BP:regulation of transcription, DNA-templated;BP:transcription, DNA-templated;BP:phosphorelay signal transduction system;BP:regulation of transcription, DNA-templated;BP:phosphorelay signal transduction system;BP:regulation of transcription, DNA-templated;BP:transcription, DNA-templated;CC:intracellular;CC:intracellular;CC:intracellular;MF:DNA binding;MF:DNA binding;MF:DNA binding;
BAB_RS20595	-	DUF971 domain-containing protein	0.794	-0.333694676	0.006407467	0.029452347	no	down	mRNA	39.89	31.16	37.97	26.5	26.13	28.92	36.34	27.1833333	COG3536	S:Function unknown	GO:0016853	MF:isomerase activity;
BAB_RS28455	-	LysR family transcriptional regulator	0.682	-0.551843233	0.006467199	0.029684442	no	down	mRNA	10.3	8.92	9.24	5.46	5.39	7.48	9.48667	6.11	ENOG410YGJ0	K:Transcription	GO:0006351;GO:0003700;GO:0003677	BP:transcription, DNA-templated;MF:transcription factor activity, sequence-specific DNA binding;MF:DNA binding;
BAB_RS19725	-	phosphate acyltransferase	0.848	-0.237623104	0.006678463	0.030350637	no	down	mRNA	100	93.49	101.09	84.75	79.02	71.86	98.1933	78.5433333	COG0416	I:Lipid transport and metabolism	GO:0006633;GO:0008654;GO:0006633;GO:0008654;GO:0005737;GO:00	BP:fatty acid biosynthetic process;BP:phospholipid biosynthetic process;BP:fatty acid biosynthetic process;BP:phospholipid biosynthetic process;CC:cytoplasm;CC:cytoplasm;MF:transferase activity, transferring acyl groups other than amino-acyl groups;MF:transferase activity, transferring acyl groups other than amino-acyl groups;
BAB_RS26210	-	apolipoprotein N-acyltransferase	0.881	-0.18219459	0.006646901	0.03037908	no	down	mRNA	64.3	61.15	66.67	51.97	54.45	53.02	64.04	53.1466667	COG0815	M:Cell wall/membrane/envelope biogenesis	GO:0042158;GO:0042158;GO:0016021;GO:0005886;GO:0016021;GO:00	BP:lipoprotein biosynthetic process;BP:lipoprotein biosynthetic process;CC:integral component of membrane;CC:plasma membrane;CC:integral component of membrane;CC:plasma membrane;MF:isomerase activity;MF:N-acyltransferase activity;MF:N-acyltransferase activity;
BAB_RS17955	-	DUF2007 domain-containing protein	0.648	-0.6256109	0.006670792	0.030401778	no	down	mRNA	303.09	212.03	174.52	135.06	145.63	155.28	229.88	145.323333	ENOG411263M	S:Function unknown	GO:0065003;GO:0019627;GO:0065003;GO:0019627;GO:0016151;GO:00	BP:macromolecular complex assembly;BP:urea metabolic process;BP:macromolecular complex assembly;BP:urea metabolic process;MF:nickel cation binding;MF:nickel cation binding;
BAB_RS24535	-	hypothetical protein	0.858	-0.221162029	0.006644672	0.030412154	no	down	mRNA	94.46	85.77	93.52	74.93	75.05	72.52	91.25	74.1666667	ENOG4111X9M	S:Function unknown	GO:0016021	CC:integral component of membrane;
BAB_RS24620	-	membrane protein	0.859	-0.218624484	0.006736621	0.030485582	no	down	mRNA	304.08	327.56	338.82	276.41	254	259.75	323.487	263.386667	COG1512	S:Function unknown	GO:0016021	CC:integral component of membrane;
BAB_RS28565	-	glucose-6-phosphate dehydrogenase	0.886	-0.174596844	0.006800961	0.030690294	no	down	mRNA	248.89	221.82	238.89	191.66	197.72	202.68	236.533	197.353333	COG0364	G:Carbohydrate transport and metabolism	GO:0006006;GO:0006098;GO:0006098;GO:0006006;GO:0004345;GO:00	BP:glucose metabolic process;BP:pentose-phosphate shunt;BP:pentose-phosphate shunt;BP:glucose metabolic process;MF:glucose-6-phosphate dehydrogenase activity;MF:NADP binding;MF:glucose-6-phosphate dehydrogenase activity;MF:NADP binding;
BAB_RS21560	-	zinc metalloprotease	0.864	-0.210611007	0.00683102	0.030782702	no	down	mRNA	78.98	80.81	80.33	63.15	65.2	67.56	80.04	65.3033333	COG0750	M:Cell wall/membrane/envelope biogenesis	GO:0016021;GO:0005886;GO:0016021;GO:0005886;GO:0046872;GO:00	CC:integral component of membrane;CC:plasma membrane;CC:integral component of membrane;CC:plasma membrane;MF:metal ion binding;MF:metalloendopeptidase activity;MF:metal ion binding;MF:metalloendopeptidase activity;
BAB_RS18130	-	TlyA family rRNA (cytidine-2'-O)-methyltransferase	0.774	-0.369850577	0.007039957	0.031547254	no	down	mRNA	22.56	22.8	25.25	15.67	18.55	17.58	23.5367	17.2666667	COG1189	J:Translation, ribosomal structure and biogenesis	GO:0051301;GO:0008168;GO:0003723	BP:cell division;MF:methyltransferase activity;MF:RNA binding;
BAB_RS22690	-	thymidylate synthase	0.846	-0.240910014	0.007060002	0.031593018	no	down	mRNA	124.09	111.14	109.59	96.22	88.49	90.79	114.94	91.8333333	COG0207	F:Nucleotide transport and metabolism	GO:0006235;GO:0006231;GO:0006235;GO:0006231;GO:0006231;GO:00	BP:dTTP biosynthetic process;BP:dTMP biosynthetic process;BP:dTTP biosynthetic process;BP:dTMP biosynthetic process;BP:dTMP biosynthetic process;BP:dTTP biosynthetic process;CC:cytoplasm;CC:cytoplasm;CC:cytoplasm;MF:thymidylate synthase activity;MF:oxidoreductase activity;MF:thymidylate synthase activity;MF:thymidylate synthase activity;
BAB_RS20765	-	septal ring lytic transglycosylase RlpA family protein	0.868	-0.204978783	0.007130558	0.031731972	no	down	mRNA	210.66	227.62	229.78	185.21	175.98	186.44	222.687	182.543333	COG0797	M:Cell wall/membrane/envelope biogenesis	GO:0071555;GO:0000270;GO:0071555;GO:0000270;GO:0016829;GO:00	BP:cell wall organization;BP:peptidoglycan metabolic process;BP:cell wall organization;BP:peptidoglycan metabolic process;MF:lyase activity;MF:lytic endotransglycosylase activity;MF:lyase activity;MF:lytic endotransglycosylase activity;
BAB_RS32520	-	hypothetical protein	0.794	-0.331881677	0.0072174	0.031810024	no	down	mRNA	33.2	29.11	31.72	23.23	25.48	21.62	31.3433	23.4433333	ENOG410Y5WW	S:Function unknown	------	------
BAB_RS22635	-	ATP-binding protein	0.877	-0.18896898	0.007165388	0.031842867	no	down	mRNA	71.83	66.49	74.43	60.19	59.48	55.97	70.9167	58.5466667	COG0507	L:Replication, recombination and repair	GO:0008854;GO:0005524	MF:exodeoxyribonuclease V activity;MF:ATP binding;
BAB_RS16695	-	endonuclease III	0.845	-0.242539528	0.007216183	0.031848346	no	down	mRNA	48.49	49.33	46.89	39.24	38.85	37.36	48.2367	38.4833333	COG0177	L:Replication, recombination and repair	GO:0006284;GO:0006284;GO:0051539;GO:0140078;GO:0004519;GO:00	BP:base-excision repair;BP:base-excision repair;MF:4 iron, 4 sulfur cluster binding;MF:old GO;MF:endonuclease activity;MF:DNA N-glycosylase activity;MF:DNA binding;MF:metal ion binding;MF:4 iron, 4 sulfur cluster binding;MF:old GO;MF:DNA binding;MF:DNA N-glycosylase activity;MF:endonuclease activity;MF:metal ion binding;
BAB_RS29515	-	phosphatidylcholine/phosphatidylserine synthase	0.872	-0.198408177	0.007337737	0.032163916	no	down	mRNA	138.34	119.84	133.08	106.35	104.39	111.03	130.42	107.256667	COG1183	S:Function unknown	GO:0008654;GO:0016021;GO:0005886;GO:0050520	BP:phospholipid biosynthetic process;CC:integral component of membrane;CC:plasma membrane;MF:phosphatidylcholine synthase activity;
BAB_RS33370	-	hypothetical protein	0.772	-0.374168893	0.007319332	0.032171016	no	down	mRNA	77.4	81.56	83.38	60.04	55.06	66.19	80.78	60.43	------	------	------	------
BAB_RS21010	-	mechanosensitive ion channel family protein	0.855	-0.225536384	0.007479111	0.032649978	no	down	mRNA	73.77	67.58	66.09	56.64	54.24	56.21	69.1467	55.6966667	COG0668	M:Cell wall/membrane/envelope biogenesis	GO:0071470;GO:0071470;GO:0016021;GO:0016021;GO:0008381;GO:00	BP:cellular response to osmotic stress;BP:cellular response to osmotic stress;CC:integral component of membrane;CC:integral component of membrane;MF:mechanically-gated ion channel activity;MF:mechanically-gated ion channel activity;
BAB_RS18830	-	penicillin-binding protein 1A	0.888	-0.171324522	0.007521567	0.032790766	no	down	mRNA	143.22	138.4	145.5	115.53	124.8	116.8	142.373	119.043333	COG0744	S:Function unknown	GO:0008658;GO:0016740;GO:0016740;GO:0008658	MF:penicillin binding;MF:transferase activity;MF:transferase activity;MF:penicillin binding;
BAB_RS30605	-	DUF2478 domain-containing protein	0.8	-0.322213032	0.007572311	0.032833786	no	down	mRNA	130.07	139.69	155.04	106.14	96.3	122.07	141.6	108.17	ENOG41125H8	S:Function unknown	------	------
BAB_RS24670	-	LysR family transcriptional regulator	0.844	-0.245104573	0.007554471	0.032845082	no	down	mRNA	24.37	23.28	27.94	19.91	20.78	19.39	25.1967	20.0266667	ENOG410XSUY	K:Transcription	GO:0006351;GO:0006351;GO:0003700;GO:0003677;GO:0003677;GO:00	BP:transcription, DNA-templated;BP:transcription, DNA-templated;MF:transcription factor activity, sequence-specific DNA binding;MF:DNA binding;MF:DNA binding;MF:transcription factor activity, sequence-specific DNA binding;
BAB_RS28940	-	TIGR01244 family phosphatase	0.862	-0.213948459	0.007605848	0.032934758	no	down	mRNA	256.19	220.39	231.23	201.84	186.93	187.06	235.937	191.943333	COG3453	S:Function unknown	GO:0016787;GO:0016787;GO:0016491	MF:hydrolase activity;MF:hydrolase activity;MF:oxidoreductase activity;
BAB_RS20885	-	hypothetical protein	0.811	-0.302619673	0.007629338	0.032992012	no	down	mRNA	40.19	38.45	44.47	29.34	29.42	35.76	41.0367	31.5066667	COG0008	J:Translation, ribosomal structure and biogenesis	------	------
BAB_RS29365	-	DNA-protecting protein DprA	0.825	-0.278319728	0.007836299	0.033705529	no	down	mRNA	17.62	16.37	19.68	14.51	14.2	12.93	17.89	13.88	COG0758	L:Replication, recombination and repair	GO:0009294	BP:DNA mediated transformation;
BAB_RS21240	-	LysR family transcriptional regulator	0.858	-0.221341993	0.007916611	0.034005443	no	down	mRNA	94.56	90.38	94.74	68.25	79.29	78.59	93.2267	75.3766667	COG0583;ENOG410XS6G	C:Energy production and conversion;K:Transcripti	------	------
BAB_RS29160	-	3-carboxy-cis%2Ccis-muconate cycloisomerase	0.819	-0.288397476	0.007956234	0.034130013	no	down	mRNA	23.13	19.78	24.96	17.33	17.9	17.06	22.6233	17.43	COG0015	F:Nucleotide transport and metabolism	GO:0019619;GO:0019619;GO:0047472;GO:0016829;GO:0047472;GO:00	BP:3,4-dihydroxybenzoate catabolic process;BP:3,4-dihydroxybenzoate catabolic process;MF:3-carboxy-cis,cis-muconate cycloisomerase activity;MF:lyase activity;MF:3-carboxy-cis,cis-muconate cycloisomerase activity;MF:glucuronate-2-sulfatase activity;
BAB_RS23280	-	class I SAM-dependent methyltransferase	0.807	-0.309749759	0.008047994	0.034431698	no	down	mRNA	19.18	17.04	19	14.59	12.66	14.76	18.4067	14.0033333	COG1565	S:Function unknown	GO:0008168	MF:methyltransferase activity;
BAB_RS30405	-	EAL domain-containing protein	0.862	-0.214782608	0.008065956	0.034462655	no	down	mRNA	58.44	48.25	53.83	42.76	43.05	44.11	53.5067	43.3066667	COG4943	K:Transcription	GO:0016021	CC:integral component of membrane;
BAB_RS33030	-	hypothetical protein	0.851	-0.232035183	0.008093787	0.034535641	no	down	mRNA	104.41	85.25	101.6	76.79	82.56	73.9	97.0867	77.75	------	------	------	------
BAB_RS21545	-	beta-hydroxyacyl-ACP dehydratase	0.871	-0.199994408	0.008172515	0.034779194	no	down	mRNA	315.25	278.13	289.71	236.01	239.75	257.71	294.363	244.49	COG0764	I:Lipid transport and metabolism	GO:0006633;GO:0009245;GO:0006633;GO:0009245;GO:0006633;GO:00	BP:fatty acid biosynthetic process;BP:lipid A biosynthetic process;BP:fatty acid biosynthetic process;BP:lipid A biosynthetic process;BP:fatty acid biosynthetic process;BP:lipid A biosynthetic process;CC:cytoplasm;CC:cytoplasm;CC:cytoplasm;MF:3-hydroxyoctanoyl-[acyl-carrier-protein] dehydratase activity;MF:3-hydroxyoctanoyl-[acyl-carrier-protein] dehydratase activity;MF:3-hydroxyoctanoyl-[acyl-carrier-protein] dehydratase activity;MF:transferase activity, transferring glycosyl groups;
BAB_RS18230	-	acyl carrier protein	0.778	-0.36196698	0.008212324	0.034856272	no	down	mRNA	4022.03	2844.73	3328.09	2649.97	2670.64	2568.83	3398.28	2629.81333	COG0236	I:Lipid transport and metabolism	GO:0006633;GO:0005737;GO:0005737;GO:0000036;GO:0031177;GO:00	BP:fatty acid biosynthetic process;CC:cytoplasm;CC:cytoplasm;MF:ACP phosphopantetheine attachment site binding involved in fatty acid biosynthetic process;MF:phosphopantetheine binding;MF:phosphopantetheine binding;MF:ACP phosphopantetheine attachment site binding involved in fatty acid biosynthetic process;
BAB_RS16660	-	LPS export ABC transporter ATP-binding protein	0.863	-0.212705447	0.008249812	0.034969192	no	down	mRNA	140.77	136.03	132.93	110.46	107.33	116.86	136.577	111.55	COG1137	S:Function unknown	GO:0055085;GO:0055085;GO:0043190;GO:0043190;GO:0016887;GO:00	BP:transmembrane transport;BP:transmembrane transport;CC:ATP-binding cassett;CC:ATP-binding cassett;MF:ATPase activity;MF:ATP binding;MF:ATP binding;MF:ATPase activity;
BAB_RS26410	-	BMP family ABC transporter substrate-binding protein	0.838	-0.254343114	0.008420145	0.035550492	no	down	mRNA	187.72	165.38	159.56	124.85	140.99	139.58	170.887	135.14	COG1744	S:Function unknown	GO:0008643;GO:0005886;GO:0005886	BP:carbohydrate transport;CC:plasma membrane;CC:plasma membrane;
BAB_RS28165	-	DeoR/GlpR transcriptional regulator	0.881	-0.183343725	0.008444602	0.035560298	no	down	mRNA	109.43	100.94	103.87	86.96	85.62	88.16	104.747	86.9133333	COG1349	S:Function unknown	GO:0006351;GO:0006351;GO:0006351;GO:0005622;GO:0005622;GO:00	BP:transcription, DNA-templated;BP:transcription, DNA-templated;BP:transcription, DNA-templated;CC:intracellular;CC:intracellular;CC:intracellular;MF:transcription factor activity, sequence-specific DNA binding;MF:DNA binding;MF:DNA binding;MF:transcription factor activity, sequence-specific DNA binding;MF:transferase activity;MF:DNA binding;MF:transcription factor activity, sequence-specific DNA binding;
BAB_RS19615	-	exopolyphosphatase	0.861	-0.216679108	0.008413023	0.035567161	no	down	mRNA	66.84	73.43	76.73	58.35	58.09	59.91	72.3333	58.7833333	COG0248;COG0248	F:Nucleotide transport and metabolism;P:Inorganic	GO:0006793;GO:0004309	BP:phosphorus metabolic process;MF:exopolyphosphatase activity;
BAB_RS25495	-	surface protein	0.878	-0.187093252	0.008444301	0.035605694	no	down	mRNA	44.06	44.04	46.31	37.89	37.24	36.07	44.8033	37.0666667	ENOG410XQE6;ENOG410Z37M	S:Function unknown;	------	------
BAB_RS17085	-	L-fuconate dehydratase	0.879	-0.186497864	0.008501403	0.035659281	no	down	mRNA	109.12	100.93	105.48	81.93	87.96	91.11	105.177	87	COG4948	S:Function unknown	GO:0009063;GO:0016052;GO:0016052;GO:0009063;GO:0050023;GO:00	BP:cellular amino acid catabolic process;BP:carbohydrate catabolic process;BP:carbohydrate catabolic process;BP:cellular amino acid catabolic process;MF:L-fuconate dehydratase activity;MF:metal ion binding;MF:L-fuconate dehydratase activity;MF:metal ion binding;
BAB_RS30475	-	ABC transporter permease	0.832	-0.265492854	0.008493421	0.035672367	no	down	mRNA	54.53	44.12	48.98	36	38.17	41.29	49.21	38.4866667	COG1176	S:Function unknown	GO:0055085;GO:0016021;GO:0005886	BP:transmembrane transport;CC:integral component of membrane;CC:plasma membrane;
BAB_RS24615	-	TPM domain-containing protein	0.87	-0.200101109	0.008585844	0.035826386	no	down	mRNA	152.69	157.23	156.14	122.32	131.37	130.29	155.353	127.993333	COG3762	S:Function unknown	GO:0016021	CC:integral component of membrane;
BAB_RS30765	-	formylglycine-generating enzyme family protein	0.714	-0.485126781	0.008634055	0.035934222	no	down	mRNA	91.15	84.13	71.23	49.79	69.48	46.28	82.17	55.1833333	COG1262	S:Function unknown	------	------
BAB_RS21440	-	pyruvate dehydrogenase (acetyl-transferring) E1 component subunit alpha	0.876	-0.190667957	0.00869238	0.036083485	no	down	mRNA	465.29	423.46	436.74	342.9	368.3	385.27	441.83	365.49	COG1071	C:Energy production and conversion	GO:0006086;GO:0006086;GO:0043231;GO:0043231;GO:0004739;GO:00	BP:acetyl-CoA biosynthetic process from pyruvate;BP:acetyl-CoA biosynthetic process from pyruvate;CC:intracellular membrane-bounded organelle;CC:intracellular membrane-bounded organelle;MF:pyruvate dehydrogenas;MF:transaminase activity;MF:pyruvate dehydrogenas;
BAB_RS19855	-	NADH-quinone oxidoreductase subunit A	0.819	-0.287752793	0.008714848	0.036130074	no	down	mRNA	696.2	552.43	537.69	508.48	443.16	444.06	595.44	465.233333	COG0838	C:Energy production and conversion	GO:0016021;GO:0005886;GO:0016021;GO:0005886;GO:0008137;GO:00	CC:integral component of membrane;CC:plasma membrane;CC:integral component of membrane;CC:plasma membrane;MF:NADH dehydrogenas;MF:quinone binding;MF:NADH dehydrogenas;MF:quinone binding;
BAB_RS16985	-	DUF1794 domain-containing protein	0.869	-0.202231178	0.008775488	0.03633459	no	down	mRNA	143.49	126.26	141.22	104.75	114	118.37	136.99	112.373333	COG5514	S:Function unknown	------	------
BAB_RS29290	-	GGDEF domain-containing protein	0.721	-0.471803912	0.008835071	0.036534212	no	down	mRNA	19.27	22.44	20.35	13.83	14.83	14.12	20.6867	14.26	ENOG410XNMH	L:Replication, recombination and repair	GO:0016021	CC:integral component of membrane;
BAB_RS25700	-	dihydrodipicolinate synthase family protein	0.845	-0.242321321	0.008985823	0.037062196	no	down	mRNA	25.68	26.09	27.22	21.98	20.77	20.27	26.33	21.0066667	ENOG410XRSJ	S:Function unknown	------	------
BAB_RS23750	-	dihydroxyacetone kinase subunit L	0.815	-0.295539506	0.009023883	0.037076388	no	down	mRNA	37.31	29.25	42.19	27.78	28.02	27.33	36.25	27.71	COG2376	G:Carbohydrate transport and metabolism	GO:0006071;GO:0006071;GO:0004371;GO:0004371;GO:0016757	BP:glycerol metabolic process;BP:glycerol metabolic process;MF:glycerone kinase activity;MF:glycerone kinase activity;MF:transferase activity, transferring glycosyl groups;
BAB_RS19905	-	NADH-quinone oxidoreductase subunit K	0.889	-0.168995185	0.009077125	0.037200003	no	down	mRNA	1976.46	1735.36	1653.73	1536.61	1596.93	1519.04	1788.52	1550.86	COG0713	C:Energy production and conversion	GO:0042773;GO:0042773;GO:0016021;GO:0005886;GO:0016021;GO:00	BP:ATP synthesis coupled electron transport;BP:ATP synthesis coupled electron transport;CC:integral component of membrane;CC:plasma membrane;CC:integral component of membrane;CC:plasma membrane;MF:oxidoreductase activity, acting on NA;MF:quinone binding;MF:oxidoreductase activity, acting on NA;MF:quinone binding;MF:NADH dehydrogenas;
BAB_RS32515	-	autotransporter outer membrane beta-barrel domain-containing protein	0.845	-0.242798108	0.009263499	0.037723224	no	down	mRNA	1901.9	1589.6	1622.33	1327.49	1484.64	1279.28	1704.61	1363.80333	COG3637	M:Cell wall/membrane/envelope biogenesis	GO:0019867	CC:outer membrane;
BAB_RS29530	-	ABC transporter ATP-binding protein	0.862	-0.213712005	0.009429458	0.037965975	no	down	mRNA	60.25	60.87	69.15	54.97	49.51	50.19	63.4233	51.5566667	COG3845	C:Energy production and conversion	GO:0008643;GO:0016887;GO:0005524	BP:carbohydrate transport;MF:ATPase activity;MF:ATP binding;
BAB_RS21670	-	hypothetical protein	0.771	-0.374609692	0.009426612	0.038002139	no	down	mRNA	96.37	86.54	95.33	69.57	76.82	59.84	92.7467	68.7433333	------	------	------	------
BAB_RS29930	-	glyoxalase/bleomycin resistance/extradiol dioxygenase family protein	0.796	-0.329592589	0.009466747	0.038020823	no	down	mRNA	25.11	25.51	30.43	22.26	20.91	17.72	27.0167	20.2966667	COG3324	E:Amino acid transport and metabolism	GO:0051213	MF:dioxygenase activity;
BAB_RS23260	-	tRNA-binding protein	0.801	-0.319726687	0.00942534	0.0380926	no	down	mRNA	32.33	34.01	35.9	22.9	25.73	29.08	34.08	25.9033333	COG0073	J:Translation, ribosomal structure and biogenesis	GO:0000049	MF:tRNA binding;
BAB_RS24210	-	5-formyltetrahydrofolate cyclo-ligase	0.842	-0.248607487	0.009568687	0.038334404	no	down	mRNA	95.55	81.83	102.71	73.29	76.48	73.86	93.3633	74.5433333	COG0212	H:Coenzyme transport and metabolism	GO:0030272;GO:0005524;GO:0046872;GO:0030272;GO:0046872;GO:00	MF:5-formyltetrahydrofolate cyclo-ligase activity;MF:ATP binding;MF:metal ion binding;MF:5-formyltetrahydrofolate cyclo-ligase activity;MF:metal ion binding;MF:ATP binding;
BAB_RS23350	-	cytochrome b	0.852	-0.230866141	0.009635397	0.038553588	no	down	mRNA	731.21	606.24	626.67	496.69	532.61	548.63	654.707	525.976667	COG1290	C:Energy production and conversion	GO:0006122;GO:0006122;GO:0016021;GO:0045275;GO:0016021;GO:00	BP:mitochondrial electron transport, ubiquinol to cytochrome c;BP:mitochondrial electron transport, ubiquinol to cytochrome c;CC:integral component of membrane;CC:respiratory chain complex III;CC:integral component of membrane;CC:respiratory chain complex III;MF:ubiquinol-cytochrome-c reductase activity;MF:ubiquinol-cytochrome-c reductase activity;
BAB_RS31005	-	transglutaminase	0.828	-0.273045328	0.009973618	0.039610919	no	down	mRNA	40.44	38.06	43.95	31.93	31.11	33.16	40.8167	32.0666667	COG3672	E:Amino acid transport and metabolism	------	------
BAB_RS26120	-	DUF454 domain-containing protein	0.812	-0.299796173	0.010002937	0.039678319	no	down	mRNA	45.73	46.55	48.95	38.56	38.01	32.1	47.0767	36.2233333	COG2832	S:Function unknown	GO:0016021	CC:integral component of membrane;
BAB_RS22090	-	single-stranded-DNA-specific exonuclease RecJ	0.881	-0.183131558	0.010041484	0.039733112	no	down	mRNA	51.05	50.57	57.27	45.09	43.95	42.73	52.9633	43.9233333	COG0608	L:Replication, recombination and repair	GO:0006310;GO:0006281;GO:0008409;GO:0003676;GO:0016491	BP:DNA recombination;BP:DNA repair;MF:5'-3' exonuclease activity;MF:nucleic acid binding;MF:oxidoreductase activity;
BAB_RS24380	-	RNA polymerase factor sigma-32	0.898	-0.154654773	0.010093996	0.039793877	no	down	mRNA	342.34	317.85	337.62	278.48	278.66	287.04	332.603	281.393333	COG0568	S:Function unknown	GO:0006352;GO:0006352;GO:0003700;GO:0003677;GO:0016987;GO:00	BP:DNA-templated transcription, initiation;BP:DNA-templated transcription, initiation;MF:transcription factor activity, sequence-specific DNA binding;MF:DNA binding;MF:sigma factor activity;MF:sigma factor activity;MF:DNA binding;MF:transcription factor activity, sequence-specific DNA binding;
BAB_RS21205	-	hypothetical protein	0.876	-0.190249551	0.010107353	0.039797702	no	down	mRNA	163.29	137.42	147.97	123.93	125.28	120.63	149.56	123.28	ENOG411225P	S:Function unknown	------	------
BAB_RS17325	-	hypothetical protein	0.876	-0.190249551	0.010107353	0.039797702	no	down	mRNA	163.29	137.42	147.97	123.93	125.28	120.63	149.56	123.28	ENOG411225P	S:Function unknown	------	------
BAB_RS30255	-	3-oxoacyl-ACP reductase FabG	0.763	-0.389510307	0.010083833	0.039802647	no	down	mRNA	29.24	22.51	26.29	16.01	20.56	19.3	26.0133	18.6233333	ENOG410XQH3	I:Lipid transport and metabolism	GO:0004316;GO:0102132;GO:0102131;GO:0016491;GO:0016491;GO:00	MF:3-oxoacyl-[acyl-carrier-protein] reductas;MF:3-oxo-pimeloyl-[acp] methyl ester reductase activity;MF:old GO;MF:oxidoreductase activity;MF:oxidoreductase activity;MF:nucleotide binding;MF:oxidoreductase activity;
BAB_RS25660	-	3-ketoacyl-ACP reductase	0.544	-0.877084627	0.010075732	0.039819588	no	down	mRNA	3.54	3.08	2.65	1.55	1.5	1.65	3.09	1.56666667	ENOG4111HAD	S:Function unknown	GO:0004316;GO:0102132;GO:0102131;GO:0016491	MF:3-oxoacyl-[acyl-carrier-protein] reductas;MF:3-oxo-pimeloyl-[acp] methyl ester reductase activity;MF:old GO;MF:oxidoreductase activity;
BAB_RS30625	-	SCP2 domain-containing protein	0.847	-0.23909708	0.010453432	0.040859949	no	down	mRNA	682.34	666.73	621.28	477.05	586.5	525.77	656.783	529.773333	COG3154	I:Lipid transport and metabolism	------	------
BAB_RS19325	-	hypothetical protein	0.844	-0.244078966	0.0104618	0.040860067	no	down	mRNA	76.81	86.32	93.17	67.33	69.4	68.73	85.4333	68.4866667	ENOG410YTSI	S:Function unknown	GO:0016021	CC:integral component of membrane;
BAB_RS32745	-	hypothetical protein	0.744	-0.427509087	0.010599329	0.04127957	no	down	mRNA	30.37	27.33	31.42	19.83	21.5	22.21	29.7067	21.18	------	------	------	------
BAB_RS29005	-	amino acid ABC transporter permease	0.845	-0.242354364	0.010745799	0.041698374	no	down	mRNA	121.94	110.31	104.23	84.66	90.16	93.87	112.16	89.5633333	COG0765;ENOG410XR35	J:Translation, ribosomal structure and biogenesis;I	GO:0071705;GO:0071705;GO:0043190;GO:0043190;GO:0022857;GO:00	BP:nitrogen compound transport;BP:nitrogen compound transport;CC:ATP-binding cassett;CC:ATP-binding cassett;MF:transmembrane transporter activity;MF:transmembrane transporter activity;
BAB_RS25015	-	succinate dehydrogenase%2C cytochrome b556 subunit	0.869	-0.202357873	0.01105005	0.042519533	no	down	mRNA	306.93	250.88	287.13	241.83	225.56	226.5	281.647	231.296667	COG2009	C:Energy production and conversion	GO:0006099;GO:0006099;GO:0016021;GO:0045281;GO:0016021;GO:00	BP:tricarboxylic acid cycle;BP:tricarboxylic acid cycle;CC:integral component of membrane;CC:succinate dehydrogenase complex;CC:integral component of membrane;CC:succinate dehydrogenase complex;MF:electron carrier activity;MF:metal ion binding;MF:succinate dehydrogenase activity;MF:succinate dehydrogenase activity;MF:metal ion binding;MF:electron carrier activity;
BAB_RS24375	-	MOSC domain-containing protein	0.853	-0.230158621	0.01108125	0.042588583	no	down	mRNA	76.58	79.37	83.16	62.8	69.19	61.13	79.7033	64.3733333	COG2258	S:Function unknown	GO:0003824;GO:0030170;GO:0030151	MF:catalytic activity;MF:pyridoxal phosphate binding;MF:molybdenum ion binding;
BAB_RS19485	-	GNAT family N-acetyltransferase	0.857	-0.222575021	0.011183179	0.04277566	no	down	mRNA	156.95	132.85	132.74	112.32	113.81	114.9	140.847	113.676667	ENOG410XRJA;ENOG411015N	S:Function unknown;	GO:0016740	MF:transferase activity;
BAB_RS18800	-	sensor histidine kinase	0.867	-0.205808539	0.011173891	0.042842137	no	down	mRNA	239.35	247.01	254.41	191.53	192.96	221.14	246.923	201.876667	ENOG410XNMH	S:Function unknown	GO:0016021;GO:0005622;GO:0016021;GO:0005622;GO:0005524;GO:00	CC:integral component of membrane;CC:intracellular;CC:integral component of membrane;CC:intracellular;MF:ATP binding;MF:phosphorelay sensor kinase activity;MF:phosphorelay sensor kinase activity;MF:ATP binding;
BAB_RS23910	-	sensor histidine kinase	0.885	-0.17660941	0.011238892	0.042886649	no	down	mRNA	180.31	179.84	199.14	147.34	155.66	164.02	186.43	155.673333	COG3920	T:Signal transduction mechanisms	GO:0005622;GO:0000155	CC:intracellular;MF:phosphorelay sensor kinase activity;
BAB_RS28750	-	glycine/betaine ABC transporter substrate-binding protein	0.864	-0.211067565	0.011281022	0.042996351	no	down	mRNA	56.74	58.69	61.98	46.33	46.68	51.79	59.1367	48.2666667	COG2113	S:Function unknown	GO:0043190;GO:0043190;GO:0022857;GO:0022857	CC:ATP-binding cassett;CC:ATP-binding cassett;MF:transmembrane transporter activity;MF:transmembrane transporter activity;
BAB_RS32280	-	IS481 family transposase	0.832	-0.264807118	0.011432866	0.043523459	no	down	mRNA	25.69	27.54	29.27	20.99	22.71	21.27	27.5	21.6566667	COG2801	L:Replication, recombination and repair	GO:0015074;GO:0003677	BP:DNA integration;MF:DNA binding;
BAB_RS22960	-	alpha/beta hydrolase	0.82	-0.286531256	0.011619681	0.044077962	no	down	mRNA	25.27	25.65	26.45	19.81	21.53	18.54	25.79	19.96	COG2267	I:Lipid transport and metabolism	GO:0016787;GO:0016787;GO:0004177	MF:hydrolase activity;MF:hydrolase activity;MF:aminopeptidase activity;
BAB_RS18985	-	bifunctional [glutamate--ammonia ligase]-adenylyl-L-tyrosine phosphorylase/[glutamate--ammonia-ligase] adenylyltransferase	0.877	-0.189969156	0.011643136	0.044114855	no	down	mRNA	25.89	23.11	25.43	21.17	20.65	19.44	24.81	20.42	COG1391;COG1391	O:Posttranslational modification, protein turnover,	GO:0000820;GO:0008152;GO:0000287;GO:0008882;GO:0047388;GO:00	BP:regulation of glutamine family amino acid metabolic process;BP:metabolic process;MF:magnesium ion binding;MF:[glutamate-ammonia-ligase] adenylyltransferase activity;MF:adenylyl-[glutamate-ammonia ligase] hydrolase activity;MF:ligase activity;MF:ATP binding;
BAB_RS16395	-	hypothetical protein	0.838	-0.254674869	0.011687165	0.044177484	no	down	mRNA	91.01	100.96	111.18	74.05	81.58	85.46	101.05	80.3633333	ENOG410Z8C8	S:Function unknown	------	------
BAB_RS28205	-	hypothetical protein	0.802	-0.317798371	0.011743853	0.044235638	no	down	mRNA	17.6	16.06	16.85	13.04	12.74	12.41	16.8367	12.73	ENOG4111AFG	S:Function unknown	------	------
BAB_RS17725	-	allantoinase PuuE	0.863	-0.212992215	0.011730103	0.044235705	no	down	mRNA	52.2	51.3	51.9	38.97	44.52	42.9	51.8	42.13	COG3195	S:Function unknown	GO:0005975;GO:0019428;GO:0019428;GO:0005975;GO:0016810;GO:00	BP:carbohydrate metabolic process;BP:allantoin biosynthetic process;BP:allantoin biosynthetic process;BP:carbohydrate metabolic process;MF:hydrolase activity, acting on carbon-nitroge;MF:hydrolase activity, acting on carbon-nitroge;
BAB_RS18810	-	DUF1491 domain-containing protein	0.811	-0.301684901	0.0117295	0.044285409	no	down	mRNA	41.53	39.12	47.72	33.15	29.66	36.13	42.79	32.98	COG5447	S:Function unknown	------	------
BAB_RS30080	-	hypothetical protein	0.603	-0.729007802	0.011845852	0.044515465	no	down	mRNA	6.73	7.05	5.29	3.51	2.86	4.58	6.35667	3.65	COG0365	K:Transcription	GO:0008152;GO:0003824	BP:metabolic process;MF:catalytic activity;
BAB_RS25095	-	LysE family translocator	0.877	-0.188944816	0.01189277	0.044535514	no	down	mRNA	105.52	100.67	102.37	82.87	86.06	87.33	102.853	85.42	COG1280	S:Function unknown	GO:0006865;GO:0006865;GO:0016021;GO:0016020;GO:0005886;GO:00	BP:amino acid transport;BP:amino acid transport;CC:integral component of membrane;CC:membrane;CC:plasma membrane;CC:integral component of membrane;CC:plasma membrane;
BAB_RS30235	-	branched-chain amino acid ABC transporter substrate-binding protein	0.785	-0.34865494	0.011914582	0.044565252	no	down	mRNA	10.97	10.5	10.16	7.41	8.2	7.73	10.5433	7.78	COG0683	S:Function unknown	GO:0006865	BP:amino acid transport;
BAB_RS25530	-	50S ribosomal protein L28	0.815	-0.294497639	0.011974468	0.044685208	no	down	mRNA	644.03	625.94	584.99	559.29	448.64	431.38	618.32	479.77	COG0227	J:Translation, ribosomal structure and biogenesis	GO:0006412;GO:0005840;GO:0003735	BP:translation;CC:ribosome;MF:structural constituent of ribosome;
BAB_RS31145	-	EEP domain-containing protein	0.89	-0.16762405	0.012084049	0.045041819	no	down	mRNA	115.08	105.28	114.47	97.26	90.06	93.39	111.61	93.57	COG3568	S:Function unknown	GO:0004519;GO:0004527	MF:endonuclease activity;MF:exonuclease activity;
BAB_RS28835	-	inositol monophosphatase	0.824	-0.278501141	0.012172683	0.045214833	no	down	mRNA	18.63	19.11	18.88	13.96	14.71	15.43	18.8733	14.7	COG0483	E:Amino acid transport and metabolism	GO:0046854;GO:0046854;GO:0008934;GO:0052833;GO:0052832;GO:00	BP:phosphatidylinositol phosphorylation;BP:phosphatidylinositol phosphorylation;MF:inositol monophosphate 1-phosphatase activity;MF:inositol monophosphate 4-phosphatase activity;MF:inositol monophosphate 3-phosphatase activity;MF:inositol monophosphate 1-phosphatase activity;MF:inositol monophosphate 3-phosphatase activity;MF:inositol monophosphate 4-phosphatase activity;
BAB_RS19280	-	M23 family peptidase	0.894	-0.161893367	0.012238285	0.045301393	no	down	mRNA	83.64	76.1	91.39	67.56	73.02	70.25	83.71	70.2766667	COG0739	M:Cell wall/membrane/envelope biogenesis	GO:0016021	CC:integral component of membrane;
BAB_RS30915	-	ABC transporter permease	0.865	-0.209705862	0.012314633	0.045479212	no	down	mRNA	88.76	86.94	89.89	66.83	77.07	73.24	88.53	72.38	COG1173	J:Translation, ribosomal structure and biogenesis	GO:0055085;GO:0055085;GO:0016021;GO:0005886;GO:0016021;GO:00	BP:transmembrane transport;BP:transmembrane transport;CC:integral component of membrane;CC:plasma membrane;CC:integral component of membrane;CC:plasma membrane;
BAB_RS16230	-	lytic transglycosylase	0.876	-0.190784086	0.012436588	0.045824261	no	down	mRNA	280.78	302.76	317.5	258.12	241.73	247.72	300.347	249.19	COG0741	M:Cell wall/membrane/envelope biogenesis	------	------
BAB_RS29910	-	homoserine O-succinyltransferase	0.89	-0.167687106	0.012533333	0.046127833	no	down	mRNA	116.33	116.1	119.72	100.74	99.61	95.26	117.383	98.5366667	COG1897	E:Amino acid transport and metabolism	GO:0019281;GO:0019281;GO:0019281;GO:0005737;GO:0005737;GO:00	BP:L-methionine biosynthetic process from homoserine via O-succinyl-L-homoserine and cystathionine;BP:L-methionine biosynthetic process from homoserine via O-succinyl-L-homoserine and cystathionine;BP:L-methionine biosynthetic process from homoserine via O-succinyl-L-homoserine and cystathionine;CC:cytoplasm;CC:cytoplasm;CC:cytoplasm;MF:homoserine O-acetyltransferase activity;MF:homoserine O-succinyltransferase activity;MF:homoserine O-acetyltransferase activity;MF:homoserine O-succinyltransferase activity;MF:hydrolase activity;MF:homoserine O-acetyltransferase activity;MF:homoserine O-succinyltransferase activity;
BAB_RS19710	-	outer membrane protein assembly factor BamE	0.825	-0.276731851	0.012582143	0.046201628	no	down	mRNA	294.95	237.73	230.79	200.84	184.24	210.81	254.49	198.63	COG2913	M:Cell wall/membrane/envelope biogenesis	GO:0019867	CC:outer membrane;
BAB_RS17755	-	cysteine desulfurase	0.822	-0.283571895	0.012675662	0.046385993	no	down	mRNA	26.89	21.92	22.5	17.8	18.02	19.14	23.77	18.32	COG3748	S:Function unknown	GO:0016021;GO:0046872;GO:0020037;GO:0009055	CC:integral component of membrane;MF:metal ion binding;MF:heme binding;MF:electron carrier activity;
BAB_RS17645	-	pantoate--beta-alanine ligase	0.865	-0.209598325	0.012757932	0.046528078	no	down	mRNA	54.06	56.03	60.31	47.8	46.67	44.98	56.8	46.4833333	COG0414	H:Coenzyme transport and metabolism	GO:0015940;GO:0015940;GO:0005737;GO:0005737;GO:0004592;GO:00	BP:pantothenate biosynthetic process;BP:pantothenate biosynthetic process;CC:cytoplasm;CC:cytoplasm;MF:pantoate-beta-alanine ligase activity;MF:lyase activity;MF:ATP binding;MF:pantoate-beta-alanine ligase activity;MF:ATP binding;
BAB_RS18940	-	ATP-binding protein	0.848	-0.238195574	0.012872393	0.046786199	no	down	mRNA	36.46	40.06	38.53	30.73	29.3	32.25	38.35	30.76	COG0642	T:Signal transduction mechanisms	GO:0016021;GO:0005622;GO:0016021;GO:0005622;GO:0005524;GO:00	CC:integral component of membrane;CC:intracellular;CC:integral component of membrane;CC:intracellular;MF:ATP binding;MF:phosphorelay sensor kinase activity;MF:phosphorelay sensor kinase activity;MF:ATP binding;
BAB_RS25665	-	GMC family oxidoreductase	0.657	-0.605617604	0.013038381	0.047335954	no	down	mRNA	2.57	2.54	3.75	1.81	1.99	1.71	2.95333	1.83666667	COG2303	S:Function unknown	GO:0050660;GO:0016614	MF:flavin adenine dinucleotide binding;MF:oxidoreductase activity, acting on CH-OH group of donors;
BAB_RS29115	-	sn-glycerol 3-phosphate ABC transporter permease	0.837	-0.25740619	0.01316431	0.047578096	no	down	mRNA	60.29	50.41	49.54	41.53	42.61	41.93	53.4133	42.0233333	COG1175	P:Inorganic ion transport and metabolism	GO:0055085;GO:0055085;GO:0008643;GO:0008643;GO:0055085;GO:00	BP:transmembrane transport;BP:transmembrane transport;BP:carbohydrate transport;BP:carbohydrate transport;BP:transmembrane transport;CC:integral component of membrane;CC:plasma membrane;CC:integral component of membrane;CC:plasma membrane;CC:integral component of membrane;CC:plasma membrane;
BAB_RS28700	-	ABC transporter permease	0.772	-0.372823044	0.013270983	0.047748788	no	down	mRNA	19.59	20.44	18.5	12.24	15.49	14.94	19.51	14.2233333	ENOG4111GHG	G:Carbohydrate transport and metabolism	GO:0055085;GO:0055085;GO:0016021;GO:0005886;GO:0016021;GO:00	BP:transmembrane transport;BP:transmembrane transport;CC:integral component of membrane;CC:plasma membrane;CC:integral component of membrane;CC:plasma membrane;
BAB_RS18615	-	glycosyl transferase family 1	0.874	-0.194532082	0.01338403	0.04804792	no	down	mRNA	92.87	80.27	84.02	71.6	68.34	71.61	85.72	70.5166667	COG0438	G:Carbohydrate transport and metabolism	GO:0016740;GO:0016757	MF:transferase activity;MF:transferase activity, transferring glycosyl groups;
BAB_RS23765	-	dihydroxyacetone kinase subunit L	0.879	-0.185348124	0.013514344	0.048353659	no	down	mRNA	219.34	218.54	229.63	171.21	189.05	195.68	222.503	185.313333	COG2376	G:Carbohydrate transport and metabolism	GO:0006071;GO:0006071;GO:0004371;GO:0004371;GO:0016757	BP:glycerol metabolic process;BP:glycerol metabolic process;MF:glycerone kinase activity;MF:glycerone kinase activity;MF:transferase activity, transferring glycosyl groups;
BAB_RS21350	-	GTPase HflX	0.866	-0.206822773	0.013530928	0.048359145	no	down	mRNA	79.59	77.65	80.05	68.11	66.88	58.68	79.0967	64.5566667	COG2262	S:Function unknown	GO:0005737;GO:0005737;GO:0003924;GO:0005525;GO:0005525;GO:00	CC:cytoplasm;CC:cytoplasm;MF:GTPase activity;MF:GTP binding;MF:GTP binding;MF:GTPase activity;
BAB_RS31030	-	peptide deformylase	0.826	-0.275002757	0.013578112	0.04847386	no	down	mRNA	104.32	86.4	83.06	73.31	69.82	71	91.26	71.3766667	COG0242	J:Translation, ribosomal structure and biogenesis	GO:0006412;GO:0006412;GO:0046872;GO:0042586;GO:0046872;GO:00	BP:translation;BP:translation;MF:metal ion binding;MF:peptide deformylase activity;MF:metal ion binding;MF:peptide deformylase activity;
BAB_RS24290	-	DUF465 domain-containing protein	0.893	-0.162996876	0.013617367	0.04850621	no	down	mRNA	209	180.56	207.29	160.27	175.45	165.48	198.95	167.066667	COG5570	S:Function unknown	------	------
BAB_RS26695	-	UTP--glucose-1-phosphate uridylyltransferase	0.907	-0.140332346	0.01368035	0.048515414	no	down	mRNA	129.13	115.94	134.9	108.2	108.54	107.07	126.657	107.936667	COG1210	M:Cell wall/membrane/envelope biogenesis	------	------
BAB_RS27570	-	LacI family transcriptional regulator	0.885	-0.175560718	0.01388526	0.049079584	no	down	mRNA	124.89	117.66	136.8	105.1	110.68	100.91	126.45	105.563333	COG1609	K:Transcription	GO:0006355;GO:0006351;GO:0006355;GO:0006351;GO:0003677;GO:00	BP:regulation of transcription, DNA-templated;BP:transcription, DNA-templated;BP:regulation of transcription, DNA-templated;BP:transcription, DNA-templated;MF:DNA binding;MF:DNA binding;
BAB_RS30280	-	NADPH quinone oxidoreductase	0.599	-0.738933029	0.014087027	0.049628966	no	down	mRNA	88.19	96.27	92.26	34.2	41.37	82.08	92.24	52.55	COG0604	E:Amino acid transport and metabolism	GO:0003960;GO:0003960;GO:0016491;GO:0016491	MF:NADP;MF:NADP;MF:oxidoreductase activity;MF:oxidoreductase activity;
BAB_RS17540	-	2'-deoxycytidine 5'-triphosphate deaminase	0.868	-0.204218612	0.014143228	0.049717934	no	down	mRNA	45.57	43.13	43.79	34.75	37.86	35.74	44.1633	36.1166667	COG0717	F:Nucleotide transport and metabolism	GO:0009394;GO:0008829	BP:2'-deoxyribonucleotide metabolic process;MF:dCTP deaminase activity;
BAB_RS30760	-	Crp/Fnr family transcriptional regulator	0.86	-0.218030848	0.014134918	0.049743144	no	down	mRNA	76.33	69.76	70.79	59.32	61.63	55.01	72.2933	58.6533333	COG0664	T:Signal transduction mechanisms	GO:0006355;GO:0006351;GO:0003677	BP:regulation of transcription, DNA-templated;BP:transcription, DNA-templated;MF:DNA binding;
BAB_RS24765	-	IS6 family transposase IS2020	0.821	-0.283777404	0.014358303	0.050308863	no	down	mRNA	58.11	46.5	47.6	39.35	37.44	40.98	50.7367	39.2566667	COG3316	L:Replication, recombination and repair	GO:0015074	BP:DNA integration;
BAB_RS23510	-	choline ABC transporter substrate-binding protein	0.872	-0.198114802	0.014536329	0.05082179	no	down	mRNA	242.33	212.77	216.95	179.51	175.75	197.31	224.017	184.19	COG2113	E:Amino acid transport and metabolism	GO:0015871;GO:0015871;GO:0043190;GO:0042597;GO:0043190;GO:00	BP:choline transport;BP:choline transport;CC:ATP-binding cassett;CC:periplasmic space;CC:ATP-binding cassett;CC:periplasmic space;MF:choline binding;MF:transmembrane transporter activity;MF:transmembrane transporter activity;MF:choline binding;
BAB_RS24255	-	YafY family transcriptional regulator	0.868	-0.20462048	0.014584216	0.050933789	no	down	mRNA	47.34	45.31	54.17	39.81	40.18	40.41	48.94	40.1333333	COG2378	K:Transcription	GO:0006351;GO:0006351;GO:0006351;GO:0005622;GO:0005622;GO:00	BP:transcription, DNA-templated;BP:transcription, DNA-templated;BP:transcription, DNA-templated;CC:intracellular;CC:intracellular;CC:intracellular;MF:transcription factor activity, sequence-specific DNA binding;MF:DNA binding;MF:transcription factor activity, sequence-specific DNA binding;MF:DNA binding;MF:DNA binding;MF:transcription factor activity, sequence-specific DNA binding;
BAB_RS19655	-	enoyl-CoA hydratase/isomerase family protein	0.88	-0.184118412	0.014684201	0.051060973	no	down	mRNA	70.78	65.02	68.21	53.88	58.32	57.09	68.0033	56.43	COG1024	H:Coenzyme transport and metabolism	GO:0003860;GO:0016853;GO:0004300	MF:3-hydroxyisobutyryl-CoA hydrolase activity;MF:isomerase activity;MF:enoyl-CoA hydratase activity;
BAB_RS32810	-	hypothetical protein	0.79	-0.340950339	0.014710075	0.051095643	no	down	mRNA	39.87	38.93	49.09	31.58	31.94	33.31	42.63	32.2766667	COG2388	S:Function unknown	------	------
BAB_RS23020	-	hypothetical protein	0.695	-0.524513329	0.014683392	0.051113475	no	down	mRNA	15.4	11.8	12.75	9.44	7.51	9.29	13.3167	8.74666667	------	------	------	------
BAB_RS20895	-	dipeptide epimerase	0.841	-0.248980383	0.014772029	0.051200138	no	down	mRNA	73.3	63.09	59.74	52.57	50.89	51.92	65.3767	51.7933333	COG4948	J:Translation, ribosomal structure and biogenesis	GO:0018849;GO:0018850	MF:muconate cycloisomerase activity;MF:chloromuconate cycloisomerase activity;
BAB_RS18725	-	hypothetical protein	0.868	-0.203445993	0.015089974	0.052133427	no	down	mRNA	350.8	346.4	375.98	272.9	308.28	312.8	357.727	297.993333	ENOG4111VRT	S:Function unknown	------	------
BAB_RS16065	-	LysR family transcriptional regulator	0.756	-0.403647568	0.015289794	0.052710416	no	down	mRNA	20.55	18.86	16.04	14.48	11.67	13.34	18.4833	13.1633333	COG0583	K:Transcription	GO:0006351;GO:0006351;GO:0003700;GO:0003677;GO:0003677;GO:00	BP:transcription, DNA-templated;BP:transcription, DNA-templated;MF:transcription factor activity, sequence-specific DNA binding;MF:DNA binding;MF:DNA binding;MF:transcription factor activity, sequence-specific DNA binding;
BAB_RS33035	-	hypothetical protein	0.495	-1.013788886	0.015403416	0.053045204	no	down	mRNA	27.4	32.32	38.79	10.95	21.58	18.08	32.8367	16.87	------	------	------	------
BAB_RS22720	-	DUF4169 domain-containing protein	0.911	-0.13478699	0.01554392	0.053414563	no	down	mRNA	1080.12	1008.48	1083.83	920.35	926.69	911.85	1057.48	919.63	ENOG410XWSV	S:Function unknown	------	------
BAB_RS16000	-	branched-chain amino acid ABC transporter substrate-binding protein	0.863	-0.212510107	0.015684837	0.053783758	no	down	mRNA	69.98	62.59	61.77	51.4	55.21	51.29	64.78	52.6333333	COG0683	E:Amino acid transport and metabolism	GO:0006865	BP:amino acid transport;
BAB_RS19480	-	adenylate kinase	0.82	-0.285804957	0.015845625	0.054161696	no	down	mRNA	134.69	104.6	102.52	91.59	83.93	89.41	113.937	88.31	COG2019	F:Nucleotide transport and metabolism	GO:0016301;GO:0004017;GO:0004017	MF:kinase activity;MF:adenylate kinase activity;MF:adenylate kinase activity;
BAB_RS28150	-	sugar ABC transporter ATP-binding protein	0.905	-0.143700441	0.016104414	0.054871137	no	down	mRNA	149.28	138	153.82	120.06	127.55	128.37	147.033	125.326667	COG1129	S:Function unknown	GO:0005524;GO:0016887;GO:0016301;GO:0016887;GO:0005524	MF:ATP binding;MF:ATPase activity;MF:kinase activity;MF:ATPase activity;MF:ATP binding;
BAB_RS22375	-	serine hydrolase family protein	0.597	-0.743354509	0.016139809	0.054933481	no	down	mRNA	34.2	27.39	22.27	20.31	14.24	13.12	27.9533	15.89	COG3545	S:Function unknown	GO:0016787	MF:hydrolase activity;
BAB_RS22115	-	hypothetical protein	0.879	-0.18665127	0.016296838	0.055409249	no	down	mRNA	264.42	227	248.2	189.33	214.5	211.03	246.54	204.953333	------	------	GO:0016021	CC:integral component of membrane;
BAB_RS29155	-	branched-chain amino acid ABC transporter substrate-binding protein	0.841	-0.250291903	0.0163176	0.055421194	no	down	mRNA	31.91	25.99	31.68	23.4	22.13	25.35	29.86	23.6266667	COG0683	F:Nucleotide transport and metabolism	GO:0006865	BP:amino acid transport;
BAB_RS19310	-	LPS export ABC transporter permease LptF	0.855	-0.225591005	0.016406861	0.055665517	no	down	mRNA	204.85	177.69	177.18	145.67	141.26	164.58	186.573	150.503333	COG0795	S:Function unknown	GO:0055085;GO:0043190	BP:transmembrane transport;CC:ATP-binding cassett;
BAB_RS31820	-	ROK family transcriptional regulator	0.832	-0.264723295	0.016735814	0.056483371	no	down	mRNA	21.95	21.51	26.5	18.83	17.5	18.8	23.32	18.3766667	COG1940	G:Carbohydrate transport and metabolism	GO:0016301	MF:kinase activity;

BAB_RS16460	-	class I SAM-dependent methyltransferase	0.842	-0.248487859	0.017076386	0.057271844	no	down	mRNA	47.91	43.27	43.77	36.07	32.7	38.56	44.9833	35.7766667	COG0500	Q:Secondary metabolites biosynthesis, transport a	GO:0008168	MF:methyltransferase activity;
BAB_RS28335	-	MerR family DNA-binding transcriptional regulator	0.897	-0.157227959	0.017103738	0.057303764	no	down	mRNA	203.19	198.66	214.8	177.22	176.77	169.98	205.55	174.656667	COG0789	S:Function unknown	GO:0006355;GO:0006355;GO:0003677;GO:0003677	BP:regulation of transcription, DNA-templated;BP:regulation of transcription, DNA-templated;MF:DNA binding;MF:DNA binding;
BAB_RS19940	-	DUF1467 domain-containing protein	0.846	-0.241389306	0.017458879	0.058371881	no	down	mRNA	82.3	79.43	92.71	70.22	71.6	62.93	84.8133	68.25	COG5454	S:Function unknown	GO:0016021	CC:integral component of membrane;
BAB_RS16780	-	DUF934 domain-containing protein	0.702	-0.511077434	0.01751056	0.058483815	no	down	mRNA	20.99	14	14.6	12.19	9.89	10.36	16.53	10.8133333	COG3749	S:Function unknown	GO:0016491	MF:oxidoreductase activity;
BAB_RS32540	-	hypothetical protein	0.805	-0.313303041	0.017624742	0.058743046	no	down	mRNA	24.17	18.08	23.18	17.33	14.8	17.35	21.81	16.4933333	------	------	------	------
BAB_RS30050	-	transcriptional regulator	0.679	-0.557793934	0.017738329	0.058938212	no	down	mRNA	27.76	27.95	34.82	19.25	22.48	18.63	30.1767	20.12	COG0640	S:Function unknown	GO:0003700	MF:transcription factor activity, sequence-specific DNA binding;
BAB_RS32535	-	hypothetical protein	0.816	-0.294052626	0.017788106	0.059042546	no	down	mRNA	18.02	14.93	19.14	11.72	15.21	12.84	17.3633	13.2566667	------	------	------	------
BAB_RS32410	-	amino acid ABC transporter permease	0.636	-0.652163174	0.018688278	0.061648291	no	down	mRNA	4.1	4.15	5.01	3.15	3.09	1.67	4.42	2.63666667	COG4597	E:Amino acid transport and metabolism	GO:0071705;GO:0043190;GO:0022857	BP:nitrogen compound transport;CC:ATP-binding cassett;MF:transmembrane transporter activity;
BAB_RS21885	-	50S ribosomal protein L16	0.88	-0.184191544	0.018732699	0.061668199	no	down	mRNA	1280.31	1089.22	1170.65	968.65	1086.2	930.46	1180.06	995.103333	COG0197	J:Translation, ribosomal structure and biogenesis	GO:0006412;GO:0006412;GO:0005840;GO:0005840;GO:0003735;GO:00	BP:translation;BP:translation;CC:ribosome;CC:ribosome;MF:structural constituent of ribosome;MF:rRNA binding;MF:tRNA binding;MF:rRNA binding;MF:structural constituent of ribosome;MF:tRNA binding;
BAB_RS19230	-	hypothetical protein	0.874	-0.194465816	0.018761928	0.061701203	no	down	mRNA	31.26	32.06	32.96	26.05	27.01	26.36	32.0933	26.4733333	ENOG4111Q0U	S:Function unknown	------	------
BAB_RS24720	-	gamma-glutamyl-phosphate reductase	0.857	-0.222446957	0.018804166	0.061776876	no	down	mRNA	40.68	40.47	42.52	31.44	37	31.42	41.2233	33.2866667	COG0014	E:Amino acid transport and metabolism	GO:0055129;GO:0055129;GO:0005737;GO:0005737;GO:0004350;GO:00	BP:L-proline biosynthetic process;BP:L-proline biosynthetic process;CC:cytoplasm;CC:cytoplasm;MF:glutamate-5-semialdehyde dehydrogenase activity;MF:NADP binding;MF:transferase activity;MF:NADP binding;MF:glutamate-5-semialdehyde dehydrogenase activity;
BAB_RS21840	-	50S ribosomal protein L18	0.894	-0.161941135	0.019384425	0.063230618	no	down	mRNA	1419.28	1331.37	1506.38	1232.19	1188.99	1234.92	1419.01	1218.7	COG0256	J:Translation, ribosomal structure and biogenesis	GO:0006412;GO:0005840;GO:0003735;GO:0019843	BP:translation;CC:ribosome;MF:structural constituent of ribosome;MF:rRNA binding;
BAB_RS26500	-	ABC transporter ATP-binding protein	0.67	-0.577086074	0.019433294	0.063325735	no	down	mRNA	10.66	11.24	8.99	4.66	7.45	7.37	10.2967	6.49333333	COG0410	S:Function unknown	GO:0016887;GO:0005524	MF:ATPase activity;MF:ATP binding;
BAB_RS25205	-	hypothetical protein	0.818	-0.289025305	0.019484957	0.063429753	no	down	mRNA	64.49	50.56	55.49	44.58	41.79	45.97	56.8467	44.1133333	------	------	------	------
BAB_RS26680	-	type IV secretion system protein virB2	0.776	-0.366314606	0.019519292	0.063477212	no	down	mRNA	36.1	27.37	27.1	20.76	21.53	23.72	30.19	22.0033333	ENOG410XUMD	U:Intracellular trafficking, secretion, and vesicular t	GO:0009405;GO:0016021;GO:0005886;GO:0016021	BP:pathogenesis;CC:integral component of membrane;CC:plasma membrane;CC:integral component of membrane;
BAB_RS33360	-	sugar ABC transporter substrate-binding protein	0.825	-0.277766286	0.019903229	0.064464792	no	down	mRNA	21.83	25.72	28.62	18.79	20.1	20.74	25.39	19.8766667	COG1653	G:Carbohydrate transport and metabolism	GO:0055085;GO:0008643;GO:0055085	BP:transmembrane transport;BP:carbohydrate transport;BP:transmembrane transport;
BAB_RS30030	-	AI-2E family transporter	0.888	-0.170959098	0.020097688	0.064898362	no	down	mRNA	165.11	147.96	152.42	123.13	135.54	131.28	155.163	129.983333	COG0628	V:Defense mechanisms	GO:0016021	CC:integral component of membrane;
BAB_RS17060	-	L-rhamnose mutarotase	0.765	-0.385794462	0.020086458	0.064927355	no	down	mRNA	87.1	87.11	106.98	69.5	70.76	72.28	93.73	70.8466667	COG3254	S:Function unknown	GO:0005737;GO:0005737;GO:0016857;GO:0016857	CC:cytoplasm;CC:cytoplasm;MF:racemase and epimerase activity, acting on carbohydrates and derivatives;MF:racemase and epimerase activity, acting on carbohydrates and derivatives;
BAB_RS20980	-	histidine phosphatase family protein	0.868	-0.205023104	0.020186511	0.065054422	no	down	mRNA	51.35	44.81	55.71	44.29	38.8	40.92	50.6233	41.3366667	COG2062	T:Signal transduction mechanisms	------	------
BAB_RS26295	-	transcriptional repressor	0.852	-0.231070544	0.020173408	0.06507747	no	down	mRNA	650.74	501.88	512.36	474.38	436.1	436.51	554.993	448.996667	COG0735	J:Translation, ribosomal structure and biogenesis	GO:0006351;GO:0005737;GO:0003700;GO:0046872;GO:0003677	BP:transcription, DNA-templated;CC:cytoplasm;MF:transcription factor activity, sequence-specific DNA binding;MF:metal ion binding;MF:DNA binding;
BAB_RS27780	-	2-dehydro-3-deoxygalactonokinase	0.873	-0.195369469	0.020459922	0.065737728	no	down	mRNA	298.1	296.05	356.51	246.55	284.21	253.08	316.887	261.28	COG3734	G:Carbohydrate transport and metabolism	GO:0034194;GO:0008671	BP:D-galactonate catabolic process;MF:2-dehydro-3-deoxygalactonokinase activity;
BAB_RS19270	-	citramalate synthase	0.894	-0.161122312	0.020772211	0.066409068	no	down	mRNA	96.97	93.4	95.28	83.59	80.3	76.78	95.2167	80.2233333	COG0119	J:Translation, ribosomal structure and biogenesis	GO:0009098;GO:0009098;GO:0003852;GO:0016829;GO:0003852	BP:leucine biosynthetic process;BP:leucine biosynthetic process;MF:2-isopropylmalate synthase activity;MF:lyase activity;MF:2-isopropylmalate synthase activity;
BAB_RS24295	-	DUF465 domain-containing protein	0.769	-0.379405504	0.020807666	0.066456294	no	down	mRNA	192.28	228.53	203.97	175.05	143.9	161.75	208.26	160.233333	COG5481	S:Function unknown	------	------
BAB_RS22755	-	ATP-dependent DNA helicase	0.897	-0.156931518	0.020770271	0.066469006	no	down	mRNA	43.59	44.73	46.38	38.4	38.42	36.95	44.9	37.9233333	COG0210	L:Replication, recombination and repair	GO:0004003;GO:0005524;GO:0003677;GO:0005524;GO:0004003;GO:00	MF:ATP-dependent DNA helicase activity;MF:ATP binding;MF:DNA binding;MF:ATP binding;MF:ATP-dependent DNA helicase activity;MF:DNA binding;
BAB_RS25310	-	cysteine synthase A	0.801	-0.320978479	0.020867705	0.066515808	no	down	mRNA	470.16	463.23	460.06	329.07	292.45	430.08	464.483	350.533333	COG0031	G:Carbohydrate transport and metabolism	GO:0006535;GO:0006535;GO:0006535;GO:0004124;GO:0047189;GO:00	BP:cysteine biosynthetic process from serine;BP:cysteine biosynthetic process from serine;BP:cysteine biosynthetic process from serine;MF:cysteine synthase activity;MF:2,3-diaminopropionate N-oxalyltransferase activity;MF:cysteine synthase activity;MF:cysteine synthase activity;
BAB_RS33345	-	hypothetical protein	0.771	-0.376036743	0.020852328	0.066532799	no	down	mRNA	317.49	241.49	214.96	183.75	186.88	217.8	257.98	196.143333	------	------	------	------
BAB_RS24390	-	CarD family transcriptional regulator	0.885	-0.175505508	0.021010796	0.066839294	no	down	mRNA	1927.47	1666.03	1708.84	1545.62	1520.83	1387.84	1767.45	1484.76333	COG1329	K:Transcription	------	------
BAB_RS19635	-	DNA topoisomerase IV subunit A	0.904	-0.145818906	0.021089249	0.067022509	no	down	mRNA	80.65	70.45	78.12	62.57	67.14	64.95	76.4067	64.8866667	COG0188	L:Replication, recombination and repair	GO:0007059;GO:0006265;GO:0006265;GO:0007059;GO:0005694;GO:00	BP:chromosome segregation;BP:DNA topological change;BP:DNA topological change;BP:chromosome segregation;CC:chromosome;CC:extrinsic component of plasma membrane;CC:extrinsic component of plasma membrane;CC:chromosome;MF:ATP binding;MF:DNA binding;MF:DNA topoisomerase type I;MF:DNA binding;MF:DNA topoisomerase type I;MF:ATP binding;
BAB_RS31855	-	ABC transporter ATP-binding protein	0.824	-0.278657259	0.021280846	0.067497884	no	down	mRNA	39.02	35.91	39.66	33.29	29.45	26.59	38.1967	29.7766667	COG0411	E:Amino acid transport and metabolism	GO:0016887;GO:0005524;GO:0005524;GO:0016887	MF:ATPase activity;MF:ATP binding;MF:ATP binding;MF:ATPase activity;
BAB_RS20585	-	molybdenum cofactor guanylyltransferase	0.892	-0.165361178	0.021389166	0.067641134	no	down	mRNA	111.85	101.17	105.39	87.9	90.07	90.44	106.137	89.47	COG0746	H:Coenzyme transport and metabolism	GO:0006777;GO:0005737;GO:0061603;GO:0046872;GO:0005525	BP:Mo-molybdopterin cofactor biosynthetic process;CC:cytoplasm;MF:molybdenum cofactor guanylyltransferase activity;MF:metal ion binding;MF:GTP binding;
BAB_RS33520	-	type II toxin-antitoxin system PemK/MazF family toxin	0.757	-0.401752079	0.021614421	0.068219189	no	down	mRNA	18.81	19.44	17.1	14.82	12.51	12.44	18.45	13.2566667	COG2337	T:Signal transduction mechanisms	GO:0003677	MF:DNA binding;
BAB_RS32510	-	hypothetical protein	0.834	-0.261276376	0.021772787	0.068316373	no	down	mRNA	18.53	15.03	15.34	13.42	12.54	12.23	16.3	12.73	------	------	------	------
BAB_RS24930	-	uroporphyrinogen III methyltransferase	0.782	-0.354647017	0.021667385	0.068319244	no	down	mRNA	13.93	14.53	14.34	9.58	11.19	10.94	14.2667	10.57	COG1587	H:Coenzyme transport and metabolism	GO:0033014;GO:0033014;GO:0004852;GO:0004852	BP:tetrapyrrole biosynthetic process;BP:tetrapyrrole biosynthetic process;MF:uroporphyrinogen-III synthase activity;MF:uroporphyrinogen-III synthase activity;
BAB_RS23390	-	N-acetyltransferase	0.882	-0.181251976	0.021764967	0.068358593	no	down	mRNA	56.04	50.5	52.9	45.2	45.68	41.38	53.1467	44.0866667	COG3153	S:Function unknown	GO:0016740;GO:0016746	MF:transferase activity;MF:transferase activity, transferring acyl groups;
BAB_RS23200	-	peptide-binding protein	0.862	-0.214160556	0.02172339	0.068428677	no	down	mRNA	49.66	46.01	49.1	39.71	41.24	36.93	48.2567	39.2933333	COG4991	I:Lipid transport and metabolism	------	------
BAB_RS30635	-	U32 family peptidase	0.855	-0.225173228	0.02197277	0.068876595	no	down	mRNA	316.05	322.42	293.34	222.63	267.18	264.97	310.603	251.593333	COG0826	O:Posttranslational modification, protein turnover,	GO:0008233	MF:peptidase activity;
BAB_RS26805	-	nickel/cobalt exporter	0.876	-0.19089796	0.022288936	0.069731599	no	down	mRNA	78.65	79.17	80.26	59.85	68.3	68.91	79.36	65.6866667	COG2215	P:Inorganic ion transport and metabolism	GO:0016021;GO:0005886;GO:0016021;GO:0005886;GO:0046872;GO:00	CC:integral component of membrane;CC:plasma membrane;CC:integral component of membrane;CC:plasma membrane;MF:metal ion binding;MF:nickel cation transmembrane transporter activity;MF:nickel cation transmembrane transporter activity;MF:metal ion binding;
BAB_RS17175	-	hypothetical protein	0.849	-0.235721731	0.022358131	0.069880034	no	down	mRNA	125.05	130.9	130.75	101.75	112.71	101.81	128.9	105.423333	------	------	GO:0016021	CC:integral component of membrane;
BAB_RS31150	-	cardiolipin synthase	0.887	-0.172519805	0.022478881	0.070121015	no	down	mRNA	35.67	29.72	35.67	27.31	28.03	28.83	33.6867	28.0566667	COG1502	I:Lipid transport and metabolism	GO:0032049;GO:0032049;GO:0016021;GO:0005886;GO:0016021;GO:00	BP:cardiolipin biosynthetic process;BP:cardiolipin biosynthetic process;CC:integral component of membrane;CC:plasma membrane;CC:integral component of membrane;CC:plasma membrane;MF:cardiolipin synthase activity;MF:cardiolipin synthase activity;
BAB_RS28375	-	GntR family transcriptional regulator	0.86	-0.217878611	0.022459052	0.070127245	no	down	mRNA	36.06	33.69	37.15	26.8	28.66	31.38	35.6333	28.9466667	COG1802	G:Carbohydrate transport and metabolism	GO:0006351;GO:0006351;GO:0003700;GO:0003677;GO:0003677;GO:00	BP:transcription, DNA-templated;BP:transcription, DNA-templated;MF:transcription factor activity, sequence-specific DNA binding;MF:DNA binding;MF:DNA binding;MF:transcription factor activity, sequence-specific DNA binding;
BAB_RS24440	-	3-hydroxybutyrate dehydrogenase	0.882	-0.181148654	0.02270039	0.070606345	no	down	mRNA	63.61	60.35	68.5	48.65	53.82	57.43	64.1533	53.3	ENOG410XPTP	C:Energy production and conversion	GO:0003858;GO:0008233;GO:0003858	MF:3-hydroxybutyrate dehydrogenase activity;MF:peptidase activity;MF:3-hydroxybutyrate dehydrogenase activity;
BAB_RS26800	-	hypothetical protein	0.759	-0.39690244	0.022693331	0.070652783	no	down	mRNA	26.04	23.77	19.5	19.19	14.76	15.58	23.1033	16.51	ENOG410ZAA4	S:Function unknown	GO:0016021	CC:integral component of membrane;
BAB_RS33195	-	prenyltransferase	0.822	-0.283468433	0.022755425	0.070709072	no	down	mRNA	15.18	12.1	13.56	10.85	9.74	10.9	13.6133	10.4966667	COG0382	S:Function unknown	GO:0016021;GO:0016765	CC:integral component of membrane;MF:transferase activity, transferring alkyl or ary;
BAB_RS29120	-	sn-glycerol-3-phosphate-binding periplasmic protein UgpB	0.874	-0.194097161	0.022818826	0.070769197	no	down	mRNA	168.74	148.19	145.71	119.68	129.77	131.82	154.213	127.09	COG1653	P:Inorganic ion transport and metabolism	GO:0008643;GO:0008643;GO:0042597;GO:0042597	BP:carbohydrate transport;BP:carbohydrate transport;CC:periplasmic space;CC:periplasmic space;
BAB_RS32730	-	D-lyxose/D-mannose family sugar isomerase	0.821	-0.284283889	0.022798176	0.07077347	no	down	mRNA	64.61	52.93	62.52	45.19	44.49	51.4	60.02	47.0266667	COG3822	S:Function unknown	------	------
BAB_RS29105	-	ABC transporter ATP-binding protein	0.869	-0.202075009	0.022867325	0.070851221	no	down	mRNA	75.83	73.47	77.23	55.38	67.21	63.26	75.51	61.95	COG3839;COG3842	P:Inorganic ion transport and metabolism;	GO:0001407;GO:0008643;GO:0001407;GO:0008643;GO:0008643;GO:00	BP:glycerophosphodiester transport;BP:carbohydrate transport;BP:glycerophosphodiester transport;BP:carbohydrate transport;BP:carbohydrate transport;BP:glycerophosphodiester transport;CC:ATP-binding cassett;CC:ATP-binding cassett;CC:ATP-binding cassett;MF:glycerol-3-phosphate-transporting ATPase activity;MF:ATP binding;MF:glycerol-3-phosphate-transporting ATPase activity;MF:ATP binding;MF:ATP binding;MF:glycerol-3-phosphate-transporting ATPase activity;
BAB_RS30120	-	glycerol kinase	0.363	-1.463651791	0.02317989	0.071612487	no	down	mRNA	33.17	71.72	94.47	37.79	31.07	14.1	66.4533	27.6533333	COG0554	C:Energy production and conversion	GO:0019563;GO:0006072;GO:0005524;GO:0004370	BP:glycerol catabolic process;BP:glycerol-3-phosphate metabolic process;MF:ATP binding;MF:glycerol kinase activity;
BAB_RS26025	-	translation initiation factor IF-3	0.841	-0.249997109	0.023370945	0.071926097	no	down	mRNA	1271.85	1026.81	992.62	955.56	824.54	838.2	1097.09	872.766667	COG0290	J:Translation, ribosomal structure and biogenesis	GO:0005737;GO:0003743	CC:cytoplasm;MF:translation initiation factor activity;
BAB_RS27680	-	MetQ/NlpA family ABC transporter substrate-binding protein	0.888	-0.171630882	0.023332937	0.071946955	no	down	mRNA	308.33	298.59	289.19	252.2	236.23	264.46	298.703	250.963333	COG1464	P:Inorganic ion transport and metabolism	------	------
BAB_RS27655	-	30S ribosomal protein S21	0.828	-0.272316798	0.023706781	0.072889844	no	down	mRNA	2918.55	2319.7	2318.26	2319.14	1792.46	1870.8	2518.84	1994.13333	COG0828	J:Translation, ribosomal structure and biogenesis	GO:0006412;GO:0005840;GO:0003735	BP:translation;CC:ribosome;MF:structural constituent of ribosome;
BAB_RS19305	-	LPS export ABC transporter permease LptG	0.901	-0.151063938	0.023912295	0.073241375	no	down	mRNA	224.4	210.02	211.67	183.28	177.1	189	215.363	183.126667	COG0795	S:Function unknown	GO:0055085;GO:0043190	BP:transmembrane transport;CC:ATP-binding cassett;
BAB_RS18685	-	asparaginase	0.733	-0.448526227	0.023936523	0.073245762	no	down	mRNA	9.85	11.79	10.16	8.61	7.52	5.93	10.6	7.35333333	COG4448	E:Amino acid transport and metabolism	------	------
BAB_RS25040	-	hypothetical protein	0.759	-0.39709369	0.023910692	0.073306348	no	down	mRNA	12.42	12.71	11.83	8.82	9.66	7.99	12.32	8.82333333	COG0730;COG0730	F:Nucleotide transport and metabolism;H:Coenzym	GO:0016021	CC:integral component of membrane;
BAB_RS18460	-	DUF2793 domain-containing protein	0.851	-0.232916926	0.02399764	0.073362909	no	down	mRNA	26.97	27.13	28.85	20.15	23.76	22.75	27.65	22.22	ENOG410XPKR	S:Function unknown	------	------
BAB_RS16385	-	DsbE family thiol:disulfide interchange protein	0.882	-0.181037534	0.024448866	0.074388453	no	down	mRNA	108.52	104.71	113.25	85.59	96.68	91.12	108.827	91.13	COG0526	O:Posttranslational modification, protein turnover,	GO:0045454;GO:0017004;GO:0045454;GO:0017004;GO:0045454;GO:00	BP:cell redox homeostasis;BP:cytochrome complex assembly;BP:cell redox homeostasis;BP:cytochrome complex assembly;BP:cell redox homeostasis;BP:cytochrome complex assembly;CC:outer membrane-bounded periplasmic space;CC:outer membrane-bounded periplasmic space;CC:outer membrane-bounded periplasmic space;MF:disulfide oxidoreductase activity;MF:disulfide oxidoreductase activity;MF:disulfide oxidoreductase activity;
BAB_RS32335	-	hypothetical protein	0.771	-0.375505906	0.024524606	0.074548307	no	down	mRNA	71.67	54.02	56.64	42.44	37.65	53.3	60.7767	44.4633333	------	------	------	------
BAB_RS17710	-	C4-dicarboxylate ABC transporter	0.712	-0.489368176	0.024671658	0.074712571	no	down	mRNA	4.96	6.53	4.76	3.29	4.18	3.45	5.41667	3.64	COG4664	Q:Secondary metabolites biosynthesis, transport a	GO:0016021	CC:integral component of membrane;
BAB_RS26810	-	DUF1007 domain-containing protein	0.866	-0.208241557	0.024951022	0.0754164	no	down	mRNA	94.22	84.82	90.32	66.55	73.31	80.9	89.7867	73.5866667	COG3683	S:Function unknown	------	------
BAB_RS28355	-	RND transporter	0.509	-0.97556786	0.025121773	0.075789912	no	down	mRNA	19.8	27.12	35.03	18.28	14.89	10.72	27.3167	14.63	ENOG410XSF7	V:Defense mechanisms	------	------
BAB_RS32145	-	dihydropyrimidinase	0.734	-0.44577084	0.025207645	0.075977639	no	down	mRNA	5.45	5.05	5.12	4.21	3.74	2.76	5.20667	3.57	COG0044	F:Nucleotide transport and metabolism	GO:0005737;GO:0016810	CC:cytoplasm;MF:hydrolase activity, acting on carbon-nitroge;
BAB_RS32940	-	hypothetical protein	0.813	-0.298397497	0.02538009	0.076069243	no	down	mRNA	115.97	99.05	100.37	91.02	81.88	73.85	105.13	82.25	ENOG410Z7I0	S:Function unknown	------	------
BAB_RS31520	-	flagellar protein FlgJ	0.731	-0.452533854	0.025452076	0.076072111	no	down	mRNA	17.25	10.73	14.06	8.6	9.67	10.41	14.0133	9.56	ENOG41121KP	S:Function unknown	------	------
BAB_RS17910	-	ribonuclease HII	0.899	-0.154006863	0.025354427	0.076134368	no	down	mRNA	143.89	138.63	141	117.99	123.05	119.81	141.173	120.283333	COG0164	L:Replication, recombination and repair	GO:0006401;GO:0005737;GO:0030145;GO:0004523;GO:0003723;GO:00	BP:RNA catabolic process;CC:cytoplasm;MF:manganese ion binding;MF:RNA-DNA hybrid ribonuclease activity;MF:RNA binding;MF:metal ion binding;
BAB_RS28535	-	ABC transporter ATP-binding protein	0.757	-0.401048173	0.025528719	0.076230274	no	down	mRNA	9.01	9.54	10.78	7.5	6.73	6.96	9.77667	7.06333333	COG0444	S:Function unknown	GO:0005524;GO:0016887;GO:0005524;GO:0016887;GO:0016301;GO:00	MF:ATP binding;MF:ATPase activity;MF:ATP binding;MF:ATPase activity;MF:kinase activity;MF:ATPase activity;MF:ATP binding;MF:kinase activity;MF:ATPase activity;MF:ATP binding;
BAB_RS18355	-	hypothetical protein	0.893	-0.163609223	0.025657281	0.076472026	no	down	mRNA	137.97	126.34	128.42	114.23	106.53	110.49	130.91	110.416667	ENOG410YSGE;ENOG41124YM	S:Function unknown;	------	------
BAB_RS29875	-	hypothetical protein	0.829	-0.271327875	0.025706896	0.076548893	no	down	mRNA	124.68	95.39	96.37	88.91	76.88	81.91	105.48	82.5666667	------	------	------	------
BAB_RS30400	-	LysR family transcriptional regulator	0.891	-0.166145661	0.025821407	0.076747623	no	down	mRNA	168.99	154.35	165.78	129.55	149.94	130.6	163.04	136.696667	ENOG410Z409	K:Transcription	GO:0006351;GO:0003700;GO:0003677	BP:transcription, DNA-templated;MF:transcription factor activity, sequence-specific DNA binding;MF:DNA binding;
BAB_RS23590	-	N-acetyltransferase	0.876	-0.191282957	0.02618415	0.077325069	no	down	mRNA	261.18	264.53	238.83	197.68	219.67	223.19	254.847	213.513333	COG2388	S:Function unknown	GO:0016740	MF:transferase activity;
BAB_RS21570	-	isoprenyl transferase	0.887	-0.17227864	0.026176306	0.077373019	no	down	mRNA	94.03	82.83	92.61	71.12	78.79	75.98	89.8233	75.2966667	COG0020	I:Lipid transport and metabolism	GO:0000287;GO:0004659;GO:0016765;GO:0046872;GO:0004659;GO:00	MF:magnesium ion binding;MF:prenyltransferase activity;MF:transferase activity, transferring alkyl or ary;MF:metal ion binding;MF:prenyltransferase activity;MF:magnesium ion binding;MF:transferase activity, transferring alkyl or ary;MF:metal ion binding;MF:transferase activity, transferring acyl groups;MF:prenyltransferase activity;MF:magnesium ion binding;MF:magnesium ion binding;MF:transferase activity, transferring acyl groups;MF:prenyltransferase activity;
BAB_RS17485	-	amino acid ABC transporter	0.891	-0.167014318	0.026144748	0.07742219	no	down	mRNA	51.31	47.84	52.3	42.36	43.63	41.25	50.4833	42.4133333	COG0834	E:Amino acid transport and metabolism	------	------
BAB_RS15975	-	microcin C ABC transporter permease YeiB	0.906	-0.143161398	0.026131217	0.077453507	no	down	mRNA	111.82	102.14	108.03	90.82	91.26	92.82	107.33	91.6333333	COG4174	P:Inorganic ion transport and metabolism	GO:0055085;GO:0016021;GO:0005886	BP:transmembrane transport;CC:integral component of membrane;CC:plasma membrane;
BAB_RS29110	-	sn-glycerol 3-phosphate ABC transporter permease	0.862	-0.214418739	0.026338256	0.077637446	no	down	mRNA	66.78	57.9	61	45.16	53.17	52.52	61.8933	50.2833333	COG0395	P:Inorganic ion transport and metabolism	GO:0015794;GO:0001407;GO:0008643;GO:0015794;GO:0001407;GO:00	BP:glycerol-3-phosphate transport;BP:glycerophosphodiester transport;BP:carbohydrate transport;BP:glycerol-3-phosphate transport;BP:glycerophosphodiester transport;CC:ATP-binding cassett;CC:integral component of plasma membrane;CC:ATP-binding cassett;CC:integral component of plasma membrane;
BAB_RS17075	-	NAD(P)-dependent oxidoreductase	0.905	-0.143286697	0.026419815	0.077806475	no	down	mRNA	171.4	160.27	167.05	144.41	141.71	141.28	166.24	142.466667	ENOG410XSIT	S:Function unknown	------	------
BAB_RS21100	-	X-Pro dipeptidase	0.807	-0.309105643	0.026662416	0.078234102	no	down	mRNA	12.3	11.52	16.53	10.29	10.85	9.55	13.45	10.23	COG0006	E:Amino acid transport and metabolism	GO:0016787	MF:hydrolase activity;
BAB_RS18620	-	sugar ABC transporter ATP-binding protein	0.834	-0.262210443	0.026660821	0.07830093	no	down	mRNA	23.72	19.38	22.33	19.15	15.18	16.94	21.81	17.09	COG1129	P:Inorganic ion transport and metabolism	GO:0005524;GO:0016887;GO:0016887;GO:0005524	MF:ATP binding;MF:ATPase activity;MF:ATPase activity;MF:ATP binding;
BAB_RS19205	-	DNA repair protein RecO	0.851	-0.233135183	0.026843663	0.07869406	no	down	mRNA	37.17	39.21	41.77	29.14	31.74	34.53	39.3833	31.8033333	COG1381	L:Replication, recombination and repair	GO:0006310;GO:0006281	BP:DNA recombination;BP:DNA repair;
BAB_RS26505	-	glyoxalase/bleomycin resistance/extradiol dioxygenase family protein	0.593	-0.752906227	0.026979497	0.079020168	no	down	mRNA	11.25	7.37	9.2	4.23	7.16	3.73	9.27333	5.04	COG0346;ENOG410XUYC	S:Function unknown;	GO:0016829;GO:0051213;GO:0051213;GO:0016829;GO:0051213;GO:00	MF:lyase activity;MF:dioxygenase activity;MF:dioxygenase activity;MF:lyase activity;MF:dioxygenase activity;MF:lactoylglutathione lyase activity;
BAB_RS18980	-	sensor histidine kinase	0.892	-0.165455127	0.027056446	0.079173369	no	down	mRNA	43.71	38.72	45.47	34.48	37.22	35.58	42.6333	35.76	COG0642	T:Signal transduction mechanisms	GO:0016021;GO:0005622;GO:0005524;GO:0000155	CC:integral component of membrane;CC:intracellular;MF:ATP binding;MF:phosphorelay sensor kinase activity;
BAB_RS16295	-	DUF1232 domain-containing protein	0.861	-0.215571354	0.027103924	0.079240134	no	down	mRNA	197.63	182.15	228.27	169.07	178.53	147.09	202.683	164.896667	COG3339	S:Function unknown	------	------
BAB_RS26730	-	homospermidine synthase	0.902	-0.148855801	0.027262326	0.079630776	no	down	mRNA	386.25	370.35	378.3	301.67	326.1	337.52	378.3	321.763333	COG5310	Q:Secondary metabolites biosynthesis, transport a	GO:0047296;GO:0016491;GO:0016491	MF:homospermidine synthase activity;MF:oxidoreductase activity;MF:oxidoreductase activity;
BAB_RS27945	-	LysR family transcriptional regulator	0.897	-0.156643388	0.027530998	0.08005167	no	down	mRNA	118.08	104.12	114.97	89.66	96.49	99.12	112.39	95.09	ENOG410XS6G	S:Function unknown	GO:0006351;GO:0016021;GO:0003700;GO:0003677	BP:transcription, DNA-templated;CC:integral component of membrane;MF:transcription factor activity, sequence-specific DNA binding;MF:DNA binding;
BAB_RS28200	-	hypothetical protein	0.735	-0.443968287	0.027527611	0.080114324	no	down	mRNA	9.32	9.14	7.93	5.96	5.38	7.02	8.79667	6.12	ENOG4111AFG	S:Function unknown	GO:0016021	CC:integral component of membrane;
BAB_RS28060	-	CoA ester lyase	0.897	-0.156855322	0.027524466	0.080177797	no	down	mRNA	99.62	97.47	102.12	79.8	88.62	85.09	99.7367	84.5033333	COG2301	E:Amino acid transport and metabolism	GO:0008815;GO:0046872;GO:0016829;GO:0046872	MF:citrat;MF:metal ion binding;MF:lyase activity;MF:metal ion binding;
BAB_RS25820	-	transglycosylase	0.882	-0.181348178	0.027676302	0.080256281	no	down	mRNA	36.32	35.71	38.27	31.31	29.06	31.55	36.7667	30.64	COG2821	M:Cell wall/membrane/envelope biogenesis	GO:0009254;GO:0009254;GO:0019867;GO:0019867;GO:0004553;GO:00	BP:peptidoglycan turnover;BP:peptidoglycan turnover;CC:outer membrane;CC:outer membrane;MF:hydrolase activity, hydrolyzing O-glycosyl compounds;MF:hydrolase activity, hydrolyzing O-glycosyl compounds;
BAB_RS31830	-	AraC family transcriptional regulator	0.812	-0.300972784	0.027660528	0.080282995	no	down	mRNA	12.97	12.94	11.16	8.75	10.32	9.19	12.3567	9.42	COG2207	G:Carbohydrate transport and metabolism	GO:0006351;GO:0003700;GO:0043565	BP:transcription, DNA-templated;MF:transcription factor activity, sequence-specific DNA binding;MF:sequence-specific DNA binding;
BAB_RS32860	-	hypothetical protein	0.623	-0.682059239	0.027803624	0.080335471	no	down	mRNA	354.91	265.97	346.6	224.45	166.04	246.45	322.493	212.313333	------	------	------	------
BAB_RS17450	-	HAD family hydrolase	0.908	-0.139656037	0.027868777	0.080451376	no	down	mRNA	69.13	66.72	70.5	59.39	60.34	56.59	68.7833	58.7733333	COG0546	S:Function unknown	GO:0008152;GO:0008152;GO:0016787;GO:0016787;GO:0008233;GO:00	BP:metabolic process;BP:metabolic process;MF:hydrolase activity;MF:hydrolase activity;MF:peptidase activity;MF:phosphoglycolate phosphatase activity;
BAB_RS16165	-	hypothetical protein	0.801	-0.319973109	0.028055411	0.080917447	no	down	mRNA	25.31	30.7	31.35	22.7	23.88	20.16	29.12	22.2466667	ENOG410Y48T	S:Function unknown	------	------
BAB_RS28305	-	hypothetical protein	0.908	-0.13999668	0.028290005	0.081447837	no	down	mRNA	226.41	202.09	212.4	177.06	183.06	188.35	213.633	182.823333	ENOG410YM9C;ENOG41127GP	S:Function unknown;	GO:0016021	CC:integral component of membrane;
BAB_RS25985	-	N-(5'-phosphoribosyl)anthranilate isomerase	0.883	-0.17887968	0.028280138	0.081492452	no	down	mRNA	125.49	118.85	138.85	108.73	112.02	99.99	127.73	106.913333	COG0135	E:Amino acid transport and metabolism	GO:0000162;GO:0004640	BP:tryptophan biosynthetic process;MF:phosphoribosylanthranilate isomerase activity;
BAB_RS18535	-	IS5/IS1182 family transposase	0.914	-0.129135	0.028478414	0.081843601	no	down	mRNA	45.22	42.84	51.59	39.51	40.66	39.66	46.55	39.9433333	ENOG4111SY5	L:Replication, recombination and repair	------	------
BAB_RS18405	-	chromosome segregation protein SMC	0.877	-0.18907649	0.028573914	0.081898293	no	down	mRNA	25.07	28.25	30.29	21.84	23.64	23.65	27.87	23.0433333	COG1196	D:Cell cycle control, cell division, chromosome par	GO:0030261;GO:0006260;GO:0007062;GO:0030261;GO:0006260;GO:00	BP:chromosome condensation;BP:DNA replication;BP:sister chromatid cohesion;BP:chromosome condensation;BP:DNA replication;BP:sister chromatid cohesion;CC:chromosome;CC:cytoplasm;CC:cytoplasm;CC:chromosome;MF:ATP binding;MF:DNA binding;MF:DNA binding;MF:ATP binding;
BAB_RS17410	-	Zn-dependent hydrolase	0.89	-0.168433229	0.028534528	0.08193158	no	down	mRNA	82.47	77.79	90.6	64.81	70.22	75.34	83.62	70.1233333	COG0624	F:Nucleotide transport and metabolism	GO:0008152;GO:0008152;GO:0008152;GO:0016813;GO:0016746;GO:00	BP:metabolic process;BP:metabolic process;BP:metabolic process;MF:hydrolase activity, acting on carbon-nitroge;MF:transferase activity, transferring acyl groups;MF:beta-ureidopropionase activity;MF:hydrolase activity, acting on carbon-nitroge;MF:hydrolase activity, acting on carbon-nitroge;MF:transferase activity, transferring acyl groups;
BAB_RS29985	-	ABC transporter substrate-binding protein	0.793	-0.334864571	0.029086119	0.082996183	no	down	mRNA	20.8	15.47	16.83	15.08	12.58	11.67	17.7	13.11	COG0715	K:Transcription	------	------
BAB_RS21540	-	acyl-[acyl-carrier-protein]--UDP-N-acetylglucosamine O-acyltransferase	0.904	-0.145153687	0.029204028	0.083258689	no	down	mRNA	277.61	268.82	274.6	238.77	241.99	221.46	273.677	234.073333	COG1043	M:Cell wall/membrane/envelope biogenesis	GO:0009245;GO:0009245;GO:0005737;GO:0005737;GO:0008780;GO:00	BP:lipid A biosynthetic process;BP:lipid A biosynthetic process;CC:cytoplasm;CC:cytoplasm;MF:acyl-[acyl-carrier-protein]-UDP-N-acetylglucosamine O-acyltransferase activity;MF:acyl-[acyl-carrier-protein]-UDP-N-acetylglucosamine O-acyltransferase activity;
BAB_RS22710	-	hypothetical protein	0.886	-0.175399014	0.029312691	0.08342044	no	down	mRNA	533.75	473.04	463.9	385.78	405.35	443.27	490.23	411.466667	COG3814	S:Function unknown	------	------
BAB_RS18020	-	adenosine deaminase	0.832	-0.265249941	0.029414042	0.083634794	no	down	mRNA	17.16	15.71	15.02	11.37	12.53	13.56	15.9633	12.4866667	ENOG410XNMH	I:Lipid transport and metabolism	GO:0016021	CC:integral component of membrane;
BAB_RS22785	-	DUF2461 domain-containing protein	0.869	-0.201824266	0.0294764	0.08366405	no	down	mRNA	44.37	37.65	44.14	34.28	36.17	32.83	42.0533	34.4266667	COG5587	S:Function unknown	------	------
BAB_RS20345	-	leucyl/phenylalanyl-tRNA--protein transferase	0.801	-0.319228624	0.029761082	0.084323065	no	down	mRNA	20.55	18.06	25.08	15.09	18.48	14.45	21.23	16.0066667	COG2360	O:Posttranslational modification, protein turnover,	GO:0030163;GO:0030163;GO:0005737;GO:0005737;GO:0008914;GO:00	BP:protein catabolic process;BP:protein catabolic process;CC:cytoplasm;CC:cytoplasm;MF:leucyltransferase activity;MF:leucyltransferase activity;
BAB_RS25245	-	amino acid ABC transporter substrate-binding protein	0.719	-0.475214435	0.029975073	0.084630852	no	down	mRNA	6.84	5.22	5.6	3.84	3.66	4.4	5.88667	3.96666667	COG0834;ENOG4110X5B;ENOG4111M2K	S:Function unknown;E:Amino acid transport and m	------	------
BAB_RS25070	-	ribosome maturation factor RimM	0.853	-0.230006612	0.030297175	0.085465165	no	down	mRNA	81.94	73.21	68.01	62.76	57.79	59.61	74.3867	60.0533333	COG0806	J:Translation, ribosomal structure and biogenesis	GO:0042274;GO:0006364;GO:0042274;GO:0006364;GO:0005840;GO:00	BP:ribosomal small subunit biogenesis;BP:rRNA processing;BP:ribosomal small subunit biogenesis;BP:rRNA processing;CC:ribosome;CC:ribosome;MF:ribosome binding;MF:ribosome binding;
BAB_RS32020	-	malate dehydrogenase	0.741	-0.431936358	0.030581612	0.086116317	no	down	mRNA	8.05	6.39	7.13	6.13	4.74	4.08	7.19	4.98333333	COG0281;COG0280	C:Energy production and conversion;	GO:0004471;GO:0051287	MF:malate dehydrogenas;MF:NAD binding;
BAB_RS22390	-	sulfate ABC transporter substrate-binding protein	0.707	-0.501202483	0.031388937	0.088080921	no	down	mRNA	733.91	710.51	695.08	438.59	320.05	671.63	713.167	476.756667	COG4150	P:Inorganic ion transport and metabolism	GO:0015419;GO:1901681;GO:1901681;GO:0015419	MF:ATPase-coupled sulfate transmembrane transporter activity;MF:sulfur compound binding;MF:sulfur compound binding;MF:ATPase-coupled sulfate transmembrane transporter activity;
BAB_RS21420	-	hypothetical protein	0.841	-0.249549018	0.031427739	0.088112849	no	down	mRNA	237.56	252.98	289.99	196.64	227.18	213.81	260.177	212.543333	------	------	GO:0016021	CC:integral component of membrane;
BAB_RS29430	-	histidine kinase	0.923	-0.115242481	0.031658358	0.08852768	no	down	mRNA	183.6	178.88	195.18	160.82	164.04	160.06	185.887	161.64	ENOG410XNMH	H:Coenzyme transport and metabolism	GO:0018298;GO:0009405;GO:0005622;GO:0005622;GO:0000155;GO:00	BP:protein-chromophore linkage;BP:pathogenesis;CC:intracellular;CC:intracellular;MF:phosphorelay sensor kinase activity;MF:photoreceptor activity;MF:phosphorelay sensor kinase activity;MF:ATP binding;
BAB_RS16425	-	sulfate ABC transporter substrate-binding protein	0.84	-0.251631869	0.031748724	0.088703175	no	down	mRNA	86.62	79.98	82.37	60.69	58.44	78.25	82.99	65.7933333	COG1613	P:Inorganic ion transport and metabolism	GO:1901681;GO:0015419;GO:0015419;GO:1901681	MF:sulfur compound binding;MF:ATPase-coupled sulfate transmembrane transporter activity;MF:ATPase-coupled sulfate transmembrane transporter activity;MF:sulfur compound binding;
BAB_RS32130	-	hypothetical protein	0.828	-0.271723471	0.032308215	0.089953463	no	down	mRNA	45.88	36.01	38.91	33.31	31.57	29.36	40.2667	31.4133333	------	------	------	------
BAB_RS33210	-	hypothetical protein	0.832	-0.265486576	0.032258823	0.089971875	no	down	mRNA	48.1	46.57	57.24	36.11	46.19	37.15	50.6367	39.8166667	------	------	GO:0043565	MF:sequence-specific DNA binding;
BAB_RS19365	-	excinuclease ABC subunit UvrC	0.896	-0.158281268	0.032304926	0.090022314	no	down	mRNA	25.64	24.42	26.22	22.05	21.81	20.44	25.4267	21.4333333	COG0322	L:Replication, recombination and repair	GO:0006289;GO:0009432;GO:0006289;GO:0009432;GO:0005737;GO:00	BP:nucleotide-excision repair;BP:SOS response;BP:nucleotide-excision repair;BP:SOS response;CC:cytoplasm;CC:excinuclease repair complex;CC:cytoplasm;CC:excinuclease repair complex;MF:DNA binding;MF:excinuclease ABC activity;MF:excinuclease ABC activity;MF:DNA binding;
BAB_RS16465	-	carbohydrate kinase	0.884	-0.177278854	0.032526093	0.09009167	no	down	mRNA	68.85	67.64	64.45	53.68	57.99	56.13	66.98	55.9333333	COG0524	G:Carbohydrate transport and metabolism	GO:0016773;GO:0008865;GO:0016301;GO:0016773;GO:0008865;GO:00	MF:phosphotransferase activity, alcohol group as acceptor;MF:fructokinase activity;MF:kinase activity;MF:phosphotransferase activity, alcohol group as acceptor;MF:fructokinase activity;MF:kinase activity;MF:nucleotide binding;
BAB_RS18185	-	DNA repair protein RadA	0.896	-0.158151921	0.03297951	0.091111921	no	down	mRNA	81.35	84.55	98.16	73.37	74.71	75.04	88.02	74.3733333	COG1066	O:Posttranslational modification, protein turnover,	GO:0000725;GO:0006281;GO:0000725;GO:0008094;GO:0046872;GO:00	BP:recombinational repair;BP:DNA repair;BP:recombinational repair;MF:DNA-dependent ATPase activity;MF:metal ion binding;MF:damaged DNA binding;MF:ATP binding;MF:DNA-dependent ATPase activity;MF:metal ion binding;MF:damaged DNA binding;MF:ATP binding;
BAB_RS28580	-	AzlD family protein	0.791	-0.338608977	0.033367345	0.091946209	no	down	mRNA	175.13	123.22	106.81	101.94	99.82	104.85	135.053	102.203333	COG4541	E:Amino acid transport and metabolism	GO:0016021	CC:integral component of membrane;
BAB_RS26875	-	polyamine ABC transporter ATP-binding protein	0.904	-0.145093859	0.03356285	0.092405686	no	down	mRNA	48.08	48.77	50.57	40.58	42.29	42.81	49.14	41.8933333	COG3842	G:Carbohydrate transport and metabolism	GO:0043190;GO:0043190;GO:0015417;GO:0005524;GO:0005524;GO:00	CC:ATP-binding cassett;CC:ATP-binding cassett;MF:polyamine-transporting ATPase activity;MF:ATP binding;MF:ATP binding;MF:polyamine-transporting ATPase activity;
BAB_RS19900	-	NADH-quinone oxidoreductase subunit J	0.912	-0.133335847	0.033765001	0.092803207	no	down	mRNA	339.03	333.28	344.68	280.8	303.67	296.09	338.997	293.52	COG0839	C:Energy production and conversion	GO:0016021;GO:0008137	CC:integral component of membrane;MF:NADH dehydrogenas;
BAB_RS19680	-	transcriptional regulator NrdR	0.902	-0.148940176	0.033901376	0.093018892	no	down	mRNA	385.48	345.9	351.59	309.18	293.63	323.54	360.99	308.783333	COG1327	K:Transcription	GO:0045892;GO:0006351;GO:0045892;GO:0006351;GO:0045892;GO:00	BP:negative regulation of transcription, DNA-templated;BP:transcription, DNA-templated;BP:negative regulation of transcription, DNA-templated;BP:transcription, DNA-templated;BP:negative regulation of transcription, DNA-templated;BP:transcription, DNA-templated;MF:DNA binding;MF:zinc ion binding;MF:ATP binding;MF:ATP binding;MF:DNA binding;MF:zinc ion binding;MF:DNA binding;MF:ATP binding;MF:zinc ion binding;
BAB_RS22895	-	N-acetylmuramoyl-L-alanine amidase	0.9	-0.151334655	0.03415974	0.093567983	no	down	mRNA	95.64	97.45	95.86	81.71	81.29	82.98	96.3167	81.9933333	COG3023	M:Cell wall/membrane/envelope biogenesis	GO:0009253;GO:0008745	BP:peptidoglycan catabolic process;MF:N-acetylmuramoyl-L-alanine amidase activity;
BAB_RS28285	-	DUF4065 domain-containing protein	0.822	-0.2820952	0.034373647	0.093993641	no	down	mRNA	30.19	26.88	28.78	23.33	20.64	23.13	28.6167	22.3666667	COG3600	S:Function unknown	------	------
BAB_RS31015	-	hypothetical protein	0.862	-0.214397153	0.034616461	0.094336463	no	down	mRNA	27.39	23.13	25.44	20.75	19.92	20.94	25.32	20.5366667	ENOG4112BIC	S:Function unknown	GO:0016021	CC:integral component of membrane;
BAB_RS17520	-	Hsp33 family molecular chaperone	0.895	-0.160638225	0.034897967	0.094942565	no	down	mRNA	107.89	93.02	107.65	88.59	91.13	79.86	102.853	86.5266667	COG1281	O:Posttranslational modification, protein turnover,	GO:0006457;GO:0006457;GO:0005737;GO:0005737;GO:0051082;GO:00	BP:protein folding;BP:protein folding;CC:cytoplasm;CC:cytoplasm;MF:unfolded protein binding;MF:unfolded protein binding;
BAB_RS19490	-	helix-turn-helix domain-containing protein	0.889	-0.169496658	0.03502655	0.095131282	no	down	mRNA	243.01	206.72	210.67	177.59	194.95	182.01	220.133	184.85	COG1396	S:Function unknown	GO:0043565	MF:sequence-specific DNA binding;
BAB_RS16815	-	aspartate aminotransferase family protein	0.903	-0.146515587	0.035412052	0.096015969	no	down	mRNA	150.62	138.79	145.6	114.3	126.6	129.43	145.003	123.443333	COG0161	E:Amino acid transport and metabolism	GO:0008483;GO:0030170;GO:0008483;GO:0016223;GO:0030170;GO:00	MF:transaminase activity;MF:pyridoxal phosphate binding;MF:transaminase activity;MF:beta-alanine-pyruvate transaminase activity;MF:pyridoxal phosphate binding;MF:pyridoxal phosphate binding;MF:transaminase activity;
BAB_RS29220	-	amidohydrolase	0.905	-0.144003369	0.036457353	0.098434852	no	down	mRNA	80.5	77.82	76.61	66.85	66.39	67.11	78.31	66.7833333	COG1574	S:Function unknown	GO:0016810	MF:hydrolase activity, acting on carbon-nitroge;
BAB_RS25400	-	class I SAM-dependent methyltransferase	0.887	-0.172967043	0.03666721	0.098835358	no	down	mRNA	141.13	146.91	156.43	119.01	135.77	118.69	148.157	124.49	ENOG410XQUG	S:Function unknown	GO:0008168	MF:methyltransferase activity;
BAB_RS29300	-	alkaline phosphatase	0.793	-0.33542204	0.03675713	0.098994683	no	down	mRNA	3.14	3.55	3.29	2.36	2.5	2.62	3.32667	2.49333333	ENOG410ZVEV	S:Function unknown	GO:0009166;GO:0009166;GO:0016787;GO:0016787	BP:nucleotide catabolic process;BP:nucleotide catabolic process;MF:hydrolase activity;MF:hydrolase activity;
BAB_RS29505	-	heat shock protein HspQ	0.858	-0.220664671	0.037107858	0.099605302	no	down	mRNA	252.55	227.9	214.38	206.35	196.76	165.98	231.61	189.696667	COG3785	S:Function unknown	GO:0003677	MF:DNA binding;
BAB_RS22030	-	PAS domain-containing protein	0.864	-0.210911333	0.037487157	0.100539429	no	down	mRNA	33.88	29.73	32.8	27.63	25.43	25.56	32.1367	26.2066667	COG5388	S:Function unknown	------	------
BAB_RS22575	-	pyridoxamine 5'-phosphate oxidase family protein	0.9	-0.152749408	0.037729647	0.101105385	no	down	mRNA	69.53	65.44	67.22	54.02	60.01	57.37	67.3967	57.1333333	COG3467	S:Function unknown	GO:0048037	MF:cofactor binding;
BAB_RS23870	-	tRNA glutamyl-Q(34) synthetase GluQRS	0.833	-0.26282314	0.037905747	0.101323764	no	down	mRNA	19.76	20.17	22.52	17.82	17.09	14.27	20.8167	16.3933333	COG0008	L:Replication, recombination and repair	GO:0006418;GO:0006418;GO:0006418;GO:0005737;GO:0005737;GO:00	BP:tRNA aminoacylation for protein translation;BP:tRNA aminoacylation for protein translation;BP:tRNA aminoacylation for protein translation;CC:cytoplasm;CC:cytoplasm;CC:cytoplasm;MF:aminoacyl-tRNA ligase activity;MF:ATP binding;MF:aminoacyl-tRNA ligase activity;MF:ATP binding;MF:ATP binding;MF:glutamate-tRNA ligase activity;
BAB_RS26465	-	RNA polymerase subunit sigma-24	0.88	-0.184300339	0.037893999	0.101376702	no	down	mRNA	50.58	51.98	60.25	45.47	46.09	44.29	54.27	45.2833333	COG1595	S:Function unknown	GO:0006352;GO:0006352;GO:0003700;GO:0003677;GO:0016987;GO:00	BP:DNA-templated transcription, initiation;BP:DNA-templated transcription, initiation;MF:transcription factor activity, sequence-specific DNA binding;MF:DNA binding;MF:sigma factor activity;MF:sigma factor activity;MF:DNA binding;MF:transcription factor activity, sequence-specific DNA binding;
BAB_RS18140	-	TIGR00730 family Rossman fold protein	0.903	-0.147818769	0.038658544	0.103078758	no	down	mRNA	96.04	89.12	94.84	82.77	80.63	75	93.3333	79.4666667	COG1611	S:Function unknown	GO:0009691;GO:0016787	BP:cytokinin biosynthetic process;MF:hydrolase activity;
BAB_RS23940	-	hypothetical protein	0.909	-0.137265823	0.038697728	0.103097679	no	down	mRNA	830.03	734.6	733.92	644.98	659.09	675.79	766.183	659.953333	------	------	GO:0016021	CC:integral component of membrane;
BAB_RS27180	-	FCD domain-containing protein	0.902	-0.14893634	0.038738604	0.103121073	no	down	mRNA	76.55	67.02	79.37	59.02	64.32	65.83	74.3133	63.0566667	COG2186	S:Function unknown	GO:0006351;GO:0003700;GO:0003677	BP:transcription, DNA-templated;MF:transcription factor activity, sequence-specific DNA binding;MF:DNA binding;
BAB_RS17205	-	ImmA/IrrE family metallo-endopeptidase	0.901	-0.150552781	0.039488654	0.104770475	no	down	mRNA	84.87	76.45	85.7	66.58	70.34	73.33	82.34	70.0833333	COG2856	S:Function unknown	GO:0016787	MF:hydrolase activity;
BAB_RS22545	-	hypothetical protein	0.848	-0.238470739	0.039556118	0.104862877	no	down	mRNA	56.12	55.22	48.32	39.6	44.29	44.3	53.22	42.73	ENOG4111W3C	S:Function unknown	GO:0016021	CC:integral component of membrane;
BAB_RS31850	-	branched-chain amino acid ABC transporter permease	0.811	-0.301403511	0.039957143	0.105751484	no	down	mRNA	26.67	31.23	24.84	22.97	21.28	19.35	27.58	21.2	COG4177	E:Amino acid transport and metabolism	GO:0016021;GO:0005886;GO:0022857	CC:integral component of membrane;CC:plasma membrane;MF:transmembrane transporter activity;
BAB_RS32720	-	hypothetical protein	0.839	-0.253438676	0.039949808	0.105819236	no	down	mRNA	13.36	10.29	14.7	10.13	9.68	10.4	12.7833	10.07	------	------	------	------
BAB_RS20440	-	RNA-binding protein	0.902	-0.149468181	0.040143238	0.106069263	no	down	mRNA	68.06	59.42	61.22	55.21	52.95	51.78	62.9	53.3133333	ENOG410XQJZ	S:Function unknown	GO:0016021	CC:integral component of membrane;
BAB_RS26750	-	sigma-54-dependent Fis family transcriptional regulator	0.902	-0.148665457	0.040480028	0.106521155	no	down	mRNA	43.71	44.48	45.15	38.96	36.45	38.03	44.4467	37.8133333	COG2204	E:Amino acid transport and metabolism	GO:0000160;GO:0006355;GO:0006351;GO:0005622;GO:0043565;GO:00	BP:phosphorelay signal transduction system;BP:regulation of transcription, DNA-templated;BP:transcription, DNA-templated;CC:intracellular;MF:sequence-specific DNA binding;MF:transcription factor binding;MF:ATP binding;
BAB_RS31325	-	GntR family transcriptional regulator	0.779	-0.359799908	0.040614678	0.106788019	no	down	mRNA	18.31	13.48	14.62	10.66	12.65	10.38	15.47	11.23	COG1802;ENOG4111XU0;ENOG410ZZTA	S:Function unknown;K:Transcription;K:Transcriptio	GO:0006351;GO:0006351;GO:0003700;GO:0003677;GO:0003677;GO:00	BP:transcription, DNA-templated;BP:transcription, DNA-templated;MF:transcription factor activity, sequence-specific DNA binding;MF:DNA binding;MF:DNA binding;MF:transcription factor activity, sequence-specific DNA binding;
BAB_RS28975	-	ABC transporter ATP-binding protein	0.855	-0.225537195	0.04072628	0.106993898	no	down	mRNA	23.98	20.34	25.03	20.28	16.63	18.96	23.1167	18.6233333	COG3839;COG3842	G:Carbohydrate transport and metabolism;	GO:0016887;GO:0005524;GO:0005524;GO:0016887	MF:ATPase activity;MF:ATP binding;MF:ATP binding;MF:ATPase activity;
BAB_RS20165	-	Lrp/AsnC family transcriptional regulator	0.87	-0.200945237	0.040927511	0.107347015	no	down	mRNA	48.02	46.95	47.84	35.03	37.84	44.36	47.6033	39.0766667	COG1522	K:Transcription	GO:0006351;GO:0005622;GO:0003700;GO:0043565	BP:transcription, DNA-templated;CC:intracellular;MF:transcription factor activity, sequence-specific DNA binding;MF:sequence-specific DNA binding;
BAB_RS17310	-	integrase	0.809	-0.305388568	0.041103359	0.107632513	no	down	mRNA	12.46	10.83	10.93	9.71	7.09	9.28	11.4067	8.69333333	COG0582	S:Function unknown	GO:0006310;GO:0015074;GO:0015074;GO:0006310;GO:0003677;GO:00	BP:DNA recombination;BP:DNA integration;BP:DNA integration;BP:DNA recombination;MF:DNA binding;MF:DNA binding;
BAB_RS31210	-	IS3 family transposase	0.833	-0.263804336	0.041241993	0.107907592	no	down	mRNA	9.91	9.43	10.37	7.27	8.81	7.13	9.90333	7.73666667	COG2801	L:Replication, recombination and repair	GO:0015074;GO:0006313;GO:0003677;GO:0004803	BP:DNA integration;BP:transposition, DNA-mediated;MF:DNA binding;MF:transposase activity;
BAB_RS30940	-	GntR family transcriptional regulator	0.87	-0.201432149	0.041521049	0.108461081	no	down	mRNA	388.8	425.8	511.93	343.32	369.28	382.54	442.177	365.046667	COG1802	J:Translation, ribosomal structure and biogenesis	GO:0006351;GO:0006351;GO:0003700;GO:0003677;GO:0003677;GO:00	BP:transcription, DNA-templated;BP:transcription, DNA-templated;MF:transcription factor activity, sequence-specific DNA binding;MF:DNA binding;MF:DNA binding;MF:transcription factor activity, sequence-specific DNA binding;
BAB_RS23265	-	hypothetical protein	0.907	-0.141247888	0.041594793	0.108565451	no	down	mRNA	231.08	206.2	221.12	173.48	195.83	193.84	219.467	187.716667	COG5465	S:Function unknown	------	------
BAB_RS23220	-	BA14K family protein	0.911	-0.133687365	0.041903208	0.108927999	no	down	mRNA	375.28	328.9	356.21	315.74	289.77	304.58	353.463	303.363333	ENOG41125UP	S:Function unknown	GO:0009405;GO:0016021	BP:pathogenesis;CC:integral component of membrane;
BAB_RS32530	-	hypothetical protein	0.552	-0.857798852	0.041892044	0.108987156	no	down	mRNA	5.25	3.73	4.83	3.68	1.07	2.57	4.60333	2.44	ENOG410Z5YV	S:Function unknown	GO:0016021	CC:integral component of membrane;
BAB_RS17650	-	3-methyl-2-oxobutanoate hydroxymethyltransferase	0.893	-0.16257838	0.042371989	0.109879903	no	down	mRNA	65.29	68.5	71.37	55.06	60.01	58.56	68.3867	57.8766667	COG0413	H:Coenzyme transport and metabolism	GO:0015940;GO:0015940;GO:0015940;GO:0005737;GO:0005737;GO:00	BP:pantothenate biosynthetic process;BP:pantothenate biosynthetic process;BP:pantothenate biosynthetic process;CC:cytoplasm;CC:cytoplasm;CC:cytoplasm;MF:methyltransferase activity;MF:metal ion binding;MF:3-methyl-2-oxobutanoate hydroxymethyltransferase activity;MF:3-methyl-2-oxobutanoate hydroxymethyltransferase activity;MF:metal ion binding;MF:metal ion binding;MF:3-methyl-2-oxobutanoate hydroxymethyltransferase activity;MF:methyltransferase activity;
BAB_RS17705	-	C4-dicarboxylate ABC transporter	0.584	-0.776318722	0.04242959	0.109940543	no	down	mRNA	12.25	6.36	7.28	2.68	5.58	5.49	8.63	4.58333333	COG4665	Q:Secondary metabolites biosynthesis, transport a	GO:0016021	CC:integral component of membrane;
BAB_RS28365	-	AcrB/AcrD/AcrF family protein	0.857	-0.222198866	0.044136036	0.113538096	no	down	mRNA	8.81	7.53	8.12	6.62	6.79	6.22	8.15333	6.54333333	COG0841	P:Inorganic ion transport and metabolism	GO:0016021;GO:0022857	CC:integral component of membrane;MF:transmembrane transporter activity;
BAB_RS23655	-	AsmA family protein	0.912	-0.132335834	0.044303807	0.113787476	no	down	mRNA	31.05	28	30.55	24.85	25.64	26.34	29.8667	25.61	COG2982	M:Cell wall/membrane/envelope biogenesis	------	------
BAB_RS19530	-	hypothetical protein	0.412	-1.279159638	0.044730004	0.114790339	no	down	mRNA	2.5	2.65	1.79	1	1.26	0.27	2.31333	0.84333333	ENOG410YJV0	S:Function unknown	------	------
BAB_RS21515	-	competence protein ComEC	0.894	-0.161387788	0.044835336	0.114968822	no	down	mRNA	14.12	13.28	16.5	11.85	12.35	12.72	14.6333	12.3066667	COG0658	S:Function unknown	GO:0016021	CC:integral component of membrane;
BAB_RS24325	-	DUF1036 domain-containing protein	0.893	-0.163625677	0.0450499	0.115334922	no	down	mRNA	134.08	115.66	125.45	102.85	107.31	109.07	125.063	106.41	COG5480	S:Function unknown	------	------
BAB_RS30610	-	UbiX family flavin prenyltransferase	0.629	-0.669146587	0.045213907	0.115570632	no	down	mRNA	3.8	2.85	4.86	2.17	1.83	2.99	3.83667	2.33	COG0163	H:Coenzyme transport and metabolism	GO:0051188;GO:0051188;GO:0051188;GO:0016829;GO:0004659;GO:00	BP:cofactor biosynthetic process;BP:cofactor biosynthetic process;BP:cofactor biosynthetic process;MF:lyase activity;MF:prenyltransferase activity;MF:lyase activity;MF:prenyltransferase activity;
BAB_RS23570	-	RNA methyltransferase	0.834	-0.262198124	0.045203919	0.115637095	no	down	mRNA	15.92	14.95	17.06	12.22	13.73	11.73	15.9767	12.56	COG0566	P:Inorganic ion transport and metabolism	GO:0006396;GO:0006396;GO:0008173;GO:0003723;GO:0003723;GO:00	BP:RNA processing;BP:RNA processing;MF:RNA methyltransferase activity;MF:RNA binding;MF:RNA binding;MF:RNA methyltransferase activity;
BAB_RS20770	-	D-alanyl-D-alanine carboxypeptidase	0.884	-0.177661496	0.045423223	0.115921221	no	down	mRNA	37.17	39.85	42.55	34.46	31.06	34.54	39.8567	33.3533333	COG1686	M:Cell wall/membrane/envelope biogenesis	GO:0009002	MF:serine-type D-Ala-D-Ala carboxypeptidase activity;
BAB_RS17625	-	LysR family transcriptional regulator	0.874	-0.194732681	0.045559971	0.116085794	no	down	mRNA	39.39	36.93	44.84	36.13	32.63	31.27	40.3867	33.3433333	ENOG410XNYY	K:Transcription	GO:0006351;GO:0006351;GO:0003700;GO:0003677;GO:0003677;GO:00	BP:transcription, DNA-templated;BP:transcription, DNA-templated;MF:transcription factor activity, sequence-specific DNA binding;MF:DNA binding;MF:DNA binding;MF:transcription factor activity, sequence-specific DNA binding;
BAB_RS30580	-	nitrate reductase subunit alpha	0.903	-0.146550195	0.04552814	0.116096758	no	down	mRNA	658.95	681.11	692.43	557.47	564.09	607.47	677.497	576.343333	COG5013	C:Energy production and conversion	GO:0042126;GO:0042126;GO:0009325;GO:0009325;GO:0009055;GO:00	BP:nitrate metabolic process;BP:nitrate metabolic process;CC:nitrate reductase complex;CC:nitrate reductase complex;MF:electron carrier activity;MF:iron-sulfur cluster binding;MF:metal ion binding;MF:molybdopterin cofactor binding;MF:nitrate reductase activity;MF:nitrate reductase activity;MF:molybdopterin cofactor binding;MF:iron-sulfur cluster binding;MF:mannose-1-phosphate guanylyltransferase activity;MF:metal ion binding;MF:electron carrier activity;
BAB_RS29240	-	amino acid ABC transporter	0.872	-0.197839565	0.045786291	0.116570012	no	down	mRNA	44.25	38.99	43.46	31.4	37.04	35.77	42.2333	34.7366667	ENOG410YVP9;COG0834	E:Amino acid transport and metabolism;	GO:0016020;GO:0016020;GO:0004970;GO:0004970	CC:membrane;CC:membrane;MF:ionotropic glutamate receptor activity;MF:ionotropic glutamate receptor activity;
BAB_RS32610	-	hypothetical protein	0.773	-0.371079267	0.045883099	0.116723988	no	down	mRNA	22.84	31.09	20.84	20.37	16.66	17.91	24.9233	18.3133333	------	------	------	------
BAB_RS16840	-	Crp/Fnr family transcriptional regulator	0.869	-0.203219361	0.046135455	0.117273115	no	down	mRNA	30.31	28.88	28.25	24.11	24.66	22.83	29.1467	23.8666667	COG0664	T:Signal transduction mechanisms	GO:0006355;GO:0006351;GO:0006355;GO:0006351;GO:0003677;GO:00	BP:regulation of transcription, DNA-templated;BP:transcription, DNA-templated;BP:regulation of transcription, DNA-templated;BP:transcription, DNA-templated;MF:DNA binding;MF:DNA binding;

BAB_RS26280	-	enoyl-[acyl-carrier-protein] reductase FabI	0.924	-0.114285803	0.046282747	0.117554519	no	down	mRNA	295.23	280.98	292.67	248.62	260.08	250.46	289.627	253.053333	COG0623	J:Translation, ribosomal structure and biogenesis	GO:0006633;GO:0006633;GO:0004318;GO:0000166;GO:0004318	BP:fatty acid biosynthetic process;BP:fatty acid biosynthetic process;MF:enoyl-[acyl-carrier-protein] reductas;MF:nucleotide binding;MF:enoyl-[acyl-carrier-protein] reductas;
BAB_RS19755	-	undecaprenyl-phosphate glucose phosphotransferase	0.897	-0.156196558	0.046788213	0.118463773	no	down	mRNA	32.76	33.5	35.09	27.93	27.54	30.38	33.7833	28.6166667	COG2148	M:Cell wall/membrane/envelope biogenesis	GO:0016021;GO:0016740	CC:integral component of membrane;MF:transferase activity;
BAB_RS17900	-	F0F1 ATP synthase subunit B'	0.913	-0.131486594	0.047323396	0.119442319	no	down	mRNA	448.61	454.25	469.85	419.94	378.62	388.37	457.57	395.643333	COG0711	C:Energy production and conversion	GO:0015986;GO:0015986;GO:0015986;GO:0016021;GO:0005886;GO:00	BP:ATP synthesis coupled proton transport;BP:ATP synthesis coupled proton transport;BP:ATP synthesis coupled proton transport;CC:integral component of membrane;CC:plasma membrane;CC:proton-transporting ATP synthase complex, coupling factor;CC:proton-transporting ATP synthase complex, coupling factor;CC:integral component of membrane;CC:plasma membrane;CC:integral component of membrane;CC:proton-transporting ATP synthase complex, coupling factor;CC:plasma membrane;MF:proton-transporting ATP synthase activity, rotational mechanism;MF:proton-transporting ATP synthase activity, rotational mechanism;MF:hydrogen ion transmembrane transporter activity;MF:proton-transporting ATP synthase activity, rotational mechanism;
BAB_RS28460	-	LysR family transcriptional regulator	0.588	-0.766219753	0.047288559	0.119448224	no	down	mRNA	48.82	68.92	45.31	40.98	23.06	34.14	54.35	32.7266667	ENOG410YGJ0;ENOG410Y19Q	K:Transcription;	GO:0006351;GO:0003677;GO:0003700	BP:transcription, DNA-templated;MF:DNA binding;MF:transcription factor activity, sequence-specific DNA binding;
BAB_RS22135	-	DUF2218 domain-containing protein	0.825	-0.277383329	0.047404626	0.119553425	no	down	mRNA	186.12	182.84	158.14	158.31	131.97	138.68	175.7	142.986667	COG3553	S:Function unknown	------	------
BAB_RS19705	hppA	K+-insensitive pyrophosphate-energized proton pump	0.908	-0.139751212	0.047818055	0.120407063	no	down	mRNA	109.93	107.92	115.77	86.09	100.23	98.68	111.207	95	COG3808	C:Energy production and conversion	GO:1902600;GO:1902600;GO:1902600;GO:0005887;GO:0016021;GO:00	BP:hydrogen ion transmembrane transport;BP:hydrogen ion transmembrane transport;BP:hydrogen ion transmembrane transport;CC:integral component of plasma membrane;CC:integral component of membrane;CC:plasma membrane;CC:integral component of membrane;CC:integral component of plasma membrane;CC:plasma membrane;MF:hydrogen-translocating pyrophosphatase activity;MF:inorganic diphosphatase activity;MF:magnesium ion binding;MF:hydrogen-translocating pyrophosphatase activity;MF:inorganic diphosphatase activity;MF:metal ion binding;MF:inorganic diphosphatase activity;MF:magnesium ion binding;MF:hydrogen-translocating pyrophosphatase activity;MF:metal ion binding;
BAB_RS23660	-	putrescine ABC transporter permease PotI	0.883	-0.179517281	0.048528191	0.121908584	no	down	mRNA	66.72	67.32	83.63	56.66	64.23	60.46	72.5567	60.45	COG1177	P:Inorganic ion transport and metabolism	GO:0055085;GO:0016021;GO:0005886	BP:transmembrane transport;CC:integral component of membrane;CC:plasma membrane;
BAB_RS17940	-	membrane protein	0.654	-0.612655676	0.048616564	0.121939907	no	down	mRNA	353.12	294.25	436.9	262.78	289.78	205.51	361.423	252.69	ENOG410XZUE	S:Function unknown	GO:0016021	CC:integral component of membrane;
BAB_RS29055	-	FAD-binding dehydrogenase	0.889	-0.170401803	0.048616483	0.122034968	no	down	mRNA	21.68	23.99	25.32	19.82	20.05	19.74	23.6633	19.87	COG3573	C:Energy production and conversion	GO:0016627;GO:0016627;GO:0004497	MF:oxidoreductase activity, acting on the CH-CH group of donors;MF:oxidoreductase activity, acting on the CH-CH group of donors;MF:monooxygenase activity;
BAB_RS26910	-	TetR/AcrR family transcriptional regulator	0.861	-0.216367411	0.049043684	0.122819451	no	down	mRNA	59.96	59.5	60.45	54.73	49.62	41.95	59.97	48.7666667	ENOG4111WDK	K:Transcription	GO:0006355;GO:0006351;GO:0006355;GO:0006351;GO:0003677;GO:00	BP:regulation of transcription, DNA-templated;BP:transcription, DNA-templated;BP:regulation of transcription, DNA-templated;BP:transcription, DNA-templated;MF:DNA binding;MF:DNA binding;
BAB_RS22565	-	fluoride efflux transporter CrcB	0.9	-0.151387564	0.049084409	0.122825706	no	down	mRNA	691.69	602.03	619.05	593.57	527.6	533.26	637.59	551.476667	COG0239	D:Cell cycle control, cell division, chromosome par	GO:0016021;GO:0005887;GO:0005886;GO:0005887;GO:0005887;GO:00	CC:integral component of membrane;CC:integral component of plasma membrane;CC:plasma membrane;CC:integral component of plasma membrane;CC:integral component of plasma membrane;MF:inorganic anion transmembrane transporter activity;MF:inorganic anion transmembrane transporter activity;MF:inorganic anion transmembrane transporter activity;
BAB_RS23835	-	-	0.671	-0.576434773	0.049270555	0.123195558	no	down	mRNA	21373.5	32085.2	24827.4	20065.32	9837.88	19879.81	26095.4	16594.3367	COG0048;ENOG4111UA7;ENOG4111VRG	S:Function unknown;	------	------
BAB_RS31620	-	flagellin	0.866	-0.208012614	0.049401889	0.123427893	no	down	mRNA	80.21	66.76	65.05	61.37	53.35	58.13	70.6733	57.6166667	COG1344	N:Cell motility	GO:0071973;GO:0071973;GO:0009420;GO:0005576;GO:0005576;GO:00	BP:bacterial-type flagellum-dependent cell motility;BP:bacterial-type flagellum-dependent cell motility;CC:bacterial-type flagellum filament;CC:extracellular region;CC:extracellular region;CC:bacterial-type flagellum filament;MF:structural molecule activity;MF:structural molecule activity;
BAB_RS16905	-	aldehyde dehydrogenase family protein	0.902	-0.149305412	0.049786299	0.12419517	no	down	mRNA	41.02	41.26	45.37	33.32	36.96	38.18	42.55	36.1533333	COG1012	V:Defense mechanisms	GO:0004029;GO:0016620	MF:aldehyde dehydrogenas;MF:oxidoreductase activity, acting on the aldehyde or oxo group of donors, NAD or NADP as acceptor;
BAB_RS16370	-	heme exporter protein CcmB	0.869	-0.202856292	0.049871439	0.124311043	no	down	mRNA	42.4	38.71	41.95	30.42	34.75	36.01	41.02	33.7266667	COG2386	O:Posttranslational modification, protein turnover,	GO:0017004;GO:0017004;GO:0016021;GO:0005886;GO:0016021;GO:00	BP:cytochrome complex assembly;BP:cytochrome complex assembly;CC:integral component of membrane;CC:plasma membrane;CC:integral component of membrane;CC:plasma membrane;MF:heme transporter activity;MF:heme transporter activity;
BAB_RS21785	-	replication-associated recombination protein A	0.899	-0.152852112	0.049927759	0.124354953	no	down	mRNA	31.74	27.45	32.46	26.66	25.41	25.44	30.55	25.8366667	COG2256	L:Replication, recombination and repair	GO:0006260;GO:0006260;GO:0006260;GO:0005524;GO:0003677;GO:00	BP:DNA replication;BP:DNA replication;BP:DNA replication;MF:ATP binding;MF:DNA binding;MF:kinase activity;MF:ATP binding;MF:DNA binding;MF:ATP binding;MF:DNA binding;
BAB_RS20600	-	FAD-dependent monooxygenase	0.834	-0.262126026	0.050064626	0.124502819	no	down	mRNA	9.31	7.16	9.46	6.94	6.54	6.79	8.64333	6.75666667	COG0654	S:Function unknown	GO:0071949;GO:0004497	MF:FAD binding;MF:monooxygenase activity;
BAB_RS23215	-	cold-shock protein	0.908	-0.139935871	0.050835129	0.126126078	no	down	mRNA	23866.3	23465.6	22829.5	19196.2	21366.3	20665.15	23387.2	20409.2167	COG1278	L:Replication, recombination and repair	------	------
BAB_RS17745	-	xanthine dehydrogenase accessory protein XdhC	0.801	-0.319712122	0.050881281	0.126143175	no	down	mRNA	8.8	9.86	11.27	7.8	7.24	7.88	9.97667	7.64	COG1975	O:Posttranslational modification, protein turnover,	------	------
BAB_RS18835	-	hypothetical protein	0.791	-0.338971983	0.051239405	0.126542819	no	down	mRNA	12.34	12.26	13.82	11.84	8.17	8.83	12.8067	9.61333333	COG5323	S:Function unknown	------	------
BAB_RS17280	-	UPF0262 family protein	0.873	-0.196308704	0.05144451	0.126854345	no	down	mRNA	74.55	68.08	75.42	60.98	63.41	57.03	72.6833	60.4733333	COG5328	S:Function unknown	------	------
BAB_RS22955	-	hypothetical protein	0.825	-0.276787084	0.052469509	0.128788796	no	down	mRNA	21.9	23.43	25.98	21.11	17.01	18.08	23.77	18.7333333	------	------	------	------
BAB_RS26495	-	ABC transporter ATP-binding protein	0.727	-0.459856605	0.05246115	0.128866723	no	down	mRNA	6.64	6.01	9.16	4.14	5.54	5.44	7.27	5.04	COG0411	S:Function unknown	GO:0016887;GO:0005524;GO:0005524;GO:0016887	MF:ATPase activity;MF:ATP binding;MF:ATP binding;MF:ATPase activity;
BAB_RS29730	-	SURF1 family protein	0.831	-0.266437279	0.052749713	0.129377731	no	down	mRNA	22.74	22.39	20.45	18.41	18.15	14.76	21.86	17.1066667	COG3346	S:Function unknown	GO:0016021;GO:0005886	CC:integral component of membrane;CC:plasma membrane;
BAB_RS32400	-	hypothetical protein	0.689	-0.53743664	0.052883663	0.12960733	no	down	mRNA	7.18	7.62	7.86	5.72	4.04	5.36	7.55333	5.04	------	------	------	------
BAB_RS24425	-	monofunctional biosynthetic peptidoglycan transglycosylase	0.9	-0.151891828	0.053008603	0.129814513	no	down	mRNA	24.21	23.6	25	21.52	20.02	20.22	24.27	20.5866667	COG0744	J:Translation, ribosomal structure and biogenesis	GO:0071555;GO:0009252;GO:0008360;GO:0071555;GO:0008360;GO:00	BP:cell wall organization;BP:peptidoglycan biosynthetic process;BP:regulation of cell shape;BP:cell wall organization;BP:regulation of cell shape;BP:peptidoglycan biosynthetic process;CC:integral component of membrane;CC:peptidoglycan-based cell wall;CC:plasma membrane;CC:peptidoglycan-based cell wall;CC:integral component of membrane;CC:plasma membrane;MF:peptidoglycan glycosyltransferase activity;MF:transferase activity, transferring pentosyl groups;MF:peptidoglycan glycosyltransferase activity;MF:transferase activity, transferring pentosyl groups;
BAB_RS32935	-	hypothetical protein	0.748	-0.419073905	0.053875416	0.131636282	no	down	mRNA	11.37	9.72	11.19	8.94	6.22	7.86	10.76	7.67333333	------	------	------	------
BAB_RS26080	-	orotidine-5'-phosphate decarboxylase	0.908	-0.139053817	0.053978163	0.131687043	no	down	mRNA	72.3	64.82	77.1	62.55	60.21	60.85	71.4067	61.2033333	COG0284	F:Nucleotide transport and metabolism	GO:0006207;GO:0044205;GO:0006207;GO:0044205;GO:0004590;GO:00	BP:'de novo' pyrimidine nucleobase biosynthetic process;BP:'de novo' UMP biosynthetic process;BP:'de novo' pyrimidine nucleobase biosynthetic process;BP:'de novo' UMP biosynthetic process;MF:orotidine-5'-phosphate decarboxylase activity;MF:hydrolase activity;MF:orotidine-5'-phosphate decarboxylase activity;
BAB_RS26530	-	shikimate dehydrogenase	0.712	-0.491062046	0.053944164	0.131704103	no	down	mRNA	4.24	4.85	5.33	3.57	3.67	2.49	4.80667	3.24333333	COG0169	S:Function unknown	GO:0004764	MF:shikimate 3-dehydrogenas;
BAB_RS22650	-	tRNA (adenosine(37)-N6)-dimethylallyltransferase MiaA	0.852	-0.230390579	0.054267327	0.13229205	no	down	mRNA	16.84	14.17	17.79	14.55	12.57	12	16.2667	13.04	COG0324	J:Translation, ribosomal structure and biogenesis	GO:0008033;GO:0008033;GO:0008033;GO:0052381;GO:0005524;GO:00	BP:tRNA processing;BP:tRNA processing;BP:tRNA processing;MF:tRNA dimethylallyltransferase activity;MF:ATP binding;MF:tRNA dimethylallyltransferase activity;MF:transferase activity, transferring acyl groups;MF:ATP binding;MF:transferase activity, transferring acyl groups;MF:tRNA dimethylallyltransferase activity;MF:ATP binding;
BAB_RS19500	-	integrase	0.863	-0.211781403	0.054527985	0.132726073	no	down	mRNA	11.32	8.94	11.25	8.27	8.16	9.09	10.5033	8.50666667	COG2801	S:Function unknown	GO:0015074;GO:0015074;GO:0003676;GO:0003676	BP:DNA integration;BP:DNA integration;MF:nucleic acid binding;MF:nucleic acid binding;
BAB_RS22765	-	antibiotic acetyltransferase	0.886	-0.174287731	0.055510731	0.13450677	no	down	mRNA	50.52	42.12	49.85	40.08	41.04	37.89	47.4967	39.67	COG0110	S:Function unknown	GO:0016740;GO:0008811;GO:0016740;GO:0008811	MF:transferase activity;MF:chloramphenicol O-acetyltransferase activity;MF:transferase activity;MF:chloramphenicol O-acetyltransferase activity;
BAB_RS21825	-	50S ribosomal protein L15	0.896	-0.157795816	0.056672582	0.136806165	no	down	mRNA	637.86	618.42	750.46	578	579.64	563.44	668.913	573.693333	COG0200	J:Translation, ribosomal structure and biogenesis	GO:0006412;GO:0006412;GO:0015934;GO:0015934;GO:0019843;GO:00	BP:translation;BP:translation;CC:large ribosomal subunit;CC:large ribosomal subunit;MF:rRNA binding;MF:structural constituent of ribosome;MF:structural constituent of ribosome;MF:rRNA binding;
BAB_RS17090	-	hypothetical protein	0.83	-0.268417234	0.05685578	0.137145361	no	down	mRNA	44.47	29.6	34.81	27.71	29.58	27.16	36.2933	28.15	------	------	------	------
BAB_RS18505	-	GDP-mannose 4%2C6-dehydratase	0.904	-0.145816185	0.057322783	0.137961125	no	down	mRNA	325.2	275.8	279.56	247.09	255.48	247.24	293.52	249.936667	COG0223	J:Translation, ribosomal structure and biogenesis	GO:0009058;GO:0009058;GO:0016742;GO:0016742;GO:0008446	BP:biosynthetic process;BP:biosynthetic process;MF:hydroxymethyl-, formyl- and related transferase activity;MF:hydroxymethyl-, formyl- and related transferase activity;MF:GDP-mannose 4,6-dehydratase activity;
BAB_RS28315	-	metallophosphatase	0.863	-0.213288602	0.057584406	0.138487048	no	down	mRNA	18.32	18.44	20.12	15.29	14.63	16.61	18.96	15.51	COG1407	S:Function unknown	GO:0016787	MF:hydrolase activity;
BAB_RS22350	-	hypothetical protein	0.832	-0.265182489	0.058007086	0.139191014	no	down	mRNA	62.62	71.63	66.34	46.92	47.4	64.38	66.8633	52.9	------	------	GO:0016021	CC:integral component of membrane;
BAB_RS24445	-	hypothetical protein	0.862	-0.213447767	0.058257995	0.139688759	no	down	mRNA	25.35	22.93	30.49	18.57	22.66	22.74	26.2567	21.3233333	ENOG410XRK1;ENOG410ZVF3	G:Carbohydrate transport and metabolism;	GO:0055085;GO:0016021	BP:transmembrane transport;CC:integral component of membrane;
BAB_RS17695	-	histidine kinase	0.871	-0.199046227	0.058355029	0.139817084	no	down	mRNA	24.05	19.53	21.22	18.32	16.16	18.54	21.6	17.6733333	ENOG410XNMH	S:Function unknown	GO:0016021;GO:0005622;GO:0016021;GO:0005622;GO:0000155;GO:00	CC:integral component of membrane;CC:intracellular;CC:integral component of membrane;CC:intracellular;MF:phosphorelay sensor kinase activity;MF:protein dimerization activity;MF:phosphorelay sensor kinase activity;MF:protein dimerization activity;MF:ATP binding;
BAB_RS26485	-	branched-chain amino acid ABC transporter permease	0.739	-0.435587444	0.058610435	0.140219901	no	down	mRNA	8.21	5.44	6.54	3.84	4.34	5.76	6.73	4.64666667	COG0559	S:Function unknown	GO:0016021;GO:0005886;GO:0022857	CC:integral component of membrane;CC:plasma membrane;MF:transmembrane transporter activity;
BAB_RS17905	-	ATP synthase subunit b 2	0.92	-0.120879435	0.058690511	0.140307003	no	down	mRNA	493.04	431.48	466.1	413.58	389	414.88	463.54	405.82	COG0711	C:Energy production and conversion	GO:0015986;GO:0015986;GO:0045263;GO:0016021;GO:0005886;GO:00	BP:ATP synthesis coupled proton transport;BP:ATP synthesis coupled proton transport;CC:proton-transporting ATP synthase complex, coupling factor;CC:integral component of membrane;CC:plasma membrane;CC:proton-transporting ATP synthase complex, coupling factor;CC:integral component of membrane;CC:plasma membrane;MF:proton-transporting ATP synthase activity, rotational mechanism;MF:hydrolase activity, acting on glycosyl bonds;MF:hydrogen ion transmembrane transporter activity;MF:proton-transporting ATP synthase activity, rotational mechanism;MF:hydrogen ion transmembrane transporter activity;
BAB_RS18110	-	GlsB/YeaQ/YmgE family stress response membrane protein	0.838	-0.255203596	0.058606828	0.140315753	no	down	mRNA	547.88	462.21	353.23	321	375.7	394.11	454.44	363.603333	COG2261	S:Function unknown	GO:0016021	CC:integral component of membrane;
BAB_RS31930	-	hypothetical protein	0.875	-0.192542159	0.059215977	0.141143125	no	down	mRNA	6.66	6.29	7.44	5.62	5.49	5.7	6.79667	5.60333333	COG1024	I:Lipid transport and metabolism	------	------
BAB_RS22125	-	-	0.437	-1.195790546	0.059531088	0.141683988	no	down	mRNA	18.78	56.12	47.29	23.59	21.96	13.75	40.73	19.7666667	------	------	------	------
BAB_RS21060	-	cysteine synthase A	0.852	-0.231195313	0.05994784	0.141730986	no	down	mRNA	133.71	117.19	117.93	101.27	81.67	113.64	122.943	98.86	COG0031	E:Amino acid transport and metabolism	GO:0006535;GO:0006535;GO:0004124	BP:cysteine biosynthetic process from serine;BP:cysteine biosynthetic process from serine;MF:cysteine synthase activity;
BAB_RS31090	-	methylglyoxal synthase	0.907	-0.140278045	0.05991338	0.141753822	no	down	mRNA	247.15	255.41	266.12	225.23	218.74	225.12	256.227	223.03	COG1803	G:Carbohydrate transport and metabolism	GO:0019242;GO:0019242;GO:0008929;GO:0008929;GO:0016491	BP:methylglyoxal biosynthetic process;BP:methylglyoxal biosynthetic process;MF:methylglyoxal synthase activity;MF:methylglyoxal synthase activity;MF:oxidoreductase activity;
BAB_RS25560	-	cobaltochelatase subunit CobT	0.925	-0.111887908	0.060387716	0.142561155	no	down	mRNA	36.08	33.12	36.25	29.83	31.64	30.21	35.15	30.56	COG4547	H:Coenzyme transport and metabolism	GO:0009236;GO:0009236;GO:0051116	BP:cobalamin biosynthetic process;BP:cobalamin biosynthetic process;MF:cobaltochelatase activity;
BAB_RS25280	-	asparaginase	0.673	-0.571904146	0.060541599	0.1428195	no	down	mRNA	3.81	2.52	3.27	1.32	2.43	2.19	3.2	1.98	COG0252	E:Amino acid transport and metabolism	GO:0006520;GO:0004067	BP:cellular amino acid metabolic process;MF:asparaginase activity;
BAB_RS23345	-	cytochrome c1 family protein	0.927	-0.109033965	0.060752002	0.142896179	no	down	mRNA	623.12	618.94	654.22	526.6	571.22	564.95	632.093	554.256667	COG2857	C:Energy production and conversion	GO:0016021;GO:0005840;GO:0016021;GO:0009055;GO:0020037;GO:00	CC:integral component of membrane;CC:ribosome;CC:integral component of membrane;MF:electron carrier activity;MF:heme binding;MF:metal ion binding;MF:heme binding;MF:ubiquinol-cytochrome-c reductase activity;MF:metal ion binding;MF:electron carrier activity;
BAB_RS26880	-	ABC transporter permease	0.858	-0.220359065	0.061100479	0.143610708	no	down	mRNA	46.49	45.28	38.59	36.48	33.08	36.21	43.4533	35.2566667	COG1177	G:Carbohydrate transport and metabolism	GO:0055085;GO:0055085;GO:0016021;GO:0005886;GO:0016021;GO:00	BP:transmembrane transport;BP:transmembrane transport;CC:integral component of membrane;CC:plasma membrane;CC:integral component of membrane;CC:plasma membrane;
BAB_RS26740	-	aminodeoxychorismate synthase%2C component I	0.823	-0.281196532	0.061304484	0.143984874	no	down	mRNA	14.97	15.17	15.92	12.59	9.69	13.73	15.3533	12.0033333	COG0147	E:Amino acid transport and metabolism	GO:0009396;GO:0009396;GO:0009396;GO:0047904;GO:0016829;GO:00	BP:folic acid-containing compound biosynthetic process;BP:folic acid-containing compound biosynthetic process;BP:folic acid-containing compound biosynthetic process;MF:fructose 5-dehydrogenase activity;MF:lyase activity;MF:fructose 5-dehydrogenase activity;MF:lyase activity;
BAB_RS18635	-	sugar phosphate isomerase/epimerase	0.896	-0.159071942	0.061497809	0.144333426	no	down	mRNA	35.68	30.74	34.76	26.84	29.43	28.98	33.7267	28.4166667	COG1082	G:Carbohydrate transport and metabolism	------	------
BAB_RS19060	-	hypothetical protein	0.926	-0.110843783	0.061596063	0.144458504	no	down	mRNA	499.46	473.96	503.67	410.92	425.22	457.32	492.363	431.153333	COG0790	S:Function unknown	------	------
BAB_RS30470	-	ABC transporter permease	0.868	-0.204788926	0.062069472	0.14535657	no	down	mRNA	27.57	23.56	28.08	20.37	21.89	22.64	26.4033	21.6333333	COG1177	S:Function unknown	GO:0055085;GO:0055085;GO:0016021;GO:0005886;GO:0016021;GO:00	BP:transmembrane transport;BP:transmembrane transport;CC:integral component of membrane;CC:plasma membrane;CC:integral component of membrane;CC:plasma membrane;
BAB_RS23735	-	porin family protein	0.884	-0.177508866	0.062587978	0.146464075	no	down	mRNA	2236.68	1986.49	1975.18	1566.39	1875.84	1724.34	2066.12	1722.19	ENOG4111W66;ENOG4111WJN;COG3637	M:Cell wall/membrane/envelope biogenesis;	GO:0016021	CC:integral component of membrane;
BAB_RS22120	-	DUF882 domain-containing protein	0.902	-0.148568237	0.062792128	0.146728078	no	down	mRNA	51.78	50.38	54.13	41.21	43	48.76	52.0967	44.3233333	COG3108	S:Function unknown	------	------
BAB_RS33290	-	cobalamin biosynthesis protein CbiG	0.8	-0.321561542	0.063749734	0.148641434	no	down	mRNA	33.33	24.95	26.58	23.26	19.23	21.88	28.2867	21.4566667	------	------	------	------
BAB_RS23460	-	metallophosphoesterase	0.842	-0.248543452	0.064002955	0.149123636	no	down	mRNA	11.98	12.75	11.89	9.86	9.63	9.68	12.2067	9.72333333	COG1409	S:Function unknown	GO:0016787	MF:hydrolase activity;
BAB_RS25460	-	hypothetical protein	0.906	-0.141667378	0.064138247	0.149222439	no	down	mRNA	118.57	110.73	125.18	103.74	102.02	100.38	118.16	102.046667	------	------	GO:0016021	CC:integral component of membrane;
BAB_RS25725	-	DNA polymerase III subunit delta	0.888	-0.171972083	0.064204046	0.149267438	no	down	mRNA	28.25	26.9	26.71	22.53	23.56	22.39	27.2867	22.8266667	COG1466	L:Replication, recombination and repair	GO:0006260;GO:0006260;GO:0006260;GO:0009360;GO:0009360;GO:00	BP:DNA replication;BP:DNA replication;BP:DNA replication;CC:DNA polymerase III complex;CC:DNA polymerase III complex;CC:DNA polymerase III complex;MF:DNA-directed DNA polymerase activity;MF:DNA binding;MF:DNA binding;MF:DNA-directed DNA polymerase activity;MF:carbon-monoxide dehydrogenas;MF:DNA binding;MF:DNA-directed DNA polymerase activity;
BAB_RS25060	-	ArsR family transcriptional regulator	0.895	-0.160181049	0.064318389	0.14942515	no	down	mRNA	76.01	77.97	78.87	61.93	72.94	61.7	77.6167	65.5233333	COG0640	S:Function unknown	GO:0003700	MF:transcription factor activity, sequence-specific DNA binding;
BAB_RS30290	-	cytochrome P450	0.766	-0.383818454	0.064533226	0.149491894	no	down	mRNA	27.56	34.9	23.35	16.42	25.17	20.54	28.6033	20.71	COG2124	Q:Secondary metabolites biosynthesis, transport a	GO:0020037;GO:0005506;GO:0004497;GO:0016705;GO:0005506;GO:00	MF:heme binding;MF:iron ion binding;MF:monooxygenase activity;MF:oxidoreductase activity, acting on paired donors, with incorporation or reduction of molecular oxygen;MF:iron ion binding;MF:oxidoreductase activity, acting on paired donors, with incorporation or reduction of molecular oxygen;MF:monooxygenase activity;MF:heme binding;
BAB_RS16525	-	GNAT family N-acetyltransferase	0.893	-0.163357667	0.064863973	0.149933774	no	down	mRNA	186.61	193.08	174.15	145.76	159.56	167.87	184.613	157.73	COG2153	S:Function unknown	GO:0016740	MF:transferase activity;
BAB_RS22425	-	NAD(P)-dependent oxidoreductase	0.814	-0.297711689	0.066245506	0.152907191	no	down	mRNA	10.26	9.65	10.68	7.11	8.94	7.35	10.1967	7.8	ENOG410ZEX3	S:Function unknown	GO:0016491	MF:oxidoreductase activity;
BAB_RS25140	-	diaminopimelate epimerase	0.893	-0.163896123	0.066574449	0.153445987	no	down	mRNA	66.46	57.52	56.42	52.4	50.08	49.09	60.1333	50.5233333	COG0253	E:Amino acid transport and metabolism	GO:0009089;GO:0009089;GO:0005737;GO:0005737;GO:0008837;GO:00	BP:lysine biosynthetic process via diaminopimelate;BP:lysine biosynthetic process via diaminopimelate;CC:cytoplasm;CC:cytoplasm;MF:diaminopimelate epimerase activity;MF:diaminopimelate epimerase activity;
BAB_RS19520	-	ImmA/IrrE family metallo-endopeptidase	0.9	-0.152527781	0.066537369	0.153470615	no	down	mRNA	50.04	44.51	43.64	40.25	37.92	38.8	46.0633	38.99	ENOG4111PMA	S:Function unknown	GO:0009307;GO:0009307;GO:0009307;GO:0004519;GO:0003677;GO:00	BP:DNA restriction-modification system;BP:DNA restriction-modification system;BP:DNA restriction-modification system;MF:endonuclease activity;MF:DNA binding;MF:DNA binding;MF:endonuclease activity;MF:endonuclease activity;MF:DNA binding;
BAB_RS17290	-	DUF2948 domain-containing protein	0.906	-0.141669123	0.067142361	0.154422625	no	down	mRNA	176.08	147.36	164.15	129.62	140.56	147.72	162.53	139.3	ENOG4111TQ7	S:Function unknown	------	------
BAB_RS20115	-	2-dehydro-3-deoxygluconokinase	0.867	-0.205164151	0.067125619	0.154494708	no	down	mRNA	12.26	13.11	15.29	11.34	10.82	11.26	13.5533	11.14	COG0524	G:Carbohydrate transport and metabolism	GO:0016773;GO:0016301	MF:phosphotransferase activity, alcohol group as acceptor;MF:kinase activity;
BAB_RS28125	-	sugar-binding transcriptional regulator	0.851	-0.232195386	0.067381531	0.154861845	no	down	mRNA	21.04	17.36	23.61	15.13	17.33	17.3	20.67	16.5866667	COG2390	K:Transcription	GO:0030246;GO:0030246;GO:0016301	MF:carbohydrate binding;MF:carbohydrate binding;MF:kinase activity;
BAB_RS16630	-	class I SAM-dependent rRNA methyltransferase	0.922	-0.116903518	0.067928897	0.156008253	no	down	mRNA	89.01	86.32	95.64	75.44	82.21	77.96	90.3233	78.5366667	COG1092	S:Function unknown	GO:0006364;GO:0006364;GO:0005737;GO:0005737;GO:0008168;GO:00	BP:rRNA processing;BP:rRNA processing;CC:cytoplasm;CC:cytoplasm;MF:methyltransferase activity;MF:methyltransferase activity;
BAB_RS20195	-	iron-sulfur cluster insertion protein ErpA	0.916	-0.126950024	0.068970809	0.157612524	no	down	mRNA	92.91	78.97	87.57	73.13	77.39	73.03	86.4833	74.5166667	COG0316	S:Function unknown	GO:0097428;GO:0097428;GO:0051536;GO:0005198;GO:0051536;GO:00	BP:protein maturation by iron-sulfur cluster transfer;BP:protein maturation by iron-sulfur cluster transfer;MF:iron-sulfur cluster binding;MF:structural molecule activity;MF:iron-sulfur cluster binding;MF:structural molecule activity;
BAB_RS28845	-	myo-inosose-2 dehydratase	0.894	-0.161906721	0.070118632	0.160007929	no	down	mRNA	52.45	51.3	50.94	47.06	42.49	41.12	51.5633	43.5566667	COG1082	G:Carbohydrate transport and metabolism	GO:0016853	MF:isomerase activity;
BAB_RS18845	-	phage portal protein	0.671	-0.575426203	0.070184148	0.160043767	no	down	mRNA	1.84	1.77	1.76	0.79	1.44	1.12	1.79	1.11666667	COG4695	S:Function unknown	------	------
BAB_RS17800	-	cytochrome-c oxidase%2C cbb3-type subunit II	0.936	-0.095056895	0.070420433	0.160354963	no	down	mRNA	1395.22	1316.39	1392.86	1200.57	1203.82	1235.17	1368.16	1213.18667	COG2993	C:Energy production and conversion	GO:0009060;GO:0009060;GO:0016021;GO:0016021;GO:0004129;GO:00	BP:aerobic respiration;BP:aerobic respiration;CC:integral component of membrane;CC:integral component of membrane;MF:cytochrome-c oxidase activity;MF:hydrolase activity;MF:heme binding;MF:metal ion binding;MF:cytochrome-c oxidase activity;MF:heme binding;MF:metal ion binding;
BAB_RS24360	-	thiamine ABC transporter permease	0.863	-0.212164522	0.070473312	0.160361722	no	down	mRNA	9.03	7.76	9.59	7.34	6.78	7.31	8.79333	7.14333333	COG1178	P:Inorganic ion transport and metabolism	GO:0015888;GO:0055085;GO:0015888;GO:0055085;GO:0016021;GO:00	BP:thiamine transport;BP:transmembrane transport;BP:thiamine transport;BP:transmembrane transport;CC:integral component of membrane;CC:plasma membrane;CC:integral component of membrane;CC:plasma membrane;MF:transporter activity;MF:transporter activity;
BAB_RS16090	-	HIT family protein	0.898	-0.155296054	0.070793611	0.160635502	no	down	mRNA	316.6	345.74	359.65	282.28	309.72	291.18	340.663	294.393333	COG0537;COG0537	F:Nucleotide transport and metabolism;G:Carbohy	GO:0003824	MF:catalytic activity;
BAB_RS23100	-	DNA-binding protein	0.89	-0.167459116	0.070792501	0.160746505	no	down	mRNA	100.5	83.11	83.8	69.94	75.84	78.5	89.1367	74.76	COG3279	T:Signal transduction mechanisms	GO:0016021	CC:integral component of membrane;
BAB_RS21875	-	30S ribosomal protein S17	0.917	-0.124467002	0.070770251	0.160809629	no	down	mRNA	2301.12	1927.01	1857.36	1872.75	1838.56	1710.06	2028.5	1807.12333	COG0186	J:Translation, ribosomal structure and biogenesis	GO:0006412;GO:0006412;GO:0006412;GO:0006412;GO:0005840;GO:00	BP:translation;BP:translation;BP:translation;BP:translation;CC:ribosome;CC:ribosome;CC:ribosome;CC:ribosome;MF:rRNA binding;MF:structural constituent of ribosome;MF:structural constituent of ribosome;MF:rRNA binding;MF:structural constituent of ribosome;MF:rRNA binding;MF:structural constituent of ribosome;MF:rRNA binding;
BAB_RS22065	-	cold-shock protein	0.919	-0.122212052	0.071526523	0.161955404	no	down	mRNA	253.55	242.58	272.87	201.87	229.68	234.84	256.333	222.13	COG1278	S:Function unknown	GO:0006355;GO:0003677	BP:regulation of transcription, DNA-templated;MF:DNA binding;
BAB_RS17460	-	long-chain-fatty-acid--CoA ligase	0.906	-0.142234357	0.072637935	0.164356116	no	down	mRNA	32.46	28.31	30.79	24.2	26.13	27.63	30.52	25.9866667	COG0318;COG0318	I:Lipid transport and metabolism;Q:Secondary met	GO:0008152;GO:0008152;GO:0008152;GO:0016021;GO:0016021;GO:00	BP:metabolic process;BP:metabolic process;BP:metabolic process;CC:integral component of membrane;CC:integral component of membrane;MF:catalytic activity;MF:ligase activity;MF:catalytic activity;MF:catalytic activity;
BAB_RS23455	-	NUDIX domain-containing protein	0.884	-0.177753746	0.072740147	0.164471563	no	down	mRNA	103.98	95.33	130.27	86.18	100.01	87.73	109.86	91.3066667	COG0494	M:Cell wall/membrane/envelope biogenesis	GO:0016787	MF:hydrolase activity;
BAB_RS31360	-	amidase	0.868	-0.204812963	0.074674712	0.168371825	no	down	mRNA	11.46	11.49	13.85	9.9	10.71	9.51	12.2667	10.04	COG0154	S:Function unknown	GO:0004040	MF:amidase activity;
BAB_RS27470	-	glutathione S-transferase	0.867	-0.20518764	0.075105477	0.169224332	no	down	mRNA	23.9	24.48	24.07	20.01	20.86	18.59	24.15	19.82	COG0625	S:Function unknown	GO:0016740;GO:0016740;GO:0004364	MF:transferase activity;MF:transferase activity;MF:glutathione transferase activity;
BAB_RS33325	-	quinone oxidoreductase	0.805	-0.31238494	0.075286883	0.169514193	no	down	mRNA	741.87	643.97	693.18	508.81	642.79	571.6	693.007	574.4	COG0604	C:Energy production and conversion	GO:0008270;GO:0016491	MF:zinc ion binding;MF:oxidoreductase activity;
BAB_RS16030	-	acyltransferase	0.832	-0.265643089	0.075534589	0.169952826	no	down	mRNA	18.46	21.3	18.23	13.45	15.63	16.74	19.33	15.2733333	COG1835	I:Lipid transport and metabolism	GO:0016021;GO:0016021;GO:0016747;GO:0016747	CC:integral component of membrane;CC:integral component of membrane;MF:transferase activity, transferring acyl groups other than amino-acyl groups;MF:transferase activity, transferring acyl groups other than amino-acyl groups;
BAB_RS17760	-	guanine deaminase	0.9	-0.151839964	0.075912842	0.170565007	no	down	mRNA	26.49	26.08	25.93	21.25	23.27	22.04	26.1667	22.1866667	COG0402	F:Nucleotide transport and metabolism	GO:0006147;GO:0008270;GO:0008892	BP:guanine catabolic process;MF:zinc ion binding;MF:guanine deaminase activity;
BAB_RS22630	-	STAS/SEC14 domain-containing protein	0.922	-0.116966855	0.075904707	0.170666078	no	down	mRNA	276.04	247.64	255.73	232.71	219.01	230.58	259.803	227.433333	ENOG41123N7	S:Function unknown	------	------
BAB_RS17570	-	DUF952 domain-containing protein	0.768	-0.38068124	0.07617513	0.170915288	no	down	mRNA	16.92	22.99	16.31	14.08	13.5	13.95	18.74	13.8433333	COG3502	S:Function unknown	------	------
BAB_RS30885	-	aminobutyraldehyde dehydrogenase	0.898	-0.155064708	0.076360763	0.171092839	no	down	mRNA	22.22	18.01	21.89	17.95	16.67	17.74	20.7067	17.4533333	COG0579	C:Energy production and conversion	GO:0016491	MF:oxidoreductase activity;
BAB_RS31695	-	homoserine kinase	0.875	-0.191973822	0.076534502	0.171243284	no	down	mRNA	12.54	12.46	13.31	11.03	10.06	10.61	12.77	10.5666667	COG2334	Q:Secondary metabolites biosynthesis, transport a	GO:0009088;GO:0016740;GO:0016301;GO:0004413	BP:threonine biosynthetic process;MF:transferase activity;MF:kinase activity;MF:homoserine kinase activity;
BAB_RS16855	-	glycerol-3-phosphate dehydrogenase	0.86	-0.216993465	0.07658826	0.171244314	no	down	mRNA	27.87	22.69	22.75	19.36	22.1	17.5	24.4367	19.6533333	COG0578	C:Energy production and conversion	GO:0006072;GO:0006072;GO:0009331;GO:0009331;GO:0052591;GO:00	BP:glycerol-3-phosphate metabolic process;BP:glycerol-3-phosphate metabolic process;CC:glycerol-3-phosphate dehydrogenase complex;CC:glycerol-3-phosphate dehydrogenase complex;MF:sn-glycerol-3-phosphat;MF:sn-glycerol-3-phosphat;
BAB_RS26240	-	hypothetical protein	0.909	-0.137964456	0.077214721	0.172046392	no	down	mRNA	246.97	246.25	308.96	225.37	235.29	227.24	267.393	229.3	COG2740	K:Transcription	------	------
BAB_RS17330	-	XRE family transcriptional regulator	0.795	-0.331540608	0.077187159	0.172104332	no	down	mRNA	22.5	19.34	21.27	12.74	16.27	18.78	21.0367	15.93	COG1396;ENOG41125NN	S:Function unknown;	GO:0043565	MF:sequence-specific DNA binding;
BAB_RS21890	-	30S ribosomal protein S3	0.927	-0.109091404	0.077835762	0.17318996	no	down	mRNA	1104.11	1053.12	1159.47	926.37	1040.5	944.14	1105.57	970.336667	COG0092	J:Translation, ribosomal structure and biogenesis	GO:0006412;GO:0006412;GO:0006412;GO:0015935;GO:0015935;GO:00	BP:translation;BP:translation;BP:translation;CC:small ribosomal subunit;CC:small ribosomal subunit;CC:small ribosomal subunit;MF:structural constituent of ribosome;MF:rRNA binding;MF:mRNA binding;MF:rRNA binding;MF:structural constituent of ribosome;MF:structural constituent of ribosome;MF:rRNA binding;MF:mRNA binding;
BAB_RS20110	-	galactose mutarotase	0.926	-0.110201091	0.078116517	0.173214886	no	down	mRNA	106.96	97.19	101.45	87.49	89.62	89.63	101.867	88.9133333	COG2017	G:Carbohydrate transport and metabolism	GO:0019318;GO:0019318;GO:0004034;GO:0030246;GO:0030246;GO:00	BP:hexose metabolic process;BP:hexose metabolic process;MF:aldose 1-epimerase activity;MF:carbohydrate binding;MF:carbohydrate binding;MF:aldose 1-epimerase activity;
BAB_RS25645	-	GFA family protein	0.727	-0.460371076	0.0791899	0.175232197	no	down	mRNA	13.44	16.42	17.11	14.44	9.9	8.9	15.6567	11.08	COG3791	S:Function unknown	GO:0008152;GO:0016846	BP:metabolic process;MF:carbon-sulfur lyase activity;
BAB_RS24735	-	hypothetical protein	0.861	-0.216681363	0.079495123	0.175786532	no	down	mRNA	30.94	26.92	35.1	26.61	22.52	26.76	30.9867	25.2966667	ENOG41126HB;COG5295	S:Function unknown;	------	------
BAB_RS30385	-	peptidase S58 family protein	0.922	-0.117427547	0.080064018	0.17680116	no	down	mRNA	51.54	47.95	51.09	43.96	43.65	43.22	50.1933	43.61	COG3191	P:Inorganic ion transport and metabolism	------	------
BAB_RS24935	-	hydroxymethylbilane synthase	0.917	-0.125175292	0.080239736	0.177067494	no	down	mRNA	79.42	77.1	75.72	68.08	66.03	67.01	77.4133	67.04	COG0181	H:Coenzyme transport and metabolism	GO:0018160;GO:0006782;GO:0006782;GO:0018160;GO:0004418;GO:00	BP:peptidyl-pyrromethane cofactor linkage;BP:protoporphyrinogen IX biosynthetic process;BP:protoporphyrinogen IX biosynthetic process;BP:peptidyl-pyrromethane cofactor linkage;MF:hydroxymethylbilane synthase activity;MF:hydroxymethylbilane synthase activity;
BAB_RS18340	-	N-acetyltransferase	0.84	-0.250813479	0.080775238	0.177882686	no	down	mRNA	37.62	26.37	34.94	25.98	23.82	28.66	32.9767	26.1533333	COG1670	J:Translation, ribosomal structure and biogenesis	GO:0016740;GO:0008999;GO:0016740	MF:transferase activity;MF:ribosomal-protein-alanine N-acetyltransferase activity;MF:transferase activity;
BAB_RS23815	-	-	0.561	-0.833136744	0.081054794	0.178376064	no	down	mRNA	3.7	13.59	4.48	4.47	3.16	4.33	7.25667	3.98666667	ENOG41129MV;ENOG4111P6Q;ENOG41122GY;ENOG4111PC4	S:Function unknown;	------	------
BAB_RS26590	-	alpha/beta hydrolase	0.903	-0.147227482	0.081769644	0.179703055	no	down	mRNA	20.4	20.26	24.95	18.13	18.86	18.87	21.87	18.62	COG2267	E:Amino acid transport and metabolism	GO:0004622;GO:0004622;GO:0016787	MF:lysophospholipase activity;MF:lysophospholipase activity;MF:hydrolase activity;
BAB_RS19955	-	lipoprotein-releasing system ATP-binding protein LolD	0.906	-0.143101556	0.082098432	0.180302299	no	down	mRNA	88.43	94.04	100.97	74.82	85.24	83.17	94.48	81.0766667	COG1136	V:Defense mechanisms	GO:0005886;GO:0005886;GO:0005524;GO:0016887;GO:0042954;GO:00	CC:plasma membrane;CC:plasma membrane;MF:ATP binding;MF:ATPase activity;MF:lipoprotein transporter activity;MF:lipoprotein transporter activity;MF:ATPase activity;MF:ATP binding;
BAB_RS29320	-	two-component system response regulator	0.906	-0.14269586	0.082373189	0.180412444	no	down	mRNA	332.46	290.41	294.79	240.05	266.72	289.36	305.887	265.376667	ENOG4111I86	T:Signal transduction mechanisms	GO:0000160;GO:0006355;GO:0006351;GO:0000160;GO:0006355;GO:00	BP:phosphorelay signal transduction system;BP:regulation of transcription, DNA-templated;BP:transcription, DNA-templated;BP:phosphorelay signal transduction system;BP:regulation of transcription, DNA-templated;BP:transcription, DNA-templated;BP:phosphorelay signal transduction system;CC:cytoplasm;CC:cytoplasm;CC:intracellular;CC:intracellular;MF:DNA binding;MF:DNA binding;
BAB_RS25575	-	BolA family transcriptional regulator	0.901	-0.15003467	0.082330713	0.180442415	no	down	mRNA	74.42	62.11	72.85	63.53	58.77	55.56	69.7933	59.2866667	COG0271	H:Coenzyme transport and metabolism	------	------
BAB_RS18200	-	50S ribosomal protein L9	0.889	-0.170379818	0.082226799	0.180460865	no	down	mRNA	481.35	398.44	431.22	351.12	421.75	331.12	437.003	367.996667	COG0359	J:Translation, ribosomal structure and biogenesis	GO:0006412;GO:0006412;GO:0006412;GO:0006412;GO:0005840;GO:00	BP:translation;BP:translation;BP:translation;BP:translation;CC:ribosome;CC:ribosome;CC:ribosome;CC:ribosome;MF:rRNA binding;MF:structural constituent of ribosome;MF:structural constituent of ribosome;MF:rRNA binding;MF:structural constituent of ribosome;MF:rRNA binding;MF:structural constituent of ribosome;MF:rRNA binding;
BAB_RS28470	-	hypothetical protein	0.592	-0.756244375	0.082301858	0.1805023	no	down	mRNA	117.55	132.89	166.56	89.58	86.18	101.5	139	92.42	------	------	------	------
BAB_RS32970	-	hypothetical protein	0.617	-0.697670928	0.082625289	0.180718212	no	down	mRNA	20.24	19.45	16.86	6.77	11.77	16.09	18.85	11.5433333	------	------	------	------
BAB_RS21900	-	30S ribosomal protein S19	0.895	-0.160594549	0.083299143	0.181820751	no	down	mRNA	1230.7	956.15	1019.58	916.48	965.5	951.84	1068.81	944.606667	COG0185	J:Translation, ribosomal structure and biogenesis	GO:0006412;GO:0006412;GO:0006412;GO:0006412;GO:0015935;GO:00	BP:translation;BP:translation;BP:translation;BP:translation;CC:small ribosomal subunit;CC:small ribosomal subunit;CC:small ribosomal subunit;CC:small ribosomal subunit;MF:rRNA binding;MF:structural constituent of ribosome;MF:structural constituent of ribosome;MF:rRNA binding;MF:structural constituent of ribosome;MF:rRNA binding;MF:structural constituent of ribosome;MF:rRNA binding;
BAB_RS26060	-	gfo/Idh/MocA family oxidoreductase	0.905	-0.144768276	0.083550198	0.181874517	no	down	mRNA	59.64	50.62	53.35	46.45	48.68	43.99	54.5367	46.3733333	COG0673	J:Translation, ribosomal structure and biogenesis	GO:0016021	CC:integral component of membrane;
BAB_RS24070	-	tRNA(Ile)-lysidine synthetase	0.912	-0.132496898	0.083618557	0.181900083	no	down	mRNA	50.82	51.06	60.78	46.5	48.48	44.93	54.22	46.6366667	COG0037	O:Posttranslational modification, protein turnover,	GO:0006400;GO:0008033;GO:0006400;GO:0006400;GO:0005737;GO:00	BP:tRNA modification;BP:tRNA processing;BP:tRNA modification;BP:tRNA modification;CC:cytoplasm;CC:cytoplasm;CC:cytoplasm;MF:ligase activity, forming carbon-nitrogen bonds;MF:ATP binding;MF:ATP binding;MF:isomerase activity;MF:ligase activity, forming carbon-nitrogen bonds;MF:ATP binding;MF:ligase activity, forming carbon-nitrogen bonds;
BAB_RS20105	-	hypothetical protein	0.775	-0.367831507	0.083282519	0.181908044	no	down	mRNA	11.26	7.38	7.78	5.37	6.36	7.26	8.80667	6.33	COG3926	S:Function unknown	GO:0016021	CC:integral component of membrane;
BAB_RS17995	-	type II toxin-antitoxin system RelE/ParE family toxin	0.882	-0.181512173	0.083992128	0.18234237	no	down	mRNA	36.64	30.73	33.59	30.41	26.37	27.08	33.6533	27.9533333	COG3668	S:Function unknown	------	------
BAB_RS17885	-	ATP synthase subunit	0.863	-0.212620139	0.084178559	0.182377417	no	down	mRNA	101.82	107.98	132.05	104.29	93.01	85.21	113.95	94.17	COG5336	S:Function unknown	GO:0006811;GO:0006811;GO:0016021;GO:0045263;GO:0045263;GO:00	BP:ion transport;BP:ion transport;CC:integral component of membrane;CC:proton-transporting ATP synthase complex, coupling factor;CC:proton-transporting ATP synthase complex, coupling factor;CC:integral component of membrane;
BAB_RS28745	-	binding-protein-dependent transport system inner membrane component	0.882	-0.181051185	0.084176125	0.182495201	no	down	mRNA	24.02	19.62	23.8	18.19	18.3	19.52	22.48	18.67	COG4176	S:Function unknown	GO:0055085;GO:0055085;GO:0016021;GO:0005886;GO:0016021;GO:00	BP:transmembrane transport;BP:transmembrane transport;CC:integral component of membrane;CC:plasma membrane;CC:integral component of membrane;CC:plasma membrane;
BAB_RS31655	-	aldehyde dehydrogenase	0.865	-0.209363329	0.084436603	0.182567165	no	down	mRNA	12.19	10.39	12.63	10.31	8.63	9.75	11.7367	9.56333333	COG1012	S:Function unknown	GO:0016620	MF:oxidoreductase activity, acting on the aldehyde or oxo group of donors, NAD or NADP as acceptor;
BAB_RS31740	-	branched-chain amino acid ABC transporter substrate-binding protein	0.711	-0.491240821	0.084949486	0.183183019	no	down	mRNA	1.64	1.48	1.64	1	1.12	1.06	1.58667	1.06	ENOG410XTBH;ENOG4110NBE	E:Amino acid transport and metabolism;	------	------
BAB_RS26515	-	NAD(P)-dependent oxidoreductase	0.592	-0.755757674	0.0855371	0.184202884	no	down	mRNA	7.76	2.89	5.23	1.28	3.96	3.18	5.29333	2.80666667	ENOG410YI7A	S:Function unknown	GO:0000166;GO:0016491;GO:0016491	MF:nucleotide binding;MF:oxidoreductase activity;MF:oxidoreductase activity;
BAB_RS22150	-	JAB domain-containing protein	0.833	-0.263396798	0.08667955	0.186288557	no	down	mRNA	12.76	14.83	16.87	11.51	12.28	11.6	14.82	11.7966667	COG2003	L:Replication, recombination and repair	GO:0046872;GO:0008237	MF:metal ion binding;MF:metallopeptidase activity;
BAB_RS24180	-	YebC/PmpR family DNA-binding transcriptional regulator	0.898	-0.154958864	0.087342489	0.187087612	no	down	mRNA	343.75	294.81	290.97	288.38	250.18	250.15	309.843	262.903333	COG0217	K:Transcription	GO:0006355;GO:0006351;GO:0006355;GO:0006351;GO:0005737;GO:00	BP:regulation of transcription, DNA-templated;BP:transcription, DNA-templated;BP:regulation of transcription, DNA-templated;BP:transcription, DNA-templated;CC:cytoplasm;CC:cytoplasm;MF:DNA binding;MF:DNA binding;
BAB_RS28140	-	carbohydrate kinase	0.913	-0.131497035	0.088053493	0.188108958	no	down	mRNA	30.57	31.54	34.28	28.29	28.53	26.16	32.13	27.66	COG1070	G:Carbohydrate transport and metabolism	GO:0005975;GO:0005975;GO:0005975;GO:0016310;GO:0016301;GO:00	BP:carbohydrate metabolic process;BP:carbohydrate metabolic process;BP:carbohydrate metabolic process;BP:phosphorylation;MF:kinase activity;MF:phosphotransferase activity, alcohol group as acceptor;MF:phosphotransferase activity, alcohol group as acceptor;MF:erythritol kinase activity;MF:kinase activity;MF:ATP binding;MF:erythritol kinase activity;
BAB_RS28055	-	3-isopropylmalate dehydratase small subunit	0.884	-0.17842688	0.087948615	0.188135086	no	down	mRNA	232.42	185.98	183.54	183.24	160.43	158.85	200.647	167.506667	COG0066	E:Amino acid transport and metabolism	GO:0009098;GO:0009098;GO:0009098;GO:0009316;GO:0009316;GO:00	BP:leucine biosynthetic process;BP:leucine biosynthetic process;BP:leucine biosynthetic process;CC:3-isopropylmalate dehydratase complex;CC:3-isopropylmalate dehydratase complex;CC:3-isopropylmalate dehydratase complex;MF:3-isopropylmalate dehydratase activity;MF:isomerase activity;MF:3-isopropylmalate dehydratase activity;MF:isomerase activity;MF:3-isopropylmalate dehydratase activity;
BAB_RS19370	-	short chain dehydrogenase	0.906	-0.142148327	0.088015753	0.188153435	no	down	mRNA	49.86	41.34	45.89	40.33	39.17	37.27	45.6967	38.9233333	COG1028	L:Replication, recombination and repair	------	------
BAB_RS16440	-	sulfate/thiosulfate import ATP-binding protein CysA	0.922	-0.117793832	0.08815771	0.188206461	no	down	mRNA	123.75	115.9	120.26	96.97	107.37	108.71	119.97	104.35	COG1118	P:Inorganic ion transport and metabolism	GO:0102025;GO:0008152;GO:0043190;GO:0043190;GO:0015419;GO:00	old GO;BP:metabolic process;CC:ATP-binding cassett;CC:ATP-binding cassett;MF:ATPase-coupled sulfate transmembrane transporter activity;MF:hydrolase activity, acting on glycosyl bonds;MF:ATP binding;MF:ATP binding;MF:ATPase-coupled sulfate transmembrane transporter activity;MF:old GO;
BAB_RS23275	-	prolipoprotein diacylglyceryl transferase	0.919	-0.12142357	0.088297593	0.188254921	no	down	mRNA	81.97	69.41	81.39	67.38	66.4	67.99	77.59	67.2566667	COG0682	M:Cell wall/membrane/envelope biogenesis	GO:0042158;GO:0042158;GO:0016021;GO:0005886;GO:0016021;GO:00	BP:lipoprotein biosynthetic process;BP:lipoprotein biosynthetic process;CC:integral component of membrane;CC:plasma membrane;CC:integral component of membrane;CC:plasma membrane;MF:phosphatidylglycerol-prolipoprotein diacylglyceryl transferase activity;MF:hydrolase activity;MF:hydrolase activity;MF:phosphatidylglycerol-prolipoprotein diacylglyceryl transferase activity;MF:transferase activity, transferring glycosyl groups;
BAB_RS30035	-	GntR family transcriptional regulator	0.901	-0.15004391	0.090056049	0.19174956	no	down	mRNA	40	31.36	36.43	28.37	30.26	32.48	35.93	30.37	COG1802	V:Defense mechanisms	GO:0006351;GO:0006351;GO:0003700;GO:0003677;GO:0003677;GO:00	BP:transcription, DNA-templated;BP:transcription, DNA-templated;MF:transcription factor activity, sequence-specific DNA binding;MF:DNA binding;MF:DNA binding;MF:transcription factor activity, sequence-specific DNA binding;
BAB_RS32235	-	hypothetical protein	0.757	-0.401324507	0.090225738	0.19198364	no	down	mRNA	98.89	141.1	155.57	88.83	120.08	97.29	131.853	102.066667	COG1561	S:Function unknown	------	------
BAB_RS30045	-	membrane protein	0.8	-0.322827731	0.090391093	0.192081073	no	down	mRNA	30.41	36	28.8	25.91	20.79	27.1	31.7367	24.6	COG2391	S:Function unknown	GO:0016021	CC:integral component of membrane;
BAB_RS23915	-	DUF1328 domain-containing protein	0.875	-0.193136369	0.090462486	0.192105729	no	down	mRNA	16564.6	16430.1	14293.3	12602.45	14306.78	14976.22	15762.7	13961.8167	------	------	------	------
BAB_RS32205	-	hypothetical protein	0.904	-0.145281507	0.090610466	0.192292885	no	down	mRNA	131.21	111.97	157.93	114.07	115.13	110.93	133.703	113.376667	------	------	------	------
BAB_RS16145	-	outer membrane protein assembly factor	0.926	-0.110967022	0.09141021	0.193606462	no	down	mRNA	328.45	304.84	322.91	273.73	262.25	297.68	318.733	277.886667	COG0729	M:Cell wall/membrane/envelope biogenesis	GO:0019867	CC:outer membrane;
BAB_RS24650	-	carbonic anhydrase	0.939	-0.091120362	0.091864428	0.193929309	no	down	mRNA	464.16	449.23	458.74	400.74	414.88	406.08	457.377	407.233333	COG0288	P:Inorganic ion transport and metabolism	GO:0015976;GO:0004089;GO:0008270	BP:carbon utilization;MF:carbonate dehydratase activity;MF:zinc ion binding;
BAB_RS32090	-	hypothetical protein	0.627	-0.674167345	0.091847424	0.194020891	no	down	mRNA	3.91	2.77	4.58	1.64	2.77	2.25	3.75333	2.22	------	------	------	------
BAB_RS18555	-	IS5/IS1182 family transposase	0.155	-2.686459044	0.091825108	0.194101363	no	down	mRNA	0	0	14.9	2.63	0	0.02	4.96667	0.88333333	COG3293	L:Replication, recombination and repair	GO:0006313;GO:0016021;GO:0003677;GO:0004803	BP:transposition, DNA-mediated;CC:integral component of membrane;MF:DNA binding;MF:transposase activity;
BAB_RS16520	-	hypothetical protein	0.902	-0.148134812	0.091767447	0.19410718	no	down	mRNA	205.27	170.35	165.45	156.28	151.95	153.36	180.357	153.863333	COG4291	S:Function unknown	GO:0016021	CC:integral component of membrane;
BAB_RS22050	-	serine O-acetyltransferase	0.933	-0.10057087	0.0924454	0.194644213	no	down	mRNA	141.66	138.98	142.8	121.92	122.97	127.89	141.147	124.26	COG1045	E:Amino acid transport and metabolism	GO:0006535;GO:0006535;GO:0006535;GO:0005737;GO:0005737;GO:00	BP:cysteine biosynthetic process from serine;BP:cysteine biosynthetic process from serine;BP:cysteine biosynthetic process from serine;CC:cytoplasm;CC:cytoplasm;CC:cytoplasm;MF:N-succinylarginine dihydrolase activity;MF:serine O-acetyltransferase activity;MF:N-succinylarginine dihydrolase activity;MF:serine O-acetyltransferase activity;MF:serine O-acetyltransferase activity;
BAB_RS22585	-	hypothetical protein	0.895	-0.159928799	0.09229422	0.194708686	no	down	mRNA	60.11	69.43	73.6	57.33	56.19	59.57	67.7133	57.6966667	COG0494;ENOG41122ER	S:Function unknown;	GO:0016787	MF:hydrolase activity;
BAB_RS31045	-	GIY-YIG nuclease family protein	0.776	-0.364952658	0.09263215	0.194782131	no	down	mRNA	22.55	15.41	23.66	13.73	18.22	13.17	20.54	15.04	COG2827	L:Replication, recombination and repair	GO:0004519	MF:endonuclease activity;
BAB_RS16720	hrcA	HrcA family transcriptional regulator	0.916	-0.126790687	0.092849441	0.195111349	no	down	mRNA	62.9	52.44	57.97	49.71	50.84	48.68	57.77	49.7433333	COG1420	K:Transcription	GO:0006355;GO:0006351;GO:0006355;GO:0045892;GO:0006351;GO:00	BP:regulation of transcription, DNA-templated;BP:transcription, DNA-templated;BP:regulation of transcription, DNA-templated;BP:negative regulation of transcription, DNA-templated;BP:transcription, DNA-templated;MF:DNA binding;MF:DNA binding;
BAB_RS25835	-	DUF1402 domain-containing protein	0.855	-0.226483692	0.09338184	0.196101863	no	down	mRNA	30.11	24.3	25.36	23.51	22.75	17.52	26.59	21.26	ENOG410XST5	S:Function unknown	------	------
BAB_RS25340	-	7-carboxy-7-deazaguanine synthase QueE	0.829	-0.271108442	0.093541098	0.196179862	no	down	mRNA	10.57	13.54	13.16	10.02	10.15	9.3	12.4233	9.82333333	COG0602	O:Posttranslational modification, protein turnover,	GO:0008616;GO:0016840;GO:0000287;GO:0051539;GO:1904047	BP:queuosine biosynthetic process;MF:carbon-nitrogen lyase activity;MF:magnesium ion binding;MF:4 iron, 4 sulfur cluster binding;MF:S-adenosyl-L-methionine binding;
BAB_RS16820	-	sulfate adenylyltransferase subunit CysD	0.806	-0.311592444	0.093488977	0.196198618	no	down	mRNA	8.7	6.94	8.74	5.55	5.59	7.44	8.12667	6.19333333	COG0175	E:Amino acid transport and metabolism	GO:0070814;GO:0000103;GO:0019419;GO:0070814;GO:0000103;GO:00	BP:hydrogen sulfide biosynthetic process;BP:sulfate assimilation;BP:sulfate reduction;BP:hydrogen sulfide biosynthetic process;BP:sulfate assimilation;BP:sulfate reduction;MF:ATP binding;MF:sulfate adenylyltransferas;MF:ATP binding;MF:oxidoreductase activity;MF:sulfate adenylyltransferas;
BAB_RS32330	-	cyanate transporter	0.784	-0.350918034	0.093818141	0.196376343	no	down	mRNA	3.37	3.75	5.19	3.26	3.2	2.75	4.10333	3.07	COG2807	G:Carbohydrate transport and metabolism	GO:0055085;GO:0016021;GO:0016021	BP:transmembrane transport;CC:integral component of membrane;CC:integral component of membrane;
BAB_RS24700	-	hypothetical protein	0.905	-0.143329332	0.093811613	0.196490686	no	down	mRNA	136.87	109.34	116.18	104.59	105.5	98.62	120.797	102.903333	ENOG4111KJK	S:Function unknown	------	------
BAB_RS20955	-	dihydroneopterin aldolase	0.824	-0.279394189	0.094158841	0.19696117	no	down	mRNA	79.81	65.01	69.15	61.28	62.68	44.94	71.3233	56.3	COG1539	H:Coenzyme transport and metabolism	GO:0046656;GO:0046654;GO:0046656;GO:0046654;GO:0046654;GO:00	BP:folic acid biosynthetic process;BP:tetrahydrofolate biosynthetic process;BP:folic acid biosynthetic process;BP:tetrahydrofolate biosynthetic process;BP:tetrahydrofolate biosynthetic process;BP:folic acid biosynthetic process;MF:dihydroneopterin aldolase activity;MF:hydrolase activity;MF:dihydroneopterin aldolase activity;MF:dihydroneopterin aldolase activity;MF:hydrolase activity;
BAB_RS22580	-	PhzF family phenazine biosynthesis protein	0.909	-0.138373086	0.094519755	0.19758749	no	down	mRNA	101.34	106.02	108.97	81.7	98.1	91.78	105.443	90.5266667	COG0384	S:Function unknown	GO:0009058;GO:0009058;GO:0003824;GO:0016853;GO:0003824	BP:biosynthetic process;BP:biosynthetic process;MF:catalytic activity;MF:isomerase activity;MF:catalytic activity;
BAB_RS24995	-	VOC family protein	0.927	-0.109390978	0.095345561	0.199184193	no	down	mRNA	65.29	64.24	77.12	60.13	60.54	59.54	68.8833	60.07	COG0346	I:Lipid transport and metabolism	GO:0016829;GO:0016829;GO:0004462;GO:0051213;GO:0016829;GO:00	MF:lyase activity;MF:lyase activity;MF:lactoylglutathione lyase activity;MF:dioxygenase activity;MF:lyase activity;MF:metal ion binding;MF:dioxygenase activity;MF:lactoylglutathione lyase activity;
BAB_RS30855	-	oxidoreductase	0.915	-0.127759247	0.095413146	0.199195866	no	down	mRNA	31.91	30.42	34.23	28.07	27.45	27.91	32.1867	27.81	COG2055	C:Energy production and conversion	GO:0030060;GO:0016491	MF:L-malate dehydrogenase activity;MF:oxidoreductase activity;
BAB_RS27190	-	FAD-binding oxidoreductase	0.917	-0.125344628	0.095742819	0.19962471	no	down	mRNA	27.84	28.64	30.5	24.65	24.33	26.29	28.9933	25.09	COG0277	S:Function unknown	GO:0052852;GO:0052854;GO:0050660;GO:0016614;GO:0052853;GO:00	MF:very-long-chain;MF:medium-chain;MF:flavin adenine dinucleotide binding;MF:oxidoreductase activity, acting on CH-OH group of donors;MF:long-chain;MF:flavin adenine dinucleotide binding;MF:long-chain;MF:medium-chain;MF:very-long-chain;MF:flavin adenine dinucleotide binding;MF:oxidoreductase activity, acting on CH-OH group of donors;
BAB_RS28650	-	MarR family transcriptional regulator	0.878	-0.188485369	0.096634217	0.201352619	no	down	mRNA	175.16	152.13	160.31	158.48	128.06	120.28	162.533	135.606667	ENOG41122J0;COG1846	K:Transcription;	GO:0006351;GO:0006351;GO:0003700;GO:0003677;GO:0003677;GO:00	BP:transcription, DNA-templated;BP:transcription, DNA-templated;MF:transcription factor activity, sequence-specific DNA binding;MF:DNA binding;MF:DNA binding;MF:transcription factor activity, sequence-specific DNA binding;
BAB_RS23005	-	linear amide C-N hydrolase	0.907	-0.140062764	0.09803823	0.203881445	no	down	mRNA	514.51	476.34	472.09	382.92	404.76	465.48	487.647	417.72	COG3049	M:Cell wall/membrane/envelope biogenesis	GO:0016021;GO:0016787;GO:0045302;GO:0016787	CC:integral component of membrane;MF:hydrolase activity;MF:choloylglycine hydrolase activity;MF:hydrolase activity;
BAB_RS21605	-	RidA family protein	0.925	-0.111887864	0.09897677	0.205169265	no	down	mRNA	370.49	346.37	329.83	301.66	302.94	315.95	348.897	306.85	COG0251	J:Translation, ribosomal structure and biogenesis	------	------
BAB_RS32990	-	hypothetical protein	0.61	-0.713210164	0.098819434	0.205240364	no	down	mRNA	4.11	11.08	7.19	5.25	4.12	4.35	7.46	4.57333333	------	------	------	------

BAB_RS21820	-	preprotein translocase subunit SecY	0.921	-0.118516615	0.098966688	0.205280805	no	down	mRNA	339.62	281.34	298.13	270.03	262.91	264.25	306.363	265.73	COG0201	U:Intracellular trafficking, secretion, and vesicular t	GO:0006605;GO:0065002;GO:0043952;GO:0065002;GO:0006605;GO:00	BP:protein targeting;BP:intracellular protein transmembrane transport;BP:protein transport by the Sec complex;BP:intracellular protein transmembrane transport;BP:protein targeting;BP:protein transport by the Sec complex;CC:intracellular;CC:integral component of membrane;CC:plasma membrane;CC:integral component of membrane;CC:intracellular;CC:plasma membrane;MF:methyltransferase activity;
BAB_RS22250	-	precorrin-3B C(17)-methyltransferase	0.799	-0.323806205	0.099098825	0.205289828	no	down	mRNA	39.36	49.29	32.42	33.05	29.11	30.95	40.3567	31.0366667	COG2073	H:Coenzyme transport and metabolism	GO:0009236;GO:0009236;GO:0009236;GO:0008168;GO:0008168;GO:00	BP:cobalamin biosynthetic process;BP:cobalamin biosynthetic process;BP:cobalamin biosynthetic process;MF:methyltransferase activity;MF:methyltransferase activity;MF:precorrin-3B C17-methyltransferase activity;
BAB_RS19130	-	SsrA-binding protein	0.885	-0.175996844	0.099421287	0.205560229	no	down	mRNA	37.09	37.79	36.12	34.4	31.06	27.24	37	30.9	COG0691	O:Posttranslational modification, protein turnover,	GO:0070929;GO:0070929;GO:0005737;GO:0005737;GO:0003723;GO:00	BP:trans-translation;BP:trans-translation;CC:cytoplasm;CC:cytoplasm;MF:RNA binding;MF:RNA binding;
BAB_RS31910	-	hypothetical protein	0.754	-0.406576673	0.099333574	0.205643539	no	down	mRNA	12.46	10.85	10.39	7.9	6.13	10.27	11.2333	8.1	------		------	------
BAB_RS23700	-	hypothetical protein	0.899	-0.153181226	0.099407852	0.205664796	no	down	mRNA	110.75	111.07	129.65	92.4	109.26	99.18	117.157	100.28	ENOG4111XJE;ENOG410YQFA	S:Function unknown;	------	------
BAB_RS32485	-	hypothetical protein	0.858	-0.220161856	0.099664723	0.205798685	no	down	mRNA	61.81	49.47	67.89	46.87	49.96	50.58	59.7233	49.1366667	------		------	------
BAB_RS25100	-	membrane protein	0.877	-0.190028006	0.099928029	0.206209863	no	down	mRNA	238.49	259.46	290.27	199.53	250.29	217.68	262.74	222.5	COG3686	S:Function unknown	GO:0016021	CC:integral component of membrane;
BAB_RS31625	-	autotransporter outer membrane beta-barrel domain-containing protein	0.934	-0.099007742	0.100192206	0.206489775	no	down	mRNA	7.35	6.82	7.79	6.27	6.41	6.57	7.32	6.41666667	COG3468;ENOG410XU4B	N:Cell motility;S:Function unknown	GO:0019867;GO:0004812	CC:outer membrane;MF:aminoacyl-tRNA ligase activity;
BAB_RS22595	-	BA14K family protein	0.857	-0.22186945	0.100816029	0.207376378	no	down	mRNA	298.49	201.16	230.49	225.38	183.96	183.29	243.38	197.543333	------	------	GO:0009405	BP:pathogenesis;
BAB_RS26915	-	HTH-type quorum sensing-dependent transcriptional regulator VjbR	0.942	-0.086016336	0.10076835	0.207411089	no	down	mRNA	216.68	207.37	223.27	185.2	194.22	194.97	215.773	191.463333	COG2771	K:Transcription	GO:0009372;GO:0006355;GO:0006351;GO:0006355;GO:0006351;GO:00	BP:quorum sensing;BP:regulation of transcription, DNA-templated;BP:transcription, DNA-templated;BP:regulation of transcription, DNA-templated;BP:transcription, DNA-templated;BP:quorum sensing;BP:transcription, DNA-templated;BP:regulation of transcription, DNA-templated;BP:quorum sensing;BP:regulation of transcription, DNA-templated;BP:transcription, DNA-templated;MF:DNA binding;MF:DNA binding;MF:DNA binding;MF:DNA binding;
BAB_RS19390	-	hypothetical protein	0.93	-0.105313244	0.101674397	0.208607815	no	down	mRNA	376.91	382.3	390.67	339.83	349.64	332.31	383.293	340.593333	ENOG410XYKD	S:Function unknown	------	------
BAB_RS32765	-	hypothetical protein	0.522	-0.938249699	0.101660552	0.208712687	no	down	mRNA	4.7	8.17	5.07	2.03	4.64	2.21	5.98	2.96	------		------	------
BAB_RS22320	-	LysR family transcriptional regulator	0.915	-0.128489578	0.102943905	0.210540271	no	down	mRNA	83.4	73.38	78.21	71.11	62.45	69.16	78.33	67.5733333	ENOG410XR9D	K:Transcription	GO:0006351;GO:0006351;GO:0003700;GO:0003677;GO:0003677;GO:00	BP:transcription, DNA-templated;BP:transcription, DNA-templated;MF:transcription factor activity, sequence-specific DNA binding;MF:DNA binding;MF:DNA binding;MF:transcription factor activity, sequence-specific DNA binding;
BAB_RS20930	-	reductase	0.872	-0.198053778	0.10400995	0.212450076	no	down	mRNA	129.47	142.62	135.96	129.68	113.14	98.93	136.017	113.916667	COG0633	C:Energy production and conversion	GO:0051537;GO:0009055	MF:2 iron, 2 sulfur cluster binding;MF:electron carrier activity;
BAB_RS25180	-	N-acetyltransferase	0.884	-0.177943231	0.104168716	0.21263919	no	down	mRNA	27.46	26.32	28.7	21.05	24.74	23.18	27.4933	22.99	ENOG4111XZR	S:Function unknown	GO:0016740;GO:0016740;GO:0016746	MF:transferase activity;MF:transferase activity;MF:transferase activity, transferring acyl groups;
BAB_RS22920	-	AEC family transporter	0.865	-0.208705707	0.104827256	0.213576393	no	down	mRNA	44.14	41.43	61.43	39.5	36.45	44.08	49	40.01	COG0679	S:Function unknown	GO:0055085;GO:0016021	BP:transmembrane transport;CC:integral component of membrane;
BAB_RS29140	-	ABC transporter ATP-binding protein	0.764	-0.387705443	0.104749893	0.213689782	no	down	mRNA	6.08	5.04	6.36	3.55	3.82	5.34	5.82667	4.23666667	COG0411	F:Nucleotide transport and metabolism	GO:0005524;GO:0016887;GO:0016887;GO:0005524	MF:ATP binding;MF:ATPase activity;MF:ATPase activity;MF:ATP binding;
BAB_RS28895	-	hypothetical protein	0.885	-0.176031996	0.105142061	0.213946449	no	down	mRNA	100.1	95.99	118.94	92.84	94.58	77.92	105.01	88.4466667	------	------	------	------
BAB_RS30125	-	glycerol kinase	0.717	-0.480241511	0.105734526	0.214879844	no	down	mRNA	1.53	2.93	2.88	1.4	1.91	1.79	2.44667	1.7	COG0554	C:Energy production and conversion	GO:0019563;GO:0006071;GO:0006072;GO:0004370;GO:0005524	BP:glycerol catabolic process;BP:glycerol metabolic process;BP:glycerol-3-phosphate metabolic process;MF:glycerol kinase activity;MF:ATP binding;
BAB_RS24705	-	23S rRNA (pseudouridine(1915)-N(3))-methyltransferase RlmH	0.918	-0.123599587	0.106021324	0.215326494	no	down	mRNA	721.66	742.39	813.18	616.28	719.78	659.37	759.077	665.143333	COG1576	S:Function unknown	GO:0006364;GO:0005737;GO:0005737;GO:0008168;GO:0070038;GO:00	BP:rRNA processing;CC:cytoplasm;CC:cytoplasm;MF:methyltransferase activity;MF:rRN;MF:rRN;
BAB_RS16810	-	LuxR family transcriptional regulator	0.922	-0.116445825	0.106870815	0.216777732	no	down	mRNA	86.53	83.72	87.59	76.91	69.4	78.34	85.9467	74.8833333	COG2771	K:Transcription	GO:0006355;GO:0006351;GO:0006355;GO:0006351;GO:0003677;GO:00	BP:regulation of transcription, DNA-templated;BP:transcription, DNA-templated;BP:regulation of transcription, DNA-templated;BP:transcription, DNA-templated;MF:DNA binding;MF:DNA binding;
BAB_RS27615	-	NAD(P)(+) transhydrogenase (Re/Si-specific) subunit alpha	0.932	-0.101306016	0.10698405	0.216870506	no	down	mRNA	197.01	179.85	189.91	156.57	176.18	165.05	188.923	165.933333	COG3288	C:Energy production and conversion	GO:0008750;GO:0016491;GO:0016491	MF:NA;MF:oxidoreductase activity;MF:oxidoreductase activity;
BAB_RS16570	-	DNA repair protein MmcB-related protein	0.912	-0.133419741	0.107074469	0.216916941	no	down	mRNA	195.26	192.39	207.82	172.42	187.66	155.77	198.49	171.95	COG5321	S:Function unknown	GO:0004518;GO:0003676	MF:nuclease activity;MF:nucleic acid binding;
BAB_RS19915	-	NADH-quinone oxidoreductase subunit M	0.928	-0.107576677	0.107268863	0.217173822	no	down	mRNA	517.34	453.57	476.39	400.77	443.33	421.72	482.433	421.94	COG1008	C:Energy production and conversion	GO:0006120;GO:0006120;GO:0016021;GO:0016021;GO:0008137;GO:00	BP:mitochondrial electron transport, NADH to ubiquinone;BP:mitochondrial electron transport, NADH to ubiquinone;CC:integral component of membrane;CC:integral component of membrane;MF:NADH dehydrogenas;MF:NADH dehydrogenas;
BAB_RS31350	-	ABC transporter ATP-binding protein	0.86	-0.217296699	0.107684881	0.217604731	no	down	mRNA	21.25	21	19.05	14.84	15.92	19.11	20.4333	16.6233333	COG4608	S:Function unknown	GO:0015833;GO:0015833;GO:0015833;GO:0005524;GO:0016887;GO:00	BP:peptide transport;BP:peptide transport;BP:peptide transport;MF:ATP binding;MF:ATPase activity;MF:ATP binding;MF:ATPase activity;MF:ATPase activity;MF:ATP binding;
BAB_RS25335	-	6-carboxytetrahydropterin synthase QueD	0.611	-0.710020792	0.107617149	0.21774175	no	down	mRNA	8.26	10.43	5.65	5.8	2.89	5.75	8.11333	4.81333333	COG0720	H:Coenzyme transport and metabolism	GO:0008616;GO:0008616;GO:0016829;GO:0046872;GO:0016829;GO:00	BP:queuosine biosynthetic process;BP:queuosine biosynthetic process;MF:lyase activity;MF:metal ion binding;MF:lyase activity;MF:metal ion binding;
BAB_RS28525	-	ABC transporter permease	0.887	-0.172756816	0.108295458	0.218563635	no	down	mRNA	19.3	18.29	19.51	15	17.21	15.51	19.0333	15.9066667	COG0601	S:Function unknown	GO:0055085;GO:0055085;GO:0016021;GO:0005886;GO:0016021;GO:00	BP:transmembrane transport;BP:transmembrane transport;CC:integral component of membrane;CC:plasma membrane;CC:integral component of membrane;CC:plasma membrane;
BAB_RS24830	-	M23 family peptidase	0.927	-0.109888905	0.108455975	0.218750187	no	down	mRNA	80.45	71.73	77.23	66.82	62.69	70.53	76.47	66.68	COG0739	S:Function unknown	GO:0016021;GO:0016021;GO:0016787	CC:integral component of membrane;CC:integral component of membrane;MF:hydrolase activity;
BAB_RS18195	-	class I SAM-dependent methyltransferase	0.914	-0.13003756	0.109860553	0.22102815	no	down	mRNA	77.39	84.93	95.36	70.74	78.99	72.22	85.8933	73.9833333	COG2230	M:Cell wall/membrane/envelope biogenesis	GO:0008610;GO:0008610;GO:0008610;GO:0008168;GO:0008825;GO:00	BP:lipid biosynthetic process;BP:lipid biosynthetic process;BP:lipid biosynthetic process;MF:methyltransferase activity;MF:cyclopropane-fatty-acyl-phospholipid synthase activity;MF:cyclopropane-fatty-acyl-phospholipid synthase activity;
BAB_RS17230	-	hypothetical protein	0.665	-0.588584604	0.110144697	0.221322646	no	down	mRNA	3.8	3.18	5.37	2.49	3.33	1.99	4.11667	2.60333333	------	------	GO:0016021	CC:integral component of membrane;
BAB_RS21845	-	50S ribosomal protein L6	0.936	-0.094863069	0.110365315	0.221488918	no	down	mRNA	925.9	776.01	871.48	775.74	751.36	762.89	857.797	763.33	COG0097	J:Translation, ribosomal structure and biogenesis	GO:0006412;GO:0006412;GO:0006412;GO:0005840;GO:0005840;GO:00	BP:translation;BP:translation;BP:translation;CC:ribosome;CC:ribosome;CC:ribosome;MF:rRNA binding;MF:structural constituent of ribosome;MF:structural constituent of ribosome;MF:rRNA binding;MF:structural constituent of ribosome;MF:rRNA binding;
BAB_RS19125	-	4-hydroxy-tetrahydrodipicolinate synthase	0.925	-0.112294319	0.110463654	0.221547889	no	down	mRNA	133.25	112.57	129.68	113.36	110.3	103.61	125.167	109.09	COG0329	E:Amino acid transport and metabolism	GO:0019877;GO:0009089;GO:0009089;GO:0019877;GO:0005737;GO:00	BP:diaminopimelate biosynthetic process;BP:lysine biosynthetic process via diaminopimelate;BP:lysine biosynthetic process via diaminopimelate;BP:diaminopimelate biosynthetic process;CC:cytoplasm;CC:cytoplasm;MF:4-hydroxy-tetrahydrodipicolinate synthase;MF:4-hydroxy-tetrahydrodipicolinate synthase;
BAB_RS17615	-	hypothetical protein	0.878	-0.187233462	0.111087594	0.222660287	no	down	mRNA	43.72	35.12	38.69	30.96	30.35	36.21	39.1767	32.5066667	ENOG410Y1WJ;ENOG41127ES	S:Function unknown;	GO:0006486;GO:0006486;GO:0008373;GO:0008373	BP:protein glycosylation;BP:protein glycosylation;MF:sialyltransferase activity;MF:sialyltransferase activity;
BAB_RS28235	-	M48 family peptidase	0.872	-0.197604478	0.111882977	0.224114716	no	down	mRNA	68.93	62.89	62.51	60.34	55.73	43.71	64.7767	53.26	COG1451	S:Function unknown	------	------
BAB_RS29755	-	hypothetical protein	0.761	-0.393946003	0.111984478	0.224178272	no	down	mRNA	70.86	34.92	46.93	40.84	38.79	29.28	50.9033	36.3033333	------	------	------	------
BAB_RS33375	-	hypothetical protein	0.636	-0.652745996	0.112060524	0.224190825	no	down	mRNA	5.1	7.16	4.64	3.42	3.64	3.23	5.63333	3.43	ENOG410ZCKG	S:Function unknown	------	------
BAB_RS16945	-	bifunctional hydroxymethylpyrimidine kinase/phosphomethylpyrimidine kinase	0.868	-0.204462414	0.112507723	0.224665826	no	down	mRNA	13.48	15.31	17.79	12.27	13.58	12.55	15.5267	12.8	COG0351	H:Coenzyme transport and metabolism	GO:0009228;GO:0009228;GO:0009228;GO:0009228;GO:0005524;GO:00	BP:thiamine biosynthetic process;BP:thiamine biosynthetic process;BP:thiamine biosynthetic process;BP:thiamine biosynthetic process;MF:ATP binding;MF:hydroxymethylpyrimidine kinase activity;MF:phosphomethylpyrimidine kinase activity;MF:ATP binding;MF:D-arabinono-1,4-lactone oxidase activity;MF:phosphomethylpyrimidine kinase activity;MF:phosphomethylpyrimidine kinase activity;MF:peptidase activity;MF:D-arabinono-1,4-lactone oxidase activity;MF:ATP binding;MF:ATP binding;MF:phosphomethylpyrimidine kinase activity;
BAB_RS18145	-	hypothetical protein	0.923	-0.114821183	0.11274189	0.224993597	no	down	mRNA	215.38	179.59	192.3	162.56	174.9	174.48	195.757	170.646667	COG3895	S:Function unknown	------	------
BAB_RS17835	-	bifunctional DNA-binding transcriptional regulator/O6-methylguanine-DNA methyltransferase Ada	0.924	-0.113511169	0.113320234	0.225587305	no	down	mRNA	49.48	51.78	57.14	45.57	47.52	45.1	52.8	46.0633333	COG0350	O:Posttranslational modification, protein turnover,	GO:0006281;GO:0006351;GO:0043565;GO:0047009;GO:0003700;GO:00	BP:DNA repair;BP:transcription, DNA-templated;MF:sequence-specific DNA binding;MF:3-alpha-hydroxy-5-beta-androstane-17-one 3-alpha-dehydrogenase activity;MF:transcription factor activity, sequence-specific DNA binding;MF:zinc ion binding;MF:methylated-DNA-[protein]-cysteine S-methyltransferase activity;
BAB_RS23395	-	aspartyl/asparaginyl beta-hydroxylase	0.858	-0.220378636	0.113181239	0.225590149	no	down	mRNA	163.29	184.64	140.91	134.39	131.39	149.95	162.947	138.576667	COG3555	O:Posttranslational modification, protein turnover,	GO:0018193;GO:0018193;GO:0004597	BP:peptidyl-amino acid modification;BP:peptidyl-amino acid modification;MF:peptide-aspartate beta-dioxygenase activity;
BAB_RS24565	-	FUSC family protein	0.916	-0.125974157	0.114943732	0.22825353	no	down	mRNA	58.37	61.67	60.55	51.31	49.76	55.48	60.1967	52.1833333	COG1289	S:Function unknown	GO:0016021	CC:integral component of membrane;
BAB_RS24715	-	nicotinate-nucleotide adenylyltransferase	0.816	-0.294093699	0.115194302	0.228468699	no	down	mRNA	21.27	15.26	27.31	14.83	16.6	17.89	21.28	16.44	COG1057	H:Coenzyme transport and metabolism	GO:0009435;GO:0009435;GO:0009435;GO:0004515;GO:0005524;GO:00	BP:NAD biosynthetic process;BP:NAD biosynthetic process;BP:NAD biosynthetic process;MF:nicotinate-nucleotide adenylyltransferase activity;MF:ATP binding;MF:ATP binding;MF:nicotinate-nucleotide adenylyltransferase activity;MF:nicotinate-nucleotide adenylyltransferase activity;MF:ATP binding;MF:hydrolase activity;
BAB_RS30570	-	sugar ABC transporter permease	0.821	-0.283797512	0.115165399	0.228552456	no	down	mRNA	4.75	5.22	4.66	4.14	3.8	3.4	4.87667	3.78	COG1175	G:Carbohydrate transport and metabolism	GO:0055085;GO:0055085;GO:0016021;GO:0005886;GO:0016021;GO:00	BP:transmembrane transport;BP:transmembrane transport;CC:integral component of membrane;CC:plasma membrane;CC:integral component of membrane;CC:plasma membrane;
BAB_RS29715	-	MFS transporter	0.925	-0.113252286	0.115362858	0.228661853	no	down	mRNA	34.45	31.12	34.11	29.64	28.57	28.53	33.2267	28.9133333	ENOG410XQKC	G:Carbohydrate transport and metabolism	GO:0008643;GO:0055085;GO:0016021;GO:0016021;GO:0022857;GO:00	BP:carbohydrate transport;BP:transmembrane transport;CC:integral component of membrane;CC:integral component of membrane;MF:transmembrane transporter activity;MF:transporter activity;
BAB_RS32375	-	hypothetical protein	0.914	-0.129769869	0.116066761	0.229773569	no	down	mRNA	40.63	39.2	41.38	33.37	34.06	37.34	40.4033	34.9233333	------	------	------	------
BAB_RS20430	-	DUF455 domain-containing protein	0.892	-0.165356514	0.116929421	0.231196449	no	down	mRNA	33.24	29	32.09	27.05	28.72	23.28	31.4433	26.35	COG2833	S:Function unknown	------	------
BAB_RS17960	-	polyprenyl synthetase family protein	0.937	-0.094181294	0.117335641	0.231856959	no	down	mRNA	177.47	153.75	181.27	143.6	151.58	156.55	170.83	150.576667	COG0142	H:Coenzyme transport and metabolism	GO:0008299;GO:0008299;GO:0008299;GO:0004161;GO:0016740;GO:00	BP:isoprenoid biosynthetic process;BP:isoprenoid biosynthetic process;BP:isoprenoid biosynthetic process;MF:dimethylallyltranstransferase activity;MF:transferase activity;MF:dimethylallyltranstransferase activity;MF:geranyltranstransferase activity;
BAB_RS23295	-	hypothetical protein	0.891	-0.166500553	0.118049556	0.233124292	no	down	mRNA	80.68	58.26	73.55	58.37	60.28	59.94	70.83	59.53	ENOG4111XDJ	S:Function unknown	------	------
BAB_RS21985	-	transcription termination/antitermination protein NusG	0.914	-0.130307254	0.118128935	0.233137757	no	down	mRNA	1210.07	984.84	1016.62	998.68	928.07	860.11	1070.51	928.953333	COG0250	K:Transcription	GO:0006354;GO:0006353;GO:0032784;GO:0031564;GO:0006354;GO:00	BP:DNA-templated transcription, elongation;BP:DNA-templated transcription, termination;BP:regulation of DNA-templated transcription, elongation;BP:transcription antitermination;BP:DNA-templated transcription, elongation;BP:transcription antitermination;BP:regulation of DNA-templated transcription, elongation;BP:DNA-templated transcription, termination;
BAB_RS25345	-	ATP:cob(I)alamin adenosyltransferase	0.893	-0.162698921	0.118978663	0.23438286	no	down	mRNA	38.6	32.13	40.58	31.74	29.14	33.18	37.1033	31.3533333	COG2096	S:Function unknown	GO:0016740;GO:0047161;GO:0016740;GO:0047161	MF:transferase activity;MF:tartronate O-hydroxycinnamoyltransferase activity;MF:transferase activity;MF:tartronate O-hydroxycinnamoyltransferase activity;
BAB_RS16095	-	NAD(+) diphosphatase	0.923	-0.116206704	0.11972874	0.235139634	no	down	mRNA	51.31	55.53	60.29	46.16	51.52	47.54	55.71	48.4066667	COG2816	L:Replication, recombination and repair	GO:0016787;GO:0046872	MF:hydrolase activity;MF:metal ion binding;
BAB_RS30880	-	hypothetical protein	0.918	-0.123066537	0.120468334	0.236447621	no	down	mRNA	119.14	102.43	116.7	86.98	101.47	103.87	112.757	97.44	COG2203;ENOG410XNMH	T:Signal transduction mechanisms;	------	------
BAB_RS32070	-	hypothetical protein	0.851	-0.233225852	0.120843885	0.237039928	no	down	mRNA	25.37	27.59	25.3	18.55	23.26	21.61	26.0867	21.14	------	------	------	------
BAB_RS28050	-	Zn-dependent protease	0.923	-0.116320951	0.122006808	0.238738048	no	down	mRNA	53.67	48.87	55.12	47.99	45.33	43.95	52.5533	45.7566667	COG3824	S:Function unknown	GO:0008233;GO:0003991	MF:peptidase activity;MF:acetylglutamate kinase activity;
BAB_RS30285	-	GntR family transcriptional regulator	0.858	-0.220346267	0.122089023	0.238753519	no	down	mRNA	75.45	77.51	72.01	57.38	50.25	75.53	74.99	61.0533333	ENOG4111XU0;ENOG410ZZTA	K:Transcription;	GO:0006351;GO:0006351;GO:0003700;GO:0003677;GO:0003677;GO:00	BP:transcription, DNA-templated;BP:transcription, DNA-templated;MF:transcription factor activity, sequence-specific DNA binding;MF:DNA binding;MF:DNA binding;MF:transcription factor activity, sequence-specific DNA binding;
BAB_RS16480	-	porin family protein	0.896	-0.159110594	0.121979936	0.238830916	no	down	mRNA	145.78	119.14	121.37	105.3	121.68	98.71	128.763	108.563333	COG3637	M:Cell wall/membrane/envelope biogenesis	GO:0019867	CC:outer membrane;
BAB_RS19920	-	NADH-quinone oxidoreductase subunit NuoN	0.93	-0.103936308	0.122651016	0.239415379	no	down	mRNA	407.74	378.69	384.7	325.44	367.28	334.91	390.377	342.543333	COG1007	C:Energy production and conversion	GO:0042773;GO:0016021;GO:0005886;GO:0008137;GO:0048038	BP:ATP synthesis coupled electron transport;CC:integral component of membrane;CC:plasma membrane;MF:NADH dehydrogenas;MF:quinone binding;
BAB_RS31640	-	D-xylose ABC transporter ATP-binding protein	0.917	-0.125117314	0.122943867	0.239841315	no	down	mRNA	22.64	20.94	23.43	18.47	18.47	20.95	22.3367	19.2966667	COG1129	S:Function unknown	GO:0005886;GO:0005886;GO:0015614;GO:0005524;GO:0005524;GO:00	CC:plasma membrane;CC:plasma membrane;MF:D-xylose-importing ATPase activity;MF:ATP binding;MF:ATP binding;MF:D-xylose-importing ATPase activity;
BAB_RS31575	-	flagellar hook-associated protein FlgK	0.809	-0.305981953	0.123458388	0.240698908	no	down	mRNA	4.16	3.13	3.39	2.44	3.15	2.39	3.56	2.66	COG1256	N:Cell motility	GO:0044780;GO:0044780;GO:0009424;GO:0009424;GO:0005198;GO:00	BP:bacterial-type flagellum assembly;BP:bacterial-type flagellum assembly;CC:bacterial-type flagellum hook;CC:bacterial-type flagellum hook;MF:structural molecule activity;MF:structural molecule activity;
BAB_RS28320	-	TIGR02186 family protein	0.879	-0.186476574	0.124248522	0.241799214	no	down	mRNA	18.53	20.84	21.93	16.24	17.01	18.07	20.4333	17.1066667	ENOG410Y62H	S:Function unknown	GO:0016021	CC:integral component of membrane;
BAB_RS21655	-	YdcF family protein	0.889	-0.170158013	0.12442193	0.24199011	no	down	mRNA	73.11	77.61	99.49	64.58	68.93	77.13	83.4033	70.2133333	COG1434	E:Amino acid transport and metabolism	GO:0016021	CC:integral component of membrane;
BAB_RS26345	-	enoyl-CoA hydratase	0.928	-0.10741511	0.124893782	0.242760871	no	down	mRNA	100.61	104.46	107.65	88.01	92.09	95	104.24	91.7	COG1024	L:Replication, recombination and repair	GO:0008152;GO:0008152;GO:0003824;GO:0003824;GO:0016853;GO:00	BP:metabolic process;BP:metabolic process;MF:catalytic activity;MF:catalytic activity;MF:isomerase activity;MF:enoyl-CoA hydratase activity;
BAB_RS19810	-	aminotransferase class V-fold PLP-dependent enzyme	0.945	-0.081673573	0.125308899	0.242979779	no	down	mRNA	193.76	187.56	195.94	167.67	171.09	175.29	192.42	171.35	COG2873	E:Amino acid transport and metabolism	GO:0006520;GO:0006520;GO:0016829;GO:0030170;GO:0016765;GO:00	BP:cellular amino acid metabolic process;BP:cellular amino acid metabolic process;MF:lyase activity;MF:pyridoxal phosphate binding;MF:transferase activity, transferring alkyl or ary;MF:transferase activity, transferring alkyl or ary;MF:lyase activity;MF:pyridoxal phosphate binding;MF:O-acetylhomoserine aminocarboxypropyltransferase activity;MF:cysteine synthase activity;MF:O-acetylhomoserine aminocarboxypropyltransferase activity;MF:pyridoxal phosphate binding;
BAB_RS16825	-	adenylyl-sulfate kinase	0.856	-0.2237023	0.125182498	0.243028017	no	down	mRNA	9.59	7.1	7.25	6.06	6.12	6.94	7.98	6.37333333	COG0529	P:Inorganic ion transport and metabolism	GO:0070814;GO:0000103;GO:0070814;GO:0000103;GO:0004020;GO:00	BP:hydrogen sulfide biosynthetic process;BP:sulfate assimilation;BP:hydrogen sulfide biosynthetic process;BP:sulfate assimilation;MF:adenylylsulfate kinase activity;MF:GTPase activity;MF:sulfate adenylyltransferas;MF:translation elongation factor activity;MF:GTP binding;MF:ATP binding;MF:oxidoreductase activity;MF:adenylylsulfate kinase activity;MF:ATP binding;MF:GTP binding;MF:GTPase activity;MF:sulfate adenylyltransferas;
BAB_RS22455	-	ABC transporter ATP-binding protein	0.896	-0.157782516	0.125162604	0.243136304	no	down	mRNA	49.17	49.95	63.15	48.67	46.82	42.19	54.09	45.8933333	COG1120	P:Inorganic ion transport and metabolism	GO:0005524;GO:0016887	MF:ATP binding;MF:ATPase activity;
BAB_RS19200	-	GTPase Era	0.929	-0.106535309	0.125843023	0.243721298	no	down	mRNA	156.61	151.81	157.06	146.08	130.94	131.54	155.16	136.186667	COG1159	S:Function unknown	GO:0042274;GO:0042254;GO:0042274;GO:0042274;GO:0005737;GO:00	BP:ribosomal small subunit biogenesis;BP:ribosome biogenesis;BP:ribosomal small subunit biogenesis;BP:ribosomal small subunit biogenesis;CC:cytoplasm;CC:plasma membrane;CC:cytoplasm;CC:plasma membrane;CC:cytoplasm;CC:plasma membrane;MF:small ribosomal subunit rRNA binding;MF:GTPase activity;MF:rRNA binding;MF:GTP binding;MF:small ribosomal subunit rRNA binding;MF:GTPase activity;MF:GTP binding;MF:small ribosomal subunit rRNA binding;MF:GTPase activity;MF:translation elongation factor activity;MF:GTP binding;
BAB_RS26225	-	tRNA (guanosine(46)-N7)-methyltransferase TrmB	0.908	-0.138675977	0.12671214	0.24525669	no	down	mRNA	37.09	37.07	42.08	33.29	33.03	33.93	38.7467	33.4166667	COG0220	S:Function unknown	GO:0008176	MF:tRN;
BAB_RS19095	-	-	0.743	-0.428349881	0.127681163	0.246242244	no	down	mRNA	12.63	7.02	9.63	7.27	5.7	7.5	9.76	6.82333333	------	------	GO:0003677;GO:0000150	MF:DNA binding;MF:recombinase activity;
BAB_RS26945	-	hypothetical protein	0.446	-1.166143777	0.127453884	0.246247341	no	down	mRNA	1.87	0.66	2.56	1.11	0.71	0.3	1.69667	0.70666667	ENOG410ZCGY	S:Function unknown	GO:0016021;GO:0042597	CC:integral component of membrane;CC:periplasmic space;
BAB_RS21600	-	DUF1849 domain-containing protein	0.929	-0.10652768	0.130697366	0.25130499	no	down	mRNA	384.8	333.53	334.45	314.41	299.63	308.94	350.927	307.66	ENOG4110V3Z	S:Function unknown	GO:0016021;GO:0005524	CC:integral component of membrane;MF:ATP binding;
BAB_RS26075	-	hypothetical protein	0.935	-0.097524777	0.131416036	0.25253572	no	down	mRNA	306.5	271.14	287.29	241.03	268.89	254.95	288.31	254.956667	COG3963	I:Lipid transport and metabolism	GO:0008168;GO:0000773;GO:0004608;GO:0080101;GO:0000179;GO:00	MF:methyltransferase activity;MF:phosphatidyl-N-methylethanolamine N-methyltransferase activity;MF:phosphatidylethanolamine N-methyltransferase activity;MF:phosphatidyl-N-dimethylethanolamine N-methyltransferase activity;MF:rRN;MF:rRN;MF:methyltransferase activity;MF:phosphatidylethanolamine N-methyltransferase activity;
BAB_RS32880	-	hypothetical protein	0.805	-0.312927492	0.132826334	0.254940866	no	down	mRNA	15.78	13.44	16.3	12.6	11.78	10.76	15.1733	11.7133333	COG0183	I:Lipid transport and metabolism	------	------
BAB_RS22660	-	NAD(P)/FAD-dependent oxidoreductase	0.879	-0.186734882	0.132782519	0.255009106	no	down	mRNA	18.91	20.71	17.71	17.71	15.51	14.31	19.11	15.8433333	COG2081	E:Amino acid transport and metabolism	------	------
BAB_RS25165	-	hypothetical protein	0.736	-0.44142951	0.133260103	0.255468204	no	down	mRNA	357.24	214.44	225.81	164.38	281.54	126.46	265.83	190.793333	ENOG410Z7I0	S:Function unknown	------	------
BAB_RS16375	-	heme exporter protein CcmC	0.938	-0.092374056	0.135923058	0.259489475	no	down	mRNA	141.06	138.21	149.12	121.96	130.58	127.88	142.797	126.806667	COG0755	O:Posttranslational modification, protein turnover,	GO:0017004;GO:0017004;GO:0017004;GO:0016021;GO:0005886;GO:00	BP:cytochrome complex assembly;BP:cytochrome complex assembly;BP:cytochrome complex assembly;CC:integral component of membrane;CC:plasma membrane;CC:integral component of membrane;CC:plasma membrane;CC:integral component of membrane;CC:plasma membrane;MF:heme transporter activity;MF:heme binding;MF:heme transporter activity;MF:heme binding;MF:heme binding;MF:heme transporter activity;
BAB_RS17055	-	mandelate racemase/muconate lactonizing enzyme family protein	0.931	-0.10354998	0.135694789	0.259516284	no	down	mRNA	92.09	93.11	100.8	88.61	80.08	82.79	95.3333	83.8266667	COG4948	M:Cell wall/membrane/envelope biogenesis	GO:0009063;GO:0009063;GO:0046872;GO:0046872;GO:0018838	BP:cellular amino acid catabolic process;BP:cellular amino acid catabolic process;MF:metal ion binding;MF:metal ion binding;MF:mandelate racemase activity;
BAB_RS33010	-	hypothetical protein	0.705	-0.505120377	0.135808228	0.259578726	no	down	mRNA	41.57	50.12	50.95	49.78	27.09	26.66	47.5467	34.51	------	------	------	------
BAB_RS27315	-	16S rRNA (guanine(966)-N(2))-methyltransferase RsmD	0.882	-0.180550828	0.138127758	0.26291735	no	down	mRNA	60.07	47.36	62.97	52.01	46.37	44.28	56.8	47.5533333	COG0742	L:Replication, recombination and repair	GO:0031167;GO:0008168;GO:0003676	BP:rRNA methylation;MF:methyltransferase activity;MF:nucleic acid binding;
BAB_RS29850	-	Na+/H+ antiporter subunit C	0.833	-0.263165665	0.138126801	0.263071376	no	down	mRNA	49.35	38.03	47.02	35.14	38.75	34.91	44.8	36.2666667	COG1006	P:Inorganic ion transport and metabolism	GO:0016021	CC:integral component of membrane;
BAB_RS21065	-	alanyl-tRNA editing protein	0.909	-0.138022223	0.13811476	0.263204462	no	down	mRNA	95.5	85.98	91.33	82.74	68.6	83.49	90.9367	78.2766667	COG2872	S:Function unknown	GO:0006419;GO:0006419;GO:0016787;GO:0004813;GO:0003676;GO:00	BP:alanyl-tRNA aminoacylation;BP:alanyl-tRNA aminoacylation;MF:hydrolase activity;MF:alanine-tRNA ligase activity;MF:nucleic acid binding;MF:ATP binding;MF:alanine-tRNA ligase activity;MF:ATP binding;MF:nucleic acid binding;
BAB_RS32380	-	hypothetical protein	0.869	-0.201917109	0.138848132	0.263975768	no	down	mRNA	26.66	20.88	23.45	18.75	17.5	21.98	23.6633	19.41	------	------	------	------
BAB_RS29350	-	ribonuclease R	0.933	-0.100256356	0.140295625	0.2660979	no	down	mRNA	70.85	69.49	85.09	66	68	63.66	75.1433	65.8866667	COG0557	K:Transcription	GO:0005737;GO:0005737;GO:0008859;GO:0003723;GO:0008859;GO:00	CC:cytoplasm;CC:cytoplasm;MF:exoribonuclease II activity;MF:RNA binding;MF:exoribonuclease II activity;MF:RNA binding;MF:oxidoreductase activity;
BAB_RS25830	-	XRE family transcriptional regulator	0.762	-0.391561609	0.141130304	0.26736537	no	down	mRNA	13.8	8.68	14.93	11.12	6.69	9.46	12.47	9.09	COG1396	S:Function unknown	GO:0043565	MF:sequence-specific DNA binding;
BAB_RS31755	-	ABC transporter ATP-binding protein	0.817	-0.291271875	0.142248251	0.26837559	no	down	mRNA	3.36	2.15	3.14	2.23	2.41	1.91	2.88333	2.18333333	COG0410	E:Amino acid transport and metabolism	GO:0016887;GO:0005524;GO:0005524;GO:0016887	MF:ATPase activity;MF:ATP binding;MF:ATP binding;MF:ATPase activity;
BAB_RS26195	-	PhoH family protein	0.937	-0.093856857	0.141853006	0.268417967	no	down	mRNA	453.45	441.72	506.9	401.74	443.25	394.42	467.357	413.136667	COG1702	T:Signal transduction mechanisms	GO:0005524	MF:ATP binding;
BAB_RS29615	-	YecA family protein	0.841	-0.249721156	0.14351478	0.269972476	no	down	mRNA	27.91	34.19	30.06	29	25.03	19.86	30.72	24.63	COG3318;ENOG410ZJZ4	S:Function unknown;	------	------
BAB_RS28070	-	glyoxalase	0.925	-0.112260014	0.143875891	0.27049341	no	down	mRNA	67.17	59.99	60.04	53.49	56.43	53.17	62.4	54.3633333	ENOG4111N93	S:Function unknown	GO:0016829;GO:0051213;GO:0016829;GO:0051213;GO:0004462	MF:lyase activity;MF:dioxygenase activity;MF:lyase activity;MF:dioxygenase activity;MF:lactoylglutathione lyase activity;
BAB_RS17610	-	glycosyl transferase	0.879	-0.186128243	0.144270014	0.270759085	no	down	mRNA	35.47	29.17	33.14	31.3	23.47	26.3	32.5933	27.0233333	COG3306	M:Cell wall/membrane/envelope biogenesis	GO:0016740;GO:0016740;GO:0016757	MF:transferase activity;MF:transferase activity;MF:transferase activity, transferring glycosyl groups;
BAB_RS30965	-	50S ribosomal protein L34	0.682	-0.55253063	0.144359386	0.270768656	no	down	mRNA	707.2	731.25	864	673.51	382.76	655.29	767.483	570.52	COG0230	J:Translation, ribosomal structure and biogenesis	GO:0006412;GO:0005840;GO:0003735	BP:translation;CC:ribosome;MF:structural constituent of ribosome;
BAB_RS23210	-	lytic transglycosylase	0.918	-0.12383919	0.144114535	0.270783627	no	down	mRNA	48	50.44	51.59	43.83	46.02	40.38	50.01	43.41	COG0741	L:Replication, recombination and repair	GO:0008152;GO:0016021;GO:0016798	BP:metabolic process;CC:integral component of membrane;MF:hydrolase activity, acting on glycosyl bonds;
BAB_RS22385	-	sulfate ABC transporter permease subunit CysT	0.794	-0.331900773	0.144713013	0.271115401	no	down	mRNA	82.36	95.54	90.12	57.72	47.82	97.41	89.34	67.65	COG0555	S:Function unknown	GO:0016021;GO:0016021;GO:0015419;GO:0015419	CC:integral component of membrane;CC:integral component of membrane;MF:ATPase-coupled sulfate transmembrane transporter activity;MF:ATPase-coupled sulfate transmembrane transporter activity;
BAB_RS19595	-	amino acid ABC transporter ATP-binding protein	0.937	-0.09384025	0.146038644	0.273280235	no	down	mRNA	340.19	311.45	328.93	265.94	305.03	297.26	326.857	289.41	COG1126	E:Amino acid transport and metabolism	GO:0015424;GO:0005524	MF:amino acid-transporting ATPase activity;MF:ATP binding;
BAB_RS17790	-	cytochrome-c oxidase%2C cbb3-type subunit III	0.947	-0.079200225	0.146272434	0.273399261	no	down	mRNA	1750.99	1612.95	1747.81	1522.79	1485.81	1564.69	1703.92	1524.43	COG2010	C:Energy production and conversion	GO:0006811;GO:0006119;GO:0006811;GO:0006119;GO:0006811;GO:00	BP:ion transport;BP:oxidative phosphorylation;BP:ion transport;BP:oxidative phosphorylation;BP:ion transport;BP:oxidative phosphorylation;CC:integral component of membrane;CC:plasma membrane;CC:respiratory chain;CC:integral component of membrane;CC:plasma membrane;CC:respiratory chain;CC:integral component of membrane;CC:plasma membrane;CC:respiratory chain;MF:heme binding;MF:iron ion binding;MF:electron carrier activity;MF:electron carrier activity;MF:heme binding;MF:iron ion binding;MF:hydrolase activity;MF:heme binding;MF:iron ion binding;MF:electron carrier activity;MF:heme binding;MF:iron ion binding;MF:electron carrier activity;
BAB_RS21665	-	MerR family DNA-binding transcriptional regulator	0.932	-0.101592596	0.146692878	0.274025707	no	down	mRNA	94.12	85.38	91.07	77.38	85.04	75.07	90.19	79.1633333	COG0789	S:Function unknown	GO:0006355;GO:0003677	BP:regulation of transcription, DNA-templated;MF:DNA binding;
BAB_RS18450	-	ureidoglycolate lyase	0.87	-0.200943314	0.14739657	0.275020429	no	down	mRNA	61.12	45.76	41.97	39.76	42.09	39.45	49.6167	40.4333333	COG3194	F:Nucleotide transport and metabolism	GO:0000256;GO:0006145;GO:0000256;GO:0006145;GO:0006144;GO:00	BP:allantoin catabolic process;BP:purine nucleobase catabolic process;BP:allantoin catabolic process;BP:purine nucleobase catabolic process;BP:purine nucleobase metabolic process;MF:ureidoglycolate hydrolase activity;MF:ureidoglycolate lyase activity;MF:ureidoglycolate lyase activity;MF:ureidoglycolate hydrolase activity;
BAB_RS31120	-	amidohydrolase	0.912	-0.132330241	0.14913553	0.277942262	no	down	mRNA	24.34	21.04	22.98	21.61	18.93	18	22.7867	19.5133333	COG1574	G:Carbohydrate transport and metabolism	GO:0016021;GO:0016810;GO:0016810	CC:integral component of membrane;MF:hydrolase activity, acting on carbon-nitroge;MF:hydrolase activity, acting on carbon-nitroge;
BAB_RS16300	-	4a-hydroxytetrahydrobiopterin dehydratase	0.924	-0.114548423	0.150159019	0.279040445	no	down	mRNA	104.55	89.81	102.22	83.26	90.77	85.96	98.86	86.6633333	COG2154	H:Coenzyme transport and metabolism	GO:0006729;GO:0006729;GO:0008124;GO:0008124	BP:tetrahydrobiopterin biosynthetic process;BP:tetrahydrobiopterin biosynthetic process;MF:4-alpha-hydroxytetrahydrobiopterin dehydratase activity;MF:4-alpha-hydroxytetrahydrobiopterin dehydratase activity;
BAB_RS20140	-	tRNA dihydrouridine(20/20a) synthase DusA	0.919	-0.122088379	0.150358261	0.279087864	no	down	mRNA	50	47.92	50.37	47.39	41.3	39.79	49.43	42.8266667	COG0042	J:Translation, ribosomal structure and biogenesis	GO:0016740;GO:0000049;GO:0050660;GO:0102264;GO:0010181	MF:transferase activity;MF:tRNA binding;MF:flavin adenine dinucleotide binding;MF:tRNA-dihydrouridine20 synthase activity;MF:FMN binding;
BAB_RS20835	-	bifunctional ADP-dependent NAD(P)H-hydrate dehydratase/NAD(P)H-hydrate epimerase	0.936	-0.096112031	0.150134819	0.27915693	no	down	mRNA	45.35	45.03	49.19	38.47	41.65	42.89	46.5233	41.0033333	COG0062	G:Carbohydrate transport and metabolism	GO:0046496;GO:0046496;GO:0052855;GO:0005524;GO:0046872;GO:00	BP:nicotinamide nucleotide metabolic process;BP:nicotinamide nucleotide metabolic process;MF:ADP-dependent NA;MF:ATP binding;MF:metal ion binding;MF:NADHX epimerase activity;MF:NADHX epimerase activity;MF:metal ion binding;MF:ADP-dependent NA;MF:ATP binding;
BAB_RS27785	-	2-dehydro-3-deoxy-6-phosphogalactonate aldolase	0.924	-0.114355674	0.150322383	0.279182554	no	down	mRNA	201.44	206.45	220.25	171.6	203.05	175.19	209.38	183.28	COG0800	G:Carbohydrate transport and metabolism	GO:0008152;GO:0008152;GO:0016829;GO:0016829;GO:0008674	BP:metabolic process;BP:metabolic process;MF:lyase activity;MF:lyase activity;MF:2-dehydro-3-deoxy-6-phosphogalactonate aldolase activity;
BAB_RS25330	-	7-cyano-7-deazaguanine synthase	0.835	-0.259621024	0.150585555	0.279348377	no	down	mRNA	12.46	9.35	8.36	8.28	7.85	7.27	10.0567	7.8	COG0603	S:Function unknown	GO:0008616;GO:0008616;GO:0016879;GO:0008270;GO:0046872;GO:00	BP:queuosine biosynthetic process;BP:queuosine biosynthetic process;MF:ligase activity, forming carbon-nitrogen bonds;MF:zinc ion binding;MF:metal ion binding;MF:ligase activity;MF:ATP binding;MF:ATP binding;MF:ligase activity, forming carbon-nitrogen bonds;MF:zinc ion binding;
BAB_RS19610	-	RNA degradosome polyphosphate kinase	0.951	-0.072183548	0.151700091	0.280929332	no	down	mRNA	61.49	58.57	66.56	54.91	56.84	55.05	62.2067	55.6	COG0855	P:Inorganic ion transport and metabolism	GO:0006799;GO:0006799;GO:0009358;GO:0009358;GO:0005524;GO:00	BP:polyphosphate biosynthetic process;BP:polyphosphate biosynthetic process;CC:polyphosphate kinase complex;CC:polyphosphate kinase complex;MF:ATP binding;MF:metal ion binding;MF:polyphosphate kinase activity;MF:metal ion binding;MF:polyphosphate kinase activity;MF:ATP binding;
BAB_RS19735	-	integration host factor subunit alpha	0.928	-0.108029882	0.151985601	0.281295931	no	down	mRNA	613.04	559.59	501.34	459.79	522.22	508.39	557.99	496.8	COG0776	I:Lipid transport and metabolism	GO:0006310;GO:0006355;GO:0006417;GO:0006351;GO:0006310;GO:00	BP:DNA recombination;BP:regulation of transcription, DNA-templated;BP:regulation of translation;BP:transcription, DNA-templated;BP:DNA recombination;BP:regulation of transcription, DNA-templated;BP:regulation of translation;BP:transcription, DNA-templated;MF:DNA binding;MF:DNA binding;
BAB_RS19085	-	site-specific integrase	0.88	-0.184865341	0.152191724	0.281515262	no	down	mRNA	26.92	22.31	25.67	21.38	23.51	16.78	24.9667	20.5566667	ENOG410XRDV	L:Replication, recombination and repair	GO:0006310;GO:0015074;GO:0015074;GO:0006310;GO:0003677;GO:00	BP:DNA recombination;BP:DNA integration;BP:DNA integration;BP:DNA recombination;MF:DNA binding;MF:DNA binding;
BAB_RS18080	-	DUF1344 domain-containing protein	0.923	-0.115806169	0.153198511	0.283051648	no	down	mRNA	634.69	506.24	527.42	491.63	505.99	458.74	556.117	485.453333	ENOG410XYA7	S:Function unknown	------	------
BAB_RS26555	-	protein MgtC	0.805	-0.312886926	0.154657125	0.285254501	no	down	mRNA	6.01	7.18	7.82	6.49	4.81	4.97	7.00333	5.42333333	COG1285	S:Function unknown	GO:0009405;GO:0009405;GO:0016021;GO:0005886;GO:0016021;GO:00	BP:pathogenesis;BP:pathogenesis;CC:integral component of membrane;CC:plasma membrane;CC:integral component of membrane;CC:integral component of membrane;CC:membrane;CC:plasma membrane;
BAB_RS21500	-	cyclic pyranopterin monophosphate synthase MoaC	0.916	-0.127130006	0.154829812	0.285409172	no	down	mRNA	117.74	93.87	111.82	92.28	91.92	97.49	107.81	93.8966667	COG0315	H:Coenzyme transport and metabolism	GO:0006777;GO:0061799	BP:Mo-molybdopterin cofactor biosynthetic process;MF:cyclic pyranopterin monophosphate synthase activity;
BAB_RS22495	-	urease subunit gamma	0.932	-0.101278848	0.155016908	0.285590209	no	down	mRNA	524.4	459.96	466.98	395.91	436.46	445.45	483.78	425.94	COG0831	S:Function unknown	GO:0043419;GO:0043419;GO:0005737;GO:0005737;GO:0009039;GO:00	BP:urea catabolic process;BP:urea catabolic process;CC:cytoplasm;CC:cytoplasm;MF:urease activity;MF:nickel cation binding;MF:nickel cation binding;MF:urease activity;
BAB_RS20780	-	DNA polymerase III subunit delta'	0.93	-0.104691491	0.155710179	0.28670304	no	down	mRNA	31.1	28.71	32.68	26.21	27.16	27.7	30.83	27.0233333	COG0470	L:Replication, recombination and repair	GO:0006260;GO:0006260;GO:0003887;GO:0008408;GO:0043885;GO:00	BP:DNA replication;BP:DNA replication;MF:DNA-directed DNA polymerase activity;MF:3'-5' exonuclease activity;MF:carbon-monoxide dehydrogenas;MF:3'-5' exonuclease activity;MF:DNA-directed DNA polymerase activity;
BAB_RS24350	-	thiamine diphosphokinase	0.879	-0.186516131	0.156180669	0.28724012	no	down	mRNA	38.05	29.62	29.49	25.68	29.34	24.89	32.3867	26.6366667	COG1564	H:Coenzyme transport and metabolism	GO:0009229;GO:0006772;GO:0009229;GO:0006772;GO:0009229;GO:00	BP:thiamine diphosphate biosynthetic process;BP:thiamine metabolic process;BP:thiamine diphosphate biosynthetic process;BP:thiamine metabolic process;BP:thiamine diphosphate biosynthetic process;BP:thiamine metabolic process;MF:thiamine binding;MF:kinase activity;MF:thiamine diphosphokinase activity;MF:ATP binding;MF:glutathione-disulfide reductase activity;MF:thiamine binding;MF:kinase activity;MF:thiamine diphosphokinase activity;MF:ATP binding;MF:ATP binding;MF:kinase activity;MF:thiamine binding;MF:thiamine diphosphokinase activity;
BAB_RS18510	-	hypothetical protein	0.93	-0.104972684	0.156312752	0.287318577	no	down	mRNA	398.01	345.87	335.05	313.56	321.36	312.99	359.643	315.97	ENOG410XU3R;COG0438	S:Function unknown;M:Cell wall/membrane/envelo	------	------
BAB_RS20725	-	IS5/IS1182 family transposase	0.588	-0.765884873	0.157469522	0.289279345	no	down	mRNA	4.21	2.39	2.78	1.6	1.38	2.16	3.12667	1.71333333	COG3293	L:Replication, recombination and repair	GO:0006313;GO:0016021;GO:0003677;GO:0004803	BP:transposition, DNA-mediated;CC:integral component of membrane;MF:DNA binding;MF:transposase activity;
BAB_RS23810	rrf	-	0.774	-0.368953499	0.15769391	0.289526019	no	down	mRNA	118.88	136.47	192.6	144.06	118.11	91.55	149.317	117.906667	------	------	GO:0015977;GO:0006099;GO:0008964	BP:carbon fixation;BP:tricarboxylic acid cycle;MF:phosphoenolpyruvate carboxylase activity;
BAB_RS24800	rrf	-	0.774	-0.368953499	0.15769391	0.289526019	no	down	mRNA	118.88	136.47	192.6	144.06	118.11	91.55	149.317	117.906667	------	------	GO:0015977;GO:0006099;GO:0008964	BP:carbon fixation;BP:tricarboxylic acid cycle;MF:phosphoenolpyruvate carboxylase activity;
BAB_RS31440	rrf	-	0.774	-0.368953499	0.15769391	0.289526019	no	down	mRNA	118.88	136.47	192.6	144.06	118.11	91.55	149.317	117.906667	------	------	GO:0015977;GO:0006099;GO:0008964	BP:carbon fixation;BP:tricarboxylic acid cycle;MF:phosphoenolpyruvate carboxylase activity;
BAB_RS31395	-	class II aldolase family protein	0.851	-0.233310051	0.158919362	0.290283064	no	down	mRNA	21.6	27.38	28.13	17.34	24.6	20.63	25.7033	20.8566667	COG0235	S:Function unknown	GO:0008738	MF:L-fuculose-phosphate aldolase activity;
BAB_RS17765	-	glycerate kinase	0.918	-0.123700857	0.15875705	0.290316677	no	down	mRNA	23.75	23.5	31.23	22.59	22.62	22.48	26.16	22.5633333	COG2379	G:Carbohydrate transport and metabolism	GO:0016618;GO:0016301;GO:0016301	MF:hydroxypyruvate reductase activity;MF:kinase activity;MF:kinase activity;
BAB_RS33100	-	hypothetical protein	0.699	-0.517535142	0.160011924	0.291946798	no	down	mRNA	10.52	10.71	12.5	11.42	4.11	7.81	11.2433	7.78	------	------	GO:0016787	MF:hydrolase activity;
BAB_RS21425	-	dihydrolipoyl dehydrogenase	0.947	-0.078445223	0.161796492	0.294867912	no	down	mRNA	247.74	241.76	257.24	210.52	224.99	231.19	248.913	222.233333	COG1249	H:Coenzyme transport and metabolism	GO:0045454;GO:0045454;GO:0045454;GO:0005623;GO:0005623;GO:00	BP:cell redox homeostasis;BP:cell redox homeostasis;BP:cell redox homeostasis;CC:cell;CC:cell;CC:cell;MF:dihydrolipoyl dehydrogenase activity;MF:electron carrier activity;MF:flavin adenine dinucleotide binding;MF:methyltransferase activity;MF:flavin adenine dinucleotide binding;MF:dihydrolipoyl dehydrogenase activity;MF:electron carrier activity;MF:dihydrolipoyl dehydrogenase activity;MF:electron carrier activity;MF:flavin adenine dinucleotide binding;
BAB_RS33495	-	hypothetical protein	0.716	-0.482414298	0.163490353	0.297448756	no	down	mRNA	74.64	93.79	52.12	54.59	58.25	42.3	73.5167	51.7133333	------	------	------	------
BAB_RS30335	-	5'-methylthioadenosine/S-adenosylhomocysteine nucleosidase	0.929	-0.105603437	0.163829092	0.297896362	no	down	mRNA	92.99	81.81	86.84	80.86	74.36	74.57	87.2133	76.5966667	COG0775	F:Nucleotide transport and metabolism	GO:0009116;GO:0009116;GO:0003824;GO:0003824;GO:0008930	BP:nucleoside metabolic process;BP:nucleoside metabolic process;MF:catalytic activity;MF:catalytic activity;MF:methylthioadenosine nucleosidase activity;
BAB_RS30340	-	PaaI family thioesterase	0.927	-0.108628044	0.164096494	0.298213821	no	down	mRNA	93.43	88.32	101.93	77.47	83.09	89.06	94.56	83.2066667	COG2050	F:Nucleotide transport and metabolism	------	------
BAB_RS33065	-	hypothetical protein	0.605	-0.725337759	0.164610739	0.298979257	no	down	mRNA	8.36	12.91	10.19	3.49	9.12	6.39	10.4867	6.33333333	------	------	------	------
BAB_RS31285	-	acyl-CoA dehydrogenase	0.909	-0.13820696	0.165137432	0.299428086	no	down	mRNA	48.58	37.57	49.25	39.81	40.97	34.54	45.1333	38.44	COG1960	C:Energy production and conversion	GO:0016627;GO:0050660;GO:0050660;GO:0016627	MF:oxidoreductase activity, acting on the CH-CH group of donors;MF:flavin adenine dinucleotide binding;MF:flavin adenine dinucleotide binding;MF:oxidoreductase activity, acting on the CH-CH group of donors;
BAB_RS21160	-	membrane protein insertion efficiency factor YidD	0.9	-0.152446885	0.165425886	0.299444153	no	down	mRNA	159.36	156.64	160.46	153.51	137.54	123.89	158.82	138.313333	COG0759	S:Function unknown	GO:0005886	CC:plasma membrane;
BAB_RS17680	-	hypothetical protein	0.651	-0.61935689	0.165094162	0.299518658	no	down	mRNA	11.31	7.18	10.2	7.74	3.06	7.76	9.56333	6.18666667	COG0534	E:Amino acid transport and metabolism	GO:0006811;GO:0016021;GO:0016021;GO:0016020;GO:0005886;GO:00	BP:ion transport;CC:integral component of membrane;CC:integral component of membrane;CC:membrane;CC:plasma membrane;MF:antiporter activity;MF:drug transmembrane transporter activity;MF:antiporter activity;MF:drug transmembrane transporter activity;
BAB_RS31770	-	GntR family transcriptional regulator	0.827	-0.274498564	0.165418001	0.299598667	no	down	mRNA	19.83	16.16	21.95	10.86	15.79	18.75	19.3133	15.1333333	COG2188	S:Function unknown	GO:0006351;GO:0006351;GO:0003700;GO:0003677;GO:0003677;GO:00	BP:transcription, DNA-templated;BP:transcription, DNA-templated;MF:transcription factor activity, sequence-specific DNA binding;MF:DNA binding;MF:DNA binding;MF:transcription factor activity, sequence-specific DNA binding;
BAB_RS18990	-	hypothetical protein	0.938	-0.093020959	0.165067144	0.299638833	no	down	mRNA	34.72	33.34	35.56	30.73	28.69	32.04	34.54	30.4866667	COG3306;COG3306	O:Posttranslational modification, protein turnover,	GO:0016021;GO:0016021;GO:0016740	CC:integral component of membrane;CC:integral component of membrane;MF:transferase activity;
BAB_RS29150	-	branched-chain amino acid ABC transporter permease	0.845	-0.242868013	0.166896314	0.301088073	no	down	mRNA	7.16	7.26	8.85	5.1	6.96	6.57	7.75667	6.21	COG0559	F:Nucleotide transport and metabolism	GO:0016021;GO:0005886;GO:0022857	CC:integral component of membrane;CC:plasma membrane;MF:transmembrane transporter activity;
BAB_RS20960	-	2-amino-4-hydroxy-6-hydroxymethyldihydropteridine diphosphokinase	0.913	-0.131868652	0.167423987	0.301870522	no	down	mRNA	23.64	20.2	23.94	18.75	20.92	18.43	22.5933	19.3666667	COG0801	H:Coenzyme transport and metabolism	GO:0009396;GO:0003848;GO:0016301;GO:0034015	BP:folic acid-containing compound biosynthetic process;MF:2-amino-4-hydroxy-6-hydroxymethyldihydropteridine diphosphokinase activity;MF:kinase activity;MF:L-ribulose-5-phosphate 3-epimerase activity;
BAB_RS18850	-	hypothetical protein	0.599	-0.740091888	0.167698971	0.302196744	no	down	mRNA	3.02	4.24	6.09	1.77	4.15	1.91	4.45	2.61	ENOG41129B7	S:Function unknown	GO:0016021	CC:integral component of membrane;
BAB_RS29275	-	hypothetical protein	0.797	-0.327959993	0.170124952	0.30554023	no	down	mRNA	29.45	29.48	41.09	22.79	21.67	34.53	33.34	26.33	------	------	GO:0016021	CC:integral component of membrane;
BAB_RS25380	-	hypothetical protein	0.745	-0.424479126	0.170043796	0.305565278	no	down	mRNA	209.32	242.13	235.38	206.02	146.58	196.09	228.943	182.896667	COG5567	M:Cell wall/membrane/envelope biogenesis	------	------
BAB_RS22825	-	D-alanine--D-alanine ligase	0.94	-0.089390192	0.170460978	0.305972694	no	down	mRNA	161.97	149.98	173.99	133.34	151.02	146.48	161.98	143.613333	COG1181	M:Cell wall/membrane/envelope biogenesis	GO:0071555;GO:0009252;GO:0008360;GO:0071555;GO:0008360;GO:00	BP:cell wall organization;BP:peptidoglycan biosynthetic process;BP:regulation of cell shape;BP:cell wall organization;BP:regulation of cell shape;BP:peptidoglycan biosynthetic process;BP:cell wall organization;BP:regulation of cell shape;BP:peptidoglycan biosynthetic process;BP:cell wall organization;BP:peptidoglycan biosynthetic process;BP:regulation of cell shape;CC:cytoplasm;CC:cytoplasm;CC:cytoplasm;CC:cytoplasm;MF:ATP binding;MF:D-alanine-D-alanine ligase activity;MF:lyase activity;MF:metal ion binding;MF:lyase activity;MF:D-alanine-D-alanine ligase activity;MF:metal ion binding;MF:ATP binding;MF:D-alanine-D-alanine ligase activity;MF:metal ion binding;MF:ATP binding;MF:ATP binding;MF:D-alanine-D-alanine ligase activity;MF:metal ion binding;
BAB_RS33090	-	hypothetical protein	0.865	-0.210051626	0.170899942	0.306418255	no	down	mRNA	22.52	22.1	20.91	20.15	16.86	16.88	21.8433	17.9633333	------	------	GO:0016021	CC:integral component of membrane;
BAB_RS21715	-	methylmalonyl-CoA mutase	0.908	-0.139760945	0.171286662	0.306940349	no	down	mRNA	18.34	17.46	20.04	13.58	17.8	16.26	18.6133	15.88	COG2185	I:Lipid transport and metabolism	GO:0008152;GO:0031419;GO:0046872;GO:0004494;GO:0008946;GO:00	BP:metabolic process;MF:cobalamin binding;MF:metal ion binding;MF:methylmalonyl-CoA mutase activity;MF:oligonucleotidase activity;MF:cobalamin binding;MF:methylmalonyl-CoA mutase activity;MF:metal ion binding;
BAB_RS33515	-	hypothetical protein	0.649	-0.623871678	0.171913242	0.307891442	no	down	mRNA	6.11	6.4	12.92	6.49	6.06	4.48	8.47667	5.67666667	COG3395	S:Function unknown	------	------
BAB_RS19835	-	hypothetical protein	0.899	-0.153814866	0.172067331	0.30799573	no	down	mRNA	17.88	18.51	20.74	18.19	16.04	14.27	19.0433	16.1666667	ENOG410XX30	S:Function unknown	------	------
BAB_RS20340	-	DUF2155 domain-containing protein	0.94	-0.089241726	0.172231133	0.308117278	no	down	mRNA	162.37	140.87	152.44	132.61	130.41	141.04	151.893	134.686667	COG4765	S:Function unknown	GO:0016787	MF:hydrolase activity;
BAB_RS18300	-	SURF1 family protein	0.787	-0.344729906	0.173032838	0.309035302	no	down	mRNA	5.99	7.49	10.27	6.04	4.63	7.6	7.91667	6.09	COG3346	S:Function unknown	GO:0016021;GO:0005886	CC:integral component of membrane;CC:plasma membrane;
BAB_RS19140	-	NYN domain-containing protein	0.929	-0.106264996	0.172961352	0.309079434	no	down	mRNA	203	171	183.91	172.15	150.79	166.63	185.97	163.19	COG1432	S:Function unknown	------	------
BAB_RS29830	-	cation:proton antiporter	0.889	-0.169247761	0.173344347	0.309247856	no	down	mRNA	67.42	66.29	69.15	55.43	56.21	63.64	67.62	58.4266667	COG1320	P:Inorganic ion transport and metabolism	GO:0016021;GO:0005451	CC:integral component of membrane;MF:monovalent catio;
BAB_RS32150	-	DNA mismatch repair protein MutT	0.936	-0.0950579	0.173667395	0.309480499	no	down	mRNA	69.57	61.87	70.24	55.71	59.13	63.09	67.2267	59.31	COG0494	L:Replication, recombination and repair	GO:0016787	MF:hydrolase activity;
BAB_RS32635	-	hypothetical protein	0.707	-0.500659126	0.173609888	0.309549706	no	down	mRNA	70.38	68.99	33.74	50.76	33.17	31.37	57.7033	38.4333333	------	------	------	------
BAB_RS23155	-	TrkH family potassium uptake protein	0.922	-0.116906345	0.173994267	0.30989112	no	down	mRNA	16.48	15.1	16.5	13.25	14.43	14.01	16.0267	13.8966667	COG0168	P:Inorganic ion transport and metabolism	GO:0016021;GO:0005886;GO:0016021;GO:0005886;GO:0015379;GO:00	CC:integral component of membrane;CC:plasma membrane;CC:integral component of membrane;CC:plasma membrane;MF:potassiu;MF:metal ion binding;MF:metal ion binding;MF:potassiu;
BAB_RS19620	-	ribonuclease D	0.947	-0.079197916	0.174455453	0.310196664	no	down	mRNA	97.78	93.62	103.22	90.67	84.3	87.77	98.2067	87.58	COG0349;COG0349	F:Nucleotide transport and metabolism;P:Inorganic	GO:0042780;GO:0042780;GO:0005737;GO:0005737;GO:0008408;GO:00	BP:tRNA 3'-end processing;BP:tRNA 3'-end processing;CC:cytoplasm;CC:cytoplasm;MF:3'-5' exonuclease activity;MF:nucleic acid binding;MF:nucleotide binding;MF:ribonuclease D activity;MF:3'-5' exonuclease activity;MF:ribonuclease D activity;MF:nucleotide binding;MF:nucleic acid binding;
BAB_RS31525	-	hypothetical protein	0.532	-0.910113015	0.174400185	0.310270097	no	down	mRNA	3.1	1.31	2.19	1.1	0.62	1.58	2.2	1.1	------	------	------	------
BAB_RS22625	-	pyridoxine 5'-phosphate synthase	0.926	-0.111581341	0.174597866	0.310278177	no	down	mRNA	111.01	106.72	103.72	101.39	92.23	87.92	107.15	93.8466667	COG0854	H:Coenzyme transport and metabolism	GO:0008615;GO:0008615;GO:0005737;GO:0005737;GO:0033856;GO:00	BP:pyridoxine biosynthetic process;BP:pyridoxine biosynthetic process;CC:cytoplasm;CC:cytoplasm;MF:pyridoxine 5'-phosphate synthase activity;MF:pyridoxine 5'-phosphate synthase activity;
BAB_RS18475	-	-	0.799	-0.323907247	0.176006304	0.311403224	no	down	mRNA	93.98	122.58	110.89	113.94	74.6	77.62	109.15	88.72	------	------	------	------
BAB_RS31260	-	ABC transporter substrate-binding protein	0.891	-0.16670001	0.175934962	0.311448504	no	down	mRNA	7.84	7.77	7.71	6.04	6.42	7.14	7.77333	6.53333333	COG0747;ENOG410XQF5	S:Function unknown;	------	------
BAB_RS31735	-	amidohydrolase	0.771	-0.374573468	0.176508822	0.31194876	no	down	mRNA	1.21	1.27	1.13	0.7	0.99	0.92	1.20333	0.87	COG1574	S:Function unknown	GO:0016810	MF:hydrolase activity, acting on carbon-nitroge;
BAB_RS23290	-	aminopeptidase P family protein	0.943	-0.084022296	0.17642919	0.31197963	no	down	mRNA	71.57	68.48	73.62	60.97	64.59	64.63	71.2233	63.3966667	COG0006	S:Function unknown	GO:0016787;GO:0016787;GO:0102009	MF:hydrolase activity;MF:hydrolase activity;MF:proline dipeptidase activity;
BAB_RS25770	-	protoporphyrinogen oxidase HemJ	0.937	-0.094512939	0.176852043	0.312383516	no	down	mRNA	142.83	133.99	133.04	114.61	127.56	120.42	136.62	120.863333	COG1981	S:Function unknown	GO:0016021	CC:integral component of membrane;
BAB_RS23145	-	LysR family transcriptional regulator	0.951	-0.072688295	0.177128839	0.312700527	no	down	mRNA	76.76	73.25	79.23	69.1	69.84	66.1	76.4133	68.3466667	COG0583	S:Function unknown	GO:0006351;GO:0003700;GO:0003677	BP:transcription, DNA-templated;MF:transcription factor activity, sequence-specific DNA binding;MF:DNA binding;

BAB_RS17700	-	DNA-binding response regulator	0.869	-0.203288582	0.177661491	0.313296581	no	down	mRNA	19.9	19.57	20.17	19.44	14.12	15.66	19.88	16.4066667	COG2197	S:Function unknown	GO:0000160;GO:0006355;GO:0006351;GO:0000160;GO:0006355;GO:00	BP:phosphorelay signal transduction system;BP:regulation of transcription, DNA-templated;BP:transcription, DNA-templated;BP:phosphorelay signal transduction system;BP:regulation of transcription, DNA-templated;BP:phosphorelay signal transduction system;BP:transcription, DNA-templated;BP:regulation of transcription, DNA-templated;CC:intracellular;CC:intracellular;MF:DNA binding;MF:DNA binding;
BAB_RS17825	-	salicylaldehyde dehydrogenase	0.912	-0.132368294	0.177919981	0.313580306	no	down	mRNA	28.84	26.55	24.94	21.03	23.89	23.99	26.7767	22.97	COG1012	O:Posttranslational modification, protein turnover,	GO:0016620	MF:oxidoreductase activity, acting on the aldehyde or oxo group of donors, NAD or NADP as acceptor;
BAB_RS20030	-	glutathione S-transferase family protein	0.944	-0.083106632	0.178174361	0.313684506	no	down	mRNA	357.08	306.12	327.83	291.3	288.53	304.61	330.343	294.813333	COG0625	S:Function unknown	GO:0016740;GO:0016740;GO:0016853	MF:transferase activity;MF:transferase activity;MF:isomerase activity;
BAB_RS18135	-	class I SAM-dependent RNA methyltransferase	0.933	-0.100439968	0.180019186	0.316758842	no	down	mRNA	28.41	25.81	30.6	24.72	26.14	23.51	28.2733	24.79	COG2265	J:Translation, ribosomal structure and biogenesis	GO:0006396;GO:0006396;GO:0008173;GO:0008173;GO:0046872;GO:00	BP:RNA processing;BP:RNA processing;MF:RNA methyltransferase activity;MF:RNA methyltransferase activity;MF:metal ion binding;MF:4 iron, 4 sulfur cluster binding;
BAB_RS24410	-	acetyl-CoA C-acetyltransferase	0.948	-0.076919499	0.180637514	0.31767287	no	down	mRNA	105.03	104.62	110.88	94.42	95.83	96.49	106.843	95.58	COG0183	I:Lipid transport and metabolism	GO:0008152;GO:0008152;GO:0003985;GO:0016747;GO:0016747	BP:metabolic process;BP:metabolic process;MF:acetyl-CoA C-acetyltransferase activity;MF:transferase activity, transferring acyl groups other than amino-acyl groups;MF:transferase activity, transferring acyl groups other than amino-acyl groups;
BAB_RS23975	-	hypothetical protein	0.665	-0.589112699	0.18125871	0.318068943	no	down	mRNA	1.94	1.93	1.25	1.06	1.39	0.62	1.70667	1.02333333	COG3698	S:Function unknown	------	------
BAB_RS31725	-	APC family permease	0.768	-0.380707515	0.181161535	0.318072138	no	down	mRNA	1.79	2.06	2.4	1.55	1.96	0.99	2.08333	1.5	COG0531	S:Function unknown	GO:0016021;GO:0022857	CC:integral component of membrane;MF:transmembrane transporter activity;
BAB_RS25480	-	cyclic nucleotide-binding domain-containing protein	0.932	-0.102218153	0.181607874	0.318507696	no	down	mRNA	108.12	110.83	129.74	99.07	106.94	101.71	116.23	102.573333	COG0664	M:Cell wall/membrane/envelope biogenesis	GO:0016301	MF:kinase activity;
BAB_RS25640	-	NAD(P)-dependent oxidoreductase	0.932	-0.1019085	0.182414421	0.3197477	no	down	mRNA	34.78	32.06	37.42	28.56	33.43	29.28	34.7533	30.4233333	ENOG410XQEM	S:Function unknown	GO:0047936;GO:0004316;GO:0102132;GO:0102131;GO:0102131;GO:01	MF:glucose 1-dehydrogenase [NA;MF:3-oxoacyl-[acyl-carrier-protein] reductas;MF:3-oxo-pimeloyl-[acp] methyl ester reductase activity;MF:old GO;MF:old GO;MF:3-oxo-pimeloyl-[acp] methyl ester reductase activity;MF:3-oxoacyl-[acyl-carrier-protein] reductas;
BAB_RS32010	-	hypothetical protein	0.674	-0.570177734	0.18302478	0.32064265	no	down	mRNA	4.9	1.64	3.99	1.73	2.05	2.78	3.51	2.18666667	COG5502	S:Function unknown	------	------
BAB_RS20155	-	adenosylcobinamide-GDP ribazoletransferase	0.89	-0.168162112	0.183680043	0.321615246	no	down	mRNA	14.51	14.62	15.79	11.36	13.56	12.92	14.9733	12.6133333	COG0368	H:Coenzyme transport and metabolism	GO:0009236;GO:0016021;GO:0005886;GO:0016787;GO:0008818;GO:00	BP:cobalamin biosynthetic process;CC:integral component of membrane;CC:plasma membrane;MF:hydrolase activity;MF:cobalamin 5'-phosphate synthase activity;MF:adenosylcobinamide-GDP ribazoletransferase activity;
BAB_RS18730	-	DUF853 domain-containing protein	0.943	-0.085416602	0.184165447	0.322289532	no	down	mRNA	95.55	87.56	92.43	76.62	82.69	85.29	91.8467	81.5333333	COG0433	S:Function unknown	GO:0005524	MF:ATP binding;
BAB_RS29585	-	hypothetical protein	0.777	-0.364766874	0.184420675	0.322384998	no	down	mRNA	19.24	18.64	19.95	19.21	9.37	15.31	19.2767	14.63	------	------	GO:0016021	CC:integral component of membrane;
BAB_RS16435	-	sulfate ABC transporter permease subunit CysW	0.91	-0.136494889	0.184809839	0.32288962	no	down	mRNA	24.31	24	26.67	22.43	19.85	22.44	24.9933	21.5733333	COG4208	P:Inorganic ion transport and metabolism	GO:0016021;GO:0016021;GO:0015419;GO:0015419	CC:integral component of membrane;CC:integral component of membrane;MF:ATPase-coupled sulfate transmembrane transporter activity;MF:ATPase-coupled sulfate transmembrane transporter activity;
BAB_RS24140	-	thiamine phosphate synthase	0.929	-0.105761669	0.185996376	0.324257383	no	down	mRNA	122.6	118.24	112.31	106.2	108.01	95.98	117.717	103.396667	COG0352	S:Function unknown	GO:0003824;GO:0102250;GO:0004645;GO:0102499;GO:0003824;GO:00	MF:catalytic activity;MF:old GO;MF:phosphorylase activity;MF:old GO;MF:catalytic activity;MF:thiamine-phosphate diphosphorylase activity;MF:catalytic activity;
BAB_RS33425	-	hypothetical protein	0.891	-0.16625205	0.186423793	0.324650215	no	down	mRNA	21.03	16.45	19.6	14.41	16.49	16.9	19.0267	15.9333333	------	------	GO:0016740	MF:transferase activity;
BAB_RS21385	-	two-component sensor histidine kinase	0.935	-0.096619854	0.186864848	0.325065921	no	down	mRNA	115.33	117.41	117.25	103.6	109.7	95.77	116.663	103.023333	COG3852	T:Signal transduction mechanisms	GO:0005622;GO:0005622;GO:0000155;GO:0005524;GO:0005524;GO:00	CC:intracellular;CC:intracellular;MF:phosphorelay sensor kinase activity;MF:ATP binding;MF:ATP binding;MF:phosphorelay sensor kinase activity;
BAB_RS20255	-	protein-L-isoaspartate(D-aspartate) O-methyltransferase	0.924	-0.113871159	0.188125784	0.326905433	no	down	mRNA	62.78	70.35	68.21	59.37	59.38	58.2	67.1133	58.9833333	COG2518	O:Posttranslational modification, protein turnover,	GO:0004719	MF:protein-L-isoaspartat;
BAB_RS17545	-	GlxA family transcriptional regulator	0.903	-0.147719885	0.188911645	0.327916323	no	down	mRNA	13.7	12.47	15.19	11.86	11.4	12.03	13.7867	11.7633333	COG4977	K:Transcription	GO:0006351;GO:0003700;GO:0043565	BP:transcription, DNA-templated;MF:transcription factor activity, sequence-specific DNA binding;MF:sequence-specific DNA binding;
BAB_RS18630	-	ABC transporter substrate-binding protein	0.911	-0.134284401	0.188871135	0.328023219	no	down	mRNA	34.53	31.8	30.72	28.55	29.43	25.2	32.35	27.7266667	COG1879	G:Carbohydrate transport and metabolism	------	------
BAB_RS32015	-	hypothetical protein	0.864	-0.210554971	0.189315138	0.328262028	no	down	mRNA	28.44	21.48	30.48	17.98	22.18	25.59	26.8	21.9166667	------	------	------	------
BAB_RS20330	-	N-acetyltransferase	0.928	-0.108138903	0.189614146	0.328426012	no	down	mRNA	84.31	72.57	82.27	74.87	66.52	68.66	79.7167	70.0166667	ENOG4111M30	S:Function unknown	GO:0016740;GO:0016740;GO:0016746	MF:transferase activity;MF:transferase activity;MF:transferase activity, transferring acyl groups;
BAB_RS30735	-	sugar ABC transporter permease	0.908	-0.139568257	0.18982024	0.328605836	no	down	mRNA	20.69	16.71	17.65	15.44	16.4	14.94	18.35	15.5933333	COG4214	G:Carbohydrate transport and metabolism	GO:0008643;GO:0005886;GO:0016021;GO:0016021;GO:0005886;GO:00	BP:carbohydrate transport;CC:plasma membrane;CC:integral component of membrane;CC:integral component of membrane;CC:plasma membrane;MF:transmembrane transporter activity;MF:transmembrane transporter activity;
BAB_RS18930	-	hypothetical protein	0.917	-0.125520467	0.192091845	0.332180355	no	down	mRNA	701.98	794.26	737.06	667.17	626.37	719.58	744.433	671.04	ENOG410Y80C	S:Function unknown	------	------
BAB_RS15955	-	molybdopterin-synthase adenylyltransferase MoeB	0.939	-0.090819499	0.192358964	0.332463341	no	down	mRNA	487.52	520.75	546.83	433.13	493.55	456.06	518.367	460.913333	COG0476	H:Coenzyme transport and metabolism	GO:0008641	MF:small protein activating enzyme activity;
BAB_RS25590	-	shikimate kinase	0.92	-0.120437172	0.192676917	0.332476334	no	down	mRNA	79.55	68.64	71.25	57.53	66.99	66.44	73.1467	63.6533333	COG0703	E:Amino acid transport and metabolism	GO:0009423;GO:0009073;GO:0009073;GO:0009423;GO:0009423;GO:00	BP:chorismate biosynthetic process;BP:aromatic amino acid family biosynthetic process;BP:aromatic amino acid family biosynthetic process;BP:chorismate biosynthetic process;BP:chorismate biosynthetic process;BP:aromatic amino acid family biosynthetic process;CC:cytoplasm;CC:cytoplasm;CC:cytoplasm;MF:magnesium ion binding;MF:shikimate kinase activity;MF:ATP binding;MF:ATP binding;MF:magnesium ion binding;MF:shikimate kinase activity;MF:magnesium ion binding;MF:shikimate kinase activity;MF:metal ion binding;MF:ATP binding;
BAB_RS29175	-	4-carboxymuconolactone decarboxylase	0.9	-0.151640446	0.195027475	0.335810974	no	down	mRNA	80.87	84.71	86.64	63.66	79.04	76.17	84.0733	72.9566667	COG0599	S:Function unknown	GO:0016787;GO:0016829;GO:0051920;GO:0047575;GO:0051920	MF:hydrolase activity;MF:lyase activity;MF:peroxiredoxin activity;MF:4-carboxymuconolactone decarboxylase activity;MF:peroxiredoxin activity;
BAB_RS17785	-	cytochrome c oxidase accessory protein CcoG	0.954	-0.067499952	0.196617454	0.338186231	no	down	mRNA	266.46	255.93	267.04	233.76	235.02	241.21	263.143	236.663333	COG0348	C:Energy production and conversion	GO:0055114;GO:0016021	BP:oxidation-reduction process;CC:integral component of membrane;
BAB_RS26105	-	AAA family ATPase	0.91	-0.136449748	0.197880262	0.339268561	no	down	mRNA	18.17	18.23	24.82	16.97	18.36	17.18	20.4067	17.5033333	COG0606	O:Posttranslational modification, protein turnover,	------	------
BAB_RS16765	-	DUF2849 domain-containing protein	0.758	-0.400431017	0.197878246	0.339446238	no	down	mRNA	20.71	18.71	20.38	18.02	11.68	15.77	19.9333	15.1566667	ENOG41127DV;ENOG41128DP	S:Function unknown;	------	------
BAB_RS25935	-	bifunctional tRNA (adenosine(37)-N6)-threonylcarbamoyltransferase complex ATPase subunit type 1 TsaE/phosphotransferase	0.938	-0.092497184	0.197726493	0.339548488	no	down	mRNA	53.72	50.48	50.66	44.32	44.43	48.15	51.62	45.6333333	COG3178;COG1208	S:Function unknown;M:Cell wall/membrane/envel	GO:0002949;GO:0002949;GO:0016740	BP:tRNA threonylcarbamoyladenosine modification;BP:tRNA threonylcarbamoyladenosine modification;MF:transferase activity;
BAB_RS21125	-	UDP-glucose 4-epimerase GalE	0.901	-0.149742956	0.197573221	0.339648347	no	down	mRNA	36.8	28.03	28.71	26.32	25.03	27.71	31.18	26.3533333	COG1087	M:Cell wall/membrane/envelope biogenesis	GO:0006012;GO:0006012;GO:0003978;GO:0003978	BP:galactose metabolic process;BP:galactose metabolic process;MF:UDP-glucose 4-epimerase activity;MF:UDP-glucose 4-epimerase activity;
BAB_RS31125	-	5%2C6-dimethylbenzimidazole synthase	0.898	-0.154746865	0.20037255	0.343358402	no	down	mRNA	21.46	19.15	21.13	16.14	17.25	19.09	20.58	17.4933333	COG0778	G:Carbohydrate transport and metabolism	GO:0004654;GO:0016491;GO:0016491	MF:polyribonucleotide nucleotidyltransferase activity;MF:oxidoreductase activity;MF:oxidoreductase activity;
BAB_RS30240	-	NAD-dependent succinate-semialdehyde dehydrogenase	0.923	-0.115697244	0.201290371	0.344196892	no	down	mRNA	22.06	21.66	25.89	20.89	21.07	18.6	23.2033	20.1866667	COG1012	S:Function unknown	GO:0016620	MF:oxidoreductase activity, acting on the aldehyde or oxo group of donors, NAD or NADP as acceptor;
BAB_RS26190	-	tRNA (N6-isopentenyl adenosine(37)-C2)-methylthiotransferase MiaB	0.947	-0.0779361	0.202253009	0.345658998	no	down	mRNA	120.24	109.1	127.78	111.38	102.16	104.94	119.04	106.16	COG0621	S:Function unknown	GO:0006400;GO:0005737;GO:0016740;GO:0046872;GO:0051539	BP:tRNA modification;CC:cytoplasm;MF:transferase activity;MF:metal ion binding;MF:4 iron, 4 sulfur cluster binding;
BAB_RS28520	-	ABC transporter substrate-binding protein	0.95	-0.074321507	0.203515689	0.346894382	no	down	mRNA	62.08	59.12	65.31	54.86	57.66	54.23	62.17	55.5833333	COG0747	S:Function unknown	GO:0055085;GO:0043190	BP:transmembrane transport;CC:ATP-binding cassett;
BAB_RS16620	-	lipoprotein signal peptidase	0.891	-0.166357891	0.203388802	0.34704632	no	down	mRNA	51.85	44.8	46.08	39.15	46.71	33.84	47.5767	39.9	COG0597;COG0597	M:Cell wall/membrane/envelope biogenesis;U:Intra	GO:0016021;GO:0005886;GO:0016021;GO:0005886;GO:0016021;GO:00	CC:integral component of membrane;CC:plasma membrane;CC:integral component of membrane;CC:plasma membrane;CC:integral component of membrane;CC:plasma membrane;MF:aspartic-type endopeptidase activity;MF:aspartic-type endopeptidase activity;MF:aspartic-type endopeptidase activity;MF:lyase activity;
BAB_RS32225	-	hypothetical protein	0.832	-0.265381409	0.203340039	0.347147474	no	down	mRNA	336.47	379.57	412.45	371.07	336.49	254.71	376.163	320.756667	------	------	------	------
BAB_RS18660	-	AraC family transcriptional regulator	0.894	-0.162349561	0.203864771	0.347305148	no	down	mRNA	22.35	25.64	24.45	21.21	19.07	21.56	24.1467	20.6133333	ENOG4111NRR;COG2207	K:Transcription;	GO:0006351;GO:0003700;GO:0043565	BP:transcription, DNA-templated;MF:transcription factor activity, sequence-specific DNA binding;MF:sequence-specific DNA binding;
BAB_RS16645	-	signal peptide peptidase SppA	0.953	-0.069752288	0.204459059	0.347764402	no	down	mRNA	62.9	59.35	68.54	57.34	56.71	56.92	63.5967	56.99	COG0616;COG0616	O:Posttranslational modification, protein turnover,	GO:0016021;GO:0016021;GO:0008233;GO:0016491;GO:0008233	CC:integral component of membrane;CC:integral component of membrane;MF:peptidase activity;MF:oxidoreductase activity;MF:peptidase activity;
BAB_RS24825	-	-	0.794	-0.333240171	0.205789906	0.349657837	no	down	mRNA	28039.8	45221.4	30053.7	30416.84	17914.23	29599.65	34438.3	25976.9067	COG0048;ENOG4111UA7;ENOG4111VRG	J:Translation, ribosomal structure and biogenesis;	------	------
BAB_RS32095	-	ligand-binding protein SH3	0.862	-0.214990511	0.207290089	0.35164892	no	down	mRNA	13.75	10.67	12.91	8.75	10.88	10.64	12.4433	10.09	ENOG41128S9	S:Function unknown	------	------
BAB_RS32100	-	abortive infection protein	0.652	-0.618142115	0.207245356	0.351758757	no	down	mRNA	5.06	2.98	3.36	1.51	1.49	4.05	3.8	2.35	ENOG4111HWJ	S:Function unknown	------	------
BAB_RS31610	-	hypothetical protein	0.712	-0.490172301	0.207872328	0.352450549	no	down	mRNA	1.64	1.5	2.24	1.27	1.07	1.36	1.79333	1.23333333	ENOG410ZP54;ENOG4112BMU	S:Function unknown;	------	------
BAB_RS30245	-	choline dehydrogenase	0.87	-0.201454935	0.208154584	0.352742973	no	down	mRNA	4.46	3.81	5.58	3.87	3.59	3.92	4.61667	3.79333333	COG2303	S:Function unknown	GO:0050660;GO:0016614	MF:flavin adenine dinucleotide binding;MF:oxidoreductase activity, acting on CH-OH group of donors;
BAB_RS32270	-	IS3 family transposase	0.85	-0.234360812	0.208588771	0.35310628	no	down	mRNA	13.34	8.82	12.95	8.43	11.24	8.07	11.7033	9.24666667	COG2801	L:Replication, recombination and repair	GO:0015074;GO:0003676	BP:DNA integration;MF:nucleic acid binding;
BAB_RS18975	-	DNA-binding response regulator	0.932	-0.101570055	0.209031175	0.35366886	no	down	mRNA	59.63	53.81	64.5	48.99	55.36	52.38	59.3133	52.2433333	COG0745	T:Signal transduction mechanisms	GO:0000160;GO:0006351;GO:0006355;GO:0000160;GO:0006355;GO:00	BP:phosphorelay signal transduction system;BP:transcription, DNA-templated;BP:regulation of transcription, DNA-templated;BP:phosphorelay signal transduction system;BP:regulation of transcription, DNA-templated;BP:transcription, DNA-templated;BP:phosphorelay signal transduction system;BP:transcription, DNA-templated;BP:regulation of transcription, DNA-templated;BP:phosphorelay signal transduction system;BP:regulation of transcription, DNA-templated;BP:transcription, DNA-templated;CC:intracellular;CC:intracellular;CC:intracellular;CC:intracellular;MF:DNA binding;MF:DNA binding;MF:DNA binding;
BAB_RS29245	-	FAD-binding oxidoreductase	0.879	-0.186412708	0.209601991	0.354447998	no	down	mRNA	7.14	5.88	7.11	5.24	6.26	5.03	6.71	5.51	ENOG410XPGC	C:Energy production and conversion	GO:0016491	MF:oxidoreductase activity;
BAB_RS24820	-	-	0.854	-0.228112098	0.209839707	0.354663324	no	down	mRNA	129.93	136.12	183.29	147.11	116.55	116.11	149.78	126.59	------	------	------	------
BAB_RS31460	-	-	0.854	-0.228112098	0.209839707	0.354663324	no	down	mRNA	129.93	136.12	183.29	147.11	116.55	116.11	149.78	126.59	------	------	------	------
BAB_RS24855	-	peptide chain release factor 1	0.951	-0.073096768	0.210083675	0.354702494	no	down	mRNA	275.25	251.99	258.55	241.9	233.26	229.8	261.93	234.986667	COG0216	J:Translation, ribosomal structure and biogenesis	GO:0005737;GO:0005737;GO:0016149;GO:0016149	CC:cytoplasm;CC:cytoplasm;MF:translation release factor activity, codon specific;MF:translation release factor activity, codon specific;
BAB_RS31750	-	branched-chain amino acid ABC transporter ATP-binding protein	0.718	-0.478275581	0.210285668	0.354857064	no	down	mRNA	0.77	0.7	1.75	0.64	0.82	0.77	1.07333	0.74333333	COG0411	E:Amino acid transport and metabolism	GO:0016021;GO:0005886;GO:0016021;GO:0005886;GO:0005524;GO:00	CC:integral component of membrane;CC:plasma membrane;CC:integral component of membrane;CC:plasma membrane;MF:ATP binding;MF:ATPase activity;MF:transmembrane transporter activity;MF:ATP binding;MF:transmembrane transporter activity;MF:ATPase activity;
BAB_RS33260	-	hypothetical protein	0.838	-0.254537685	0.210657182	0.355297388	no	down	mRNA	30.9	17.17	26.06	20.6	17.43	20.54	24.71	19.5233333	ENOG4111T83	S:Function unknown	------	------
BAB_RS24805	-	-	0.744	-0.426622809	0.216307124	0.362542927	no	down	mRNA	21265.6	49321.5	21297	24267.28	17704.35	23553.52	30628	21841.7167	ENOG41129MV;ENOG4111PC4	S:Function unknown;	------	------
BAB_RS17170	-	hypothetical protein	0.833	-0.264048865	0.218094331	0.365157417	no	down	mRNA	5.95	6	5.16	4.89	5.13	3.28	5.70333	4.43333333	ENOG4110S5I	S:Function unknown	------	------
BAB_RS17455	-	esterase	0.917	-0.125587499	0.219586671	0.367273282	no	down	mRNA	20.82	20.94	25.87	18.64	21.23	18.64	22.5433	19.5033333	COG4757	S:Function unknown	------	------
BAB_RS18695	-	type II toxin-antitoxin system death-on-curing family toxin	0.915	-0.127876071	0.220110917	0.367958573	no	down	mRNA	30.29	25.89	32.98	27.29	25.12	24.67	29.72	25.6933333	COG3654	S:Function unknown	GO:0016301	MF:kinase activity;
BAB_RS25805	-	protein-export protein SecB	0.957	-0.063835275	0.22185242	0.370099597	no	down	mRNA	818.73	796.07	801.07	743.7	741.57	718.71	805.29	734.66	COG1952	U:Intracellular trafficking, secretion, and vesicular	GO:0015031;GO:0006457;GO:0051262;GO:0051262;GO:0015031;GO:00	BP:protein transport;BP:protein folding;BP:protein tetramerization;BP:protein tetramerization;BP:protein transport;CC:cytoplasm;CC:cytoplasm;MF:unfolded protein binding;MF:unfolded protein binding;
BAB_RS29655	-	cellulose synthase	0.884	-0.177595958	0.222916108	0.371488307	no	down	mRNA	3.88	3.54	4.03	2.87	3.39	3.25	3.81667	3.17	COG1215	M:Cell wall/membrane/envelope biogenesis	GO:0016021;GO:0016757	CC:integral component of membrane;MF:transferase activity, transferring glycosyl groups;
BAB_RS19090	-	porin	0.791	-0.338378414	0.223345616	0.372011127	no	down	mRNA	2.11	1.99	2.06	1.45	1.35	1.82	2.05333	1.54	ENOG4111F41	M:Cell wall/membrane/envelope biogenesis	GO:0006811;GO:0006811;GO:0009279;GO:0046930;GO:0009279;GO:00	BP:ion transport;BP:ion transport;CC:cell outer membrane;CC:pore complex;CC:cell outer membrane;CC:pore complex;MF:porin activity;MF:porin activity;
BAB_RS10435	-	DNA replication and repair protein RecF	0.9	-0.152692063	0.223493833	0.372065122	no	down	mRNA	20.36	15.82	21.57	15.07	18.43	15.16	19.25	16.22	COG1195	L:Replication, recombination and repair	GO:0006281;GO:0006260;GO:0009432;GO:0006281;GO:0006260;GO:00	BP:DNA repair;BP:DNA replication;BP:SOS response;BP:DNA repair;BP:DNA replication;BP:SOS response;CC:cytoplasm;CC:cytoplasm;MF:single-stranded DNA binding;MF:ATP binding;MF:ATP binding;MF:single-stranded DNA binding;
BAB_RS21360	-	hypothetical protein	0.938	-0.09253971	0.224316442	0.373047997	no	down	mRNA	143.93	134.82	147.37	122.49	139.02	115.95	142.04	125.82	ENOG4111XIA;ENOG411266S	S:Function unknown;	------	------
BAB_RS22985	-	PhnA protein	0.929	-0.106590923	0.224277083	0.373175695	no	down	mRNA	86.64	73.78	79.47	77.31	67.4	65.24	79.9633	69.9833333	COG2824	P:Inorganic ion transport and metabolism	------	------
BAB_RS25715	-	ABC transporter permease	0.911	-0.134235482	0.225637645	0.374857163	no	down	mRNA	85.6	67.04	68.97	70.37	62.39	56.9	73.87	63.22	COG4120	S:Function unknown	GO:0016021;GO:0005886;GO:0022857	CC:integral component of membrane;CC:plasma membrane;MF:transmembrane transporter activity;
BAB_RS30220	-	gfo/Idh/MocA family oxidoreductase	0.882	-0.181335	0.22588176	0.375068783	no	down	mRNA	10	11.58	10.82	7.84	9.32	9.97	10.8	9.04333333	COG0673	K:Transcription	GO:0016491	MF:oxidoreductase activity;
BAB_RS24385	-	hypothetical protein	0.914	-0.130200151	0.226409767	0.375557348	no	down	mRNA	32.41	27.32	39.9	29.55	30.1	25.69	33.21	28.4466667	------	------	------	------
BAB_RS28635	-	exodeoxyribonuclease VII large subunit	0.92	-0.121044387	0.226320359	0.37560295	no	down	mRNA	25.72	30.2	33.18	23.53	27.51	26.37	29.7	25.8033333	COG1570	L:Replication, recombination and repair	GO:0006308;GO:0006308;GO:0005737;GO:0009318;GO:0005737;GO:00	BP:DNA catabolic process;BP:DNA catabolic process;CC:cytoplasm;CC:exodeoxyribonuclease VII complex;CC:cytoplasm;CC:exodeoxyribonuclease VII complex;MF:exodeoxyribonuclease VII activity;MF:nucleic acid binding;MF:exodeoxyribonuclease VII activity;MF:nucleic acid binding;
BAB_RS30085	-	acetyl-CoA synthetase	0.745	-0.425173941	0.227456634	0.376904675	no	down	mRNA	2.02	1.66	2.21	0.85	1.83	1.4	1.96333	1.36	COG0365	K:Transcription	GO:0008152;GO:0008152;GO:0016874;GO:0003824	BP:metabolic process;BP:metabolic process;MF:ligase activity;MF:catalytic activity;
BAB_RS24420	-	50S ribosomal protein L32	0.895	-0.159831552	0.228050865	0.377694551	no	down	mRNA	331.01	301.97	410.4	355.79	264.93	262.33	347.793	294.35	COG0333	J:Translation, ribosomal structure and biogenesis	GO:0006412;GO:0006412;GO:0015934;GO:0015934;GO:0003735;GO:00	BP:translation;BP:translation;CC:large ribosomal subunit;CC:large ribosomal subunit;MF:structural constituent of ribosome;MF:structural constituent of ribosome;
BAB_RS23635	-	glycosyl transferase	0.896	-0.158786448	0.229079855	0.379203284	no	down	mRNA	33.91	26.71	24.76	24.82	22.51	24.39	28.46	23.9066667	COG3306	S:Function unknown	GO:0016740	MF:transferase activity;
BAB_RS29420	-	membrane protein	0.893	-0.163802142	0.229724095	0.380073901	no	down	mRNA	6.9	7.48	9.11	7.03	6.51	6.36	7.83	6.63333333	ENOG411108W;COG0697	S:Function unknown;	GO:0016021	CC:integral component of membrane;
BAB_RS33400	-	uracil permease	0.658	-0.604319157	0.231108919	0.381971686	no	down	mRNA	0.89	1.11	1.98	1.21	0.68	0.71	1.32667	0.86666667	COG2233	F:Nucleotide transport and metabolism	GO:0016021;GO:0022857	CC:integral component of membrane;MF:transmembrane transporter activity;
BAB_RS17385	-	sensor histidine kinase	0.939	-0.090804779	0.232334957	0.383800625	no	down	mRNA	24.71	24.58	26.47	20.88	23.06	23.13	25.2533	22.3566667	ENOG410XPBE	T:Signal transduction mechanisms	GO:0016021;GO:0005524;GO:0016301	CC:integral component of membrane;MF:ATP binding;MF:kinase activity;
BAB_RS24285	-	MFS transporter	0.929	-0.105570805	0.232653766	0.384129779	no	down	mRNA	39.54	33.01	42.01	31.48	36.18	32.39	38.1867	33.35	ENOG410Y0GE	G:Carbohydrate transport and metabolism	GO:0055085;GO:0016021	BP:transmembrane transport;CC:integral component of membrane;
BAB_RS29965	-	two-component sensor histidine kinase	0.936	-0.09509519	0.234015465	0.385783319	no	down	mRNA	51.12	50.11	48.3	41.09	47.34	43.47	49.8433	43.9666667	ENOG410XTWD	S:Function unknown	GO:0016021;GO:0005622;GO:0016021;GO:0005622;GO:0005524;GO:00	CC:integral component of membrane;CC:intracellular;CC:integral component of membrane;CC:intracellular;MF:ATP binding;MF:phosphorelay sensor kinase activity;MF:phosphorelay sensor kinase activity;MF:ATP binding;
BAB_RS23300	-	hypothetical protein	0.739	-0.435936575	0.234586957	0.386131092	no	down	mRNA	20.56	12.2	8.5	10.12	10.2	7.11	13.7533	9.14333333	------	------	------	------
BAB_RS32295	-	IS5/IS1182 family transposase	0.895	-0.160829668	0.23497908	0.386578487	no	down	mRNA	16.55	14.09	16.65	11.98	14.62	13.23	15.7633	13.2766667	ENOG4111SY5	L:Replication, recombination and repair	------	------
BAB_RS17265	-	septum formation inhibitor Maf	0.939	-0.090043697	0.235838811	0.387199847	no	down	mRNA	105.49	102.92	109.43	100.7	93.87	88.93	105.947	94.5	COG0424	D:Cell cycle control, cell division, chromosome par	GO:0005737;GO:0047429	CC:cytoplasm;MF:nucleoside-triphosphate diphosphatase activity;
BAB_RS19350	-	molybdopterin synthase catalytic subunit	0.936	-0.09514201	0.236008004	0.387279732	no	down	mRNA	225.45	202.88	197.36	199.12	175.11	180.08	208.563	184.77	COG0314	H:Coenzyme transport and metabolism	GO:0006777;GO:0006777;GO:0030366	BP:Mo-molybdopterin cofactor biosynthetic process;BP:Mo-molybdopterin cofactor biosynthetic process;MF:molybdopterin synthase activity;
BAB_RS32835	-	hypothetical protein	0.465	-1.104971389	0.23653053	0.387741119	no	down	mRNA	0	0	0	0	0	0	0	0	------	------	------	------
BAB_RS26395	-	ABC transporter permease	0.905	-0.144568578	0.23698758	0.38809434	no	down	mRNA	18.91	15.39	19.88	15.68	14.51	16.13	18.06	15.44	COG1079	S:Function unknown	GO:0008643;GO:0016021;GO:0005886;GO:0016021;GO:0005886;GO:00	BP:carbohydrate transport;CC:integral component of membrane;CC:plasma membrane;CC:integral component of membrane;CC:plasma membrane;MF:transmembrane transporter activity;MF:transmembrane transporter activity;
BAB_RS17010	-	FAD-dependent oxidoreductase	0.741	-0.432034915	0.237429448	0.388619876	no	down	mRNA	0.8	2.2	2.09	1.25	1.23	1.25	1.69667	1.24333333	COG0446	S:Function unknown	GO:0046653;GO:0008115	BP:tetrahydrofolate metabolic process;MF:sarcosine oxidase activity;
BAB_RS23035	-	integrase	0.649	-0.623195606	0.2383069	0.389659069	no	down	mRNA	4.07	1.73	3.5	0.73	2.67	2.08	3.1	1.82666667	COG0582;ENOG4111JCD	S:Function unknown;	GO:0006310;GO:0015074;GO:0015074;GO:0006310;GO:0003677;GO:00	BP:DNA recombination;BP:DNA integration;BP:DNA integration;BP:DNA recombination;MF:DNA binding;MF:DNA binding;
BAB_RS26175	-	tRNA (adenosine(37)-N6)-threonylcarbamoyltransferase complex dimerization subunit type 1 TsaB	0.907	-0.140135824	0.239900798	0.391866428	no	down	mRNA	37.2	36.88	35.41	33.54	27.24	33.68	36.4967	31.4866667	COG1214	O:Posttranslational modification, protein turnover,	GO:0002949;GO:0002949;GO:0002949;GO:0008233;GO:0016740;GO:00	BP:tRNA threonylcarbamoyladenosine modification;BP:tRNA threonylcarbamoyladenosine modification;BP:tRNA threonylcarbamoyladenosine modification;MF:peptidase activity;MF:transferase activity;MF:metallopeptidase activity;MF:peptidase activity;MF:hydrolase activity;
BAB_RS19505	-	AAA family ATPase	0.873	-0.195490667	0.240252509	0.392042312	no	down	mRNA	20.05	14.59	14.03	11.28	13.72	14.61	16.2233	13.2033333	ENOG4111ZXV	S:Function unknown	------	------
BAB_RS25775	-	uroporphyrinogen decarboxylase	0.95	-0.074571726	0.240508078	0.392260129	no	down	mRNA	285.24	255.84	262.58	226.25	250.55	243.46	267.887	240.086667	COG0407	H:Coenzyme transport and metabolism	GO:0006782;GO:0005737;GO:0004853;GO:0016787	BP:protoporphyrinogen IX biosynthetic process;CC:cytoplasm;MF:uroporphyrinogen decarboxylase activity;MF:hydrolase activity;
BAB_RS24195	-	DUF1284 domain-containing protein	0.94	-0.088507565	0.242066147	0.394001282	no	down	mRNA	84.07	82.05	84.57	79.92	69.32	73.71	83.5633	74.3166667	COG3543	S:Function unknown	------	------
BAB_RS29595	-	IS5/IS1182 family transposase	0.88	-0.183859512	0.2420069	0.394104496	no	down	mRNA	107.39	111.74	143.67	97.62	117.24	99.1	120.933	104.653333	ENOG4111SY5;COG3293	L:Replication, recombination and repair;	------	------
BAB_RS24315	-	tetratricopeptide repeat protein	0.94	-0.089770914	0.243149644	0.394764935	no	down	mRNA	192.48	177.62	191.73	147.5	175.04	177.31	187.277	166.616667	ENOG4111WRX	S:Function unknown	GO:0016787	MF:hydrolase activity;
BAB_RS28505	-	hypothetical protein	0.949	-0.07533799	0.242925513	0.394800036	no	down	mRNA	64.43	65.29	68.35	58.41	62.16	56.58	66.0233	59.05	ENOG4111KDB	S:Function unknown	------	------
BAB_RS28210	-	hypothetical protein	0.939	-0.091205002	0.243105109	0.394892171	no	down	mRNA	77.16	75.5	74.81	65.81	70.78	65.93	75.8233	67.5066667	ENOG410YGH2	S:Function unknown	------	------
BAB_RS32595	-	hypothetical protein	0.869	-0.202211155	0.244504693	0.396364067	no	down	mRNA	12.31	13.38	14.74	12.57	10.02	11.21	13.4767	11.2666667	------	------	------	------
BAB_RS17560	-	MATE family efflux transporter	0.941	-0.087516743	0.245471703	0.397731005	no	down	mRNA	35.93	34.41	37.07	33.73	32.41	28.97	35.8033	31.7033333	COG0534	V:Defense mechanisms	GO:0016021;GO:0016021;GO:0015297;GO:0015238;GO:0015297;GO:00	CC:integral component of membrane;CC:integral component of membrane;MF:antiporter activity;MF:drug transmembrane transporter activity;MF:antiporter activity;MF:drug transmembrane transporter activity;
BAB_RS25155	-	intracellular septation protein A	0.927	-0.108932546	0.248287746	0.401888428	no	down	mRNA	78.2	63.02	67.68	58.51	58.31	66.14	69.6333	60.9866667	COG2917	D:Cell cycle control, cell division, chromosome par	GO:0090529;GO:0051301;GO:0007049;GO:0090529;GO:0016021;GO:00	BP:cell septum assembly;BP:cell division;BP:cell cycle;BP:cell septum assembly;CC:integral component of membrane;CC:integral component of plasma membrane;CC:plasma membrane;CC:integral component of plasma membrane;
BAB_RS20200	-	deoxyguanosinetriphosphate triphosphohydrolase	0.943	-0.084350299	0.248707158	0.402364601	no	down	mRNA	40.81	33.8	39.9	33.8	32.94	34.76	38.17	33.8333333	COG0232	F:Nucleotide transport and metabolism	GO:0004180;GO:0016793;GO:0016793	MF:carboxypeptidase activity;MF:triphosphoric monoester hydrolase activity;MF:triphosphoric monoester hydrolase activity;
BAB_RS18825	-	DUF1214 domain-containing protein	0.925	-0.112415955	0.249038759	0.40249574	no	down	mRNA	122.04	124.13	135.64	123.24	115.01	97.38	127.27	111.876667	COG5402	S:Function unknown	------	------
BAB_RS32440	-	hypothetical protein	0.906	-0.142655215	0.249821771	0.403558246	no	down	mRNA	50.17	50.43	51.39	41.62	47.35	43.62	50.6633	44.1966667	------	------	------	------
BAB_RS33355	-	hypothetical protein	0.748	-0.419589129	0.250305436	0.403933384	no	down	mRNA	16.57	15.21	15.6	8.26	15.23	11.32	15.7933	11.6033333	COG3346;COG3346	E:Amino acid transport and metabolism;Q:Second	------	------
BAB_RS31815	-	ABC transporter permease	0.894	-0.161736487	0.250810904	0.404140137	no	down	mRNA	14.9	14.75	14.71	11.2	12.41	14	14.7867	12.5366667	COG0600	P:Inorganic ion transport and metabolism	GO:0055085;GO:0055085;GO:0016021;GO:0005886;GO:0016021;GO:00	BP:transmembrane transport;BP:transmembrane transport;CC:integral component of membrane;CC:plasma membrane;CC:integral component of membrane;CC:plasma membrane;MF:ATP binding;MF:ATPase activity;
BAB_RS32480	-	hypothetical protein	0.869	-0.202708184	0.250754972	0.404252748	no	down	mRNA	16.03	18.17	17.02	10.99	14.45	17	17.0733	14.1466667	------	------	------	------
BAB_RS33305	-	hypothetical protein	0.782	-0.355485222	0.251065613	0.404347776	no	down	mRNA	17.06	15.84	11.71	10.49	8.99	14.26	14.87	11.2466667	------	------	GO:0006351;GO:0003700;GO:0043565	BP:transcription, DNA-templated;MF:transcription factor activity, sequence-specific DNA binding;MF:sequence-specific DNA binding;
BAB_RS33470	-	phenylacetic acid degradation protein	0.706	-0.502500467	0.251647459	0.404878962	no	down	mRNA	67.73	89.51	70.87	73.18	50.58	45.05	76.0367	56.27	------	------	------	------
BAB_RS28680	-	ABC transporter ATP-binding protein	0.946	-0.080703247	0.251841466	0.404988304	no	down	mRNA	61.39	61.1	66.58	56.27	58.16	54.76	63.0233	56.3966667	COG1120	S:Function unknown	GO:0016887;GO:0005524;GO:0005524;GO:0016887	MF:ATPase activity;MF:ATP binding;MF:ATP binding;MF:ATPase activity;
BAB_RS25910	-	ATP-binding protein	0.908	-0.13861202	0.25231677	0.405549665	no	down	mRNA	68.72	76.74	88.87	71.74	63.58	70.22	78.11	68.5133333	COG1493	T:Signal transduction mechanisms	GO:0006109;GO:0006109;GO:0005622;GO:0005622;GO:0005524;GO:00	BP:regulation of carbohydrate metabolic process;BP:regulation of carbohydrate metabolic process;CC:intracellular;CC:intracellular;MF:ATP binding;MF:phosphorelay sensor kinase activity;MF:phosphorelay sensor kinase activity;MF:ATP binding;
BAB_RS24125	-	crossover junction endodeoxyribonuclease RuvC	0.951	-0.071838651	0.253277943	0.406484531	no	down	mRNA	161.75	160.28	173.2	150.59	146.88	150.41	165.077	149.293333	COG0817	L:Replication, recombination and repair	GO:0006310;GO:0006281;GO:0006310;GO:0006281;GO:0000287;GO:00	BP:DNA recombination;BP:DNA repair;BP:DNA recombination;BP:DNA repair;MF:magnesium ion binding;MF:nucleic acid binding;MF:crossover junction endodeoxyribonuclease activity;MF:metal ion binding;MF:ligase activity;MF:crossover junction endodeoxyribonuclease activity;MF:metal ion binding;MF:nucleic acid binding;
BAB_RS30545	-	hypothetical protein	0.891	-0.166884052	0.254664497	0.40830191	no	down	mRNA	74.07	70.65	76.31	65.5	63.1	65.86	73.6767	64.82	------	------	------	------
BAB_RS27205	-	aldo/keto reductase	0.952	-0.070486984	0.255124942	0.408836129	no	down	mRNA	474.16	391.92	435.94	380.97	398.18	390.13	434.007	389.76	COG0656	S:Function unknown	GO:0016491	MF:oxidoreductase activity;
BAB_RS24675	-	DUF1275 domain-containing protein	0.93	-0.104652602	0.257226673	0.411383425	no	down	mRNA	17.71	18.09	18.37	14.94	17.42	15.06	18.0567	15.8066667	COG3619	S:Function unknown	GO:0016021	CC:integral component of membrane;
BAB_RS20230	tatA	twin-arginine translocase TatA/TatE family subunit	0.944	-0.082452828	0.257183734	0.41151959	no	down	mRNA	1697.24	1481.24	1478.71	1467.29	1417.43	1454.06	1552.4	1446.26	ENOG410XUF0	U:Intracellular trafficking, secretion, and vesicular	GO:0009306;GO:0043953;GO:0009306;GO:0016021;GO:0005887;GO:00	BP:protein secretion;BP:protein transport by the Tat complex;BP:protein secretion;CC:integral component of membrane;CC:integral component of plasma membrane;CC:plasma membrane;CC:TAT protein transport complex;CC:integral component of membrane;CC:plasma membrane;MF:protein transporter activity;MF:protein transmembrane transporter activity;MF:protein transporter activity;
BAB_RS28395	-	ABC transporter substrate-binding protein	0.922	-0.117829469	0.258750537	0.412383668	no	down	mRNA	35.83	30.89	34.62	32.39	30.27	25.06	33.78	29.24	COG4134	S:Function unknown	------	------
BAB_RS23470	-	cation transporter	0.935	-0.096946864	0.25850069	0.412394596	no	down	mRNA	65.71	57.39	66.01	61.89	53.49	51.11	63.0367	55.4966667	COG0053	P:Inorganic ion transport and metabolism	GO:0016021;GO:0005886;GO:0016021;GO:0005886;GO:0008324;GO:00	CC:integral component of membrane;CC:plasma membrane;CC:integral component of membrane;CC:plasma membrane;MF:cation transmembrane transporter activity;MF:cation transmembrane transporter activity;
BAB_RS30155	-	LPS biosynthesis protein	0.88	-0.184991658	0.258278084	0.412449048	no	down	mRNA	3.05	2.84	3.72	2.36	3.07	2.5	3.20333	2.64333333	COG3206	S:Function unknown	GO:0016021	CC:integral component of membrane;
BAB_RS31715	-	hypothetical protein	0.812	-0.300362713	0.258410631	0.412455717	no	down	mRNA	1.52	1.37	1.79	1.05	1.3	1.24	1.56	1.19666667	COG5441	S:Function unknown	GO:0008168	MF:methyltransferase activity;
BAB_RS21880	-	50S ribosomal protein L29	0.881	-0.182966293	0.259663193	0.413428066	no	down	mRNA	2369.46	2173.39	2206.56	2070.22	2178.62	1890.42	2249.8	2046.42	COG0255	J:Translation, ribosomal structure and biogenesis	GO:0006412;GO:0005840;GO:0003735	BP:translation;CC:ribosome;MF:structural constituent of ribosome;
BAB_RS28490	-	helix-turn-helix transcriptional regulator	0.919	-0.12257405	0.261590089	0.416083642	no	down	mRNA	32.06	28.43	37.31	29.68	27.19	28.34	32.6	28.4033333	COG2932	K:Transcription	GO:0003677	MF:DNA binding;
BAB_RS19890	-	NADH-quinone oxidoreductase subunit H	0.949	-0.076165813	0.263298858	0.418387354	no	down	mRNA	410.77	345.11	357.59	329.68	339.92	326.63	371.157	332.076667	COG1005	C:Energy production and conversion	GO:0016021;GO:0005886;GO:0016021;GO:0005886;GO:0016655;GO:00	CC:integral component of membrane;CC:plasma membrane;CC:integral component of membrane;CC:plasma membrane;MF:oxidoreductase activity, acting on NA;MF:quinone binding;MF:oxidoreductase activity;MF:oxidoreductase activity, acting on NA;MF:quinone binding;
BAB_RS18290	-	cytochrome c oxidase subunit 3	0.952	-0.070526128	0.263691768	0.418597653	no	down	mRNA	112.37	101.75	106.59	92.47	98.14	97.66	106.903	96.09	COG1845	O:Posttranslational modification, protein turnover,	GO:0019646;GO:0019646;GO:0016021;GO:0016021;GO:0005886;GO:00	BP:aerobic electron transport chain;BP:aerobic electron transport chain;CC:integral component of membrane;CC:integral component of membrane;CC:plasma membrane;MF:cytochrome-c oxidase activity;MF:cytochrome-c oxidase activity;
BAB_RS29710	-	IS5/IS1182 family transposase	0.836	-0.258527529	0.263571711	0.418613893	no	down	mRNA	19.55	18.57	18.81	11.82	16.2	18.18	18.9767	15.4	COG3293	L:Replication, recombination and repair	------	------
BAB_RS17635	-	LrgB family protein	0.935	-0.097250801	0.264140627	0.419103128	no	down	mRNA	491.89	466.09	502.91	494.86	402.79	395.69	486.963	431.113333	COG1346	M:Cell wall/membrane/envelope biogenesis	GO:0016021;GO:0016021;GO:0016787	CC:integral component of membrane;CC:integral component of membrane;MF:hydrolase activity;
BAB_RS31020	-	tRNA pseudouridine(38-40) synthase TruA	0.898	-0.155002568	0.264879607	0.419860966	no	down	mRNA	17.2	13.82	18.46	15.87	13.04	13.02	16.4933	13.9766667	COG0101	J:Translation, ribosomal structure and biogenesis	GO:0031119;GO:0008033;GO:0031119;GO:0001522;GO:0009982;GO:00	BP:tRNA pseudouridine synthesis;BP:tRNA processing;BP:tRNA pseudouridine synthesis;BP:pseudouridine synthesis;MF:pseudouridine synthase activity;MF:RNA binding;MF:hydrolase activity;MF:pseudouridine synthase activity;MF:RNA binding;
BAB_RS22235	-	cobalt-precorrin-5B (C(1))-methyltransferase	0.949	-0.075538083	0.265199755	0.42016115	no	down	mRNA	68.98	66.69	68.6	63.33	58.28	61.46	68.09	61.0233333	COG1903	H:Coenzyme transport and metabolism	GO:0009236;GO:0046140;GO:0009236;GO:0046140;GO:0008168;GO:00	BP:cobalamin biosynthetic process;BP:corrin biosynthetic process;BP:cobalamin biosynthetic process;BP:corrin biosynthetic process;MF:methyltransferase activity;MF:methyltransferase activity;
BAB_RS23160	-	Lrp/AsnC family transcriptional regulator	0.947	-0.077878578	0.266942193	0.422505058	no	down	mRNA	144.67	137.68	139.4	116.87	131.42	130.56	140.583	126.283333	COG1522	P:Inorganic ion transport and metabolism	GO:0006351;GO:0005622;GO:0003700;GO:0043565	BP:transcription, DNA-templated;CC:intracellular;MF:transcription factor activity, sequence-specific DNA binding;MF:sequence-specific DNA binding;
BAB_RS25595	-	hypothetical protein	0.67	-0.577769452	0.267085205	0.422523271	no	down	mRNA	188.38	123.58	111.21	58.82	73.32	151.7	141.057	94.6133333	ENOG410Y60Y	S:Function unknown	------	------
BAB_RS17805	-	cytochrome-c oxidase%2C cbb3-type subunit I	0.951	-0.073116923	0.267241951	0.422563183	no	down	mRNA	1253.6	1084.57	1145.78	1042.52	1016.4	1060.99	1161.32	1039.97	COG3278	C:Energy production and conversion	GO:0009060;GO:0009060;GO:0009060;GO:0016021;GO:0045278;GO:00	BP:aerobic respiration;BP:aerobic respiration;BP:aerobic respiration;CC:integral component of membrane;CC:plasma membrane respiratory chain complex IV;CC:integral component of membrane;CC:plasma membrane respiratory chain complex IV;CC:integral component of membrane;CC:plasma membrane respiratory chain complex IV;MF:cytochrome-c oxidase activity;MF:heme binding;MF:metal ion binding;MF:cytochrome-c oxidase activity;MF:hydrolase activity;MF:heme binding;MF:metal ion binding;MF:cytochrome-c oxidase activity;MF:heme binding;MF:metal ion binding;
BAB_RS17730	-	hypothetical protein	0.932	-0.101511735	0.2686282	0.423920632	no	down	mRNA	125.55	112.57	129.06	115.51	107.2	106.07	122.393	109.593333	COG2351	S:Function unknown	------	------
BAB_RS28730	-	alanine racemase	0.909	-0.137743496	0.269536797	0.424520455	no	down	mRNA	18.06	18.84	17.52	17.26	14.54	15.05	18.14	15.6166667	COG3616	E:Amino acid transport and metabolism	------	------
BAB_RS17095	-	hypothetical protein	0.625	-0.677568076	0.27047413	0.42557952	no	down	mRNA	42.76	34.11	33.47	43.21	16.01	9.68	36.78	22.9666667	------	------	------	------
BAB_RS22685	-	dihydrofolate reductase	0.921	-0.11879503	0.270676449	0.425689393	no	down	mRNA	82.87	68.43	83.87	58.53	75.34	71.14	78.39	68.3366667	COG0262	H:Coenzyme transport and metabolism	GO:0006545;GO:0006730;GO:0046654;GO:0006545;GO:0006730;GO:00	BP:glycine biosynthetic process;BP:one-carbon metabolic process;BP:tetrahydrofolate biosynthetic process;BP:glycine biosynthetic process;BP:one-carbon metabolic process;BP:tetrahydrofolate biosynthetic process;BP:glycine biosynthetic process;BP:one-carbon metabolic process;BP:tetrahydrofolate biosynthetic process;MF:dihydrofolate reductase activity;MF:NADP binding;MF:dihydrofolate reductase activity;MF:NADP binding;MF:dihydrofolate reductase activity;MF:kinase activity;MF:NADP binding;
BAB_RS27725	-	Lrp/AsnC family transcriptional regulator	0.918	-0.123337748	0.270473736	0.425787415	no	down	mRNA	30.4	22.75	26.41	24.84	22.9	20.6	26.52	22.78	COG1522	E:Amino acid transport and metabolism	GO:0006351;GO:0005622;GO:0003700;GO:0043565	BP:transcription, DNA-templated;CC:intracellular;MF:transcription factor activity, sequence-specific DNA binding;MF:sequence-specific DNA binding;
BAB_RS26700	-	hypothetical protein	0.93	-0.105400865	0.273215931	0.42905317	no	down	mRNA	32.59	30.47	33.03	28.34	24.47	31.49	32.03	28.1	COG5338	S:Function unknown	------	------
BAB_RS31415	-	lactate dehydrogenase	0.83	-0.267967445	0.273953692	0.430001569	no	down	mRNA	5.57	6.4	6.44	3.88	4.37	6.48	6.13667	4.91	COG0277	S:Function unknown	GO:0006089;GO:0055085;GO:0055114;GO:0006089;GO:0055085;GO:00	BP:lactate metabolic process;BP:transmembrane transport;BP:oxidation-reduction process;BP:lactate metabolic process;BP:transmembrane transport;MF:flavin adenine dinucleotide binding;MF:D-lactate dehydrogenase activity;MF:catalytic activity;MF:catalytic activity;MF:D-lactate dehydrogenase activity;MF:flavin adenine dinucleotide binding;
BAB_RS21745	-	protease	0.95	-0.074575453	0.274307338	0.430346425	no	down	mRNA	813.23	777.97	809.88	647.48	739.38	775.37	800.36	720.743333	COG0693	S:Function unknown	GO:0008233	MF:peptidase activity;
BAB_RS20910	-	MCE family protein	0.954	-0.068404173	0.275204483	0.430912283	no	down	mRNA	309.06	269.48	282.7	250.78	271.77	252.37	287.08	258.306667	COG1463	Q:Secondary metabolites biosynthesis, transport a	GO:0016021	CC:integral component of membrane;
BAB_RS25355	-	short-chain dehydrogenase	0.945	-0.081504553	0.276049839	0.431815059	no	down	mRNA	51.9	47.16	54.24	47.27	46.35	43.09	51.1	45.57	COG1028	S:Function unknown	GO:0016491	MF:oxidoreductase activity;
BAB_RS33475	-	amidase	0.945	-0.08201798	0.277155321	0.432701675	no	down	mRNA	40.61	39.17	48.87	37.68	40.39	36.19	42.8833	38.0866667	COG0154	C:Energy production and conversion	------	------
BAB_RS32740	-	hypothetical protein	0.894	-0.16145118	0.277770346	0.433240835	no	down	mRNA	11.58	9.86	12.31	8.61	9.77	10.14	11.25	9.50666667	------	------	------	------
BAB_RS27600	-	6-aminohexanoate-dimer hydrolase	0.93	-0.104460208	0.278958126	0.434882319	no	down	mRNA	52.12	43.97	43.7	37.44	44.42	40.26	46.5967	40.7066667	COG1680	S:Function unknown	GO:0016021;GO:0016021;GO:0016021;GO:0016021;GO:0019875;GO:00	CC:integral component of membrane;CC:integral component of membrane;CC:integral component of membrane;CC:integral component of membrane;MF:6-aminohexanoate-dimer hydrolase activity;MF:hydrolase activity;MF:6-aminohexanoate-dimer hydrolase activity;
BAB_RS25555	-	hypothetical protein	0.958	-0.061522651	0.279804112	0.435778289	no	down	mRNA	133.84	126.15	131.32	120.24	119.43	113.64	130.437	117.77	------	------	------	------
BAB_RS22515	-	Urease accessory protein UreF 2	0.958	-0.062375476	0.279671078	0.435782334	no	down	mRNA	231.84	224.55	241	203.16	220.04	209.27	232.463	210.823333	COG0830	S:Function unknown	GO:0006807;GO:0006807;GO:0005737;GO:0005737;GO:0016151;GO:00	BP:nitrogen compound metabolic process;BP:nitrogen compound metabolic process;CC:cytoplasm;CC:cytoplasm;MF:nickel cation binding;MF:nickel cation binding;
BAB_RS16930	-	thiazole synthase	0.82	-0.285442213	0.281748558	0.437745704	no	down	mRNA	5.24	8.15	6.48	5.75	6.13	3.62	6.62333	5.16666667	COG2022	H:Coenzyme transport and metabolism	GO:0009229;GO:0009228;GO:0005737;GO:0016740;GO:0016783	BP:thiamine diphosphate biosynthetic process;BP:thiamine biosynthetic process;CC:cytoplasm;MF:transferase activity;MF:sulfurtransferase activity;
BAB_RS28780	-	MurR/RpiR family transcriptional regulator	0.948	-0.077741831	0.282260949	0.437906532	no	down	mRNA	91.47	87.5	98.56	75.85	85.84	86.98	92.51	82.89	COG1737	C:Energy production and conversion	GO:1901135;GO:0006351;GO:1901135;GO:0006351;GO:0097367;GO:00	BP:carbohydrate derivative metabolic process;BP:transcription, DNA-templated;BP:carbohydrate derivative metabolic process;BP:transcription, DNA-templated;MF:carbohydrate derivative binding;MF:transcription factor activity, sequence-specific DNA binding;MF:DNA binding;MF:carbohydrate derivative binding;MF:DNA binding;MF:transcription factor activity, sequence-specific DNA binding;
BAB_RS30210	dppD	ABC transporter ATP-binding protein	0.879	-0.186083756	0.287972126	0.446120753	no	down	mRNA	7.08	8.4	11.08	6.76	7.56	8.03	8.85333	7.45	COG0444	K:Transcription	GO:0015031;GO:0015833;GO:0015833;GO:0005886;GO:0005524;GO:00	BP:protein transport;BP:peptide transport;BP:peptide transport;CC:plasma membrane;MF:ATP binding;MF:ATPase activity;MF:ATP binding;MF:ATPase activity;
BAB_RS29605	-	IS66 family insertion sequence hypothetical protein	0.727	-0.459276378	0.28831964	0.446443858	no	down	mRNA	13.41	8.54	12.02	10.56	9.93	2.44	11.3233	7.64333333	ENOG4111NDY;COG3436	L:Replication, recombination and repair;	------	------
BAB_RS29625	-	AraC family transcriptional regulator	0.961	-0.05812687	0.288673925	0.44677713	no	down	mRNA	178.11	174.62	199.71	160.52	166.29	173	184.147	166.603333	ENOG4111NRR	L:Replication, recombination and repair	GO:0006351;GO:0003700;GO:0043565	BP:transcription, DNA-templated;MF:transcription factor activity, sequence-specific DNA binding;MF:sequence-specific DNA binding;
BAB_RS25795	-	dephospho-CoA kinase	0.937	-0.093292205	0.290473626	0.449129817	no	down	mRNA	111.72	123.79	123.71	97.93	114.97	107.98	119.74	106.96	COG0237	H:Coenzyme transport and metabolism	GO:0015937;GO:0005737;GO:0016787;GO:0005524;GO:0004140	BP:coenzyme A biosynthetic process;CC:cytoplasm;MF:hydrolase activity;MF:ATP binding;MF:dephospho-CoA kinase activity;
BAB_RS33245	-	hypothetical protein	0.817	-0.292329534	0.29132418	0.450011822	no	down	mRNA	4.75	4.87	9.4	5.29	4.36	5.51	6.34	5.05333333	------	------	------	------
BAB_RS23670	-	putrescine/spermidine ABC transporter permease	0.929	-0.106662184	0.292085119	0.450321251	no	down	mRNA	44.84	41.1	40.64	41.22	36.05	33.55	42.1933	36.94	COG1176	P:Inorganic ion transport and metabolism	GO:0055085;GO:0055085;GO:0016021;GO:0005886;GO:0016021	BP:transmembrane transport;BP:transmembrane transport;CC:integral component of membrane;CC:plasma membrane;CC:integral component of membrane;
BAB_RS17720	-	(S)-ureidoglycine aminohydrolase	0.95	-0.073286977	0.292039658	0.450467317	no	down	mRNA	70.47	69.26	73.23	59.35	63.1	68.56	70.9867	63.67	COG3257	S:Function unknown	GO:0016787	MF:hydrolase activity;
BAB_RS23945	-	DUF883 domain-containing protein	0.963	-0.054995821	0.29202649	0.450663359	no	down	mRNA	3089.14	2920.92	2973.23	2721.79	2761.35	2880.79	2994.43	2787.97667	ENOG410Y07Z	S:Function unknown	GO:0033554;GO:0033554;GO:0016021;GO:0043022;GO:0043022	BP:cellular response to stress;BP:cellular response to stress;CC:integral component of membrane;MF:ribosome binding;MF:ribosome binding;
BAB_RS26970	-	flagellar basal-body rod protein FlgF	0.749	-0.416794924	0.294062176	0.452717667	no	down	mRNA	2.31	1.64	2.01	1.52	0.86	1.89	1.98667	1.42333333	COG4786	N:Cell motility	GO:0071973;GO:0071973;GO:0030694;GO:0030694	BP:bacterial-type flagellum-dependent cell motility;BP:bacterial-type flagellum-dependent cell motility;CC:bacterial-type flagellum basal body, rod;CC:bacterial-type flagellum basal body, rod;
BAB_RS32815	-	hypothetical protein	0.919	-0.122433637	0.29431083	0.452883475	no	down	mRNA	11.46	9.63	12.07	9.98	9.28	9.41	11.0533	9.55666667	------	------	------	------
BAB_RS31720	-	IclR family transcriptional regulator	0.646	-0.631140626	0.295086793	0.453860156	no	down	mRNA	1.14	1.49	1.34	0.58	0.29	1.71	1.32333	0.86	COG1414	S:Function unknown	GO:0006355;GO:0006351;GO:0006355;GO:0006351;GO:0003677;GO:00	BP:regulation of transcription, DNA-templated;BP:transcription, DNA-templated;BP:regulation of transcription, DNA-templated;BP:transcription, DNA-templated;MF:DNA binding;MF:DNA binding;
BAB_RS33265	-	hypothetical protein	0.882	-0.181873438	0.295396751	0.454119503	no	down	mRNA	62.92	69.37	66.46	53.25	50.98	69.84	66.25	58.0233333	------	------	------	------
BAB_RS26855	-	O-antigen ligase family protein	0.939	-0.091518379	0.295568681	0.45416651	no	down	mRNA	45.46	41.14	42.15	34.39	37.61	41.84	42.9167	37.9466667	COG3307	M:Cell wall/membrane/envelope biogenesis	GO:0016021	CC:integral component of membrane;
BAB_RS28645	-	16S rRNA (uracil(1498)-N(3))-methyltransferase	0.953	-0.069066773	0.296391804	0.455213607	no	down	mRNA	155.24	145.74	149.4	137.29	127.98	141.35	150.127	135.54	COG1385	S:Function unknown	GO:0006364;GO:0005737;GO:0008168	BP:rRNA processing;CC:cytoplasm;MF:methyltransferase activity;
BAB_RS16725	-	ribonuclease PH	0.956	-0.064769046	0.296947	0.455630712	no	down	mRNA	213.61	200.89	213.18	199.93	188.07	178.81	209.227	188.936667	COG0689	J:Translation, ribosomal structure and biogenesis	GO:0008033;GO:0016075;GO:0006364;GO:0008033;GO:0016075;GO:00	BP:tRNA processing;BP:rRNA catabolic process;BP:rRNA processing;BP:tRNA processing;BP:rRNA catabolic process;BP:rRNA processing;BP:rRNA catabolic process;BP:rRNA processing;BP:tRNA processing;MF:tRNA binding;MF:tRNA nucleotidyltransferase activity;MF:3'-5'-exoribonuclease activity;MF:tRNA binding;MF:tRNA nucleotidyltransferase activity;MF:3'-5'-exoribonuclease activity;MF:isomerase activity;MF:3'-5'-exoribonuclease activity;MF:tRNA binding;MF:tRNA nucleotidyltransferase activity;
BAB_RS26900	-	MFS transporter	0.858	-0.221427119	0.297470472	0.455780937	no	down	mRNA	16.46	14.87	15.87	16.08	12.01	10.52	15.7333	12.87	ENOG410XNN3;ENOG410ZVD3	P:Inorganic ion transport and metabolism;	GO:0055085;GO:0016021	BP:transmembrane transport;CC:integral component of membrane;
BAB_RS32450	-	hypothetical protein	0.929	-0.106323627	0.297347243	0.45580949	no	down	mRNA	9.25	8.25	9.62	8.06	7.4	8.26	9.04	7.90666667	------	------	------	------
BAB_RS22265	-	EamA family transporter	0.942	-0.085819226	0.297675613	0.455877858	no	down	mRNA	61.01	56.52	55.17	49.44	53.16	51.15	57.5667	51.25	COG2510	S:Function unknown	GO:0016021	CC:integral component of membrane;
[illegible]	[illegible]	cobalt transporter	[illegible]	[illegible]	[illegible]	[illegible]	[illegible]	[illegible]	[illegible]	[illegible]	[illegible]	[illegible]	[illegible]	[illegible]	[illegible]	[illegible]	[illegible]	[illegible]	[illegible]	[illegible]	[illegible]

BAB_RS18035	-	pyridoxine/pyridoxamine 5'-phosphate oxidase	0.942	-0.086527102	0.300794783	0.459996972	no	down	mRNA	89.19	90.33	81.31	72.86	80.51	78.72	86.9433	77.3633333	COG0259	H:Coenzyme transport and metabolism	GO:0008615;GO:0008615;GO:0004733;GO:0010181;GO:0010181;GO:00	BP:pyridoxine biosynthetic process;BP:pyridoxine biosynthetic process;MF:pyridoxamine-phosphate oxidase activity;MF:FMN binding;MF:FMN binding;MF:pyridoxamine-phosphate oxidase activity;
BAB_RS31365	-	ABC transporter substrate-binding protein	0.931	-0.103550759	0.301724085	0.460979308	no	down	mRNA	13.69	12.56	14.22	12.05	11.27	12.21	13.49	11.8433333	COG0687	S:Function unknown	------	------
BAB_RS32820	-	hypothetical protein	0.808	-0.307223891	0.307712852	0.469459351	no	down	mRNA	8.23	6.21	6.28	5.22	5.61	4.86	6.90667	5.23	------	------	------	------
BAB_RS17795	-	CcoQ/FixQ family Cbb3-type cytochrome c oxidase assembly chaperone	0.818	-0.290302592	0.308492792	0.469979772	no	down	mRNA	1470.18	1476.14	1653.42	1161.58	1319.39	1572.45	1533.25	1351.14	COG4736	C:Energy production and conversion	GO:0016021;GO:0016021;GO:0004129	CC:integral component of membrane;CC:integral component of membrane;MF:cytochrome-c oxidase activity;
BAB_RS20940	-	NAD(P)/FAD-dependent oxidoreductase	0.848	-0.237263965	0.309757766	0.471683271	no	down	mRNA	13.05	9.89	15.1	11.95	11.29	7.23	12.68	10.1566667	COG0492	O:Posttranslational modification, protein turnover,	GO:0016021;GO:0016491	CC:integral component of membrane;MF:oxidoreductase activity;
BAB_RS25500	-	hypothetical protein	0.912	-0.132910919	0.309916983	0.471702164	no	down	mRNA	13.86	11.64	12.82	12.48	10.81	9.48	12.7733	10.9233333	------	------	------	------
BAB_RS29765	-	gfo/Idh/MocA family oxidoreductase	0.929	-0.106583684	0.313318468	0.47642794	no	down	mRNA	21.97	18.57	19.63	16.12	18.7	17.61	20.0567	17.4766667	ENOG410XRSB	S:Function unknown	GO:0016491	MF:oxidoreductase activity;
BAB_RS28830	-	Fe(3+) ions import ATP-binding protein FbpC	0.915	-0.128763764	0.314977492	0.477819963	no	down	mRNA	17.88	20.07	21.38	15.54	19.96	15.8	19.7767	17.1	COG3842	E:Amino acid transport and metabolism	GO:0055072;GO:0055072;GO:0043190;GO:0043190;GO:0015408;GO:00	BP:iron ion homeostasis;BP:iron ion homeostasis;CC:ATP-binding cassett;CC:ATP-binding cassett;MF:ferric-transporting ATPase activity;MF:ATP binding;MF:ferric-transporting ATPase activity;MF:kinase activity;MF:ATP binding;
BAB_RS19965	-	DUF3008 domain-containing protein	0.917	-0.12424523	0.314967031	0.478029793	no	down	mRNA	129.53	115.55	119.62	117.15	110.09	99.9	121.567	109.046667	ENOG410XVJV	S:Function unknown	------	------
BAB_RS19430	-	UDP-glucose 4-epimerase	0.944	-0.083765932	0.31601735	0.478945162	no	down	mRNA	20.63	20.41	22.77	18.08	20.28	18.37	21.27	18.91	COG0451	M:Cell wall/membrane/envelope biogenesis	GO:0003824;GO:0050662;GO:0003824;GO:0003978;GO:0050662;GO:00	MF:catalytic activity;MF:coenzyme binding;MF:catalytic activity;MF:UDP-glucose 4-epimerase activity;MF:coenzyme binding;MF:coenzyme binding;MF:UDP-glucose 4-epimerase activity;MF:catalytic activity;
BAB_RS25815	-	calcium-binding protein	0.955	-0.065672415	0.318125148	0.48168525	no	down	mRNA	301.59	290.44	311.97	292.13	270.92	255.02	301.333	272.69	COG4395	S:Function unknown	GO:0016021	CC:integral component of membrane;
BAB_RS22430	-	sugar ABC transporter ATP-binding protein	0.887	-0.172364623	0.319179766	0.482372807	no	down	mRNA	8.01	7.51	7.03	5.73	6.22	6.92	7.51667	6.29	COG1129	S:Function unknown	GO:0008643;GO:0008643;GO:0005524;GO:0016887;GO:0016887;GO:00	BP:carbohydrate transport;BP:carbohydrate transport;MF:ATP binding;MF:ATPase activity;MF:ATPase activity;MF:ATP binding;
BAB_RS23330	-	enoyl-CoA hydratase	0.88	-0.184286514	0.318840628	0.482541186	no	down	mRNA	34.29	24.29	24.97	26.46	18.72	24.16	27.85	23.1133333	COG2030	I:Lipid transport and metabolism	------	------
BAB_RS25000	-	succinate dehydrogenase iron-sulfur subunit	0.965	-0.050781536	0.319169021	0.48258356	no	down	mRNA	411.71	366.04	407.49	365.71	362.01	353.31	395.08	360.343333	COG0479	C:Energy production and conversion	GO:0006099;GO:0006099;GO:0051538;GO:0051539;GO:0051537;GO:00	BP:tricarboxylic acid cycle;BP:tricarboxylic acid cycle;MF:3 iron, 4 sulfur cluster binding;MF:4 iron, 4 sulfur cluster binding;MF:2 iron, 2 sulfur cluster binding;MF:succinate dehydrogenas;MF:metal ion binding;MF:electron carrier activity;MF:2 iron, 2 sulfur cluster binding;MF:3 iron, 4 sulfur cluster binding;MF:4 iron, 4 sulfur cluster binding;MF:electron carrier activity;MF:metal ion binding;MF:succinate dehydrogenas;
BAB_RS31470	-	ATPase	0.916	-0.126232407	0.320007732	0.48316957	no	down	mRNA	20.58	17.93	20.42	13.92	19.06	17.75	19.6433	16.91	COG2971	S:Function unknown	GO:0016301;GO:0045127;GO:0016301	MF:kinase activity;MF:N-acetylglucosamine kinase activity;MF:kinase activity;
BAB_RS17550	-	hypothetical protein	0.918	-0.124209599	0.320001388	0.483387145	no	down	mRNA	308.25	221.09	188.74	210.52	216.12	201.87	239.36	209.503333	ENOG41123KG	S:Function unknown	GO:0016021	CC:integral component of membrane;
BAB_RS33140	-	phosphotyrosine protein phosphatase	0.813	-0.298713549	0.320655857	0.483920746	no	down	mRNA	30.12	29.38	32.78	33.5	14.08	27.07	30.76	24.8833333	------	------	GO:0016740	MF:transferase activity;
BAB_RS24895	-	ComF family protein	0.831	-0.267925484	0.321359634	0.484755166	no	down	mRNA	2.75	2.84	4.83	3.34	2.46	2.58	3.47333	2.79333333	COG1040	S:Function unknown	GO:0009116;GO:0009116;GO:0016757	BP:nucleoside metabolic process;BP:nucleoside metabolic process;MF:transferase activity, transferring glycosyl groups;
BAB_RS21445	-	cell division protein	0.932	-0.100845053	0.32166259	0.48475699	no	down	mRNA	289.3	322.25	342.39	286.67	276.95	306.37	317.98	289.996667	COG2919	D:Cell cycle control, cell division, chromosome par	GO:0007049;GO:0007049;GO:0051301;GO:0016021;GO:0016021	BP:cell cycle;BP:cell cycle;BP:cell division;CC:integral component of membrane;CC:integral component of membrane;
BAB_RS28380	-	glucuronate isomerase	0.896	-0.158260181	0.322882999	0.485684961	no	down	mRNA	10.74	10.36	8.08	8.83	7.93	7.77	9.72667	8.17666667	COG1904	G:Carbohydrate transport and metabolism	GO:0006064;GO:0008880	BP:glucuronate catabolic process;MF:glucuronate isomerase activity;
BAB_RS21180	-	patatin family protein	0.958	-0.061195292	0.323504555	0.486164703	no	down	mRNA	83.69	84.53	92.28	80.72	77.1	77.97	86.8333	78.5966667	COG1752	S:Function unknown	GO:0016042;GO:0016787	BP:lipid catabolic process;MF:hydrolase activity;
BAB_RS25510	-	hypothetical protein	0.933	-0.099618018	0.325784856	0.489133992	no	down	mRNA	4.67	4.44	5.07	4.21	4	4.27	4.72667	4.16	------	------	------	------
BAB_RS32220	-	hypothetical protein	0.932	-0.101298888	0.32664695	0.489970425	no	down	mRNA	32.27	28.73	39.87	31.71	27.08	29.87	33.6233	29.5533333	------	------	------	------
BAB_RS32735	-	D-lyxose/D-mannose family sugar isomerase	0.932	-0.100898334	0.326641877	0.490191663	no	down	mRNA	206.99	181.71	183.15	161.54	167.64	192.66	190.617	173.946667	COG3822	S:Function unknown	------	------
BAB_RS22240	-	cobalt-precorrin-6A reductase	0.934	-0.09852553	0.328077635	0.491657388	no	down	mRNA	21.11	23.37	22.42	19.22	20.66	19.27	22.3	19.7166667	COG2099	H:Coenzyme transport and metabolism	GO:0009236;GO:0009236;GO:0016994;GO:0016994	BP:cobalamin biosynthetic process;BP:cobalamin biosynthetic process;MF:precorrin-6A reductase activity;MF:precorrin-6A reductase activity;
BAB_RS19110	-	hypothetical protein	0.672	-0.573977679	0.330063968	0.493483261	no	down	mRNA	6.58	24.53	13.3	15.95	7.84	7.83	14.8033	10.54	------	------	GO:0016021	CC:integral component of membrane;
BAB_RS23680	-	polyamine ABC transporter substrate-binding protein	0.955	-0.066698056	0.33083358	0.494403857	no	down	mRNA	222.26	202.79	200.85	175.7	192.06	195.98	208.633	187.913333	COG0687	E:Amino acid transport and metabolism	GO:0015846;GO:0042597;GO:0019808	BP:polyamine transport;CC:periplasmic space;MF:polyamine binding;
BAB_RS31550	-	flagellar biosynthetic protein FliQ	0.658	-0.604138366	0.333690637	0.495454215	no	down	mRNA	3.68	1.93	4.13	3.22	1.37	1.74	3.24667	2.11	COG1987	N:Cell motility	GO:0044780;GO:0009306;GO:0009306;GO:0044780;GO:0009425;GO:00	BP:bacterial-type flagellum assembly;BP:protein secretion;BP:protein secretion;BP:bacterial-type flagellum assembly;CC:bacterial-type flagellum basal body;CC:integral component of membrane;CC:plasma membrane;CC:integral component of membrane;CC:bacterial-type flagellum basal body;CC:plasma membrane;
BAB_RS19765	-	sugar ABC transporter substrate-binding protein	0.926	-0.111299009	0.33281093	0.496896616	no	down	mRNA	204.58	196.4	189.11	170.91	144.04	203.95	196.697	172.966667	COG1596	M:Cell wall/membrane/envelope biogenesis	GO:0016020;GO:0015159	CC:membrane;MF:polysaccharide transmembrane transporter activity;
BAB_RS33430	-	hypothetical protein	0.472	-1.083853636	0.33351228	0.497712474	no	down	mRNA	0	8.21	4.56	5.29	1.16	0	4.25667	2.15	------	------	------	------
BAB_RS17920	-	hypothetical protein	0.915	-0.128383258	0.334260302	0.498597192	no	down	mRNA	31.01	28.95	30.12	28.86	27.91	23.38	30.6933	26.7166667	COG0463	M:Cell wall/membrane/envelope biogenesis	GO:0016740	MF:transferase activity;
BAB_RS23565	-	ArsR family transcriptional regulator	0.914	-0.129501867	0.3349631	0.499182022	no	down	mRNA	84.19	66.23	81.95	57.87	74.15	72.04	77.4567	68.02	COG0640	P:Inorganic ion transport and metabolism	GO:0003700	MF:transcription factor activity, sequence-specific DNA binding;
BAB_RS25905	-	HAMP domain-containing protein	0.965	-0.051551776	0.334929113	0.499362988	no	down	mRNA	77.83	73.16	80.1	69.23	69.63	71.06	77.03	69.9733333	COG0642	C:Energy production and conversion	GO:0016021;GO:0005622;GO:0000155;GO:0005524	CC:integral component of membrane;CC:intracellular;MF:phosphorelay sensor kinase activity;MF:ATP binding;
BAB_RS18625	-	ABC transporter permease	0.948	-0.077382757	0.335868718	0.500067743	no	down	mRNA	36.09	34.11	38.85	34.88	31.74	30.96	36.35	32.5266667	COG1172	G:Carbohydrate transport and metabolism	GO:0016021;GO:0005886;GO:0022857	CC:integral component of membrane;CC:plasma membrane;MF:transmembrane transporter activity;
BAB_RS31305	-	peptide ABC transporter permease	0.823	-0.281229392	0.336380517	0.500597778	no	down	mRNA	4.11	2.25	3.68	2.49	2.02	3.26	3.34667	2.59	COG1173	S:Function unknown	GO:0055085;GO:0055085;GO:0016021;GO:0005886;GO:0016021;GO:00	BP:transmembrane transport;BP:transmembrane transport;CC:integral component of membrane;CC:plasma membrane;CC:integral component of membrane;CC:plasma membrane;
BAB_RS33220	-	hypothetical protein	0.905	-0.14435699	0.33803601	0.502828564	no	down	mRNA	51.71	49.29	50.46	45.71	49.45	36.3	50.4867	43.82	------	------	GO:0055085;GO:0016020;GO:0005215	BP:transmembrane transport;CC:membrane;MF:transporter activity;
BAB_RS22550	-	cobalt ECF transporter T component CbiQ	0.936	-0.095373045	0.340223218	0.504913256	no	down	mRNA	33.05	37.64	35.83	31.28	31.88	31.66	35.5067	31.6066667	COG0619	P:Inorganic ion transport and metabolism	GO:0006824;GO:0043190;GO:0005887	BP:cobalt ion transport;CC:ATP-binding cassett;CC:integral component of plasma membrane;
BAB_RS18010	-	enoyl-CoA hydratase	0.911	-0.134403408	0.342387263	0.507187771	no	down	mRNA	19.7	15.7	15.32	12.75	15.29	15.19	16.9067	14.41	COG1024	S:Function unknown	GO:0008152;GO:0008152;GO:0008152;GO:0004300;GO:0016853;GO:00	BP:metabolic process;BP:metabolic process;BP:metabolic process;MF:enoyl-CoA hydratase activity;MF:isomerase activity;MF:catalytic activity;MF:isomerase activity;MF:catalytic activity;
BAB_RS20495	-	glycosyltransferase family 2 protein	0.963	-0.054181195	0.3423098	0.507306914	no	down	mRNA	617.23	564.19	587.8	523.08	538.56	543.6	589.74	535.08	COG0463	S:Function unknown	GO:0016740;GO:0016740;GO:0016757	MF:transferase activity;MF:transferase activity;MF:transferase activity, transferring glycosyl groups;
BAB_RS23935	-	sensor histidine kinase	0.954	-0.067889418	0.342283857	0.507502554	no	down	mRNA	58.52	53.77	70.28	51.48	56.78	55.1	60.8567	54.4533333	COG3920	K:Transcription	GO:0016021;GO:0016021;GO:0016021;GO:0005524;GO:0016301;GO:00	CC:integral component of membrane;CC:integral component of membrane;CC:integral component of membrane;MF:ATP binding;MF:kinase activity;MF:kinase activity;MF:kinase activity;
BAB_RS30565	-	carbohydrate ABC transporter permease	0.757	-0.402454172	0.342818656	0.507592785	no	down	mRNA	2.5	3.36	2.98	3.35	1.98	1.07	2.94667	2.13333333	COG0395	G:Carbohydrate transport and metabolism	GO:0055085;GO:0055085;GO:0016021;GO:0005886;GO:0016021;GO:00	BP:transmembrane transport;BP:transmembrane transport;CC:integral component of membrane;CC:plasma membrane;CC:integral component of membrane;CC:plasma membrane;
BAB_RS16350	-	DUF2794 domain-containing protein	0.919	-0.121473682	0.343216383	0.5079476	no	down	mRNA	61.7	41.75	45.66	39.45	45.69	42.93	49.7033	42.69	ENOG4111V1P	S:Function unknown	------	------
BAB_RS23925	-	hypothetical protein	0.946	-0.079843108	0.343539192	0.507957398	no	down	mRNA	641.21	475.96	507.78	466.6	511.62	500.05	541.65	492.756667	ENOG410Y6KS	S:Function unknown	------	------
BAB_RS18245	-	YicC family protein	0.956	-0.065353827	0.346874941	0.511477369	no	down	mRNA	123.43	124.38	146.01	119.45	114.78	121.85	131.273	118.693333	COG1561	S:Function unknown	------	------
BAB_RS32665	-	hypothetical protein	0.896	-0.157960802	0.350045196	0.514970337	no	down	mRNA	17.39	15.19	26.23	16.48	16.04	17.62	19.6033	16.7133333	------	------	------	------
BAB_RS21355	-	RNA-binding protein Hfq	0.905	-0.1432929	0.349413302	0.514983917	no	down	mRNA	696.13	713.29	717.69	618.88	645.34	719.78	709.037	661.333333	COG1923	T:Signal transduction mechanisms	GO:0006355;GO:0009405;GO:0006355;GO:0016021;GO:0016021;GO:00	BP:regulation of transcription, DNA-templated;BP:pathogenesis;BP:regulation of transcription, DNA-templated;CC:integral component of membrane;CC:integral component of membrane;MF:RNA binding;MF:RNA binding;
BAB_RS32050	-	hypothetical protein	0.684	-0.548462096	0.350425414	0.515293755	no	down	mRNA	77.75	330.81	204.75	53.98	218.11	207.07	204.437	159.72	------	------	------	------
BAB_RS16740	-	non-canonical purine NTP pyrophosphatase	0.956	-0.064945888	0.35117893	0.516165554	no	down	mRNA	166.85	139.97	167.2	148.16	140.95	139.64	158.007	142.916667	COG0127	F:Nucleotide transport and metabolism	GO:0009143;GO:0009117;GO:0009143;GO:0009146;GO:0009117;GO:00	BP:nucleoside triphosphate catabolic process;BP:nucleotide metabolic process;BP:nucleoside triphosphate catabolic process;BP:purine nucleoside triphosphate catabolic process;BP:nucleotide metabolic process;BP:purine nucleoside triphosphate catabolic process;MF:metal ion binding;MF:nucleoside-triphosphatase activity;MF:nucleoside-triphosphate diphosphatase activity;MF:nucleotide binding;MF:nucleoside-triphosphatase activity;MF:nucleotide binding;MF:nucleoside-triphosphate diphosphatase activity;MF:metal ion binding;MF:metal ion binding;MF:nucleoside-triphosphatase activity;MF:nucleoside-triphosphate diphosphatase activity;MF:nucleotide binding;
BAB_RS24130	-	DUF1465 domain-containing protein	0.95	-0.074282914	0.351392404	0.516243161	no	down	mRNA	235.41	230.57	256.04	202.82	240.95	210.22	240.673	217.996667	COG5317	S:Function unknown	------	------
BAB_RS16420	-	YdcF family protein	0.96	-0.059061198	0.35300455	0.518137789	no	down	mRNA	546.54	472.57	484.7	447.57	445.44	470.82	501.27	454.61	COG1434	S:Function unknown	GO:0016021	CC:integral component of membrane;
BAB_RS18520	-	ABC transporter permease	0.955	-0.066107916	0.352850373	0.518148193	no	down	mRNA	347.21	305.42	298.64	293.38	275.29	290.65	317.09	286.44	COG1682	V:Defense mechanisms	GO:0055085;GO:0055085;GO:0043190;GO:0043190	BP:transmembrane transport;BP:transmembrane transport;CC:ATP-binding cassett;CC:ATP-binding cassett;
BAB_RS26415	-	DUF922 domain-containing protein	0.937	-0.094000187	0.356183388	0.521850081	no	down	mRNA	21.29	22.18	22.8	17.78	20	21.01	22.09	19.5966667	COG5664	O:Posttranslational modification, protein turnover,	------	------
BAB_RS32800	-	hypothetical protein	0.693	-0.529738056	0.357855079	0.52382158	no	down	mRNA	2.63	12.78	6.96	7.52	5.18	3.28	7.45667	5.32666667	COG0748	P:Inorganic ion transport and metabolism	GO:0048037	MF:cofactor binding;
BAB_RS21470	-	L%2CD-transpeptidase	0.949	-0.076064256	0.359064096	0.525112854	no	down	mRNA	126.8	118.43	125.21	119.3	115.2	96.83	123.48	110.443333	COG1376	F:Nucleotide transport and metabolism	GO:0016021;GO:0016740;GO:0016740	CC:integral component of membrane;MF:transferase activity;MF:transferase activity;
BAB_RS24580	-	DUF1674 domain-containing protein	0.96	-0.059149625	0.359006727	0.52526804	no	down	mRNA	1166.11	1048.42	1031.9	978.74	1077.41	937.34	1082.14	997.83	COG5508;ENOG410XVAZ	S:Function unknown;	------	------
BAB_RS29565	-	antibiotic biosynthesis monooxygenase	0.949	-0.07586125	0.359486182	0.525490947	no	down	mRNA	166.46	156.74	147.25	128.92	152.16	141.55	156.817	140.876667	COG2329	S:Function unknown	GO:0004497	MF:monooxygenase activity;
BAB_RS31345	-	ABC transporter ATP-binding protein	0.868	-0.204377177	0.360145896	0.5262159	no	down	mRNA	5.21	3.81	6.76	4.15	3.85	5.03	5.26	4.34333333	COG0444	S:Function unknown	GO:0015833;GO:0015833;GO:0005524;GO:0016887;GO:0016887;GO:00	BP:peptide transport;BP:peptide transport;MF:ATP binding;MF:ATPase activity;MF:ATPase activity;MF:ATP binding;
BAB_RS28225	-	hypothetical protein	0.956	-0.064651355	0.363794037	0.530099883	no	down	mRNA	48.21	43.15	45.96	44.18	39.3	40.02	45.7733	41.1666667	ENOG410XXMH	S:Function unknown	------	------
BAB_RS30345	-	methyltransferase domain-containing protein	0.95	-0.074554214	0.365883733	0.531938658	no	down	mRNA	53.25	48.17	52.07	50.91	43.76	42.53	51.1633	45.7333333	COG0144	F:Nucleotide transport and metabolism	GO:0008168;GO:0003723	MF:methyltransferase activity;MF:RNA binding;
BAB_RS32875	-	hypothetical protein	0.705	-0.504303679	0.365629113	0.532049973	no	down	mRNA	22.84	22.95	25.9	20.74	8.06	26.39	23.8967	18.3966667	------	------	------	------
BAB_RS18330	-	YqgE/AlgH family protein	0.962	-0.056103288	0.365873857	0.532165098	no	down	mRNA	62.69	61.88	72.03	55.77	62.29	59.72	65.5333	59.26	COG1678	K:Transcription	------	------
BAB_RS19300	-	LPS-assembly protein LptD	0.965	-0.051448579	0.365582455	0.532223121	no	down	mRNA	149.85	144.25	148.57	137.84	128.5	135.87	147.557	134.07	COG1452	M:Cell wall/membrane/envelope biogenesis	GO:0043165;GO:0015920;GO:0010033;GO:0015920;GO:0043165;GO:00	BP:Gram-negative-bacterium-type cell outer membrane assembly;BP:lipopolysaccharide transport;BP:response to organic substance;BP:lipopolysaccharide transport;BP:Gram-negative-bacterium-type cell outer membrane assembly;BP:response to organic substance;CC:cell outer membrane;CC:cell outer membrane;
BAB_RS20325	gatB	Asp-tRNA(Asn)/Glu-tRNA(Gln) amidotransferase GatCAB subunit B	0.962	-0.055320843	0.367683316	0.533830318	no	down	mRNA	99.87	99.6	101.34	94.32	87.46	91.23	100.27	91.0033333	COG0064	J:Translation, ribosomal structure and biogenesis	GO:0006412;GO:0006412;GO:0016787;GO:0050567;GO:0016740;GO:00	BP:translation;BP:translation;MF:hydrolase activity;MF:glutaminyl-tRNA synthas;MF:transferase activity;MF:carbon-nitrogen ligase activity, with glutamine as amido-N-donor;MF:ATP binding;MF:ATP binding;MF:glutaminyl-tRNA synthas;MF:transferase activity;
BAB_RS18840	-	ATP-binding protein	0.909	-0.138216112	0.368546211	0.534117716	no	down	mRNA	8.69	7.44	8.09	7.71	7.27	5.61	8.07333	6.86333333	COG5323	S:Function unknown	------	------
BAB_RS18965	-	cytochrome c-type biogenesis protein CcmH	0.911	-0.13423156	0.368408438	0.534158985	no	down	mRNA	51.01	34.77	47.36	38.49	32.65	43.67	44.38	38.27	COG3088	O:Posttranslational modification, protein turnover,	GO:0016021;GO:0016021;GO:0046872;GO:0046872	CC:integral component of membrane;CC:integral component of membrane;MF:metal ion binding;MF:metal ion binding;
BAB_RS20995	-	PAS domain-containing sensor histidine kinase	0.961	-0.056962384	0.372153134	0.537889348	no	down	mRNA	62.06	56.38	58.28	50.73	53.53	55.57	58.9067	53.2766667	COG0784	S:Function unknown	GO:0018106;GO:0000160;GO:0016021;GO:0005622;GO:0016021;GO:00	BP:peptidyl-histidine phosphorylation;BP:phosphorelay signal transduction system;CC:integral component of membrane;CC:intracellular;CC:integral component of membrane;CC:intracellular;CC:plasma membrane;MF:phosphorelay sensor kinase activity;MF:phosphorelay sensor kinase activity;MF:protein histidine kinase activity;
BAB_RS28720	-	MurR/RpiR family transcriptional regulator	0.932	-0.102310495	0.372096258	0.53804918	no	down	mRNA	18.92	16.87	21.79	15.7	17.69	17.32	19.1933	16.9033333	COG1737	K:Transcription	GO:1901135;GO:0006351;GO:1901135;GO:0006351;GO:0097367;GO:00	BP:carbohydrate derivative metabolic process;BP:transcription, DNA-templated;BP:carbohydrate derivative metabolic process;BP:transcription, DNA-templated;MF:carbohydrate derivative binding;MF:transcription factor activity, sequence-specific DNA binding;MF:DNA binding;MF:carbohydrate derivative binding;MF:DNA binding;MF:transcription factor activity, sequence-specific DNA binding;
BAB_RS21365	-	Trk system potassium transporter TrkA	0.962	-0.056402462	0.373126025	0.53832686	no	down	mRNA	86.73	79.49	83.72	79.12	73.59	73.73	83.3133	75.48	COG0569	P:Inorganic ion transport and metabolism	GO:0005886;GO:0015079	CC:plasma membrane;MF:potassium ion transmembrane transporter activity;
BAB_RS27220	-	DNA-3-methyladenine glycosylase I	0.916	-0.127069295	0.379343105	0.544363285	no	down	mRNA	11.43	10.23	10.55	9.04	8.74	10.09	10.7367	9.29	COG2818	L:Replication, recombination and repair	GO:0006284;GO:0006284;GO:0008725;GO:0008725	BP:base-excision repair;BP:base-excision repair;MF:DNA-3-methyladenine glycosylase activity;MF:DNA-3-methyladenine glycosylase activity;
BAB_RS32900	-	AzlD domain-containing protein	0.933	-0.099712553	0.378725498	0.544449676	no	down	mRNA	16.16	12.02	15.44	13.73	11.82	12.6	14.54	12.7166667	------	------	GO:0016021	CC:integral component of membrane;
BAB_RS22670	-	DUF2065 domain-containing protein	0.936	-0.094927613	0.379893945	0.544667222	no	down	mRNA	106.69	93.05	82.96	84.2	80.13	88.62	94.2333	84.3166667	COG3242	S:Function unknown	GO:0016021	CC:integral component of membrane;
BAB_RS16850	-	DeoR/GlpR transcriptional regulator	0.935	-0.097585458	0.379740626	0.54469046	no	down	mRNA	46.64	33.51	36.98	32.82	33.62	36.09	39.0433	34.1766667	COG1349	K:Transcription	GO:0006351;GO:0006351;GO:0005622;GO:0005622;GO:0003700;GO:00	BP:transcription, DNA-templated;BP:transcription, DNA-templated;CC:intracellular;CC:intracellular;MF:transcription factor activity, sequence-specific DNA binding;MF:DNA binding;MF:DNA binding;MF:transcription factor activity, sequence-specific DNA binding;
BAB_RS16860	-	sigma-54-dependent Fis family transcriptional regulator	0.947	-0.078079155	0.383396981	0.547977981	no	down	mRNA	160.69	160.29	209.64	144.71	166.65	162.02	176.873	157.793333	COG3284	K:Transcription	GO:0043565	MF:sequence-specific DNA binding;
BAB_RS25930	-	two-component sensor histidine kinase	0.968	-0.047258289	0.383660175	0.548110335	no	down	mRNA	120.5	114.18	122.84	102.51	112.72	110.34	119.173	108.523333	ENOG410Y097	T:Signal transduction mechanisms	GO:0005622;GO:0005524;GO:0000155	CC:intracellular;MF:ATP binding;MF:phosphorelay sensor kinase activity;
BAB_RS22325	-	GlxA family transcriptional regulator	0.928	-0.107784362	0.384886396	0.548156024	no	down	mRNA	11.84	11.3	14.95	10.45	11.02	12.04	12.6967	11.17	COG4977	K:Transcription	GO:0006351;GO:0006351;GO:0016021;GO:0016021;GO:0003700;GO:00	BP:transcription, DNA-templated;BP:transcription, DNA-templated;CC:integral component of membrane;CC:integral component of membrane;MF:transcription factor activity, sequence-specific DNA binding;MF:sequence-specific DNA binding;MF:transcription factor activity, sequence-specific DNA binding;MF:sequence-specific DNA binding;
BAB_RS19995	-	tRNA 2-thiocytidine(32) synthetase TtcA	0.948	-0.077017409	0.384765789	0.548470488	no	down	mRNA	43.49	36.93	36.66	35.2	35.75	33.3	39.0267	34.75	COG0037	D:Cell cycle control, cell division, chromosome par	GO:0008033;GO:0008033;GO:0034227;GO:0034227;GO:0005737;GO:00	BP:tRNA processing;BP:tRNA processing;BP:tRNA thio-modification;BP:tRNA thio-modification;CC:cytoplasm;CC:cytoplasm;CC:cytoplasm;MF:4 iron, 4 sulfur cluster binding;MF:ATP binding;MF:metal ion binding;MF:transferase activity;MF:tRNA binding;MF:4 iron, 4 sulfur cluster binding;MF:magnesium ion binding;MF:transferase activity;MF:metal ion binding;MF:ATP binding;MF:sulfurtransferase activity;MF:4 iron, 4 sulfur cluster binding;MF:ATP binding;MF:magnesium ion binding;MF:sulfurtransferase activity;MF:tRNA binding;
BAB_RS18740	-	hypothetical protein	0.927	-0.108912844	0.384598374	0.548475178	no	down	mRNA	17.17	14.34	20.91	16.17	15.3	14.36	17.4733	15.2766667	------	------	------	------
BAB_RS16325	-	ABC transporter ATP-binding protein	0.933	-0.100812675	0.384139692	0.548551479	no	down	mRNA	74.15	66.69	69.84	72.24	61.33	51.93	70.2267	61.8333333	COG4181	Q:Secondary metabolites biosynthesis, transport a	GO:0008643;GO:0005524;GO:0016887;GO:0005524;GO:0016887	BP:carbohydrate transport;MF:ATP binding;MF:ATPase activity;MF:ATP binding;MF:ATPase activity;
BAB_RS23365	-	ABC transporter ATP-binding protein	0.962	-0.055866625	0.384592067	0.54870973	no	down	mRNA	76.37	76.95	79.07	69.99	74.12	66.55	77.4633	70.22	COG1132	C:Energy production and conversion	GO:0016021;GO:0016021;GO:0005524;GO:0042626;GO:0042626;GO:00	CC:integral component of membrane;CC:integral component of membrane;MF:ATP binding;MF:ATPase activity, coupled to transmembrane movement of substances;MF:ATPase activity, coupled to transmembrane movement of substances;MF:ATP binding;
BAB_RS31335	-	ABC transporter permease	0.91	-0.135850643	0.387015693	0.550944361	no	down	mRNA	6.53	6.84	7.41	5.59	5.56	6.85	6.92667	6	COG0601	S:Function unknown	GO:0055085;GO:0016021;GO:0005886	BP:transmembrane transport;CC:integral component of membrane;CC:plasma membrane;
BAB_RS16115	-	hypothetical protein	0.957	-0.062993885	0.38903716	0.553576791	no	down	mRNA	30.4	25.43	30.68	27.03	25.96	24.73	28.8367	25.9066667	COG0303;COG2068	H:Coenzyme transport and metabolism;S:Function	------	------
BAB_RS31765	-	sulfite exporter TauE/SafE family protein	0.918	-0.123109863	0.390535796	0.554236534	no	down	mRNA	11.63	10.61	9.73	8.93	9.36	9.29	10.6567	9.19333333	COG0730	S:Function unknown	GO:0016021;GO:0005886	CC:integral component of membrane;CC:plasma membrane;
BAB_RS29720	-	S58 family peptidase	0.943	-0.085184783	0.391253408	0.554520158	no	down	mRNA	25.67	25.5	26.99	23.06	20.73	25.96	26.0533	23.25	COG3191;COG3191	E:Amino acid transport and metabolism;Q:Second	GO:0004177	MF:aminopeptidase activity;
BAB_RS31990	-	hypothetical protein	0.75	-0.41486541	0.392992066	0.556248242	no	down	mRNA	6.17	2.9	6.81	2.45	6.22	2.3	5.29333	3.65666667	------	------	------	------
BAB_RS28275	-	hypothetical protein	0.953	-0.069917238	0.392857082	0.55630225	no	down	mRNA	23.29	22.05	25.42	21.58	20.62	21.45	23.5867	21.2166667	------	------	GO:0016021	CC:integral component of membrane;
BAB_RS18030	-	4-hydroxybenzoate octaprenyltransferase	0.953	-0.069810544	0.395276977	0.558498209	no	down	mRNA	34.59	38.94	36.67	33.36	33.77	32.07	36.7333	33.0666667	COG0382	H:Coenzyme transport and metabolism	GO:0006744;GO:0006744;GO:0006744;GO:0016021;GO:0005886;GO:00	BP:ubiquinone biosynthetic process;BP:ubiquinone biosynthetic process;BP:ubiquinone biosynthetic process;CC:integral component of membrane;CC:plasma membrane;CC:integral component of membrane;CC:integral component of membrane;CC:plasma membrane;MF:4-hydroxybenzoate octaprenyltransferase activity;MF:4-hydroxybenzoate octaprenyltransferase activity;MF:hydrolase activity;MF:4-hydroxybenzoate octaprenyltransferase activity;
BAB_RS17210	-	abortive infection protein	0.913	-0.131747454	0.397675628	0.561147033	no	down	mRNA	29.33	23.53	25.96	21.69	20.58	26.31	26.2733	22.86	ENOG4111HWJ	S:Function unknown	------	------
BAB_RS29890	-	ATP-dependent dethiobiotin synthetase BioD	0.911	-0.133847619	0.400332971	0.564153437	no	down	mRNA	28.77	18.77	22.81	22.33	18.71	18.99	23.45	20.01	COG0132	H:Coenzyme transport and metabolism	GO:0009102;GO:0009102;GO:0009102;GO:0005737;GO:0005737;GO:00	BP:biotin biosynthetic process;BP:biotin biosynthetic process;BP:biotin biosynthetic process;CC:cytoplasm;CC:cytoplasm;CC:cytoplasm;MF:lyase activity;MF:magnesium ion binding;MF:ATP binding;MF:dethiobiotin synthase activity;MF:magnesium ion binding;MF:ATP binding;MF:dethiobiotin synthase activity;MF:lyase activity;MF:magnesium ion binding;MF:ATP binding;MF:dethiobiotin synthase activity;
BAB_RS18240	-	endolytic transglycosylase MltG	0.97	-0.044508922	0.400613607	0.564301411	no	down	mRNA	192.54	167.63	191.81	163.9	172.45	167.29	183.993	167.88	COG1559	S:Function unknown	GO:0071555;GO:0009252;GO:0071555;GO:0009252;GO:0005887;GO:00	BP:cell wall organization;BP:peptidoglycan biosynthetic process;BP:cell wall organization;BP:peptidoglycan biosynthetic process;CC:integral component of plasma membrane;CC:integral component of plasma membrane;MF:lyase activity;MF:lytic endotransglycosylase activity;MF:lyase activity;MF:lytic endotransglycosylase activity;
BAB_RS25950	-	double-strand break repair helicase AddA	0.964	-0.053062329	0.40143126	0.564957792	no	down	mRNA	18.96	16.73	18.45	16.48	16.49	16.02	18.0467	16.33	COG1074	L:Replication, recombination and repair	GO:0006281;GO:0004003;GO:0004527;GO:0005524;GO:0003677	BP:DNA repair;MF:ATP-dependent DNA helicase activity;MF:exonuclease activity;MF:ATP binding;MF:DNA binding;
BAB_RS31190	-	protein SlyX	0.942	-0.086581161	0.402156859	0.565236215	no	down	mRNA	26.49	26.88	30.16	26.17	26.22	21.98	27.8433	24.79	COG2900;ENOG41121VN	S:Function unknown;	------	------
BAB_RS31615	-	flagellar M-ring protein FliF	0.871	-0.199748041	0.401906353	0.565378771	no	down	mRNA	1.37	1.52	1.22	1.17	1.09	1.11	1.37	1.12333333	COG1766	N:Cell motility	GO:0071973;GO:0071973;GO:0009431;GO:0016021;GO:0016021;GO:00	BP:bacterial-type flagellum-dependent cell motility;BP:bacterial-type flagellum-dependent cell motility;CC:bacterial-type flagellum basal body, MS ring;CC:integral component of membrane;CC:integral component of membrane;CC:bacterial-type flagellum basal body, MS ring;CC:plasma membrane;MF:motor activity;MF:motor activity;
BAB_RS25230	-	-	0.911	-0.135194041	0.404984318	0.568464226	no	down	mRNA	14.04	12.32	14.85	14.04	11.06	10.44	13.7367	11.8466667	------	------	------	------
BAB_RS18345	-	insulinase family protein	0.97	-0.043213842	0.406379076	0.569426939	no	down	mRNA	221.82	210.72	225.9	191.18	207.01	204.12	219.48	200.77	COG0612	O:Posttranslational modification, protein turnover,	GO:0046872;GO:0004222	MF:metal ion binding;MF:metalloendopeptidase activity;
BAB_RS28515	-	hypothetical protein	0.951	-0.072767334	0.409790321	0.573706449	no	down	mRNA	386.78	338.2	345.27	286.29	317.06	368.52	356.75	323.956667	ENOG410Z6J3	S:Function unknown	------	------
BAB_RS17020	-	sarcosine oxidase subunit gamma	0.913	-0.132072081	0.411426291	0.575244853	no	down	mRNA	12.16	11.46	12.21	11.85	9.28	9.92	11.9433	10.35	COG4583	E:Amino acid transport and metabolism	GO:0008115	MF:sarcosine oxidase activity;
BAB_RS25415	-	DUF2125 domain-containing protein	0.961	-0.057690742	0.411096116	0.575283894	no	down	mRNA	51.65	52.33	55.88	46.63	52.44	45.52	53.2867	48.1966667	COG4093	S:Function unknown	GO:0016021	CC:integral component of membrane;
BAB_RS19785	-	N-acetyl-gamma-glutamyl-phosphate reductase	0.969	-0.045331437	0.411418692	0.575484657	no	down	mRNA	100.81	95.08	104.18	89.51	93.72	90.91	100.023	91.38	COG0002	E:Amino acid transport and metabolism	GO:0006526;GO:0005737;GO:0003942;GO:0051287	BP:arginine biosynthetic process;CC:cytoplasm;MF:N-acetyl-gamma-glutamyl-phosphate reductase activity;MF:NAD binding;
BAB_RS17590	-	hybrid sensor histidine kinase/response regulator	0.963	-0.054504834	0.412248358	0.575893033	no	down	mRNA	14.58	13.58	14.68	12.25	12.95	13.57	14.28	12.9233333	COG0784;ENOG410XNMH	S:Function unknown;	GO:0016021;GO:0005622;GO:0000155;GO:0022857	CC:integral component of membrane;CC:intracellular;MF:phosphorelay sensor kinase activity;MF:transmembrane transporter activity;
BAB_RS22510	ureE	urease accessory protein UreE	0.963	-0.053756376	0.414868872	0.579301906	no	down	mRNA	393.62	406.47	455.77	375.25	396.24	379.08	418.62	383.523333	COG2371	S:Function unknown	GO:0006457;GO:0065003;GO:0018307;GO:0019627;GO:0018307;GO:00	BP:protein folding;BP:macromolecular complex assembly;BP:enzyme active site formation;BP:urea metabolic process;BP:enzyme active site formation;BP:protein folding;BP:macromolecular complex assembly;BP:urea metabolic process;CC:cytoplasm;CC:cytoplasm;MF:hydrolase activity;MF:nickel cation binding;MF:unfolded protein binding;MF:nickel cation binding;MF:unfolded protein binding;
BAB_RS23520	-	choline ABC transporter ATP-binding protein	0.961	-0.056976325	0.415240329	0.579317055	no	down	mRNA	69.43	58.68	65.79	55.3	58.88	61.2	64.6333	58.46	COG4175	E:Amino acid transport and metabolism	GO:0055052;GO:0055052;GO:0055052;GO:0005524;GO:0016887;GO:00	CC:ATP-binding cassett;CC:ATP-binding cassett;CC:ATP-binding cassett;MF:ATP binding;MF:ATPase activity;MF:choline transmembrane transporter activity;MF:choline transmembrane transporter activity;MF:ATP binding;MF:ATPase activity;MF:choline transmembrane transporter activity;MF:ATPase activity;MF:ATP binding;
BAB_RS16530	-	S-(hydroxymethyl)glutathione dehydrogenase/class III alcohol dehydrogenase	0.97	-0.043267131	0.416520397	0.580598714	no	down	mRNA	388.85	356.17	371.1	339.51	334.36	348.37	372.04	340.746667	COG1062	C:Energy production and conversion	GO:0006069;GO:0006069;GO:0008270;GO:0051903;GO:0016740;GO:00	BP:ethanol oxidation;BP:ethanol oxidation;MF:zinc ion binding;MF:S;MF:transferase activity;MF:zinc ion binding;MF:S;
BAB_RS30645	-	ABC transporter permease	0.923	-0.115554956	0.416758315	0.580678432	no	down	mRNA	13.22	16.24	16.32	12.06	13.95	14.35	15.26	13.4533333	COG0600	O:Posttranslational modification, protein turnover,	GO:0055085;GO:0055085;GO:0016021;GO:0005886;GO:0016021;GO:00	BP:transmembrane transport;BP:transmembrane transport;CC:integral component of membrane;CC:plasma membrane;CC:integral component of membrane;CC:plasma membrane;
BAB_RS15980	-	ABC transporter substrate-binding protein	0.957	-0.064088967	0.418647374	0.582300438	no	down	mRNA	100.9	87.29	84.54	81.06	81.06	83.51	90.91	81.8766667	COG4166	E:Amino acid transport and metabolism	GO:0055085;GO:0043190	BP:transmembrane transport;CC:ATP-binding cassett;
BAB_RS24110	-	4-hydroxybenzoyl-CoA thioesterase	0.958	-0.061400413	0.42023532	0.583751008	no	down	mRNA	78.32	73.92	74.27	68.4	64.89	72.45	75.5033	68.58	COG0824	S:Function unknown	GO:0016787	MF:hydrolase activity;
BAB_RS18785	-	amidase	0.927	-0.109020198	0.420104681	0.583821947	no	down	mRNA	8.58	7.44	10.79	8.12	8.42	6.83	8.93667	7.79	COG0154	J:Translation, ribosomal structure and biogenesis	GO:0004040;GO:0016874;GO:0016740;GO:0016740;GO:0004040;GO:00	MF:amidase activity;MF:ligase activity;MF:transferase activity;MF:transferase activity;MF:amidase activity;MF:ligase activity;MF:amidase activity;
BAB_RS33250	-	hypothetical protein	0.962	-0.056078098	0.421911829	0.585573523	no	down	mRNA	331.03	332.54	338.42	308.35	326.37	288.48	333.997	307.733333	------	------	------	------
BAB_RS24865	-	aspartate kinase	0.964	-0.053350313	0.421769408	0.585628827	no	down	mRNA	82.07	73.32	77.5	74.13	67.41	69.74	77.63	70.4266667	COG0527	E:Amino acid transport and metabolism	GO:0009089;GO:0009088;GO:0004072;GO:0016491	BP:lysine biosynthetic process via diaminopimelate;BP:threonine biosynthetic process;MF:aspartate kinase activity;MF:oxidoreductase activity;
BAB_RS21175	-	phosphoribosyl-AMP cyclohydrolase	0.955	-0.067115073	0.424356887	0.588458645	no	down	mRNA	207.9	189.99	190.93	174.35	198.32	164.57	196.273	179.08	COG0139	E:Amino acid transport and metabolism	GO:0000105;GO:0000105;GO:0000105;GO:0005737;GO:0005737;GO:00	BP:histidine biosynthetic process;BP:histidine biosynthetic process;BP:histidine biosynthetic process;CC:cytoplasm;CC:cytoplasm;CC:cytoplasm;MF:magnesium ion binding;MF:phosphoribosyl-AMP cyclohydrolase activity;MF:zinc ion binding;MF:magnesium ion binding;MF:zinc ion binding;MF:phosphoribosyl-AMP cyclohydrolase activity;MF:zinc ion binding;MF:magnesium ion binding;MF:zinc ion binding;MF:metal ion binding;MF:phosphoribosyl-AMP cyclohydrolase activity;
BAB_RS23725	-	hypothetical protein	0.805	-0.313675855	0.425128795	0.58902062	no	down	mRNA	1.93	0.65	1.53	0.93	1.25	0.79	1.37	0.99	ENOG4111FIW	S:Function unknown	------	------
BAB_RS16950	-	bifunctional diguanylate cyclase/phosphodiesterase	0.968	-0.047267575	0.425523863	0.589059961	no	down	mRNA	95.16	86.5	93.45	80.45	81.27	88.8	91.7033	83.5066667	COG2202;COG5001	T:Signal transduction mechanisms;	GO:0005622;GO:0000155	CC:intracellular;MF:phosphorelay sensor kinase activity;
BAB_RS31940	-	hypothetical protein	0.878	-0.188488685	0.426502612	0.589399094	no	down	mRNA	7.81	5.15	6.16	4.36	5.67	5.55	6.37333	5.19333333	ENOG4111PQY	S:Function unknown	GO:0016021	CC:integral component of membrane;
BAB_RS31490	-	zinc import ATP-binding protein ZnuC	0.965	-0.052077432	0.430016055	0.593998961	no	down	mRNA	92.96	87.61	92.98	76.98	85.97	86.15	91.1833	83.0333333	COG1121	P:Inorganic ion transport and metabolism	GO:0005886;GO:0005886;GO:0015633;GO:0016887;GO:0005524;GO:00	CC:plasma membrane;CC:plasma membrane;MF:zinc-transporting ATPase activity;MF:ATPase activity;MF:ATP binding;MF:zinc-transporting ATPase activity;MF:ATP binding;MF:ATPase activity;
BAB_RS22360	-	Kef family K(+) transporter	0.97	-0.044540612	0.430677378	0.594401382	no	down	mRNA	124.95	113.22	126.64	107.41	117.73	107.58	121.603	110.906667	COG1226	P:Inorganic ion transport and metabolism	GO:0006813;GO:0016021;GO:0015299	BP:potassium ion transport;CC:integral component of membrane;MF:solut;
BAB_RS20710	-	hypothetical protein	0.883	-0.179725405	0.430608334	0.594561485	no	down	mRNA	101.58	57.67	91.72	62.9	68.81	88.06	83.6567	73.2566667	COG2929	S:Function unknown	------	------
BAB_RS28495	-	hypothetical protein	0.967	-0.048054056	0.434053923	0.598804318	no	down	mRNA	76.53	75.47	83.71	70.04	72.83	72.55	78.57	71.8066667	COG1525	K:Transcription	GO:0016021	CC:integral component of membrane;
BAB_RS19335	-	damage-inducible protein DinB	0.903	-0.146651003	0.435016593	0.599874813	no	down	mRNA	12.51	8.13	11.16	9.9	7.8	9.3	10.6	9	COG2318	S:Function unknown	------	------
BAB_RS32760	-	hypothetical protein	0.917	-0.125607729	0.439554255	0.605352688	no	down	mRNA	9.96	8.56	13.21	10.01	7.95	9.67	10.5767	9.21	------	------	------	------
BAB_RS29170	-	protocatechuate 3%2C4-dioxygenase subunit beta	0.944	-0.083853892	0.442958613	0.608997015	no	down	mRNA	69.48	63.04	65.46	48.34	63.97	64.22	65.9933	58.8433333	COG3485	Q:Secondary metabolites biosynthesis, transport a	GO:0019619;GO:0019619;GO:0008199;GO:0018578;GO:0008199;GO:00	BP:3,4-dihydroxybenzoate catabolic process;BP:3,4-dihydroxybenzoate catabolic process;MF:ferric iron binding;MF:protocatechuate 3,4-dioxygenase activity;MF:ferric iron binding;MF:indoleacetylglucose-inositol O-acyltransferase activity;MF:protocatechuate 3,4-dioxygenase activity;
BAB_RS24960	-	methyltransferase	0.936	-0.09599408	0.444195869	0.609915097	no	down	mRNA	16.4	17.57	20.07	17.84	13.81	16.44	18.0133	16.03	COG3897	S:Function unknown	GO:0008168	MF:methyltransferase activity;
BAB_RS16540	-	ATP-dependent helicase HrpB	0.956	-0.064450899	0.444144883	0.610105819	no	down	mRNA	19.05	17.06	22.88	16.37	18.97	17.54	19.6633	17.6266667	COG1643	L:Replication, recombination and repair	GO:0005524;GO:0008026;GO:0003676	MF:ATP binding;MF:ATP-dependent helicase activity;MF:nucleic acid binding;
BAB_RS30230	-	alcohol dehydrogenase	0.95	-0.074017703	0.445341912	0.610966509	no	down	mRNA	16.68	15.51	19.24	15.93	14.78	15.44	17.1433	15.3833333	COG1062	S:Function unknown	GO:0016021;GO:0008270;GO:0004022;GO:0016491	CC:integral component of membrane;MF:zinc ion binding;MF:alcohol dehydrogenas;MF:oxidoreductase activity;
BAB_RS30195	-	ABC transporter permease	0.873	-0.19672586	0.446657341	0.612248309	no	down	mRNA	4.92	3.87	4.3	3.61	2.6	4.64	4.36333	3.61666667	COG0601	K:Transcription	GO:0055085;GO:0015031;GO:0055085;GO:0016021;GO:0005886;GO:00	BP:transmembrane transport;BP:protein transport;BP:transmembrane transport;CC:integral component of membrane;CC:plasma membrane;CC:integral component of membrane;CC:plasma membrane;
BAB_RS27640	-	MaoC family dehydratase	0.966	-0.049763907	0.447441811	0.612800741	no	down	mRNA	229.34	217.7	273.39	216.09	224.87	214.92	240.143	218.626667	COG2030	E:Amino acid transport and metabolism	------	------
BAB_RS19885	-	NADH-quinone oxidoreductase subunit G	0.973	-0.040036767	0.447771161	0.612990515	no	down	mRNA	387.86	364.42	398.89	335.67	364.3	353.7	383.723	351.223333	COG1034	C:Energy production and conversion	GO:0042773;GO:0016020;GO:0051539;GO:0008137;GO:0046872;GO:00	BP:ATP synthesis coupled electron transport;CC:membrane;MF:4 iron, 4 sulfur cluster binding;MF:NADH dehydrogenas;MF:metal ion binding;MF:electron carrier activity;
BAB_RS20465	-	cysteine desulfurase	0.931	-0.103810482	0.448623564	0.613895873	no	down	mRNA	13.07	17.5	17.55	15.3	14.69	12.57	16.04	14.1866667	COG1104	E:Amino acid transport and metabolism	GO:0008483;GO:0003824	MF:transaminase activity;MF:catalytic activity;
BAB_RS26340	-	formamidopyrimidine-DNA glycosylase	0.956	-0.064834471	0.449023333	0.61418134	no	down	mRNA	33.68	30.53	35.99	33.37	28.74	28.1	33.4	30.07	COG0266	L:Replication, recombination and repair	GO:0140078;GO:0006284;GO:0006289;GO:0006284;GO:0140078;GO:00	old GO;BP:base-excision repair;BP:nucleotide-excision repair;BP:base-excision repair;BP:old GO;BP:nucleotide-excision repair;MF:damaged DNA binding;MF:oxidized purine nucleobase lesion DNA N-glycosylase activity;MF:zinc ion binding;MF:oxidized purine nucleobase lesion DNA N-glycosylase activity;MF:transferase activity, transferring acyl groups;MF:zinc ion binding;MF:damaged DNA binding;
BAB_RS31785	-	sugar ABC transporter permease	0.888	-0.170871789	0.450174754	0.614970869	no	down	mRNA	4.16	4.02	5.1	4.6	3.72	2.87	4.42667	3.73	COG1175	S:Function unknown	GO:0055085;GO:0055085;GO:0016021;GO:0005886;GO:0016021;GO:00	BP:transmembrane transport;BP:transmembrane transport;CC:integral component of membrane;CC:plasma membrane;CC:integral component of membrane;CC:plasma membrane;
BAB_RS22800	-	outer membrane protein assembly factor BamD	0.974	-0.037683465	0.44998361	0.61497122	no	down	mRNA	456.48	429.21	443.99	409.2	407.08	408.5	443.227	408.26	COG4105	M:Cell wall/membrane/envelope biogenesis	GO:0043165;GO:0051205;GO:0051205;GO:0043165;GO:0009279;GO:00	BP:Gram-negative-bacterium-type cell outer membrane assembly;BP:protein insertion into membrane;BP:protein insertion into membrane;BP:Gram-negative-bacterium-type cell outer membrane assembly;CC:cell outer membrane;CC:cell outer membrane;
BAB_RS28575	-	MFS transporter	0.953	-0.069034652	0.450469792	0.615112385	no	down	mRNA	18.87	15.3	17.3	15.2	15.29	15.52	17.1567	15.3366667	ENOG410ZVCH	G:Carbohydrate transport and metabolism	GO:0055085;GO:0016021;GO:0005215	BP:transmembrane transport;CC:integral component of membrane;MF:transporter activity;
BAB_RS26865	-	gfo/Idh/MocA family oxidoreductase	0.934	-0.097869423	0.450969131	0.61527126	no	down	mRNA	11.44	12.11	11.69	11.23	10.81	9.02	11.7467	10.3533333	COG0673	S:Function unknown	GO:0047115;GO:0016491	MF:trans-1,2-dihydrobenzene-1,2-diol dehydrogenase activity;MF:oxidoreductase activity;
BAB_RS28530	-	ABC transporter permease	0.923	-0.114891287	0.452532168	0.616879871	no	down	mRNA	13.8	11.19	16.77	11.1	13.18	12.15	13.92	12.1433333	ENOG410XP0C	S:Function unknown	GO:0055085;GO:0055085;GO:0016021;GO:0005886;GO:0016021;GO:00	BP:transmembrane transport;BP:transmembrane transport;CC:integral component of membrane;CC:plasma membrane;CC:integral component of membrane;CC:plasma membrane;
BAB_RS17950	-	tRNA1(Val) (adenine(37)-N6)-methyltransferase	0.957	-0.063798794	0.453064215	0.617343224	no	down	mRNA	41.08	39.18	44.88	39.65	37.88	35.83	41.7133	37.7866667	COG4123	S:Function unknown	GO:0008168;GO:0003676	MF:methyltransferase activity;MF:nucleic acid binding;
BAB_RS32265	-	hypothetical protein	0.87	-0.201348291	0.454349156	0.61883164	no	down	mRNA	13.12	11.14	12.57	10.36	11.18	9.45	12.2767	10.33	COG3293;ENOG410XYZH;ENOG4111IPS	L:Replication, recombination and repair;	------	------
BAB_RS25270	-	aspartate ammonia-lyase	0.93	-0.105423078	0.456047203	0.619830653	no	down	mRNA	5.94	6.29	6.18	4.75	5.51	5.92	6.13667	5.39333333	COG1027	E:Amino acid transport and metabolism	GO:0006099;GO:0016829;GO:0008797	BP:tricarboxylic acid cycle;MF:lyase activity;MF:aspartate ammonia-lyase activity;
BAB_RS33280	-	cobalamin biosynthesis protein CbiG	0.974	-0.037499927	0.456265303	0.61986487	no	down	mRNA	780.35	751.74	830.26	714.96	724.12	729.17	787.45	722.75	------	------	------	------
BAB_RS24495	-	FAD-binding oxidoreductase	0.944	-0.08295383	0.455723099	0.619914613	no	down	mRNA	78.11	78.64	74.18	59.07	65.86	81.17	76.9767	68.7	COG0665	E:Amino acid transport and metabolism	GO:0016021;GO:0016491;GO:0016491;GO:0008718;GO:0016491	CC:integral component of membrane;MF:oxidoreductase activity;MF:oxidoreductase activity;MF:D-amino-acid dehydrogenase activity;MF:oxidoreductase activity;
BAB_RS21645	-	DUF1489 domain-containing protein	0.967	-0.048808482	0.458736218	0.622169468	no	down	mRNA	50.77	47.63	58.61	44.77	48.88	48.99	52.3367	47.5466667	COG5458	S:Function unknown	------	------
BAB_RS31355	-	SMP-30/gluconolactonase/LRE family protein	0.927	-0.109717148	0.459391056	0.622794711	no	down	mRNA	13.7	17.76	14.32	13.13	13.23	14.08	15.26	13.48	COG3386	S:Function unknown	------	------
BAB_RS19605	-	zinc-finger domain-containing protein	0.972	-0.041160314	0.463336106	0.627084629	no	down	mRNA	2539.57	2222.97	2265.88	2268.81	2152.47	2124.05	2342.81	2181.77667	COG4391	S:Function unknown	------	------
BAB_RS16120	-	ubiquinol oxidase subunit II	0.964	-0.052424312	0.464213146	0.627742777	no	down	mRNA	452.95	392.13	397.75	388.23	387.76	353.94	414.277	376.643333	COG1622	C:Energy production and conversion	GO:0016021;GO:0005886;GO:0070469;GO:0016021;GO:0005886;GO:00	CC:integral component of membrane;CC:plasma membrane;CC:respiratory chain;CC:integral component of membrane;CC:plasma membrane;CC:respiratory chain;MF:copper ion binding;MF:cytochrome o ubiquinol oxidase activity;MF:cytochrome-c oxidase activity;MF:cytochrome-c oxidase activity;MF:copper ion binding;MF:cytochrome o ubiquinol oxidase activity;
BAB_RS30655	-	short-chain dehydrogenase	0.926	-0.111019791	0.466578842	0.629881437	no	down	mRNA	42.9	49.97	41.31	31.31	47.85	38.18	44.7267	39.1133333	COG3213	P:Inorganic ion transport and metabolism	GO:0016021	CC:integral component of membrane;
BAB_RS20270	-	DUF815 domain-containing protein	0.967	-0.049140333	0.467858798	0.631079059	no	down	mRNA	43.95	38.07	42.27	36.38	37.94	38.68	41.43	37.6666667	COG2607	S:Function unknown	GO:0008233	MF:peptidase activity;
BAB_RS18000	-	ribbon-helix-helix protein%2C CopG family	0.935	-0.096463206	0.469262225	0.632441077	no	down	mRNA	660.48	436.03	506.5	488.31	466.68	530.11	534.337	495.033333	ENOG410YW1G	K:Transcription	GO:0006355;GO:0006355;GO:0006355;GO:0003677;GO:0003677	BP:regulation of transcription, DNA-templated;BP:regulation of transcription, DNA-templated;BP:regulation of transcription, DNA-templated;MF:DNA binding;MF:DNA binding;
BAB_RS32565	-	hypothetical protein	0.91	-0.135373978	0.469700421	0.632501028	no	down	mRNA	21.27	15.82	17.54	15.04	16.64	15.43	18.21	15.7033333	COG0503	F:Nucleotide transport and metabolism	------	------
BAB_RS31645	-	sugar ABC transporter permease	0.954	-0.06821977	0.470660866	0.633528849	no	down	mRNA	26.72	25.91	26.92	21.08	24.7	25.82	26.5167	23.8666667	COG4214	S:Function unknown	GO:0016021;GO:0005886;GO:0022857	CC:integral component of membrane;CC:plasma membrane;MF:transmembrane transporter activity;
BAB_RS21300	-	MarC family protein	0.958	-0.062473742	0.471710011	0.634675154	no	down	mRNA	111.38	112.72	146.52	101.34	114.17	117.24	123.54	110.916667	COG2095	U:Intracellular trafficking, secretion, and vesicular t	GO:0016021;GO:0005886	CC:integral component of membrane;CC:plasma membrane;
BAB_RS30775	-	hypothetical protein	0.956	-0.064511481	0.474222759	0.636989016	no	down	mRNA	49.16	43.14	44.45	39.54	39.99	44.08	45.5833	41.2033333	COG3319	Q:Secondary metabolites biosynthesis, transport a	GO:0009058;GO:0009058;GO:0016788;GO:0016788	BP:biosynthetic process;BP:biosynthetic process;MF:hydrolase activity, acting on ester bonds;MF:hydrolase activity, acting on ester bonds;
BAB_RS29870	-	molybdopterin adenylyltransferase	0.965	-0.051204076	0.473886624	0.637070177	no	down	mRNA	47.99	43.13	43.52	42.15	39.82	40.31	44.88	40.76	COG0521	H:Coenzyme transport and metabolism	GO:0006777;GO:0006777;GO:0006777;GO:0016779;GO:0016779	BP:Mo-molybdopterin cofactor biosynthetic process;BP:Mo-molybdopterin cofactor biosynthetic process;BP:Mo-molybdopterin cofactor biosynthetic process;MF:nucleotidyltransferase activity;MF:nucleotidyltransferase activity;
BAB_RS19425	-	AI-2E family transporter	0.965	-0.051170862	0.474214152	0.63724386	no	down	mRNA	86.79	76.25	77.1	69.27	72.61	76.26	80.0467	72.7133333	COG0593;COG0628	F:Nucleotide transport and metabolism;S:Function	GO:0016021	CC:integral component of membrane;
BAB_RS21140	-	nitroreductase	0.972	-0.040339208	0.47381917	0.637246126	no	down	mRNA	233.89	220.14	230.89	209.77	216.26	205.39	228.307	210.473333	COG0778	C:Energy production and conversion	GO:0016491	MF:oxidoreductase activity;
BAB_RS16990	-	Glu/Leu/Phe/Val dehydrogenase	0.973	-0.039262202	0.474949919	0.637432786	no	down	mRNA	116.3	107.05	113.56	103.37	104.2	101.29	112.303	102.953333	COG0334	E:Amino acid transport and metabolism	GO:0006520;GO:0006520;GO:0016639;GO:0016639	BP:cellular amino acid metabolic process;BP:cellular amino acid metabolic process;MF:oxidoreductase activity, acting on the CH-NH2 group of donors, NAD or NADP as acceptor;MF:oxidoreductase activity, acting on the CH-NH2 group of donors, NAD or NADP as acceptor;
BAB_RS30365	-	hydrogen peroxide-inducible genes activator	0.966	-0.050036898	0.477439968	0.639172758	no	down	mRNA	34.97	33.17	41.96	32.08	33.28	34.59	36.7	33.3166667	ENOG410XNR2	P:Inorganic ion transport and metabolism	GO:0006351;GO:0006351;GO:0003700;GO:0003677;GO:0003677;GO:00	BP:transcription, DNA-templated;BP:transcription, DNA-templated;MF:transcription factor activity, sequence-specific DNA binding;MF:DNA binding;MF:DNA binding;MF:transcription factor activity, sequence-specific DNA binding;
BAB_RS25945	-	double-strand break repair protein AddB	0.961	-0.056676697	0.478285073	0.63950476	no	down	mRNA	22.11	24.72	28.3	22.07	23.06	22.96	25.0433	22.6966667	COG2887	L:Replication, recombination and repair	------	------
BAB_RS32140	-	hypothetical protein	0.93	-0.104398189	0.479785946	0.640978064	no	down	mRNA	10.73	7.26	11.98	8.78	8.52	8.81	9.99	8.70333333	------	------	------	------
BAB_RS19210	-	hypothetical protein	0.9	-0.151262258	0.480303022	0.641135692	no	down	mRNA	10.01	7.27	8.44	8.49	7.7	5.35	8.57333	7.18	COG5350	S:Function unknown	GO:0004725	MF:protein tyrosine phosphatase activity;
BAB_RS28000	-	prolyl-tRNA synthetase associated domain-containing protein	0.956	-0.064519899	0.479744993	0.641189959	no	down	mRNA	47.28	42.86	42.51	42.44	39.18	38.14	44.2167	39.92	COG3760	S:Function unknown	GO:0002161;GO:0002161;GO:0003677	MF:aminoacyl-tRNA editing activity;MF:aminoacyl-tRNA editing activity;MF:DNA binding;
BAB_RS18050	-	enoyl-[acyl-carrier-protein] reductase FabI	0.969	-0.045148803	0.481354353	0.642272232	no	down	mRNA	224.11	189.36	206.49	195.77	186.29	184.93	206.653	188.996667	COG0623	I:Lipid transport and metabolism	GO:0006633;GO:0004318	BP:fatty acid biosynthetic process;MF:enoyl-[acyl-carrier-protein] reductas;
BAB_RS30600	-	peptidyl-prolyl cis-trans isomerase	0.966	-0.050609596	0.481646975	0.642395903	no	down	mRNA	299.16	298.82	321.51	270.58	258.64	310.45	306.497	279.89	COG0760	C:Energy production and conversion	GO:0003755	MF:peptidyl-prolyl cis-trans isomerase activity;
BAB_RS33460	-	hypothetical protein	0.959	-0.060932595	0.481944728	0.642526311	no	down	mRNA	34.71	36.21	47.85	34.61	36.19	35.91	39.59	35.57	COG1653;ENOG410XSJ0	G:Carbohydrate transport and metabolism;	------	------

BAB_RS30215	-	ABC transporter ATP-binding protein	0.875	-0.192881757	0.482935735	0.642780661	no	down	mRNA	2.87	3.48	5.68	2.96	4.18	2.96	4.01	3.36666667	COG4608	K:Transcription	GO:0015031;[illegible]	[illegible]
BAB_RS32930	-	hypothetical protein	0.845	-0.242372282	0.483370241	0.642826401	no	down	mRNA	43.21	14.19	28.37	26.9	23.69	17.27	28.59	22.62	------	------	------	------
BAB_RS21375	-	two-component sensor histidine kinase	0.974	-0.037828459	0.483233574	0.642910755	no	down	mRNA	108.8	104.54	112.34	104.43	97.14	97	108.56	99.5233333	COG5000	T:Signal transduction mechanisms	GO:0006355;[illegible]	[illegible]
BAB_RS21195	-	integrase	0.909	-0.138290904	0.483735512	0.643046007	no	down	mRNA	3.88	4.83	6.32	4.12	5.04	3.86	5.01	4.34	ENOG410Z2SC;ENOG411073R	L:Replication, recombination and repair;	GO:0006310;[illegible]	[illegible]
BAB_RS26895	-	MFS transporter	0.889	-0.17042383	0.484186762	0.64337968	no	down	mRNA	7.01	4.5	4.34	5.18	4.48	3.24	5.28333	4.3	ENOG410XNN3;ENOG410ZVD3	P:Inorganic ion transport and metabolism;	GO:0016021;[illegible]	[illegible]
BAB_RS23730	-	MarC family protein	0.913	-0.131406151	0.490133156	0.648331754	no	down	mRNA	8.42	11.02	8.99	8.24	8.03	8.62	9.47667	8.29666667	COG2095	S:Function unknown	GO:0016021;[illegible]	[illegible]
BAB_RS19330	-	ABC transporter ATP-binding protein	0.962	-0.056024111	0.491693869	0.649593915	no	down	mRNA	25.2	24.99	28.13	26.09	22.07	22.84	26.1067	23.6666667	COG0488	S:Function unknown	GO:0006438;[illegible]	[illegible]
BAB_RS21595	-	DUF4440 domain-containing protein	0.972	-0.040566676	0.492383737	0.649703879	no	down	mRNA	933.95	818.03	838.35	755.71	847.88	808.34	863.443	803.976667	COG4875	S:Function unknown	GO:0005516;[illegible]	[illegible]
BAB_RS25825	-	DNA mismatch repair protein MutS	0.957	-0.062955408	0.492072506	0.649826947	no	down	mRNA	51.86	45.62	55.26	44.28	46.98	47.61	50.9133	46.29	COG2840	S:Function unknown	------	------
BAB_RS23560	-	hypothetical protein	0.91	-0.136586845	0.493715452	0.649859791	no	down	mRNA	20.49	13.34	17.38	15.93	12.23	16	17.07	14.72	------	------	------	------
BAB_RS17675	-	trans-3-hydroxy-L-proline dehydratase	0.946	-0.079552137	0.492716442	0.649875997	no	down	mRNA	12.39	10.88	11.97	10.56	11	9.77	11.7467	10.4433333	COG3938	E:Amino acid transport and metabolism	GO:0050346	[illegible]
BAB_RS31925	-	hypothetical protein	0.922	-0.117847084	0.493163553	0.649932114	no	down	mRNA	82.09	85.09	100.38	83.01	67.92	94.93	89.1867	81.9533333	------	------	------	------
BAB_RS23415	-	DUF1285 domain-containing protein	0.966	-0.049324364	0.493682524	0.650082766	no	down	mRNA	81.13	78.86	87.38	70.54	77.54	78.64	82.4567	75.5733333	COG3816	S:Function unknown	------	------
BAB_RS31340	-	ABC transporter permease	0.92	-0.120197517	0.493120442	0.650141969	no	down	mRNA	9.04	8.04	10.64	6.84	7.8	9.63	9.24	8.09	COG1173	S:Function unknown	GO:0055085;[illegible]	[illegible]
BAB_RS26185	-	1-acyl-sn-glycerol-3-phosphate acyltransferase	0.923	-0.115650732	0.495267915	0.65136955	no	down	mRNA	15.12	15.02	21.88	12.38	16.45	16.92	17.34	15.25	COG0204	S:Function unknown	GO:0008152;[illegible]	[illegible]
BAB_RS18390	-	A/G-specific adenine glycosylase	0.926	-0.110228852	0.497163595	0.652793882	no	down	mRNA	11.34	9.48	12.11	11.59	9.32	7.79	10.9767	9.56666667	COG1194	L:Replication, recombination and repair	GO:0006284;[illegible]	[illegible]
BAB_RS24590	-	MFS transporter	0.971	-0.042135091	0.497095671	0.652971542	no	down	mRNA	92.56	84.9	87.08	79.88	83.16	79	88.18	80.68	COG0144	J:Translation, ribosomal structure and biogenesis	GO:0006355;[illegible]	[illegible]
BAB_RS18150	-	LysM peptidoglycan-binding domain-containing protein	0.975	-0.036171518	0.499259304	0.655277837	no	down	mRNA	136.52	134.82	143.68	125.06	130.3	126.46	138.34	127.273333	COG1652	S:Function unknown	GO:0016021	[illegible]
BAB_RS16605	-	phosphate acetyltransferase	0.922	-0.117837603	0.500817335	0.656518195	no	down	mRNA	5.99	6.66	6.17	6.13	5.73	4.5	6.27333	5.45333333	COG0280	C:Energy production and conversion	GO:0008152;[illegible]	[illegible]
BAB_RS31810	-	ATP-binding protein	0.939	-0.090567944	0.502661662	0.658130367	no	down	mRNA	15.07	12.06	12.95	11.28	11.81	12.25	13.36	11.78	COG1116	P:Inorganic ion transport and metabolism	GO:0005886;[illegible]	[illegible]
BAB_RS26575	-	response regulator	0.973	-0.039596589	0.504656404	0.659935297	no	down	mRNA	331.16	319.66	330.39	308.05	290.59	311.72	327.07	303.453333	ENOG4111IFM	T:Signal transduction mechanisms	GO:0000160;[illegible]	[illegible]
BAB_RS16670	-	ribosomal subunit interface protein	0.974	-0.037782453	0.50454853	0.660062877	no	down	mRNA	1672.5	1592.42	1609.81	1416.22	1576.33	1516.59	1624.91	1503.04667	COG1544	J:Translation, ribosomal structure and biogenesis	GO:0006417;[illegible]	[illegible]
BAB_RS22645	-	acetolactate synthase 3 large subunit	0.973	-0.039394481	0.506534392	0.661852379	no	down	mRNA	78.1	75.42	77.21	69.72	68.82	73.05	76.91	70.53	COG0028	E:Amino acid transport and metabolism	GO:0009099;[illegible]	[illegible]
BAB_RS19105	-	hypothetical protein	0.958	-0.062151347	0.50792787	0.662595309	no	down	mRNA	81.28	80.6	83.24	84.35	70.89	67.09	81.7067	74.11	ENOG4111Y3F;COG2885	S:Function unknown;M:Cell wall/membrane/envelo[illegible]	------	------
BAB_RS32865	-	hypothetical protein	0.721	-0.471086484	0.507903347	0.662832435	no	down	mRNA	67.17	22.15	67.86	48.86	62.67	9.23	52.3933	40.2533333	------	------	------	------
BAB_RS21120	-	bile acid:sodium symporter	0.961	-0.057256099	0.50929173	0.663028497	no	down	mRNA	36.42	36.65	35.68	30.54	32.67	35.6	36.25	32.9366667	COG0385	S:Function unknown	GO:0016021	[illegible]
BAB_RS19240	-	TIGR02301 family protein	0.958	-0.062648919	0.50916375	0.663130576	no	down	mRNA	89.3	104.19	108.25	85.37	94.69	96.12	100.58	92.06	COG5451	S:Function unknown	------	------
BAB_RS25265	-	arginase	0.936	-0.096110537	0.511651358	0.665561058	no	down	mRNA	9.6	7.31	8.4	6.79	7.69	7.65	8.43667	7.37666667	COG0010	E:Amino acid transport and metabolism	GO:0016787;[illegible]	[illegible]
BAB_RS32705	ssrA	-	0.907	-0.141383146	0.511942329	0.665670053	no	down	mRNA	3309.23	3605.48	6119.82	4077.37	3939.11	3535.13	4344.84	3850.53667	------	------	GO:0016021;[illegible]	[illegible]
BAB_RS28390	-	mannitol dehydrogenase family protein	0.911	-0.134394283	0.512714405	0.66613481	no	down	mRNA	4.47	2.68	3.2	2.62	3.14	2.89	3.45	2.88333333	COG0246	G:Carbohydrate transport and metabolism	GO:0019594;[illegible]	[illegible]
BAB_RS22290	-	cobalt transporter	0.972	-0.040852019	0.512614735	0.666274735	no	down	mRNA	409.04	420.65	426.12	359.04	390.25	407.8	418.603	385.696667	COG5446	S:Function unknown	GO:0016021	[illegible]
BAB_RS28965	-	hypothetical protein	0.845	-0.243641068	0.514144441	0.667722753	no	down	mRNA	84.41	60.52	120.89	65.47	93.58	90.3	88.6067	83.1166667	COG0395	G:Carbohydrate transport and metabolism	GO:0055085;[illegible]	[illegible]
BAB_RS31495	-	membrane protein	0.969	-0.045856634	0.514862024	0.668384519	no	down	mRNA	62.16	59.97	61.25	53.45	58.33	56.11	61.1267	55.9633333	COG1108	P:Inorganic ion transport and metabolism	GO:0043190;[illegible]	[illegible]
BAB_RS22255	-	hypothetical protein	0.961	-0.056677037	0.516807806	0.670368785	no	down	mRNA	184.8	152.42	169.62	136.84	164.04	163.15	168.947	154.676667	COG4103	S:Function unknown	------	------
BAB_RS23930	-	RNA polymerase sigma factor	0.967	-0.0480738	0.516696128	0.670494613	no	down	mRNA	121.48	116.41	117.57	115.97	107.23	103.39	118.487	108.863333	COG1595	K:Transcription	GO:0006352;[illegible]	[illegible]
BAB_RS20415	-	IS5/IS1182 family transposase	0.719	-0.476094151	0.517846928	0.670904104	no	down	mRNA	19.6	41.31	56.38	56.22	0.6	32.2	39.0967	29.6733333	COG3293	L:Replication, recombination and repair	------	------
BAB_RS22775	-	50S ribosomal protein L11 methyltransferase	0.972	-0.040920992	0.518073617	0.67092726	no	down	mRNA	124.26	107.22	118.55	107.01	110.33	103.62	116.677	106.986667	COG2264	J:Translation, ribosomal structure and biogenesis	GO:0005737;[illegible]	[illegible]
BAB_RS24275	-	DUF1192 domain-containing protein	0.893	-0.164070786	0.517826075	0.671147712	no	down	mRNA	154.21	230.63	263.75	184.49	225.32	208.37	216.197	206.06	COG5509	S:Function unknown	------	------
BAB_RS10420	-	chromosomal replication initiator protein DnaA	0.971	-0.041841801	0.521393225	0.674139409	no	down	mRNA	357.57	325.3	327.29	296.81	303.33	324.46	336.72	308.2	COG0593	L:Replication, recombination and repair	GO:0006270;[illegible]	[illegible]
BAB_RS30320	-	cupredoxin domain-containing protein	0.968	-0.046341163	0.521240698	0.674213511	no	down	mRNA	535.4	552.29	532.31	485.91	546.45	478.47	540	503.61	ENOG41126MP;ENOG41123N3	S:Function unknown;	------	------
BAB_RS25360	-	YihY/virulence factor BrkB family protein	0.965	-0.05118456	0.521177386	0.674403118	no	down	mRNA	80.79	65.49	70.19	67.5	64.98	64.16	72.1567	65.5466667	COG1295	S:Function unknown	GO:0016021;[illegible]	[illegible]
BAB_RS18790	-	cysteine desulfuration protein SufE	0.972	-0.041444321	0.522760063	0.675634788	no	down	mRNA	165	162.11	165.48	157.12	153.97	141.73	164.197	150.94	COG2166	S:Function unknown	------	------
BAB_RS20360	-	3-dehydroquinate dehydratase	0.961	-0.057378753	0.524052677	0.676217371	no	down	mRNA	250.01	232.11	226.23	243.84	207.34	197.66	236.117	216.28	COG0757	E:Amino acid transport and metabolism	GO:0009073;[illegible]	[illegible]
BAB_RS17620	-	hypothetical protein	0.965	-0.051306498	0.523939226	0.6763426	no	down	mRNA	862.21	759.44	735.67	684.95	804.99	760.95	785.773	750.296667	------	------	------	------
BAB_RS31555	flgD	flagellar basal body rod modification protein FlgD	0.874	-0.194358072	0.523805623	0.676441908	no	down	mRNA	6.74	7.93	9.39	8.66	5.09	7.16	8.02	6.97	COG1843	N:Cell motility	------	[illegible]
BAB_RS21170	-	GTP cyclohydrolase I FolE	0.971	-0.04238589	0.525351304	0.676534565	no	down	mRNA	650.76	551.94	570.36	564.27	538.6	524.12	591.02	542.33	COG0302	H:Coenzyme transport and metabolism	GO:0035998;[illegible]	[illegible]
BAB_RS31515	-	flagellar protein FlgN	0.912	-0.133566507	0.526279532	0.677458387	no	down	mRNA	27.65	16.69	17.51	16.05	20.37	15.62	20.6167	17.3466667	ENOG410XXYI	S:Function unknown	GO:0044780	[illegible]
BAB_RS18120	-	pirin family protein	0.966	-0.05015968	0.526543677	0.677526967	no	down	mRNA	106.21	94.12	107.95	103.18	92.45	84.9	102.76	93.51	COG1741	S:Function unknown	------	------
BAB_RS29665	-	NAD-dependent epimerase/dehydratase family protein	0.903	-0.146921174	0.528335108	0.679559929	no	down	mRNA	3.73	2.47	3.29	2.99	2.72	2.23	3.16333	2.64666667	COG1088;COG0451	M:Cell wall/membrane/envelope biogenesis;	GO:0008460	[illegible]
BAB_RS28875	-	hydrogen peroxide-inducible genes activator	0.974	-0.037913938	0.529115276	0.680018953	no	down	mRNA	76.53	74.34	77.76	70.58	72.49	66.73	76.21	69.9333333	ENOG410XNR2	K:Transcription	GO:0006351;[illegible]	[illegible]
BAB_RS25655	-	alanine-phosphoribitol ligase	0.943	-0.085270345	0.528917791	0.68003716	no	down	mRNA	5.96	4.77	6.1	4.57	5.38	4.89	5.61	4.94666667	COG2303	S:Function unknown	GO:0050660;[illegible]	[illegible]
BAB_RS17270	-	translation initiation factor IF-1	0.951	-0.071808363	0.531059887	0.681972588	no	down	mRNA	1024.01	727.95	765.37	821.53	786.32	767.49	839.11	791.78	COG0361	J:Translation, ribosomal structure and biogenesis	GO:0005737;[illegible]	[illegible]
BAB_RS29475	-	succinate dehydrogenase assembly factor 2 family protein	0.947	-0.078059242	0.53354944	0.684349043	no	down	mRNA	97.67	90.22	84.28	83.84	84.06	84.48	90.7233	84.1266667	COG2938	S:Function unknown	------	------
BAB_RS22925	-	methylenetetrahydrofolate reductase [NAD(P)H]	0.973	-0.039995043	0.534528549	0.684511849	no	down	mRNA	632.78	593.76	618.61	590.55	567.28	531.26	615.05	563.03	COG0685	E:Amino acid transport and metabolism	GO:0009086;[illegible]	[illegible]
BAB_RS25685	-	acyl CoA:acetate/3-ketoacid CoA transferase	0.955	-0.066494812	0.534113954	0.684799735	no	down	mRNA	8.62	8.03	9.79	8.05	7.54	8.25	8.81333	7.94666667	COG4670	I:Lipid transport and metabolism	GO:0008152;[illegible]	[illegible]
BAB_RS25395	-	hypoxanthine phosphoribosyltransferase	0.965	-0.050832975	0.53698227	0.686832815	no	down	mRNA	48.15	38.77	46.05	42.71	38.06	39.91	44.3233	40.2266667	COG0634	F:Nucleotide transport and metabolism	GO:0032264;[illegible]	[illegible]
BAB_RS17115	-	AlpA family transcriptional regulator	0.865	-0.209293186	0.539194404	0.688839611	no	down	mRNA	40.77	42.7	54.13	33.44	51.44	42.41	45.8667	42.43	COG3311	K:Transcription	GO:0003677	[illegible]
BAB_RS16865	-	aldehyde dehydrogenase	0.969	-0.044929442	0.54016115	0.689800387	no	down	mRNA	1545.48	1480.46	1692.86	1291.23	1478.2	1534.37	1572.93	1434.6	COG1012	C:Energy production and conversion	GO:0016620	[illegible]
BAB_RS29380	-	aspartate carbamoyltransferase	0.966	-0.050335518	0.540913359	0.69048654	no	down	mRNA	55.68	45.89	51.97	49.62	46.18	43.66	51.18	46.4866667	COG0540	F:Nucleotide transport and metabolism	GO:0006207;[illegible]	[illegible]
BAB_RS30090	-	3-hydroxyacyl-CoA dehydrogenase	0.903	-0.146671803	0.54117454	0.690545591	no	down	mRNA	3.09	3.84	3.47	3.15	2.75	3.11	3.46667	3.00333333	ENOG410XNNW	S:Function unknown	GO:0004316;[illegible]	[illegible]
BAB_RS25800	-	DNA polymerase III subunit epsilon	0.977	-0.033518277	0.542862988	0.691327301	no	down	mRNA	46.11	43.84	49.19	42.44	43.12	42.36	46.38	42.64	COG0847	L:Replication, recombination and repair	GO:0006260;[illegible]	[illegible]
BAB_RS16335	-	BAX inhibitor (BI)-1/YccA family protein	0.97	-0.043400577	0.543103395	0.691359432	no	down	mRNA	3777.12	3094.44	3245.15	3079.97	3148.3	3059.39	3372.24	3095.88667	COG0670	S:Function unknown	GO:0016021	[illegible]
BAB_RS28180	-	FAD-binding oxidoreductase	0.924	-0.113870554	0.542754584	0.691463314	no	down	mRNA	3.57	5.02	4.92	4.12	4.35	3.43	4.50333	3.96666667	COG0277	S:Function unknown	GO:0050660;[illegible]	[illegible]
BAB_RS33455	-	hypothetical protein	0.762	-0.392349607	0.54239935	0.691559171	no	down	mRNA	2.89	2.44	1.72	0.34	2.03	2.58	2.35	1.65	------	------	------	------
BAB_RS27715	-	LysR family transcriptional regulator	0.929	-0.106310759	0.542350673	0.691771621	no	down	mRNA	12.74	12.43	11.88	12.67	11.62	8.03	12.35	10.7733333	ENOG410XSUY	E:Amino acid transport and metabolism	GO:0006351;[illegible]	[illegible]
BAB_RS25455	-	cell division ATP-binding protein FtsE	0.969	-0.045545551	0.544443743	0.692242875	no	down	mRNA	87.87	76.24	83.14	73.32	73	80.54	82.4167	75.62	COG2884	D:Cell cycle control, cell division, chromosome par[illegible]	GO:0051301;[illegible]	[illegible]
BAB_RS31265	-	MFS transporter	0.944	-0.083392716	0.54583415	0.692913917	no	down	mRNA	4.26	3.68	3.88	3.33	3.38	3.76	3.94	3.49	COG1071	S:Function unknown	GO:0016624	[illegible]
BAB_RS17935	-	4-diphosphocytidyl-2C-methyl-D-erythritol kinase	0.964	-0.05229815	0.545673397	0.692983646	no	down	mRNA	29.86	27.72	32.46	26.72	26.73	28.72	30.0133	27.39	COG1947	I:Lipid transport and metabolism	GO:0019288;[illegible]	[illegible]
BAB_RS16345	-	N-acetyltransferase	0.918	-0.123153884	0.548391003	0.695335553	no	down	mRNA	11.43	12.61	13.92	12.27	9.7	11.98	12.6533	11.3166667	COG1247	M:Cell wall/membrane/envelope biogenesis	GO:0016740;[illegible]	[illegible]
BAB_RS26335	-	pilus biosynthesis protein	0.929	-0.106246409	0.54998275	0.696803855	no	down	mRNA	318.66	264.13	274.35	272.5	293.49	245.25	285.713	270.413333	------	------	------	------
BAB_RS32600	-	hypothetical protein	0.846	-0.240920636	0.549799079	0.696845934	no	down	mRNA	7.56	5.91	3.74	4.98	4.89	3.38	5.73667	4.41666667	------	------	------	------
BAB_RS18275	-	protoheme IX farnesyltransferase	0.967	-0.048081925	0.551090704	0.697932373	no	down	mRNA	46.46	46.29	54.4	46.26	46.2	42.2	49.05	44.8866667	COG0109	O:Posttranslational modification, protein turnover, [illegible]	GO:0048034;[illegible]	[illegible]
BAB_RS16675	-	PTS IIA-like nitrogen-regulatory protein PtsN	0.98	-0.029442402	0.551733839	0.698196465	no	down	mRNA	252.12	228.98	265.24	226.52	235.48	226.33	248.78	229.443333	COG1762	G:Carbohydrate transport and metabolism	GO:0009401;[illegible]	[illegible]
BAB_RS29345	-	DUF983 domain-containing protein	0.973	-0.039012057	0.553650934	0.69924546	no	down	mRNA	485	486.48	535.59	456.41	489.14	458.3	502.357	467.95	COG5349	G:Carbohydrate transport and metabolism	GO:0016021	[illegible]
BAB_RS26385	-	ribokinase	0.978	-0.032583323	0.554221013	0.699690418	no	down	mRNA	41.06	39.24	42.18	36.75	38.01	37.9	40.8267	37.5533333	COG0524	L:Replication, recombination and repair	GO:0019303;[illegible]	[illegible]
BAB_RS24035	-	N-acetyltransferase	0.961	-0.057120097	0.555159791	0.699775758	no	down	mRNA	142.32	108.89	110.63	113.53	107.86	106.34	120.613	109.243333	ENOG41123CT	S:Function unknown	GO:0016740	[illegible]
BAB_RS16235	-	DNA-binding protein	0.964	-0.052807727	0.555429987	0.699841784	no	down	mRNA	69.21	75.35	91.89	68.22	71.92	75.37	78.8167	71.8366667	COG1272	S:Function unknown	GO:0016021;[illegible]	[illegible]
BAB_RS24635	-	chorismate mutase	0.921	-0.119213045	0.556572518	0.700731779	no	down	mRNA	28.04	19.1	27.48	22.52	21.2	22.82	24.8733	22.18	COG1605	E:Amino acid transport and metabolism	GO:0046417;[illegible]	[illegible]
BAB_RS24055	-	porin family protein	0.97	-0.043607538	0.557946958	0.701362901	no	down	mRNA	165.13	142.65	144	136.17	131.55	145.9	150.593	137.873333	COG3637	E:Amino acid transport and metabolism	------	------
BAB_RS33255	-	hypothetical protein	0.943	-0.084382837	0.557780834	0.701428501	no	down	mRNA	359.69	370.44	601.9	391.42	395.56	388.48	444.01	391.82	------	------	------	------
BAB_RS27735	-	amino acid ABC transporter substrate-binding protein	0.936	-0.095650174	0.558547624	0.701843377	no	down	mRNA	4.56	4.03	5.8	3.79	3.94	5.02	4.79667	4.25	COG0834	E:Amino acid transport and metabolism	------	------
BAB_RS25675	-	enoyl-CoA hydratase/isomerase family protein	0.892	-0.164539058	0.560189716	0.703356607	no	down	mRNA	2.31	3.01	4.07	2.33	2.65	3.22	3.13	2.73333333	ENOG410Z8FE	S:Function unknown	GO:0008152;[illegible]	[illegible]
BAB_RS25250	-	amino acid ABC transporter permease	0.82	-0.286097065	0.561642264	0.704629674	no	down	mRNA	1.59	1.69	3.18	1.66	1.21	2.54	2.15333	1.80333333	COG0765	E:Amino acid transport and metabolism	GO:0071705;[illegible]	[illegible]
BAB_RS16835	-	-	0.74	-0.433939466	0.561557297	0.70479828	no	down	mRNA	2.45	5.01	6.95	5.38	4.71	1.48	4.80333	3.85666667	------	------	------	------
BAB_RS29225	-	AsnC family transcriptional regulator	0.978	-0.031882808	0.562592495	0.705546325	no	down	mRNA	696.88	655.38	651.35	641.06	606.73	624.79	667.87	624.193333	COG1522	S:Function unknown	GO:0005622;[illegible]	[illegible]
BAB_RS25960	-	bifunctional folylpolyglutamate synthase/dihydrofolate synthase	0.979	-0.030492208	0.562867064	0.705615246	no	down	mRNA	125.85	114.59	127.43	113.43	116.51	108.95	122.623	112.963333	COG0285	H:Coenzyme transport and metabolism	GO:0005524;[illegible]	[illegible]
BAB_RS23990	-	efflux RND transporter periplasmic adaptor subunit	0.964	-0.052623691	0.563431696	0.706047597	no	down	mRNA	43.49	36.25	42	41.2	35.26	33.83	40.58	36.7633333	COG0845	V:Defense mechanisms	GO:0055085;[illegible]	[illegible]
BAB_RS22105	-	homoserine dehydrogenase	0.98	-0.02917533	0.564285881	0.706842314	no	down	mRNA	190.2	181.64	197.21	178.01	178.64	168.89	189.683	175.18	COG0460	E:Amino acid transport and metabolism	GO:0009097;[illegible]	[illegible]
BAB_RS24775	-	branched-chain amino acid ABC transporter permease	0.943	-0.08521321	0.568331223	0.711632198	no	down	mRNA	14.26	15.87	13.6	12.47	12.7	14.06	14.5767	13.0766667	COG1296	E:Amino acid transport and metabolism	GO:0016021	[illegible]
BAB_RS25690	-	gfo/Idh/MocA family oxidoreductase	0.902	-0.149369475	0.569449276	0.712754392	no	down	mRNA	3.09	4.12	2.8	2.53	3.5	2.43	3.33667	2.82	ENOG410XRWS	S:Function unknown	GO:0047061;[illegible]	[illegible]
BAB_RS33050	-	hypothetical protein	0.853	-0.22921041	0.570191003	0.713404864	no	down	mRNA	7.35	20.36	12.21	11.7	13.7	9.13	13.3067	11.51	------	------	------	------
BAB_RS32725	-	hypothetical protein	0.869	-0.20244851	0.571342717	0.713456724	no	down	mRNA	86.27	27.9	61.03	54.1	49.93	46.58	58.4	50.2033333	ENOG410ZEUG	S:Function unknown	GO:0016021	[illegible]
BAB_RS23360	-	ABC transporter ATP-binding protein	0.975	-0.036501893	0.571098888	0.713707011	no	down	mRNA	21.48	19.79	21.87	19.35	19.16	19.4	21.0467	19.3033333	COG1132	C:Energy production and conversion	GO:0016021;[illegible]	[illegible]
BAB_RS17080	-	FAA hydrolase family protein	0.973	-0.039175052	0.570971192	0.713825074	no	down	mRNA	313.3	274.65	292.25	249.88	265.49	293.92	293.4	269.763333	COG0179	S:Function unknown	GO:0008152;[illegible]	[illegible]
BAB_RS16020	-	methylcrotonoyl-CoA carboxylase	0.924	-0.114199237	0.570964155	0.714094134	no	down	mRNA	1.81	2.15	2.13	1.79	1.93	1.61	2.03	1.77666667	COG4799	I:Lipid transport and metabolism	GO:0016740;[illegible]	[illegible]
BAB_RS18705	-	magnesium transporter CorA	0.968	-0.046951915	0.572265195	0.714331031	no	down	mRNA	134.6	120.29	119.93	108.53	107.13	126.74	124.94	114.133333	COG0598	P:Inorganic ion transport and metabolism	GO:0016020;[illegible]	[illegible]
BAB_RS22335	-	FAD-dependent oxidoreductase	0.929	-0.105761007	0.573365545	0.715148873	no	down	mRNA	2.05	1.81	2.7	1.97	2.1	1.67	2.18667	1.91333333	COG0665	E:Amino acid transport and metabolism	GO:0016491	[illegible]
BAB_RS31680	-	5-carboxymethyl-2-hydroxymuconate semialdehyde dehydrogenase	0.913	-0.131907548	0.574080499	0.715762764	no	down	mRNA	3.07	2.54	2.23	2.56	1.7	2.45	2.61333	2.23666667	COG1012	Q:Secondary metabolites biosynthesis, transport a[illegible]	GO:1901023;[illegible]	[illegible]
BAB_RS27155	-	outermembrane transporter	0.974	-0.038219037	0.574690047	0.715967089	no	down	mRNA	18.77	17.03	21.9	16.78	18.31	17.6	19.2333	17.5633333	COG3468;COG4625	C:Energy production and conversion;S:Function u[illegible]	GO:0019867	[illegible]
BAB_RS22975	-	aromatic ring-hydroxylating dioxygenase subunit alpha	0.918	-0.123656423	0.57545209	0.716360932	no	down	mRNA	3.65	3.67	4.24	2.91	4.41	2.6	3.85333	3.30666667	COG4638	P:Inorganic ion transport and metabolism	GO:0019439;[illegible]	[illegible]
BAB_RS22220	-	threonine-phosphate decarboxylase	0.97	-0.043395578	0.57707485	0.718102824	no	down	mRNA	35.81	32.44	34.59	32.51	31.54	29.95	34.28	31.3333333	COG0079	H:Coenzyme transport and metabolism	GO:0009236;[illegible]	[illegible]
BAB_RS25425	-	-	0.94	-0.088567232	0.577761135	0.718400359	no	down	mRNA	31.34	19.72	22.67	23.24	21.06	20.31	24.5767	21.5366667	------	------	------	------
BAB_RS16545	-	ATP-dependent helicase	0.862	-0.214439837	0.581542198	0.721984189	no	down	mRNA	1.51	3.43	2.66	2.55	2	1.94	2.53333	2.16333333	COG1643	L:Replication, recombination and repair	GO:0008026	[illegible]
BAB_RS17895	-	F0F1 ATP synthase subunit C	0.962	-0.055885014	0.581471325	0.722175249	no	down	mRNA	3532.58	3097.7	3223.85	3171.01	3169.84	3176.63	3284.71	3172.49333	ENOG41121M2	C:Energy production and conversion	GO:0015986;[illegible]	[illegible]
BAB_RS22010	-	-	0.741	-0.432850676	0.58130076	0.722242592	no	down	mRNA	0	0	698.78	236.44	0	0	232.927	78.8133333	------	------	GO:0016021	[illegible]
BAB_RS19715	-	ubiquinol-cytochrome c chaperone	0.967	-0.048334517	0.581280365	0.72249664	no	down	mRNA	44.03	45.94	46.04	41.81	40.22	43.07	45.3367	41.7	COG5452	S:Function unknown	------	------
BAB_RS33330	-	IS5/IS1182 family transposase	0.873	-0.196179083	0.584345135	0.72294991	no	down	mRNA	4.91	5.35	4.73	3.09	3.68	5.98	4.99667	4.25	COG3293	L:Replication, recombination and repair	------	------
BAB_RS25670	-	sorbosone dehydrogenase	0.943	-0.085329482	0.584227137	0.723082354	no	down	mRNA	3.77	4.29	3.85	3.52	3.53	3.58	3.97	3.54333333	COG1012	S:Function unknown	GO:0016620	[illegible]
BAB_RS29470	-	DNA helicase RecG	0.972	-0.040395998	0.583778679	0.723084386	no	down	mRNA	12.47	12.92	14.42	11.79	12.33	12.37	13.27	12.1633333	COG1200	L:Replication, recombination and repair	GO:0006310;[illegible]	[illegible]
BAB_RS28870	-	DUF1612 domain-containing protein	0.968	-0.04715134	0.585894915	0.724030909	no	down	mRNA	18.18	15.85	17.47	15.56	16.26	14.96	17.1667	15.5933333	ENOG4110EWM	S:Function unknown	------	------
BAB_RS18315	-	ribonuclease HI	0.965	-0.052060485	0.587697105	0.725978777	no	down	mRNA	22.27	21.62	26.19	22.36	21.33	20.26	23.36	21.3166667	COG0328	L:Replication, recombination and repair	GO:0006401;[illegible]	[illegible]
BAB_RS16040	-	TIGR02300 family protein	0.973	-0.03999128	0.588047441	0.726132371	no	down	mRNA	347.75	298.6	321.75	320.87	297.73	274.2	322.7	297.6	COG4530	S:Function unknown	------	------
BAB_RS25420	-	gamma-glutamylcyclotransferase	0.961	-0.056794165	0.589921235	0.727328061	no	down	mRNA	28.55	26.81	33.69	27.64	26.53	27.2	29.6833	27.1233333	COG3703	P:Inorganic ion transport and metabolism	GO:0006751;[illegible]	[illegible]
BAB_RS32305	-	hypothetical protein	0.869	-0.202222433	0.589892061	0.727571283	no	down	mRNA	13.5	10.61	14.3	12.94	10.31	10.65	12.8033	11.3	COG1091	M:Cell wall/membrane/envelope biogenesis	GO:0019305;[illegible]	[illegible]
BAB_RS20945	-	DUF922 domain-containing protein	0.977	-0.033193368	0.590989473	0.727807274	no	down	mRNA	170.58	154.04	157.07	141.96	150.44	151.55	160.563	147.983333	COG5661	O:Posttranslational modification, protein turnover, [illegible]	------	------
BAB_RS33525	-	homoserine O-succinyltransferase	0.876	-0.191453447	0.589800248	0.728016979	no	down	mRNA	85.51	130.77	153.96	133.96	136.11	91.94	123.413	120.67	ENOG411226E	S:Function unknown	GO:0016740	[illegible]
BAB_RS32955	-	hypothetical protein	0.856	-0.224821595	0.590791499	0.728121629	no	down	mRNA	16.53	20.74	10.81	10.82	7.64	22.13	16.0267	13.53	------	------	------	------
BAB_RS33055	-	hypothetical protein	0.951	-0.072060245	0.59448031	0.731265404	no	down	mRNA	11.55	11.04	12.11	9.39	11.34	10.43	11.5667	10.3866667	------	------	------	------
BAB_RS30800	-	hypothetical protein	0.906	-0.141810086	0.597385027	0.733714867	no	down	mRNA	59.21	102.21	58.81	44.33	84.44	61.52	73.41	63.43	ENOG410ZKMU	S:Function unknown	GO:0016021	[illegible]
BAB_RS16340	-	N-acetyltransferase	0.954	-0.067838028	0.598589825	0.734632968	no	down	mRNA	46.11	47.35	39.8	43.66	37.19	40.54	44.42	40.4633333	COG1247	M:Cell wall/membrane/envelope biogenesis	GO:0016740	[illegible]
BAB_RS29790	-	N-acetylglucosamine kinase	0.967	-0.048882435	0.598531167	0.734841666	no	down	mRNA	37.31	35.49	43.1	32.03	37.91	36.01	38.6333	35.3166667	COG2971	G:Carbohydrate transport and metabolism	GO:0016301;[illegible]	[illegible]
BAB_RS23885	-	RNA polymerase sigma factor RpoH	0.98	-0.029727111	0.600109019	0.735093511	no	down	mRNA	826.27	812.11	876.17	810.67	754.59	755.74	838.183	773.666667	COG0568	C:Energy production and conversion	GO:0006950;[illegible]	[illegible]
BAB_RS30160	-	DNA-binding response regulator	0.896	-0.157901794	0.600020203	0.735265032	no	down	mRNA	1.91	4.2	4.81	2.94	3.36	3.35	3.64	3.21666667	COG2197	T:Signal transduction mechanisms	GO:0006355;[illegible]	[illegible]
BAB_RS20745	-	glycosyl transferase	0.976	-0.034405397	0.599741061	0.735764807	no	down	mRNA	263.16	238.89	242.63	243	217.26	225.59	248.227	228.616667	COG0438;ENOG410XU3R	S:Function unknown;	GO:0016740	[illegible]
BAB_RS18515	-	polysaccharide/polyol phosphate ABC transporter ATP-binding protein	0.969	-0.045267598	0.601195206	0.735862933	no	down	mRNA	310.04	251.63	244.3	235.67	252.39	249.78	268.657	245.946667	COG1134;COG1134	G:Carbohydrate transport and metabolism;M:Cell [illegible]	GO:0005524;[illegible]	[illegible]
BAB_RS18860	-	phage major capsid protein	0.952	-0.070589324	0.600988382	0.735890119	no	down	mRNA	6.74	7.1	6.84	5.81	6.69	6.05	6.89333	6.18333333	COG4653	S:Function unknown	------	------
BAB_RS18690	-	transcription elongation factor	0.977	-0.033956192	0.603692149	0.738075675	no	down	mRNA	249.7	253.1	277.91	235.5	250.53	241.23	260.237	242.42	COG0782	K:Transcription	GO:0032784;[illegible]	[illegible]
BAB_RS17190	-	hypothetical protein	0.854	-0.228118993	0.604100182	0.738293604	no	down	mRNA	60.07	86.33	142.48	75.14	121.69	95.6	96.2933	97.4766667	------	------	------	------
BAB_RS30040	-	membrane protein	0.956	-0.065354327	0.60536215	0.738992624	no	down	mRNA	30.6	32.55	35.2	27.17	33.7	28.82	32.7833	29.8966667	COG2391	S:Function unknown	GO:0016021	[illegible]
BAB_RS33320	-	hypothetical protein	0.954	-0.068666626	0.604931053	0.739027937	no	down	mRNA	62.3	52.01	57.06	52.85	49.35	56.1	57.1233	52.7666667	------	------	------	------
BAB_RS27575	-	2-hydroxyacid dehydrogenase	0.981	-0.027443086	0.606353395	0.739921557	no	down	mRNA	123.49	114.46	122.21	108.09	112.71	112.14	120.053	110.98	COG1052	K:Transcription	GO:0051287;[illegible]	[illegible]
BAB_RS20505	-	NAD(+) kinase	0.973	-0.039555975	0.609744931	0.743495432	no	down	mRNA	135.73	118.9	116.91	119.95	111.68	109.06	123.847	113.563333	COG0061	G:Carbohydrate transport and metabolism	GO:0019674;[illegible]	[illegible]
BAB_RS26975	fliI	flagellum-specific ATP synthase FliI	0.923	-0.115741831	0.611958654	0.745346154	no	down	mRNA	1.56	1.57	1.99	1.4	1.55	1.54	1.70667	1.49666667	COG1157;COG1157	N:Cell motility;U:Intracellular trafficking, secretion, [illegible]	GO:0030254;[illegible]	[illegible]
BAB_RS20125	-	alpha-D-ribose 1-methylphosphonate 5-triphosphate diphosphatase	0.96	-0.058412663	0.612320007	0.745503669	no	down	mRNA	12.23	13.85	16.6	13.23	11.98	13.73	14.2267	12.98	COG3454	P:Inorganic ion transport and metabolism	GO:0019700;[illegible]	[illegible]
BAB_RS20450	-	anhydro-N-acetylmuramic acid kinase	0.97	-0.044314841	0.611904119	0.745562357	no	down	mRNA	21.04	21.89	25.65	22.19	19.47	21.22	22.86	20.96	COG2377	O:Posttranslational modification, protein turnover, [illegible]	GO:0097175;[illegible]	[illegible]
BAB_RS19525	-	hypothetical protein	0.917	-0.124618348	0.612899839	0.745644522	no	down	mRNA	15.08	16.34	9.69	12.15	10.85	12.7	13.7033	11.9	ENOG410YJV0	S:Function unknown	------	------
BAB_RS21640	-	hypothetical protein	0.979	-0.030094783	0.614869228	0.747474397	no	down	mRNA	381.24	353.14	370.27	322.93	355.8	354.76	368.217	344.496667	------	------	------	------
BAB_RS25955	-	thioredoxin	0.978	-0.032452987	0.615412051	0.747851331	no	down	mRNA	336.49	300.69	343.18	275.79	310.14	320.71	326.787	302.213333	COG0526	L:Replication, recombination and repair	GO:0045454;[illegible]	[illegible]
BAB_RS22965	-	hypothetical protein	0.915	-0.127770459	0.61567383	0.747886585	no	down	mRNA	8.8	8.52	11.27	7.73	10.53	6.81	9.53	8.35666667	ENOG410ZEUG	S:Function unknown	GO:0016021	[illegible]
BAB_RS26845	-	glycosyl transferase	0.965	-0.05070162	0.616663672	0.748522998	no	down	mRNA	40.68	37.76	36.65	34.93	30.75	39.22	38.3633	34.9666667	ENOG410Y1RP	M:Cell wall/membrane/envelope biogenesis	GO:0016740	[illegible]
BAB_RS20570	-	hypothetical protein	0.97	-0.043533065	0.619139773	0.749264065	no	down	mRNA	778.72	831.23	833.08	783.01	778.76	776.1	814.343	779.29	COG0625	I:Lipid transport and metabolism	GO:0016740;[illegible]	[illegible]
BAB_RS31380	-	ABC transporter ATP-binding protein	0.949	-0.075114291	0.618250907	0.7493173	no	down	mRNA	9.04	9.75	9.31	7.34	8.05	9.93	9.36667	8.44	COG3842	S:Function unknown	GO:0043190;[illegible]	[illegible]
BAB_RS20985	-	RNA polymerase-binding protein DksA	0.979	-0.030400283	0.618487032	0.749320827	no	down	mRNA	242.84	209.38	233.35	222.41	202.23	206.72	228.523	210.453333	COG1734	T:Signal transduction mechanisms	GO:0010468;[illegible]	[illegible]
BAB_RS23720	-	hypothetical protein	0.953	-0.069002849	0.618023664	0.749324541	no	down	mRNA	25.23	23.64	28.04	23	25.35	21.63	25.6367	23.3266667	ENOG4111FIW	S:Function unknown	------	------
BAB_RS30865	-	GntR family transcriptional regulator	0.967	-0.048796552	0.6189579	0.749326199	no	down	mRNA	40.48	33.95	34.5	31.01	33.23	34.92	36.31	33.0533333	ENOG410XPZK	K:Transcription	GO:0006351;[illegible]	[illegible]
BAB_RS18880	-	phage major tail protein%2C TP901-1 family	0.868	-0.203781944	0.619513016	0.749433479	no	down	mRNA	2.91	2.74	4.7	2.02	3.67	3.1	3.45	2.93	COG5437	S:Function unknown	------	------
BAB_RS16940	-	glycine oxidase ThiO	0.925	-0.112534357	0.617647305	0.749433834	no	down	mRNA	4.05	3.27	5.17	4.09	3.92	2.87	4.16333	3.62666667	COG0665	E:Amino acid transport and metabolism	GO:0046416;[illegible]	[illegible]
BAB_RS16710	-	trimeric intracellular cation channel family protein	0.973	-0.039490603	0.617948639	0.749516412	no	down	mRNA	51.63	47.24	51.81	43.57	49.59	45.29	50.2267	46.15	COG2860	S:Function unknown	GO:0016021	[illegible]
BAB_RS28555	-	phosphogluconate dehydratase	0.982	-0.026906813	0.620829862	0.750179144	no	down	mRNA	133.3	127.43	134.33	115.1	124.07	125.91	131.687	121.693333	COG0129	H:Coenzyme transport and metabolism	GO:0009255;[illegible]	[illegible]
BAB_RS32045	-	glyoxalase/bleomycin resistance/extradiol dioxygenase family protein	0.946	-0.080187087	0.621952324	0.750688512	no	down	mRNA	90.09	123.29	131.9	121.43	103.21	94.83	115.093	106.49	ENOG4111NNF;COG0346	S:Function unknown;	GO:0051213	[illegible]
BAB_RS32275	-	hypothetical protein	0.755	-0.404872267	0.62356973	0.751793449	no	down	mRNA	15.06	9.94	55.8	14.7	35.15	33.25	26.9333	27.7	------	------	------	------
BAB_RS31225	-	CoA transferase	0.971	-0.04232386	0.626197368	0.752139119	no	down	mRNA	51.3	48.2	47.86	48.63	46.01	40.1	49.12	44.9133333	COG1804	L:Replication, recombination and repair	GO:0008152;[illegible]	[illegible]
BAB_RS31290	-	penicillin amidase	0.965	-0.05209074	0.625283348	0.752166005	no	down	mRNA	15.77	12.93	16.02	15.13	13.7	11.56	14.9067	13.4633333	COG2366	S:Function unknown	GO:0017000;[illegible]	[illegible]
BAB_RS20150	-	DUF1289 domain-containing protein	0.847	-0.240364789	0.625765796	0.752183128	no	down	mRNA	141.84	388.7	297.31	281.13	121.33	397.51	275.95	266.656667	ENOG410XVKB;COG3313	S:Function unknown;	------	------
BAB_RS25295	-	MFS transporter	0.947	-0.078419543	0.624890736	0.752257001	no	down	mRNA	773.89	756.26	743.01	519.55	617.82	895.97	757.72	677.78	ENOG410XPWC	G:Carbohydrate transport and metabolism	GO:0055085;[illegible]	[illegible]
BAB_RS16515	-	S-formylglutathione hydrolase	0.976	-0.035001335	0.62518898	0.752334155	no	down	mRNA	128.6	113.96	111.52	108.88	109.73	107.22	118.027	108.61	COG0627	S:Function unknown	GO:0046294;[illegible]	[illegible]
BAB_RS20750	-	glycoside hydrolase	0.98	-0.029672959	0.624738999	0.752356223	no	down	mRNA	230.02	208.33	215.99	207.56	190.75	205.63	218.113	201.313333	COG0438	S:Function unknown	GO:0016021;[illegible]	[illegible]
[illegible]	[illegible]	IS5/IS1182 family [illegible]	[illegible]	[illegible]	[illegible]	[illegible]	[illegible]	[illegible]	[illegible]	[illegible]	[illegible]	[illegible]	[illegible]	[illegible]	[illegible]	[illegible]	[illegible]	[illegible]	[illegible]	[illegible]	[illegible]

BAB_RS18805	-	peptidoglycan-binding protein	0.972	-0.041538977	0.627840398	0.752705671	no	down	mRNA	119.84	138.52	139.68	116.44	117.48	132.75	132.68	122.223333	COG3409	M:Cell wall/membrane/envelope biogenesis	GO:0016021	CC:integral component of membrane;
BAB_RS23845	gabD	NAD-dependent succinate-semialdehyde dehydrogenase	0.98	-0.028825787	0.62810973	0.752747692	no	down	mRNA	429.63	388.73	397.87	364.65	369.85	389.5	405.41	374.666667	COG1012	S:Function unknown	GO:0009450;GO:0009013	BP:gamma-aminobutyric acid catabolic process;MF:succinate-semialdehyde dehydrogenase [NA;
BAB_RS25780	-	phosphoenolpyruvate synthetase regulatory protein	0.972	-0.040610294	0.63144754	0.755339146	no	down	mRNA	44.3	37.65	39.58	34.08	38.16	38.89	40.51	37.0433333	COG1806	S:Function unknown	GO:0006470;GO:0005524;GO:0004674;GO:0043531;GO:0005524;GO:00	BP:protein dephosphorylation;MF:ATP binding;MF:protein serine/threonine kinase activity;MF:ADP binding;MF:ATP binding;MF:phosphotransferase activity, phosphate group as acceptor;MF:protein serine/threonine kinase activity;
BAB_RS29860	-	hydroxyacylglutathione hydrolase	0.978	-0.031679977	0.631446949	0.755619757	no	down	mRNA	74.49	70.68	80.15	63.99	74.41	69.08	75.1067	69.16	COG4529	S:Function unknown	GO:0016787;GO:0016787;GO:0004497;GO:0004416	MF:hydrolase activity;MF:hydrolase activity;MF:monooxygenase activity;MF:hydroxyacylglutathione hydrolase activity;
BAB_RS29270	-	hypothetical protein	0.903	-0.147585747	0.633131528	0.757071678	no	down	mRNA	400.47	272.27	489.06	415.75	383.8	343.04	387.267	380.863333	------	------	------	------
BAB_RS31665	-	2-oxo-hepta-3-ene-1%2C7-dioic acid hydratase	0.947	-0.078130756	0.634905211	0.758627907	no	down	mRNA	10.41	8.3	10.05	6.87	9.2	9.51	9.58667	8.52666667	COG3971	Q:Secondary metabolites biosynthesis, transport a	GO:0019439;GO:0047662;GO:0018817;GO:0016829	BP:aromatic compound catabolic process;MF:aminobenzoate decarboxylase activity;MF:2-oxo-hept-3-ene-1,7-dioate hydratase activity;MF:lyase activity;
BAB_RS23235	-	hypothetical protein	0.816	-0.292477606	0.635760064	0.759366946	no	down	mRNA	5.74	4.02	4.21	1.92	1.64	7.78	4.65667	3.78	------	------	------	------
BAB_RS21740	-	RNA methyltransferase	0.98	-0.028829615	0.636782473	0.760305494	no	down	mRNA	59.18	53.05	64.65	51.98	55.15	55.81	58.96	54.3133333	COG0565	J:Translation, ribosomal structure and biogenesis	GO:0008033;GO:0008033;GO:0005737;GO:0005737;GO:0008173;GO:00	BP:tRNA processing;BP:tRNA processing;CC:cytoplasm;CC:cytoplasm;MF:RNA methyltransferase activity;MF:RNA binding;MF:RNA binding;MF:RNA methyltransferase activity;
BAB_RS26620	-	amino acid permease	0.975	-0.037205392	0.638653234	0.761972834	no	down	mRNA	137.33	117.16	120.09	104.95	114.92	123.68	124.86	114.516667	COG1113	E:Amino acid transport and metabolism	GO:0006865;GO:0016021;GO:0022857	BP:amino acid transport;CC:integral component of membrane;MF:transmembrane transporter activity;
BAB_RS32300	-	hypothetical protein	0.945	-0.081532741	0.644726007	0.767223948	no	down	mRNA	16.33	14.86	20.67	18.33	14.07	14.63	17.2867	15.6766667	COG2801	L:Replication, recombination and repair	GO:0015074	BP:DNA integration;
BAB_RS31985	-	hypothetical protein	0.943	-0.084184415	0.644281764	0.767263643	no	down	mRNA	35.45	41.5	40.12	40.36	28.82	39.34	39.0233	36.1733333	COG0394	T:Signal transduction mechanisms	------	------
BAB_RS16575	-	L%2CD-transpeptidase	0.981	-0.027846702	0.645812699	0.767948262	no	down	mRNA	130.23	116.41	131.72	118.3	109.38	121.85	126.12	116.51	COG1376	S:Function unknown	GO:0016740	MF:transferase activity;
BAB_RS19015	-	uracil-DNA glycosylase	0.977	-0.033182209	0.647738471	0.769383996	no	down	mRNA	140.39	141.72	145.47	139.77	135.18	120.16	142.527	131.703333	COG1573	L:Replication, recombination and repair	GO:0008152;GO:0050407;GO:0003887;GO:0016798	BP:metabolic process;MF:[glycogen-synthase-D] phosphatase activity;MF:DNA-directed DNA polymerase activity;MF:hydrolase activity, acting on glycosyl bonds;
BAB_RS17240	-	hypothetical protein	0.819	-0.287365147	0.648483723	0.769984554	no	down	mRNA	2.39	0.84	3.9	2.83	1.2	1.9	2.37667	1.97666667	------	------	------	------
BAB_RS24610	-	NAD-glutamate dehydrogenase	0.983	-0.025352532	0.653204676	0.773588878	no	down	mRNA	262.37	244.93	258.1	227.77	235.98	243.65	255.133	235.8	COG2902	E:Amino acid transport and metabolism	GO:0019551;GO:0004352	BP:glutamate catabolic process to 2-oxoglutarate;MF:glutamate dehydrogenas;
BAB_RS25490	-	hypothetical protein	0.963	-0.054376579	0.655133769	0.775301953	no	down	mRNA	25.13	24.87	32.14	25.65	23.79	26.13	27.38	25.19	COG3786	S:Function unknown	GO:0016740	MF:transferase activity;
BAB_RS30200	-	ABC transporter permease	0.938	-0.093069426	0.656692633	0.776574689	no	down	mRNA	9.17	5.85	5.62	6.22	5.97	5.6	6.88	5.93	COG1173	K:Transcription	GO:0055085;GO:0015031;GO:0055085;GO:0016021;GO:0005886;GO:00	BP:transmembrane transport;BP:protein transport;BP:transmembrane transport;CC:integral component of membrane;CC:plasma membrane;CC:integral component of membrane;CC:plasma membrane;
BAB_RS10425	-	DNA polymerase III subunit beta	0.985	-0.02173769	0.661932524	0.782483149	no	down	mRNA	207.14	188.32	203.43	182.18	188.92	184.46	199.63	185.186667	COG0592	L:Replication, recombination and repair	GO:0006260;GO:0006260;GO:0005737;GO:0009360;GO:0005737;GO:00	BP:DNA replication;BP:DNA replication;CC:cytoplasm;CC:DNA polymerase III complex;CC:cytoplasm;CC:DNA polymerase III complex;MF:3'-5' exonuclease activity;MF:DNA-directed DNA polymerase activity;MF:DNA binding;MF:3'-5' exonuclease activity;MF:DNA binding;MF:DNA-directed DNA polymerase activity;
BAB_RS22840	-	undecaprenyldiphospho-muramoylpentapeptide beta-N-acetylglucosaminyltransferase	0.978	-0.031636976	0.664454173	0.784598037	no	down	mRNA	63.43	69.82	77.39	63.78	65.39	65.53	70.2133	64.9	COG0707	M:Cell wall/membrane/envelope biogenesis	GO:0005975;GO:0071555;GO:0008360;GO:0051301;GO:0009252;GO:00	BP:carbohydrate metabolic process;BP:cell wall organization;BP:regulation of cell shape;BP:cell division;BP:peptidoglycan biosynthetic process;BP:lipid glycosylation;BP:cell cycle;BP:carbohydrate metabolic process;BP:cell cycle;BP:cell division;BP:cell wall organization;BP:lipid glycosylation;BP:peptidoglycan biosynthetic process;BP:regulation of cell shape;BP:carbohydrate metabolic process;BP:cell wall organization;BP:regulation of cell shape;BP:cell cycle;BP:peptidoglycan biosynthetic process;BP:lipid glycosylation;BP:cell division;CC:plasma membrane;CC:plasma membrane;CC:plasma membrane;MF:undecaprenyldiphospho-muramoylpentapeptide beta-N-acetylglucosaminyltransferase activity;MF:UDP-N-acetyl-D-glucosamin;MF:oxidoreductase activity;MF:UDP-N-acetyl-D-glucosamin;MF:undecaprenyldiphospho-muramoylpentapeptide beta-N-acetylglucosaminyltransferase activity;MF:undecaprenyldiphospho-muramoylpentapeptide beta-N-acetylglucosaminyltransferase [illegible]
BAB_RS16180	-	lactoylglutathione lyase	0.955	-0.066074818	0.668893113	0.787524212	no	down	mRNA	27.24	22.5	20.45	23.68	19.68	19.74	23.3967	21.0333333	COG0346	E:Amino acid transport and metabolism	GO:0004462;GO:0046872;GO:0046872;GO:0051213;GO:0004462	MF:lactoylglutathione lyase activity;MF:metal ion binding;MF:metal ion binding;MF:dioxygenase activity;MF:lactoylglutathione lyase activity;
BAB_RS30860	-	dihydrodipicolinate synthase family protein	0.98	-0.02966899	0.672886945	0.790777525	no	down	mRNA	96.36	89.78	90.31	80	90.97	84.46	92.15	85.1433333	COG0329	C:Energy production and conversion	GO:0008152;GO:0008152;GO:0016829;GO:0008840;GO:0016829	BP:metabolic process;BP:metabolic process;MF:lyase activity;MF:4-hydroxy-tetrahydrodipicolinate synthase;MF:lyase activity;
BAB_RS19030	-	AMP nucleosidase	0.983	-0.025319818	0.672885793	0.791065515	no	down	mRNA	183.85	180.73	190.88	158.2	177.71	177.87	185.153	171.26	COG0775	F:Nucleotide transport and metabolism	GO:0044209;GO:0009116;GO:0008714	BP:AMP salvage;BP:nucleoside metabolic process;MF:AMP nucleosidase activity;
BAB_RS26260	-	30S ribosomal protein S15	0.978	-0.032599984	0.672789755	0.791242124	no	down	mRNA	2001.64	1743.17	1807.22	1899.43	1565.45	1697.07	1850.68	1720.65	COG0184	J:Translation, ribosomal structure and biogenesis	GO:0006412;GO:0006412;GO:0005840;GO:0005840;GO:0019843;GO:00	BP:translation;BP:translation;CC:ribosome;CC:ribosome;MF:rRNA binding;MF:structural constituent of ribosome;MF:structural constituent of ribosome;MF:rRNA binding;
BAB_RS31100	-	ABC transporter ATP-binding protein	0.98	-0.029690695	0.67458832	0.792197468	no	down	mRNA	71.06	58.23	66.24	62.84	57.21	59.88	65.1767	59.9766667	COG1132	G:Carbohydrate transport and metabolism	GO:0016021;GO:0016021;GO:0005524;GO:0042626;GO:0042626;GO:00	CC:integral component of membrane;CC:integral component of membrane;MF:ATP binding;MF:ATPase activity, coupled to transmembrane movement of substances;MF:ATPase activity, coupled to transmembrane movement of substances;MF:ATP binding;
BAB_RS29060	-	sulfite exporter TauE/SafE family protein	0.971	-0.042829479	0.675995506	0.793559942	no	down	mRNA	40.91	41.75	37.24	37.51	38.97	33.41	39.9667	36.63	COG0730	C:Energy production and conversion	GO:0016021;GO:0005886	CC:integral component of membrane;CC:plasma membrane;
BAB_RS25035	-	allantoin permease	0.948	-0.076683241	0.676289189	0.793614742	no	down	mRNA	2.55	2.58	2.81	2.25	2.19	2.71	2.64667	2.38333333	COG1953;COG1953	F:Nucleotide transport and metabolism;H:Coenzyr	GO:0016021;GO:0022857	CC:integral component of membrane;MF:transmembrane transporter activity;
BAB_RS30185	-	FadR family transcriptional regulator	0.966	-0.050345952	0.681667202	0.798758833	no	down	mRNA	34.12	32.42	29.26	24.59	32.02	30.55	31.9333	29.0533333	COG2186;ENOG4111STD	G:Carbohydrate transport and metabolism;K:Trans	GO:0006351;GO:0003700;GO:0003677	BP:transcription, DNA-templated;MF:transcription factor activity, sequence-specific DNA binding;MF:DNA binding;
BAB_RS32390	-	hypothetical protein	0.89	-0.167634422	0.681575438	0.798942678	no	down	mRNA	5.41	3.06	4.22	2.57	3	5.18	4.23	3.58333333	------	------	------	------
BAB_RS20920	-	kinesin	0.981	-0.027220506	0.683652402	0.799918124	no	down	mRNA	761.17	756.98	785.33	669.28	738.96	718.86	767.827	709.033333	ENOG410XPG5	S:Function unknown	GO:0006869;GO:0042157;GO:0005576;GO:0016021;GO:0008289	BP:lipid transport;BP:lipoprotein metabolic process;CC:extracellular region;CC:integral component of membrane;MF:lipid binding;
BAB_RS25275	glnQ	amino acid ABC transporter ATP-binding protein	0.942	-0.086547473	0.683286809	0.800073075	no	down	mRNA	4.34	5.05	5.31	3.69	4.82	4.69	4.9	4.4	COG1126	E:Amino acid transport and metabolism	GO:0015424;GO:0005524	MF:amino acid-transporting ATPase activity;MF:ATP binding;
BAB_RS24205	-	TIGR00282 family metallophosphoesterase	0.976	-0.035732879	0.68454366	0.800378013	no	down	mRNA	71.36	58.25	65.07	65.46	58.13	55.03	64.8933	59.54	COG1692	S:Function unknown	------	------
BAB_RS30980	-	YihA family ribosome biogenesis GTP-binding protein	0.979	-0.030907	0.685139511	0.800783285	no	down	mRNA	56.03	56.28	57.86	55.63	53.1	48.68	56.7233	52.47	COG0218	S:Function unknown	------	------
BAB_RS23640	-	hypothetical protein	0.885	-0.175704342	0.68677323	0.802400868	no	down	mRNA	9.71	7.59	13.02	13.94	5.99	7.59	10.1067	9.17333333	------	------	GO:0016021	CC:integral component of membrane;
BAB_RS31450	-	hypothetical protein	0.93	-0.105277879	0.68705009	0.802432548	no	down	mRNA	71.83	126.32	186.73	127.98	115.36	110.05	128.293	117.796667	------	------	------	------
BAB_RS21870	-	50S ribosomal protein L14	0.983	-0.024275144	0.689955602	0.804656025	no	down	mRNA	1225.39	1062.61	1147.52	1119.31	1087.31	1053.97	1145.17	1086.86333	COG0093	J:Translation, ribosomal structure and biogenesis	GO:0006412;GO:0015934;GO:0003735;GO:0019843	BP:translation;CC:large ribosomal subunit;MF:structural constituent of ribosome;MF:rRNA binding;
BAB_RS29990	-	ABC transporter ATP-binding protein	0.96	-0.059063504	0.691595611	0.805691334	no	down	mRNA	15.52	15.15	15.1	15.51	14.72	11.16	15.2567	13.7966667	COG1116	P:Inorganic ion transport and metabolism	GO:0005524;GO:0016887;GO:0016887;GO:0005524	MF:ATP binding;MF:ATPase activity;MF:ATPase activity;MF:ATP binding;
BAB_RS19750	-	-	0.819	-0.28817363	0.692670916	0.806359294	no	down	mRNA	10.29	5.65	9.04	13.74	1	6.29	8.32667	7.01	------	------	------	------
BAB_RS29390	-	Holliday junction resolvase RuvX	0.979	-0.030947215	0.696347722	0.808881139	no	down	mRNA	34.25	35.22	40.99	33.2	34.74	34.39	36.82	34.11	COG0816	L:Replication, recombination and repair	GO:0006364;GO:0000967;GO:0000967;GO:0006364;GO:0000967;GO:00	BP:rRNA processing;BP:rRNA 5'-end processing;BP:rRNA 5'-end processing;BP:rRNA processing;BP:rRNA 5'-end processing;CC:cytoplasm;CC:cytoplasm;CC:cytoplasm;MF:nuclease activity;MF:helicase activity;MF:nuclease activity;MF:nuclease activity;
BAB_RS17440	-	glucose-6-phosphate isomerase	0.986	-0.020091432	0.696292695	0.809109739	no	down	mRNA	242.62	226.77	243.72	212.41	228.96	220.68	237.703	220.683333	COG0166	G:Carbohydrate transport and metabolism	GO:0006096;GO:0006094;GO:0005737;GO:0004347	BP:glycolytic process;BP:gluconeogenesis;CC:cytoplasm;MF:glucose-6-phosphate isomerase activity;
BAB_RS28190	-	NAD(P)-dependent oxidoreductase	0.958	-0.061620783	0.697243089	0.809628495	no	down	mRNA	13.34	10.07	9.27	8.97	10.25	9.88	10.8933	9.7	COG0451	S:Function unknown	GO:0003824;GO:0050662;GO:0008460;GO:0003824;GO:0050662	MF:catalytic activity;MF:coenzyme binding;MF:dTDP-glucose 4,6-dehydratase activity;MF:catalytic activity;MF:coenzyme binding;
BAB_RS20390	-	N-acetylmuramoyl-L-alanine amidase	0.983	-0.025112068	0.697667057	0.809828126	no	down	mRNA	93.48	84.56	91.58	88	77.8	83.68	89.8733	83.16	COG0860	M:Cell wall/membrane/envelope biogenesis	GO:0009253;GO:0008745	BP:peptidoglycan catabolic process;MF:N-acetylmuramoyl-L-alanine amidase activity;
BAB_RS23370	-	tRNA (cytidine(34)-2'-O)-methyltransferase	0.958	-0.061353063	0.700688895	0.812162128	no	down	mRNA	32.35	27.3	23.94	27.52	22.03	26.28	27.8633	25.2766667	COG0219	J:Translation, ribosomal structure and biogenesis	GO:0005737;GO:0005737;GO:0003723;GO:0008757;GO:0008175;GO:00	CC:cytoplasm;CC:cytoplasm;MF:RNA binding;MF:S-adenosylmethionine-dependent methyltransferase activity;MF:tRNA methyltransferase activity;MF:S-adenosylmethionine-dependent methyltransferase activity;MF:RNA binding;MF:tRNA methyltransferase activity;
BAB_RS28630	-	LysR family transcriptional regulator	0.982	-0.025618169	0.70057199	0.81231967	no	down	mRNA	121.22	127.39	123.71	114.6	118.92	112.53	124.107	115.35	ENOG410XR9D	S:Function unknown	GO:0006351;GO:0006351;GO:0016021;GO:0016021;GO:0003677;GO:00	BP:transcription, DNA-templated;BP:transcription, DNA-templated;CC:integral component of membrane;CC:integral component of membrane;MF:DNA binding;MF:transcription factor activity, sequence-specific DNA binding;MF:transcription factor activity, sequence-specific DNA binding;MF:DNA binding;
BAB_RS26055	-	phenylalanine--tRNA ligase subunit beta	0.985	-0.021846863	0.702647675	0.813552065	no	down	mRNA	69.31	68.98	76.18	65.42	69.58	63.77	71.49	66.2566667	COG0073	J:Translation, ribosomal structure and biogenesis	GO:0006432;GO:0005737;GO:0004826;GO:0000049;GO:0000287;GO:00	BP:phenylalanyl-tRNA aminoacylation;CC:cytoplasm;MF:phenylalanine-tRNA ligase activity;MF:tRNA binding;MF:magnesium ion binding;MF:transferase activity;MF:ATP binding;
BAB_RS26985	-	phosphatase PAP2 family protein	0.983	-0.024275417	0.702217632	0.813640552	no	down	mRNA	100.19	85.8	93.97	89.97	83.54	85.4	93.32	86.3033333	COG0671	S:Function unknown	GO:0016021	CC:integral component of membrane;
BAB_RS28065	-	DUF1737 domain-containing protein	0.945	-0.081301889	0.703565116	0.814027626	no	down	mRNA	581.38	551.59	594.01	592.1	625.49	498.87	575.66	572.153333	COG5515	S:Function unknown	------	------
BAB_RS17445	-	peptidase M14	0.986	-0.020742754	0.706327016	0.816047718	no	down	mRNA	396.85	359.84	373.25	338.17	362.05	351.11	376.647	350.443333	COG3608	S:Function unknown	GO:0008152;GO:0008152;GO:0008152;GO:0046872;GO:0016788;GO:00	BP:metabolic process;BP:metabolic process;BP:metabolic process;MF:metal ion binding;MF:hydrolase activity, acting on ester bonds;MF:hydrolase activity, acting on ester bonds;MF:metal ion binding;MF:metal ion binding;MF:hydrolase activity, acting on ester bonds;
BAB_RS25750	-	tRNA uridine-5-carboxymethylaminomethyl(34) synthesis GTPase MnmE	0.968	-0.047485806	0.706268176	0.816273255	no	down	mRNA	11.16	8.23	10.73	9.56	8.12	9.66	10.04	9.11333333	COG0486	S:Function unknown	GO:0006400;GO:0005737;GO:0046872;GO:0003924;GO:0005525	BP:tRNA modification;CC:cytoplasm;MF:metal ion binding;MF:GTPase activity;MF:GTP binding;
BAB_RS32080	-	hypothetical protein	0.893	-0.163322608	0.706084361	0.816354463	no	down	mRNA	6.82	10.4	12.9	7.01	7.37	14.44	10.04	9.60666667	ENOG4112B6W	S:Function unknown	------	------
BAB_RS27365	-	3-deoxy-D-manno-octulosonic acid transferase	0.985	-0.021401699	0.708135955	0.817549703	no	down	mRNA	100.36	101.12	110.5	96.97	98.68	94.28	103.993	96.6433333	COG1519	M:Cell wall/membrane/envelope biogenesis	GO:0016740	MF:transferase activity;
BAB_RS21025	-	xanthine phosphoribosyltransferase	0.969	-0.045844231	0.712010874	0.820254908	no	down	mRNA	104.03	82.38	90.04	99.22	82.75	70.29	92.15	84.0866667	COG0503	F:Nucleotide transport and metabolism	GO:0032265;GO:0006166;GO:0006166;GO:0032265;GO:0006166;GO:00	BP:XMP salvage;BP:purine ribonucleoside salvage;BP:purine ribonucleoside salvage;BP:XMP salvage;BP:purine ribonucleoside salvage;BP:XMP salvage;CC:plasma membrane;CC:plasma membrane;CC:plasma membrane;MF:magnesium ion binding;MF:xanthine phosphoribosyltransferase activity;MF:metal ion binding;MF:metal ion binding;MF:xanthine phosphoribosyltransferase activity;MF:magnesium ion binding;MF:xanthine phosphoribosyltransferase activity;
BAB_RS31445	-	-	0.918	-0.123205128	0.711568335	0.820627804	no	down	mRNA	3.05	2.04	1.2	2.16	1.93	1.14	2.09667	1.74333333	ENOG41129MV;ENOG4111PC4	S:Function unknown;	------	------
BAB_RS29840	-	Na+/H+ antiporter subunit E	0.979	-0.030901366	0.718205703	0.825320073	no	down	mRNA	116.77	102.65	95.26	96.91	96.61	98.61	104.893	97.3766667	COG1863	P:Inorganic ion transport and metabolism	GO:0016021;GO:0008324	CC:integral component of membrane;MF:cation transmembrane transporter activity;
BAB_RS23410	-	CoA pyrophosphatase	0.976	-0.034482654	0.719398078	0.826394718	no	down	mRNA	44.44	45.89	46.81	40.55	46	40.66	45.7133	42.4033333	COG0494	J:Translation, ribosomal structure and biogenesis	GO:0016787	MF:hydrolase activity;
BAB_RS29670	-	hypothetical protein	0.983	-0.024830728	0.719938578	0.826720032	no	down	mRNA	564.14	568.65	557.36	479.93	534.75	570.62	563.383	528.433333	COG5455	S:Function unknown	------	------
BAB_RS27480	-	hypothetical protein	0.971	-0.042253011	0.721369935	0.827772	no	down	mRNA	94.17	104.06	96.6	76.7	106.37	89.59	98.2767	90.8866667	ENOG410Y7VJ	S:Function unknown	------	------
BAB_RS27210	-	hypothetical protein	0.974	-0.038715125	0.722042543	0.827952423	no	down	mRNA	28.93	24.84	34.85	26.82	29.5	24.91	29.54	27.0766667	------	------	------	------
BAB_RS33240	-	hypothetical protein	0.97	-0.043202175	0.722020578	0.82822282	no	down	mRNA	9.22	9.19	10.2	7.84	10.08	8.18	9.53667	8.7	------	------	------	------
BAB_RS20950	-	dihydropteroate synthase	0.978	-0.032678529	0.726514806	0.831893111	no	down	mRNA	27.36	25.08	29.24	24.09	24.57	26.92	27.2267	25.1933333	COG0294	H:Coenzyme transport and metabolism	GO:0046656;GO:0046654;GO:0046654;GO:0046656;GO:0046654;GO:00	BP:folic acid biosynthetic process;BP:tetrahydrofolate biosynthetic process;BP:tetrahydrofolate biosynthetic process;BP:folic acid biosynthetic process;BP:tetrahydrofolate biosynthetic process;BP:folic acid biosynthetic process;MF:dihydropteroate synthase activity;MF:metal ion binding;MF:metal ion binding;MF:transferase activity, transferring acyl groups;MF:dihydropteroate synthase activity;MF:metal ion binding;MF:dihydropteroate synthase activity;
BAB_RS26250	-	ribosome-binding factor A	0.982	-0.026147771	0.729512327	0.834730452	no	down	mRNA	82.83	78.87	87.42	83.33	73.59	75.02	83.04	77.3133333	COG0858	J:Translation, ribosomal structure and biogenesis	GO:0030490;GO:0030490;GO:0006364;GO:0006364;GO:0005737;GO:00	BP:maturation of SSU-rRNA;BP:maturation of SSU-rRNA;BP:rRNA processing;BP:rRNA processing;CC:cytoplasm;CC:cytoplasm;CC:cytoplasm;
BAB_RS21410	-	ubiquinone-binding protein	0.98	-0.028765218	0.730121664	0.834833063	no	down	mRNA	25.65	22.78	25.17	21.49	24.38	21.92	24.5333	22.5966667	COG2867	I:Lipid transport and metabolism	------	------
BAB_RS29030	-	enterobactin ABC transporter permease	0.954	-0.06817503	0.731387877	0.835686078	no	down	mRNA	3.77	4.96	4.23	3.78	4.07	3.93	4.32	3.92666667	COG4605;COG4604	P:Inorganic ion transport and metabolism;	GO:0016021;GO:0005886;GO:0005215	CC:integral component of membrane;CC:plasma membrane;MF:transporter activity;
BAB_RS19340	-	nucleoside-diphosphate kinase	0.986	-0.0196324	0.733307059	0.835798361	no	down	mRNA	126.93	118.3	125.41	119.75	114.1	110.73	123.547	114.86	COG0105	F:Nucleotide transport and metabolism	GO:0006241;GO:0006183;GO:0006228;GO:0006228;GO:0006241;GO:00	BP:CTP biosynthetic process;BP:GTP biosynthetic process;BP:UTP biosynthetic process;BP:UTP biosynthetic process;BP:CTP biosynthetic process;BP:GTP biosynthetic process;BP:UTP biosynthetic process;BP:CTP biosynthetic process;BP:GTP biosynthetic process;CC:cytoplasm;CC:cytoplasm;CC:cytoplasm;MF:ATP binding;MF:metal ion binding;MF:nucleoside diphosphate kinase activity;MF:nucleoside diphosphate kinase activity;MF:metal ion binding;MF:ATP binding;MF:nucleoside diphosphate kinase activity;MF:ATP binding;MF:metal ion binding;
BAB_RS22330	-	FAD-binding oxidoreductase	0.944	-0.082951554	0.73329684	0.836083303	no	down	mRNA	9.78	12.33	13.29	11.17	8.17	13.6	11.8	10.98	COG0665	K:Transcription	GO:0008168;GO:0016491	MF:methyltransferase activity;MF:oxidoreductase activity;
BAB_RS29480	-	transcription-repair coupling factor	0.983	-0.025299302	0.733022022	0.83636355	no	down	mRNA	16.19	14.7	16.35	15.79	13.69	14.13	15.7467	14.5366667	COG1197	L:Replication, recombination and repair	GO:0000716;GO:0006355;GO:0005737;GO:0016787;GO:0016740;GO:00	BP:transcription-coupled nucleotide-excision repair, DNA damage recognition;BP:regulation of transcription, DNA-templated;CC:cytoplasm;MF:hydrolase activity;MF:transferase activity;MF:damaged DNA binding;MF:ATP binding;
BAB_RS32170	-	hypothetical protein	0.884	-0.177764378	0.732465796	0.836620193	no	down	mRNA	1.4	3.93	6.91	4.53	2.8	4.44	4.08	3.92333333	------	------	------	------
BAB_RS23340	-	adenine phosphoribosyltransferase	0.982	-0.026006718	0.733005905	0.836642263	no	down	mRNA	33.84	30.83	38.1	29.23	33.41	32.33	34.2567	31.6566667	COG0503	I:Lipid transport and metabolism	GO:0044209;GO:0006166;GO:0006168;GO:0006168;GO:0044209;GO:00	BP:AMP salvage;BP:purine ribonucleoside salvage;BP:adenine salvage;BP:adenine salvage;BP:AMP salvage;BP:purine ribonucleoside salvage;BP:adenine salvage;BP:AMP salvage;BP:purine ribonucleoside salvage;CC:cytoplasm;CC:cytoplasm;CC:cytoplasm;MF:transferase activity, transferring acyl groups;MF:adenine phosphoribosyltransferase activity;MF:adenine phosphoribosyltransferase activity;MF:adenine phosphoribosyltransferase activity;
BAB_RS19495	-	hypothetical protein	0.959	-0.060088308	0.739091184	0.841197298	no	down	mRNA	10.64	7.57	7.56	7	7.9	8.06	8.59	7.65333333	ENOG4111UR8	S:Function unknown	------	------
BAB_RS16755	-	YraN family protein	0.933	-0.099617268	0.740363863	0.842347412	no	down	mRNA	16.25	8.82	19.21	11.28	13.3	15.92	14.76	13.5	COG0792	L:Replication, recombination and repair	GO:0004518;GO:0003676;GO:0004518;GO:0004519;GO:0003676	MF:nuclease activity;MF:nucleic acid binding;MF:nuclease activity;MF:endonuclease activity;MF:nucleic acid binding;
BAB_RS30795	-	DUF2249 domain-containing protein	0.955	-0.065947434	0.741418737	0.843248992	no	down	mRNA	85.26	115.1	71.18	64.01	98.76	84.69	90.5133	82.4866667	ENOG4111QPN	S:Function unknown	------	------
BAB_RS26965	-	DUF1217 domain-containing protein	0.926	-0.110841094	0.741837805	0.843427059	no	down	mRNA	3.39	1.47	2.18	1.93	1.82	2.18	2.34667	1.97666667	ENOG4111G6Q	S:Function unknown	------	------
BAB_RS31275	-	3-hydroxyacyl-CoA dehydrogenase	0.972	-0.040354776	0.743404301	0.844909098	no	down	mRNA	11.27	11.4	13.03	12.74	10.35	9.7	11.9	10.93	COG1250	C:Energy production and conversion	GO:0006631;GO:0006631;GO:0003857;GO:0003857	BP:fatty acid metabolic process;BP:fatty acid metabolic process;MF:3-hydroxyacyl-CoA dehydrogenase activity;MF:3-hydroxyacyl-CoA dehydrogenase activity;
BAB_RS23255	-	hypothetical protein	0.94	-0.089968966	0.743925623	0.845202626	no	down	mRNA	13.78	10.21	16.3	11.23	10.72	15.53	13.43	12.4933333	------	------	------	------
BAB_RS21450	-	class I SAM-dependent methyltransferase	0.982	-0.026844738	0.74501289	0.845540945	no	down	mRNA	30.6	25.47	28.75	26.56	25.15	26.59	28.2733	26.1	COG0500	D:Cell cycle control, cell division, chromosome par	GO:0008168	MF:methyltransferase activity;
BAB_RS30970	-	ribonuclease P protein component	0.971	-0.042272046	0.744930445	0.84574612	no	down	mRNA	93.33	82.38	78.44	89.82	65.83	80.5	84.7167	78.7166667	COG0594	J:Translation, ribosomal structure and biogenesis	GO:0001682;GO:0008033;GO:0004526;GO:0000049;GO:0004526;GO:00	BP:tRNA 5'-leader removal;BP:tRNA processing;MF:ribonuclease P activity;MF:tRNA binding;MF:ribonuclease P activity;MF:tRNA binding;
BAB_RS32460	-	hypothetical protein	0.969	-0.044883184	0.74610051	0.84647632	no	down	mRNA	11.75	12.32	15.7	11.83	11.58	13.4	13.2567	12.27	------	------	------	------
BAB_RS29775	-	sugar ABC transporter permease	0.973	-0.039116608	0.747440478	0.846800513	no	down	mRNA	22.53	17.13	17.33	17.89	17.88	16	18.9967	17.2566667	ENOG410XSXC	G:Carbohydrate transport and metabolism	GO:0055085;GO:0055085;GO:0016021;GO:0005886;GO:0016021;GO:00	BP:transmembrane transport;BP:transmembrane transport;CC:integral component of membrane;CC:plasma membrane;CC:integral component of membrane;CC:plasma membrane;
BAB_RS27475	-	VOC family virulence protein	0.983	-0.024486282	0.74673959	0.846902331	no	down	mRNA	278.05	284.16	277.75	244.13	283.54	269.24	279.987	265.636667	ENOG4111Q4U;COG0346	S:Function unknown;	GO:0018583;GO:0051213;GO:0018583;GO:0051213	MF:biphenyl-2,3-diol 1,2-dioxygenase activity;MF:dioxygenase activity;MF:biphenyl-2,3-diol 1,2-dioxygenase activity;MF:dioxygenase activity;
BAB_RS26315	-	alpha/beta hydrolase	0.987	-0.019048309	0.747402113	0.847055728	no	down	mRNA	44.7	43.58	50	42.5	43.81	42.17	46.0933	42.8266667	COG0596;ENOG410YNJD	C:Energy production and conversion;S:Function u	GO:0016787;GO:0004177;GO:0016740;GO:0016787;GO:0016740	MF:hydrolase activity;MF:aminopeptidase activity;MF:transferase activity;MF:hydrolase activity;MF:transferase activity;
BAB_RS30165	-	Crp/Fnr family transcriptional regulator	0.955	-0.066148003	0.749001821	0.847374244	no	down	mRNA	8.97	6.38	7.16	6.12	5.57	8.51	7.50333	6.73333333	COG0664	T:Signal transduction mechanisms	GO:0006355;GO:0006351;GO:0003677	BP:regulation of transcription, DNA-templated;BP:transcription, DNA-templated;MF:DNA binding;
BAB_RS26170	-	DNA starvation/stationary phase protection protein Dps	0.986	-0.020308705	0.748769041	0.847409274	no	down	mRNA	1975.84	1813.78	1905.73	1739.73	1727.4	1821.78	1898.45	1762.97	COG0783	P:Inorganic ion transport and metabolism	GO:0006879;GO:0006950;GO:0006879;GO:0006950;GO:0005623;GO:00	BP:cellular iron ion homeostasis;BP:response to stress;BP:cellular iron ion homeostasis;BP:response to stress;CC:cell;CC:cell;MF:oxidoreductase activity, oxidizing metal ions;MF:ferric iron binding;MF:DNA binding;MF:ferric iron binding;MF:oxidoreductase activity, oxidizing metal ions;
BAB_RS21330	-	HU family DNA-binding protein	0.984	-0.023010772	0.748351079	0.847533316	no	down	mRNA	877.22	812.6	916.28	723.79	892.71	805.35	868.7	807.283333	COG0776	L:Replication, recombination and repair	GO:0030261;GO:0030261;GO:0003677;GO:0003677	BP:chromosome condensation;BP:chromosome condensation;MF:DNA binding;MF:DNA binding;
BAB_RS22315	-	entericidin%2C EcnA/B family	0.986	-0.020085424	0.751014823	0.84845662	no	down	mRNA	202.25	189.49	225.19	177.67	205.98	188.97	205.643	190.873333	COG5510	S:Function unknown	GO:0009636;GO:0009636;GO:0009636;GO:0016021;GO:0016020;GO:00	BP:response to toxic substance;BP:response to toxic substance;BP:response to toxic substance;CC:integral component of membrane;CC:membrane;CC:membrane;
BAB_RS19115	-	endonuclease	0.987	-0.018545477	0.751773654	0.848717059	no	down	mRNA	36.15	35.31	41.31	35.46	34.89	34.33	37.59	34.8933333	COG3743	S:Function unknown	GO:0016021;GO:0005840;GO:0016021	CC:integral component of membrane;CC:ribosome;CC:integral component of membrane;
BAB_RS20870	-	diacylglycerol kinase	0.967	-0.04857476	0.7521593	0.848854174	no	down	mRNA	34.81	30.95	25.55	24.07	28.79	30.46	30.4367	27.7733333	COG0818	M:Cell wall/membrane/envelope biogenesis	GO:0006654;GO:0006654;GO:0016021;GO:0005886;GO:0016021;GO:00	BP:phosphatidic acid biosynthetic process;BP:phosphatidic acid biosynthetic process;CC:integral component of membrane;CC:plasma membrane;CC:integral component of membrane;CC:plasma membrane;MF:diacylglycerol kinase activity;MF:ATP binding;MF:ATP binding;MF:diacylglycerol kinase activity;
BAB_RS21675	-	VanZ family protein	0.969	-0.046051613	0.751676615	0.848905787	no	down	mRNA	37.54	34.93	44.97	36.49	33.98	39.9	39.1467	36.79	ENOG410XYU3	S:Function unknown	GO:0016021	CC:integral component of membrane;
BAB_RS17435	-	carbon monoxide dehydrogenase	0.973	-0.039023086	0.755123955	0.851900726	no	down	mRNA	37.06	34.22	30.59	27.83	32.91	33	33.9567	31.2466667	COG3427	S:Function unknown	------	------
BAB_RS25260	-	amino acid ABC transporter ATP-binding protein	0.901	-0.150534284	0.755930556	0.852511364	no	down	mRNA	0.72	2.81	1.15	1.41	1.55	1.15	1.56	1.37	COG1126	E:Amino acid transport and metabolism	GO:0015424;GO:0005524	MF:amino acid-transporting ATPase activity;MF:ATP binding;
BAB_RS24370	-	metalloprotease	0.987	-0.018576942	0.75795486	0.854494374	no	down	mRNA	57.63	58.82	61.86	54.65	56.9	54.45	59.4367	55.3333333	COG4784	O:Posttranslational modification, protein turnover,	GO:0004222	MF:metalloendopeptidase activity;
BAB_RS32005	-	hypothetical protein	0.895	-0.159513297	0.759336836	0.855152209	no	down	mRNA	9.12	2.13	4.38	3.12	5.7	3.37	5.21	4.06333333	------	------	------	------
BAB_RS32455	-	hypothetical protein	0.959	-0.059780207	0.761053713	0.856185428	no	down	mRNA	2.68	1.98	3.57	2.22	2.82	2.36	2.74333	2.46666667	------	------	------	------
BAB_RS28810	-	ArsR family transcriptional regulator	0.962	-0.055624794	0.761976412	0.85692342	no	down	mRNA	11.55	12.26	9.55	8.1	10.85	11.34	11.12	10.0966667	COG1522	E:Amino acid transport and metabolism	GO:0006351;GO:0005622;GO:0003700;GO:0043565	BP:transcription, DNA-templated;CC:intracellular;MF:transcription factor activity, sequence-specific DNA binding;MF:sequence-specific DNA binding;
BAB_RS18565	-	glycosyltransferase family 1 protein	0.985	-0.021761658	0.762639946	0.857069656	no	down	mRNA	136.7	113.9	117.8	114.21	112.99	114.57	122.8	113.923333	COG0438	L:Replication, recombination and repair	GO:0016757;GO:0016740;GO:0016757	MF:transferase activity, transferring glycosyl groups;MF:transferase activity;MF:transferase activity, transferring glycosyl groups;
BAB_RS21415	-	lipoyl synthase	0.985	-0.021699464	0.7640249	0.858325875	no	down	mRNA	256.64	204.68	225.87	217.09	216.79	203.88	229.063	212.586667	COG0320	H:Coenzyme transport and metabolism	GO:0009249;GO:0009249;GO:0005737;GO:0005737;GO:0051539;GO:00	BP:protein lipoylation;BP:protein lipoylation;CC:cytoplasm;CC:cytoplasm;MF:4 iron, 4 sulfur cluster binding;MF:lipoate synthase activity;MF:metal ion binding;MF:metal ion binding;MF:4 iron, 4 sulfur cluster binding;MF:lipoate synthase activity;
BAB_RS17780	-	nitrogen fixation protein FixH	0.988	-0.017512314	0.766123994	0.859481981	no	down	mRNA	555.36	554.12	604.11	538.32	527.92	550.75	571.197	538.996667	COG5456	P:Inorganic ion transport and metabolism	GO:0016021	CC:integral component of membrane;
BAB_RS16980	-	ABC transporter substrate-binding protein	0.987	-0.01816853	0.768434982	0.861472993	no	down	mRNA	218.97	187.54	205.89	197.33	182.97	190.17	204.133	190.156667	COG1732	E:Amino acid transport and metabolism	GO:0043190;GO:0043190;GO:0022857;GO:0022857	CC:ATP-binding cassett;CC:ATP-binding cassett;MF:transmembrane transporter activity;MF:transmembrane transporter activity;
BAB_RS29635	-	DUF995 domain-containing protein	0.977	-0.034028994	0.769195497	0.861724245	no	down	mRNA	16.91	18.62	21.38	19.13	17.05	16.67	18.97	17.6166667	ENOG410YP7G;ENOG4112A49	S:Function unknown;	GO:0016021	CC:integral component of membrane;
BAB_RS28935	-	hypothetical protein	0.98	-0.029388175	0.770112387	0.862150208	no	down	mRNA	32.32	32.27	37.54	32.27	34.18	28.23	34.0433	31.56	------	------	GO:0016021	CC:integral component of membrane;
BAB_RS32790	-	hypothetical protein	0.954	-0.067989569	0.771991171	0.863351073	no	down	mRNA	9.47	5.81	9.9	9.36	7.12	6.09	8.39333	7.52333333	------	------	------	------
BAB_RS30720	-	RES domain-containing protein	0.979	-0.030545399	0.771880718	0.863528115	no	down	mRNA	37.76	31.06	33.35	33.64	27.99	32.65	34.0567	31.4266667	ENOG4111IRQ	S:Function unknown	------	------
BAB_RS29905	-	hypothetical protein	0.972	-0.04090913	0.772633558	0.863768832	no	down	mRNA	22.5	17.65	26.4	19.09	21.83	20.35	22.1833	20.4233333	COG3757	H:Coenzyme transport and metabolism	------	------
BAB_RS31530	-	flagellar type III secretion system protein FliR	0.898	-0.15584455	0.774285917	0.864413013	no	down	mRNA	0.78	0.56	1.63	1.18	0.5	1.01	0.99	0.89666667	COG1684	N:Cell motility	GO:0044780;GO:0006605;GO:0006605;GO:0044780;GO:0044780;GO:00	BP:bacterial-type flagellum assembly;BP:protein targeting;BP:protein targeting;BP:bacterial-type flagellum assembly;BP:bacterial-type flagellum assembly;BP:protein targeting;CC:integral component of membrane;CC:bacterial-type flagellum basal body;CC:intracellular;CC:plasma membrane;CC:integral component of membrane;CC:bacterial-type flagellum basal body;CC:plasma membrane;CC:intracellular;CC:bacterial-type flagellum basal body;CC:integral component of membrane;CC:intracellular;CC:plasma membrane;
BAB_RS30250	-	shikimate dehydrogenase	0.973	-0.039224612	0.774622325	0.864488201	no	down	mRNA	17.53	17.26	16.53	17.04	16.89	13.12	17.1067	15.6833333	COG0169	S:Function unknown	GO:0004764	MF:shikimate 3-dehydrogenas;
BAB_RS21630	-	ATP-dependent Clp protease adaptor ClpS	0.985	-0.022036865	0.775738062	0.865132382	no	down	mRNA	956.26	743.78	735.96	763.87	780.96	748.6	812	764.476667	COG2127	O:Posttranslational modification, protein turnover,	GO:0030163;GO:0030163;GO:0008233	BP:protein catabolic process;BP:protein catabolic process;MF:peptidase activity;
BAB_RS16910	-	ABC transporter permease	0.961	-0.057037555	0.777243995	0.866511089	no	down	mRNA	5	5.06	6.3	5.23	4.31	5.57	5.45333	5.03666667	COG0600	P:Inorganic ion transport and metabolism	GO:0055085;GO:0055085;GO:0016021;GO:0005886;GO:0016021;GO:00	BP:transmembrane transport;BP:transmembrane transport;CC:integral component of membrane;CC:plasma membrane;CC:integral component of membrane;CC:plasma membrane;
BAB_RS30055	-	DUF808 domain-containing protein	0.98	-0.029583917	0.778248519	0.867029297	no	down	mRNA	18.5	15.31	16.64	16.01	14.51	15.94	16.8167	15.4866667	COG2354	S:Function unknown	GO:0016021	CC:integral component of membrane;
BAB_RS27805	-	sugar ABC transporter permease	0.986	-0.020367371	0.779295465	0.867293498	no	down	mRNA	26.97	23.54	29.09	22.7	25.38	25.6	26.5333	24.56	COG4214	G:Carbohydrate transport and metabolism	GO:0008643;GO:0005886;GO:0016021;GO:0016021;GO:0005886;GO:00	BP:carbohydrate transport;CC:plasma membrane;CC:integral component of membrane;CC:integral component of membrane;CC:plasma membrane;MF:transmembrane transporter activity;MF:transmembrane transporter activity;
BAB_RS18090	-	3%2C4-dihydroxy-2-butanone-4-phosphate synthase	0.983	-0.024246109	0.781682015	0.868445475	no	down	mRNA	87.96	90.72	82.61	75.58	89.13	77.87	87.0967	80.86	COG0108	H:Coenzyme transport and metabolism	GO:0009231;GO:0016787;GO:0000287;GO:0030145;GO:0008686	BP:riboflavin biosynthetic process;MF:hydrolase activity;MF:magnesium ion binding;MF:manganese ion binding;MF:3,4-dihydroxy-2-butanone-4-phosphate synthase activity;
BAB_RS19515	-	hypothetical protein	0.981	-0.027707641	0.781647336	0.868707329	no	down	mRNA	32.9	25.09	34.75	26.78	30.38	28.17	30.9133	28.4433333	------	------	------	------
BAB_RS24250	-	hypothetical protein	0.958	-0.062630466	0.781147362	0.868752674	no	down	mRNA	251.99	202.52	295.4	247.49	253.21	253.84	249.97	251.513333	------	------	GO:0016021	CC:integral component of membrane;
BAB_RS26860	-	gfo/Idh/MocA family oxidoreductase	0.983	-0.025058481	0.782841609	0.868832501	no	down	mRNA	29.46	28.13	31.24	25.21	25.91	31.3	29.61	27.4733333	ENOG410XQKZ	S:Function unknown	GO:0016491	MF:oxidoreductase activity;
BAB_RS24335	-	DUF2312 domain-containing protein	0.989	-0.015300451	0.781516401	0.868862351	no	down	mRNA	764.38	643.73	720.47	646.03	678.45	669.99	709.527	664.823333	COG3750	S:Function unknown	------	------
BAB_RS31860	livF	ABC transporter ATP-binding protein	0.984	-0.023587268	0.78473042	0.869127486	no	down	mRNA	27.06	28.78	31.82	28.23	26.18	27.35	29.22	27.2533333	COG0410	E:Amino acid transport and metabolism	GO:0005524;GO:0016887;GO:0016301;GO:0016887;GO:0005524	MF:ATP binding;MF:ATPase activity;MF:kinase activity;MF:ATPase activity;MF:ATP binding;
BAB_RS21490	-	anthranilate phosphoribosyltransferase	0.986	-0.021070316	0.786426471	0.870405874	no	down	mRNA	83.55	84.93	104.95	87.07	83.36	84.25	91.1433	84.8933333	COG0547	E:Amino acid transport and metabolism	GO:0000162;GO:0000162;GO:0000162;GO:0004048;GO:0046872;GO:00	BP:tryptophan biosynthetic process;BP:tryptophan biosynthetic process;BP:tryptophan biosynthetic process;MF:anthranilate phosphoribosyltransferase activity;MF:metal ion binding;MF:metal ion binding;MF:anthranilate phosphoribosyltransferase activity;MF:magnesium ion binding;MF:metal ion binding;MF:anthranilate phosphoribosyltransferase activity;MF:magnesium ion binding;
BAB_RS32260	-	IS481 family transposase	0.983	-0.024484465	0.787379605	0.870860816	no	down	mRNA	41.31	36.55	38.92	35.25	33.18	39.98	38.9267	36.1366667	ENOG410XP5C	L:Replication, recombination and repair	GO:0015074;GO:0003677	BP:DNA integration;MF:DNA binding;
BAB_RS21285	-	phosphopantetheine adenylyltransferase	0.985	-0.021899739	0.789017049	0.870873163	no	down	mRNA	62.45	64.78	78.75	62.5	67.03	62.32	68.66	63.95	COG0669	H:Coenzyme transport and metabolism	GO:0015937;GO:0015937;GO:0015937;GO:0005737;GO:0005737;GO:00	BP:coenzyme A biosynthetic process;BP:coenzyme A biosynthetic process;BP:coenzyme A biosynthetic process;CC:cytoplasm;CC:cytoplasm;CC:cytoplasm;MF:pantetheine-phosphate adenylyltransferase activity;MF:ATP binding;MF:ATP binding;MF:pantetheine-phosphate adenylyltransferase activity;MF:pantetheine-phosphate adenylyltransferase activity;MF:ATP binding;MF:oxidoreductase activity;
BAB_RS32250	-	hypothetical protein	0.912	-0.133042793	0.788570962	0.870979202	no	down	mRNA	12.45	8.5	7.05	5.47	8.39	10.55	9.33333	8.13666667	COG1194	L:Replication, recombination and repair	------	------
BAB_RS20185	-	exodeoxyribonuclease III	0.987	-0.018149949	0.788853296	0.870991629	no	down	mRNA	68.05	56.91	62.25	55.55	59.09	59.1	62.4033	57.9133333	COG0708	L:Replication, recombination and repair	GO:0006281;GO:0006281;GO:0005622;GO:0005622;GO:0003677;GO:00	BP:DNA repair;BP:DNA repair;CC:intracellular;CC:intracellular;MF:DNA binding;MF:endonuclease activity;MF:exodeoxyribonuclease III activity;MF:endonuclease activity;MF:exodeoxyribonuclease III activity;MF:DNA binding;
BAB_RS25050	-	antibiotic biosynthesis monooxygenase	0.955	-0.06576294	0.787825473	0.871054111	no	down	mRNA	14.39	16.01	22.09	17.31	16.88	16.05	17.4967	16.7466667	COG2329	S:Function unknown	GO:0004497	MF:monooxygenase activity;
BAB_RS28590	-	Lrp/AsnC family transcriptional regulator	0.979	-0.030831807	0.788426224	0.871118795	no	down	mRNA	98.34	90.54	98.01	74.96	87.35	105.87	95.63	89.3933333	COG1522	E:Amino acid transport and metabolism	GO:0006351;GO:0005622;GO:0003700;GO:0043565	BP:transcription, DNA-templated;CC:intracellular;MF:transcription factor activity, sequence-specific DNA binding;MF:sequence-specific DNA binding;
BAB_RS22885	-	divalent metal cation transporter	0.975	-0.036572572	0.787373298	0.871153722	no	down	mRNA	45.42	38.64	43.01	31.18	48.27	36.71	42.3567	38.72	COG1914	P:Inorganic ion transport and metabolism	GO:0016021;GO:0005886;GO:0016021;GO:0005886;GO:0046873;GO:00	CC:integral component of membrane;CC:plasma membrane;CC:integral component of membrane;CC:plasma membrane;MF:metal ion transmembrane transporter activity;MF:metal ion binding;MF:symporter activity;MF:metal ion binding;MF:metal ion transmembrane transporter activity;MF:symporter activity;
BAB_RS30585	-	nitrate reductase subunit beta	0.989	-0.016097241	0.790797647	0.871341852	no	down	mRNA	502.83	508.6	529.88	477.21	452.26	507.98	513.77	479.15	COG1140	C:Energy production and conversion	GO:0042126;GO:0042126;GO:0042126;GO:0009325;GO:0009325;GO:00	BP:nitrate metabolic process;BP:nitrate metabolic process;BP:nitrate metabolic process;CC:nitrate reductase complex;CC:nitrate reductase complex;CC:nitrate reductase complex;MF:nitrate reductase activity;MF:nitrate reductase activity;MF:nitrate reductase activity;MF:mannose-1-phosphate guanylyltransferase activity;
BAB_RS21910	-	50S ribosomal protein L23	0.982	-0.025487455	0.791088747	0.871363779	no	down	mRNA	1269.67	1148.98	1363.61	1193.98	1242.53	1226.13	1260.75	1220.88	COG0089	J:Translation, ribosomal structure and biogenesis	GO:0006412;GO:0006412;GO:0005840;GO:0005840;GO:0003735;GO:00	BP:translation;BP:translation;CC:ribosome;CC:ribosome;MF:structural constituent of ribosome;MF:rRNA binding;MF:structural constituent of ribosome;MF:rRNA binding;
BAB_RS22990	-	bifunctional nicotinamidase/pyrazinamidase	0.987	-0.018152064	0.790133202	0.871506343	no	down	mRNA	60.37	58.92	61.07	54.56	57.24	56.77	60.12	56.19	COG1335	Q:Secondary metabolites biosynthesis, transport a	GO:0008152;GO:0008152;GO:0008152;GO:0003824;GO:0016787;GO:00	BP:metabolic process;BP:metabolic process;BP:metabolic process;MF:catalytic activity;MF:hydrolase activity;MF:catalytic activity;MF:nicotinamidase activity;
BAB_RS26550	-	hypothetical protein	0.934	-0.098348524	0.789881156	0.871527525	no	down	mRNA	27.18	42.7	46.39	42.99	32.15	45.06	38.7567	40.0666667	------	------	GO:0016021	CC:integral component of membrane;
BAB_RS28405	-	sugar ABC transporter permease	0.975	-0.036434188	0.793420703	0.87333358	no	down	mRNA	13.29	11.69	12.03	12.69	11.33	9.87	12.3367	11.2966667	COG1175	P:Inorganic ion transport and metabolism	GO:0055085;GO:0055085;GO:0016021;GO:0005886;GO:0016021;GO:00	BP:transmembrane transport;BP:transmembrane transport;CC:integral component of membrane;CC:plasma membrane;CC:integral component of membrane;CC:plasma membrane;
BAB_RS20285	-	membrane protein	0.979	-0.030805467	0.794740739	0.874486984	no	down	mRNA	72.59	67.91	59.15	62.95	58.01	66.55	66.55	62.5033333	COG3737	S:Function unknown	------	------
BAB_RS32770	-	hypothetical protein	0.983	-0.025387727	0.797450682	0.875968903	no	down	mRNA	241.62	221.76	316.08	221.92	263.15	234.18	259.82	239.75	------	------	------	------
BAB_RS18865	-	hypothetical protein	0.927	-0.10903067	0.796634705	0.875971016	no	down	mRNA	1.58	1.8	1.59	1.33	1.45	1.62	1.65667	1.46666667	ENOG4111USG	S:Function unknown	------	------
BAB_RS22570	-	hypothetical protein	0.989	-0.015723855	0.798343625	0.876350553	no	down	mRNA	171.79	148.95	160.74	146.45	143.73	158	160.493	149.393333	ENOG4111M81	S:Function unknown	GO:0016021	CC:integral component of membrane;
BAB_RS33185	-	hypothetical protein	0.911	-0.133797026	0.798878509	0.8766382	no	down	mRNA	6.44	26.21	15.97	8.69	21.51	17.28	16.2067	15.8266667	------	------	GO:0005524	MF:ATP binding;
BAB_RS20845	-	phosphoglycolate phosphatase	0.986	-0.020837018	0.800279615	0.877576247	no	down	mRNA	118.28	98.39	102.53	91.22	99.89	105.97	106.4	99.0266667	COG0546	G:Carbohydrate transport and metabolism	GO:0005975;GO:0046295;GO:0046295;GO:0005975;GO:0046872;GO:00	BP:carbohydrate metabolic process;BP:glycolate biosynthetic process;BP:glycolate biosynthetic process;BP:carbohydrate metabolic process;MF:metal ion binding;MF:phosphoglycolate phosphatase activity;MF:ligase activity;MF:metal ion binding;MF:phosphoglycolate phosphatase activity;
BAB_RS19745	-	quaternary ammonium compound-resistance protein SugE	0.961	-0.058103208	0.800020016	0.877591093	no	down	mRNA	21.28	23.48	15.32	20.08	15.58	19.75	20.0267	18.47	COG2076	P:Inorganic ion transport and metabolism	GO:0016021;GO:0005886	CC:integral component of membrane;CC:plasma membrane;
BAB_RS28290	-	-	0.962	-0.056130135	0.802084942	0.878955975	no	down	mRNA	561.77	432.27	463.69	585.37	506.17	311.13	485.91	467.556667	------	------	------	------
BAB_RS30310	-	4Fe-4S binding domain-containing protein	0.988	-0.017572887	0.803501867	0.880208489	no	down	mRNA	104.67	113.62	115.19	98.12	110.68	102.2	111.16	103.666667	COG0348	G:Carbohydrate transport and metabolism	GO:0016021	CC:integral component of membrane;
BAB_RS18205	-	DUF2232 domain-containing protein	0.984	-0.023862385	0.804067419	0.880527817	no	down	mRNA	33.44	30.63	34.26	33.82	30.66	26.61	32.7767	30.3633333	ENOG4111KED;ENOG4110225	S:Function unknown;	GO:0016021	CC:integral component of membrane;
BAB_RS25580	-	HlyC/CorC family transporter	0.984	-0.023859705	0.805052292	0.881005795	no	down	mRNA	19.51	19.22	24.24	18.52	21.04	18.89	20.99	19.4833333	COG4536	P:Inorganic ion transport and metabolism	GO:0016021;GO:0050660;GO:0016614	CC:integral component of membrane;MF:flavin adenine dinucleotide binding;MF:oxidoreductase activity, acting on CH-OH group of donors;
BAB_RS18640	-	gfo/Idh/MocA family oxidoreductase	0.989	-0.016567746	0.804959199	0.881204057	no	down	mRNA	44.12	44.53	45.67	39.77	43.68	41.78	44.7733	41.7433333	COG0673	G:Carbohydrate transport and metabolism	GO:0016491	MF:oxidoreductase activity;
BAB_RS25065	-	tRNA (guanosine(37)-N1)-methyltransferase TrmD	0.985	-0.022113817	0.806503869	0.881693409	no	down	mRNA	35.52	38.81	39.71	37.66	34.87	34.05	38.0133	35.5266667	COG0336	J:Translation, ribosomal structure and biogenesis	GO:0005737;GO:0005737;GO:0052906;GO:0016787;GO:0052906	CC:cytoplasm;CC:cytoplasm;MF:tRN;MF:hydrolase activity;MF:tRN;
BAB_RS29885	-	8-amino-7-oxononanoate synthase	0.984	-0.023267745	0.806239972	0.881704912	no	down	mRNA	50.35	53.98	52.41	53.26	49.7	42.79	52.2467	48.5833333	COG0156	H:Coenzyme transport and metabolism	GO:0009058;GO:0009058;GO:0008710;GO:0003824;GO:0030170;GO:00	BP:biosynthetic process;BP:biosynthetic process;MF:8-amino-7-oxononanoate synthase activity;MF:catalytic activity;MF:pyridoxal phosphate binding;MF:transaminase activity;MF:catalytic activity;MF:pyridoxal phosphate binding;
BAB_RS21220	-	hypothetical protein	0.891	-0.167208954	0.807752599	0.882458041	no	down	mRNA	0.35	0.75	1.53	0.42	1.07	0.9	0.87667	0.79666667	COG3617	K:Transcription	------	------
BAB_RS29760	-	ABC transporter ATP-binding protein	0.982	-0.026458107	0.808160158	0.882603191	no	down	mRNA	15.13	12.72	14.58	12.18	13.52	13.45	14.1433	13.05	COG3839;COG3842	C:Energy production and conversion;	GO:0043190;GO:0043190;GO:0043190;GO:0005524;GO:0016887;GO:00	CC:ATP-binding cassett;CC:ATP-binding cassett;CC:ATP-binding cassett;MF:ATP binding;MF:ATPase activity;MF:transmembrane transporter activity;MF:transmembrane transporter activity;MF:ATP binding;MF:ATPase activity;MF:transmembrane transporter activity;MF:ATPase activity;MF:ATP binding;
BAB_RS20370	-	peptidase	0.988	-0.016833836	0.808497979	0.882672106	no	down	mRNA	63.45	64.95	62.06	60.58	57.84	59.3	63.4867	59.24	COG4783	O:Posttranslational modification, protein turnover,	GO:0004222	MF:metalloendopeptidase activity;
BAB_RS28360	-	efflux RND transporter periplasmic adaptor subunit	0.953	-0.069125604	0.812888516	0.885960245	no	down	mRNA	2.96	3.01	3.79	2.05	3.77	3	3.25333	2.94	ENOG410XSF7	V:Defense mechanisms	------	------
BAB_RS26035	-	50S ribosomal protein L35	0.986	-0.020223197	0.815011588	0.88737114	no	down	mRNA	600.36	471.03	480.92	532.19	459.11	478.08	517.437	489.793333	COG0291	J:Translation, ribosomal structure and biogenesis	GO:0006412;GO:0005840;GO:0003735	BP:translation;CC:ribosome;MF:structural constituent of ribosome;
BAB_RS24740	-	N-acetyltransferase	0.976	-0.034732694	0.815870182	0.888005046	no	down	mRNA	23.45	21.95	21.91	24.89	18.29	19.18	22.4367	20.7866667	COG0454;ENOG4111U3U	K:Transcription;S:Function unknown	GO:0016740	MF:transferase activity;
BAB_RS22995	-	DUF475 domain-containing protein	0.984	-0.023444363	0.816618916	0.88851899	no	down	mRNA	181.07	142.16	144.27	153.96	149.67	128.9	155.833	144.176667	COG2899	S:Function unknown	GO:0016021	CC:integral component of membrane;
BAB_RS17670	-	saccharopine dehydrogenase	0.987	-0.019361493	0.817710774	0.888804032	no	down	mRNA	138.77	111.58	113.96	115.1	115.21	107.84	121.437	112.716667	COG1748	E:Amino acid transport and metabolism	GO:0016491	MF:oxidoreductase activity;
BAB_RS19695	-	6%2C7-dimethyl-8-ribityllumazine synthase	0.987	-0.018195754	0.817478797	0.88885258	no	down	mRNA	168.74	179.59	187.89	180.98	161.41	162.88	178.74	168.423333	COG0054	H:Coenzyme transport and metabolism	GO:0009231;GO:0009231;GO:0009231;GO:0009349;GO:0009349;GO:00	BP:riboflavin biosynthetic process;BP:riboflavin biosynthetic process;BP:riboflavin biosynthetic process;CC:riboflavin synthase complex;CC:riboflavin synthase complex;CC:riboflavin synthase complex;MF:6,7-dimethyl-8-ribityllumazine synthase activity;MF:6,7-dimethyl-8-ribityllumazine synthase activity;MF:lyase activity;MF:6,7-dimethyl-8-ribityllumazine synthase activity;MF:lyase activity;MF:lyase activity;
BAB_RS27105	-	flagellar basal body protein FliL	0.899	-0.153757452	0.819111968	0.889725069	no	down	mRNA	0.41	1.16	1.26	0.74	0.62	1.32	0.94333	0.89333333	ENOG411242A	N:Cell motility	GO:0071973;GO:0006935;GO:0006935;GO:0071973;GO:0071973;GO:00	BP:bacterial-type flagellum-dependent cell motility;BP:chemotaxis;BP:chemotaxis;BP:bacterial-type flagellum-dependent cell motility;BP:bacterial-type flagellum-dependent cell motility;BP:chemotaxis;CC:bacterial-type flagellum basal body;CC:plasma membrane;CC:bacterial-type flagellum basal body;CC:plasma membrane;CC:bacterial-type flagellum basal body;CC:integral component of membrane;CC:plasma membrane;
BAB_RS24695	-	membrane protein	0.989	-0.016418398	0.820004165	0.890092359	no	down	mRNA	80.88	81.29	100.15	80.16	80.34	84.03	87.44	81.51	COG4942	D:Cell cycle control, cell division, chromosome par	------	------
BAB_RS32785	-	hypothetical protein	0.981	-0.02725367	0.819776746	0.890146227	no	down	mRNA	18.65	19.05	26.32	19.69	19.42	20.54	21.34	19.8833333	COG1496	S:Function unknown	------	------
BAB_RS30190	-	peptide ABC transporter substrate-binding protein	0.988	-0.018144497	0.82232628	0.892010242	no	down	mRNA	31.82	26.35	30.59	25.19	28.01	29.11	29.5867	27.4366667	COG0747	K:Transcription	GO:0015031;GO:0055085;GO:0055085;GO:0043190;GO:0042597;GO:00	BP:protein transport;BP:transmembrane transport;BP:transmembrane transport;CC:ATP-binding cassett;CC:periplasmic space;CC:ATP-binding cassett;
BAB_RS33225	-	AcrB/AcrD/AcrF family protein	0.961	-0.056843853	0.822218623	0.892194676	no	down	mRNA	2.34	3.09	2.59	2.43	2.64	2.26	2.67333	2.44333333	COG0841	V:Defense mechanisms	GO:0016021;GO:0022857	CC:integral component of membrane;MF:transmembrane transporter activity;
BAB_RS28025	-	6-O-methylguanine DNA methyltransferase	0.975	-0.036733313	0.822954608	0.892390536	no	down	mRNA	6.07	7.73	8.21	5.93	6.76	7.83	7.33667	6.84	COG0350	E:Amino acid transport and metabolism	GO:0006281;GO:0006351;GO:0006281;GO:0006351;GO:0006281;GO:00	BP:DNA repair;BP:transcription, DNA-templated;BP:DNA repair;BP:transcription, DNA-templated;BP:DNA repair;BP:transcription, DNA-templated;MF:sequence-specific DNA binding;MF:3-alpha-hydroxy-5-beta-androstane-17-one 3-alpha-dehydrogenase activity;MF:transcription factor activity, sequence-specific DNA binding;MF:zinc ion binding;MF:methylated-DNA-[protein]-cysteine S-methyltransferase activity;MF:sequence-specific DNA binding;MF:3-alpha-hydroxy-5-beta-androstane-17-one 3-alpha-dehydrogenase activity;MF:transcription factor activity, sequence-specific DNA binding;MF:methylated-DNA-[protein]-cysteine S-methyltransferase activity;MF:zinc ion binding;MF:transcription factor activity, sequence-specific DNA binding;MF:methylated-DNA-[protein]-cysteine S-methyltransferase activity;MF:sequence-specific DNA binding;MF:zinc ion binding;
BAB_RS28160	-	D-ribose ABC transporter substrate-binding protein	0.991	-0.013268542	0.824457987	0.893116491	no	down	mRNA	638.72	554.2	595.67	528.14	558.74	586.44	596.197	557.773333	COG1879	S:Function unknown	------	------
BAB_RS28030	-	MFS transporter	0.986	-0.020446518	0.825164009	0.893278962	no	down	mRNA	26.35	24.19	23.76	20.23	24.54	24.09	24.7667	22.9533333	ENOG410XP7I	E:Amino acid transport and metabolism	GO:0008643;GO:0016021;GO:0016021;GO:0022857;GO:0016301;GO:00	BP:carbohydrate transport;CC:integral component of membrane;CC:integral component of membrane;MF:transmembrane transporter activity;MF:kinase activity;MF:transmembrane transporter activity;
BAB_RS20840	-	hypothetical protein	0.979	-0.030361319	0.825799781	0.893666115	no	down	mRNA	25.58	31.41	36.19	29.74	27.59	30.63	31.06	29.32	------	------	------	------
BAB_RS23530	-	ABC transporter ATP-binding protein	0.984	-0.02395676	0.827573488	0.894380631	no	down	mRNA	23.55	18.31	23.82	20.83	20.36	19.58	21.8933	20.2566667	COG0444;COG0444	E:Amino acid transport and metabolism;P:Inorgani	GO:0015833;GO:0016887;GO:0005524	BP:peptide transport;MF:ATPase activity;MF:ATP binding;

BAB_RS25010	-	succinate dehydrogenase%2C hydrophobic membrane anchor protein	0.986	-0.020230597	0.827423211	0.894519104	no	down	mRNA	288.07	214.19	225.54	239.87	221.11	222.88	242.6	227.953333	COG2142	C:Energy production and conversion	GO:0006099;GO:0016021;GO:0000104;GO:0020037	BP:tricarboxylic acid cycle;CC:integral component of membrane;MF:succinate dehydrogenase activity;MF:heme binding;
BAB_RS27755	-	FadR family transcriptional regulator	0.991	-0.013148578	0.826908584	0.894564741	no	down	mRNA	87.1	85.44	104.55	84.2	87.26	86.58	92.3633	86.0133333	COG2186	E:Amino acid transport and metabolism	GO:0006351;GO:0003700;GO:0003677	BP:transcription, DNA-templated;MF:transcription factor activity, sequence-specific DNA binding;MF:DNA binding;
BAB_RS22395	-	hypothetical protein	0.971	-0.04229555	0.829068053	0.895694571	no	down	mRNA	272.96	347.81	453.74	320.36	360.99	379.62	358.17	353.656667	------	------	------	------
BAB_RS32025	-	hypothetical protein	0.987	-0.018940677	0.831514796	0.897432663	no	down	mRNA	51.25	45.33	62.16	47.33	53.45	46.27	52.9133	49.0166667	------	------	------	------
BAB_RS29575	-	YbaK/EbsC family protein	0.987	-0.018658361	0.831840611	0.897482835	no	down	mRNA	173.21	160.48	157.98	132.14	163.63	162.89	163.89	152.886667	COG2606	S:Function unknown	GO:0004812;GO:0002161;GO:0002161	MF:aminoacyl-tRNA ligase activity;MF:aminoacyl-tRNA editing activity;MF:aminoacyl-tRNA editing activity;
BAB_RS27085	-	flagellar basal body P-ring protein FlgI	0.982	-0.026713354	0.835341561	0.90065518	no	down	mRNA	10.37	10.73	11.52	10.17	9.29	10.99	10.8733	10.15	COG1706	N:Cell motility	GO:0071973;GO:0009428;GO:0030288;GO:0005198	BP:bacterial-type flagellum-dependent cell motility;CC:bacterial-type flagellum basal body, distal rod, P ring;CC:outer membrane-bounded periplasmic space;MF:structural molecule activity;
BAB_RS24245	prpE	propionyl-CoA synthetase	0.99	-0.013983438	0.836970909	0.901504369	no	down	mRNA	68.84	63.38	70.53	57.79	68.51	62.53	67.5833	62.9433333	COG0365	I:Lipid transport and metabolism	GO:0008152;GO:0008152;GO:0050218;GO:0016874;GO:0016874	BP:metabolic process;BP:metabolic process;MF:propionate-CoA ligase activity;MF:ligase activity;MF:ligase activity;
BAB_RS27890	-	hypothetical protein	0.974	-0.038570108	0.837686845	0.901973135	no	down	mRNA	96.86	96.64	113.9	81.81	108.9	113.04	102.467	101.25	ENOG410Y2WI	S:Function unknown	------	------
BAB_RS31690	-	homoprotocatechuate degradation operon regulator HpaR	0.988	-0.017644229	0.838632602	0.902386655	no	down	mRNA	71.56	74.86	81.85	76.7	72.63	64.57	76.09	71.3	COG1846	Q:Secondary metabolites biosynthesis, transport a	GO:0045892;GO:0010124;GO:0006351;GO:0003700;GO:0003677	BP:negative regulation of transcription, DNA-templated;BP:phenylacetate catabolic process;BP:transcription, DNA-templated;MF:transcription factor activity, sequence-specific DNA binding;MF:DNA binding;
BAB_RS27215	-	hypothetical protein	0.988	-0.017701262	0.839816713	0.903055924	no	down	mRNA	196.42	186.77	189.78	168.43	161.38	205.16	190.99	178.323333	COG1376	S:Function unknown	GO:0016021;GO:0016740;GO:0016740	CC:integral component of membrane;MF:transferase activity;MF:transferase activity;
BAB_RS17015	-	sarcosine oxidase subunit alpha	0.971	-0.041942572	0.841196019	0.90423647	no	down	mRNA	4.81	4.22	3.4	3.54	4.49	3.11	4.14333	3.71333333	COG0446	S:Function unknown	GO:0046653;GO:0016740;GO:0008115	BP:tetrahydrofolate metabolic process;MF:transferase activity;MF:sarcosine oxidase activity;
BAB_RS24075	-	tol-pal system protein YbgF	0.993	-0.009997334	0.844244006	0.906906049	no	down	mRNA	156.98	143.45	152.37	142.82	140.21	140.34	150.933	141.123333	COG1729	S:Function unknown	GO:0043093;GO:0030288	BP:FtsZ-dependent cytokinesis;CC:outer membrane-bounded periplasmic space;
BAB_RS20535	-	transporter	0.992	-0.011002109	0.845087174	0.907205175	no	down	mRNA	357.94	348.3	359.64	340.21	342.05	315.07	355.293	332.443333	COG1538	M:Cell wall/membrane/envelope biogenesis	GO:0046677;GO:0016021;GO:0009279;GO:0019867;GO:0019867;GO:00	BP:response to antibiotic;CC:integral component of membrane;CC:cell outer membrane;CC:outer membrane;CC:outer membrane;MF:efflux transmembrane transporter activity;MF:efflux transmembrane transporter activity;
BAB_RS19720	-	DUF177 domain-containing protein	0.988	-0.01691813	0.844845348	0.907248698	no	down	mRNA	75.8	64.32	64.23	66.23	61.06	63.4	68.1167	63.5633333	ENOG41126CC	S:Function unknown	------	------
BAB_RS29145	-	branched-chain amino acid ABC transporter permease	0.981	-0.027510084	0.84804348	0.907649467	no	down	mRNA	11.75	11.82	10.77	9.48	10.1	12.3	11.4467	10.6266667	COG4177	F:Nucleotide transport and metabolism	------	------
BAB_RS24510	-	ATP synthase epsilon chain	0.993	-0.010303939	0.848371176	0.907697831	no	down	mRNA	368.07	345.56	362.05	343.91	340.98	330.44	358.56	338.443333	COG0355	C:Energy production and conversion	GO:0015986;GO:0015986;GO:0015986;GO:0015986;GO:0005886;GO:00	BP:ATP synthesis coupled proton transport;BP:ATP synthesis coupled proton transport;BP:ATP synthesis coupled proton transport;BP:ATP synthesis coupled proton transport;CC:plasma membrane;CC:proton-transporting ATP synthase complex, catalytic core;CC:plasma membrane;CC:proton-transporting ATP synthase complex, catalytic core;CC:plasma membrane;CC:proton-transporting ATP synthase complex, catalytic core;CC:plasma membrane;CC:proton-transporting ATP synthase complex, catalytic core;CC:plasma membrane;MF:proton-transporting ATP synthase activity, rotational mechanism;MF:ATP binding;MF:hydrolase activity, acting on glycosyl bonds;MF:proton-transporting ATP synthase activity, rotational mechanism;MF:ATP binding;MF:proton-transporting ATP synthase activity, rotational mechanism;MF:proton-transporting ATP synthase activity, rotational mechanism;MF:ATP binding;
BAB_RS15990	-	hypothetical protein	0.982	-0.026110173	0.845883451	0.90775669	no	down	mRNA	678.3	670.56	678.22	692.08	684.84	653.44	675.693	676.786667	------	------	------	------
BAB_RS22460	-	iron ABC transporter permease	0.991	-0.013533793	0.847702345	0.907889211	no	down	mRNA	52.39	50.71	60.75	50.67	51.78	50.97	54.6167	51.14	COG0609	P:Inorganic ion transport and metabolism	GO:0016021;GO:0005886;GO:0005215	CC:integral component of membrane;CC:plasma membrane;MF:transporter activity;
BAB_RS32975	-	hypothetical protein	0.988	-0.016803674	0.84799763	0.907902827	no	down	mRNA	25.06	20.43	24.03	20.52	22.97	20.94	23.1733	21.4766667	------	------	------	------
BAB_RS15965	-	ABC transporter ATP-binding protein	0.992	-0.011566765	0.847489026	0.907963401	no	down	mRNA	66.47	62.74	67.79	63.86	61.82	58.32	65.6667	61.3333333	COG4172	S:Function unknown	GO:0015833;GO:0005524;GO:0016887	BP:peptide transport;MF:ATP binding;MF:ATPase activity;
BAB_RS22695	-	DUF2853 domain-containing protein	0.991	-0.01330099	0.847452572	0.90822719	no	down	mRNA	4158.68	3740.05	3767.53	3371.27	4052.39	3904.59	3888.75	3776.08333	ENOG4111U1X	S:Function unknown	------	------
BAB_RS22245	-	precorrin-4 C(11)-methyltransferase	0.99	-0.015138001	0.846660104	0.908286783	no	down	mRNA	24.46	20.75	23.37	20.28	22.25	21.16	22.86	21.23	COG2875	H:Coenzyme transport and metabolism	GO:0009236;GO:0055114;GO:0009236;GO:0055114;GO:0046026;GO:00	BP:cobalamin biosynthetic process;BP:oxidation-reduction process;BP:cobalamin biosynthetic process;BP:oxidation-reduction process;MF:precorrin-4 C11-methyltransferase activity;MF:exodeoxyribonuclease I activity;MF:precorrin-4 C11-methyltransferase activity;
BAB_RS18920	-	host specificity protein	0.967	-0.047963688	0.851302591	0.910228029	no	down	mRNA	0.88	1.76	1.66	1.06	1.32	1.65	1.43333	1.34333333	ENOG410XPQ3	S:Function unknown	------	------
BAB_RS28350	-	camphor resistance protein CrcB	0.981	-0.028209153	0.852474671	0.910874998	no	down	mRNA	35.84	31.9	41.57	30.87	39.01	33.14	36.4367	34.34	COG0239	S:Function unknown	GO:0016021;GO:0005887;GO:0005886;GO:0005887;GO:0015103;GO:00	CC:integral component of membrane;CC:integral component of plasma membrane;CC:plasma membrane;CC:integral component of plasma membrane;MF:inorganic anion transmembrane transporter activity;MF:inorganic anion transmembrane transporter activity;
BAB_RS31270	-	hypothetical protein	0.937	-0.093338122	0.853700749	0.910973267	no	down	mRNA	30.05	23.55	31.57	36.71	11.82	37.88	28.39	28.8033333	COG1071	S:Function unknown	GO:0016624	MF:oxidoreductase activity, acting on the aldehyde or oxo group of donors, disulfide as acceptor;
BAB_RS17220	-	hypothetical protein	0.888	-0.171813239	0.853196932	0.911040792	no	down	mRNA	15.14	0	3.14	2.4	5.23	5.27	6.09333	4.3	------	------	------	------
BAB_RS30835	-	heme-copper oxidase subunit III	0.963	-0.054120985	0.855683925	0.911575747	no	down	mRNA	6.12	8.25	5.57	3.87	7.31	7.07	6.64667	6.08333333	COG1845	P:Inorganic ion transport and metabolism	GO:0019646;GO:0019646;GO:0016021;GO:0005886;GO:0016021;GO:00	BP:aerobic electron transport chain;BP:aerobic electron transport chain;CC:integral component of membrane;CC:plasma membrane;CC:integral component of membrane;CC:plasma membrane;MF:cytochrome-c oxidase activity;MF:cytochrome-c oxidase activity;
BAB_RS27565	-	hypothetical protein	0.98	-0.029178308	0.856068925	0.911683612	no	down	mRNA	157.86	154.43	110.75	124.26	157.05	124.94	141.013	135.416667	ENOG410XUB6;ENOG4111JSB	K:Transcription;	GO:0006351;GO:0006351;GO:0003700;GO:0003677;GO:0003677;GO:00	BP:transcription, DNA-templated;BP:transcription, DNA-templated;MF:transcription factor activity, sequence-specific DNA binding;MF:DNA binding;MF:DNA binding;MF:transcription factor activity, sequence-specific DNA binding;
BAB_RS18910	-	peptidase P60	0.905	-0.144338567	0.855170914	0.912239093	no	down	mRNA	1.14	2	0.68	0.67	0.57	2.17	1.27333	1.13666667	COG0791	M:Cell wall/membrane/envelope biogenesis	------	------
BAB_RS27280	-	TIGR01459 family HAD-type hydrolase	0.987	-0.019179132	0.855466415	0.912251441	no	down	mRNA	63.02	58.13	67.41	68.46	56.02	51.27	62.8533	58.5833333	COG0647	G:Carbohydrate transport and metabolism	GO:0016787	MF:hydrolase activity;
BAB_RS29165	-	protocatechuate 3%2C4-dioxygenase subunit alpha	0.987	-0.019582585	0.857166999	0.912550553	no	down	mRNA	60.92	51.32	59.02	45.33	55.05	59.66	57.0867	53.3466667	COG3485	Q:Secondary metabolites biosynthesis, transport a	GO:0019439;GO:0019439;GO:0019439;GO:0019439;GO:0008199;GO:00	BP:aromatic compound catabolic process;BP:aromatic compound catabolic process;BP:aromatic compound catabolic process;BP:aromatic compound catabolic process;MF:ferric iron binding;MF:protocatechuate 3,4-dioxygenase activity;MF:ferric iron binding;MF:indoleacetylglucose-inositol O-acyltransferase activity;MF:protocatechuate 3,4-dioxygenase activity;MF:ferric iron binding;MF:protocatechuate 3,4-dioxygenase activity;MF:ferric iron binding;MF:protocatechuate 3,4-dioxygenase activity;MF:indoleacetylglucose-inositol O-acyltransferase activity;
BAB_RS18365	-	hypothetical protein	0.993	-0.009799159	0.861974899	0.91615129	no	down	mRNA	70.22	70.6	76.59	69.95	65.94	67.47	72.47	67.7866667	------	------	------	------
BAB_RS31410	-	hypothetical protein	0.983	-0.025089836	0.864374773	0.916277184	no	down	mRNA	22.22	16.75	20.79	20.81	16.87	17.8	19.92	18.4933333	COG1961	S:Function unknown	------	------
BAB_RS18410	-	cation transporter	0.975	-0.036331902	0.861914138	0.91638985	no	down	mRNA	4.18	4.99	6.57	4.45	5.54	4.73	5.24667	4.90666667	COG1230	P:Inorganic ion transport and metabolism	GO:0016021;GO:0008324	CC:integral component of membrane;MF:cation transmembrane transporter activity;
BAB_RS31295	-	peptide ABC transporter substrate-binding protein	0.981	-0.027859702	0.863376851	0.916731601	no	down	mRNA	6.21	6.7	5.56	6.66	5.4	5.01	6.15667	5.69	COG0747;ENOG410XS7C	S:Function unknown;	GO:0055085;GO:0055085;GO:0055085;GO:0043190;GO:0042597;GO:00	BP:transmembrane transport;BP:transmembrane transport;BP:transmembrane transport;CC:ATP-binding cassett;CC:periplasmic space;CC:ATP-binding cassett;CC:ATP-binding cassett;CC:periplasmic space;
BAB_RS22830	-	UDP-N-acetylenolpyruvoylglucosamine reductase	0.992	-0.010998735	0.865268071	0.916921606	no	down	mRNA	100.46	89.58	92.7	90.78	85.73	88.04	94.2467	88.1833333	COG0812	M:Cell wall/membrane/envelope biogenesis	GO:0071555;GO:0008360;GO:0051301;GO:0009252;GO:0007049;GO:00	BP:cell wall organization;BP:regulation of cell shape;BP:cell division;BP:peptidoglycan biosynthetic process;BP:cell cycle;CC:cytoplasm;MF:flavin adenine dinucleotide binding;MF:UDP-N-acetylmuramate dehydrogenase activity;MF:transferase activity;
BAB_RS21680	-	hypothetical protein	0.992	-0.011166148	0.866663902	0.917795358	no	down	mRNA	21703	22107.2	19991.3	18985.95	21540.82	21311.02	21267.2	20612.5967	ENOG411277S	S:Function unknown	GO:0016021	CC:integral component of membrane;
BAB_RS31405	-	four-carbon acid sugar kinase family protein	0.976	-0.035089914	0.867708416	0.91799379	no	down	mRNA	14.84	8.8	8.62	10.48	9.84	8.6	10.7533	9.64	COG3395	S:Function unknown	------	------
BAB_RS25790	-	shikimate dehydrogenase	0.992	-0.010862533	0.867587789	0.918471027	no	down	mRNA	107.63	96.66	100.49	93.87	99.46	92.15	101.593	95.16	COG0169	E:Amino acid transport and metabolism	GO:0009423;GO:0019632;GO:0009073;GO:0050661;GO:0016853;GO:00	BP:chorismate biosynthetic process;BP:shikimate metabolic process;BP:aromatic amino acid family biosynthetic process;MF:NADP binding;MF:isomerase activity;MF:shikimate 3-dehydrogenas;
BAB_RS16260	-	invasion associated locus B family protein	0.984	-0.02273734	0.869764796	0.918958991	no	down	mRNA	18.71	22.93	26.28	19.01	22.21	22.8	22.64	21.34	ENOG4111W93	S:Function unknown	------	------
BAB_RS21855	-	30S ribosomal protein S14	0.992	-0.011219477	0.87013675	0.919049763	no	down	mRNA	1232.93	1108.84	1179.11	1180.99	1092	1129.9	1173.63	1134.29667	COG0199	J:Translation, ribosomal structure and biogenesis	GO:0006412;GO:0006412;GO:0005840;GO:0005840;GO:0019843;GO:00	BP:translation;BP:translation;CC:ribosome;CC:ribosome;MF:rRNA binding;MF:structural constituent of ribosome;MF:structural constituent of ribosome;MF:rRNA binding;
BAB_RS33150	-	hypothetical protein	0.941	-0.087531607	0.870847493	0.919498191	no	down	mRNA	34.7	37.05	16.72	39.11	13.03	27.78	29.49	26.64	------	------	------	------
BAB_RS20760	-	arsenate reductase (glutaredoxin)	0.992	-0.01185105	0.875445999	0.922837269	no	down	mRNA	54.56	54.45	58.11	52.97	53.29	51	55.7067	52.42	COG1393	S:Function unknown	GO:0008794	MF:arsenate reductas;
BAB_RS32695	-	hypothetical protein	0.988	-0.017271166	0.87915783	0.924626549	no	down	mRNA	8.06	8.49	9.89	7.56	8.92	8.21	8.81333	8.23	------	------	------	------
BAB_RS23905	-	transcriptional repressor	0.992	-0.010979863	0.88006848	0.924978746	no	down	mRNA	113.23	93.28	119.03	92.84	103.62	106.79	108.513	101.083333	COG0735	S:Function unknown	GO:0006351;GO:0006351;GO:0005737;GO:0005737;GO:0003677;GO:00	BP:transcription, DNA-templated;BP:transcription, DNA-templated;CC:cytoplasm;CC:cytoplasm;MF:DNA binding;MF:transcription factor activity, sequence-specific DNA binding;MF:DNA binding;MF:transcription factor activity, sequence-specific DNA binding;MF:metal ion binding;MF:transcription factor activity, sequence-specific DNA binding;MF:metal ion binding;MF:DNA binding;
BAB_RS28040	-	MarR family transcriptional regulator	0.988	-0.0173614	0.880781632	0.925425567	no	down	mRNA	36.67	38.93	35.08	29.58	35.9	38.18	36.8933	34.5533333	ENOG4111TPH	K:Transcription	GO:0006351;GO:0006351;GO:0003700;GO:0003677;GO:0003677;GO:00	BP:transcription, DNA-templated;BP:transcription, DNA-templated;MF:transcription factor activity, sequence-specific DNA binding;MF:DNA binding;MF:DNA binding;MF:transcription factor activity, sequence-specific DNA binding;
BAB_RS28890	-	MFS transporter	0.985	-0.021791716	0.881806346	0.926199342	no	down	mRNA	8.64	6.16	7.58	6.98	7.38	6.15	7.46	6.83666667	ENOG410XP7I	S:Function unknown	GO:0016021;GO:0022857	CC:integral component of membrane;MF:transmembrane transporter activity;
BAB_RS26045	-	hypothetical protein	0.99	-0.014737145	0.884708759	0.92742879	no	down	mRNA	25.92	21.15	26.25	24.89	21.35	22.09	24.44	22.7766667	ENOG4111QUA	S:Function unknown	GO:0004674	MF:protein serine/threonine kinase activity;
BAB_RS18085	-	chorismate synthase	0.992	-0.01088644	0.883644767	0.927523893	no	down	mRNA	43.36	42.37	41.32	38.27	42.01	38.54	42.35	39.6066667	COG0082	E:Amino acid transport and metabolism	GO:0009073;GO:0009423;GO:0009423;GO:0009073;GO:0004107;GO:00	BP:aromatic amino acid family biosynthetic process;BP:chorismate biosynthetic process;BP:chorismate biosynthetic process;BP:aromatic amino acid family biosynthetic process;MF:chorismate synthase activity;MF:oxidoreductase activity;MF:chorismate synthase activity;
BAB_RS16595	-	murein biosynthesis integral membrane protein MurJ	0.993	-0.009805728	0.884397632	0.927707996	no	down	mRNA	54.08	50.51	50.41	46.32	50.1	48.55	51.6667	48.3233333	COG0728	S:Function unknown	GO:0071555;GO:0008360;GO:0009252;GO:0071555;GO:0009252;GO:00	BP:cell wall organization;BP:regulation of cell shape;BP:peptidoglycan biosynthetic process;BP:cell wall organization;BP:peptidoglycan biosynthetic process;BP:regulation of cell shape;BP:cell wall organization;BP:regulation of cell shape;BP:peptidoglycan biosynthetic process;CC:integral component of plasma membrane;CC:integral component of plasma membrane;CC:integral component of plasma membrane;MF:lipid-linked peptidoglycan transporter activity;MF:lipid-linked peptidoglycan transporter activity;MF:lipid-linked peptidoglycan transporter activity;
BAB_RS32700	-	hypothetical protein	0.948	-0.076432607	0.884704428	0.927726935	no	down	mRNA	17.26	32	14.84	22.83	28.61	7.6	21.3667	19.68	------	------	------	------
BAB_RS17340	-	urease accessory protein ureD 1	0.989	-0.015908879	0.88571747	0.92788074	no	down	mRNA	24.32	23.16	23.69	24.03	23.3	18.94	23.7233	22.09	COG0829	O:Posttranslational modification, protein turnover,	GO:0006807;GO:0006807;GO:0009405;GO:0005737;GO:0005737;GO:00	BP:nitrogen compound metabolic process;BP:nitrogen compound metabolic process;BP:pathogenesis;CC:cytoplasm;CC:cytoplasm;MF:nickel cation binding;MF:nickel cation binding;
BAB_RS20810	-	EamA/RhaT family transporter	0.981	-0.027814147	0.885453935	0.927907206	no	down	mRNA	7.24	7.84	6.93	8.5	5.77	6.22	7.33667	6.83	COG0697	J:Translation, ribosomal structure and biogenesis	GO:0016021	CC:integral component of membrane;
BAB_RS31760	-	DUF1634 domain-containing protein	0.981	-0.027307135	0.88654184	0.928441633	no	down	mRNA	9.92	7.42	7.93	6.92	8.63	7.55	8.42333	7.7	ENOG410Y4K8;ENOG4112DAS	S:Function unknown;	GO:0016021	CC:integral component of membrane;
BAB_RS27115	-	DUF4381 domain-containing protein	0.984	-0.02314645	0.887437525	0.928774192	no	down	mRNA	40.27	39.08	40.85	28.89	38.93	45.86	40.0667	37.8933333	ENOG410Z7HJ	S:Function unknown	GO:0016021	CC:integral component of membrane;
BAB_RS31650	-	AraC family transcriptional regulator	0.953	-0.070087406	0.893085927	0.933165881	no	down	mRNA	0.57	0.4	0.92	0.6	0.36	0.82	0.63	0.59333333	COG2207	S:Function unknown	GO:0006351;GO:0003700;GO:0043565	BP:transcription, DNA-templated;MF:transcription factor activity, sequence-specific DNA binding;MF:sequence-specific DNA binding;
BAB_RS31565	-	flagellar biosynthesis regulator FlaF	0.944	-0.082584136	0.893570931	0.933369116	no	down	mRNA	4.9	2.06	5.99	1.71	3.41	7.41	4.31667	4.17666667	COG5442	N:Cell motility	GO:0044781	BP:bacterial-type flagellum organization;
BAB_RS31745	-	branched-chain amino acid ABC transporter permease	0.958	-0.062593115	0.894483466	0.933715197	no	down	mRNA	0.98	0.58	1.8	0.79	1.42	0.84	1.12	1.01666667	COG0559	E:Amino acid transport and metabolism	GO:0016021;GO:0005886;GO:0022857	CC:integral component of membrane;CC:plasma membrane;MF:transmembrane transporter activity;
BAB_RS26920	-	hypothetical protein	0.991	-0.013375244	0.894366327	0.93389633	no	down	mRNA	23.29	22.79	28.69	24.37	24.79	20.7	24.9233	23.2866667	ENOG4111W4Z	S:Function unknown	------	------
BAB_RS25105	-	dihydrolipoyllysine-residue succinyltransferase	0.995	-0.007676014	0.896767736	0.935491797	no	down	mRNA	732.61	716.22	824.89	669	747.78	714.82	757.907	710.533333	COG0508	S:Function unknown	GO:0006099;GO:0033512;GO:0033512;GO:0006099;GO:0006099;GO:00	BP:tricarboxylic acid cycle;BP:L-lysine catabolic process to acetyl-CoA via saccharopine;BP:L-lysine catabolic process to acetyl-CoA via saccharopine;BP:tricarboxylic acid cycle;BP:tricarboxylic acid cycle;BP:L-lysine catabolic process to acetyl-CoA via saccharopine;CC:oxoglutarate dehydrogenase complex;CC:oxoglutarate dehydrogenase complex;CC:oxoglutarate dehydrogenase complex;MF:dihydrolipoyllysine-residue succinyltransferase activity;MF:dihydrolipoyllysine-residue succinyltransferase activity;MF:hydrolase activity;MF:dihydrolipoyllysine-residue succinyltransferase activity;
BAB_RS19045	-	thiol:disulfide oxidoreductase	0.995	-0.007375997	0.89652475	0.935542066	no	down	mRNA	84.87	79.64	86.68	73.92	80.64	80.7	83.73	78.42	COG0625	O:Posttranslational modification, protein turnover,	GO:0016740;GO:0004364	MF:transferase activity;MF:glutathione transferase activity;
BAB_RS29835	-	K+/H+ antiporter subunit F	0.979	-0.029986117	0.897491323	0.935942753	no	down	mRNA	61.59	54.25	54.14	37.78	64.87	62.97	56.66	55.2066667	COG2212	P:Inorganic ion transport and metabolism	GO:0016021;GO:0015075	CC:integral component of membrane;MF:ion transmembrane transporter activity;
BAB_RS22225	-	hydrogenobyrinate a%2Cc-diamide synthase	0.994	-0.008334593	0.898238354	0.936417855	no	down	mRNA	60.73	61.61	67.87	57.16	62.74	58.52	63.4033	59.4733333	COG1797	H:Coenzyme transport and metabolism	GO:0009236;GO:0006541;GO:0009236;GO:0006541;GO:0006541;GO:00	BP:cobalamin biosynthetic process;BP:glutamine metabolic process;BP:cobalamin biosynthetic process;BP:glutamine metabolic process;BP:glutamine metabolic process;BP:cobalamin biosynthetic process;MF:ATP binding;MF:cobyrinic acid a,c-diamide synthase activity;MF:hydrogenobyrinic acid a,c-diamide synthas;MF:lyase activity;MF:ATP binding;MF:cobyrinic acid a,c-diamide synthase activity;MF:hydrogenobyrinic acid a,c-diamide synthas;MF:cobyrinic acid a,c-diamide synthase activity;MF:hydrogenobyrinic acid a,c-diamide synthas;MF:lyase activity;MF:ATP binding;
BAB_RS21070	-	3-mercaptopyruvate sulfurtransferase	0.995	-0.006993612	0.899053096	0.936963217	no	down	mRNA	401.89	366.27	405.28	344.63	370.81	387.91	391.147	367.783333	COG2897	P:Inorganic ion transport and metabolism	GO:0004792	MF:thiosulfate sulfurtransferase activity;
BAB_RS29230	-	saccharopine dehydrogenase	0.993	-0.010246464	0.901714363	0.938823152	no	down	mRNA	32.08	32.21	32.39	27.64	30.25	32.76	32.2267	30.2166667	COG1748	S:Function unknown	GO:0016491	MF:oxidoreductase activity;
BAB_RS19290	-	4-hydroxythreonine-4-phosphate dehydrogenase PdxA	0.993	-0.010660321	0.902444085	0.939278537	no	down	mRNA	58.63	65.88	62.83	63.21	55.69	57.33	62.4467	58.7433333	COG1995	H:Coenzyme transport and metabolism	GO:0042823;GO:0008615;GO:0042823;GO:0008615;GO:0042823;GO:00	BP:pyridoxal phosphate biosynthetic process;BP:pyridoxine biosynthetic process;BP:pyridoxal phosphate biosynthetic process;BP:pyridoxine biosynthetic process;BP:pyridoxal phosphate biosynthetic process;BP:pyridoxine biosynthetic process;CC:cytoplasm;CC:cytoplasm;CC:cytoplasm;MF:4-hydroxythreonine-4-phosphate dehydrogenase activity;MF:cobalt ion binding;MF:magnesium ion binding;MF:NAD binding;MF:zinc ion binding;MF:magnesium ion binding;MF:NAD binding;MF:cobalt ion binding;MF:zinc ion binding;MF:4-hydroxythreonine-4-phosphate dehydrogenase activity;MF:metal ion binding;MF:NAD binding;MF:cobalt ion binding;MF:zinc ion binding;MF:4-hydroxythreonine-4-phosphate dehydrogenase activity;MF:magnesium ion binding;
BAB_RS32625	-	hypothetical protein	0.991	-0.013518654	0.904624961	0.941243523	no	down	mRNA	36.46	31.64	44.51	34.57	34.73	36.69	37.5367	35.33	------	------	------	------
BAB_RS16655	-	organic solvent tolerance protein OstA	0.996	-0.006077813	0.906919534	0.942105549	no	down	mRNA	328.98	299.67	332.94	305.05	302.22	300.15	320.53	302.473333	COG1934	S:Function unknown	------	------
BAB_RS21865	-	50S ribosomal protein L24	0.993	-0.010516227	0.906877742	0.942366813	no	down	mRNA	790.34	697.86	739.69	779.19	643.92	738.76	742.63	720.623333	COG0198	J:Translation, ribosomal structure and biogenesis	GO:0006412;GO:0006412;GO:0005840;GO:0005840;GO:0003735;GO:00	BP:translation;BP:translation;CC:ribosome;CC:ribosome;MF:structural constituent of ribosome;MF:rRNA binding;MF:structural constituent of ribosome;MF:rRNA binding;
BAB_RS17525	-	Co2+/Mg2+ efflux protein ApaG	0.993	-0.0096218	0.909767734	0.944453547	no	down	mRNA	18.88	18.39	23.55	19.42	19.39	18.1	20.2733	18.97	COG2967	P:Inorganic ion transport and metabolism	------	------
BAB_RS30665	-	Crp/Fnr family transcriptional regulator	0.992	-0.011722216	0.913020192	0.946912162	no	down	mRNA	34.48	40.32	45.05	37.5	40.2	35.43	39.95	37.71	COG0664	C:Energy production and conversion	GO:0006355;GO:0006351;GO:0006355;GO:0006351;GO:0003677;GO:00	BP:regulation of transcription, DNA-templated;BP:transcription, DNA-templated;BP:regulation of transcription, DNA-templated;BP:transcription, DNA-templated;MF:DNA binding;MF:DNA binding;
BAB_RS21905	-	50S ribosomal protein L2	0.996	-0.006405976	0.912876833	0.947069184	no	down	mRNA	1178.21	1035.81	1138.95	997.86	1119.74	1037.06	1117.66	1051.55333	COG0090	J:Translation, ribosomal structure and biogenesis	GO:0006412;GO:0006412;GO:0015934;GO:0015934;GO:0003735;GO:00	BP:translation;BP:translation;CC:large ribosomal subunit;CC:large ribosomal subunit;MF:structural constituent of ribosome;MF:transferase activity;MF:rRNA binding;MF:rRNA binding;MF:structural constituent of ribosome;MF:transferase activity;
BAB_RS25940	-	nucleotidyltransferase family protein	0.995	-0.007915652	0.914976046	0.947106326	no	down	mRNA	75.07	80.73	85.75	76.44	77.12	74.68	80.5167	76.08	COG1208	M:Cell wall/membrane/envelope biogenesis	GO:0016740;GO:0016779;GO:0016740;GO:0016779;GO:0004475	MF:transferase activity;MF:nucleotidyltransferase activity;MF:transferase activity;MF:nucleotidyltransferase activity;MF:mannose-1-phosphate guanylyltransferase activity;
BAB_RS21700	-	XRE family transcriptional regulator	0.996	-0.005561721	0.914714607	0.947140842	no	down	mRNA	277.53	258.49	274.62	244.29	256.74	259.15	270.213	253.393333	COG1396;COG3800	S:Function unknown;K:Transcription	GO:0006355;GO:0043565	BP:regulation of transcription, DNA-templated;MF:sequence-specific DNA binding;
BAB_RS25975	-	hypothetical protein	0.992	-0.011586445	0.914671415	0.947401437	no	down	mRNA	97	99.21	113.89	90.42	111.69	92.87	103.367	98.3266667	------	------	------	------
BAB_RS32345	-	hypothetical protein	0.964	-0.052390564	0.915871836	0.94742312	no	down	mRNA	1.67	2.6	1.04	2	1.19	1.71	1.77	1.63333333	COG5449	S:Function unknown	------	------
BAB_RS29415	-	DNA topoisomerase IV subunit B	0.996	-0.005482047	0.914176629	0.947499842	no	down	mRNA	270.55	262.12	295.09	249.27	266.56	259.8	275.92	258.543333	COG0187	J:Translation, ribosomal structure and biogenesis	GO:0007059;GO:0006265;GO:0006265;GO:0007059;GO:0005694;GO:00	BP:chromosome segregation;BP:DNA topological change;BP:DNA topological change;BP:chromosome segregation;CC:chromosome;CC:chromosome;MF:ATP binding;MF:DNA binding;MF:DNA topoisomerase type I;MF:DNA binding;MF:DNA topoisomerase type I;MF:ATP binding;
BAB_RS17740	-	xanthine dehydrogenase molybdopterin binding subunit	0.996	-0.006443568	0.915693991	0.947544216	no	down	mRNA	58.95	54.51	58.86	51.45	57.15	52.77	57.44	53.79	COG4631	F:Nucleotide transport and metabolism	------	------
BAB_RS27675	-	methionine ABC transporter ATP-binding protein	0.993	-0.010297693	0.913951908	0.947572598	no	down	mRNA	47.27	44.53	42.77	47.07	38.91	40.03	44.8567	42.0033333	COG1135	P:Inorganic ion transport and metabolism	GO:0015821;GO:0015821;GO:0005886;GO:0005886;GO:0005524;GO:00	BP:methionine transport;BP:methionine transport;CC:plasma membrane;CC:plasma membrane;MF:ATP binding;MF:ATPase activity, coupled to transmembrane movement of substances;MF:amino acid-transporting ATPase activity;MF:ATP binding;
BAB_RS25855	-	DUF2628 domain-containing protein	0.993	-0.009850041	0.92320966	0.954092196	no	down	mRNA	70.25	82.1	80.39	75.6	69.39	77.2	77.58	74.0633333	ENOG410Y0HN;ENOG410ZT6I;ENOG41102NA	S:Function unknown;	GO:0016021	CC:integral component of membrane;
BAB_RS17140	-	hypothetical protein	0.985	-0.021713868	0.925774469	0.954287253	no	down	mRNA	10.32	8.37	9.45	7.09	11.53	7.19	9.38	8.60333333	------	------	------	------
BAB_RS31025	-	methionyl-tRNA formyltransferase	0.995	-0.006717568	0.925753187	0.954571563	no	down	mRNA	45.71	44.14	46.88	44.8	42.04	41.81	45.5767	42.8833333	COG0223	J:Translation, ribosomal structure and biogenesis	GO:0004479;GO:0004479;GO:0016746	MF:methionyl-tRNA formyltransferase activity;MF:methionyl-tRNA formyltransferase activity;MF:transferase activity, transferring acyl groups;
BAB_RS18700	-	AbrB/MazE/SpoVT family DNA-binding domain-containing protein	0.985	-0.02210511	0.925216475	0.954630871	no	down	mRNA	173.52	203.8	211.82	181.32	205.21	234.16	196.38	206.896667	ENOG41123JA	K:Transcription	GO:0003677	MF:DNA binding;
BAB_RS29815	-	thiol reductant ABC exporter subunit CydC	0.996	-0.00640574	0.925532262	0.954650131	no	down	mRNA	97.65	109.47	115.44	99.33	102.24	101.44	107.52	101.003333	COG4987	V:Defense mechanisms	GO:0034775;GO:0045454;GO:0045454;GO:0034775;GO:0016021;GO:00	BP:glutathione transmembrane transport;BP:cell redox homeostasis;BP:cell redox homeostasis;BP:glutathione transmembrane transport;CC:integral component of membrane;CC:cell;CC:cell;CC:integral component of membrane;MF:ATPase activity, coupled to transmembrane movement of substances;MF:ATP binding;MF:ATP binding;MF:ATPase activity, coupled to transmembrane movement of substances;
BAB_RS19285	-	ribosomal RNA small subunit methyltransferase A	0.995	-0.006719292	0.927678136	0.955023983	no	down	mRNA	34.79	34.42	39.48	35.72	31.72	34.68	36.23	34.04	COG0030	J:Translation, ribosomal structure and biogenesis	GO:0005737;GO:0005737;GO:0052908;GO:0000179;GO:0003723;GO:00	CC:cytoplasm;CC:cytoplasm;MF:16S rRN;MF:rRN;MF:RNA binding;MF:16S rRN;MF:RNA binding;MF:rRN;
BAB_RS28655	-	FUSC family protein	0.995	-0.006527776	0.928026691	0.955076796	no	down	mRNA	15.77	15.74	17.63	15.83	15.37	14.88	16.38	15.36	COG1289	K:Transcription	GO:0016021;GO:0005886;GO:0022857	CC:integral component of membrane;CC:plasma membrane;MF:transmembrane transporter activity;
BAB_RS25635	-	-	0.988	-0.017406476	0.929079989	0.955242882	no	down	mRNA	20.57	16.72	21.41	15.15	23.01	16.8	19.5667	18.32	------	------	------	------
BAB_RS19960	-	DNA polymerase III subunit alpha	0.997	-0.005021068	0.927079863	0.955326363	no	down	mRNA	135.26	127.72	139.64	118.5	132.56	126.16	134.207	125.74	COG0587	V:Defense mechanisms	GO:0006260;GO:0006260;GO:0005737;GO:0005737;GO:0003887;GO:00	BP:DNA replication;BP:DNA replication;CC:cytoplasm;CC:cytoplasm;MF:DNA-directed DNA polymerase activity;MF:3'-5' exonuclease activity;MF:nucleic acid binding;MF:3'-5' exonuclease activity;MF:DNA-directed DNA polymerase activity;MF:nucleic acid binding;MF:oxidoreductase activity;
BAB_RS29610	-	IS66 family transposase	0.994	-0.008142417	0.929044539	0.955512197	no	down	mRNA	22.74	19.64	20.49	18.26	21.41	18.93	20.9567	19.5333333	COG3436	L:Replication, recombination and repair	------	------
BAB_RS18165	-	CDP-diacylglycerol--serine O-phosphatidyltransferase	0.995	-0.007093449	0.92889453	0.955663825	no	down	mRNA	46.46	45.08	45.8	44.02	40.19	45.16	45.78	43.1233333	COG1183	I:Lipid transport and metabolism	GO:0102991;GO:0008654;GO:0008654;GO:0016021;GO:0016020;GO:00	old GO;BP:phospholipid biosynthetic process;BP:phospholipid biosynthetic process;CC:integral component of membrane;CC:membrane;CC:integral component of membrane;MF:phosphotransferase activity, for other substituted phosphate groups;MF:palmitoyl-CoA hydrolase activity;MF:CDP-diacylglycerol-serine O-phosphatidyltransferase activity;MF:old GO;MF:palmitoyl-CoA hydrolase activity;MF:phosphotransferase activity, for other substituted phosphate groups;
BAB_RS20170	-	uracil-DNA glycosylase	0.993	-0.010270418	0.930838179	0.956132694	no	down	mRNA	32.95	35.06	40.09	37.11	35.62	29.27	36.0333	34	COG1573	K:Transcription	------	------
BAB_RS31070	-	glutathione S-transferase family protein	0.995	-0.006773168	0.932169878	0.956888759	no	down	mRNA	102.7	90.81	92.23	92.87	93.68	81.72	95.2467	89.4233333	ENOG410XPYM	O:Posttranslational modification, protein turnover,	GO:0016740;GO:0016740;GO:0004364	MF:transferase activity;MF:transferase activity;MF:glutathione transferase activity;
BAB_RS31230	-	dipeptide ABC transporter ATP-binding protein	0.993	-0.010602403	0.933115805	0.956942573	no	down	mRNA	21.98	20.74	25.22	24.78	20.93	18	22.6467	21.2366667	COG4608	L:Replication, recombination and repair	GO:0015833;GO:0015833;GO:0005524;GO:0016887;GO:0005524;GO:00	BP:peptide transport;BP:peptide transport;MF:ATP binding;MF:ATPase activity;MF:ATP binding;MF:ATPase activity;
BAB_RS20490	-	SUF system Fe-S cluster assembly protein	0.994	-0.008143491	0.933012932	0.957142576	no	down	mRNA	57.56	50.15	69.34	56.2	59.7	49.51	59.0167	55.1366667	COG2151	S:Function unknown	------	------
BAB_RS19700	-	N utilization substance protein B	0.996	-0.00566615	0.934923966	0.95757434	no	down	mRNA	245	202.94	228.35	222.9	204.42	211.92	225.43	213.08	COG0781	K:Transcription	GO:0031564;GO:0006353;GO:0006353;GO:0031564;GO:0003723;GO:00	BP:transcription antitermination;BP:DNA-templated transcription, termination;BP:DNA-templated transcription, termination;BP:transcription antitermination;MF:RNA binding;MF:RNA binding;
BAB_RS17215	-	XRE family transcriptional regulator	0.985	-0.022476969	0.935775895	0.958141476	no	down	mRNA	23.26	27.52	32.06	25.31	34.88	22.57	27.6133	27.5866667	COG1396;ENOG41120I2	K:Transcription;	GO:0043565	MF:sequence-specific DNA binding;
BAB_RS22640	-	acetolactate synthase small subunit	0.996	-0.005077241	0.93805713	0.958949271	no	down	mRNA	77.74	72.49	75.09	70.52	72.05	69.72	75.1067	70.7633333	COG0440	E:Amino acid transport and metabolism	GO:0009082;GO:0009082;GO:0050630;GO:0003984;GO:0102719;GO:00	BP:branched-chain amino acid biosynthetic process;BP:branched-chain amino acid biosynthetic process;MF:;MF:acetolactate synthase activity;MF:old GO;MF:acetolactate synthase activity;
BAB_RS20145	-	TIGR02281 family clan AA aspartic protease	0.995	-0.007726109	0.937317986	0.959109137	no	down	mRNA	20.49	20.95	20.96	18.99	20.69	18.96	20.8	19.5466667	COG3577	S:Function unknown	GO:0016021;GO:0016021;GO:0004190;GO:0004190	CC:integral component of membrane;CC:integral component of membrane;MF:aspartic-type endopeptidase activity;MF:aspartic-type endopeptidase activity;
BAB_RS32245	-	hypothetical protein	0.995	-0.006578904	0.937748155	0.959243815	no	down	mRNA	33.33	33.12	40.01	34	33.14	33.25	35.4867	33.4633333	------	------	------	------
BAB_RS19510	-	hypothetical protein	0.997	-0.004693202	0.941826895	0.961273765	no	down	mRNA	411.85	362.33	379.49	343.7	383.55	355.75	384.557	361	ENOG410ZYWB;ENOG4111MQC;ENOG410ZQ5W	S:Function unknown;	------	------
BAB_RS24000	-	Lrp/AsnC family transcriptional regulator	0.994	-0.008456107	0.94346803	0.962642992	no	down	mRNA	21.87	21.32	18.92	19.76	19.63	18.9	20.7033	19.43	COG1522	F:Nucleotide transport and metabolism	GO:0006351;GO:0005622;GO:0003700;GO:0043565	BP:transcription, DNA-templated;CC:intracellular;MF:transcription factor activity, sequence-specific DNA binding;MF:sequence-specific DNA binding;
BAB_RS21850	-	30S ribosomal protein S8	0.997	-0.00393106	0.944937783	0.963530656	no	down	mRNA	1199.67	1036.7	1142.13	1112.58	1071.28	1042.83	1126.17	1075.56333	COG0096	J:Translation, ribosomal structure and biogenesis	GO:0006412;GO:0006412;GO:0005840;GO:0005840;GO:0019843;GO:00	BP:translation;BP:translation;CC:ribosome;CC:ribosome;MF:rRNA binding;MF:structural constituent of ribosome;MF:structural constituent of ribosome;MF:rRNA binding;
BAB_RS19355	-	molybdopterin converting factor subunit 1	0.992	-0.01120245	0.944654463	0.963547552	no	down	mRNA	1070.83	818.63	1012.52	1084.75	991.19	828.2	967.327	968.046667	COG1977	H:Coenzyme transport and metabolism	GO:0006777;GO:0006777;GO:0000166;GO:0000166	BP:Mo-molybdopterin cofactor biosynthetic process;BP:Mo-molybdopterin cofactor biosynthetic process;MF:nucleotide binding;MF:nucleotide binding;
BAB_RS26815	-	LysR family transcriptional regulator	0.996	-0.005458085	0.946227101	0.964233325	no	down	mRNA	64.49	64.6	63.28	55	65.23	60.87	64.1233	60.3666667	ENOG410XQWS	K:Transcription	GO:0006351;GO:0006351;GO:0003700;GO:0003677;GO:0003677;GO:00	BP:transcription, DNA-templated;BP:transcription, DNA-templated;MF:transcription factor activity, sequence-specific DNA binding;MF:DNA binding;MF:DNA binding;MF:transcription factor activity, sequence-specific DNA binding;
BAB_RS33080	-	hypothetical protein	0.989	-0.016244617	0.947190448	0.964603141	no	down	mRNA	195.83	208.8	145.08	176.43	196.83	174.59	183.237	182.616667	COG0725	P:Inorganic ion transport and metabolism	------	------
BAB_RS27125	-	LysR family transcriptional regulator	0.995	-0.006659282	0.947084387	0.964800931	no	down	mRNA	16.77	18.28	18.98	16.64	16.56	17.84	18.01	17.0133333	ENOG410XQGJ	K:Transcription	GO:0006351;GO:0006351;GO:0003700;GO:0003677;GO:0003677;GO:00	BP:transcription, DNA-templated;BP:transcription, DNA-templated;MF:transcription factor activity, sequence-specific DNA binding;MF:DNA binding;MF:DNA binding;MF:transcription factor activity, sequence-specific DNA binding;
BAB_RS32320	-	FadR family transcriptional regulator	0.989	-0.015592044	0.951032433	0.967595696	no	down	mRNA	5.93	6.59	6.23	5.56	5.35	7.04	6.25	5.98333333	COG2186	K:Transcription	GO:0006351;GO:0003677;GO:0003700	BP:transcription, DNA-templated;MF:DNA binding;MF:transcription factor activity, sequence-specific DNA binding;
BAB_RS22805	-	UDP-3-O-[3-hydroxymyristoyl] N-acetylglucosamine deacetylase	0.997	-0.004718778	0.9524195	0.968087268	no	down	mRNA	110.91	88.04	95.54	96.15	88.2	91.52	98.1633	91.9566667	COG0774	M:Cell wall/membrane/envelope biogenesis	------	------
BAB_RS24540	-	primosomal protein N'	0.996	-0.005533398	0.952280643	0.968252439	no	down	mRNA	22.32	21.43	30.03	23.08	23.34	22.61	24.5933	23.01	COG1198	L:Replication, recombination and repair	GO:0006268;GO:0006269;GO:1990077;GO:0004003;GO:0008270;GO:00	BP:DNA unwinding involved in DNA replication;BP:DNA replication, synthesis of RNA primer;CC:primosome complex;MF:ATP-dependent DNA helicase activity;MF:zinc ion binding;MF:DNA binding;MF:ATP binding;
BAB_RS16060	-	putative sulfate exporter family transporter	0.995	-0.00793387	0.951988509	0.968261817	no	down	mRNA	17.27	15.86	19.32	15.51	14.08	19.83	17.4833	16.4733333	COG2855	S:Function unknown	GO:0016021;GO:0005886	CC:integral component of membrane;CC:plasma membrane;
BAB_RS27265	-	hypothetical protein	0.997	-0.003873854	0.956685491	0.970274774	no	down	mRNA	1046.02	947.97	817.6	868.87	962.02	950.36	937.197	927.083333	ENOG410Z7N3	S:Function unknown	------	------
BAB_RS20880	-	glutamate--tRNA ligase 1	0.998	-0.003054476	0.95649414	0.970387013	no	down	mRNA	64.48	60.1	64.83	59.26	60.99	57.62	63.1367	59.29	COG0008	J:Translation, ribosomal structure and biogenesis	GO:0006424;GO:0006424;GO:0006424;GO:0006424;GO:0005737;GO:00	BP:glutamyl-tRNA aminoacylation;BP:glutamyl-tRNA aminoacylation;BP:glutamyl-tRNA aminoacylation;BP:glutamyl-tRNA aminoacylation;CC:cytoplasm;CC:cytoplasm;CC:cytoplasm;CC:cytoplasm;MF:hydrolase activity;MF:tRNA binding;MF:glutamate-tRNA ligase activity;MF:zinc ion binding;MF:ATP binding;MF:tRNA binding;MF:glutamate-tRNA ligase activity;MF:zinc ion binding;MF:ATP binding;MF:glutamate-tRNA ligase activity;MF:hydrolase activity;MF:tRNA binding;MF:zinc ion binding;MF:ATP binding;MF:glutamate-tRNA ligase activity;MF:tRNA binding;MF:zinc ion binding;
BAB_RS19465	-	-	0.994	-0.009138675	0.96445367	0.976304235	no	down	mRNA	13.9	18.34	13.25	16.02	13.57	13.92	15.1633	14.5033333	------	------	------	------
BAB_RS26585	-	histidinol-phosphatase	0.998	-0.00245177	0.968138067	0.979416753	no	down	mRNA	167.13	165.29	180.45	153.36	174.33	154.83	170.957	160.84	COG0483	E:Amino acid transport and metabolism	GO:0046854;GO:0046854;GO:0004401;GO:0004401	BP:phosphatidylinositol phosphorylation;BP:phosphatidylinositol phosphorylation;MF:histidinol-phosphatase activity;MF:histidinol-phosphatase activity;
BAB_RS16795	-	hypothetical protein	0.998	-0.002920808	0.969778433	0.980458812	no	down	mRNA	116.6	115.45	138.58	113.3	123.61	114.21	123.543	117.04	ENOG410Y71F	S:Function unknown	GO:0016021	CC:integral component of membrane;
BAB_RS18950	-	c-type cytochrome biogenesis protein CcmI	0.998	-0.002872469	0.972630801	0.982415204	no	down	mRNA	124.08	110.54	118.08	122.73	110.59	98.3	117.567	110.54	COG4235	O:Posttranslational modification, protein turnover,	GO:0016021	CC:integral component of membrane;
BAB_RS24345	-	hypothetical protein	0.998	-0.002957891	0.97232514	0.982415307	no	down	mRNA	1843.74	2096.73	2036.56	1849.03	2078.41	1888.83	1992.34	1938.75667	------	------	GO:0016021;GO:0019867	CC:integral component of membrane;CC:outer membrane;
BAB_RS24870	-	bifunctional 3-demethylubiquinone 3-O-methyltransferase/2-octaprenyl-6-hydroxy phenol methylase	0.999	-0.001773683	0.976424927	0.985937552	no	down	mRNA	800.55	769.83	834.93	712.8	766.86	780.08	801.77	753.246667	COG2227	H:Coenzyme transport and metabolism	GO:0006744;GO:0006744;GO:0006744;GO:0008425;GO:0008689;GO:00	BP:ubiquinone biosynthetic process;BP:ubiquinone biosynthetic process;BP:ubiquinone biosynthetic process;MF:2-polyprenyl-6-methoxy-1,4-benzoquinone methyltransferase activity;MF:3-demethylubiquinone-9 3-O-methyltransferase activity;MF:2-polyprenyl-6-methoxy-1,4-benzoquinone methyltransferase activity;MF:3-demethylubiquinone-9 3-O-methyltransferase activity;MF:3-demethylubiquinone-9 3-O-methyltransferase activity;MF:2-polyprenyl-6-methoxy-1,4-benzoquinone methyltransferase activity;MF:oxidoreductase activity;
BAB_RS27185	-	LysR family transcriptional regulator	0.998	-0.002249069	0.97819187	0.987411398	no	down	mRNA	30.95	26.61	31.97	26.64	29.71	27.76	29.8433	28.0366667	ENOG410XQWS	S:Function unknown	GO:0006351;GO:0006351;GO:0003700;GO:0003677;GO:0003677;GO:00	BP:transcription, DNA-templated;BP:transcription, DNA-templated;MF:transcription factor activity, sequence-specific DNA binding;MF:DNA binding;MF:DNA binding;MF:transcription factor activity, sequence-specific DNA binding;
BAB_RS31595	-	flagellar hook-length control protein FliK	0.997	-0.004701007	0.979371591	0.987981451	no	down	mRNA	3.98	4.33	3.39	3.75	3.51	3.73	3.9	3.66333333	ENOG410ZV2B	N:Cell motility	------	------
BAB_RS19460	-	ETC complex I subunit region	0.999	-0.001387709	0.980737433	0.989048767	no	down	mRNA	503.11	453.3	466.07	467.95	438.75	443.59	474.16	450.096667	ENOG4111TR4	C:Energy production and conversion	GO:0022900;GO:0022900;GO:0008137;GO:0016651;GO:0016651	BP:electron transport chain;BP:electron transport chain;MF:NADH dehydrogenas;MF:oxidoreductase activity, acting on NA;MF:oxidoreductase activity, acting on NA;
BAB_RS24310	-	50S ribosomal protein L36	0.994	-0.009085414	0.983680875	0.990773245	no	down	mRNA	599.81	387.63	512.97	614.97	607.2	308.21	500.137	510.126667	COG0257	J:Translation, ribosomal structure and biogenesis	GO:0006412;GO:0005840;GO:0003735	BP:translation;CC:ribosome;MF:structural constituent of ribosome;
BAB_RS22230	-	uroporphyrinogen-III C-methyltransferase	0.999	-0.001298534	0.983602205	0.991004668	no	down	mRNA	94.79	89.86	95.23	83.28	88.56	92.48	93.2933	88.1066667	COG0007	H:Coenzyme transport and metabolism	GO:0019354;GO:0055114;GO:0055114;GO:0019354;GO:0045552;GO:00	BP:siroheme biosynthetic process;BP:oxidation-reduction process;BP:oxidation-reduction process;BP:siroheme biosynthetic process;MF:dihydrokaempferol 4-reductase activity;MF:uroporphyrin-III C-methyltransferase activity;MF:methyltransferase activity;MF:methyltransferase activity;
BAB_RS16195	-	LysR family transcriptional regulator	0.999	-0.002062098	0.984593574	0.991381747	no	down	mRNA	34.19	25.33	34.43	28.41	28.66	31.21	31.3167	29.4266667	ENOG410XRUN	K:Transcription	GO:0006351;GO:0006351;GO:0003700;GO:0003677;GO:0003677;GO:00	BP:transcription, DNA-templated;BP:transcription, DNA-templated;MF:transcription factor activity, sequence-specific DNA binding;MF:DNA binding;MF:DNA binding;MF:transcription factor activity, sequence-specific DNA binding;
BAB_RS26090	-	SMP-30/gluconolactonase/LRE family protein	0.999	-0.001317739	0.984906592	0.99138624	no	down	mRNA	71.54	70.72	69.77	61.89	69.18	68.76	70.6767	66.61	COG3386	G:Carbohydrate transport and metabolism	------	------
BAB_RS32885	-	hypothetical protein	0.998	-0.003121847	0.986216558	0.991772708	no	down	mRNA	9.19	9.87	8.09	6.85	8.76	10	9.05	8.53666667	------	------	------	------
BAB_RS16560	-	IS5/IS1182 family transposase	0.989	-0.016670147	0.985723878	0.991898158	no	down	mRNA	0	2.05	2.75	3.27	1.85	0	1.6	1.70666667	COG3293	L:Replication, recombination and repair	GO:0006313;GO:0016021;GO:0003677;GO:0004803	BP:transposition, DNA-mediated;CC:integral component of membrane;MF:DNA binding;MF:transposase activity;
BAB_RS29705	-	IS5/IS1182 family transposase	0.989	-0.016670147	0.985723878	0.991898158	no	down	mRNA	0	2.05	2.75	3.27	1.85	0	1.6	1.70666667	COG3293	L:Replication, recombination and repair	GO:0006313;GO:0016021;GO:0003677;GO:0004803	BP:transposition, DNA-mediated;CC:integral component of membrane;MF:DNA binding;MF:transposase activity;
BAB_RS24980	-	ammonium transporter	0.999	-0.00162561	0.992135879	0.995544216	no	down	mRNA	5.57	6.36	8.22	5.28	6.66	7.24	6.71667	6.39333333	ENOG410XNMH	D:Cell cycle control, cell division, chromosome par	GO:0016021;GO:0016021;GO:0008519;GO:0008519	CC:integral component of membrane;CC:integral component of membrane;MF:ammonium transmembrane transporter activity;MF:ammonium transmembrane transporter activity;
BAB_RS20815	-	haloacid dehalogenase	0.999	-0.001308732	0.993394385	0.996495835	no	down	mRNA	13.81	13.03	15.15	12.36	15.63	11.64	13.9967	13.21	COG0546	J:Translation, ribosomal structure and biogenesis	GO:0008152;GO:0008152;GO:0016787;GO:0008967;GO:0016787	BP:metabolic process;BP:metabolic process;MF:hydrolase activity;MF:phosphoglycolate phosphatase activity;MF:hydrolase activity;
BAB_RS16150	-	translocation/assembly module TamB	1	-0.000331947	0.994876719	0.997048939	no	down	mRNA	209.18	201.1	214.44	193.81	193.15	200.41	208.24	195.79	COG2911	S:Function unknown	------	------
BAB_RS23185	-	membrane protein	1	-0.000600795	0.994480727	0.997274212	no	down	mRNA	49.49	46.73	55.35	43.81	50.84	48.9	50.5233	47.85	COG3169	S:Function unknown	GO:0016021	CC:integral component of membrane;
BAB_RS18855	-	HK97 family phage prohead protease	0.999	-0.000881666	0.996535424	0.997466766	no	down	mRNA	10.84	8.22	8.27	9.88	8.58	6.97	9.11	8.47666667	COG3740	S:Function unknown	GO:0008233	MF:peptidase activity;
BAB_RS33180	-	hypothetical protein	1	-0.000533444	0.999089692	0.999400742	no	down	mRNA	0	22.15	43.18	40.72	15.67	36.91	21.7767	31.1	------	------	GO:0016021	CC:integral component of membrane;
