# Supplementary material for: Erythronate utilization activates VdtR regulating its metabolism to promote Brucella proliferation, inducing abortion in mice
Source: Microbiol Spectr. 2023 Sep 6;11(5):e02074-23. doi: 10.1128/spectrum.02074-23 (PMC10580937; doi:10.1128/spectrum.02074-23)
Supplement: Supplemental tables — Tables S1 and S2. [file spectrum.02074-23-s0003.pdf]

Table S1 Bacterial strains and plasmids used in this study

Strains or plasmids	Description	Sources
**Bacterial strains**		
B. abortus 2308	Wild-type strain; Smooth phenotype	CVCC
B. suis 1330	Wild-type strain; Smooth phenotype	CVCC
B. melitensis M5	Attenuated vaccine strain; Smooth phenotype	CVCC
B. melitensis 16M	Wild-type strain; Smooth phenotype	CVCC
E. coli DH5α	F^- φ80lacZΔM15Δ(lacZYA-argF)U169 recA1 endA1 hsdR17(r^-,m^+) phoA supE44 thi-1 gyrA96 relA1 λ^-	Invitrogen
2308Δ*vdtR*	A *BAB_RS27040* gene deletion mutant derived from *B. abortus* strain 2308	This study
2308Δ*vdtR*-Rev	Revertant strain harboring the pVdtR plasmid	This study
2308(pBBR1MCS)	2308 strain harboring the pBBR1MCS plasmid	This study
2308(gc-over)	*BAB_RS27025-BAB_RS27055* over-expressed strain in *B. abortus* 2308	This study
1330Δ*vdtR*	*vdtR* gene deletion mutant strain derived from *B. suis* 1330	This study
1330Δ*vdtR*-Sibling	Sibling revertant strain carrying the intact *vdtR* gene	This study
M5Δ*vdtR*	*vdtR* gene deletion mutant strain derived from *B. melitensis* M5	This study
M5Δ*vdtR*-Sibling	Sibling revertant strain carrying the intact *vdtR* gene	This study
16MΔ*vdtR*	*vdtR* gene deletion mutant strain derived from *B. melitensis* 16M	This study
16MΔ*vdtR*-Sibling	Sibling revertant strain carrying the intact *vdtR* gene	This study
2308Δ*otnK*	*otnK* gene deletion mutant strain derived from *B. abortus* 2308	This study
M5Δ*otnK*	*otnK* gene deletion mutant strain derived from *B. melitensis* M5	This study
16MΔ*otnK*	*otnK* gene deletion mutant strain derived from *B. melitensis* 16M	This study
1330Δ*otnK*	*otnK* gene deletion mutant strain derived from *B. suis* 1330	This study
2308Δ*eryA*	*eryA* gene deletion mutant strain derived from *B. abortus* 2308	This study
2308Δ*eryA-otnK*	*eryA* and *otnK* double gene deletion mutant strain derived from *B. abortus* 2308	This study
2308Δ*mfs*	*mfs* gene deletion mutant strain derived from *B. abortus* 2308	This study
2308(P_{ltnD}-LacZ)	2308 strain harboring the recombinant plasmid pMCR-P_{ltnD}-LacZ	This study
2308(P_{otnK}-LacZ)	2308 strain harboring the recombinant plasmid pMCR-P_{otnK}-LacZ	This study
2308(P_{otnI}-LacZ)	2308 strain harboring the recombinant plasmid pMCR-P_{otnI}-LacZ	This study
2308(P_{vdtR}-LacZ)	2308 strain harboring the recombinant plasmid pMCR-P_{vdtR}-LacZ	This study
2308(P_{denD}-LacZ)	2308 strain harboring the recombinant plasmid pMCR-P_{denD}-LacZ	This study
2308(P_{otnC}-LacZ)	2308 strain harboring the recombinant plasmid pMCR-P_{otnC}-LacZ	This

		study
2308(P_{mfs}-LacZ)	2308 strain harboring the recombinant plasmid pMCR-P_{mfs}-LacZ	This study
2308Δ*vdtR*(P_{ltnD}-LacZ)	2308Δ*vdtR* strain harboring the recombinant plasmid pMCR-P_{ltnD}-LacZ	This study
2308Δ*vdtR*(P_{otnK}-LacZ)	2308Δ*vdtR* strain harboring the recombinant plasmid pMCR-P_{otnK}-LacZ	This study
2308Δ*vdtR*(P_{otnI}-LacZ)	2308Δ*vdtR* strain harboring the recombinant plasmid pMCR-P_{otnI}-LacZ	This study
2308Δ*vdtR*(P_{vdtR}-LacZ)	2308Δ*vdtR* strain harboring the recombinant plasmid pMCR-P_{vdtR}-LacZ	This study
2308Δ*vdtR*(P_{denD}-LacZ)	2308Δ*vdtR* strain harboring the recombinant plasmid pMCR-P_{denD}-LacZ	This study
2308Δ*vdtR*(P_{otnC}-LacZ)	2308Δ*vdtR* strain harboring the recombinant plasmid pMCR-P_{otnC}-LacZ	This study
2308Δ*vdtR*(P_{mfs}-LacZ)	2308Δ*vdtR* strain harboring the recombinant plasmid pMCR-P_{mfs}-LacZ	This study
2308Δ*vdtR*-Rev(P_{ltnD}-LacZ)	2308Δ*vdtR*-Rev strain harboring the recombinant plasmid pMCR-P_{ltnD}-LacZ	This study
**Plasmids**		
pKB	Kan^R; pUC19 derived plasmid containing SacB gene	This study
pBBR1MCS	Cm^R; Broad host - range cloning vector	(1)
pUC19	AmpR; pUC cloning vector	Takara
pKB-Δ*vdtR*	The recombinant suicide plasmid for *vdtR* deletion	This study
pKB-*vdtR*	The revertant plasmid containing the intact *vdtR* gene	This study
pKB-Δ*otnK*	The recombinant suicide plasmid for *otnK* deletion	This study
pKB-Δ*eryA*	The recombinant suicide plasmid for *eryA* deletion	This study
pBBR1MCS-gc-over	The aldolase-DUF 1537 gene cluster over-expression plasmid	This study
pMCR-P_{ltnD}-LacZ	The recombinant plasmid for *ltnD* promoter activity evaluation	This study
pMCR-P_{otnK}-LacZ	The recombinant plasmid for *otnK* promoter activity evaluation	This study
pMCR-P_{otnI}-LacZ	The recombinant plasmid for *otnI* promoter activity evaluation	This study
pMCR-P_{vdtR}-LacZ	The recombinant plasmid for *vdtR* promoter activity evaluation	This study
pMCR-P_{denD}-LacZ	The recombinant plasmid for *denD* promoter activity evaluation	This study
pMCR-P_{otnC}-LacZ	The recombinant plasmid for *otnC* promoter activity evaluation	This study
pMCR-P_{mfs}-LacZ	The recombinant plasmid for *mfs* promoter activity evaluation	This study
pColdTF-VdtR	Plasmid for rVdtR protein expression	This study
pUC19-P_{ltnD}	Plasmid for construction of labeled probes Cy5.5-P_{ltnD}	This study
pUC19-Neg	Plasmid for construction of non-specific competition probes	This study
pUC19-P1	Plasmid for construction of labeled probes Cy5.5-P1	This study

pUC19-P2	Plasmid for construction of labeled probes Cy5.5-P2	This study
pUC19-P3	Plasmid for construction of labeled probes Cy5.5-P3	This study
pUC19-P2+P3	Plasmid for construction of labeled probes Cy5.5-P2+P3	This study

References

1. Kovach ME, Phillips RW, Elzer PH, Roop RM, 2nd, Peterson KM. 1994. pBBR1MCS: a broad-host-range cloning vector. Biotechniques 16:800-2.

Table S2 Primers used in this study

**Primers**	**Sequences (5′-3′)**
Primers for construction of deletion mutant and revertant mutant	
vdtR-UF	GGTACCCGGGGATCCACGCGCTTCATTGTTGCAGG
vdtR-UR	CGGCAATCACTCATGCGCTTTGATCCCGGT
vdtR-DF	AAGCGCATGAGTGATTGCCGCGTTCCTTTC
vdtR-DR	TGCCTGCAGGTCGACTCGCGGATTATGCCGCTTGC
otnK-UF	GGTACCCGGGGATCCGTCTTCGCACCTATGGCATC
otnK-UR	GAACAAATCTCAATGACGCCAAGGCGCATG
otnK-DF	GGCGTCATTGAGATTTGTTCGGGCAGCTTG
otnK-DR	TGCCTGCAGGTCGACCATTTTCTCGATCAGGCGGG
eryA-UF	GGTACCCGGGGATCCTTGGCCGGGTGGTGGGCTTG
eryA-UR	CCTCGCGGGCCGAAACGCGCTTCACGCATGGCTGACACAG
eryA-DF	CTGTGTCAGCCATGCGTGAAGCGCGTTTCGGCCCGCGAGG
eryA-DR	TGCCTGCAGGTCGACCGCGGCGTGCGGAGACGACC
mfs-UF	GGTACCCGGGGATCCTATTGCTGCCCGCAACCTCG
mfs-UR	TCGGCATCCACAGCGTGCTGTTTTTCATTGCTTTC
mfs-DF	CAATGAAAAACAGCACGCTGTGGATGCCGACGCTC
mfs-DR	TGCCTGCAGGTCGACCTGCGAATGGGCGTATGGTA
Primers for construction of over-expression strain	
gc-over-F	AGAACTAGTGGATCCGGAGTTTCCCGCCTTTACCG
gc-over-R	CAAAAGCTGGGTACCTGCAGATAAGTTGCGAATGG
Primers for co-transcriptional analysis	
F1-F	CCGATAAAGAACAGCCCTGC
F1-R	GTTGTTTCCGGCAATTCCCT
F2-F	CAGCCCTTGTCCAATTTGCT
F2-R	GTTCACGCAATCACTGTCGA
F3-F	GTGATGACTGAACGGGCAAA
F3-R	ATTCAATGCAATCCCGCTCG
F4-F	GATCGCCGCTTTCTCATTGT
F4-R	CCCGCCTGATCGAGAAAATG
F5-F	GACCCGAAATAGCTGCCATG
F5-R	AGATTTGTTCGGGCAGCTTG
F6-F	CGTTGCCAACCAGAAATCCT
F6-R	CGCATGAAACATGTGGTGGA
Primers for qPCR	
RT-*mfs*-F	CCTGAGGTGGTGGAGAACAT
RT-*mfs*-R	CCGATAAAGAACAGCCCTGC
RT-*otnC*-F	AGCAAATTGGACAAGGGCTG
RT-*otnC*-R	AATGGTGGGAAGGTGTTTGC
RT-*denD*-F	CTGGTTGGCGATTATTCCCG
RT-*denD*-R	TCGACAGTGATTGCGTGAAC
RT-*vdtR*-F	ACAATGAGAAAGCGGCGATC
RT-*vdtR*-R	CGCAGGATTGGTTTTCTCGT
RT-*otnI*-F	CCGGCTATTTCATCGTGCAT
RT-*otnI*-R	CATTTTCTCGATCAGGCGGG
RT-*otnK*-F	GAATTCACCGTCATCTGCCC
RT-*otnK* -R	AATATTTCCTGCACGTCCGC
RT-*ltnD*-F	TTTTGTCAAGGATCTGCGCC
RT-*ltnD*-R	CTGTCGGGAAGTTTGATGCC
RT-*BAB_RS27060*-F	GACGCAGCTTACCATGACTG

RT-*BAB_RS27060*-R	TCCTGTTCAACATCGACCGT
RT-*BAB_RS27065*-F	ACATTGAAGATCGCGGCATC
RT-*BAB_RS27065*-R	AGGTATCCTTGCCCACTTCC
RT-*BAB_RS27070*-F	GAAATTACAGGCGGCAGAGG
RT-*BAB_RS27070*-R	GAGATAGGCCTGCACGATCT
RT-*BAB_RS27815*-F	CCATCACCGACCTCAATGTC
RT-*BAB_RS27815*-R	TTGCCATGAACTTGCGTCAA
RT-*BAB_RS27820*-F	AACGCACTTCTCAATACGGC
RT-*BAB_RS27820*-R	CTTGTTTCCTTGCCCAGTCC
RT-*BAB_RS27825*-F	ATCTGGAAAGATGGGGAGGC
RT-*BAB_RS27825*-R	TCCGTGCCGATGTTGAAATC
RT-*BAB_RS27830*-F	AATTCCGGCCTGATGATTGC
RT-*BAB_RS27830*-R	GCATTGAGATTGGCGGTCAT
RT-*BAB_RS27835*-F	TTCAAGCACCGTTTTCACCC
RT-*BAB_RS27835*-R	CCAGTGCAGCTTCTTCGATC
RT-*BAB_RS27840*-F	TCCATTGAGACAGGCGTTCT
RT-*BAB_RS27840*-R	CCCGCCATGCCATATTGAAA
RT-*BAB_RS29675*-F	AATTATGTGAAGGACGCGCC
RT-*BAB_RS29675*-R	TCGTTCAGTCTGGCCTTGAT
RT-*BAB_RS29680*-F	CTTTTGAAGGAAGCAGGCGT
RT-*BAB_RS29680*-R	TAATCTCCGATCCAGCCGTC
RT-*BAB_RS29685*-F	TCTGTTGCATGGCGATCTTG
RT-*BAB_RS29685*-R	CGAGGAAAGTGCCGATGATG
RT-*BAB_RS29690*-F	CTTGAACAGGTATTGGCCCG
RT-*BAB_RS29690*-R	ATTCATCGTCAGTTTGCCGG
RT-*BAB_RS29695*-F	CCCGCACAATGATCCTGAAG
RT-*BAB_RS29695*-R	AGTCCGTGATCCTTCTGCAA
RT-*BAB_RS33230*-F	CGTGGAAATTGACCGAGACC
RT-*BAB_RS33230*-R	CAGGTCTATTTTCGCCAGCC
RT-*BAB_RS19575*-F	AATTCAAAGAAGCTGGCGGG
RT-*BAB_RS19575*-R	ATAGTCGGCCATGTTCAGCT
RT-*BAB_RS21730*-F	GCAAACATCAAATCGGTCGC
RT-*BAB_RS21730*-R	GCCCACAAAGAAATAGGCGT
RT-*BAB_RS24660*-F	CAATCGAACTCTGGGCCAC
RT-*BAB_RS24660*-R	AGGAAATGAGCCAGACGATGA
RT-*BAB_RS24665*-F	TGAATTCGGCAATGGTGTGG
RT-*BAB_RS24665*-R	GCCGCCAAAGGTGTAATAGG
RT-*BAB_RS27685*-F	GAAGCTGGCGGAAAAGACAA
RT-*BAB_RS27685*-R	ATTGATCGATGAACAGCCGC
RT-*BAB_RS27690*-F	GCTTGTCTTCCAGTTCGCAA
RT-*BAB_RS27690*-R	ATGTTGCAAGCCAGTTCTGG
RT-*BAB_RS27700*-F	TATGAAGGGCTGGGCTTTGT
RT-*BAB_RS27700*-R	AGAGGTTGGGGAAGAGTTCG
RT-*BAB_RS27710*-F	AAATGGTCGTATGAAGCGGC
RT-*BAB_RS27710*-R	GCTTGTTCTGATGGTCTGCC
RT-16S-F	ACGTGCTACAATGGTGGTGA
RT-16S-R	CAGAGTGCAATCCGAACTGA
RT-β-actin-F	AGAGGGAAATCGTGCGTGAC
RT-β-actin-R	CAATAGTGATGACCTGGCCGT
FAM-β-actin	CACTGCCGCATCCTCTTCCTCCC
RT-TNFα-F	CATCTTCTCAAAATTCGAGTGACAA
RT-TNFα-R	TGGGAGTAGACAAGGTACAACCC
FAM-TNFα	CACGTCGTAGCAAACCACCAAGTGGA
RT-IL1β-F	CAACCAACAAGTGATATTCTCCATG
RT-IL1β-R	GATCCACACTCTCCAGCTGCA

FAM-IL1β	CTGTGTAATGAAAGACGGCACACCCACC
RT-IP10-F	GCCGTCATTTTCTGCCTCAT
RT-IP10-R	GCTTCCCTATGGCCCTCATT
FAM-IP10	TCTCGCAAGGACGGTCCGCTG
RT-MIP1β-F	TTCCTGCTGTTTCTCTTACACCT
RT-MIP1β-R	CTGTCTGCCTCTTTTGGTCAG
FAM-MIP1β	TGCTCCAAGCCAGCTGTGGT
Primers for promoter activity analysis	
P_0-F	CGGGATCCACCATGATTACGGATTCACTGG
P_0-R	GCTCTAGATTATTTTTGACACCAGACCAAC
P_{mfs}-F	CTCTGGTAGGGGTACCCCTATTTCGTGATGCGGATC
P_{mfs}-R	CCGTAATCATGGTGGATCCCATTGCTTTCACTCCCGGAA
P_{otnC}-F	CTCTGGTAGGGGTACCCGCAATCACTGTCGAAGGTT
P_{otnC}-R	CCGTAATCATGGTGGATCCCATGTTCTTTGTTCCAAATT
P_{denD}-F	CTCTGGTAGGGGTACCCTCGCATTCATCTCGACATC
P_{denD}-R	CCGTAATCATGGTGGATCCCATCAGATTTTCCCTTCGAC
P_{vdtR}-F	CTCTGGTAGGGGTACCCAACCGCCCCAATGTCGCCG
P_{vdtR}-R	CCGTAATCATGGTGGATCCCATGCGCTTTGATCCCGGTA
P_{otnI}-F	CTCTGGTAGGGGTACCGCAGGAGCGATTTGGTGGCG
P_{otnI}-R	CCGTAATCATGGTGGATCCCATTATTTATCTCCTTCCTG
P_{otnK}-F	CTCTGGTAGGGGTACCTCCGCTCGACATCATGTATG
P_{otnK}-R	CCGTAATCATGGTGGATCCCATGTCATGCACTCCTGTCG
P_{ltnD}-F	CTCTGGTAGGGGTACCGGAGTTTCCCGCCTTTACCG
P_{ltnD}-R	TCCGTAATCATGGTGGATCCCATAATCACTCCGTGCGTTTATG
Primers for EMSA	
rVdtR-F	GGCATATGGAGCTCGGTACCATGATACCGGCTGAACGGCAGG
rVdtR-R	AGGTCGACAAGCTTGAATTCTCAGATTTTCCCTTCGACTTTGC
Cy5.5-pUC19-F	AGCTCGGTACCCGGGGATCC
Cy5.5-pUC19-R	TTGCATGCCTGCAGGTCGAC
Neg-F	GGTACCCGGGGATCCGCAAGCTGACCCTGAAGTTC
Neg-R	TGCCTGCAGGTCGACGTCTTGTAGTTGCCGTCGTC
Cy5.5-P1-F	GGTACCCGGGGATCCGGAGTTTCCCGCCTTTACCG
Cy5.5-P1-R	TGCCTGCAGGTCGACGGCAATTGCAGATTGCTGCC
Cy5.5-P2-F	GGTACCCGGGGATCCGGCGCGGTTCCACAGGCGTC
Cy5.5-P2-R	TGCCTGCAGGTCGACAAACCACCGGGTATGAGCGG
Cy5.5-P3-F	GGTACCCGGGGATCCCCTTAATAGTTTGAATTTCC
Cy5.5-P3-R	TGCCTGCAGGTCGACAATCACTCCGTGCGTTTATG
Cy5.5-P4-F	CCTTAATAGTTTGAATTTCC
Cy5.5-P5-F	CCGCTCATACCCGGTGGTTTG
Cy5.5-P6-F	GTCCGCGCCCGGTTCGTACC
Cy5.5-P7-F	ATATATCGCTTTTTTATGAG
Cy5.5-P8-F	AAAATATGTTCATAATCGAATTG
